# A Family of Chemoreceptors in *Tribolium castaneum* (Tenebrionidae: Coleoptera)

**DOI:** 10.1371/journal.pone.0001319

**Published:** 2007-12-19

**Authors:** Mohatmed Abdel-latief

**Affiliations:** Department of Applied Zoology, Animal Ecology, Institute of Biology, Freie Universität Berlin, Berlin, Germany; The Rockefeller University, United States of America

## Abstract

Chemoperception in invertebrates is mediated by a family of G-protein-coupled receptors (GPCR). To date nothing is known about the molecular mechanisms of chemoperception in coleopteran species. Recently the genome of *Tribolium castaneum* was sequenced for use as a model species for the Coleoptera. Using blast searches analyses of the *T. castaneum* genome with previously predicted amino acid sequences of insect chemoreceptor genes, a putative chemoreceptor family consisting of 62 gustatory receptors (Grs) and 26 olfactory receptors (Ors) was identified. The receptors have seven transmembrane domains (7TMs) and all belong to the GPCR receptor family. The expression of the *T. castaneum* chemoreceptor genes was investigated using quantification real- time RT-PCR and *in situ* whole mount RT-PCR analysis in the antennae, mouth parts, and prolegs of the adults and larvae. All of the predicted *Tcas*Grs were expressed in the labium, maxillae, and prolegs of the adults but *Tcas*Gr13, 19, 28, 47, 62, 98, and 61 were not expressed in the prolegs. The *Tcas*Ors were localized only in the antennae and not in any of the beetles gustatory organs with one exception; the *Tcas*Or16 (like *Dmel*Or83b), which was localized in the antennae, labium, and prolegs of the beetles. A group of six *Tcas*Grs that presents a lineage with the sugar receptors subfamily in *Drosophila melanogaster* were localized in the lacinia of the *Tribolium* larvae. *Tcas*Gr1, 3, and 39, presented an ortholog to CO_2_ receptors in *D. melanogaster* and *Anopheles gambiae* was recorded. Low expression of almost all of the predicted chemoreceptor genes was observed in the head tissues that contain the brains and suboesophageal ganglion (SOG). These findings demonstrate the identification of a chemoreceptor family in *Tribolium*, which is evolutionarily related to other insect species.

## Introduction

In living animals the chemical senses are important to detect various environmental chemical informations. The insect gustatory and olfactory systems play important roles in development and reproduction (the mate partner or an oviposition site) as well as in searching for host plants [1]. Insects are covered with chemosensory structures known as sensilla, were sensory neurons for olfactory stimuli and taste are located. Chemosensory sensilla are present on the antennae, mouth parts, legs, wings and the ovipositor [2]. Most of the olfactory receptor neurons (ORNs) are located on the antennae; whereas the gustatory neurons are present on the mouth parts and the legs [2]. In vertebrates, chemosensation is mediated by a large family of G-protein-coupled receptors (GPCR) of the rhodopsin-related receptor superfamily, which are known as odorant receptors [3]. Receptors that belong to the GPCR families T2R and T1R mediate the sensing of sweet and bitter tastes and are known as gustatory receptors (Gr) in vertebrates. The vertebrate chemosensory receptor superfamily is evolutionarily distant from the invertebrate chemosensory receptor family [3].

Genome analysis of *Drosophila melanogaster* genome elucidated the presence of 62 olfactory receptors (Or), which are encoded by 60 genes, and 68 gustatory receptors (Gr), which are also encoded by 60 genes [4], [5], [6], [7], [8], [9]. From the genome of *Anopheles gambiae* 79 olfactory receptors and 76 gustatory receptors were identified [10]. The genome of *Apis mellifera* encoded 170 Or and 10 Gr receptor genes [11] and *Bombyx mori* genome encoded 41 olfactory receptors [12], 17 of which appear to be orthologs of *Helicoverpa virescens* [13], [14]. Recently the genome of *Aedes aegypti* was found to contains genes for 131 Or receptors, and 88 gustatory receptors [15]. In the last few years much had been made in understanding the molecular and neurological mechanism of insect chemoperception, especially in *D. melanogaster*, including the fact that the number of glomerulus's is almost in a ratio of 1:1 to Or receptors [16], [1]. Using this information it is now possible to predict the number of olfactory receptors in different insect species.

The red flour beetle, *Tribolium castaneum* is an insect pest that belongs to the Tenebrionidae family within the order Coleoptera. It presents the most destructive species of stored product insects. It attacks stored grain products, dried pet food, dried flowers, chocolate, nuts, seeds, and even dried museum specimens [17]. *Tribolium* beetles are also considered as secondary pests, where they infest previously damaged and addicted grains [18]. Recently, the genome of *T. castaneum* has been sequenced to 7-fold coverage using a whole genome shotgun approach and assembled using the HGSC's assembly engine Atlas (http://www.hgsc.bcm.tmc.edu/projects/tribolium/).

The present paper describes the molecular characterization of a chemoreceptor family in *T. castaneum.* The receptor gene sequences provide novel information to study their molecular evolution in relation to other insect chemoreceptor gene family. The molecular data have allowed studies on the expression of receptor gene transcripts in various tissues of the beetles to be conducted, which may help to elucidate their physiological significance.

## Results

### Prediction of *T. castaneum* chemoreceptors

Using blast searches of the genome sequence of *Tribolium castaneum*, a family of 88 receptors was identified (Table 1 and Figure S1A). All of the predicted proteins were found to share a common structure of seven transmembrane domains, belonging to the G protein-coupled receptors (GPCRs) (Figure S1B). Sixty two of the genes showed sequence similarity to the gustatory receptor families of *Drosophila melanogaster* [5], *Anopheles gambiae* [10], *Apis mellifera* [11], and *Aedes aegypti* (unpublished data) with 6 to 13% identity (Figure S1C). Most receptors of the insect Gr receptors also found to share a signature motif with a *PKFSAGFFDIDRTLLFSIFGAITTYLIILIQF* amino acid sequences in the putative seventh transmembrane domain at the C-terminus (Figure S2).

**Table 1:** List of all identified chemoreceptors genes in *T. castaneum*

Number	Gene bank accession Number	Gene proposed name	Map position	Number of introns	Size (amino acids)
1	AM292322	*Tcas*Gr10	7	3	373
2	AM292323	*Tcas*Gr2	5	6	586
3	AM292324	*Tcas*Gr38	7	2	398
4	AM292325	*Tcas*Gr4	2	1	309
5	AM292326	*Tcas*Gr5	7	3	522
6	AM292327	*Tcas*Gr6	4	2	387
7	AM292328	*Tcas*Gr7	5	7	429
8	AM292329	*Tcas*Gr104	7	3	379
9	AM292330	*Tcas*Gr9	5	5	408
10	AM292331	*Tcas*Gr1	7	1	437
11	AM292332	*Tcas*Gr11	7	2	344
12	AM292333	*Tcas*Gr12	Un	2	316
13	AM292334	*Tcas*Gr13	Un	2	390
14	AM292335	*Tcas*Gr14	7	1	374
15	AM292336	*Tcas*Gr15	5	5	455
16	AM292337	*Tcas*Gr16	10	2	452
17	AM292338	*Tcas*Gr17	10	3	372
18	AM292339	*Tcas*Gr150	10	2	387
19	AM292340	*Tcas*Gr19	7	2	355
20	AM292341	TcasGr20	6	4	393
21	AM292342	*Tcas*Gr21	5	4	385
22	AM292343	*Tcas*Gr22	10	1	301
23	AM292344	*Tcas*Gr79	Un	4	291
24	AM292345	*Tcas*Gr123	7	1	384
25	AM292346	*Tcas*Gr25	Un	0	314
26	AM292347	*Tcas*Gr26	4	2	387
27	AM292348	*Tcas*Gr27	6	4	346
28	AM292349	*Tcas*Gr28	5	3	250
29	AM292350	*Tcas*Gr29	9	7	429
30	AM292351	*Tcas*Gr30	5	6	394
31	AM292352	*Tcas*Gr31	5	4	250
32	AM292353	*Tcas*Gr32	7	4	651
33	AM292354	*Tcas*Gr47	6	1	321
34	AM292355	*Tcas*Gr34	6	1	324
35	AM292356	*Tcas*Gr35	6	1	251
36	AM292357	*Tcas*Gr105	7	1	355
37	AM292358	*Tcas*Gr37	7	3	331
38	AM292359	*Tcas*Gr3	5	2	436
39	AM292360	*Tcas*Gr39	7	1	437
40	AM292361	*Tcas*Gr40	5	1	373
41	AM292362	*Tcas*Gr41	Un	1	398
42	AM292363	*Tcas*Gr71	6	5	347
43	AM292364	*Tcas*Gr43	8	2	353
44	AM292365	*Tcas*Gr44	2	1	313
45	AM292366	*Tcas*Gr45	4	3	379
46	AM292367	*Tcas*Gr46	7	7	1451
47	AM292368	*Tcas*Gr33	4	2	387
48	AM292369	*Tcas*Gr48	Un	2	408
49	AM292370	*Tcas*Gr49	7	1	418
50	AM292371	*Tcas*Gr50	7	3	350
51	AM292372	*Tcas*Gr51	7	5	771
52	AM292373	*Tcas*Gr54	7	2	360
53	AM292374	*Tcas*Gr62	7	4	659
54	AM292375	*Tcas*Gr52	4	3	311
55	AM292376	*Tcas*Gr125	7	3	402
56	AM292377	*Tcas*Gr56	5	4	358
57	AM292378	*Tcas*Gr57	2	3	387
58	AM292379	*Tcas*Gr98	4	4	376
59	AM292380	*Tcas*Gr59	Un	0	489
60	AM292381	*Tcas*Gr60	10	4	364
61	AM292382	*Tcas*Or61	7	7	670
62	AM292383	*Tcas*Or53	7	3	319
1	AM689931	*Tcas*Or1	7	2	353
2	AM689904	*Tcas*Or2	7	0	314
3	AM689905	*Tcas*Or3	4	2	413
4	AM689906	*Tcas*Or4	2	0	378
5	AM689907	*Tcas*Or5	2	0	299
6	AM689908	*Tcas*Or6	8	5	475
7	AM689909	*Tcas*Or7	2	1	311
8	AM689910	*Tcas*Or63	9	4	383
9	AM689911	*Tcas*Or9	9	1	294
10	AM689912	*Tcas*Or10	8	1	330
11	AM689913	*Tcas*Or11	8	0	332
12	AM689914	*Tcas*Or12	2	2	374
13	AM689915	*Tcas*Or13	6	5	405
14	AM689916	*Tcas*Or56	10	5	433
15	AM689917	*Tcas*Or15	10	1	340
16	AM689918	*Tcas*Or16	6	4	475
17	AM689919	*Tcas*Or17	8	3	243
18	AM689920	*Tcas*Or18	8	1	410
19	AM689921	*Tcas*Or19	9	0	331
20	AM689922	*Tcas*Or20	10	1	386
21	AM689923	*Tcas*Or21	8	0	240
22	AM689924	*Tcas*Or22	9	1	382
23	AM689925	*Tcas*Or23	9	4	360
24	AM689926	*Tcas*Or24	9	0	343
25	AM689927	*Tcas*Or25	9	6	791
26	AM689928	*Tcas*Or26	9	0	338

Un = Chromosome LGUn

Another 26 identified genes showed sequence similarity to the olfactory receptor families of *D. melanogaster* [4], [6], A. *gambiae* [10], *A. mellifera* [11], *Bombyx mori* [12], *Helicoverpa virescens* [13], [14], and *A. aegypti* [15] with about 15% homology (Figure S1D). The receptor gene family was code named *T. castaneum* olfactory receptor family (*Tcas*Or).

### Genome analyses

The *Tcas*Gr receptors amino acid sequence lengths are extremely diverse. They range from 250 aa (*Tcas*Gr28) to 1451 aa in *Tcas*Gr46 (Table 1). The mean length of the *Tcas*Grs is about 412 aa. Within the predicted *Tcas*Gr receptors four groups consisting of more than ten members were found to have at least 1-4 introns (Table 1). Another three groups with two to four members were found to contain 5-7 introns, and in one group of two receptors no introns were found. The *Tcas*Or receptors also varied in their aa lengths (240 aa for *Tcas*Or21 to 791 aa for *Tcas*Or25). The mean length of the *Tcas*Or receptors was about 374 aa (Table 1). Eight of the predicted *Tcas*Or receptors contained no introns. Seven receptors had one intron each and two groups, each consists of three receptors had two and five introns each, respectively. One group of three receptors had four introns each (Table 1), TcasOr17 had three introns, and the *Tcas*Or25 had six introns. 64 of the *T. castaneum* chemoreceptor genes were found to contain a conserved intron near the carboxyl terminus (Table 1).

24 receptor genes including 22 *Tcas*Gr genes and two *Tcas*Or genes were located on chromosome 7 (Table 1 and Figure S3). The evolutionary relationships of these genes, based on their amino acid sequences, showed that they arrange in five subfamilies consisting of eight, two, four, seven and two proteins (Figure S3). A weak bootstrap value supported the evolutionary relationships between the different families. The *Tcas*Gr19 gene does not belong to any of the subfamilies. 7, 7, 11, 8, 7, 9, and 8 receptor genes of *Tcas*Gr and Or receptors were localized on the chromosomes two, four, five, six, eight, nine and ten, respectively (Table 1). Seven of the predicted *Tcas*Gr genes were localized on the Un chromosome. There was not suggestion that any of the predicted genes was a pseudogene, as none were interrupted by a stop codon nor were any frame shift mutations detected (Table 1). GPCRHMM protein analysis of all the putative *T. castaneum* chemoreceptors family showed that only the *Tcas*Gr19 and *Tcas*Gr40 are true GPCR proteins (Table 2 and S2). Within chemoreceptors from other insect species, *Amel*Gr5 from, *A. mellifera* is also a true GPCR protein (Table 2 and S2).

**Table 2:** List of insect chemoreceptors that belong to the GPCR superfamily based on GPCRHMM analysis.

Sequence identifier		Global	Local	Prediction
Proposed name	Accession number			
*Tcas*Gr19	(AM292340)	60.36	75.14	GPCR
*Tcas*Gr40	(AM292361)	3.34	15.90	GPCR
*Amel*Gr5		6.61	15.71	GPCR

For other details see Figure S2.

### Phylogenetic analysis of *T. castaneum* chemoreceptor family

**Figure 1:**
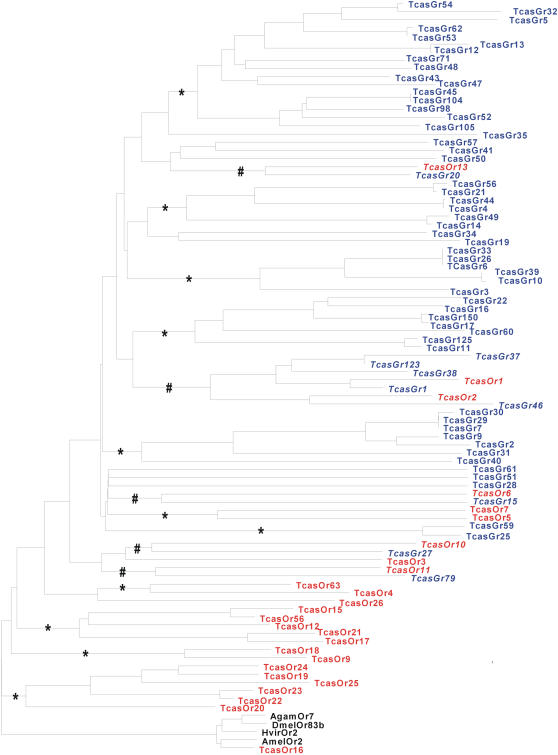
Neighbor joining tree of *T. castaneum* chemoreceptor genes family. The *T. castaneum* putative chemoreceptor genes (*Tcas*Gr and *Tcas*Or) are indicated to the right. The corrected distance tree was rooted by declaring the *Tcas*Or16 and its homologs (*Dmel*Or83b, *Amel*Or2, *Agam*Or7, and *Hvir*Or2) as outgroup, based on their position at the base of the insect Or family in the phylogenetic analysis of [11]. Receptors that represent lineages are indicated in bold and italics, and the supported bootstrap value (>50%) is indicated with #. * represents lineages of either *Tcas*Gr or *Tcas*Or receptors supported with a bootstrap value of >50%. *Tcas*Gr are written in blue letters, *Tcas*Or are written in red, and the members of *Tcas*Or16 outgroup are written in black. The amino acid sequences alignment was carried out using CLUSTAL X [47]. The neighbour joining tree was produced using the PHYLIP package [48] and based on the consensus of 1000 bootstrap replicates. Tree drawing was performed as corrected-distance cladogram with the help of TreeView 1.6.6. program.

The entire chemoreceptor family of *T. castaneum* consists of 88 putative receptors (Figure S1A). Alignment analysis of the *T. castaneum* chemoreceptor gene family (*Tcas*Gr and *Tcas*Or) indicated that the family members share a high degree of sequence divergence (Figure S1C, S1D). Phylogenetic analysis revealed the presence of five distinct lineages of *TcasGr* and *TcasOr* receptor subfamilies (Figure 1). One lineage containing seven chemoreceptors (*Tcas*Gr37, 123, 38, 1, and 46, and *Tcas*Or1 and 2) was identified. Another four lineages each consisting of two receptors, *Tcas*Or13 and *Tcas*Gr20, *Tcas*Or6 and *Tcas*Gr15, *Tcas*Or10 and *Tcas*Gr27, and *Tcas*Or11 and *Tcas*Gr79, respectively, (Figure 1) were found. The *Tcas*Gr receptors alone represented six lineages of proteins, and the *Tcas*Or receptors formed five lineages of proteins (Figure 1). The *Tcas*Or16 displayed a lineage with its homologs from other insect species (*Dmel*Or83b, *Amel*Or2, *Hvir*Or2, and *Agam*Or7) (Figure 1).

### Phylogenetic analysis of the insect gustatory receptor (Gr) family

The genome of *T. castaneum* encoded at least 62 Gr genes compared to 60 Gr genes in *D. melanogaster* [9], 52 Gr genes in *A. gambiae*, [10], 10 Gr genes in *A. mellifera* [11], and 88 Gr genes in *A. aegypti* (unpublished data). The 62 *T. castaneum* Gr genes showed thirteen lineages in a phylogenetic analysis of all known insect Grs (Figure S4). Based on the role of *Dmel*Gr5a as a trehalose receptor, six of the *T. castaneum* Gr receptors, *Tcas*Gr2, *Tcas*Gr7, *Tcas*Gr9, *Tcas*Gr29, *Tcas*Gr30, and *Tcas*Gr31, were clustered confidently with eight sugar receptors each of *D. melanogaster* and *A. gambiae*, nine sugar receptor candidates of *A. aegypti* (unpublished data), and two (Gr1 and 2) of *A. mellifera* [9], [19], [11]. An ortholog for the highly conserved lineage of the *Dmel*Gr21a, *Dmel*Gr63a, and *Agam*Gr22-24 proteins was also identified. The *Tcas*Gr1 and *Tcas*Gr39 clustered confidently with *Dmel*Gr21a, *Agam*Gr22, and *Aaeg*Gr21b, respectively. The *Tcas*Gr3 represented an ortholog with *Agam*Gr24, *Dmel*Gr63a, and *Aaeg*Gr63a (Figure S4). *Tcas*Gr6, *Tcas*Gr26 and *Tcas*Gr33 clustered confidently with the *Aaeg*Gr21a and *Agam*Gr23 proteins. None of the *Amel*Grs clustered within these orthologs (Figure S4). There was a weak bootstrap value supporting the lineage of *Tcas*Gr10, *Tcas*Gr46, *Tcas*Gr38, *Tcas*Gr62, and *Tcas*Gr123 as being orthologs to *Amel*Gr3, *Dmel*Gr43a, *Agam*Gr25 and *Aaeg*Gr43a (Figure S4). The remaining *Tcas*Gr lineages had no apparent orthologs to any other known insect Gr lineage. Within the *Tcas*Gr receptors, alternative splicing relationships between *Tcas*Gr2 and *Tcas*Gr9, *Tcas*Gr7, *Tcas*Gr29 and *Tcas*Gr30, *Tcas*Gr6, *Tcas*Gr26 and *Tcas*Gr33, and *Tcas*Gr17 and *Tcas*Gr150 existed (data not shown).

### Phylogenetic analysis of insect olfactory receptor (Or) families

Phylogenetic analysis of the proteins encoded by the 26 *Tcas*Or receptor genes, along with the Ors of *D. melanogaster* [5], [6], *A. gambiae* [10], *A. mellifera* [11], *H. virescens* [13], [14], *B. mori* [12], and *A. aegypti* [15] showed that they comprised six lineages (Figure S5). Three lineages consisted of four, two, and five *Tcas*Or receptors, respectively, and had no apparent orthologs in any other insect species identified (Figure S5). One subfamily was extraordinarily expanded to 18 Or receptors including *Tcas*Or9 and *Tcas*Or18 together with 16 members of *Amel*Or (*Amel*Or142-148 and *Amel*Or150-158) as orthologs (Figure S5). Another subfamily represents the only orthologs between *Tcas*Or and the lepidopteran Ors, where the *Tcas*Or13 forms a strong lineage with *Bmor*Or13, *Hvir*Or4, *Hvir*Or1, and *Hvir*Or5. No relationships between *Tcas*Or and any Or receptors of flies was detected with one exception: *Tcas*Or16, which was show to be an ortholog with *Dmel*Or83b, *Agam*Or7, *Amel*Or2, *Aaeg*Or2, *Bomr*Or2 and *Bmor*Or2a (Figure S5). Alignment analysis also showed that *Tcas*Or16 had a 75% identity in its amino acid sequences to *Dmel*Or83b (data not shown).

### Quantification real time RT-PCR analysis

**Figure 2:**
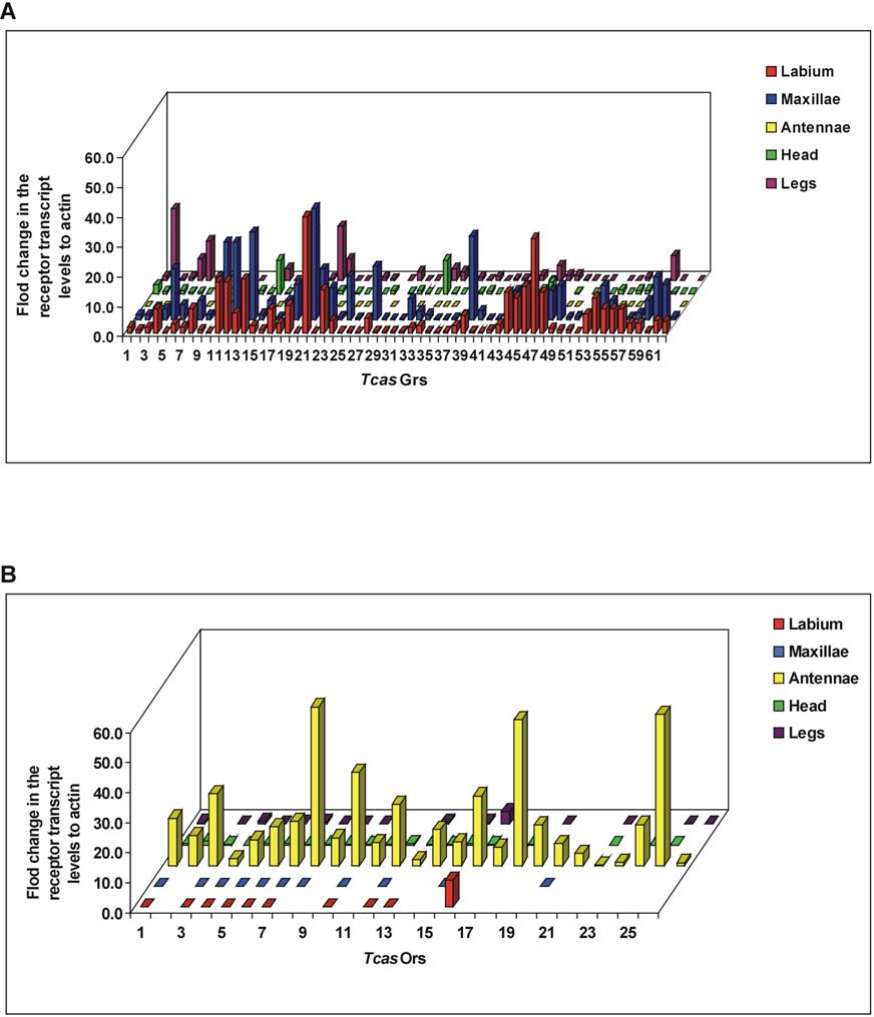
Quantitative real time RT-PCR analysis of *T. castaneum* chemoreceptor genes family. It shows the relative transcription levels of (A) *Tcas*Gr receptor genes, and (B) *Tcas*Or receptor genes in the labium, maxillae, antennae, heads (without gustatory and olfactory organs), and prolegs of adult *T. castaneum*. The genes are encoded on X axon with numbers as described in Table 1. The data on Y axon represented mean±SD normalized relative to the actin related protein transcript levels. All samples were run in triplicate and the entire assay was performed twice for each biological pool.

Tissue-specific expression patterns of the *T. castaneum* chemoreceptor genes were determined by quantitative (Q) real time RT-PCR analysis. The amplification efficiency of each primer set was validated; standard curves (5× serial dilutions starting with 2 ng/µl RNA concentration) yielded regression lines with r^2^ values >0.97 and slopes ranging from 3.07–3.20 (a slope of 3.13 indicates 100% amplification efficiency). Q-RT-PCR analysis revealed that the 62 *Tcas*Gr genes were expressed as a single transcript in *T. castaneum* labium and maxillae tissues (gustatory organs of the mouth parts) (Figure 2A). 55 of the putative *Tcas*Gr genes were also expressed in the femur, tibia, and tarsus of the adult *T. castaneum* prolegs (Figure 2A); while seven of the predicted genes were not expressed in the adult prolegs (Figure 2A). The receptors genes were also expressed to a low level in the head tissues [brain- suboesophageal ganglion (SOG)].

For the *Tcas*Or receptor genes the amplification efficiency of the respective primer sets was validated as described above and the r^2^ values were >0.98 and slopes ranged from 3.04–3.12 (a slope of 3.08 indicates 100% amplification efficiency). All the *Tcas*Or receptors were expressed as a single copy in the adult antennae (olfactory organ) (Figure 2B). The *Tcas*Or16 was also expressed in the labium and the prolegs of adult beetles (gustatory tissues) (Figure 2B). Expression of the *Tcas*Or receptor genes in head tissues (brain-SOG) was also recorded.

### Tissues specific localization using *in situ* whole mount RT-PCR analysis

**Figure 3:**
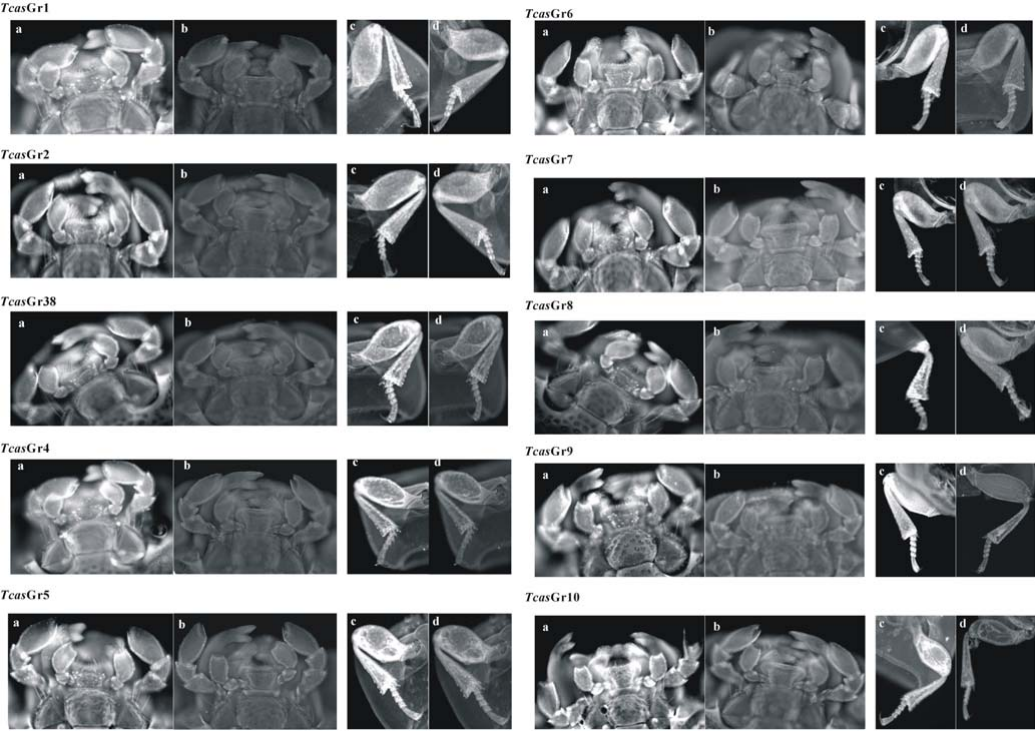
Tissue specific localization of the T. castaneum Gr receptors using whole-mount in situ RT-PCR analysis. The TcasGr1, 2, 38, and 4–10 candidates were localized in the labium and maxillae of the adult beetles, but also in the femur, tibia and tarsus of the prolegs. (a) shows the expression of the TcasGr receptors in the labium and maxillae of adult mouth parts. (c) shows the expression of the TcasGr receptors in the femur, tibia, and tarsus of adult prolegs. (b) and (d) show the sense negative controls. The TcasGr mRNAs were labeled using Dig 11-dUTP (Roche) and visualized by fluorescence in situ RT-PCR analysis using HNPP/Fast Red TR (Roche).

**Figure 4:**
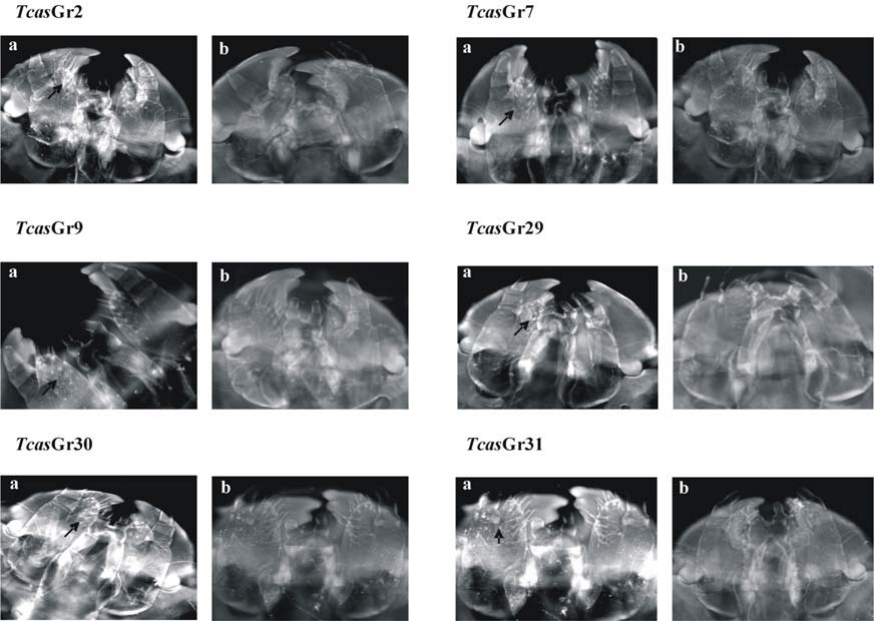
Tissue specific localization of T. castaneum Gr sugar receptor candidates in the head of larval using whole-mount in situ RT-PCR analysis. The receptors were localized in the lacina of the maxillae. (a) shows the antisense mRNA expression. (b) shows the sense negative controls. For other details see Figure 3.

**Figure 5:**
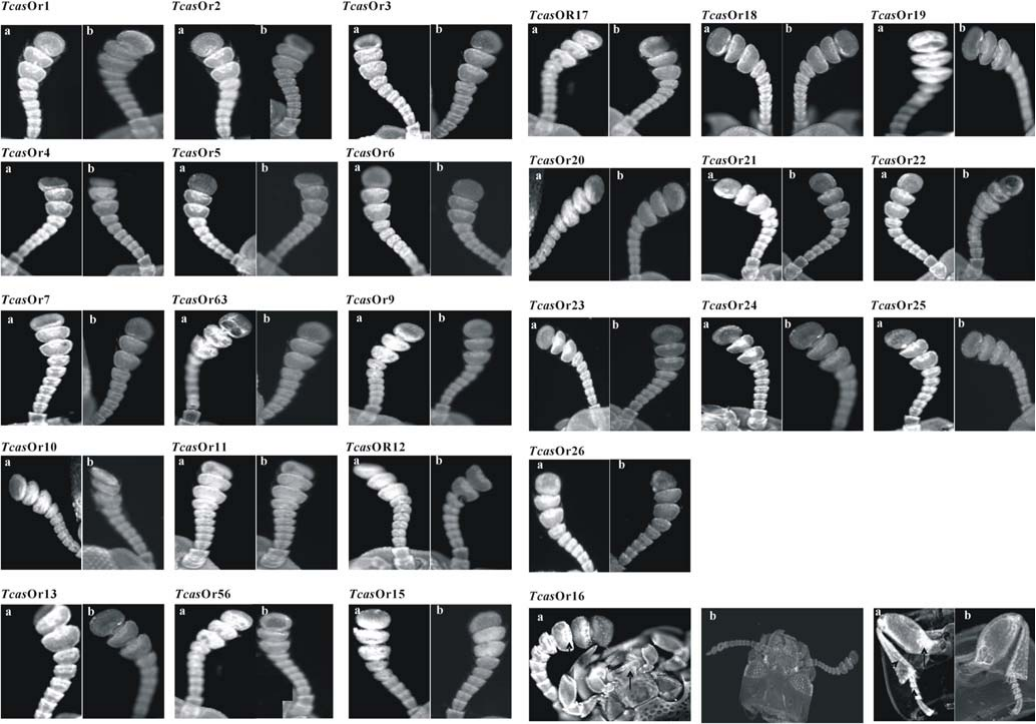
Tissue specific localization of the *T. castaneum* Or receptor genes in the antennae of adult beetles using whole-mount *in situ* RT-PCR. (a) shows the antisense mRNA expression. (b) shows the sense negative controls. The last line shows the expression of the *Tcas*Or16 receptor (homolog to *Dmel*Or83b) in the antennae, mouth parts (labium and maxillae), and in the prolegs (femur, tibia, and tarsus) of adult beetles. For other details see Figure 3.

Whole mount *in situ* RT-PCR analysis showed that all the putative *Tcas*Gr receptor genes were localized in the labium and maxillae of adult *Tribolium* (Figures 3 and S6). 55 of the *Tcas*Gr were also localized in the femur, tibia, and tarsus of the adult prolegs (Figures 3 and S6). During the last instar larvae, *Tcas*Gr2, 7, 9, 29, 30, and 31, which are candidates for sugar receptors, as shown in the phylogenetic analysis (Figure S4), were localized in the lacinia of the maxillae (Figure 4). 25 members of *Tcas*Or genes were localized in the antennae of adult beetles (Figure 5), and the *Tcas*Or16 was localized not only in the antennae but also in the labium, maxillae, and prolegs of the beetles (Figure 5). The negative controls using the sense RNA probes did not show any positive signal in the tissues studied (Figures 3, 4, 5, and S6).

## Discussion

Taken together the data in this study characterizes a chemoreceptor family *in T. castaneum,* which consists of 88 receptors. 62 are gustatory receptors (*Tcas*Grs), which are expressed mainly in gustatory organs of the beetle's mouth parts and the prolegs. Another 26 are olfactory receptors (*Tcas*Ors) that are localized only in the antenna; while the *Tcas*Or16 (homolog to *Dmel*Or83b) was localized in gustatory and olfactory organs of *Tribolium*. In previous studies, members of *Dmel*Gr receptors were localized in the labellum, pharynx, legs, and anterior wing margins of *D. melanogaster* [5], and the *Dmel*Ors were localized mainly in the antennae of the fly [6]. In *A. aegypti* 64 Or receptors were expressed in the antennae of the mosquitos [10]. In *B. mori* all of the 41 *Bmor*Or genes were localized in the antennae [12].

In this paper I have showed that *T. castaneum* has at least 62 gustatory receptors and 26 olfactory receptors. However, since conservation of these gene families was extremely low (6–15%), the number of chemoreceptors might be underestimated. This phenomenal was previously recorded in other insect species: For example [5] first characterized a family of 42 chemoreceptors genes, 19 of them being gustatory receptors. Later [7] and [8] extended the family to 54 and 56, respectively. Afterwards [9] extended the chemoreceptor family in *Drosophila* to 130 receptors of 68 Grs and 62 Ors. The *Tribolium* genome has at least 200 Gr receptors and 250 Or receptors, where many of them are pseudogenes (Hugh Robertson; personal communication). This means that the chemoreceptor family in the beetles may well be added to in future.

Alignment analysis showed that the *Tcas*Gr receptors shared a signature motif of 32 amino acids. The data agreed well with [8], who showed that 23 of the *Dmel*Gr and 33 of the *Dmel*Or share a 33 amino acids motif. Within the *T. castaneum* novel chemoreceptor genes family none of the predicted receptor genes appear to be pseudogene. Within the *D. melanogaster* chemoreceptor family only *Dmel*Or98p is a pseudogene [9]. The olfactory receptors family of *A. mellifera* included 7 pseudogenes [11]. In *A. aegypti* 21 of the identified Ors are pseudogene [15]. 46 of the genes have a conserved intron near the carboxyl terminus.

In *Drosophila* half of the predicted Gr genes had a single intron in the 7TM region [5], [7]. In this study most of the *T. castaneum* chemoreceptors can be arranged into large clusters up to 8 genes that have 0–4 introns. 64 memebrs of *T. castaneum* chemoreceptors had one intron localized in the 7TM region. Some of the Grs in *Drosophila* are encoded by alternatively spliced genes [5], and in *A. gambiae* 5 Grs are also alternative spliced genes [4]. The *Tribolium* Gr family included also alternative spliced genes. The *Drosophila* chemoreceptors are distributed on 5 chromosomes and presented in clusters of more than 3 genes [4]. The olfacory receptors in *A. gambiae* are distributed over three chromosomes, arranged mostly in pairs, triples or large clusters up to 9 genes [10]. The *T. castaneum* chemoreceptors are distributed on nine chromosomes mostly arranged in clusters of 7 genes or in large clusters up to 24 genes. This pattern of lineage-specific subfamily may reflect the physiological, biological, and ecological importance of these receptors.

Insect chemoreceptors belonged to the GPCR class A, the bovine rhodopsin like family [20], where the second extracellular loop (E2 loop) has extensive contact with many extracellular regions as well as with the ligand retinal. Length variations, especially in the E2 loop and in terminal fragments, could contribute to the functional specificity between the ligands and the G-proteins [21]. Abnormal length configuration in the E2 loop may also play a negative regulatory role in receptor activation, where it stabilizes the inactive state of the receptor [22]. The *T. castaneum* chemoreceptor proteins have an E2 loop with a mean of 36 amino acids. The shortest E2 loops (12 amino acids) were found in *Tcas*Gr7, 29, 30 and 60, whereas the longest E2 loop (87 amino acids) was found in *Tcas*Gr49. The data is in line with [23], who showed that the invertebrate Or receptors have a relatively long E2 loop with 47 amino acids on average.

Insect Ors do not belong to the GPCR family [24]. Recent studies using GPCRHMM analysis also strongly rejects the claim that the arthropod-specific Or receptors are GPCRs [25]. A detailed analysis showed that these receptor sequences, including *D. melanogaster* Gr and *A. gambiae* Gr, are very different from other GPCR proteins [25]. GPCRHMM protein analysis shows that the *Tcas*Gr19, *Tcas*Gr40, and *Amel*Gr5 are true GPCR protein. It seems that these proteins form a superfamily of environment-sensing receptors, which have little in common with odorant receptors in the mammals, and probably have a different membrane topology [25].

Phylogenetic analysis of the Gr families reveals similar pattern of largely lineage-specific gene subfamily. There are 13 orthologous pairs of Grs shared by the *T. castaneum*, *D. melanogaster*, *A. gambiae*, *A. mellifera*, and *A. aegypti* species, but only two of them are conserved (*Dmel*Gr5a ortholog and the *Dmel*Gr21a, *Dmel*Gr63a, and *Agam*Gr22-24 ortholog), suggesting that these receptors might be sufficiently conserved to recognize the same ligands in different species. An ortholog which is represented by six receptor genes in *T. castaneum* was evolutionary related to the sugar receptors candidates of *D. melanogaster* (*Dmel*Gr5a subfamily) [19], [9], *A. gambiae* [10], *A. aegypti* (unpublished data), and *Amel*Gr1 and *Amel*Gr2 of *A. mellifera* [11]. Another ortholog of *Tcas*Gr1, *Tcas*Gr39, and *Tcas*Gr3 is related to *Dmel*Gr21a, *Dmel*Gr63a, and *Agam*Gr22-24 receptor gene subfamily. None of the *Amel*Grs clustered within these orthologs. Recently in [26], it was shown that the *Dmel*Gr21a and *Dmel*Gr63a and their homologs in *A. gambiae* (*Agam*Gr22 and *Agam*Gr24) are true carbon dioxide receptors. Whether, *Tcas*Gr1, TcasGr39, and *Tcas*Gr3 are CO_2_ receptors is still unknown.

One exception to the rapid evolution can be seen in the expressed *Dmel*Or83b receptor and its orthologous in other insect species, including *Amel*Or2, *Agam*Or7, *Hvir*Or2, *Bmor*Or2, *Bmor*Or2a, and *Aaeg*Or7 [11], [10], [27], [12], [15], where they expressed in olfactory and gustatory organs. *Dmel*Or83b has an unusual function, acting as “courier” supporting the functions of other Or receptors in the dendritic membrane of olfactory receptor neurons (ORNs) [24], [28], [29]. *Dmel*Or83b and its ortholog *Agam*Or7 were also broadly expressed throughout olfactory and gustatory tissues of *Drosophila* and *Anopheles* [24], [30]. Absence of *Dmel*Or83b in *Drosophila* limited the activity in neurons of other olfactory receptors. This gravely reduced the physiological and behavioral responses of adult *Drosophila* to a wide range of odorants, altered adult metabolism, enhanced stress resistance and extended their life span [31]. In *T. castaneum* the *Tcas*Or16 is an ortholog to *Dmel*Or83b. It was expressed in gustatory and olfactory organs of the beetles and shared 75% of its' amino acid sequences identity with *Dmel*Or83b. Whether the *Tcas*Or16 receptor is also necessary for the mediation of other receptors functions, and is essential for the development of the beetles, has still to be elucidated.

The organs of smell (olfactory sensilla) in Coleoptera species lie not only on the antennae but also on the wings and the legs [32], while taste sensilla are concentrated on the maxillae and labial palpi and other mouthparts and are often found on tibiae or tarsi of the front legs. In insect the ORNs had been shown to detect odor quality, quantity, and temporal changes in odor concentration [33], [34], [35]. The antenna in *Drosophila* has about 1200 ORNs and 120 on the maxillary palp [36], [37]. On the antennae of the mosquito *A. gambiae* about 1500-1600 ORNs are present [38]. In Tenebrionidae species *Tenebrio molitor* the antenna bear 2861 ORNs distributed on antennal segments 11, 10, 9, 8, 7, 6, and 5 with 1338, 705, 455, 208, 118, 34, and 3 sensilla, respectively, [39]. 564 of these sensilla probably have olfactory and or gustatory functions and belonged to thin-walled peg sensilla. Other type of sensilla such as thick-walled peg organs, grooved peg organs, and smooth-surfaced peg organs sensilla are located on the antennal segments 11, 10, 9, 8, 7, 6, 5, and 4, respectively, and have olfactory functions. The larvae of *T. castaneum* have 21 sensilla organized into 1 trichoid, 8 styloconic, 1 placoid, 9 campaniform, and 2 coeloconic sensilla on the antennae [40].

All of the *Tcas*Or genes were localized on the antennal segments 9-4. Q real time RT-PCR analyses showed also that 25 of the *Tcas*Or genes were expressed in the antennae. The *Tcas*Or16 (homolog to *Dmel*Or83b) was localized in the antennae, maxillae, labium, and prolegs of the beetles. Low expression was recorded within the brain-SOG tissues. I suggest that the *Tcas*Or16 was mainly expressed in the flat-tipped peg sensilla, chaetica sensillum or in thin-walled peg sensilla. Such sensilla were previously suggested to have gustatory functions in *T. molitor* (Tenebrionidae) [39]. The *Drosophila* olfactory sensilla were mainly located on the antennae and maxillary palp, in each of which one or few olfactory receptor genes are expressed [6], [41], [4].

Q-RT-PCR analysis revealed that the 62 *Tcas*Gr genes were expressed as a single transcript in *T. castaneum* labium and maxillae tissues (gustatory organs of the mouth parts). Low expression levels of the genes were found in the head tissues (brain-SOG). *In situ* whole mount RT-PCR analyses also showed that the Gr receptors were localized in the maxillae and labium of the beetles. Within the Teneprionidae *Cryphaeus cornutus* taste sensilla are located on the maxillae and labial palp. There are 12 sensilla on the maxillary palp and 28 on the labial palp [42]. In coleopteran species the number of sensilla on the palps ranges from 4 to 12. In *Drosophila* the taste sensilla are located on the labial palp, proboscis, labral and cibarial sense organ on the pharynx, where their neurons projects axons to the SOG of the brain, processing gustatory information [6], [41], [4]. A few chemosensory neurons were located on the legs of *Drosophila*, legs bristles, tibia and tarsi, on the anterior wing margin, and on the abdominal vaginal plate. 57 *Tcas*Gr genes were expressed and localized in the proleg tissues RNA (trochanter, femur and Tarsi). In beetle legs there are a group of ORNs located at the end of each trochanter, sometimes on the femur, commonly on the tibia, and sometimes on the tibial spine and on the tarsi [32]. The number of the ORNs on the six legs varied from 49- 341. For example, *Tenebrio molitor* has 66 chemoreceptor sensilla on the legs, 145 on the elytra and 462 on the wings, while *Uloma impressa* has 80 chemoreceptor sensilla on the legs, 119 on the elytra and 361 olfactory sensilla on the wings [32]. Expression studies and tissue specific localization of the predicted *Tcas*Gr and *Tcas*Or genes in the wings and other two pairs of legs have not yet been carried out.

An approximate 1:1 ratio of Or genes to glomeruli was found in the neurobiological organization of the olfactory system in several different insects such as *D. melanogaster* [43], [44], *A. mellifera* [11], *B. mori* [12]. *A. gambiae* has 61- 60 glomeruli [45] while the number of Or receptors is 79 [10]. Expression analyses in the larval and adult olfactory organs also reveal that the ratio of odorant receptors to antennal glomeruli was not one to one in *A. aegypti* [15]. In this study 26 olfactory receptors were predicted. The total number of olfactory sensilla within the Tenebrionidae species is in general more than 26. Taking this into account the family of olfactory receptors in *T. castaneum* might be an underestimate. Future studies to prove this theory may be interesting.

The results demonstrated here present a putative chemoreceptor family in *T. castaneum*, an insect species with biological and evolutionary history different from those for *D. melanogaster*, *A. gambiae*, *B. mori*, or *A. mellifera.* Knowledge of the genes structure, characterization of their expression, and their evolutionary relationship to other insect species are significant steps towards a deeper understanding of how chemical stimuli in *Tribolium* are perceived and processed. These results also open the way to the characterization of their homologs in other coleopteran species.

## Materials and Methods

### Bioinformatics analyses

Known insect chemoreceptors sequences which had been submitted to GEN-BANK (National Center for Biotechnology Information) were used to search for similar genes in *Tribolium castaneum* genome sequences. The protein sequences were used to perform a TBLASTN [46] comparing a protein sequence against nucleotide database through two servers: http://www.ncbi.nlm.nih.gov/projects/genome/seq/BlastGen/BlastGen.cgitaxid7070 and http://www.hgsc.bcm.tmc.edu/projects/tribolium/. The predicted nucleotide sequences were translated into amino acid sequences using a variety of bioinformatic algorithms including the Baylor College of Medicine six frame translation server (http://searchlauncher.bcm.tmc.edu/seq-util/Options/sixframe.html/), the translate tool server at http://www.expasy.org/tools/dna.html/, and EMBOSS transeq server at http://www.ebi.ac.uk/emboss/transeq/. Prediction of intron/exon splice sites was carried out using the online servers http://deepc2.psi.iastate.edu/cgi-bin/sp.cgi/ and http://www.fruitfly.org/seq_tools/splice.html. Transmembrane helix domain within the predicted proteins was done using TMHMM program; http://www.cbs.dtu.dk/services/TMHMM-2.0/, Tm-pred program; http://www.ch.embnet.org/software/TMPRED_form.html, and HMMTOP version 2.0 program; http://www.enzim.hu/hmmtop/html/submit.html/. The identified proteins were analyzed to GPCR structure using the GPCRHMM server (http://gpcrhmm.cgb.ki.se/tm.html/ ) [26]. The blast servers http://www.expasy.ch/tools/blast/ and http://www.ncbi.nlm.nih.gov/BLAST/Blast.cgi/ were used for comparison with other known protein sequences from different species. The servers http://www.soe.ucsc.edu/research/compbio/gpcr-subclass/, and http://pfam.janelia.org/hmmsearch.shtml/ were used to classify the predicted proteins into subfamily. Finally all of the chemoreceptor DNA sequences were amplified using PCR techniques for further study.

### Beetles rearing

*T. castaneum* beetles were purchased from Biologische Bundesanstalt (BBA); Berlin, Germany, and were reared in glass containers provided with growing medium under constant conditions of 30°C and 70% relative humidity at L 16: D 8 hrs photoperiod.

### Genomic DNA extraction

To amplify the complete receptor gene sequences of the putative chemoreceptors, genomic DNA of *T. castaneum* was extracted and used as a PCR template in the following manner: individual *T. castaneum* adults were frozen in liquid nitrogen and ground to a fine powder. The powder was resuspended and the genomic DNA was extracted according to the protocol supplied by the manufacturer of QIAamp® DNA Mini kit (50) (Qiagen GmbH). The extracted genomic DNA was later used as a template in PCR reactions to amplify the whole receptor gene sequences. The genomic DNA (100 ng/µl) was used as a template in combination with the specific TcasGr1-62/OrF1-26-TcasGrR1-62-OrR1-26) (Table S1A). The PCR program was 95°C for 3 min, followed by 10 cycles of 94°C for 30 sec, 55°C for 45 sec decreasing by 1.0°C per cycle and 68°C for 2 min, followed by 35 cycles of 94°C for 30 sec, 40–45°C for 45 sec, 68°C for 2 min, and a final extension step of 68°C for 10 min.

### Cloning and sequencing

All of the amplified DNAs were eluted from low melting point agarose gel (Biozym Biotech, Oldendorf, Germany) using GFX™ purification kit (Pharmacia Biotech, Freiburg, Germany), and ligated into plasmids with the StrataClone™ PCR vector cloning kit supplied with StrataClone™ SoloPack competent cells (Stratagene) for amplification. Plasmid DNAs were later purified using QIAprep® Spin Miniprep kit (Qiagen GmbH). The templates were sequenced by MWG Biotech (Ebersberg, Germany).

### Phylogenetic analysis

The *D. melanogaster*, *A. gambiae*, *A. mellifera*, *H. virescens*, *B. mori*, and *A. aegypti* olfactory and gustatory receptor sequences were downloaded from the genomic DNA archive database at the national center for biotechnology information (NCBI) (http://www.ncbi.nih.gov/blast/agtrace.html/). Alignment analysis of *T. castaneum* Gr and Or receptor deduced amino acid sequences were performed by means of AlignX server; http://www.ebi.ac.uk/clustalw/ [47]. The corrected distance of tree was rooted by declaring the *Tcas*Or16 and its homologs (*Dmel*Or83b, *Amel*Or2, *Agam*Or7, and *Hvir*Or2) as outgroup, based on their position at the base of the insect Or family in the phylogenetic analysis of [11]. The neighbour joining tree was produced using the PHYLIP package [48] and based on the consensus of 1000 bootstrap replicates. Tree drawing was performed as corrected-distance cladogram with the help of TreeView 1.6.6. program.

### Quantitative real time RT-PCR analysis

To investigate the expression of *T. castaneum* chemoreceptors in different tissues, total RNAs were extracted from two different biological pools of labium (gustatory tissue), maxillae (gustatory tissue), antennae (olfactory tissue), heads (from which olfactory and gustatory tissues had been removed but still included brain suboesophageal ganglion (SOG)), and prolegs of *T. castaneum* adults (N  = 100 per biological pool). The total RNA was extracted using Invertebrate RNA Kit (Peqlab Biotechnologie GmbH) in combination with a DNase treatment (RNase-free DNase set; Qiagen) to eliminate potential genomic DNA contamination. After extraction, the RNA samples were stored at −80°C until use.

The expression of the *T. castaneum* chemoreceptors was performed using quantitative real time RT-PCR analysis. The Q-real time RT-PCR reactions were preformed in 20 µl reaction volume containing 10 µl of 2× FullVelocity™ SYBR® Green Q-RT-PCR master mix, 0.3 µl diluted reference dye, 0.05 µl StrataScript® RT/RNase block enzyme mixture (Stratagene), 1 µl pro primer (20 pmoL) (Table S1B), 2 µl RNA (2 ng/µl), and 5.65 µl DEPC H_2_O. To generate a relative standard curve, the endogenous protein *T. castaneum* actin (EMBL Nucleotide Sequence Data Base, accession number; XM_961644 was used. The actin primer nucleotide sequences were 5′-ATGTCGGCGACGCTAC-3′ as forward primer and 5′-GCTATACTGATACGGAC-3′ as reverse primer. With these primers a fragment of 224 bp was yielded and later used as a relative standard. Relative standard curves for the *Tcas*Gr and *Tcas*Or receptors were generated with a series of five dilutions of the RNA from adult *T. castaneum* labium, maxillae, antennae, heads and prolegs, respectively. A dilution series of a standard sample (actin) was included within each RT-PCR run. Reactions were run in triplicate on MX3005P Real Time PCR System (Stratagene) using the following thermal cycling profiles of 50°C (30 min) and 95°C (5 min), followed by 40 steps of 95°C for 30 sec, 45–50°C for 1 min and 72°C for 30 sec. Afterwards, the PCR products were heated to 95°C for 30 sec, cooled to 45°C for 1 min and heated to 95°C for 30 sec to measure the dissociation curves. All assays were performed twice for each RNA biological pool to minimize variations due to sample handling.

The specificity of the different PCR amplification reactions was double checked using analysis of the dissociation curves for the *T. castaneum* chemoreceptor gene transcripts and their endogenous control, which showed a single melting peak. The different PCR products were later analyzed via gel electrophoresis, which proved the presence of a single band of the expected size for each chemoreceptor gene transcript. In all negative controls, no amplification of the fluorescent signal was detected, proving that there was no contamination with genomic DNA in the RNA samples

### Whole-mount *in situ* RT-PCR analysis

The head tissues of *T. castaneum* adults, including the labium, maxillae, and antennae, the prolegs of adult beetles, and the heads of last instar larvae were dissected and immediately placed in fixative buffer (4% paraformaldehyde in DEPC-PBS buffer (130 mM NaCl, 7 mM Na_2_HPO_4_, 3 mM NaH_2_PO_4_ )). After decolourization by incubation in H_2_O_2_ for 1 h. the tissues were dehydrated in a series of 30, 50, 70, 95, and 96% (v/v) ethanol/H_2_O followed by protein digestion using proteinase K solution (2 μg/ml) for 15 min. For other details see [49].

## Supporting Information

Table S1 Nucleotide sequences (5-3′) of the primers that were used in this study
(0.36 MB PDF)

Table S2 Insect chemoreceptors that are not belong to the GPCR superfamily.
(0.09 MB DOC)

Figure S1 *T. castaneum* putative chemoreceptors gene family. (A) The deduced amino acid sequences from the cDNA of the *T. castaeum* gustatory (Gr) and olfactory receptors (Or). (B) Table showing the *T. castaneum* chemoreceptor names (accession numbers) and the predicted TM domain positions for each receptor using the GPCRHMM server; http://gpcrhmm.cgb.ki.se/. (C) Alignment analysis of the insect gustatory receptor superfamily including *T. castaneum* Gr, *D. melanogaster* Gr, *A. gambiae* Gr, *A. mellifera* Gr, and *A. aegypti* Gr using the http://www.ebi.ac.uk/clustalw/server. The gene names are given at the left and the amino acid positions are given at the right. (D) Alignment analysis of the insect olfactory receptor superfamily including the putative *T. castaneum* Or, *D. melanogaster* Or, *A. gambiae* Or, *A. mellifera* Or, *H. virescens* Or, *B. mori* Or, and *A. aegypti* Or7. For other details see (C).
(0.71 MB PDF)

Figure S2 The signature motif of the Gr receptors in insect species. Sequence alignments of the Gr gene families of *T. castaneum*, *D. melanogaster*, *A. gambiae*, *A. mellifera*, and *A. aegypti* revealed a common amino acid motif in the Gr sequences. The Gr sequences are marked to the left with their proposed gene name. The average similarity in the sequence motif was more than 50%. Alignment of the amino acid sequence was analyzed using CLC Free Workbench 3.2.2. software. The consensus alignment and the colouring of the conserved residues was asigned using ClustalX.
(0.88 MB PDF)

Figure S3 The molecular evolution of 24 chemoreceptor genes localized on chromosome seven of *T. castaneum*. The proposed gene names for the chemoreceptor are given to the right. The main lineages within the receptors are supported by bootstrap values >50% and are indicated with *. Only TcasGr19 does not belong to any of the orthologs within the tree. For other details see Figure S2.
(0.01 MB PDF)

Figure S4 Neighbor joining tree of the insect gustatory receptor superfamily. *D. melanogaster* (DmelGr), *A. gambiae* (AgamGr), *A. mellifera* (AmelGr), *A. aegypti* (AaegGr) (unpublished data), and *T. castaneum* Gr receptor gene sequences were used to draw the orthologous relationships between the gustatory receptors of the different insect species. Insect Gr receptors are indicated at the right. Receptors that represent lineages between different insect species are written in bold and italic. The supported bootstrap values (>50%) are denoted with #. The insect sugar receptor subfamily is shown in blue. The insect CO2 receptor subfamily is indicated in green. A weak bootstrap value (<50%) is indicated with a black square, and the appropriate orthologs are written in red. * represents lineages of only *T. castaneum* Gr receptor orthologs supported with a bootstrap value of >50%. For other details see Figure 1.
(0.02 MB PDF)

Figure S5 Neighbor joining tree of the insect olfactory receptor superfamily. Insect olfactory receptor genes (Or) are indicated to the right. The corrected distance tree was rooted by declaring the TcasOr16 and its homologs (DmelOr83b, AmelOr2, AgamOr7, BmorOr2, BmorOr2a, and HvirOr2) as outgroup. Receptors that represent lineages between different insect species are written in blue, and the supported bootstrap values (>50%) are denoted with #. * represents lineages of only T. castaneum Or receptor orthologs supported with a bootstrap value of >50%. For other details see Figure 1.
(0.03 MB PDF)

Figure S6 Tissue specific localization of the T. castaneum Gr receptors. The T. castaneum Gr11-62 were localized in the labium and maxillae of the adult beetles, and in the femur, tibia and tarsus of the larval prolegs. For other details see Figure 5.
(2.03 MB PDF)
