# Supplementary material for: A Family of Chemoreceptors in Tribolium castaneum (Tenebrionidae: Coleoptera)
Source: PLoS One. 2007 Dec 19;2(12):e1319. doi: 10.1371/journal.pone.0001319 (PMC2121604; doi:10.1371/journal.pone.0001319)
Supplement: Table S1 — Nucleotide sequences (5-3′) of the primers that were used in this study (0.36 MB PDF) [file pone.0001319.s001.pdf]

Table S1. Nucleotide sequences (5-3’) of the primers that were used in this study.

(A)

Target gene	Forward primer name	Forward primer sequence (5'-3')	Reverse primer name	Reverse primer sequence (5'-3')	Accession number	
*Tcas*GR1	F1	ATGCAACACGAAGTCGTC	R1	TTAACCAAAACTTACCGAAA	AM292322	*T. castaneum* Grs
*Tcas*GR2	F2	ATGGAAAAGTCGACGAAA	R2	TTAAGCGCAGGTTAAACTG	AM292323	
*Tcas*GR3	F3	ATGTACCACCAAGACCAAGC	R3	TTAGTTGGGTGTTTGCGTAGTG	AM292359	
*Tcas*GR4	F4	ATGGTTCGGCCAGAGCTTGAC	R4	TCATATCGAAAAGTTTTGTG	AM292325	
*Tcas*GR5	F5	ATGACAATACAGTTGTTG	R5	TTAACTGGTCAAGTTTTCAA	AM292326	
*Tcas*GR6	F6	ATGGAAATAAGCGACTTGG	R6	TTATCCCCGCATATTGAGTA	AM292327	
*Tcas*GR7	F7	ATGAGAGTGCAGCCTTCAAAC	R7	TCACAGGTAAACGGGGCACG	AM292328	
*Tcas*GR104	F8	ATGTGTTATTTCAATTACTC	R8	TTATTTAGCTGAGCTTTGAT	AM292329	
*Tcas*GR9	F9	ATGGAAAAGTCGACGAAAAT	R9	TTAGCAAATAGAGTTTCTTG	AM292330	
*Tcas*GR10	F10	ATGAGAAATGACCATGGGTCC	R10	TTAGGACATGGTTGTATTCCC	AM292331	
*Tcas*GR11	F11	ATGATCAAATTTTCCAAAG	R11	CTACAAAATAATAATTAC	AM292332	
*Tcas*GR12	F12	ATGTCCAACTTGAAGAAGCTC	R12	CTAAACTTGCACTATTTTATCG	AM292333	
*Tcas*GR13	F13	ATGCGGTTTTTGGGGGAAAGC	R13	CTAAACTTGCACTATTTTATC	AM292334	
*Tcas*GR14	F14	ATGCCCCTCCTTCCCAAGAC	R14	TTAAAAATTGAACTGGATCA	AM292335	
*Tcas*GR15	F15	ATGGACATTAAATCAATCAA	R15	TTATGTCAAGTAGAAAGCGTG	AM292336	
*Tcas*GR16	F16	ATGCCTCAAGTCAAACTCCAC	R16	CTACTCCACCAAAATTGTAC	AM292337	
*Tcas*GR17	F17	ATGAACACGTTTAAAATTTTCC	R17	TTAGGCGGTGGTACCATTGCC	AM292338	
*Tcas*GR150	F18	ATGAACACGTTTAAAATTTTCC	R18	TTAGGCGGTGGTACCATTGCC	AM292339	
*Tcas*GR19	F19	ATGACAAGTAAAGCACGCAC	R19	TTACCAAACAACGAAGAGCC	AM292340	
*Tcas*GR20	F20	ATGACACCTCACGCTTTAATT	R20	TTAGTAAACATTCCCATGAATAC	AM292341	
*Tcas*GR21	F21	ATGTTGCCTAAACAATTTT	R21	TTAATAACTTTCTGTCGCG	AM292342	
*Tcas*GR22	F22	ATGTTCCGCGACTTGGGCAG	R22	TCAATAAAGAGTTGAGTTTGTG	AM292343	

*Tcas*GR79	F23	ATGGAATACAGATCGCAAAC	R23	TTAATTAATTTGAAATTGTAAC	AM292344
*Tcas*GR123	F24	ATGTCGGATTTCCAAAAACTG	R24	TTATTGTCCGGCAAGTGATGTG	AM292345
*Tcas*GR25	F25	ATGTCACAAGTTATGCTAATC	R25	CTAATTTTTTGGAATTTTTTGA	AM292346
*Tcas*GR26	F26	ATGGAAATAAGCGACTTGGCC	R26	TTATCCCCGCATATTGAG	AM292347
*Tcas*GR27	F27	ATGTCGCACTCGAACCCC	R27	TTAATTGTTCCTAATATCGTGC	AM292348
*Tcas*GR28	F28	ATGCGTTTAATAAGAAAGATG	R28	TTAACGTCTTACTTCAAC	AM292349
*Tcas*GR29	F29	ATGAGAGTGCAGCCTTCA	R29	TCACAGGTAAACGGGGCACG	AM292350
*Tcas*GR30	F30	ATGAGAGTGCAGCCTTCAAAC	R30	TCACAGGTAAACGGGGCACG	AM292351
*Tcas*GR31	F31	ATGGGCTCGGTTGCAATTAATC	R31	TTAAAACTGATCAATTAAGAATG	AM292352
*Tcas*GR32	F32	ATGAGCTTCAAGTTGTTGAG	R32	TTATGGTTGCGAATAGTTCG	AM292353
*Tcas*GR33	F33	ATGGAAATAAGCGACTTGGCC	R33	TTATCCCCGCATATTGAGTAAAC	AM292368
*Tcas*GR34	F34	ATGAGTTCCCAACTATTG	R34	TTATCGTTCTTTATCGAACTGG	AM292355
*Tcas*GR35	F35	ATGCGTTTGAAATGGTATTAC	R35	TCAATTTTCTTTAAATTGG	AM292356
*Tcas*GR105	F36	ATGTATTTTTCACGACGAG	R36	TCAAAAACCGAATTCAATAATTAC	AM292357
*Tcas*GR37	F37	ATGCTGAAGTGGGTCCTTA	R37	TTATGGTTGCGAATAGTTCG	AM292358
*Tcas*GR38	F38	ATGAATAATAAACTTAACATG	R38	TTAAATTCGCTCAACTTGG	AM292324
*Tcas*GR39	F39	ATGAGAAATGACCATGGGTC	R39	ACGTTAGCAATTGCTCTTCTTG	AM292360
*Tcas*GR40	F40	ATGGTGTTTGCCGAGCTCTG	R40	TTATACCACAGCGAAAATAGC	AM292361
*Tcas*GR41	F41	ATGATCGAAGTTGGTAAAAC	R41	CTAGTTGTATTTTCCCTGCTTTTG	AM292362
*Tcas*GR71	F42	ATGACAGACCACAACCTAC	R42	TCAGTGTTTATTATTAAACTG	AM292363
*Tcas*GR43	F43	ATGGATTTCCAGTTGTTAC	R43	TTAGGAATTACGAAACTGCAC	AM292364
*Tcas*GR44	F44	ATGACTAGAGCTATGGTTCG	R44	TCATATCGAAAAGTTTTGTGGC	AM292365
*Tcas*GR45	F45	ATGTGTTATTTCAATTACTC	R45	TTATTTAGCTGAGCTTTGAT	AM292366
*Tcas*GR46	F46	ATGGAAGAAAAACTGGTG	R46	TTACTGTTTAAGTGATGCCG	AM292367
*Tcas*GR47	F47	ATGGATTTAGAAGTGTTGG	R47	TTAATCCTGAAATTGCGCAACC	AM292354
*Tcas*GR48	F48	ATGCACACACTTCAACATTTTAC	R48	TTAAATACTATACTCGTATG	AM292369
*Tcas*GR49	F49	ATGTGCCAAATAAAAAACAAG	R49	TCATCTGAAATTTGCCTC	AM292370
*Tcas*GR50	F50	ATGTCGCTTTATTCGTCCATC	R50	TCACTGTTCTGTTTCTTTG	AM292371
*Tcas*GR51	F51	ATGAACATGATTCTCAAACTATC	R51	TTATTCTTTTTGGTTTTGATT	AM292372

*Tcas*GR54	F52	ATGCAGGATTCGTTCCAATATATC	R52	TTACAGCGACATATTAAATTG	AM292375	
*Tcas*GR53	F53	ATGCTGAAGTGGGTCCTTAAAG	R53	GACGATCAAAAACGTGAC	AM292383	
*Tcas*GR52	F52	ATGAGCTTCAAGTTGTTGAG	R52	TTACAAGTTGATGTTTGTG	AM292373	
*Tcas*GR125	F55	ATGATCAAATTTTCCAAAG	R55	TTAACAAGTGCTATTGTTTG	AM292376	
*Tcas*GR56	F56	ATGTTGCCTAAACAATTTTTAAA	R56	TTATTTGTAACACTTTTTACTC	AM292377	
*Tcas*GR57	F57	ATGAAAGAAGTGCCCGGTTGGC	R57	TTATTCGATAAACTGTATCG	AM292378	
*Tcas*GR98	F58	ATGTCATACAGACTGTCTCGC	R58	TTATTTATTTAAACCTTG	AM292379	
*Tcas*GR59	F59	ATGTGGAGTGAAAATATTACAC	R59	CTAATTTTTTGGAATTTTTTG	AM292380	
*Tcas*GR60	F60	ATGATACTAGTACTACTAGTG	R60	TCAATAAAGCCAAAACAATTTG	AM292381	
*Tcas*GR61	F61	ATGACCAAAAGCACAGTCTATG	R61	TTATGGCCCACAAGTGGGTTTG	AM292382	
*Tcas*GR62	F62	ATGATGGATAGAATAAATCAC	R62	TTATTGTCCGGCAAGTGATG	AM292374	
*Tcas*Or1	F1	ATGAATAATTCCAGGCCTGC	R1	TCATTCTCTCACCTCATG	AM689931	*T. castaneum* Ors
*Tcas*Or2	F2	ATGTTTAAAACCGTAACGAGG	R2	TCACTAAATTCCTGAAATTAC	AM689904	
*Tcas*Or3	F3	ATGTCCAAAAGTGAAAAAATCC	R3	TCAGTGATTTATGCCGTG	AM689905	
*Tcas*Or4	F4	ATGGACGAAGAGTTTTTAATCG	R4	TCATTTGACAATTTCTTTGTG	AM689906	
*Tcas*Or5	F5	ATGGTGTGGTCACCCAAAGATG	R5	AAGTATCACTAGTTGCAAAC	AM689907	
*Tcas*Or6	F6	ATGAGTCCGCACGATTCGGG	R6	TTACTCTAACACAAATCTGTG	AM689908	
*Tcas*Or7	F7	ATGTACCGACTTGTCGCTTCC	R7	TCACTAACTCACAGTGTAAGC	AM689909	
*Tcas*Or63	F8	ATGGGTTTTATGATACAGG	R8	TTAGGAATCTTTATACAGGCG	AM689910	
*Tcas*Or9	F9	ATGGAAAAATCGTCAAAATTATC	R9	TCAGTTAACTCCTTCAAGAGC	AM689911	
*Tcas*Or10	F10	ATGGCCACTTTCCTACTTTAC	R10	TCATACCAGCAAAAAAGTTCG	AM689912	
*Tcas*Or11	F11	ATGGGCAAAGTCAAGTTTACG	R11	TTATTTCTGATTCATAGCGTTG	AM689913	
*Tcas*Or12	F12	ATGGCACATAATCTGGACG	R12	TTATTCCAAATTAAGCAAATAG	AM689914	
*Tcas*Or13	F13	ATGAGTGCAATGTCGACGG	R13	TTAATTAAACTGAAACAAAATC	AM689915	
*Tcas*Or56	F14	ATGACCGCAACGAAAAGCC	R14	TTAAATATTCATTTGCCTC	AM689916	
*Tcas*Or15	F15	ATGAGCAAAAACCTCAAAG	R15	AGTATTCATTTGTTTCAAAAAC	AM689917	
*Tcas*Or16	F16	ATGATGAAATTCAAGGTC	R16	TCATTTGAGTTGCACCAACACC	AM689918	
*Tcas*Or17	F17	ATGTCCAACTTTAGCTGG	R17	TTACCTCAAAATTTCTCTATGG	AM689919	

*Tcas*Or18	F18	ATGGCAAAACTGGAGTACC	R18	TTAATCTTTCCCCAAATCAGTC	AM689920	
*Tcas*Or19	F19	ATGGTTCTACTTCCGACTG	R19	TCATACGGTAACAAACAGAGC	AM689921	
*Tcas*Or20	F20	ATGATGGACGAGACTTACC	R20	ACGGTTTTAATTTCAAAAGTAG	AM689922	
*Tcas*Or21	F21	ATGGAGAAATATGATTGG	R21	TTACTTCAAAATTTCTTGGTGG	AM689923	
*Tcas*Or22	F22	ATGGCAAAGGACTCGTCCC	R22	CACTAAGTGGACTGGTGCAAA	AM689924	
*Tcas*Or23	F23	ATGGCAAAAGACACGTCCC	R23	ACTATGTGGATTTGTGCAAAAG	AM689925	
*Tcas*Or24	F24	ATGACTGAAGAGAAACAG	R24	ATTGCAGGATTGTGAGAATGG	AM689926	
*Tcas*Or25	F25	ATGACCGAAGAGAAAGAG	R25	TTACTACCCCATCATAAAGCC	AM689927	
*Tcas*Or26	F26	ATGATGGAATCAACCGTC	R26	ACTAAGAACTAGCGTTAAG	AM689928	

(B)

*Tcas*GR1	GrF1	TGGTAACACGTTTCAATGAG	GrR1	CACAAATAATGCCAAGGAAAG	AM292322	*T. castaneum* Grs
*Tcas*GR2	GrF2	TAATCAAATCTTTTAACGAG	GrR2	GAGCTTTCCACAACGAG	AM292323	
*Tcas*GR3	GrF3	GTCTCAAGTCGCCGATTATC	GrR3	GCTGTGACAGTAACCCGTAG	AM292359	
*Tcas*GR4	GrF4	CAATCTTTGGAGTGTCTTTC	GrR4	GTGTAAACAGTGGCCCACAGTTGG	AM292325	
*Tcas*GR5	GrF5	GTGTTGATCTCTTCAATAGTC	GrR5	CCATAAAATAAAGATCACACTGTAGG	AM292326	
*Tcas*GR6	GrF6	GAGTGCTGTGGTTATCAC	GrR6	AACTGAAACGAATACCGTGATC	AM292327	
*Tcas*GR7	GrF7	CGTACATAGTCCTCATGTC	GrR7	CACAAGCACACCGTACGCACG	AM292328	
*Tcas*GR104	GrF8	TTTAACGACGTCTTCGGG	GrR8	ACTAGGCACAAATTCGCGGC	AM292329	
*Tcas*GR9	GrF9	CATCGTTTCTCAACAACTTC	GrR9	CGTGACCACAGCACTCG	AM292330	
*Tcas*GR10	GrF10	ATCCTGGGCGCATATCGCG	GrR10	TGAAGGAGATCCCCTGGTCC	AM292331	
*Tcas*GR11	GrF11	GTACATCTTGTGCCGTC	GrR11	CCAAATCCATATGGAAG	AM292332	
*Tcas*GR12	GrF12	AATGATATTTTCGGCTGG	GrR12	CTGATCGTACTCTCCCGCATTGC	AM292333	
*Tcas*GR13	GrF13	AATGATATTTTCGGCTGG	GrR13	CTGATCGTACTCTCCCGCATTGC	AM292334	
*Tcas*GR14	GrF14	CACCACAATCACGGCCATGG	GrR14	CACGAGCAGCCAATAGAAGCAG	AM292335	
*Tcas*GR15	GrF15	GTTTTCTTCACCGTTCCCAG	GrR15	ACGCCACGTTCACAATTTGATC	AM292336	
*Tcas*GR16	GrF16	GTGACTCCTATATTGTCATG	GrR16	CGCTTCTTGACACAAGTCGGTCAC	AM292337	
*Tcas*GR17	GrF17	TGGAGACGCATATGCGGTAC	GrR17	GTGACTTTTGCCTCACGACTC	AM292338	

*Tcas*GR150	GrF18	TGGAGACGCATATGCGGTAC	GrR18	GTGACTTTTGCCTCACGACTC	AM292339
*Tcas*GR19	GrF19	CATTTATTATTGTTAGTGTGC	GrR19	GGCAAAAATAATAAATGCAAAG	AM292340
*Tcas*GR20	GrF20	GAACGCTTGAACGAGTTTATG	GrR20	GATACCCAGCGTTACACACCTC	AM292341
*Tcas*GR21	GrF21	GTGGACCAAGTTGCAGCCC	GrR21	TAATTGCAACTAAAAACAGGGCTG	AM292342
*Tcas*GR22	GrF22	TGATTGGTGATTTGCACACT	GrR22	CGTTGCAAAGATCACTCGC	AM292343
*Tcas*GR79	GrF23	GAGAATTTGACCAAATTGTGG	GrR23	ACCTACAAGTCCAAGAAATGTAT	AM292344
*Tcas*GR123	GrF24	AATGTCCAAGCGGGCG	GrR24	TGTAAGTGGTGACACC	AM292345
*Tcas*GR25	GrF25	TGGCCGAAGCCTTTAGCAGC	GrR25	GAGGTAAGCGCCAGATTCTCC	AM292346
*Tcas*GR26	GrF26	GAGTGCTGTGGTTATCAC	GrR26	AACTGAAACGAATACCGTGATC	AM292347
*Tcas*GR27	GrF27	GTACCAGGGAATTCTAGTCG	GrR27	AGAGGCGTCGTACCAGTGC	AM292348
*Tcas*GR28	GrF28	GTTTTTCCAGTGGAACACC	GrR28	GAGAATAATCAATAAGCGTG	AM292349
*Tcas*GR29	GrF29	CGTACATAGTCCTCATGTC	GrR29	GATCAAGAAACCAAACGACC	AM292350
*Tcas*GR30	GrF30	CGTACATAGTCCTCATGTC	GrR30	GATCAAGAAACCAAACGACC	AM292351
*Tcas*GR31	GrF31	CGACAACAACATGTCCTAC	GrR31	CAAACCGAAATTAATCGC	AM292352
*Tcas*GR32	GrF32	CGATATTTTCGGGTGGCCC	GrR32	CCAGGAAATTACAGTAACGTTGG	AM292353
*Tcas*GR33	GrF33	GAGTGCTGTGGTTATCAC	GrR33	AACTGAAACGAATACCGTGATC	AM292368
*Tcas*GR34	GrF34	CGCTAGTTTCGCTAATTTTCC	GrR34	GTGGAAAAAACCGCCGGCTGTAAAC	AM292355
*Tcas*GR35	GrF35	TGATATTTTCGGCTGG	GrR35	CCACCCAAAGAATAAAAAGCCACAC	AM292356
*Tcas*GR105	GrF36	ACGACATCTTTGGCTGGA	GrR36	GTTGTAATTGTGGCCAG	AM292357
*Tcas*GR37	GrF37	ACGACATCTTTGGCTGGAC	GrR37	GTCCCAAATGTGTTGAAAAGAGC	AM292358
*Tcas*GR38	GrF38	GTGTGAAACTCTTCAATGAGG	GrR38	TAAAGGGAACACTGGCCAGGGCC	AM292324
*Tcas*GR39	GrF39	ATCCTGGGCGCATATCGCG	GrR39	TGAAGGAGATCCCCTGGTCC	AM292360
*Tcas*GR40	GrF40	CGGGGTACAATCTCACCACC	GrR40	CCAATTATCAAGCGCCATTTCC	AM292361
*Tcas*GR41	GrF41	CGTTGGTGTTATTTACACAAC	GrR41	CTACCAGTTTAGTTACCATG	AM292362
*Tcas*GR71	GrF42	TGAGTTTTTTGGGTGGCCC	GrR42	CATAAATGCAACGCCAACG	AM292363
*Tcas*GR43	GrF43	CGCAACTTTGGATTTCCTCG	GrR43	CCACACAGTCACACAAGATAATC	AM292364
*Tcas*GR44	GrF44	CAATCTTTGGAGTGTCTTTC	GrR44	GTGTAAACAGTGGCCCACAGTTGG	AM292365
*Tcas*GR45	GrF45	TTTAACGACGTCTTCGGG	GrR45	ACTAGGCACAAATTCGCGGC	AM292366
*Tcas*GR46	GrF46	TAATCAAATCTTTTAACGAG	GrR46	AGGTGGTAACACCTCC	AM292367

*Tcas*GR47	GrF47	GTGTGGACGGGTGATGG	GrR47	CAGCAGCTGTTATCTTAACTT	AM292354	
*Tcas*GR48	GrF48	GATTATGCTGGCTTTGAGC	GrR48	CAGAATTGAGTACCAAATAGCC	AM292369	
*Tcas*GR49	GrF49	CACCACAATCACGGCCATGG	GrR49	CACGAGCAGCCAATAGAAGCAG	AM292370	
*Tcas*GR50	GrF50	CGTCTTGTCCACATTTTCC	GrR50	GTCGCAAAGATACGCAAGTGCC	AM292371	
*Tcas*GR51	GrF51	CGAGAATTTGTCCGAAATGG	GrR51	CAATGGCATCAAAACTCTTCAC	AM292372	
*Tcas*GR52	GrF52	CAAAATTTTCGGCTGGTCC	GrR52	AAACTGAAAGGTGTATTATAGCG	AM292375	
*Tcas*GR53	GrF53	CGATATTTTCGGGTGGCCC	GrR53	CCAGGAAATTACAGTAACGTTGG	AM292383	
*Tcas*GR54	GrF54	AATGATATTTTCGGGTGGC	GrR62	CCAGAAAATCACCACAATAATGTAC	AM292373	
*Tcas*GR125	GrF55	CGTATCCATTGGTGTTTCGC	GrR55	TGCTGGCACAAGCATACACC	AM292376	
*Tcas*GR56	GrF56	GTGGACCAAGTTGCAGCCC	GrR56	TAATTGCAACTAAAAACAGGGCTG	AM292377	
*Tcas*GR57	GrF57	ATGGCTTGGCAATGCTTGAG	GrR57	GACCAAATTTGTCCCAAACGAG	AM292378	
*Tcas*GR98	GrF58	TGACATTTTTGGTTGGCCG	GrR58	CGTCTGGACAAGACTCG	AM292379	
*Tcas*GR59	GrF59	TGGCCGAAGCCTTTAGCAGC	GrR59	GAGGTAAGCGCCAGATTCTCC	AM292380	
*Tcas*GR60	GrF60	GCGCTTGATGCACCGCC	GrR60	GTACTAGTACTACAACCGC	AM292381	
*Tcas*GR61	GrF61	TTCTGTAATGTTTTGATGGAG	GrR61	CAAACCTGCGCCTAAGTATAA	AM292382	
*Tcas*GR62	GrF62	GGTGAAACTGTTCAATGAG	GrR53	CGAACGTAACATTAGACAAAATA	AM292374	
*Tcas*Or1	OrF1	CGAAAGTAAGTCTCTCGGTAC	OrR1	CCAAATTGTTAATCCATACTCAG	AM689931	*T. castaneum* Ors
*Tcas*Or2	OrF2	TAATCAAATCTTTTAACGAG	OrR2	TACCTCTTCAATCGTCG	AM689904	
*Tcas*Or3	OrF3	CTCTTGAAAGAGGAGACTTTAC	OrR3	GGAACCACCCAACGCACATGCCG	AM689905	
*Tcas*Or4	OrF4	CTCGTGCAAGTTCATCGC	OrR4	CGTGTTGTAGGGCAACCAGCC	AM689906	
*Tcas*Or5	OrF5	TGGGTTTGTCCAGTAATGC	OrR5	AAAGCCATCGGAATCCAC	AM689907	
*Tcas*Or6	OrF6	TCTGTGCTCCTTGTTTGTTC	OrR6	TCACAGCGAAATAGACAGCG	AM689908	
*Tcas*Or7	OrF7	AATCTTGAGGAAAGTGTTAG	OrR7	AAAGCCGAAGCTGTCCCG	AM689909	
*Tcas*Or63	OrF8	CCTCCAGTTGTTCGGGAGCG	OrR8	CGTAGTAGCAGTAGGTGGCAACC	AM689910	
*Tcas*Or9	OrF9	GCCGACATTGTTGGGACGC	OrR9	GTACGAGGAAGCGGACATTG	AM689911	
*Tcas*Or10	OrF10	CGGCTAAAATCTCAATTGGG	OrR10	GTCAATTTGACCGGACTTTGGGCC	AM689912	
*Tcas*Or11	OrF11	GGTTCTTTGTTAATCACCTG	OrR11	TCGCAGCTCTTTGGCCCCG	AM689913	
*Tcas*Or12	OrF12	ACTAATCTTTTCACAACCTG	OrR12	ACAAATAAATCTCCATGAGGAC	AM689914	

*Tcas*Or13	OrF13	GAGCAAGTGCGCGATTTAATC	OrR13	CCTGTGGTAAATATAATGGCG	AM689915
*Tcas*Or56	OrF14	GCAGATGCCGTCATTTGCGG	OrR14	CTCGATTCGTTCAATCTCTCG	AM689916
*Tcas*Or15	OrF15	GGGAATCCCGATAGGGG	OrR15	CGAGCCTGCTCCTCTCAC	AM689917
*Tcas*Or16	OrF16	ACGAGGATCTTAAGGGGGTG	OrR16	CCACCCAGTATTTGATCGCCG	AM689918
*Tcas*Or17	OrF17	GGACAAATGGACCAAAGAC	OrR17	CTTGGTGGTGTTTTATGCACG	AM689919
*Tcas*Or18	OrF18	CATCATAATTTCAACCGAC	OrR18	TTCCCCAAATCAGTCAC	AM689920
*Tcas*Or19	OrF19	TCCACCTCGCGACCACCG	OrR19	ACAAACAGAGCAAAGCTC	AM689921
*Tcas*Or20	OrF20	CGAACCGATTTGGCGTTC	OrR20	GTGAAATATGAATAAGCCG	AM689922
*Tcas*Or21	OrF21	TTGGGGGAGCGACGCTG	OrR21	GCACGTTATTAGGTCGCG	AM689923
*Tcas*Or22	OrF22	AATGGATGCTATGTGGTC	OrR22	GGGCAAACGACATGATACC	AM689924
*Tcas*Or23	OrF23	CGCTACCAGCAACACATC	OrR23	GACATGATCCCGATCGGGG	AM689925
*Tcas*Or24	OrF24	CACTCATCTCACAACCAC	OrR24	CCGCAACAGACAAACTCG	AM689926
*Tcas*Or25	OrF25	CGTCTCTCCCATTATCACC	OrR25	TACCGAAAAGAACGCCTG	AM689927
*Tcas*Or26	OrF26	AGGAGGAAGATGTTGGCC	OrR26	GGGACACGTAACTAAACGGC	AM689928

(A) Nucleotide sequences of the primers that had been used to amplify the *T. castaneum* chemoreceptor (*Tcas*GR and *Tcas*Or) DNAs using the genomic DNA as a template. (B) Nucleotide sequences of the primers that were used in Q-real time RT-PCR and whole- mount *in situ* RT-PCR analysis. The name of the gene target is given at the left, followed by the primer name and the primer sequence (5’-3’).
