# Supplementary material for: A Family of Chemoreceptors in Tribolium castaneum (Tenebrionidae: Coleoptera)
Source: PLoS One. 2007 Dec 19;2(12):e1319. doi: 10.1371/journal.pone.0001319 (PMC2121604; doi:10.1371/journal.pone.0001319)
Supplement: Table S2 — Insect chemoreceptors that are not belong to the GPCR superfamily. (0.09 MB DOC) [file pone.0001319.s002.doc]

**Table S2.** Insect chemoreceptors that are not belong to the GPCR superfamily.

Sequence identifier Global Local Prediction

TcasGr1(AM292322) -41.98 - No

TcasGr2(AM292323) -69.56 - No

TcasGr3(AM292359) -38.49 - No

TcasGr4(AM292325) -26.10 - No

TcasGr5(AM292326) -67.80 - No

TCasGr6(AM292327) -57.98 - No

TcasGr7(AM292328) -60.16 - No

TcasGr104(AM292329) -7.81 - No

TcasGr9(AM292330) -48.42 - No

TcasGr10(AM292331) -32.84 - No

TcasGr11(AM292332) -55.81 - No

TcasGr12(AM292333) -14.60 - No

TcasGr13(AM292334) -6.85 - No

TcasGr14(AM292335) -53.36 - No

TcasGr15(AM292336) -74.05 - No

TcasGr16(AM292337) -79.22 - No

TcasGr17(AM292338) -19.55 - No

TcasGr150(AM292339) -28.70 - No

TcasGr20(AM292341) -44.25 - No

TcasGr21(AM292342) -16.33 - No

TcasGr22(AM292343) -91.82 - No

TcasGr79(AM292344) -64.13 - No

TcasGr123(AM292345) -67.74 - No

TcasGr25(AM292346) -71.22 - No

TcasGr26(AM292347) -57.98 - No

TcasGr27(AM292348) -57.81 - No

TcasGr28(AM292349) -82.49 - No

TcasGr29(AM292350) -60.16 - No

TcasGr30(AM292351) -59.03 - No

TcasGr31(AM292352) -38.86 - No

TcasGr32(AM292353) -89.62 - No

TcasGr33(AM292354) -50.65 - No

TcasGr34(AM292355) -21.98 - No

TcasGr35(AM292356) -84.04 - No

TcasGr105(AM292357) -47.90 - No

TcasGr37(AM292358) -47.90 - No

TcasGr38(AM292359) -62.75 - No

TcasGr39(AM292360) -36.55 - No

TcasGr41(AM292362) -25.86 - No

TcasGr42(AM292363) -29.36 - No

TcasGr43(AM292364) -42.31 - No

TcasGr44(AM292365) -25.85 - No

TcasGr45(AM292366) -7.81 - No

TcasGr46(AM292367) -172.09 - No

TcasGr47(AM292354) -57.98 - No

TcasGr48(AM292369) -49.80 - No

TcasGr49(AM292370) -67.03 - No

TcasGr50(AM292371) -30.44 - No

TcasGr51(AM292372) -94.33 - No

TcasGr52(AM292375) -31.16 - No

TcasGr53(AM292383) -69.29 - No

TcasGr54(AM292373) -58.11 - No

TcasGr125(AM292376) -52.18 - No

TcasGr56(AM292377) -6.01 - No

TcasGr57(AM292378) -24.10 - No

TcasGr98(AM292379) -25.95 - No

TcasGr59(AM292380) -23.94 - No

TcasGr60(AM292381) -60.64 - No

TcasGr61(AM292382) -35.86 - No

TcasGr62(AM292374) -48.36 - No

TcasOr1(AM689931) -15.88 - No

TcasOr2(AM689904) -48.64 - No

TcasOr3(AM689905) -54.50 - No

TcasOr4(AM689906) -62.50 - No

TcasOr5(AM689907) -57.07 - No

TcasOr6(AM689908) -76.91 - No

TcasOr7(AM689909) -71.02 - No

TcasOr63(AM689910) -42.93 - No

TcasOr9(AM689911) -89.37 - No

TcasOr10(AM689912) -80.91 - No

TcasOr11(AM689913) -87.84 - No

TcasOr12(AM689914) -61.29 - No

TcasOr13(AM689915) -44.14 - No

TcasOr56(AM689916) -62.34 - No

TcasOr15(AM689917) -82.80 - No

TcasOr16(AM689918) -75.68 - No

TcasOr17(AM689919) -112.80 - No

TcasOr18(AM689920) -57.34 - No

TcasOr19(AM689921) -72.93 - No

TcasOr20(AM689922) -61.45 - No

TcasOr21(AM689923) -112.67 - No

TcasOr22(AM689924) -57.80 - No

TcasOr23(AM689925) -60.36 - No

TcasOr24(AM689926) -67.33 - No

TcasOr25(AM689927) -101.02 - No

TcasOr26(AM689928) -61.71 - No

AgamOr1 -78.64 - No

AgamOr2 -47.26 - No

AgamOr3 -56.70 - No

AgamOr4 -30.85 - No

AgamOr5 -82.26 - No

AgamOr6 -61.54 - No

AgamOr7 -69.83 - No

AgamOr8 -16.91 - No

AgamOr9 -75.31 - No

AgamOr10 -33.35 - No

AgamOr11 -50.57 - No

AgamOr12 -48.87 - No

AgamOr13 -42.30 - No

AgamOr14 -45.08 - No

AgamOr15 -38.32 - No

AgamOr16 -68.22 - No

AgamOr17 -39.27 - No

AgamOr18 -58.02 - No

AgamOr19 -53.84 - No

AgamOr20 -50.25 - No

AgamOr21 -59.24 - No

AgamOr22 -54.88 - No

AgamOr23 -67.71 - No

AgamOr24 -34.04 - No

AgamOr25 -65.54 - No

AgamOr26 -21.85 - No

AgamOr27 -44.05 - No

AgamOr28 -34.42 - No

AgamOr29 -51.50 - No

AgamOr30 -58.01 - No

AgamOr31 -41.09 - No

AgamOr32 -60.65 - No

AgamOr33 -94.73 - No

AgamOr34 -26.67 - No

AgamOr35 -63.34 - No

AgamOr36 -86.05 - No

AgamOr37 -52.79 - No

AgamOr38 -66.14 - No

AgamOr39 -54.07 - No

AgamOr40 -65.58 - No

AgamOr41 -46.34 - No

AgamOr42 -51.31 - No

AgamOr43 -69.68 - No

AgamOr44 -65.81 - No

AgamOr45 -76.10 - No

AgamOr46 -73.35 - No

AgamOr47 -68.41 - No

AgamOr48 -69.81 - No

AgamOr49 -49.46 - No

AgamOr50 -59.69 - No

AgamOr51 -39.77 - No

AgamOr52 -75.12 - No

AgamOr53 -46.66 - No

AgamOr54 -49.31 - No

AgamOr55 -44.28 - No

AgamOr56 -46.29 - No

AgamOr57 -43.25 - No

AgamOr58 -59.30 - No

AgamOr59 -80.56 - No

AgamOr60 -74.45 - No

AgamOr61 -63.30 - No

AgamOr62 -58.81 - No

AgamOr63 -62.31 - No

AgamOr64 -30.43 - No

AgamOr65 -72.80 - No

AgamOr66 -43.93 - No

AgamOr67 -43.46 - No

AgamOr68 -55.42 - No

AgamOr69 -53.43 - No

AgamOr70 -55.19 - No

AgamOr71 -61.28 - No

AgamOr72 -61.17 - No

AgamOr73 -61.31 - No

AgamOr74 -60.24 - No

AgamOr75 -67.23 - No

AgamOr76 -67.98 - No

AgamOr77 -66.88 - No

AgamOr78 -68.46 - No

AgamOr79 -68.42 - No

DmelOr1a -53.21 - No

DmelOr2a -69.03 - No

DmelOr7a -66.70 - No

DmelOr9a -48.40 - No

DmelOr10a -56.32 - No

DmelOr13a -55.10 - No

DmelOr19a -57.91 - No

DmelOr19b -59.46 - No

DmelOr22a -65.25 - No

DmelOr22b -41.53 - No

DmelOr22c -59.94 - No

DmelOr23a -59.79 - No

DmelOr24a -49.65 - No

DmelOr30a -51.29 - No

DmelOr33a -52.99 - No

DmelOr33b -66.45 - No

DmelOr33c -64.79 - No

DmelOr35a -98.58 - No

DmelOr42a -51.13 - No

DmelOr42b -65.13 - No

DmelOr43a -51.73 - No

DmelOr43b -48.17 - No

DmelOr45a -75.67 - No

DmelOr45b -64.62 - No

DmelOr46a -61.69 - No

DmelOr46b -64.52 - No

DmelOr47a -44.45 - No

DmelOr47b -52.65 - No

DmelOr49a -64.39 - No

DmelOr49b -57.91 - No

DmelOr56a -70.47 - No

DmelOr59a -52.86 - No

DmelOr59b -51.33 - No

DmelOr59c -49.51 - No

DmelOr63a -63.74 - No

DmelOr65a -42.20 - No

DmelOr65b -41.60 - No

DmelOr65c -31.77 - No

DmelOr67a -51.09 - No

DmelOr67b -52.77 - No

DmelOr67c -59.20 - No

DmelOr67d -50.49 - No

DmelOr69a -38.72 - No

DmelOr69b -42.20 - No

DmelOr71a -46.95 - No

DmelOr74a -54.78 - No

DmelOr82a -61.35 - No

DmelOr83a -61.07 - No

DmelOr83b -81.59 - No

DmelOr83c -60.90 - No

DmelOr85a -48.20 - No

DmelOr85b -59.88 - No

DmelOr85c -45.47 - No

DmelOr85d -32.69 - No

DmelOr85e -79.16 - No

DmelOr85f -40.94 - No

DmelOr88a -68.73 - No

DmelOr92a -65.81 - No

DmelOr94a -49.94 - No

DmelOr94b -45.82 - No

DmelOr98a -53.13 - No

DmelOr98b -29.88 - No

AmelOr1 -53.50 - No

AmelOr2 -81.57 - No

AmelOr3 -47.16 - No

AmelOr4 -44.83 - No

AmelOr5 -41.52 - No

AmelOr6 -38.44 - No

AmelOr7 -34.35 - No

AmelOr8 -69.76 - No

AmelOr9 -61.73 - No

AmelOr10 -52.56 - No

AmelOr11 -67.02 - No

AmelOr12 -53.45 - No

AmelOr13 -73.43 - No

AmelOr14 -64.23 - No

AmelOr15 -69.43 - No

AmelOr16 -59.84 - No

AmelOr17 -50.36 - No

AmelOr18 -68.86 - No

AmelOr19 -82.51 - No

AmelOr20 -61.22 - No

AmelOr21 -67.65 - No

AmelOr22 -51.68 - No

AmelOr23 -67.09 - No

AmelOr24 -52.28 - No

AmelOr25 -59.06 - No

AmelOr26 -62.10 - No

AmelOr27 -66.28 - No

AmelOr28 -62.80 - No

AmelOr29 -57.76 - No

AmelOr30 -64.66 - No

AmelOr31 -75.96 - No

AmelOr32 -51.75 - No

AmelOr33 -51.64 - No

AmelOr34 -57.36 - No

AmelOr35 -49.18 - No

AmelOr36 -76.58 - No

AmelOr37 -67.22 - No

AmelOr38 -61.50 - No

AmelOr39 -71.30 - No

AmelOr40 -75.53 - No

AmelOr41 -67.72 - No

AmelOr42 -67.68 - No

AmelOr43 -63.43 - No

AmelOr44 -69.09 - No

AmelOr45 -64.99 - No

AmelOr46 -65.96 - No

AmelOr47 -56.53 - No

AmelOr48 -60.18 - No

AmelOr49 -71.46 - No

AmelOr50 -61.35 - No

AmelOr51 -54.60 - No

AmelOr52 -54.84 - No

AmelOr53 -63.95 - No

AmelOr54 -62.50 - No

AmelOr55 -54.09 - No

AmelOr56 -48.43 - No

AmelOr57 -52.01 - No

AmelOr58 -58.14 - No

AmelOr59 -46.69 - No

AmelOr60 -47.31 - No

AmelOr61 -56.80 - No

AmelOr62 -69.16 - No

AmelOr63 -48.52 - No

AmelOr64 -52.07 - No

AmelOr65 -49.11 - No

AmelOr66 -33.74 - No

AmelOr67 -44.86 - No

AmelOr68 -23.18 - No

AmelOr69 -40.73 - No

AmelOr70 -43.02 - No

AmelOr71 -3.32 - No

AmelOr72 -30.70 - No

AmelOr73 -45.14 - No

AmelOr74 -51.57 - No

AmelOr75 -34.87 - No

AmelOr76 -52.50 - No

AmelOr77 -73.11 - No

AmelOr78 -39.62 - No

AmelOr79 -45.75 - No

AmelOr80 -38.76 - No

AmelOr81 -37.02 - No

AmelOr82 -48.51 - No

AmelOr83 -38.34 - No

AmelOr84 -32.18 - No

AmelOr85 -33.69 - No

AmelOr86 -54.06 - No

AmelOr87 -33.64 - No

AmelOr88 -48.00 - No

AmelOr89 -46.98 - No

AmelOr90 -37.29 - No

AmelOr91 -50.71 - No

AmelOr92 -56.54 - No

AmelOr93 -49.79 - No

AmelOr94 -50.84 - No

AmelOr95 -58.65 - No

AmelOr96 -44.85 - No

AmelOr97 -63.95 - No

AmelOr98 -70.56 - No

AmelOr99 -80.81 - No

AmelOr100 -55.28 - No

AmelOr101 -74.25 - No

AmelOr102 -62.07 - No

AmelOr103 -69.73 - No

AmelOr104 -75.92 - No

AmelOr105 -62.30 - No

AmelOr106 -83.73 - No

AmelOr107 -70.87 - No

AmelOr108 -67.63 - No

AmelOr109 -79.72 - No

AmelOr110 -73.01 - No

AmelOr111 -74.13 - No

AmelOr112 -50.28 - No

AmelOr113 -43.93 - No

AmelOr114 -61.27 - No

AmelOr115 -61.50 - No

AmelOr116 -18.95 - No

AmelOr117 -16.52 - No

AmelOr118 -30.28 - No

AmelOr119 -28.93 - No

AmelOr120 -37.25 - No

AmelOr121 -64.15 - No

AmelOr122 -52.27 - No

AmelOr123 -32.94 - No

AmelOr125 -59.40 - No

AmelOr126 -30.31 - No

AmelOr127 -30.37 - No

AmelOr128 -35.45 - No

AmelOr129 -48.01 - No

AmelOr130 -47.62 - No

AmelOr131 -32.36 - No

AmelOr132 -30.09 - No

AmelOr133 -40.69 - No

AmelOr134 -32.23 - No

AmelOr135 -27.92 - No

AmelOr136 -40.70 - No

AmelOr137 -75.11 - No

AmelOr138 -57.64 - No

AmelOr139 -36.80 - No

AmelOr140 -79.28 - No

AmelOr141 -57.27 - No

AmelOr142 -44.42 - No

AmelOr143 -36.78 - No

AmelOr144 -47.65 - No

AmelOr145 -46.78 - No

AmelOr146 -38.12 - No

AmelOr147 -27.64 - No

AmelOr148 -28.89 - No

AmelOr150 -34.74 - No

AmelOr151 -45.69 - No

AmelOr152 -33.08 - No

AmelOr153 -39.15 - No

AmelOr154 -36.48 - No

AmelOr155 -39.05 - No

AmelOr156 -28.60 - No

AmelOr157 -34.08 - No

AmelOr158 -19.45 - No

AmelOr160 -43.45 - No

AmelOr161 -48.84 - No

AmelOr162 -55.08 - No

AmelOr163 -40.08 - No

AmelOr164 -38.21 - No

AmelOr165 -41.55 - No

AmelOr166 -36.03 - No

AmelOr167 -39.22 - No

AmelOr168 -64.05 - No

AmelOr169 -65.21 - No

AmelOr170 -78.14 - No

AaegOr7 -80.20 - No

HvirOr2 -69.50 - No

HvirOr9 -47.50 - No

HvirOr8 -48.87 - No

HvirOr7 -72.94 - No

HvirOr6 -50.53 - No

HvirOr3 -80.24 - No

HvirOr5 -75.85 - No

HvirOr4 -66.33 - No

HvirOr1 -28.48 - No

BmorOr20 -67.23 - No

BmorOr19 -77.40 - No

BmorOr23 -39.18 - No

BmorOr22 -53.73 - No

BmorOr21 -58.61 - No

BmorOr6 -48.28 - No

BmorOr7 -43.57 - No

BmorOr8 -62.74 - No

BmorOr9 -64.04 - No

BmorOr10 -58.11 - No

BmorOr11 -57.94 - No

BmorOr12 -53.33 - No

BmorOr13 -46.75 - No

BmorOr15 -77.40 - No

BmorOr16 -39.18 - No

BmorOr17 -53.73 - No

BmorOr18 -67.23 - No

BmorOr2 -68.16 - No

BmorOr1 -51.15 - No

BmorOr2a -68.16 - No

BmorOr1a -51.15 - No

BmorOr5 -46.17 - No

BmorOr4 -40.52 - No

BmorOr3 -67.23 - No

BmorOr1b -51.38 - No

AgamGr1 -15.30 - No

AgamGr2 -96.19 - No

AgamGr3 -69.28 - No

AgamGr4 -68.13 - No

AgamGr5 -45.85 - No

AgamGr6 -47.75 - No

AgamGr7 -77.53 - No

AgamGr8 -75.24 - No

AgamGr9a -61.88 - No

AgamGr9b -48.28 - No

AgamGr9c -49.14 - No

AgamGr9d -36.36 - No

AgamGr9e -42.60 - No

AgamGr9f -38.61 - No

AgamGr9g -39.40 - No

AgamGr9h -25.20 - No

AgamGr9i -45.97 - No

AgamGr9j -55.15 - No

AgamGr9k -60.85 - No

AgamGr9l -53.53 - No

AgamGr9m -51.75 - No

AgamGr9n -63.04 - No

AgamGr10 -60.11 - No

AgamGr11 -58.83 - No

AgamGr12 -36.39 - No

AgamGr13 -49.35 - No

AgamGr14 -45.02 - No

AgamGr15 -34.51 - No

AgamGr16 -51.82 - No

AgamGr17 -42.46 - No

AgamGr18 -50.68 - No

AgamGr19 -52.61 - No

AgamGr20 -55.11 - No

AgamGr21 -61.67 - No

AgamGr22 -27.75 - No

AgamGr23 -51.49 - No

AgamGr24 -42.95 - No

AgamGr25 -27.37 - No

AgamGr26 -33.59 - No

AgamGr27 -66.02 - No

AgamGr28 -34.48 - No

AgamGr29 -75.90 - No

AgamGr30 -51.57 - No

AgamGr31 -36.04 - No

AgamGr32a -30.28 - No

AgamGr32b -45.05 - No

AgamGr33 -30.97 - No

AgamGr34 -35.13 - No

AgamGr35 -31.68 - No

AgamGr36 -22.36 - No

AgamGr37a -45.57 - No

AgamGr37b -28.21 - No

AgamGr37c -53.15 - No

AgamGr37d -31.79 - No

AgamGr37e -39.11 - No

AgamGr37f -42.02 - No

AgamGr38 -28.40 - No

AgamGr39 -6.58 - No

AgamGr40 -35.60 - No

AgamGr41 -39.98 - No

AgamGr42 -14.69 - No

AgamGr43 -60.90 - No

AgamGr44 -67.57 - No

AgamGr45 -55.80 - No

AgamGr46 -43.16 - No

AgamGr47 -59.53 - No

AgamGr48 -32.28 - No

AgamGr49a -58.72 - No

AgamGr49b -49.66 - No

AgamGr50 -51.17 - No

AgamGr51 -39.93 - No

AgamGr52 -27.77 - No

DmelGr2a -86.46 - No

DmelGr5a -48.65 - No

DmelGr8a -67.02 - No

DmelGr9a -51.52 - No

DmelGr10a -64.41 - No

DmelGr10b -32.46 - No

DmelGr21a -20.47 - No

DmelGr22a -69.11 - No

DmelGr22b -50.85 - No

DmelGr22c -51.02 - No

DmelGr22d -58.83 - No

DmelGr22e -57.84 - No

DmelGr22f -37.46 - No

DmelGr23aA -67.83 - No

DmelGr23aB -46.00 - No

DmelGr28a -57.68 - No

DmelGr28bA -24.78 - No

DmelGr28bB -31.48 - No

DmelGr28bC -34.14 - No

DmelGr28bD -38.95 - No

DmelGr28bE -47.83 - No

DmelGr32a -41.56 - No

DmelGr33a -51.65 - No

DmelGr36a -57.07 - No

DmelGr36b -72.42 - No

DmelGr36c -75.68 - No

DmelGr39aA -27.80 - No

DmelGr39aB -41.93 - No

DmelGr39aC -4.08 - No

DmelGr39aD -4.11 - No

DmelGr39b -47.87 - No

DmelGr43a -61.01 - No

DmelGr47a -74.18 - No

DmelGr47b -36.64 - No

DmelGr57a -82.29 - No

DmelGr58a -25.10 - No

DmelGr58b -30.43 - No

DmelGr58c -78.28 - No

DmelGr59a -53.23 - No

DmelGr59b -46.50 - No

DmelGr59c -54.67 - No

DmelGr59d -31.79 - No

DmelGr59e -82.19 - No

DmelGr59f -28.31 - No

DmelGr61a -59.35 - No

DmelGr63a -56.71 - No

DmelGr64a -46.87 - No

DmelGr64b -43.93 - No

DmelGr64c -40.99 - No

DmelGr64d -65.46 - No

DmelGr64e -74.06 - No

DmelGr64f -54.55 - No

DmelGr66a -66.52 - No

DmelGr68a -49.68 - No

DmelGr77a -41.06 - No

DmelGr85a -47.93 - No

DmelGr89a -34.92 - No

DmelGr92a -66.41 - No

Dmelr93a -53.20 - No

DmelGr93b -36.90 - No

DmelGr93c -74.44 - No

DmelGr93d -46.83 - No

DmelGr94a -32.83 - No

DmelGr97a -28.41 - No

DmelGr98a -54.40 - No

DmelGr98b -71.59 - No

DmelGr98c -53.05 - No

DmelGr98d -69.24 - No

AmelGr1 -46.18 - No

AmelGr2 -39.81 - No

AmelGr3 -55.30 - No

AmelGr4 -9.72 - No

AmelGr6 -57.19 - No

AmelGr7 -43.54 - No

AmelGr8 -42.82 - No

AmelGr9 -34.81 - No

AmelGr10 -17.61 - No

AaegGr -37.78 - No

AaegGr1 -50.16 - No

AaegGr61a -46.18 - No

AaegGr64a -71.55 - No

AaegGr64b -51.39 - No

AaegGr64c -37.14 - No

AaegGr64d -71.26 - No

AaegGr64e -49.29 - No

AaegGr43a -47.41 - No

AaegGr21a -62.53 - No

AaegGr21b -28.40 - No

AaegGr28c -27.84 - No

AaegGr93a -19.20 - No

AaegGr63a -49.50 - No

AaegGr28b -38.32 - No

AaegGr28a -95.66 - No

AaegGr64f -65.96 - No

AaegGr66a -89.51 - No

AaegGr28d -35.19 - No

AaegGr64a3 -78.04 - No

AaegGr28e -41.12 - No

The analysis (GPCRHMM) was based on the hidden Markov model that minimizes the common topology of GPCRs. Global is scoring, which takes the sequence through the hidden Markov model, including the N- and C-termini and is calculated by the forward algorithm. Local is a score, which is only calculated for proteins with a global score higher than a threshold of 0.
