# Supplementary material for: A Family of Chemoreceptors in Tribolium castaneum (Tenebrionidae: Coleoptera)
Source: PLoS One. 2007 Dec 19;2(12):e1319. doi: 10.1371/journal.pone.0001319 (PMC2121604; doi:10.1371/journal.pone.0001319)
Supplement: Figure S1 — T. castaneum putative chemoreceptors gene family. (A) The deduced amino acid sequences from the cDNA of the T. castaeum gustatory (Gr) and olfactory receptors (Or). (B) Table showing the T. castaneum chemoreceptor names (accession numbers) and the predicted TM domain positions for each receptor using the GPCRHMM server; http://gpcrhmm.cgb.ki.se/. (C) Alignment analysis of the insect gustatory receptor superfamily including T. castaneum Gr, D. melanogaster Gr, A. gambiae Gr, A. mellifera Gr, and A. aegypti Gr using the http://www.ebi.ac.uk/clustalw/server. The gene names are given at the left and the amino acid positions are given at the right. (D) Alignment analysis of the insect olfactory receptor superfamily including the putative T. castaneum Or, D. melanogaster Or, A. gambiae Or, A. mellifera Or, H. virescens Or, B. mori Or, and A. aegypti Or7. For other details see (C). (0.71 MB PDF) [file pone.0001319.s003.pdf]

A

```
>TcasGr1(AM292322)
MQHEVVKKFLHSVRIVFVQSEIFCLVNFNHRESYFRLSKAKSFCTFVAALVYCSVTFFALSE
LLTDLATSILLKVSSLLIGFCASIYVGTVWINTSINGTKFIEFINKLIEFDVKLQNVSLIIN
YENQRTRSRIHLFVRYVFVTSYLLFDYFVQRNQEYKYYQILSHLSGIFFTVFNVAQCYLTTE
LVLMLQTRFVILNKQLTKITVKNFATKTQSAVLGKICTLHHHLSKLVTRFNEIFGLGLLLMF
AVSFLIITQVIFVICVLVQSEKIVWLHLVYISFLGIICAADVFYICHVCYATIQEVRAKIYF
FYLVKFYKVSKSGELIHKIETNEHEIIDKIEMFSLQILNERAEFNAAGFFPIDYTLVFSVSF
G
>TcasGr2(AM292323)
MEKSTKIFPVPGDKNLHKSYSKMLILAQIFGFFPVQGVRGPDFRSLRFSWKSARVVYALFTL
LGTFLISGFQMQKIATKGLDLLEANRLFFFLTGVMASLLFLNLAKRWPKFVKDWCVVDATFA
SYGWPKGLNKKLNTLTVVFMLIALGEHWLSRYSWAKVFLVEHILVQTNKLVLALECNNTTAE
GFTYFLGNMSFAHIFALVEYDIFKALVLQTINLQITFIWTYNDLFVMLISTALAYRFGQITR
RIAAVASEKIKNEIIWKKLREDYTRQCRLVRKVDKEIAYIVLLTFASDLLFILIQLFNSLRR
MRNDLERLYFYWSFALLITRIVCLCLFGAKVHDESIKPLLTLNSVPTEIYNLEIQRFIQQIG
NSDIAITGKNFFSITRGLILSFVDYFVFLLCSILVNIAFIKLATEWPQLMKAWLKIELLVGN
LGMRRNFRKKLDFIFTFITLLTIGLYFQLCEFSRLNFSVEHLLMELSRAIDSVACSKTVSDG
IRHYYVNVTFPHLFNGLVDYSLWKALIFQISNLQTTFGGTFGDTFIILLSMAFATRMKQSRT
KIEALVKSHIAGAIVTYELVVIQFNLRL
>TcasGr3(AM292359)
MYHQDQAVSILGEAIPKRRSVFLESGVNSADSFKASKVGPAPPIKFINKSSTDKFGNGAIYE
VLKPIYALMRIVGIFPIKNTEPGMFRVAPELLGYSVVVFVVVMGYIGFIEWDKVEIVRSQEG
RFEEAVIDYLFTVYLLPIIINPLVLYEARKLANVVTDWVNFERIYYKLTKKKLSVFFGNKPV
ILTVVLPLLACGVMVVTHITMAHFKIIQVVPYCYINCLIYLIGGFWFMQCDVVGKVASQLAE
DFQMALKHVGPSSQVADYRSLWMLLSKLIRDVGNASGYTVTFLCLYLFLIITLTIYGLLSQL
QAGFSTKDIGLTINAGLAIFILYFICDEAHYASNCLRVQFQKKLLLVELSWMNDEAQQEINM
FLKATEMSPTDISLVGFFDVNRNLFKSLLATMVTYLVVLLQFQISIPEEASPTNSTTITTQT
PN
>TcasGr4(AM292325)
MVRPELDLNSLKCITKVLTILGLLSCSFPKKKLVYCIIFGAIATLTCIEGLRDCPRKYSTPL
AKITTILQRYCSVLLVFLTYFFNILFRKKLLNAVKILSKIDETLNSKPLKPVKIRTKVYLVY
CSSALVLAAMTTFHLIYEAHGFSYKCGIQYHICNTVISATVCFMMLLMLEIWRRFTILNNYL
TIVLGETWTSLSVYHLVEISEIHFDLCQAADDLNRYFEVQVLTIFGVSFYFFISTFFYFFTT
GNIIATYDKQFYIHNALIVRALFLFFQLWATVYTSSQVTREVQSRSNRMTPLKLQPQNFSI
>TcasGr5(AM292326)
MTIQLLSLICKFGGWMGLTPVSVEPTGFSPKGYSLLWILLFTLGVTISGVYRTDFYKKLSPM
RLIVQTCLDFLLLALNISTILTTVRKQQQWAQLIQNLKVVATTKTKAWFLPFVVTNIIFVLF
HTYEAFVWTRIMGVAYYEQYAVEFFQFYAQFIVYFLIYAVLEMLLQKYKTVTYVMTGQLKAQ
NSNLLKSVCADLCLLSECVDLFNSLFGFLVLLLVALTTLQLLIYIQVIVIGTKNTIETVAYS
VIFILWHIVGTFSGIFLCDLIRREIANVQVLAYKIEAKRRDNEIKMFVKIVDASCRNFTAAR
FFELNRRTILGVSNAVITFLIVMVQKASYEKLSPVVLFIQVAADTVLFILNISTIIITARKK
QQWNSLIKILKTVSNRNDKGDIFWFSPFLVANLAFVTIVTYETFVWTQIMGAEFFKLYAVEY
FQMYAQFIVGTFNSVFICDLIEQKVKNIQMLVYQNEAEEVKILLDVINHFPHFTAARFFDLN
RKTILGVLNALFTFLIVVVQFENLTS
>TCasGr6(AM292327)
MEISDLAQLYGNELHIKQISKWLRGSARAQEIQKRSELDSKDGHVIDEHDQFFRDHKLLLVL
FRVLGVMPIQRGEIGRITFGWTSIPMLYAYVFYVVTTVLVVLVGYERFDILLNKSKNLQFPL
LSTLIIIISLGCLILAVVFLLTLSALLEGFTLYHTTAYLHIITMINMNCALWYINCRAVGNA
STALAESFQNVLVPTASNSKSTKVLFKDVDRNCSAYIIAHYRVLWLSLSDLLQKMGNAYART
YSTYSLFMMANITVAVYGFTSEIVDHGIRFSFKEIGLLVDSTYCLFLLFVCDCSHQASLNI
```

ARRVQVTLLQVNLSQVDPATRKEIDIFLVAIQMNPPKVSLKGYTVVNRELVTASVATIAIYL
IVLLQFKISLLNMRG
>TcasGr7(AM292328)
MRVQPSNTPPEKSQLSTADTANLINKSLGKHNLQKSFAKVVILAQIFGFFPAQGILGRDFRA
IHFTWASARVGYTIVTILGATFVTVLQLHKIFAKGLNVIEANRLFFYCSGLASGYLYLKLAM
KWPRFMKDWSCVEVMMASYGWPAGLNRRLNVLLAVFMSLALIEYILMQTNKLVLALECNNST
SEGFDYFFGKMSYSHIFSLMDYNIVMALVLQFITLQHTFIWVFNDVFVMLLSTALAYRFTQV
TDRTQSMSESKNKSESAWKNLREDYNRLCRLCKRVDEEISYIVLMSFASDLLFILIQLFNSL
RQMKNNLERIYFYWSFGFLIVRTVCLCLFGGKVNDESTQPMLVLNSVSADVYNLEIQRFIHQ
IGTLEVAFTGKNFFSITRGLILSIAGAIVSYELVLMQFNDSLLETISEQIDSCPVYL
>TcasGr104(AM292329)
MCYFNYSKKDIRSLSFFYKICNIFGIVPPYSFEKPDSQKSLWKKIQGVVLVSIIAAGTAYSI
YVRHTYYRRLYTITHLVLDYLDEFLIVALAFQVILNSCFCDPAKWIRLNNNLQYIDEVLKNR
DSHETNLLRNVSVQFFVYVILYILATVFLAYVWICEMGVVTLQAYTLHLFCVLYVVLLHFLT
YNLSLGIKLRYDDVNNLFRIEESEEKNIIVSLRQIGSLSQLLSETVALFNDVFGTCLILITG
KSIVQLLTCLNFITNNLKSENEEFKEKLLAANLCLVIYTLVSVSLVMVTCDACTIASKQTIS
WCYKLQEQFVTNSEVRTELFKLAQHVSANIVHITAGNYFEINKATLCGIFGTTTTYFIVILQ
FNQSSAK
>TcasGr9(AM292330)
MEKSTKIFPVPGDKNLHKSYSKMLILAQIFGFFPVQGVRGPDFRSLRFSWKSARVVYALFTL
LGTFLISGFQMQKIATKGLDLLEANRLFFFLTGVMASLLFLNLAKRWPKFVKDWCVVDATFA
SYGWPKGLNKKLNTLTVVFMLIALGEHWLSRYSWAKVFLVEHILVQTNKLVLALECNNTTAE
GFTYFLGNMSFAHIFALVEYDIFKALVLQTINLQITFIWTYNDLFVMLISTALAYRFGQITR
RIAAVASEKVTSEKSWIALNDDYNRLCHLCFLLDEKLSYIILTSFLNNFYFIIFQVFESFYL
HKATTLETVYYFISLGLLILRLVSVCFYGSWINEESKLSLDLLSYVPREVYNKEISRLVETL
KFQSITSAVVTYELIVIQFRKKMERDEKPYSRNSIC
>TcasGr10(AM292331)
MRNDHGSNTHLHPDDAIRRAKIVKVAASPTSANPDEEPDPELLDRYDNFYQTTKSLLVLFQI
MGVMPIERSGKGRTTFRWLSSTSIYAYFIFGAETIFVTMVFKERLYLILRPGKRFDEYIYGI
IFLSILIPHFLLPVAAWTNGTEVAKFKNMWTRFQLKYYQVTGTPIIFHNLTLITYSLCVISW
AVGIGIMLAQYYLQADMLLWHTFGYYHILAMLNCLCSLWFINCTAKGRVAVWMCNNLHKALE
SRNPAKILGAYRDLWVDLSHMMQQLGKAYSGMYSMYCLLILLTTIVASYGSVTEIMDQGISF
KEAGLFMIAFYCMTLLYIICNEGHHATRKMGPEFRERLLNVNLSAVDQKTRQEVHMFLMAIE
KNPPIMNLNGYANVNRKLISSTVTSIATYLVMLMQFRLTLMRNAQLAARRAIANVSVSSGNT
TMS
>TcasGr11(AM292332)
MIKFSKVKSYFTTHHDTLHNIYTSMKPVYAICKLIGLNTLRIGRKGELKQHKSDYFYFSFYI
TSYTLLSVYSLFRIATNENNSLVINKRLIFIECFVMMALTLIVTLFTFLARGTLIKSFDMLS
HVDVSFIKAGFRLEYKQLLKRSYLIISFVLFSLLARVPVMLMTISADFIQQIMLFVSALIKA
FSKYQFVVLVLQLQHRFGKINRTMRSFFSDNKQDDKIPQISDNLYILCRLHYKLTSVMQKIN
SAFSVQLLVSIGVSLFDVLFQAYYLYYVATGKASFVTVPMIVCPIVWLMDEVVEIYLLVYAC
ASTCEQANDTPSILHELRNNYFHMDLENNVIIIL
>TcasGr12(AM292333)
MSNLKKLKFLLKLGQLLGLAPVKFYVQKFYVVILAFLLTAGAIISLVYQAPVYKELTDIKLV
LNILSDVTLWLSNVNSLTLTLKRKKWNLLFKTLTHDEPRKSSLVQLILTQAIFLMFVSYNIY
VCQTVSNFEYFQRKILLMIQLYYLYFDVIVISTFLQLFLEHYEVINRQTLDTADVKTLTTVV
EKECYKMKVTIDAFNDIFGWSFLLFTLYSSLLLLDYVSCVVALIILILQCGRVRSEVVKILN
VLKKIALRRQTTNGDLDELIVTLSTHYFPVFSACDYFTIETKTVLDVLGVTVTYLIVIIQFD
DKIVQV
>TcasGr13(AM292334)
MRFLGESPNGIYLYHDGRVKLLEGRRVVDCDDVSSVSQSEAGDEDRFSFTSSMVQSLDGNSS
DLENSSEENCVSMSNLKKLKFLLKLGQLLGLAPVKFYVQKFYVVILAFLLTAGAIISLVYQA
PVYKELTDIKLVLNILSDVTLWLSNVNSLTLTLKRKKWNLLFKTLTHDEPRKSSLVQLILTQ

AIFLMFVSYNIYVCQTVSNFEYFQRKILLMIQLYYLYFDVIVISTFLQLFLEHYEVINRQTL
DTADVKTLTTVVEKECYKMKVTIDAFNDIFGWSFLLFTLYSSLLLLDYVSCVVALIILILQC
GRVRSEVVKILNVLKKIALRRQTTNGDLDELIVTLSAHYFPVFSACDYFTIETKTVLDVLGV
TVTYLIVIIQFDDKIVQV
>TcasGr14(AM292335)
MPLLPKTPLPLTLLYKLLGIIQFPISAHFSFMSRFLCLPFYSYFFYLSYIYTTYSRKLSGIF
KYIDQMAGYTGFLAMLTSMVMFYKRSNDLKTLLSNLESIQIYSIKPKERNSNHWVRSGLFAL
ITGNVLFYPFLPSDVSYNLFSFVPLVVNALDHLFLNDILSDICDKFEQINQHFRRQIKSVDL
FVIFPLTKAEKVRNLKEDEVTFSVQKIQELSHLHYKLANFTVKISGLFEITTITAMVMWFGY
VIDTMYLFIHIRSRQEDTDTLVVIYTFNLFYLCFCFYWLLVMVAMFSRTQQSANKTATFVHE
IWNKYALKNEVDKRVRHLQLVAIRLLNTKLQFTAKDFFNLDWTFCHMMIAALTTYLVILIQF
NF
>TcasGr15(AM292336)
MDIKSIKTCFYVAKFFAITPSSIYDKPTKLYQKLYPVLMFLIYTITEIYSVYERRYMNAHLS
GIQLVVRNLLDISLYLNAYHVFVTMMTLKRHKWSTLFEILLKLESKDLSRTFYGFVIVAHLL
FLVIFVLNIWCWIVEIGGWLAFTKQLLIKFVEVYPLFVLLIVSNVVLKILVEKYRHQKNFIN
ESNVETVKFNLFLLSQAVETFNDIFGWTILQTVFFTVPRVLMYFDIFITEHESKPNWLFFLA
NFLTNLLFWEFDQIVNVAYRMETWNVVFNGSRKNLDVMKSFVNFRPSFTAARFFEIDRINAE
SRKPIRILFDVSSEIYNVEYKNVEFWRNIREDYDRLARLTQFLDKELSYLILFSIGFNCFWV
MKLLYNVLRFGAESQTINFMFSFGFILLRFLSVFESCTRLNNESRKPSFVIQFSHIPVDNIE
FAGALVTYELTLFQYHAFYLT
>TcasGr16(AM292337)
MPQVKLHKHYVPIILLSKILGIAPISYKNSNVNYSKIGIVQTVAYFVIFIILTIVFFEKRSA
LSPFELIQIRTINFVATFRTIANIILMAVLFLGTFIGRAKFLAILKQMRNLEPEFLKLDQLG
ATVRNNRQIRRMVVFLIGVSLSFNLFGDLLTLFIKEELDLVWREVCTFVVQVYPRLVVNTIN
LTFLTLLMILEGRFRVINDGIQALINDSRKVTRFPTELTFCNKIKHLVNLHKNLVKVAKDHN
SLYSLHLLLWITVTFILLVGDSYIVMYVFFFHLSDVRYVMVVYLLKNVVLYGLELFILAKIV
TDLCQEANSTKKLIVKIKIDIDKEDERNEVISSALKLSQNELEITACKFFSIDNALLFSANS
TKKLIVKIRIDIDKEDERNEIISSALKLSQSELEITACKFFSIDNALLLSICGASSSYLFIM
IQLDLGNKKNETSTILVE
>TcasGr17(AM292338)
MNTFKIFRPIKILAEIFGLSPMTQKLTLSPLKVLQTVILGTTCIYLTSLYIWFILTQMTFSG
VTSPTIMKFVLSFRTGSFTFLMVVAVLSSGTTFKLVAKTLENMTKIDKDLTELGQKKYLEKR
NRHIFRTSVLLIFVMHLMFDTLGGLYSTWSKKSKQVEYFLIHWYPRMLISTKLLAHFLILTV
LKARFEAINNIIDSIRQNSQTRNQAVPTGTIKTLVQMHSNLVTTSEEVCDSFSIFGLVWISL
IFVVLIGDAYAVLNSVLFQNVYKPLPIVLSIVKNCLSYCFNLYHLASKSNQLSREAKVTKTV
LLGVKIDICNEEERNSHCLGGTITRTMWYFQICGSAFSYLFIMLQFDLNNKKNQSLGNGTTA
>TcasGr150(AM292339)
MNTFKIFRPIKILAEIFGLSPMTQKLTLSPLKVLQTVILGTTCIYLTSLYIWFILTQMTFSG
VTSPTIMKFVLSFRTGSFTFLMVVAVLSSGTTFKLVAKTLENMTKIDKDLTELGQKKYLEKR
NRHIFRTSVLLIFVMHLMFDTLGGLYSTWSKKSKQVEYFLIHWYPRMLISTKLLAHFLILTV
LKARFEAINNIIDSIRQNSQTRNQAVPTGTIKTLVQMHSNLVTTSEEVCDSFSIFGLVWISL
IFVVLIGDAYAVLNSVLFQNVYKPLPIVLSIVKNCLSYCFNLYHLASKSNQLSREAKVTKTV
LLGVKIDICNEEERNSVVASVLQLMENKIEITACRLFNIDNALLFAICGSAFSYLFIMLQFD
LNNKKNQSLGNGTTA
>TcasGr19(AM292340)
MTSKARTFTIKCPVKKRKCTVSNVSLHSLKNKRSGASLQVGILDSLVFKCYNLRRIYYFYCV
SITFNVHLLFLLCSGYFTVHLLFYCPFIIFTVHFLLCTYYFYYAFIILLCVYYFYYAFIIFT
VHLLFLLCIYYFVVPLFFLLCIYYFYCAFIIFTVHLLFLLCIYHFFCAFIIFTMHLLFCCAF
IFFNMHLLFLLCLDYFTLHLLFLPCIYYFYSAFIIFTIHLLFYCVLIILLCIYYFYYAFILF
TVHLLLLVCIYYFYYMHLLFCCAFIIFTMHLLFLLCIYYFYCALIILLCIYYFCRAFIIFPT
HFYCAFSLYPLKSTFYLNVVRPFPERSMGITPMIWNSTRRLFVVW
>TcasGr20(AM292341)

MTPHALISSDFHDVIKPLINLTKIFGFLPVCSSQKGQKTCLNLSINYCICGYIFSAILFSLS
ILGLTEDLNSAPIRMKNPIVKYIILIDLGEVAVTVLAGILTTPFKLPYFWKMLTGFGKIEQV
LPPHHAPEEKKRSFWAVTIFLVLAASVIFFDVVVWGLSASDLGVFFKRFLPVYVSYLLIFVQ
QIPFCFFVRVIRLKIEDLNGAFRAGLERVQNGENQWISVMKVNKQPKVIQGKSALSYERLNE
FMRLVEEIEDVIEAINGFYGVHTLLILFSCLLHLVVTPYYLLIEVCNAGYLPFILLQVAWIV
LHLIRLMYQVTTNLVLKLLTSPLLPDIKEQVEILAVQVSNKKIKFSSFGMLKISRSLLTSFG
GALTTFLVILFQYGGIHGNVY
>TcasGr21(AM292342)
MLPKQFLKLFKDPCDVYTAIHPLFYVCTFFGLAPYSLVRVENGKKVFKFAWWPLTRNALLVL
ILLGALTYHAIFDLISFKDSDLQQKLRYFEEVFSSLLSCCSVIFGCIFALKVIEVFKNIEEV
DVAFRSLAVWVPYKHLYVNILIHLSGLVTIVATLTVTIIFFASYQYGTKTYSLFIVFMTVIL
PYFINLLMELQYCHYLNILRVRYQLLNEYLETLVQETNRTSVEGWTDVSNVKRKSKEISKLP
KSMLAISDPVFIVDQVAALHIKLTDTAHMINYAFCVQQLLRITVAFISIVTALFLVAINFNK
SSSEENEGKTTQLDYFFTFWAFSNACEVMAIVWITSETCEEIVAGVTTYLVILIQFNNSDFV
QRNSTEFDNATESY
>TcasGr22(AM292343)
MFRDLGSTTMMVLNLFFSQMYFTKKLEQMRQVHEVDNQLKQMGCDLDIPNSVVQKHLILLMV
LVNLIFNTCGEVFSAWVRCQDQILIFLVNWYPRLIIGIMNSTLNLIFLLIQTRFEMINNIIT
RGDVTSTTIKKLFNLHKILVKVVREINGIFAFQVLMCTSMNFVLLIGDLHTSIYIIFFDMFY
QHHKIVLDMGKNCVTYVFDLFYLSKRASDLCNEANKTKILLVGLKIDIDQEEERNVVVTSVL
KLMQNKLEITACRLFSIDNALLFSICGAASSYLFIMLQLDIGSKTQGTNSTLY
>TcasGr79(AM292344)
MNSIPIETIFKFGKYTLLTPKSRIEKKPSRQKQFYALFLIFFYTFGEVFTLVLRIRNMEYRS
QTLMQTTLRLIRDLSLYFSVIYSLLRVKRMMRSWYYLLTNLTYNNTNRKYYWLNVCCLNLSI
VTIFFNFATKKLEQFIVRFMKYHFFGTLKLWSQLFSTFAGIEVLHVVKQCYHLQKINVQQSG
IFINLPFLEHNLIRLKNCVIYFNRIFGWNILFGHIFTVCRTLIYIDDNVKGLGKQYNIASSK
TLKLSINIISLVQLWIFQLYLLILCDQILREFDQIVVILSKVKSILNKKCCEKCGLKKIEFC
RPTFSAARFYGIDFSNTFLGLVGAVVTFLIVLLQFQIN
>TcasGr123(AM292345)
MSDFQKLYQKFVNSIRILLFQSRIFGLVTFTPDRSKFRPSSLRFCLNILSIFTYVPMILYCV
YTTATYDSFMIYKTTNIIILGLNVVYVVTAWICAITKRDMFVEFLLKLVDFDSKLQTMNIKV
NYHRVHRKIMVQSLGRVGVLVVIIGTLAIIANLNGDTMIVEAMAYILLAMNSAVCHQTIELV
KMLKIRFVILNKQINNLIEYFQKNKIGPVETKGTNKQLNTLNKICALHHHLSKLVKLFNETF
GIVLLLMFGVSFVVIVITIFFFTANVQAGELYFMSLLNPILSNVTFVIDVVYVCDVCYSTIE
EANKAGELIHKIDTEDHDIRDEIEMFSLQIANEQVEFNAAGFFAINYTLVFSILGGVTTYII
ILIQLATSLAGQ
>TcasGr25(AM292346)
MSQVMLIQFCAFVVVLKQKFCVVNQYIKQVCKLNNGHYKNFLSQVEIIHNEILVTHNEIQTI
FSVPLLMKIASQFVGIFCSLYFCIFGYIYDDEMVVPQNFHDIFLPLLCILTNTLEILITVTV
CELTILEYKRTKKLLYRIPVTKTDSMLIRNINLFSLQLAHQKLEFSACGFFLINGTLLHTIV
GAVTVYLIMFIQFDIATTTKGAAPAVSKARQFLPALPGYVPVYIRPGDTPLEDINPDLAEAF
SSYARKNARLSFGRAEPVFDGKVPSNGSLNQISDIDDVNFQEENLALTSTEHSLSQSQHIQK
IPKN
>TcasGr26(AM292347)
MEISDLAQLYGNELHIKQISKWLRGSARAQEIQKRSELDSKDGHVIDEHDQFFRDHKLLLVL
FRVLGVMPIQRGEIGRITFGWTSIPMLYAYVFYVVTTVLVVLVGYERFDILLNKSKNLQFPL
LSTLIIIISLGCLILAVVFLLTLSALLEGFTLYHTTAYLHIITMINMNCALWYINCRAVGNA
STALAESFQNVLVPTASNSKSTKVLFKDVDRNCSAYIIAHYRVLWLSLSDLLQKMGNAYART
YSTYSLFMMANITVAVYGFTSEIVDHGIRFSFKEIGLLVDSTYCLFLLFVFCDCSHQASLNI
ARRVQVTLLQVNLSQVDPATRKEIDIFLVAIQMNPPKVSLKGYTVVNRELVTASVATIAIYL
IVLLQFKISLLNMRG
>TcasGr27(AM292348)

MSHSNPLEAFKLNTFFLKALTVWHVENPTYRLYKIFVVFSFAVTFFSAWICALVNYNVSEIS
ENFYYLPAMSTGPLKYAIFQKNFTNIVNLTHLLETQYAKIRTENQKKIFDESVIFERKVMKN
FAILIIPTCVAMFIVPYFQDRREMPLIVWFPFDYKQPVVFDLVYFILAFACISIAYTNVSTD
AFFYTCLIQIETQCEIVSDTLRNLDKIVTNGFRNVAESRKIFIECIEQYNVILRYTKIVSDT
YQGILVVQFFCSLVALCLTMYKLSLTEDLYFAIYEMHWYDASKQIQNEVFIFMGQLEKPIVF
YVANIFSLDLDTFKKIMQKAWSFFTALKNMHDIRNN
>TcasGr28(AM292349)
MRLIRKMKSDLVLTIEASDAVLAYPQPSFHQTFSFVVIFGQFFGIMPLHGVSRKNVQEIRLE
WKSFRFVYAVYNIFGAFVMGLFCILKFALDGLMLDKTGICVSNVFDFVHFYYPVATMSFYVL
NFFGSIQFIIISKHWVTIMKEWSFMEMSMRNYGSSINMKKRFVVMTSVIMTLALGNPYCFNS
SVYKVFPVEHLLFIANAFITSQSCENATLYSGDEMYFRVAFPSVFTLIDYSLWKACFVEVRR
>TcasGr29(AM292350)
MRVQPSNTPPEKSQLSTADTANLINKSLGKHNLQKSFAKVVILAQIFGFFPAQGILGRDFRA
IHFTWASARVGYTIVTILGATFVTVLQLHKIFAKGLNVIEANRLFFYCSGLASGYLYLKLAM
KWPRFMKDWSCVEVMMASYGWPAGLNRRLNVLLAVFMSLALIEYILMQTNKLVLALECNNST
SEGFDYFFGKMSYSHIFSLMDYNIVMALVLQFITLQHTFIWVFNDVFVMLLSTALAYRFTQV
TDRTQSMSESKNKSESAWKNLREDYNRLCRLCKRVDEEISYIVLMSFASDLLFILIQLFNSL
RQMKNNLERIYFYWSFGFLIVRTVCLCLFGGKVNDESTQPMLVLNSVSADVYNLEIQRFIHQ
IGTLEVAFTGKNFFSITRGLILSIAGAIVSYELVLMQFNDSLLETISEQIDSCPVYL
>TcasGr30(AM292351)
MRVQPSNTPPEKSQLSTADTANLINKSLGKHNLQKSFAKVVILAQIFGFFPAQGILGRDFRA
IHFTWASARVGYTIVTILGATFVTVLQLHKIFAKGLNVIEANRLFFYCSGLASGYLYLKLAM
KWPRFMKDWSCVEVMMASYGWPAGLNRRLNVLLAVFMSLALIEYILMQTNKLVLALECNNST
SEGFDYFFGKMSYSHIFSLMDYNIVMALVLQFITLQHTFIWVFNDVFVMLLSTALAYRFTQV
TDRTQSMSESKNKSESAWKNLREDYNRLCRLCKRVDEEISYIVLMSFASDLLFILIQLFNSL
RQMKNNLERIYFYWSFGFLIIQRFIHQIGTLEVAFTGKNFFSITRGLILSIAGAIVSYELVL
MQFNDSLLETISEQIDSCPVYL
>TcasGr31(AM292352)
MGSVAINHSLKKRLKIITIVILVVATGNIIHTTSNKQLQFFAVEHLLIQCYIAVSIFGSSSF
EADLRQFYKTAYSAIFTVIDFSLCKAILVHAITIRSTFSWTFIDVFIMLTSTAFVFRLKQLN
AKVEMLKNARVKNTALWKQLRYEHYRLYQLSVLIDNNMSYIIIVSFATNLYFIIIQLFGSMK
IVKGTLKTAYYLISFALLIMRLISVCLCGASVHSESSKVLPLLFSVSSSSYNCEVLFSFLID
QF
>TcasGr32(AM292353)
MSFKLLRLVLKVGHFFAITPGFRKNRTTCPEKIYAYGVVAFITLGVAVSVFYRAKDYAKFIH
IKAVVQITLDATLYVQNIYTVLTALTKKPLWFKLLKNLKMVQNHNNIREKSHYCLFVASNCF
FWSYQSYMSYIFANIMGLEFYKQFAIEYFQMYVLFFVNFAFFVVVKMLLVRYRNLTKQLRFK
LRFFEKLGNRRLGFVQRLDQIQFDICLLKEGVDLVNDIFGWQILFLITYATLQILVYLHLAV
MLGFQDIYMIVYIIVVIFWYTVNASTNIFLCDEICNEGQTILGISYSLEKLKTGDEIQDLIS
TIKDNFPRFYAARFFVINRGTILGILDAIVTFLIVMIQFEMTQNSTNINFVSTFFKIPFYST
LRPINLLVQIILDVILLILNVHTILTTITKRNQWCKLLENLKLDRENNSSFVVANVLFQLIH
VYYTVVFTLLLGVDFVKEYAFEYIQLYSQFILYFLLFATLKILLGKYTQLKACLMEEKRPMV
SLQVIKAQIYSLRETVDVFNDIFGWPFLLLITFTSLQIMVYLQHIFVKSRPSPATIISNVTV
ISWQAVCTFYNILLCDSVAHTAGELLGAVYSLDLELKHIENFNDFVTALKDNVPVFAAARFY
AINRSTIFRMFNAIVTFLIVMVQFETNYSQP
>TcasGr433(AM292368)
MEISDLAQLYGNELHIKQISKWLRGSARAQEIQKRSELDSKDGHVIDEHDQFFRDHKLLLVL
FRVLGVMPIQRGEIGRITFGWTSIPMLYAYVFYVVTTVLVVLVGYERFDILLNKSKNLQFPL
LSTLIIIISLGCLILAVVFLLTLSALLEGFTLYHTTAYLHIITMINMNCALWYINCRAVGNA
STALAESFQNVLVPTASNSKSTKVLFKDVDRNCSAYIIAHYRVLWLSLSDLLQKMGNAYART
YSTYSLFMMANITVAVYGFTSEIVDHGIRFSFKEIGLLVDSTYCLFLLFVFCDCSHQASLNI
ARRVQVTLLQVNLSQVDPATRKEIDIFLVAIQMNPPKVSLKGYTVVNRELVTASVATIAIYL
IVLLQFKISLLNMRG

>TcasGr34(AM292355)
MSSQLLKLTHDALKILGIGPKSNKIYSFLLLTTLTLLVILSSIDRPYLKSYTYIKLVVSVLM
DFIAYFFNFWTILFSRSDFFQQFECDNEPKLRHYLIFIFVNVFFWVIILMSNYAFTRIILLK
SVLEIVIIDIEIYSQFLYGFMIYLILDTIKSKYRQMHRLLANYKVITSDEFFYLVSKIESLA
CELKNTVDDFNDIFGISFLLIISYSTLHFVNYIDDLFFFRFEKSKFEPFLISNISLVSLIFL
FNCTLIIMCDCVRSEASKVVKNAQKLRHKFDLKTLVLNFPKFTAGGFFHVKKSTIFSILNTV
STLLIVMVQFDKER
>TcasGr35(AM292356)
MRLKWYYITISMAFIIFVSLEIYHIYTVVEESGFENFVKTYLVSDFENYVTFFCSLFIVVIL
KLILAKYRQQDVTLLQITKAKNLLYSKQIVTILKKTKSNIFILKESVDTFNDIFGWIILCNI
FEAAAKSLIYIDMIIKKNVTQQNSDVFIFDNVWLFILWVGILSTIFLCDTILKKVDDILSQA
YKLEASFDDLATYETDEVQIFIDVVQHNRPEFKAARFFSIDRSTLFSVLNSLTTFLLVMIQF
KEN
>TcasGr105(AM292357)
MYFSRRDIRFLRPIFAVCRLFALVPYYNFEKFSLEHQLWQKIQAWTYLILLTTWTVISASTR
IKSFKFLTIGVGITTDTIDRVFTVAIPFVGIANSCILNQDKWRLLNNNFQQIDKIFKTRDDR
VKVYQAPVLQLFLYIFTYLSVTCYVTYAWSPIDHTESNIINDMCSLYYILQITLIVNYALAL
KNRFKELQKYLKIETSCRYVGTLYRILTTMMEMVNDIFGWTLILILAKCITSSLSSLYTCIA
GYEFNANFTISYILFALFNTFGTILLIMTCSGVHSESQKLVGVCYEYQDRFSDESKKRRELL
RLAQQVNANIAQITAANFFDISRSTFLGILATITTYFIVIIEFGF
>TcasGr37(AM292358)
MLKWVLKVGNVLAMTPTKEKRFPHRIHSYFMVAFYVVFSSVSTFFKIPFYSTLRPINLLVQI
ILDVILLILNVHTILTTITKRNQWCKLLENLKLDRENNSSFVVANVLFQLIHVYYTVVFTLL
LGVDFVKEYAFEYIQLYSQFILYFLLFATLKILLGKYTQLKACLMEEKRPMVSLQVIKAQIY
SLRETVDVFNDIFGWPFLLLITFTSLQIMVYLQHIFVKSRPSPATIISNVTVISWQAVCTFY
NILLCDSVAHTAGELLGAVYSLDLELKHIENFNDFVTALKDNVPVFAAARFYAINRSTIFRM
FNAIVTFLIVMVQFETNYSQP
>TcasGr38(AM292324)
MNNKLNMVLVKSMLQRIKMEEKLVVEKFLNSLQIYLQHNQIFGFVTFTCTRSNFRSSKLLIL
YNIILQVLFVSFVSYWLYLVLEADDMLPIYKNTYLIILFADFAYLETTWICTLLKKDKLLEL
FKRLIHFDTKCQENSTVIDYKRHKKRLLCYLLARYVALALVILFSEILVIVSEQEWSFSTGL
LVMIFNSALSYKASEIVVMLRSRFAILNKQIRFLNQYLRLKPEGRISNRRVFISFSKICYLH
QHLSKSVKLFNEVFGVSLLVLFGNSFLSIVLALFRTAAELQASQIKWTRIAYMALASVPFIF
DSIHLCDVCYSTIGTVSWCELNFDWSSQVSKAGELIHQIQTEDHDIIDEIEMFSLQIANEQV
EFNAAGFFPINYTLVFSVRSVQVERI
>TcasGr39(AM292360)
MRNDHGSNTHLHPDDAIRRAKIVKVAASPTSANPDEEPDPELLDRYDNFYQTTKSLLVLFQI
MGVMPIERSGKGRTTFRWLSSTSIYAYFIFGAETIFVTMVFKERLYLILRPGKRFDEYIYGI
IFLSILIPHFLLPVAAWTNGTEVAKFKNMWTRFQLKYYQVTGTPIIFHNLTLITYSLCVISW
AVGIGIMLAQYYLQADMLLWHTFGYYHILAMLNCLCSLWFINCTAKGRVAVWMCNNLHKALE
SRNPAKILGAYRDLWVDLSHMMQQLGKAYSGMYSMYCLLILLTTIVASYGSVTEIMDQGISF
KEAGLFMIAFYCMTLLYIICNEGHHATRKMGPEFRERLLNVNLSAVDQKTRQEVHMFLMAIE
KNPPIMNLNGYANVNRKLISSTVTSIATYLVMLMQFRLTLMRNAQLAARRAIAN
>TcasGr40(AM292361)
MVFAELWKKIRNRGTPPKFELPTTHNCLKKVLLLSQIVGVFPLNHLNEEPEKLHFTFKSWKV
LYTSLTSFGYLFCASLSFYKAFKIGILLNQLITPLFFFHSFMTSVLFSQVASRWPLFLNEWT
KIEINLLKHYQSTTDLHKKIRYSAFGMIFVALLEHCFSMLNYVYSSKCENNSTGVEHFFKKQ
FHYIFTYMPYNIVFGLCLTTFGLLPVNGVTSEKCNLTFSWYSKKVFYSKIIISGSIFMTTTS
FYRILNSGYNLTTFGSFIFNANSAIEGIIFFNLAKSWPQLIEKWSRVEMALDNWKNDKSLKR
KFYLTICTIMSAAAVEHILSIVNTCSQIPSEVEDKYTTYFLNTYPHLFNFFEFSVPLAIFAV
V
>TcasGr41(AM292362)

MIEVGKTRVKRASNQLFRFGQFCAICVPKITSTNNEVPRWYICYTIGVIITIGCLCIWSIFN
KWNQFRSYSNFSVFLFDIITIVTLTSTSILLIVNAVFVKEKRIRTLLIKLSEIKENTRLAEK
QARISILELVAVHLVLAFLYSFDLFANGITFGWHTYIFDVPNFVNEYVQAIEVLYICSYTVL
IRNKISNLNTSLIHSTHLNGDIVKEHQNLHNKICNLIDEFNECFGLQILGILFVGVIYTTYT
IFLLLIFGSGSATPENNVSVVYVLILYILESLLYMVTKLVVVTFLKFIKKIQIFGIMITKAC
MSASNLIQRTPNICYKLLSNLGIFPKTTNEQLLRKELLLFAEQMGQRQVAFKVGGFLNIDYG
TLYSLFGSTAMNVIILLQFQKQGKYN
>TcasGr71(AM292363)
MTDHNLLRLIVTIGEFFAMTPSRPKTRAKIYALCVVTALVTPSALSIIYYRQPIYAKISETK
SIVAMVMGTIVNAFSCYTVLAPVLCKRQQYCQLMSKLLGNHQLVYNCHTFGRFLAPNLTYLL
VALYSGYVWTDILGFGYFREYLAEAVQLYFQFYYTYFLCVIVCIFRGKYRNINLLLTEQLQN
RRFFAKKMEGLVCSIKELNMIFNEFFGWPIIMIILYSSLMVLNYIDEVFNNNFGYDKKQYIG
VVVANVGVAFMINVGTVTLILSCDAVLEEVKTTLLLAYKIRQVFPNEKKNISEFINVVLNNY
PDFSAAGFFSINKTTLLQIIGNVTTFFIIIIQFNNKH
>TcasGr43(AM292364)
MDFQLLQIMFTIGKFFALTPRSIKLEKQTFIQKFYAVAFFLALSIGVVVSIYSKSFHRNEIH
IKIVLMFFKEASLYCFNYYVIVVTTFYKRRTWTRLLKNLESCAKIKKTKRYCYYYYAFFGVH
LAYGVVIIYSICVQTEKSVFDFLCRHTVEYFQNYIHFFYQLLLYLITDMILMRYKELNRVIL
DNMSRNHLFLVRRTETLAQILNQTVNFYNSAFGWPIAFLIFFATLDFLDNVDTIMYEKTNVT
FVHLAVLNILRVGKNFVCLLSLIILCDCVVKEAQKSLVLTYEMRWYCQTATMEEKQELYEFS
NFVSQHLPKFSAANFFDIERSTILSVLGTACTFLIIIVQFRNS
>TcasGr44(AM292365)
MTRAMVRPELDLNSLKCITKVLTILGLLSCSFPKKKLVYCIIFGAIATLTCIEGLRDCPRKY
STPLAKITTILQRYCSVLLVFLTYFFNILFRKKLLNAVKILSKIDETLNSKPLKPVKIRTKV
YLVYCSSALVLAAMTTFHLIYEAHGFSYKCGIQYHICNTVISATVCFMMLLMLEIWRRFTIL
NNYLTIVLGETWTSLSVYHLVEISEIHFDLCQAADDLNRYFEVQVLTIFGVSFYFFISTFFY
FFTTGNIIATYDKQFYIHNALIVRALFLFFQLWATVYTSSQVTREVQSRSNRMTPLKLQPQN
FSI
>TcasGr45(AM292366)
MCYFNYSKKDIRSLSFFYKICNIFGIVPPYSFEKPDSQKSLWKKIQGVVLVSIIAAGTAYSI
YVRHTYYRRLYTITHLVLDYLDEFLIVALAFQVILNSCFCDPAKWIRLNNNLQYIDEVLKNR
DSHETNLLRNVSVQFFVYVILYILATVFLAYVWICEMGVVTLQAYTLHLFCVLYVVLLHFLT
YNLSLGIKLRYDDVNNLFRIEESEEKNIIVSLRQIGSLSQLLSETVALFNDVFGTCLILITG
KSIVQLLTCLNFITNNLKSENEEFKEKLLAANLCLVIYTLVSVSLVMVTCDACTIASKQTIS
WCYKLQEQFVTNSEVRTELFKLAQHVSANIVHITAGNYFEINKATLCGIFGTTTTYFIVILQ
FNQSSAK
>TcasGr46(AM292367)
MEEKLVVEKFLNSLQIYLQHNQIFGFVTFTCTRSNFRSSKLLILYNIILQVLFVSFVSYWLY
LVLEADDMLPIYKNTYLIIILFADFAYLETTWICTLLKKDKLLELFKRLIHFDTKCQENSTVI
DYKRHKKRLLCYLLARYVALALVILFSEILVIVSEQEWSFSTGLLVMIFNSALSYKASEIVV
MLRSRFAILNKQIRFLNQYLRLKPEGRISNRRVFISFSKICYLHQHLSKSVKLFNEVFGVSL
LVLFGNSFLSIVLALFRTAAELQASQIKWTRIAYMALASVPFIFDSIHLCDVCYSTIGTVSK
AGELIHQIQTEDHDIIDEIEMFSLQIANEQVEFNAAGFFPINYTLVFSVLKQYVMQQQVKNF
RHSVRSVFFLSEIFGLVNLKYRETYFRLSKTKTFCTLVTALVYCSLAIFVLCELLIEGTTSI
LINVPSLIIHVSTSAYVATVWINSVINRWKFIEFIRKVLEFDVKCVSNYTKQQSKIHLIVRS
VFVTTYLMFDYCTVLRVQRFNNYQSLAHYLRVFFTVFNVVHCYLASELVLMLKNRFVTLNVQ
LTKLTKNCATKAQSVVLGRICTLHHHLSKLVTRFNEIFGLGLLLMFGVSFLLITQTIFIICV
IVQSEQIAWLHLLYIFLVGIMYAADVFYICHVCCSTIHEVSKAGELIHKIETNDHEIIDKIE
MFSLQILNERAGFSAAGFFPIDYSLVFSETKLLLFKITSVMIAVSGLVYIATVWICTLMNRC
KFSEFMTKLLEFDVKLQSDRLMIDYKTQKTGSKIHLFVKCVFLMGYQIFDWVQKEQSLAHSV
GVFLTIFNAAHCYLATELVLMLKNRYIILNGRLIKMETKAQSVALGKICTLHHHLSKLIRLF
NEIFGHGLLLMFGISFLLVTQTIFALCVVLQLQENDWLQLGYLIFVSTLYTANVVCICHVCY
STIQEVSKSGELIHKIATNDHEIIDKIEMFSLQILNERAGFTAAGFFPIDYTSAFSSEKIVW

LHLVYISFLGIICAADVFYICHVCYATIQEVRAKIYFFYLVKFYKVSKSGELIHKIETNEHE
IIDKIEMFSLQILNERAEFNAAGFFPIDYTLVFSKFTSSIRMLLIQGQIFGLITFGCSNRCF
FPSKIRICWNVLLNCVYLFLCGFCVYEFASDERIKLVIKAIIIMILSGCIIFTEGIWICSLI
HRKKIVKFLDGIMAFDIQLKQVVNYKKKKFQRMVVARYVYYATIFTIITATSSFVTIHNVIG
QFLGYFIGLITFVMSQHCVELVSMIKARFVVVNQQIGGIVTYFSTNLPKRETEVRKKSLDFG
KLCSLHHHLSKLIKSFNEIYGVPLLLFFGLNFLIITQAMFLVVGQLQASQIHWQKIIIIVMS
SVTYGIDTVAVCDACYSTIEEKNLQVNESGKLIHKIDAQDCDFVDEIEMFSMFERFHVNKLH
FLLQIIGGVTTYIIILIQLSASLKQ
>TcasGr47(AM292354)
MDLEVLEIYFTVANKFSGKLWPISIVLIYTVGVVYSFKRRIFYQDESPISRLVYIGTDLSYF
ISNVYQILAYNFWKKKYWSEFLANLKLLKGRKIRKNLYYLLLFVINVLYVLTLCYAVYYWRQ
QYEDFWHRYTLSFIENYCQFLYNCFHSTLLLMILSRYQRLKAQLWQNRYETVAHGMTVQKTT
LFFFNRTFGVPIFLVLVFVQMQSVQDLSDIFYYKDTAQLVSLALLMQLTQLVPALFVIYLCG
RVMEEQNKIRLITFEMNKSFERDKNKLGDFVALIRDAQVKITAADFFILDKSTILKVLDTVV
AFLMVVAQFQD
>TcasGr48(AM292369)
MHTLQHFTIDTRSTHSMKNFKYLKVLVTFAHFICLFPITINIRKNGLNYNFTKKCYFLRMVL
IDLLIVGCILKHIFNILMKNVTLNDVVFIFCSAPIIITILDIEILGYVNKAKFGKLLINLYT
INYNFKESERNDYVSLLQLVFVFCYFIYLFTVYYIYYSVHEASIINFGYTLAKFMIFTSTCI
YTNLLRIIEADFSKLNHLLSGTENLDLVLPIYSQLVFMCKKINKLYGHQLLLTILTYLIWTI
YEMYHLAILWSCTSTNCPRFLIMLALSYTVIQEVMLFTILWNCQNTRVASEDFKAIWYSILI
KKADSFCKKKLENYSLQLINHRVVFTAMGLYVLNMEHFFSVKCSIQSESLLNDILFPVAGFI
SDFDCDINSIQYFCLMARCLNTLIFYYHISTYEYSI
>TcasGr49(AM292370)
MCQIKNKLLLLDSSTQPNYLILSNKNNTYLVLNKYLLVKHSKPKMPLLPKTPLPLTLLYKLL
GIIQFPISAHFSFMSRFLCLPFYSYFFYLSYIYTTYSRKLSGIFKYIDQMAGYTGFLAMLTS
MVMFYKRSNDLKTLLSNLESIQIYSIKPKERNSNHWVRSGLFALITGNVLFYPFLPSDVSYN
LFSFVPLVVNALDHLFLNDILSDICDKFEQINQHFRRQIKSVDLFVIFPLTKAEKVRNLKED
EVTFSVQKIQELSHLHYKLANFTVKISGLFEITTITAMVMWFGYVIDTMYLFIHIRSRQEDT
DTLVVIYTFNLFYLCFCFYWLLVMVAMFSRTQQSANKTATFVHEIWNKYALKNEVDKRVRHL
QLVAIRLLNTKLQFTAKDFFNLDWTFCHMVSHKWLNYINQFEANFR
>TcasGr50(AM292371)
MSLYSSINSLVLISKIFALLPVKKHHRLEKLVPCVYLTSFSVVLSLGSFIVSVYLTTKIDND
GESISLASGWLDLYLGITIQTLGIISNCINAKQITIFFDKIQRIDKQFGLLGYSINYRRISI
FVNLAILGVFGEFVLVFVPDYVFFVENEPILYLIFSYCPIVTTGIIKIQFATCAHLILQRVN
HVKKILEKETQTVAFPKDNHFLALLDIVHGVHNELCNLCQVANSIYGFQNFLFVLSTFSICI
TQFYYCYDSGFNSDNLTDVYFTLQWAFLQILEILALAYLCDKVQLKIGEIKYSVNKLIFRSN
DPKVTLELCGMISTYLIIMIQLDLARTVPKFENKNKETEQ
>TcasGr51(AM292372)
MNMILKLSLKDIVFINPLVKYLNIFFITPWYDFPTNQKYYPSLAKCYACLLMVVKILWVIYW
LQDDALKSVYASLLCTEKIFLFAIYTNLSILTTVTIFKSAFWDVDKWRTLFTNLQYIDINLQ
NKGKKESKLMKNFYFWFVLKQVMFLCYSTYGIQVFGTMQRTSFFKTFLNSCLMDVYYEFLLI
SLINILIRSFKSRYKDLNQKLETACEQSKSVEELMNIANYYRILGDTVEIFNSLFGYQIILV
IFDCCLETVSALNGAFLYTINGQGQFNIEMFLCNMSLLTVIPIHVWTSKEKISFLRGFWLSP
AVDMFYEFQIIELMTSVVKSLESRYADLNENFLQCQDEKFESFVYYHRILGETVDLFNSLFG
YQTLLITFHIGSQLVLHTVKTVLKTTMSTFSDITFMRTVWKFFNIFLITPFYNFNENTIHSK
LCKLYATFLIALKLVWIVALFKNENLSEMVKQFLFTQKFTFVAGLATLSVLNLLSVIKSSFL
DINTWKMLVKSFDAIDSKLQTGGKVETSICKNFCFGFITKQLIFMIFVTYQLYCWSSFLKIS
VLQSLWASPTCDLMYEFQIFTFLMSLLQSIKRRYVLLNEKVEAIKTSPKMRHELQKLAQVYR
ILGETIETWNKLFGYQILLITFQSGLNVVSCINFPLGVLDRGNFDIEIALCNGIFLLLTLGS
LLQMVISMDSTVEEAKTFVDRCYKMQEQFTLNFRNSDELIKFISIAKHNVRTFSAGGFFDIR
KNLIFSLIANVAMYFVISVQFNQNQKE
>TcasGr52(AM292375)

MQDSFQYINRSELITSKIVSFVNSIFLVMSAVTIILSSLIFNQEQWSSLNKNFQFIDKKLNN
RNSKSRVFYENVNFQFGSILALFGVVQIYIFSGFIVPSGVVYKIGYWLHQICHICELHTIFL
VCNVLTVFKIRYKDLNKALESSKISVGLIRDTAVLYRLLADNVANFNKIFGWSLIFLFGRIV
LQLLSSLGFLLYASKKQFTFEETFAIIHLSVFVYTLVSVILIIMGCDVVASNSRKTAEMCYK
LQGFYPEEAPQRKELLRFASESRGSVAKFTAADFFEIDRGTFFGILSTTTSYFIIMIQFNMS
L
>TcasGr53(AM292383)
MLKWVLKVGNVLAMTPTKEKRFPHRIHSYFMVAFYVVFSSVSTFFKIPFYSTLRPINLLVQI
ILDVILLILNVHTILTTITKRNQWCKLLENLKLDRENNSSFVVANVLFQLIHVYYTVVFTLL
LGVDFVKEYAFEYIQLYSQFILYFLLFATLKILLGKYTQLKACLMEEKRPMVSLQVIKAQIY
SLRETVDVFNDIFGWPFLLLITFTSLQIMVYLQHIFVKSRPSPATIISNVTVISWQAVCTFY
NILLCDSVAHTAGELLGAVYSLDLELKHIENFNDFVTALKDNVPVFAAARFYAINRSTIFRM
FNAIVTFLI
>TcasGr54(AM292373)
MSFKLLRLVLKVGHFFAITPGFRKNRTTCPEKIYAYGVVAFITLGVAVSVFYRAKDYAKFIH
IKAVVQITLDATLYVQNIYTVLTALTKKPLWFKLLKNLKMVQNHNNIREKSHYCLFVASNCF
FWSYQSYMSYIFANIMGLEFYKQFAIEYFQMYVLFFVNFAFFVVVKMLLVRYRNLTKQLRFK
LRFFEKLGNRRLGFVQRLDQIQFDICLLKEGVDLVNDIFGWQILFLITYATLQILVYLHLAV
MLGFQDIYMIVYIIVVIFWYTVNASTNIFLCDEICNEGQTILGISYSLEKLKTGDEIQDLIS
TIKDNFPRFYAARFFVINRGTILGILDAIVTFLIVMIQFEMTQNSTNINL
>TcasGr125(AM292376)
MIKFSKVKSYFTTHHDTLHNIYTSMKPVYAICKLIGLNTLRIGRKGELKQHKSDYFYFSFYI
TSYTLLSVYSLFRIATNENNSLVINKRLIFIECFVMMALTLIVTLFTFLARGTLIKSFDMLS
HVDVSFIKAGFRLEYKQLLKRSYLIISFVLFSLLARVPVMLMTISADFIQQIMLFVSALIKA
FSKYQFVVLVLQLQHRFGKINRTMRSFFSDNKQDDKIPQISDNLYILCRLHYKLTSVMQKIN
SAFSVQLLVSIGVSLFDVLFQAYYLYYVATGKASFVTVPMIVCPIVWLMDEVVEIYLLVYAC
ASTCEQANDTPSILHELRNNYFHMDLENNVQSYSLQLLHQKVQFSVLGFFVVDYTLLYSIVG
AVTTYLVIFIQFDQSSNSRNNYVLTNNSTC
>TcasGr56(AM292377)
MLPKQFLKLFKDPCDVYTAIHPLFYVCTFFGLAPYSLVRVENGKKVFKFAWWPLTRNALLVL
ILLGALTYHAIFDLISFKDSDLQQKLRYFEEVFSSLLSCCSVIFGCIFALKVIEVFKNIEEV
DVAFRSLAVWVPYKHLYVNILIHLSGLVTIVATLTVTIIFFASYQYGTKTYSLFIVFMTVIL
PYFINLLMELQYCHYLNILRVRYQLLNEYLETLVQETNRTSVEGWTDVSNVKRKSKEISKLP
KSMLAISDPVFIVDQVAALHIKLTDTAHMINYAFCVQQLLRITVAFISIVTALFLVAINFNK
SSSEENEGKTTQLDYFFTFWAFSNACEVMAIVWITSETCEEVSKKCYK
>TcasGr57(AM292378)
MKEVPGWLTPPHSLGNTVTPVSLRFKLYTIVHIFIIIALYAHSSYGRENFIYGSMNMTVAIT
DKIANFMLTFFNVSLRIILVFSKGKVIKSFFNQGYELSKQEIFNCCSKKSFRMNFAIFNLYM
VLLLLFDAYLWISSVGVRMFQYYIGRSFTYYVCNTTIFLIFHSVLPIKRFFTSLGIAFDNIM
KNLICEIDGRHEFFLAEFKSAKTPPNKLNCYDLRRVRKNYNSICELVDAFNNIYGLAMLEII
IVVITYVLNLTDLFLVYGMSKSRNIEGVSFGTNLVILCALWITTLLLFTILLAYGCAGATSE
AEKIAKICFYWLNEIPTMPVSIKDQTIKEELALLAQQSTSRTSKFSAAGFFPVDFTLLGFIF
GSVTSYIIISIQFIE
>TcasGr98(AM292379)
MSYRLSRNDIRFLKVMYKLSHFLSITPNYDFENFVIISPRCDKISAICFLLSTILGTCWIIY
VRIYCKEIRFFEISFEILGSLDSLILLMVLVSIILGSLKTKEWAKLNNKFQYIDEKLKTRDQ
KERNLFKNAYFQLVLSISVYSSSVGYTQYVRIAAKGLPTLKLFALHEFYGFCYLWVLILICN
IALAFKQRYQLINEQMAVRINPKNCASFVIEVRKLSRLLGEMVGLFNDIFGWPLVFITGRFV
IKILVALNFFTSTLEIDNLYLKHKLEISSLVQTAITLVAMCGLVLICDSAKSESQQTVFLCY
KLMEKFPERSHEQQELYMAAQVIEKSVANFTAAGFFDVKRSTLFGILATTTTYLIVTIQFNQ
GLNK
>TcasGr59(AM292380)

MWSENITDVLKHVWFLSKLFLLCPRSIDEKIKRQERHSFYKIYKYTYNIVAVSLTAYMVYIT
TNLRITQKLSIVTQLADLIFSASANASGVATVFFCLFYQNKLIEVISKLHKLDNKLKHMLIW
KSYKRTQIFITCELFFVILLWISFFLNFMLHCNNTTWRCLYRWIVLYTLSKMSQVMLIQFCA
FVVVLKQKFCVVNQYIKQVCKLNNGHYKNFLSQVEIIHNEILVTHNEIQTIFSVPLLMKIAS
QFVGIFCSLYFCIFGYIYDDEMVVPQNFHDIFLPLLCILTNTLEILITVTVCELTILEYKRT
KKLLYRIPVTKTDSMLIRNINLFSLQLAHQKLEFSACGFFLINGTLLHTIVGAVTVYLIMFI
QFDIATTTKGAAPAVSKARQFLPALPGYVPVYIRPGDTPLEDINPDLAEAFSSYARKNARLS
FGRAEPVFDGKVPSNGSLNQISDIDDVNFQEENLALTSTEHSLSQSQHIQKIPKN
>TcasGr60(AM292381)
MILVLLVIVNLGLNGLIDWYTVCLVPLNPYQFFLAVQYPRIVVSTFNIVFYMYTLILEERFK
LINLIIKQEEFFSPKIKRMVLLHKTLVRVAKSLNQVFAPQLLLWVLLGVMVVTVDLHVFFFT
LFNKLLGQYPVGIVGVKNASLFIFDLIFLSFRCTKLCKEANKTAFLWYHIKIDREDEEARDD
FIKLGIKLSNDKLEITACRLFKIDNSLILDLLVFITINFVVLLGDLYVTLYIILTNNQAKYC
TTLIGLIKNCVIVIFELYYLSHVCQNVSTEVIGNILRLMHRRFEITALRLFTIDNKLLFGVL
AYYSTTCDIATILDFWVRCVLSLHCAAVGGSANIMSGCSTSTSTNINTKLFWLY
>TcasGr61(AM292382)
MTKSTVYDTVSWLILLSLLLGLYPSYLQTVGKCQRLKTDKNCTIVIIFHLILFAVLLYFASL
EKNPLFDGQLYSFNTFAKLLIFVVSLSGNSAVFIMLIISFMHKKSFKNFVNNVAALDETLAK
LGQYINYETDYYVCLAMTITGPLVIIGNIAMELWNMPRENIEPLPNVILACHMLSFLMVHQG
ETQFVAANVILKTRFKTINNILENLWAKKLIVVKDKKKSRSDEQTIDICMRCHDQLCDMCGA
INMLFGFPIIIGCLIQFNTIVFSFCYCYYNSKMTKDGVYNTLFFIVYVSLLLGPHPTYFGKT
GRKTVLKTNKYCNFIILFYLSVFVILVYYASLDETPNPVTNGKLYNFNTFAKLLILVVSLSG
TFGFFGTHILSYINKASFKKIVNTVATFDETWAKLGLEINHKEDFRVCLVFTLTGPFFTFCN
VLMEMWNIPRENIDPIPLVVVLTHLIPFMLIHQGETQFVVANIILRRRFALINSILRKLYKN
KYRKLIVDGKKSEEQIVDICIRSHDKLCDVCDSVNRIFGFIIIIGCLIQFNTIVFAFCYCYY
SISIRPISTLGWFFWSLLRIYELARKAAFAHLNSNEARATLNWVSKLIIRSNPSLEEKLQIF
ALQLTHRAPTFTALGLFPINGSFAFTVVGAATTYITIIYQFQVNKPTCGP
>TcasGr62(AM292374)
MMDRINHKKFINTTKLLLWCDQTIGLITFDINTPSFKLSKIRSFLNITASAVLLPFAIYHIF
MHIVPTKLISFYKSTAILEIAFEVIFIVTVWMTGAVKHSKIAHFLNKMIRLDERFQSVGLQI
DTVREKRRIKIHFFLRSLFAIMLPLGILLISQDVIANVLLFIFIVVKSGVAFQTIEFVSIIR
NRFVILNRYIEESISKYKHAELVMPLCKSCDLHHRLSKLIKQLNATYGLILLLMFTSHFIFI
VVSIFYMSAYLISNPIMWDRVMLLTFWSAFFIVNVLYICNQCYNTVEESRIFGLVTFTPDRS
KFRPSSLRFCLNILSIFTYVPMILYCVYTTATYDSFMIYKTTNIIILGLNVVYVVTAWICAI
TKRDMFVEFLLKLVDFDSKLQTMNIKVNYHRVHRKIMVQSLGRVGVLVVIIGTLAIIANLNG
DTMIVEAMAYILLAMNSAVCHQTIELVKMLKIRFVILNKQINNLIEYFQKNKIGPVETKGTN
KQLNTLNKICALHHHLSKLVKLFNETFGIVLLLMFGVSFVVIVITIFFFTANVQAGELYFMS
LLNPILSNVTFVIDVVYVCDVCYSTIEEANKAGELIHKIDTEDHDIRDEIEMFSLQIANEQV
EFNAAGFFAINYTLVFSILGGVTTYIIILIQLATSLAGQ
>TcasOr1(AM689931)
MNNSRPANCTHRPKLSYLFQMRNVPQSYCFVTILFDSVNKLGTQIVNPYSVKQYVMQQQVKN
FRHSVRSVFFLSEIFGLVNLKYRETYFRLSKTKTFCTLVTALVYCSLAIFVLCELLIEGTTS
ILINVPSLIIHVSTSAYVATVWINSVINRWKFIEFIRKVLEFDVKCVSNYTKQQSKIHLIVR
SVFVTTYLMFDYCTVLRVQRFNNYQSLAHYLRVFFTVFNVVHCYLASELVLMLKNRFVTLNV
QLTKLTKNCATKAQSVVLGRICTLHHHLSKLVTRFNEIFGLGLLLMFGVSFLLITQTIFIIC
VIVQSEQIAWLHLLYIFLVGIMYAADVFYICHVCCSTIHEVRE
>TcasOr2(AM689904)
MFKTVTREKVFQKFTSSIRMLLIQGQIFGLITFGCSNRCFFPSKIRICWNVLLNCVYLFLCG
FCVYEFASDERIKLVIKAIIIMILSGCIIFTEGIWICSLIHRKKIVKFLDGIMAFDIQLKQV
VNYKKKKFQRMVVARYVYYATIFTIITATSSFVTIHNVIGQFLGYFIGLITFVMSQHCVELV
SMIKARFVVVNQQIGGIVTYFSTNLPKRETEVRKKSLDFGKLCSLHHHLSKLIKSFNEIYGV
PLLLFFGLNFLIITQAMFLVVGQLQASQIHWQKIIIIVMSSVTYGIDTVAVCDACYSTIEEV
ISGI

>TcasOr3(AM689905)
MSKSEKIHTLATYFDSNIAFLKLTAFWIYDDETTRRKKYLQHAYNIFWIFYLFVAYQPAELL
YVYYSFNDLSVFLRALRDIGNHVSLAYKAFNYFIMRRDILKLMETLQHGNYHYEDCGDFQPK
LIVDEEKKEALKWTKYFLNFCNAICLSMFANGVFTFIFLSDKQYVERNGQRVYHQEQPVNTV
SPFGSGTKLRFFVTFIYTMIALTFYAWTIVALDSLFITIMSCISSHLKILQGAFKTVRARCI
MRTRAERLLKEETLHDPPFLENCVNKEMIRKLESIYSTQTFVQTFISLGEMCFSLYLLSETA
DQNIGNEITYLIATGFELLMYCWFGNRITEASLKISYALYESDWFPTSLSFKKQIIFTMTRM
QKPINVTIGKITPLAFSTFLTIARGAYSFFTFLKQRHGINH
>TcasOr4(AM689906)
MDEEFLIGTFETEKKFLRYGSFYPCGKRIKFIFLGLFMFVYSWTEFLSMITVLFVERDNLTK
LSETLLFCMTQAAFLFKLVNFLYHNKTMLRIESILKNPILNCLDQFEKNIIEKYMIRVKYLA
RLFRILCILTVSFYGLFPFIDEDPDHMLPLPGWFPFDVKTHQIELVIAQTCGIAIGAFLNST
LDILPTILITLGSAQFDILKIRLENITSVDTSKSWLVKKAIKKCVIYHTILLKKIFYGFELW
QIKPKIDSCKFIAKIFRILCILVVLFYTLVPYLDDKEDLSLPLPGWLPYNTKKYYYPTVIFQ
VMSVSVSAYNNSSIDVLTCMLITVASAEFNLLKGALKTIDFHPKGHNTKQLIEAKFENCVNH
HKEIVK
>TcasOr5(AM689907)
MVWSPKDVYDLTLPILPAWKYFGLPAYKIIKTPNFKSLTHNNSKLNYIMVIISTTAYLYYFK
VGDKNTNSFEKHMTMYRLVASFVNYMGAVIFCHKHKNKFLSNIEKCALLQQRLNKLGKKEHS
FNKMAKILLTIYTTKLSSMIVVTVNDIVKISTSESHSTVKLGWYCNFITEMFLFYFLIVLRS
LYSQLLNCTKFKESAQQVKELGKIHKDLNKLAMEVNETFGELMLLRFSGDFIYLIVGLSSNA
VLNAEYETVLDFVWTFTVYVLWIPMALGFIALTVWYFEDILHHVGLQLVIL
>TcasOr6(AM689908)
MSPHDSGTFARIRKIFSILVYTSTVVLSMAELFFNYKDLETVIRATESFFTQYGLAWKIAVF
VVYKTELAQIIRLCDNLWPLDEFGTGHNFQFLHKFLRRFFLLYTGNLALLCTQFAVTAFFDD
QFKSVMVYYGEKESRSQIYDNFVFTLQVIYLYVGCFVVAGFDCFFFYLLGHAVTELKMLTIS
FSCKEIGRNWGYEERFKCSVKHHIHVLELLDKINKVYSVMLLNQHLCSLFVQLTQVMDKVEF
SDPLFFLNVIGMHPFKADKFSKFRLAFSIAVYFAVIFSGVLELIVNSQGLETYARASDTLIP
QCQLVCKIFVLAKYKKQIARLLNGSQRFWDLGQFGARYGNSFGKTHKYLKSFFLLYKVMLTF
TCLQFLAVKIIFKIPKPIAISFGETKGLEPLYDHLYLVLHAMITLVTINLVNGFDGLFFYFI
GHVLTELKMVKVAFGDSPIETNWSEEKRFKFAVRHHRFVLE
>TcasOr7(AM689909)
MYRLVASFVNYMGAESAQQVKELGKIHKDLNKLAMEVNETFGELMLLRFSGDFIYLIVGLSS
NAVLNAEYETVLDFVWTFTVYVLWIPMALGFIALTVWYFEDILHHDARINENLTNLIEATKQ
DYIFRYCFYTGFVFYGASDFTILVLITPVEKLYGHFLNNLENHNLEESVRVFFMLQDLAQST
FNIIEEFMLAKILIDFFYASTDVFYGTASALEVQTSLLATVLSSFIVVLWLLFVVHSIVTIS
FVFGKVKEQHQVMNEIICERFRSKAAKNYRCEAKLLLRGKVNPLKFTICGFLPLDYCLAYTV
S
>TcasOr63(AM689910)
MGFMIQDYDLRNAFSLERKLMLVVGFYPKRDNKHEILYWLSAFFNLLISYGQLTTMIIQMVF
DRSDLSKLTESLLYFFTHFTFLCKLLNFQYYSKDLIEIENFLTDPIFYGYSFEQLDIIKAKI
RSCAFISNAFRICCTFTCSFYCLVPFIDESRKKILPLPGWFPYDTTNYYYSTFFVQSLSLFI
SAYCNTAIDILTWKLITLASAQFEILKENLTKIDYEGGFNETKGALVRCITHHAKIVNYTER
VEAIFSKGIFLQLFGSVIVICTTGFQLIVVPIPSVQFAVLGTYLCGMTTQVATYCYYGHEVM
TTSDAIGMSLYLSNWYASHVKIRKIVMIFLEKTKKPTIVKAGNFITLSLATLTQILRSAYSY
FAVLQRLYKDS
>TcasOr9(AM689911)
MEKSSKLSDKFVFYWTFNCIASSLMPMIASLLGGNKNLPMVVWYPYDPNKTPYFHLTYIWEI
FCISNLGLIYAVLDLVFPCIAIVLGQQFKILASNFKNNVYRALVDSEVSEKIVQTFSKNLHN
DSFNEEIFEIMNSAKFKKNNARYLRKNVKHHQQLLQYCADVSDILSIFLMGKVSAAIFNTLF
MAFSLITTGNRAMIFGLGSYMVSTSIELLIYTYSGQVLTQNADIVGTLYESPWYMCDVHFQR
TFHIVQMRASKIVNVKAGNYFTMSASSYITFMKSLGIVYSALEGVN
>TcasOr10(AM689912)

MATFLLYKTEMLDLIKKSERFWKLDRFGDLQAKNLHSTYPIFQIFFYVYVVILFLTCAMFAL
VNWIFDTGKPISLCYGESEGLETPWVEFYIVLQSVEVTIIFLGITGYDMVFLYYAGSVCIQF
QMLKMAFAERKMNERQFLKAVKHHEFLLQYVEQLGDIYSMWFLLQYFSSLFGICFGLFLISK
EGLPTEPERLSKYFPYIFSFTMQSFTFCMTGTMLSDWSSEISDEIFHSDWSDDQVYKNKTAR
LIVMNRAQRPAKISIGKFLDLNLRSFILSLDISNVIFYVDWLDDEVYKSKTARLVIMNKAQS
PVKLTIGKFTGMDLRTFLLV
>TcasOr11(AM689913)
MGKVKFTEPLEFLNVVGLNPENCSNFSLFRRVISLGFFLVVITLGLLELLLHFEGLETCSRA
SEAMIVQYQLFIKIAVLLKHRKNLVVLMQKTRKFWPLDKFGQDAKIERPHKLLKAFFFAYKL
IMILMALQYILRKFVSKNGKPLAIAFGESKGLSPKVDHLYFVLHSTSTFVVLHAVTGFDRLF
FFLIGHVLTELKLVKKSYRLTQNRREKFLETVQHHAFALEIPPDLGHVTKYVPYVISFITQT
FTFCFIGSLLITWSLQVPDAIFYNDWGKNQAYKYKTDKIIAMIRGQRAAKLTLGGFGDLDLE
SFNLVVKNAFSFFTFVNAMNQK
>TcasOr12(AM689914)
MAHNLDVDLTEFVRFNIKCIQFFGYFSPDFRNNSQRKLLFRIYAALFVGFAFILSLLSQIAN
MVDAFGDIEKMTEASFLLFTNLVQCFKIYTFANHGKKVWNLVYSMNRSDFKPNNLAQYWIVV
NEIKTSKIISKLFLLACTLTCVSWAISPLLDKRGSDELRLPLSGWYPFSTEKSPAFEFAYTY
QIFTTWVGGLGDISMDTFMSGTIMVISTQLSLLKDGLENVARNIKHDKSSVNKNLIQCACHY
RSIIQFAAEVTNLFTTCITAQFVVGVIIVCMSMFQMSLVSVLSFQFAAMLLYQICVLMEIYL
WCFYGNEVMLKSDQLTQAAYMSEWLDGTEEFKQNLLFLMTRTQFPLKLYASGGKVTQSYLLN
LE
>TcasOr13(AM689915)
MSAMSTVIVSGEMFDLFSPVLYLSRIFCLQPLKWIKTDYSYVISKSGPYMAYSILTSLFLIT
ASIYGLTQVYSMEAVYLIRLSSNTDRFVTFSDVVVVLIPCIIGVPIASHNIEKTIKYFSFLR
QFDCCLQKQPPKTWKSFFVPIITIVFTATILLFDAVMWLTLIARNQSIFILSLPYYVCYCFT
MIIEIMFWQFVNSIKIRLILLNERLEQIGGEDFTIVRIDRKKINSIVDTLKVEKKKQAKCGV
TNEQVRDLIIAYQKLTDAANLVNDSFGLLILIVISGCLVHLLATPYALYAIIFTTGNTMFII
TQSIWMTGHVLRLLLIVEPCHGCILVAKTTTQLVCKLLCLDLDKEVKKSLEFFMTYLAECQI
RFTAYGFTKLHRGLLTTITGAVTTYLVILFQFN
>TcasOr56(AM689916)
MTATKSLKEIPPIYLRVHLTVLQILGIDILPVESVPQNLFYTYTALIISTMCLFTIAEFLDM
VLNYEDIYRLTFGLCYCVTHVLGTVKMFLMLYLRKKLWGNLTTLEEGIFKPNPTRGGPEELQ
IVNDAITMCNRQGYVFYTLVFLIIGARLLYASLANWPYDKHNYFDGNVTVIVNTKEMPYTTW
MPFDYNDSPLYETIFAFQIFSTTVYGFYIGAADAVICGFMMLIKAQFLIVKRELETLIERAQ
KAAIAENPDNEDNFGREIERIELLDKRTQDYVAKYANECVYHHQELIALCDHAEEDFCYLML
LQFISSLLIVCFQLFQVSTLSPDSVEFFSMVCYLLLMLFQLLCYCWHGNEVQIVSGELSRYA
FGINWIIMRESPKKTLLLLMMRAQRPCYFTAGKFSLLSLQTFMTIVRGAGSYFMFLRQMNI
>TcasOr15(AM689917)
MSKNLKEIPPVYLKVHLTVLQILGIDILPNERIPQTLFYTYSVLLIATMVVFTTAECLDLVL
NYEDIYKLTFGLCCCVTHVLGAAKMFLMLYLRKKLWGYFTTLENGIFKPNPCRGGAEEFEIV
TSAINMCKRQGYVFYVLTVGVTGGQGLYAALANLPYDKHNYFDGNVTVVVNTKQMPYATWTP
FDYNDSPLYEIMFAFQIFSTTLYGFYIGAADAVICGFLMLIKAQFLIVKRELETLVERAQRA
GNPDRGDFGGGINRIEMLDDGTQVFVEKCANECVYHHQELIALCEHAEEDFCYLMLLQFISS
LLIVCFQLFQLSTVVRGAGSYFMFLKQMNT
>TcasOr16(AM689918)
MMKFKVTGLVADLMPNIRLIQASGHFMLNYHADNSGALHTLRLGYCCMHLVFVLVQYGCNFV
NLVLERGDVNDLAANTITVLFFTHCVTKFVYFAVRSKLFYRTLGIWNQPNSHPLFVESNNRY
HGIALKKMRRLLYIIIIWTSFSAIAWTGITFVGDSVHNIKDPENENLTITEPIPRLLVKAWY
PWDAMSGMPYYITLVFQIYYVFFSLAHANLLDSLFCSWLIFACEQLQHLKEIMKPLMELSAT
LDTYVPKSADLFRAPSATSQDQLIENDYNEKNEDLKGVYSTRQELGGHFRGGALQNFGSGGV
GPNGLTKKQELMVRSAIKYWVERHKHVVRLVTAIGDAYGVALLLHMLTSTIMLTLLAYQATK
ITGVDKYAATVLGYLLFALAQVFHFCIFGNRLIEESSSVMEAAYSCHWYDGSEEAKTFVQIV
CQQCQKAMSISGAKFFTISLDLFASVLGAVVTYFMVLVQLK

>TcasOr17(AM689919)
MSNFSWKAAVETNITTLKILGLWPKGDETYKLNFYTLYAVFGVIGLLCAHSFVQIFNIYFIV
DDLEAFTSSIFVTLSCLGTVAKTYYLLQNMQMLKELFISINKDIFQPKNNKQILLVEPSIKF
WQRFYLIFRVLCYCTTFFWSSYPILDKWTKDHRLPFLAWYPYDSTKSPFYELTYIHQVVSIW
YLVSASLNIDMLIAALNMFVGAQCDLLCDNLRNIGQNSKEIGKNLVKCIEHHREILR
>TcasOr18(AM689920)
MAKLEYLTGATFTLKCAVLYPIDSNNPKIKKILYAVWAIFFILTFVTGFIQCFVFVCINPFD
LVQEAMIIMSLVFYKNWQNMVALVTNINKNFHRATDNVIEKISMDQASELSDKLAYVWTSSL
AVGSVVPVVLAIATGNLEMPMPAWFPYDYNKSPVFEITYLWQVFCLITLAIIYGASDMFFPC
ITIIIGQQFKILASNFKNNFYTSLIKLGAEESIVQNFSKDIKTHEFRSFYIKYGNIFKILNN
AKFQTLNRAFLKRNIKHHKLLLRFCEDLNKILNTFLLIRVSAIVFNLIFIGFNIIISTDRTL
MLGFCNYFCFGSTELFIHTYSGQILTENADFLWTLYECPWYLCDVTYQKMLILVQMRVKRMV
STKAGNFFTMIAPSFIAFQRAVFSYITLLKEVTDLGKD
>TcasOr19(AM689921)
MVLLPTDFGRFRHHPHIRSAGFDAPLEAMEEGMDISEDLGIFAAIIGLVTMMVLYVVKQKDL
SSLLQDLSNFEKFGKPPNFDKVNKRLDFLVKFVFSYAIFGAVVYNLMRIIEIPHCKRNRKIR
EVCGVFVPLWTPFDADYPPVLVLVASVVFIVVILIDKVTLLVSLQVLEISCHIRLRIEQLNA
MILNCFNGDLQSSRKRLNDCVQYHIDIISYSERFNNCFTNGMFIHLATTGIIIGCLENQNVE
GATPGGVLHLFGWIISLFTSCLAGQILLDSSTSVADALYNSQWYTADVKLRKNLILMIQRSQ
KPLFLATGAFNIMSFALFVTV
>TcasOr20(AM689922)
MMDETYLQFFVKSFTYLNMLPEKTTFCTTIQQYYVSVIITITTFPILADLVSQFYEESISFT
SVNENFVALSALFAVIYVSVCFINRKHKIRALIADLALFETFSSKAVITETDKSVKFYTKLF
IVYGIVGNLCYGLLPILGYKKCHESKSVHMTRYGIPCGLVVRFLFPFKFDYSPLAELVALYE
ILVCILGTSVVIVVTTLICGVLIHITVQLQCLRKIILDLSQVNDLEILEHKMKFCVKYHTAI
LDYGIRTDLAFNQMMLLHITWTGFIISVLGFEISTTDDYVEAFRFFMHLLGWLGMLFVVCYY
GQKILDESLAIADAVYTFLWYKKSVIVQRYVLLILLRSQKPLTLRACGVKVMSLATFLGVLY
SAYSYFTLLLKLKP
>TcasOr21(AM689923)
MEKYDWMQAIKTNILILKIVGLWPDSEDYKFDFYALHASVWLSTLLVASTFFQGINIIFILD
DVKALTGTAYVLLTEILAVIKTYFVVKNMKMLKHLMQSLNNNKLFQPRSHEQIKLIQPSLKF
WKLLYNLFHSLVGGATLFWILFPIVDKKEKRLPFLGWYIVDTKVSPYYEIVYGFQFCSCCYM
SALIINIDTLIAALNVYIGNQIDILCNNLRNLKAGCSIERDLITCIKHHQEILK
>TcasOr22(AM689924)
MAKDSSPVLQESIEIMKYLQLWPQNEHTNLRRRFCFVILLCSPLTLGLAAHLIVCIKDNLDV
ELSANIAVLSAVTGLTYMLIVFVWSQDKVVHLLAKLGTHEIFGTPDNFKKRSRRLDFYAKLY
SYYCYFGIVIYSLLQIIEMPECRKINEEKGLSEICGMIVPFWAPFDIDWFPLKQIFWFDQLL
GIYIIIKGGAAVSITTFEVAQYICLKIKHLNSLLRQAFDDPHEEVIEKRLLHCIRYQQHIIR
TNELFNVCYKHCNGCYVVMVGIIIASLLNQIIKVTAPGLKKASELLSILQAGFAAFSSAVTR
VISESLTIPDAALDSKWYEAPIKSQKMLLLLLVRSQKAFNLHATPVGIMSFALFVALLKTSY
SYFTLLHQST
>TcasOr23(AM689925)
MAKDTSPVLRESIEVMKYLQLWPQNERTNLRRRYFIVIFLCSPLHLGLATHLVVCLKDNLDV
DLSANIAVLSAVTGLTYMLIVFVWSQDKLVHLLAKLDTHEIFGTPDNLTKRSRRLNFYAKLY
SYYCYFGIVIYSLVQIIEMPQCRKMNEEKGLSEICGMIVPFWAPFDIDWFPLKQIFWLNQLL
GIYIIIKGGAAVSITTFEVAQYICLKIKHLNRLLREAFDDPCDVVVEQKLLHCIRYQQHIIR
TNELFNVCFKHCNGCYVVMVGIIIASLLNQILKEKSVGALVHFAGWICSFFICCHAGQAVIS
ESLTIPEAALDSHWYEAPVKYKKVLLLLLVRSQKAFNLQATPIGIMSFDL
>TcasOr24(AM689926)
MTEEKQLRICLSSCFFLKWSFMWPTKSEEFRTSKGLYFRLLAFVIISGLTFTAMIVMHLLKS
VEAGDYDISEDIAILATNTGYILMMLLYIIRQKDLESLLVDLSSFKKYQKPPKFDEVNRKLE
WCTRMVFGYCVFGSVFYNLVKILAIPSCKKSRRINEVCGVAIPYWVWFDTENWSIKLPLILH
TFLVIIIVDKVTLLVSLQVLEIACNIKLRLDQLNCMLVSCFDGDVEASRRRLNECIKYHKEI

ISYSEIFSKCFSIEMFTHLTTTGIICGCLENQVVQEHRPEAILHIGGWITAIFVSSFGGQIL
IDSSLSVAEAAYSSAWYEADIMKMSYSILTILQ
>TcasOr25(AM689927)
MTEEKELRLCLWSCYYLKLSLMWPLKREEFKSSKGLYLRLLVFVIISGSTFTAMIFMHLYKS
LKVGSYDVSEDLAILASNIGYVLMMTMYVSRQKDLELLLLDLSDFKTYGKPPNFDKVRKRMD
LYAHLIFFYSMFGSFVYNMDKIILIDKCKEARRINEVCGSAIPFWTPFETEDLFTLTLVITY
VLINIFVVVKVAMTVSVQVLEISSHINLRIEQLKIFIAGCFDRDFKASRERLDFCIRYHNVI
IDFSERFSRCFSYVMFIHLAITGIIIGCLENQIVQEHQPEAMLHMGGWSTATFIACYGGQLL
MDASTSIADEFYNCPWYEADVKMRKDLILIILRAQKALFVSTGPFNVLSFALFVSLKTSFIW
PPKNEEMTPSRWFYIKLAVLLTVSVIILYVPVCIHIHKLFERDLDASEDFALMFSYYGFLST
MIFYSKNHKKICGLIYNLCSFEEFGKPPQFDELNQRLDFYSKLASGYTIIGMAVYIGMKIYK
IPDCKAERGAIQVCGLTVPLLCEQFNEAYYWTAFSHIVCSVLACACVAHQAVNQDVLAVLHY
IGWIMSIFVVCWAGQILTDSSVTVADACFGSNWLRACPRLRRDLSFMMRRSQKCVELRAGPF
NVLCFSLFVSPIITKHICERQNAARHFDDICGLVTNSWMPFDIKKFPNKQLFYLWQTYSVYW
TYEGSAAISYLIVATMEHVLMRIWHLQGMIQEAINTEDAQVRKSRLDKCLEYHNHVFLLAAD
VDRIYQRSLFVHVLFSGVLFGIMGFSILTVGISVKTLTCRRKGFMMG
>TcasOr26(AM689928)
MMESTVTRLKRMYLWPTASVTSRKPAFFLITFSCFLLYGSVMHLIVNDISMEEVHVIETTAG
QFGVLYYLTLFTIYRKGILEIYADLSNFTKFGKPYNFDKRNKQLNQWSRWFSVVLYFFVISV
FAWPGIFTQSCEDLNVALNKTEVCGVVSPVWLPFRFDYKPMKQFVYFWQSFCCLYSNGGAGT
ISFAMSETIEHLILRVEDLKILFPKIVAERSPEVRRKMLAKWVDYHLWLLSIGKLMNDTYRY
SFSVIVLCAGTLFGCIGYTVMKFHSVGTTVYNSEWCDTDVDFQKGVILITIRAQKPVRIYAG
PFSYVSHLLILTVFQTSYSYINLLNASS

B

```
ID   TcasGr1(AM292322)
FT   DOMAIN        1     41       NON CYTOPLASMIC.
FT   TRANSMEM     42     61
FT   DOMAIN       62     70       CYTOPLASMIC.
FT   TRANSMEM     71     93
FT   DOMAIN       94    134       NON CYTOPLASMIC.
FT   TRANSMEM    135    154
FT   DOMAIN      155    170       CYTOPLASMIC.
FT   TRANSMEM    171    191
FT   DOMAIN      192    207       NON CYTOPLASMIC.
FT   TRANSMEM    208    229
FT   DOMAIN      230    241       CYTOPLASMIC.
FT   TRANSMEM    242    266
FT   DOMAIN      267    272       NON CYTOPLASMIC.
FT   TRANSMEM    273    296
FT   DOMAIN      297    373       CYTOPLASMIC.
//
ID   TcasGr2(AM292323)
FT   DOMAIN        1    187       NON CYTOPLASMIC.
FT   TRANSMEM    188    209
FT   DOMAIN      210    222       CYTOPLASMIC.
FT   TRANSMEM    223    246
FT   DOMAIN      247    284       NON CYTOPLASMIC.
FT   TRANSMEM    285    305
FT   DOMAIN      306    317       CYTOPLASMIC.
FT   TRANSMEM    318    339
FT   DOMAIN      340    390       NON CYTOPLASMIC.
FT   TRANSMEM    391    412
FT   DOMAIN      413    440       CYTOPLASMIC.
FT   TRANSMEM    441    463
FT   DOMAIN      464    521       NON CYTOPLASMIC.
FT   TRANSMEM    522    545
FT   DOMAIN      546    586       CYTOPLASMIC.
//
ID   TcasGr3(AM292359)
FT   DOMAIN        1     58       NON CYTOPLASMIC.
FT   TRANSMEM     59     79
FT   DOMAIN       80     91       CYTOPLASMIC.
FT   TRANSMEM     92    111
FT   DOMAIN      112    133       NON CYTOPLASMIC.
FT   TRANSMEM    134    155
FT   DOMAIN      156    176       CYTOPLASMIC.
FT   TRANSMEM    177    196
FT   DOMAIN      197    211       NON CYTOPLASMIC.
FT   TRANSMEM    212    234
FT   DOMAIN      235    286       CYTOPLASMIC.
FT   TRANSMEM    287    308
FT   DOMAIN      309    313       NON CYTOPLASMIC.
FT   TRANSMEM    314    335
FT   DOMAIN      336    436       CYTOPLASMIC.
//
```

```
ID   TcasGr4(AM292325)
FT   DOMAIN         1     32        NON CYTOPLASMIC.
FT   TRANSMEM      33     51
FT   DOMAIN        52     64        CYTOPLASMIC.
FT   TRANSMEM      65     88
FT   DOMAIN        89    119        NON CYTOPLASMIC.
FT   TRANSMEM     120    142
FT   DOMAIN       143    155        CYTOPLASMIC.
FT   TRANSMEM     156    176
FT   DOMAIN       177    192        NON CYTOPLASMIC.
FT   TRANSMEM     193    213
FT   DOMAIN       214    226        CYTOPLASMIC.
FT   TRANSMEM     227    248
FT   DOMAIN       249    258        NON CYTOPLASMIC.
FT   TRANSMEM     259    281
FT   DOMAIN       282    309        CYTOPLASMIC.
//
ID   TcasGr5(AM292326)
FT   DOMAIN         1    109        NON CYTOPLASMIC.
FT   TRANSMEM     110    131
FT   DOMAIN       132    147        CYTOPLASMIC.
FT   TRANSMEM     148    169
FT   DOMAIN       170    209        NON CYTOPLASMIC.
FT   TRANSMEM     210    230
FT   DOMAIN       231    244        CYTOPLASMIC.
FT   TRANSMEM     245    265
FT   DOMAIN       266    311        NON CYTOPLASMIC.
FT   TRANSMEM     312    334
FT   DOMAIN       335    344        CYTOPLASMIC.
FT   TRANSMEM     345    369
FT   DOMAIN       370    392        NON CYTOPLASMIC.
FT   TRANSMEM     393    413
FT   DOMAIN       414    522        CYTOPLASMIC.
//
ID   TCasGr6(AM292327)
FT   DOMAIN         1    127        NON CYTOPLASMIC.
FT   TRANSMEM     128    151
FT   DOMAIN       152    161        CYTOPLASMIC.
FT   TRANSMEM     162    186
FT   DOMAIN       187    212        NON CYTOPLASMIC.
FT   TRANSMEM     213    232
FT   DOMAIN       233    247        CYTOPLASMIC.
FT   TRANSMEM     248    267
FT   DOMAIN       268    282        NON CYTOPLASMIC.
FT   TRANSMEM     283    304
FT   DOMAIN       305    336        CYTOPLASMIC.
FT   TRANSMEM     337    356
FT   DOMAIN       357    358        NON CYTOPLASMIC.
FT   TRANSMEM     359    378
FT   DOMAIN       379    387        CYTOPLASMIC.
//
ID   TcasGr7(AM292328)
FT   DOMAIN         1     73        NON CYTOPLASMIC.
```

FT TRANSMEM 74 95
FT DOMAIN 96 105 CYTOPLASMIC.
FT TRANSMEM 106 125
FT DOMAIN 126 150 NON CYTOPLASMIC.
FT TRANSMEM 151 172
FT DOMAIN 173 191 CYTOPLASMIC.
FT TRANSMEM 192 212
FT DOMAIN 213 225 NON CYTOPLASMIC.
FT TRANSMEM 226 246
FT DOMAIN 247 288 CYTOPLASMIC.
FT TRANSMEM 289 310
FT DOMAIN 311 319 NON CYTOPLASMIC.
FT TRANSMEM 320 340
FT DOMAIN 341 429 CYTOPLASMIC.
//
ID TcasGr104(AM292329)
FT DOMAIN 1 44 NON CYTOPLASMIC.
FT TRANSMEM 45 64
FT DOMAIN 65 74 CYTOPLASMIC.
FT TRANSMEM 75 97
FT DOMAIN 98 138 NON CYTOPLASMIC.
FT TRANSMEM 139 160
FT DOMAIN 161 173 CYTOPLASMIC.
FT TRANSMEM 174 193
FT DOMAIN 194 231 NON CYTOPLASMIC.
FT TRANSMEM 232 258
FT DOMAIN 259 280 CYTOPLASMIC.
FT TRANSMEM 281 304
FT DOMAIN 305 348 NON CYTOPLASMIC.
FT TRANSMEM 349 370
FT DOMAIN 371 379 CYTOPLASMIC.
//
ID TcasGr9(AM292330)
FT DOMAIN 1 54 NON CYTOPLASMIC.
FT TRANSMEM 55 77
FT DOMAIN 78 88 CYTOPLASMIC.
FT TRANSMEM 89 110
FT DOMAIN 111 138 NON CYTOPLASMIC.
FT TRANSMEM 139 159
FT DOMAIN 160 173 CYTOPLASMIC.
FT TRANSMEM 174 193
FT DOMAIN 194 223 NON CYTOPLASMIC.
FT TRANSMEM 224 244
FT DOMAIN 245 286 CYTOPLASMIC.
FT TRANSMEM 287 310
FT DOMAIN 311 318 NON CYTOPLASMIC.
FT TRANSMEM 319 339
FT DOMAIN 340 408 CYTOPLASMIC.
//
ID TcasGr10(AM292331)
FT DOMAIN 1 85 NON CYTOPLASMIC.
FT TRANSMEM 86 110
FT DOMAIN 111 120 CYTOPLASMIC.

FT	TRANSMEM	121	141	
FT	DOMAIN	142	167	NON CYTOPLASMIC.
FT	TRANSMEM	168	190	
FT	DOMAIN	191	208	CYTOPLASMIC.
FT	TRANSMEM	209	228	
FT	DOMAIN	229	269	NON CYTOPLASMIC.
FT	TRANSMEM	270	294	
FT	DOMAIN	295	306	CYTOPLASMIC.
FT	TRANSMEM	307	329	
FT	DOMAIN	330	385	NON CYTOPLASMIC.
FT	TRANSMEM	386	406	
FT	DOMAIN	407	437	CYTOPLASMIC.
//				
**ID**	**TcasGr11(AM292332)**			
FT	DOMAIN	1	56	NON CYTOPLASMIC.
FT	TRANSMEM	57	77	
FT	DOMAIN	78	89	CYTOPLASMIC.
FT	TRANSMEM	90	112	
FT	DOMAIN	113	140	NON CYTOPLASMIC.
FT	TRANSMEM	141	161	
FT	DOMAIN	162	175	CYTOPLASMIC.
FT	TRANSMEM	176	197	
FT	DOMAIN	198	240	NON CYTOPLASMIC.
FT	TRANSMEM	241	264	
FT	DOMAIN	265	276	CYTOPLASMIC.
FT	TRANSMEM	277	297	
FT	DOMAIN	298	302	NON CYTOPLASMIC.
FT	TRANSMEM	303	324	
FT	DOMAIN	325	344	CYTOPLASMIC.
//				
**ID**	**TcasGr12(AM292333)**			
FT	DOMAIN	1	28	NON CYTOPLASMIC.
FT	TRANSMEM	29	48	
FT	DOMAIN	49	60	CYTOPLASMIC.
FT	TRANSMEM	61	83	
FT	DOMAIN	84	104	NON CYTOPLASMIC.
FT	TRANSMEM	105	125	
FT	DOMAIN	126	142	CYTOPLASMIC.
FT	TRANSMEM	143	163	
FT	DOMAIN	164	209	NON CYTOPLASMIC.
FT	TRANSMEM	210	234	
FT	DOMAIN	235	266	CYTOPLASMIC.
FT	TRANSMEM	267	287	
FT	DOMAIN	288	290	NON CYTOPLASMIC.
FT	TRANSMEM	291	309	
FT	DOMAIN	310	316	CYTOPLASMIC.
//				
**ID**	**TcasGr13(AM292334)**			
FT	DOMAIN	1	102	NON CYTOPLASMIC.
FT	TRANSMEM	103	122	
FT	DOMAIN	123	134	CYTOPLASMIC.
FT	TRANSMEM	135	157	
FT	DOMAIN	158	178	NON CYTOPLASMIC.

```
FT   TRANSMEM    179    199
FT   DOMAIN      200    216       CYTOPLASMIC.
FT   TRANSMEM    217    237
FT   DOMAIN      238    283       NON CYTOPLASMIC.
FT   TRANSMEM    284    308
FT   DOMAIN      309    340       CYTOPLASMIC.
FT   TRANSMEM    341    361
FT   DOMAIN      362    364       NON CYTOPLASMIC.
FT   TRANSMEM    365    383
FT   DOMAIN      384    390       CYTOPLASMIC.
//
ID   TcasGr14(AM292335)
FT   SIGNAL        1     27
FT   DOMAIN        1     10       N-REGION.
FT   DOMAIN       11     21       H-REGION.
FT   DOMAIN       22     27       C-REGION.
FT   DOMAIN       28    114       NON CYTOPLASMIC.
FT   TRANSMEM    115    135
FT   DOMAIN      136    148       CYTOPLASMIC.
FT   TRANSMEM    149    173
FT   DOMAIN      174    185       NON CYTOPLASMIC.
FT   TRANSMEM    186    206
FT   DOMAIN      207    225       CYTOPLASMIC.
FT   TRANSMEM    226    246
FT   DOMAIN      247    270       NON CYTOPLASMIC.
FT   TRANSMEM    271    294
FT   DOMAIN      295    323       CYTOPLASMIC.
FT   TRANSMEM    324    344
FT   DOMAIN      345    347       NON CYTOPLASMIC.
FT   TRANSMEM    348    369
FT   DOMAIN      370    374       CYTOPLASMIC.
//
ID   TcasGr15(AM292336)
FT   DOMAIN        1    117       NON CYTOPLASMIC.
FT   TRANSMEM    118    138
FT   DOMAIN      139    149       CYTOPLASMIC.
FT   TRANSMEM    150    175
FT   DOMAIN      176    242       NON CYTOPLASMIC.
FT   TRANSMEM    243    262
FT   DOMAIN      263    282       CYTOPLASMIC.
FT   TRANSMEM    283    302
FT   DOMAIN      303    358       NON CYTOPLASMIC.
FT   TRANSMEM    359    380
FT   DOMAIN      381    390       CYTOPLASMIC.
FT   TRANSMEM    391    412
FT   DOMAIN      413    420       NON CYTOPLASMIC.
FT   TRANSMEM    421    442
FT   DOMAIN      443    455       CYTOPLASMIC.
//
ID   TcasGr16(AM292337)
FT   DOMAIN        1     36       NON CYTOPLASMIC.
FT   TRANSMEM     37     57
FT   DOMAIN       58     72       CYTOPLASMIC.
```

```
FT   TRANSMEM      73     98
FT   DOMAIN        99    132       NON CYTOPLASMIC.
FT   TRANSMEM     133    154
FT   DOMAIN       155    175       CYTOPLASMIC.
FT   TRANSMEM     176    196
FT   DOMAIN       197    254       NON CYTOPLASMIC.
FT   TRANSMEM     255    279
FT   DOMAIN       280    288       CYTOPLASMIC.
FT   TRANSMEM     289    310
FT   DOMAIN       311    350       NON CYTOPLASMIC.
FT   TRANSMEM     351    371
FT   DOMAIN       372    452       CYTOPLASMIC.
//
ID   TcasGr17(AM292338)
FT   DOMAIN         1     32       NON CYTOPLASMIC.
FT   TRANSMEM      33     54
FT   DOMAIN        55     67       CYTOPLASMIC.
FT   TRANSMEM      68     89
FT   DOMAIN        90    125       NON CYTOPLASMIC.
FT   TRANSMEM     126    147
FT   DOMAIN       148    162       CYTOPLASMIC.
FT   TRANSMEM     163    184
FT   DOMAIN       185    236       NON CYTOPLASMIC.
FT   TRANSMEM     237    261
FT   DOMAIN       262    274       CYTOPLASMIC.
FT   TRANSMEM     275    295
FT   DOMAIN       296    333       NON CYTOPLASMIC.
FT   TRANSMEM     334    353
FT   DOMAIN       354    372       CYTOPLASMIC.
//
ID   TcasGr150(AM292339)
FT   DOMAIN         1     32       NON CYTOPLASMIC.
FT   TRANSMEM      33     54
FT   DOMAIN        55     67       CYTOPLASMIC.
FT   TRANSMEM      68     89
FT   DOMAIN        90    125       NON CYTOPLASMIC.
FT   TRANSMEM     126    147
FT   DOMAIN       148    162       CYTOPLASMIC.
FT   TRANSMEM     163    184
FT   DOMAIN       185    236       NON CYTOPLASMIC.
FT   TRANSMEM     237    261
FT   DOMAIN       262    274       CYTOPLASMIC.
FT   TRANSMEM     275    295
FT   DOMAIN       296    345       NON CYTOPLASMIC.
FT   TRANSMEM     346    368
FT   DOMAIN       369    387       CYTOPLASMIC.
//
ID   TcasGr19(AM292340)
FT   DOMAIN         1     55       NON CYTOPLASMIC.
FT   TRANSMEM      56     79
FT   DOMAIN        80     88       CYTOPLASMIC.
FT   TRANSMEM      89    111
FT   DOMAIN       112    124       NON CYTOPLASMIC.
```

FT	TRANSMEM	125	150	
FT	DOMAIN	151	168	CYTOPLASMIC.
FT	TRANSMEM	169	189	
FT	DOMAIN	190	219	NON CYTOPLASMIC.
FT	TRANSMEM	220	242	
FT	DOMAIN	243	251	CYTOPLASMIC.
FT	TRANSMEM	252	275	
FT	DOMAIN	276	278	NON CYTOPLASMIC.
FT	TRANSMEM	279	301	
FT	DOMAIN	302	355	CYTOPLASMIC.
//				
**ID**	**TcasGr20(AM292341)**			
FT	DOMAIN	1	51	NON CYTOPLASMIC.
FT	TRANSMEM	52	73	
FT	DOMAIN	74	83	CYTOPLASMIC.
FT	TRANSMEM	84	109	
FT	DOMAIN	110	136	NON CYTOPLASMIC.
FT	TRANSMEM	137	159	
FT	DOMAIN	160	175	CYTOPLASMIC.
FT	TRANSMEM	176	197	
FT	DOMAIN	198	265	NON CYTOPLASMIC.
FT	TRANSMEM	266	291	
FT	DOMAIN	292	312	CYTOPLASMIC.
FT	TRANSMEM	313	334	
FT	DOMAIN	335	360	NON CYTOPLASMIC.
FT	TRANSMEM	361	383	
FT	DOMAIN	384	393	CYTOPLASMIC.
//				
**ID**	**TcasGr21(AM292342)**			
FT	DOMAIN	1	48	NON CYTOPLASMIC.
FT	TRANSMEM	49	74	
FT	DOMAIN	75	89	CYTOPLASMIC.
FT	TRANSMEM	90	115	
FT	DOMAIN	116	143	NON CYTOPLASMIC.
FT	TRANSMEM	144	164	
FT	DOMAIN	165	176	CYTOPLASMIC.
FT	TRANSMEM	177	196	
FT	DOMAIN	197	250	NON CYTOPLASMIC.
FT	TRANSMEM	251	272	
FT	DOMAIN	273	286	CYTOPLASMIC.
FT	TRANSMEM	287	308	
FT	DOMAIN	309	325	NON CYTOPLASMIC.
FT	TRANSMEM	326	345	
FT	DOMAIN	346	386	CYTOPLASMIC.
//				
**ID**	**TcasGr22(AM292343)**			
FT	DOMAIN	1	51	NON CYTOPLASMIC.
FT	TRANSMEM	52	71	
FT	DOMAIN	72	80	CYTOPLASMIC.
FT	TRANSMEM	81	98	
FT	DOMAIN	99	110	NON CYTOPLASMIC.
FT	TRANSMEM	111	129	
FT	DOMAIN	130	149	CYTOPLASMIC.

```
FT   TRANSMEM    150    170
FT   DOMAIN      171    184       NON CYTOPLASMIC.
FT   TRANSMEM    185    209
FT   DOMAIN      210    246       CYTOPLASMIC.
FT   TRANSMEM    247    265
FT   DOMAIN      266    267       NON CYTOPLASMIC.
FT   TRANSMEM    268    287
FT   DOMAIN      288    301       CYTOPLASMIC.
//
ID   TcasGr79(AM292344)
FT   DOMAIN        1     54       NON CYTOPLASMIC.
FT   TRANSMEM     55     75
FT   DOMAIN       76     85       CYTOPLASMIC.
FT   TRANSMEM     86    108
FT   DOMAIN      109    122       NON CYTOPLASMIC.
FT   TRANSMEM    123    142
FT   DOMAIN      143    153       CYTOPLASMIC.
FT   TRANSMEM    154    173
FT   DOMAIN      174    192       NON CYTOPLASMIC.
FT   TRANSMEM    193    216
FT   DOMAIN      217    243       CYTOPLASMIC.
FT   TRANSMEM    244    263
FT   DOMAIN      264    266       NON CYTOPLASMIC.
FT   TRANSMEM    267    286
FT   DOMAIN      287    291       CYTOPLASMIC.
//
ID   TcasGr123(AM292345)
FT   DOMAIN        1     72       NON CYTOPLASMIC.
FT   TRANSMEM     73     97
FT   DOMAIN       98    105       CYTOPLASMIC.
FT   TRANSMEM    106    124
FT   DOMAIN      125    142       NON CYTOPLASMIC.
FT   TRANSMEM    143    164
FT   DOMAIN      165    176       CYTOPLASMIC.
FT   TRANSMEM    177    196
FT   DOMAIN      197    243       NON CYTOPLASMIC.
FT   TRANSMEM    244    270
FT   DOMAIN      271    280       CYTOPLASMIC.
FT   TRANSMEM    281    301
FT   DOMAIN      302    351       NON CYTOPLASMIC.
FT   TRANSMEM    352    374
FT   DOMAIN      375    384       CYTOPLASMIC.
//
ID   TcasGr25(AM292346)
FT   SIGNAL        1     18
FT   DOMAIN        1      5       N-REGION.
FT   DOMAIN        6     13       H-REGION.
FT   DOMAIN       14     18       C-REGION.
FT   DOMAIN       19     44       NON CYTOPLASMIC.
FT   TRANSMEM     45     63
FT   DOMAIN       64     70       CYTOPLASMIC.
FT   TRANSMEM     71     90
FT   DOMAIN       91    100       NON CYTOPLASMIC.
```

```
FT   TRANSMEM    101    120
FT   DOMAIN      121    137       CYTOPLASMIC.
FT   TRANSMEM    138    157
FT   DOMAIN      158    173       NON CYTOPLASMIC.
FT   TRANSMEM    174    197
FT   DOMAIN      198    211       CYTOPLASMIC.
FT   TRANSMEM    212    230
FT   DOMAIN      231    233       NON CYTOPLASMIC.
FT   TRANSMEM    234    252
FT   DOMAIN      253    314       CYTOPLASMIC.
//
ID   TcasGr26(AM292347)
FT   DOMAIN        1    127       NON CYTOPLASMIC.
FT   TRANSMEM    128    151
FT   DOMAIN      152    161       CYTOPLASMIC.
FT   TRANSMEM    162    186
FT   DOMAIN      187    212       NON CYTOPLASMIC.
FT   TRANSMEM    213    232
FT   DOMAIN      233    247       CYTOPLASMIC.
FT   TRANSMEM    248    267
FT   DOMAIN      268    282       NON CYTOPLASMIC.
FT   TRANSMEM    283    304
FT   DOMAIN      305    336       CYTOPLASMIC.
FT   TRANSMEM    337    356
FT   DOMAIN      357    358       NON CYTOPLASMIC.
FT   TRANSMEM    359    378
FT   DOMAIN      379    387       CYTOPLASMIC.
//
ID   TcasGr27(AM292348)
FT   DOMAIN        1      6       NON CYTOPLASMIC.
FT   TRANSMEM      7     25
FT   DOMAIN       26     34       CYTOPLASMIC.
FT   TRANSMEM     35     56
FT   DOMAIN       57     77       NON CYTOPLASMIC.
FT   TRANSMEM     78     99
FT   DOMAIN      100    120       CYTOPLASMIC.
FT   TRANSMEM    121    142
FT   DOMAIN      143    168       NON CYTOPLASMIC.
FT   TRANSMEM    169    194
FT   DOMAIN      195    250       CYTOPLASMIC.
FT   TRANSMEM    251    273
FT   DOMAIN      274    296       NON CYTOPLASMIC.
FT   TRANSMEM    297    318
FT   DOMAIN      319    346       CYTOPLASMIC.
//
ID   TcasGr28(AM292349)
FT   DOMAIN        1     28       NON CYTOPLASMIC.
FT   TRANSMEM     29     49
FT   DOMAIN       50     60       CYTOPLASMIC.
FT   TRANSMEM     61     81
FT   DOMAIN       82     92       NON CYTOPLASMIC.
FT   TRANSMEM     93    112
FT   DOMAIN      113    123       CYTOPLASMIC.
```

```
FT   TRANSMEM    124    143
FT   DOMAIN      144    165       NON CYTOPLASMIC.
FT   TRANSMEM    166    186
FT   DOMAIN      187    196       CYTOPLASMIC.
FT   TRANSMEM    197    217
FT   DOMAIN      218    220       NON CYTOPLASMIC.
FT   TRANSMEM    221    239
FT   DOMAIN      240    248       CYTOPLASMIC.
//
ID   TcasGr29(AM292350)
FT   DOMAIN        1     73       NON CYTOPLASMIC.
FT   TRANSMEM     74     95
FT   DOMAIN       96    105       CYTOPLASMIC.
FT   TRANSMEM    106    125
FT   DOMAIN      126    150       NON CYTOPLASMIC.
FT   TRANSMEM    151    172
FT   DOMAIN      173    191       CYTOPLASMIC.
FT   TRANSMEM    192    212
FT   DOMAIN      213    225       NON CYTOPLASMIC.
FT   TRANSMEM    226    246
FT   DOMAIN      247    288       CYTOPLASMIC.
FT   TRANSMEM    289    310
FT   DOMAIN      311    319       NON CYTOPLASMIC.
FT   TRANSMEM    320    340
FT   DOMAIN      341    429       CYTOPLASMIC.
//
ID   TcasGr30(AM292351)
FT   DOMAIN        1     73       NON CYTOPLASMIC.
FT   TRANSMEM     74     95
FT   DOMAIN       96    105       CYTOPLASMIC.
FT   TRANSMEM    106    125
FT   DOMAIN      126    150       NON CYTOPLASMIC.
FT   TRANSMEM    151    172
FT   DOMAIN      173    191       CYTOPLASMIC.
FT   TRANSMEM    192    212
FT   DOMAIN      213    225       NON CYTOPLASMIC.
FT   TRANSMEM    226    246
FT   DOMAIN      247    288       CYTOPLASMIC.
FT   TRANSMEM    289    310
FT   DOMAIN      311    319       NON CYTOPLASMIC.
FT   TRANSMEM    320    341
FT   DOMAIN      342    394       CYTOPLASMIC.
//
ID   TcasGr31(AM292352)
FT   DOMAIN        1     13       NON CYTOPLASMIC.
FT   TRANSMEM     14     32
FT   DOMAIN       33     37       CYTOPLASMIC.
FT   TRANSMEM     38     56
FT   DOMAIN       57     66       NON CYTOPLASMIC.
FT   TRANSMEM     67     86
FT   DOMAIN       87     99       CYTOPLASMIC.
FT   TRANSMEM    100    120
FT   DOMAIN      121    153       NON CYTOPLASMIC.
```

```
FT   TRANSMEM    154    179
FT   DOMAIN      180    190       CYTOPLASMIC.
FT   TRANSMEM    191    211
FT   DOMAIN      212    224       NON CYTOPLASMIC.
FT   TRANSMEM    225    247
FT   DOMAIN      248    250       CYTOPLASMIC.
//
ID   TcasGr32(AM292353)
FT   DOMAIN        1    151       NON CYTOPLASMIC.
FT   TRANSMEM    152    174
FT   DOMAIN      175    188       CYTOPLASMIC.
FT   TRANSMEM    189    209
FT   DOMAIN      210    223       NON CYTOPLASMIC.
FT   TRANSMEM    224    243
FT   DOMAIN      244    257       CYTOPLASMIC.
FT   TRANSMEM    258    278
FT   DOMAIN      279    323       NON CYTOPLASMIC.
FT   TRANSMEM    324    347
FT   DOMAIN      348    375       CYTOPLASMIC.
FT   TRANSMEM    376    398
FT   DOMAIN      399    422       NON CYTOPLASMIC.
FT   TRANSMEM    423    445
FT   DOMAIN      446    651       CYTOPLASMIC.
//
ID   TcasGr33(AM292368)
FT   DOMAIN        1    127       NON CYTOPLASMIC.
FT   TRANSMEM    128    151
FT   DOMAIN      152    161       CYTOPLASMIC.
FT   TRANSMEM    162    186
FT   DOMAIN      187    212       NON CYTOPLASMIC.
FT   TRANSMEM    213    232
FT   DOMAIN      233    247       CYTOPLASMIC.
FT   TRANSMEM    248    267
FT   DOMAIN      268    282       NON CYTOPLASMIC.
FT   TRANSMEM    283    304
FT   DOMAIN      305    336       CYTOPLASMIC.
FT   TRANSMEM    337    356
FT   DOMAIN      357    358       NON CYTOPLASMIC.
FT   TRANSMEM    359    378
FT   DOMAIN      379    387       CYTOPLASMIC.
//

ID   TcasGr34(AM292355)
FT   DOMAIN        1     24       NON CYTOPLASMIC.
FT   TRANSMEM     25     43
FT   DOMAIN       44     52       CYTOPLASMIC.
FT   TRANSMEM     53     76
FT   DOMAIN       77     92       NON CYTOPLASMIC.
FT   TRANSMEM     93    113
FT   DOMAIN      114    128       CYTOPLASMIC.
FT   TRANSMEM    129    150
FT   DOMAIN      151    195       NON CYTOPLASMIC.
FT   TRANSMEM    196    218
```

```
FT   DOMAIN      219    235        CYTOPLASMIC.
FT   TRANSMEM    236    256
FT   DOMAIN      257    295        NON CYTOPLASMIC.
FT   TRANSMEM    296    318
FT   DOMAIN      319    324        CYTOPLASMIC.
//
ID   TcasGr35(AM292356)
FT   DOMAIN        1      4        NON CYTOPLASMIC.
FT   TRANSMEM      5     26
FT   DOMAIN       27     39        CYTOPLASMIC.
FT   TRANSMEM     40     62
FT   DOMAIN       63     73        NON CYTOPLASMIC.
FT   TRANSMEM     74     93
FT   DOMAIN       94    114        CYTOPLASMIC.
FT   TRANSMEM    115    135
FT   DOMAIN      136    149        NON CYTOPLASMIC.
FT   TRANSMEM    150    172
FT   DOMAIN      173    203        CYTOPLASMIC.
FT   TRANSMEM    204    224
FT   DOMAIN      225    227        NON CYTOPLASMIC.
FT   TRANSMEM    228    248
FT   DOMAIN      249    251        CYTOPLASMIC.
//
ID   TcasGr36(AM292357)
FT   DOMAIN        1     43        NON CYTOPLASMIC.
FT   TRANSMEM     44     63
FT   DOMAIN       64     70        CYTOPLASMIC.
FT   TRANSMEM     71     93
FT   DOMAIN       94    131        NON CYTOPLASMIC.
FT   TRANSMEM    132    151
FT   DOMAIN      152    166        CYTOPLASMIC.
FT   TRANSMEM    167    186
FT   DOMAIN      187    212        NON CYTOPLASMIC.
FT   TRANSMEM    213    236
FT   DOMAIN      237    253        CYTOPLASMIC.
FT   TRANSMEM    254    276
FT   DOMAIN      277    328        NON CYTOPLASMIC.
FT   TRANSMEM    329    350
FT   DOMAIN      351    355        CYTOPLASMIC.
//
ID   TcasGr37(AM292358)
FT   DOMAIN        1     25        NON CYTOPLASMIC.
FT   TRANSMEM     26     47
FT   DOMAIN       48     57        CYTOPLASMIC.
FT   TRANSMEM     58     80
FT   DOMAIN       81    107        NON CYTOPLASMIC.
FT   TRANSMEM    108    129
FT   DOMAIN      130    143        CYTOPLASMIC.
FT   TRANSMEM    144    164
FT   DOMAIN      165    194        NON CYTOPLASMIC.
FT   TRANSMEM    195    218
FT   DOMAIN      219    236        CYTOPLASMIC.
FT   TRANSMEM    237    260
```

```
FT   DOMAIN      261    283       NON CYTOPLASMIC.
FT   TRANSMEM    284    303
FT   DOMAIN      304    331       CYTOPLASMIC.
//
```
ID   TcasGr38(AM292324)
```
FT   DOMAIN        1     58       NON CYTOPLASMIC.
FT   TRANSMEM     59     81
FT   DOMAIN       82     95       CYTOPLASMIC.
FT   TRANSMEM     96    118
FT   DOMAIN      119    149       NON CYTOPLASMIC.
FT   TRANSMEM    150    170
FT   DOMAIN      171    185       CYTOPLASMIC.
FT   TRANSMEM    186    206
FT   DOMAIN      207    257       NON CYTOPLASMIC.
FT   TRANSMEM    258    282
FT   DOMAIN      283    294       CYTOPLASMIC.
FT   TRANSMEM    295    315
FT   DOMAIN      316    369       NON CYTOPLASMIC.
FT   TRANSMEM    370    390
FT   DOMAIN      391    398       CYTOPLASMIC.
//
```
ID   TcasGr39(AM292360)
```
FT   DOMAIN        1     85       NON CYTOPLASMIC.
FT   TRANSMEM     86    110
FT   DOMAIN      111    120       CYTOPLASMIC.
FT   TRANSMEM    121    141
FT   DOMAIN      142    167       NON CYTOPLASMIC.
FT   TRANSMEM    168    190
FT   DOMAIN      191    208       CYTOPLASMIC.
FT   TRANSMEM    209    228
FT   DOMAIN      229    269       NON CYTOPLASMIC.
FT   TRANSMEM    270    294
FT   DOMAIN      295    306       CYTOPLASMIC.
FT   TRANSMEM    307    329
FT   DOMAIN      330    385       NON CYTOPLASMIC.
FT   TRANSMEM    386    406
FT   DOMAIN      407    426       CYTOPLASMIC.
//
```
ID   TcasGr40(AM292361)
```
FT   DOMAIN        1     61       NON CYTOPLASMIC.
FT   TRANSMEM     62     84
FT   DOMAIN       85     96       CYTOPLASMIC.
FT   TRANSMEM     97    120
FT   DOMAIN      121    148       NON CYTOPLASMIC.
FT   TRANSMEM    149    170
FT   DOMAIN      171    188       CYTOPLASMIC.
FT   TRANSMEM    189    210
FT   DOMAIN      211    258       NON CYTOPLASMIC.
FT   TRANSMEM    259    282
FT   DOMAIN      283    307       CYTOPLASMIC.
FT   TRANSMEM    308    331
FT   DOMAIN      332    349       NON CYTOPLASMIC.
FT   TRANSMEM    350    372
```

```
FT   DOMAIN      373    373        CYTOPLASMIC.
//
ID   TcasGr41(AM292362)
FT   DOMAIN        1     39        NON CYTOPLASMIC.
FT   TRANSMEM     40     61
FT   DOMAIN       62     72        CYTOPLASMIC.
FT   TRANSMEM     73     95
FT   DOMAIN       96    125        NON CYTOPLASMIC.
FT   TRANSMEM    126    148
FT   DOMAIN      149    166        CYTOPLASMIC.
FT   TRANSMEM    167    187
FT   DOMAIN      188    230        NON CYTOPLASMIC.
FT   TRANSMEM    231    255
FT   DOMAIN      256    269        CYTOPLASMIC.
FT   TRANSMEM    270    293
FT   DOMAIN      294    314        NON CYTOPLASMIC.
FT   TRANSMEM    315    335
FT   DOMAIN      336    398        CYTOPLASMIC.
//
ID   TcasGr71(AM292363)
FT   DOMAIN        1     29        NON CYTOPLASMIC.
FT   TRANSMEM     30     50
FT   DOMAIN       51     63        CYTOPLASMIC.
FT   TRANSMEM     64     86
FT   DOMAIN       87    111        NON CYTOPLASMIC.
FT   TRANSMEM    112    135
FT   DOMAIN      136    149        CYTOPLASMIC.
FT   TRANSMEM    150    169
FT   DOMAIN      170    204        NON CYTOPLASMIC.
FT   TRANSMEM    205    231
FT   DOMAIN      232    246        CYTOPLASMIC.
FT   TRANSMEM    247    269
FT   DOMAIN      270    320        NON CYTOPLASMIC.
FT   TRANSMEM    321    341
FT   DOMAIN      342    347        CYTOPLASMIC.
//
ID   TcasGr43(AM292364)
FT   DOMAIN        1     33        NON CYTOPLASMIC.
FT   TRANSMEM     34     52
FT   DOMAIN       53     60        CYTOPLASMIC.
FT   TRANSMEM     61     85
FT   DOMAIN       86    119        NON CYTOPLASMIC.
FT   TRANSMEM    120    141
FT   DOMAIN      142    158        CYTOPLASMIC.
FT   TRANSMEM    159    178
FT   DOMAIN      179    210        NON CYTOPLASMIC.
FT   TRANSMEM    211    234
FT   DOMAIN      235    253        CYTOPLASMIC.
FT   TRANSMEM    254    273
FT   DOMAIN      274    325        NON CYTOPLASMIC.
FT   TRANSMEM    326    347
FT   DOMAIN      348    353        CYTOPLASMIC.
//
```

```
ID    TcasGr44(AM292365)
FT    DOMAIN          1     36        NON CYTOPLASMIC.
FT    TRANSMEM       37     55
FT    DOMAIN         56     68        CYTOPLASMIC.
FT    TRANSMEM       69     92
FT    DOMAIN         93    123        NON CYTOPLASMIC.
FT    TRANSMEM      124    146
FT    DOMAIN        147    159        CYTOPLASMIC.
FT    TRANSMEM      160    180
FT    DOMAIN        181    196        NON CYTOPLASMIC.
FT    TRANSMEM      197    217
FT    DOMAIN        218    230        CYTOPLASMIC.
FT    TRANSMEM      231    252
FT    DOMAIN        253    262        NON CYTOPLASMIC.
FT    TRANSMEM      263    285
FT    DOMAIN        286    313        CYTOPLASMIC.
//
ID    TcasGr45(AM292366)
FT    DOMAIN          1     44        NON CYTOPLASMIC.
FT    TRANSMEM       45     64
FT    DOMAIN         65     74        CYTOPLASMIC.
FT    TRANSMEM       75     97
FT    DOMAIN         98    138        NON CYTOPLASMIC.
FT    TRANSMEM      139    160
FT    DOMAIN        161    173        CYTOPLASMIC.
FT    TRANSMEM      174    193
FT    DOMAIN        194    231        NON CYTOPLASMIC.
FT    TRANSMEM      232    258
FT    DOMAIN        259    280        CYTOPLASMIC.
FT    TRANSMEM      281    304
FT    DOMAIN        305    348        NON CYTOPLASMIC.
FT    TRANSMEM      349    370
FT    DOMAIN        371    379        CYTOPLASMIC.
//
ID    TcasGr46(AM292367)
FT    DOMAIN          1   1155        NON CYTOPLASMIC.
FT    TRANSMEM     1156   1175
FT    DOMAIN       1176   1182        CYTOPLASMIC.
FT    TRANSMEM     1183   1201
FT    DOMAIN       1202   1221        NON CYTOPLASMIC.
FT    TRANSMEM     1222   1245
FT    DOMAIN       1246   1266        CYTOPLASMIC.
FT    TRANSMEM     1267   1287
FT    DOMAIN       1288   1319        NON CYTOPLASMIC.
FT    TRANSMEM     1320   1345
FT    DOMAIN       1346   1358        CYTOPLASMIC.
FT    TRANSMEM     1359   1383
FT    DOMAIN       1384   1419        NON CYTOPLASMIC.
FT    TRANSMEM     1420   1442
FT    DOMAIN       1443   1451        CYTOPLASMIC.
//
ID    TcasGr47(AM292354)
FT    DOMAIN          1     19        NON CYTOPLASMIC.
```

```
FT   TRANSMEM     20     37
FT   DOMAIN       38     51       CYTOPLASMIC.
FT   TRANSMEM     52     71
FT   DOMAIN       72     98       NON CYTOPLASMIC.
FT   TRANSMEM     99    121
FT   DOMAIN      122    136       CYTOPLASMIC.
FT   TRANSMEM    137    158
FT   DOMAIN      159    186       NON CYTOPLASMIC.
FT   TRANSMEM    187    207
FT   DOMAIN      208    227       CYTOPLASMIC.
FT   TRANSMEM    228    251
FT   DOMAIN      252    278       NON CYTOPLASMIC.
FT   TRANSMEM    279    298
FT   DOMAIN      299    321       CYTOPLASMIC.
//
```
ID   TcasGr48(AM292369)
```
FT   DOMAIN        1     55       NON CYTOPLASMIC.
FT   TRANSMEM     56     76
FT   DOMAIN       77     87       CYTOPLASMIC.
FT   TRANSMEM     88    110
FT   DOMAIN      111    137       NON CYTOPLASMIC.
FT   TRANSMEM    138    158
FT   DOMAIN      159    174       CYTOPLASMIC.
FT   TRANSMEM    175    194
FT   DOMAIN      195    267       NON CYTOPLASMIC.
FT   TRANSMEM    268    291
FT   DOMAIN      292    332       CYTOPLASMIC.
FT   TRANSMEM    333    351
FT   DOMAIN      352    363       NON CYTOPLASMIC.
FT   TRANSMEM    364    386
FT   DOMAIN      387    408       CYTOPLASMIC.
//
```
ID   TcasGr49(AM292370)
```
FT   DOMAIN        1     55       NON CYTOPLASMIC.
FT   TRANSMEM     56     76
FT   DOMAIN       77     86       CYTOPLASMIC.
FT   TRANSMEM     87    105
FT   DOMAIN      106    115       NON CYTOPLASMIC.
FT   TRANSMEM    116    135
FT   DOMAIN      136    157       CYTOPLASMIC.
FT   TRANSMEM    158    179
FT   DOMAIN      180    267       NON CYTOPLASMIC.
FT   TRANSMEM    268    294
FT   DOMAIN      295    311       CYTOPLASMIC.
FT   TRANSMEM    312    338
FT   DOMAIN      339    378       NON CYTOPLASMIC.
FT   TRANSMEM    379    400
FT   DOMAIN      401    418       CYTOPLASMIC.
//
```
ID   TcasGr50(AM292371)
```
FT   DOMAIN        1     34       NON CYTOPLASMIC.
FT   TRANSMEM     35     55
FT   DOMAIN       56     65       CYTOPLASMIC.
```

```
FT   TRANSMEM     66     87
FT   DOMAIN       88    116       NON CYTOPLASMIC.
FT   TRANSMEM    117    137
FT   DOMAIN      138    153       CYTOPLASMIC.
FT   TRANSMEM    154    175
FT   DOMAIN      176    230       NON CYTOPLASMIC.
FT   TRANSMEM    231    255
FT   DOMAIN      256    266       CYTOPLASMIC.
FT   TRANSMEM    267    287
FT   DOMAIN      288    313       NON CYTOPLASMIC.
FT   TRANSMEM    314    332
FT   DOMAIN      333    350       CYTOPLASMIC.
//
ID   TcasGr51(AM292372)
FT   DOMAIN        1    369       NON CYTOPLASMIC.
FT   TRANSMEM    370    391
FT   DOMAIN      392    403       CYTOPLASMIC.
FT   TRANSMEM    404    423
FT   DOMAIN      424    437       NON CYTOPLASMIC.
FT   TRANSMEM    438    459
FT   DOMAIN      460    474       CYTOPLASMIC.
FT   TRANSMEM    475    496
FT   DOMAIN      497    530       NON CYTOPLASMIC.
FT   TRANSMEM    531    555
FT   DOMAIN      556    637       CYTOPLASMIC.
FT   TRANSMEM    638    659
FT   DOMAIN      660    665       NON CYTOPLASMIC.
FT   TRANSMEM    666    690
FT   DOMAIN      691    771       CYTOPLASMIC.
//
ID   TcasGr52(AM292375)
FT   DOMAIN        1     12       NON CYTOPLASMIC.
FT   TRANSMEM     13     32
FT   DOMAIN       33     38       CYTOPLASMIC.
FT   TRANSMEM     39     57
FT   DOMAIN       58     73       NON CYTOPLASMIC.
FT   TRANSMEM     74     93
FT   DOMAIN       94    112       CYTOPLASMIC.
FT   TRANSMEM    113    132
FT   DOMAIN      133    166       NON CYTOPLASMIC.
FT   TRANSMEM    167    187
FT   DOMAIN      188    208       CYTOPLASMIC.
FT   TRANSMEM    209    230
FT   DOMAIN      231    282       NON CYTOPLASMIC.
FT   TRANSMEM    283    304
FT   DOMAIN      305    311       CYTOPLASMIC.
//
ID   TcasGr53(AM292383)
FT   DOMAIN        1     25       NON CYTOPLASMIC.
FT   TRANSMEM     26     47
FT   DOMAIN       48     57       CYTOPLASMIC.
FT   TRANSMEM     58     80
FT   DOMAIN       81    107       NON CYTOPLASMIC.
```

```
FT   TRANSMEM    108    129
FT   DOMAIN      130    143       CYTOPLASMIC.
FT   TRANSMEM    144    164
FT   DOMAIN      165    194       NON CYTOPLASMIC.
FT   TRANSMEM    195    218
FT   DOMAIN      219    236       CYTOPLASMIC.
FT   TRANSMEM    237    260
FT   DOMAIN      261    283       NON CYTOPLASMIC.
FT   TRANSMEM    284    303
FT   DOMAIN      304    319       CYTOPLASMIC.
//
ID   TcasGr54(AM292373)
FT   DOMAIN        1     32       NON CYTOPLASMIC.
FT   TRANSMEM     33     52
FT   DOMAIN       53     59       CYTOPLASMIC.
FT   TRANSMEM     60     80
FT   DOMAIN       81    114       NON CYTOPLASMIC.
FT   TRANSMEM    115    137
FT   DOMAIN      138    154       CYTOPLASMIC.
FT   TRANSMEM    155    174
FT   DOMAIN      175    217       NON CYTOPLASMIC.
FT   TRANSMEM    218    244
FT   DOMAIN      245    254       CYTOPLASMIC.
FT   TRANSMEM    255    278
FT   DOMAIN      279    324       NON CYTOPLASMIC.
FT   TRANSMEM    325    347
FT   DOMAIN      348    360       CYTOPLASMIC.
//
ID   TcasGr125(AM292376)
FT   DOMAIN        1     56       NON CYTOPLASMIC.
FT   TRANSMEM     57     77
FT   DOMAIN       78     89       CYTOPLASMIC.
FT   TRANSMEM     90    112
FT   DOMAIN      113    140       NON CYTOPLASMIC.
FT   TRANSMEM    141    161
FT   DOMAIN      162    175       CYTOPLASMIC.
FT   TRANSMEM    176    197
FT   DOMAIN      198    240       NON CYTOPLASMIC.
FT   TRANSMEM    241    264
FT   DOMAIN      265    276       CYTOPLASMIC.
FT   TRANSMEM    277    297
FT   DOMAIN      298    358       NON CYTOPLASMIC.
FT   TRANSMEM    359    381
FT   DOMAIN      382    402       CYTOPLASMIC.
//
ID   TcasGr56(AM292377)
FT   DOMAIN        1     48       NON CYTOPLASMIC.
FT   TRANSMEM     49     74
FT   DOMAIN       75     89       CYTOPLASMIC.
FT   TRANSMEM     90    115
FT   DOMAIN      116    143       NON CYTOPLASMIC.
FT   TRANSMEM    144    164
FT   DOMAIN      165    176       CYTOPLASMIC.
```

```
FT   TRANSMEM    177    196
FT   DOMAIN      197    250        NON CYTOPLASMIC.
FT   TRANSMEM    251    272
FT   DOMAIN      273    286        CYTOPLASMIC.
FT   TRANSMEM    287    308
FT   DOMAIN      309    325        NON CYTOPLASMIC.
FT   TRANSMEM    326    345
FT   DOMAIN      346    358        CYTOPLASMIC.
//
ID   TcasGr57(AM292378)
FT   DOMAIN        1     27        NON CYTOPLASMIC.
FT   TRANSMEM     28     51
FT   DOMAIN       52     64        CYTOPLASMIC.
FT   TRANSMEM     65     89
FT   DOMAIN       90    112        NON CYTOPLASMIC.
FT   TRANSMEM    113    134
FT   DOMAIN      135    151        CYTOPLASMIC.
FT   TRANSMEM    152    171
FT   DOMAIN      172    240        NON CYTOPLASMIC.
FT   TRANSMEM    241    265
FT   DOMAIN      266    281        CYTOPLASMIC.
FT   TRANSMEM    282    302
FT   DOMAIN      303    359        NON CYTOPLASMIC.
FT   TRANSMEM    360    382
FT   DOMAIN      383    387        CYTOPLASMIC.
//
ID   TcasGr98(AM292379)
FT   DOMAIN        1     43        NON CYTOPLASMIC.
FT   TRANSMEM     44     63
FT   DOMAIN       64     74        CYTOPLASMIC.
FT   TRANSMEM     75     97
FT   DOMAIN       98    132        NON CYTOPLASMIC.
FT   TRANSMEM    133    154
FT   DOMAIN      155    171        CYTOPLASMIC.
FT   TRANSMEM    172    191
FT   DOMAIN      192    228        NON CYTOPLASMIC.
FT   TRANSMEM    229    253
FT   DOMAIN      254    274        CYTOPLASMIC.
FT   TRANSMEM    275    295
FT   DOMAIN      296    345        NON CYTOPLASMIC.
FT   TRANSMEM    346    367
FT   DOMAIN      368    376        CYTOPLASMIC.
//
ID   TcasGr59(AM292380)
FT   DOMAIN        1     42        NON CYTOPLASMIC.
FT   TRANSMEM     43     61
FT   DOMAIN       62     72        CYTOPLASMIC.
FT   TRANSMEM     73     96
FT   DOMAIN       97    129        NON CYTOPLASMIC.
FT   TRANSMEM    130    152
FT   DOMAIN      153    168        CYTOPLASMIC.
FT   TRANSMEM    169    191
FT   DOMAIN      192    239        NON CYTOPLASMIC.
```

```
FT   TRANSMEM    240    266
FT   DOMAIN      267    282       CYTOPLASMIC.
FT   TRANSMEM    283    305
FT   DOMAIN      306    348       NON CYTOPLASMIC.
FT   TRANSMEM    349    371
FT   DOMAIN      372    489       CYTOPLASMIC.
//
```
ID   TcasGr60(AM292381)
```
FT   SIGNAL        1     16
FT   DOMAIN        1      1       N-REGION.
FT   DOMAIN        2     11       H-REGION.
FT   DOMAIN       12     16       C-REGION.
FT   DOMAIN       17    102       NON CYTOPLASMIC.
FT   TRANSMEM    103    123
FT   DOMAIN      124    136       CYTOPLASMIC.
FT   TRANSMEM    137    155
FT   DOMAIN      156    182       NON CYTOPLASMIC.
FT   TRANSMEM    183    201
FT   DOMAIN      202    216       CYTOPLASMIC.
FT   TRANSMEM    217    237
FT   DOMAIN      238    250       NON CYTOPLASMIC.
FT   TRANSMEM    251    272
FT   DOMAIN      273    294       CYTOPLASMIC.
FT   TRANSMEM    295    315
FT   DOMAIN      316    325       NON CYTOPLASMIC.
FT   TRANSMEM    326    348
FT   DOMAIN      349    364       CYTOPLASMIC.
//
```
ID   TcasGr61(AM292382)
```
FT   SIGNAL        1     27
FT   DOMAIN        1      9       N-REGION.
FT   DOMAIN       10     22       H-REGION.
FT   DOMAIN       23     27       C-REGION.
FT   DOMAIN       28    254       NON CYTOPLASMIC.
FT   TRANSMEM    255    277
FT   DOMAIN      278    285       CYTOPLASMIC.
FT   TRANSMEM    286    305
FT   DOMAIN      306    323       NON CYTOPLASMIC.
FT   TRANSMEM    324    345
FT   DOMAIN      346    362       CYTOPLASMIC.
FT   TRANSMEM    363    384
FT   DOMAIN      385    416       NON CYTOPLASMIC.
FT   TRANSMEM    417    440
FT   DOMAIN      441    452       CYTOPLASMIC.
FT   TRANSMEM    453    479
FT   DOMAIN      480    535       NON CYTOPLASMIC.
FT   TRANSMEM    536    558
FT   DOMAIN      559    670       CYTOPLASMIC.
//
```
ID   TcasGr62(AM292374)
```
FT   SIGNAL        1     25
FT   DOMAIN        1      9       N-REGION.
FT   DOMAIN       10     20       H-REGION.
```

```
FT   DOMAIN       21     25       C-REGION.
FT   DOMAIN       26    231       NON CYTOPLASMIC.
FT   TRANSMEM    232    254
FT   DOMAIN      255    263       CYTOPLASMIC.
FT   TRANSMEM    264    284
FT   DOMAIN      285    314       NON CYTOPLASMIC.
FT   TRANSMEM    315    337
FT   DOMAIN      338    353       CYTOPLASMIC.
FT   TRANSMEM    354    373
FT   DOMAIN      374    409       NON CYTOPLASMIC.
FT   TRANSMEM    410    432
FT   DOMAIN      433    524       CYTOPLASMIC.
FT   TRANSMEM    525    547
FT   DOMAIN      548    555       NON CYTOPLASMIC.
FT   TRANSMEM    556    577
FT   DOMAIN      578    659       CYTOPLASMIC.
//
ID   TcasOr1(AM689931)
FT   DOMAIN        1     64       NON CYTOPLASMIC.
FT   TRANSMEM     65     84
FT   DOMAIN       85     96       CYTOPLASMIC.
FT   TRANSMEM     97    120
FT   DOMAIN      121    131       NON CYTOPLASMIC.
FT   TRANSMEM    132    157
FT   DOMAIN      158    177       CYTOPLASMIC.
FT   TRANSMEM    178    196
FT   DOMAIN      197    212       NON CYTOPLASMIC.
FT   TRANSMEM    213    239
FT   DOMAIN      240    288       CYTOPLASMIC.
FT   TRANSMEM    289    313
FT   DOMAIN      314    319       NON CYTOPLASMIC.
FT   TRANSMEM    320    341
FT   DOMAIN      342    353       CYTOPLASMIC.
//
ID   TcasOr2(AM689904)
FT   DOMAIN        1     20       NON CYTOPLASMIC.
FT   TRANSMEM     21     41
FT   DOMAIN       42     49       CYTOPLASMIC.
FT   TRANSMEM     50     69
FT   DOMAIN       70    100       NON CYTOPLASMIC.
FT   TRANSMEM    101    121
FT   DOMAIN      122    139       CYTOPLASMIC.
FT   TRANSMEM    140    160
FT   DOMAIN      161    184       NON CYTOPLASMIC.
FT   TRANSMEM    185    207
FT   DOMAIN      208    249       CYTOPLASMIC.
FT   TRANSMEM    250    272
FT   DOMAIN      273    282       NON CYTOPLASMIC.
FT   TRANSMEM    283    307
FT   DOMAIN      308    314       CYTOPLASMIC.
//
ID   TcasOr3(AM689905)
FT   DOMAIN        1      8       NON CYTOPLASMIC.
```

FT	TRANSMEM	9	29	
FT	DOMAIN	30	41	CYTOPLASMIC.
FT	TRANSMEM	42	62	
FT	DOMAIN	63	95	NON CYTOPLASMIC.
FT	TRANSMEM	96	117	
FT	DOMAIN	118	139	CYTOPLASMIC.
FT	TRANSMEM	140	163	
FT	DOMAIN	164	199	NON CYTOPLASMIC.
FT	TRANSMEM	200	225	
FT	DOMAIN	226	285	CYTOPLASMIC.
FT	TRANSMEM	286	307	
FT	DOMAIN	308	315	NON CYTOPLASMIC.
FT	TRANSMEM	316	335	
FT	DOMAIN	336	413	CYTOPLASMIC.
//				
**ID**	**TcasOr4(AM689906)**			
FT	DOMAIN	1	28	NON CYTOPLASMIC.
FT	TRANSMEM	29	50	
FT	DOMAIN	51	58	CYTOPLASMIC.
FT	TRANSMEM	59	78	
FT	DOMAIN	79	88	NON CYTOPLASMIC.
FT	TRANSMEM	89	109	
FT	DOMAIN	110	125	CYTOPLASMIC.
FT	TRANSMEM	126	144	
FT	DOMAIN	145	169	NON CYTOPLASMIC.
FT	TRANSMEM	170	197	
FT	DOMAIN	198	259	CYTOPLASMIC.
FT	TRANSMEM	260	281	
FT	DOMAIN	282	307	NON CYTOPLASMIC.
FT	TRANSMEM	308	332	
FT	DOMAIN	333	378	CYTOPLASMIC.
//				
**ID**	**TcasOr5(AM689907)**			
FT	DOMAIN	1	10	NON CYTOPLASMIC.
FT	TRANSMEM	11	30	
FT	DOMAIN	31	44	CYTOPLASMIC.
FT	TRANSMEM	45	64	
FT	DOMAIN	65	76	NON CYTOPLASMIC.
FT	TRANSMEM	77	99	
FT	DOMAIN	100	123	CYTOPLASMIC.
FT	TRANSMEM	124	146	
FT	DOMAIN	147	164	NON CYTOPLASMIC.
FT	TRANSMEM	165	188	
FT	DOMAIN	189	228	CYTOPLASMIC.
FT	TRANSMEM	229	250	
FT	DOMAIN	251	265	NON CYTOPLASMIC.
FT	TRANSMEM	266	285	
FT	DOMAIN	286	299	CYTOPLASMIC.
//				
**ID**	**TcasOr6(AM689908)**			
FT	SIGNAL	1	32	
FT	DOMAIN	1	14	N-REGION.
FT	DOMAIN	15	27	H-REGION.

```
FT   DOMAIN       28     32       C-REGION.
FT   DOMAIN       33    100       NON CYTOPLASMIC.
FT   TRANSMEM    101    122
FT   DOMAIN      123    140       CYTOPLASMIC.
FT   TRANSMEM    141    165
FT   DOMAIN      166    181       NON CYTOPLASMIC.
FT   TRANSMEM    182    202
FT   DOMAIN      203    221       CYTOPLASMIC.
FT   TRANSMEM    222    243
FT   DOMAIN      244    270       NON CYTOPLASMIC.
FT   TRANSMEM    271    293
FT   DOMAIN      294    362       CYTOPLASMIC.
FT   TRANSMEM    363    384
FT   DOMAIN      385    409       NON CYTOPLASMIC.
FT   TRANSMEM    410    433
FT   DOMAIN      434    475       CYTOPLASMIC.
//
ID   TcasOr7(AM689909)
FT   DOMAIN        1      7       NON CYTOPLASMIC.
FT   TRANSMEM      8     27
FT   DOMAIN       28     40       CYTOPLASMIC.
FT   TRANSMEM     41     60
FT   DOMAIN       61     79       NON CYTOPLASMIC.
FT   TRANSMEM     80    104
FT   DOMAIN      105    125       CYTOPLASMIC.
FT   TRANSMEM    126    149
FT   DOMAIN      150    175       NON CYTOPLASMIC.
FT   TRANSMEM    176    200
FT   DOMAIN      201    223       CYTOPLASMIC.
FT   TRANSMEM    224    251
FT   DOMAIN      252    288       NON CYTOPLASMIC.
FT   TRANSMEM    289    310
FT   DOMAIN      311    311       CYTOPLASMIC.
//
ID   TcasOr63(AM689910)
FT   DOMAIN        1     35       NON CYTOPLASMIC.
FT   TRANSMEM     36     62
FT   DOMAIN       63     73       CYTOPLASMIC.
FT   TRANSMEM     74     94
FT   DOMAIN       95    132       NON CYTOPLASMIC.
FT   TRANSMEM    133    154
FT   DOMAIN      155    171       CYTOPLASMIC.
FT   TRANSMEM    172    193
FT   DOMAIN      194    249       NON CYTOPLASMIC.
FT   TRANSMEM    250    273
FT   DOMAIN      274    284       CYTOPLASMIC.
FT   TRANSMEM    285    305
FT   DOMAIN      306    307       NON CYTOPLASMIC.
FT   TRANSMEM    308    329
FT   DOMAIN      330    383       CYTOPLASMIC.
//
ID   TcasOr9(AM689911)
FT   DOMAIN        1     55       NON CYTOPLASMIC.
```

FT	TRANSMEM	56	75	
FT	DOMAIN	76	81	CYTOPLASMIC.
FT	TRANSMEM	82	101	
FT	DOMAIN	102	121	NON CYTOPLASMIC.
FT	TRANSMEM	122	143	
FT	DOMAIN	144	160	CYTOPLASMIC.
FT	TRANSMEM	161	182	
FT	DOMAIN	183	196	NON CYTOPLASMIC.
FT	TRANSMEM	197	219	
FT	DOMAIN	220	229	CYTOPLASMIC.
FT	TRANSMEM	230	252	
FT	DOMAIN	253	259	NON CYTOPLASMIC.
FT	TRANSMEM	260	280	
FT	DOMAIN	281	294	CYTOPLASMIC.
//				
**ID**	**TcasOr10(AM689912)**			
FT	DOMAIN	1	44	NON CYTOPLASMIC.
FT	TRANSMEM	45	67	
FT	DOMAIN	68	86	CYTOPLASMIC.
FT	TRANSMEM	87	106	
FT	DOMAIN	107	121	NON CYTOPLASMIC.
FT	TRANSMEM	122	142	
FT	DOMAIN	143	160	CYTOPLASMIC.
FT	TRANSMEM	161	183	
FT	DOMAIN	184	199	NON CYTOPLASMIC.
FT	TRANSMEM	200	223	
FT	DOMAIN	224	262	CYTOPLASMIC.
FT	TRANSMEM	263	287	
FT	DOMAIN	288	302	NON CYTOPLASMIC.
FT	TRANSMEM	303	324	
FT	DOMAIN	325	330	CYTOPLASMIC.
//				
**ID**	**TcasOr11(AM689913)**			
FT	DOMAIN	1	31	NON CYTOPLASMIC.
FT	TRANSMEM	32	53	
FT	DOMAIN	54	65	CYTOPLASMIC.
FT	TRANSMEM	66	88	
FT	DOMAIN	89	113	NON CYTOPLASMIC.
FT	TRANSMEM	114	136	
FT	DOMAIN	137	159	CYTOPLASMIC.
FT	TRANSMEM	160	180	
FT	DOMAIN	181	237	NON CYTOPLASMIC.
FT	TRANSMEM	238	261	
FT	DOMAIN	262	286	CYTOPLASMIC.
FT	TRANSMEM	287	305	
FT	DOMAIN	306	308	NON CYTOPLASMIC.
FT	TRANSMEM	309	329	
FT	DOMAIN	330	332	CYTOPLASMIC.
//				
**ID**	**TcasOr12(AM689914)**			
FT	DOMAIN	1	8	NON CYTOPLASMIC.
FT	TRANSMEM	9	27	
FT	DOMAIN	28	38	CYTOPLASMIC.

```
FT   TRANSMEM     39    63
FT   DOMAIN       64    89       NON CYTOPLASMIC.
FT   TRANSMEM     90   113
FT   DOMAIN      114   131       CYTOPLASMIC.
FT   TRANSMEM    132   154
FT   DOMAIN      155   188       NON CYTOPLASMIC.
FT   TRANSMEM    189   212
FT   DOMAIN      213   263       CYTOPLASMIC.
FT   TRANSMEM    264   287
FT   DOMAIN      288   293       NON CYTOPLASMIC.
FT   TRANSMEM    294   315
FT   DOMAIN      316   374       CYTOPLASMIC.
//
```
ID   TcasOr13(AM689915)
```
FT   DOMAIN        1    47       NON CYTOPLASMIC.
FT   TRANSMEM     48    68
FT   DOMAIN       69    77       CYTOPLASMIC.
FT   TRANSMEM     78   101
FT   DOMAIN      102   140       NON CYTOPLASMIC.
FT   TRANSMEM    141   162
FT   DOMAIN      163   178       CYTOPLASMIC.
FT   TRANSMEM    179   200
FT   DOMAIN      201   266       NON CYTOPLASMIC.
FT   TRANSMEM    267   290
FT   DOMAIN      291   306       CYTOPLASMIC.
FT   TRANSMEM    307   326
FT   DOMAIN      327   379       NON CYTOPLASMIC.
FT   TRANSMEM    380   401
FT   DOMAIN      402   405       CYTOPLASMIC.
//
```
ID   TcasOr56(AM689916)
```
FT   DOMAIN        1    38       NON CYTOPLASMIC.
FT   TRANSMEM     39    60
FT   DOMAIN       61    71       CYTOPLASMIC.
FT   TRANSMEM     72    92
FT   DOMAIN       93   134       NON CYTOPLASMIC.
FT   TRANSMEM    135   154
FT   DOMAIN      155   169       CYTOPLASMIC.
FT   TRANSMEM    170   189
FT   DOMAIN      190   213       NON CYTOPLASMIC.
FT   TRANSMEM    214   236
FT   DOMAIN      237   306       CYTOPLASMIC.
FT   TRANSMEM    307   330
FT   DOMAIN      331   336       NON CYTOPLASMIC.
FT   TRANSMEM    337   358
FT   DOMAIN      359   433       CYTOPLASMIC.
//
```
ID   TcasOr15(AM689917)
```
FT   DOMAIN        1    36       NON CYTOPLASMIC.
FT   TRANSMEM     37    62
FT   DOMAIN       63    69       CYTOPLASMIC.
FT   TRANSMEM     70    89
FT   DOMAIN       90    99       NON CYTOPLASMIC.
```

```
FT   TRANSMEM    100    123
FT   DOMAIN      124    136       CYTOPLASMIC.
FT   TRANSMEM    137    156
FT   DOMAIN      157    199       NON CYTOPLASMIC.
FT   TRANSMEM    200    227
FT   DOMAIN      228    243       CYTOPLASMIC.
FT   TRANSMEM    244    266
FT   DOMAIN      267    301       NON CYTOPLASMIC.
FT   TRANSMEM    302    325
FT   DOMAIN      326    340       CYTOPLASMIC.
//
ID   TcasOr16(AM689918)
FT   DOMAIN        1    203       NON CYTOPLASMIC.
FT   TRANSMEM    204    228
FT   DOMAIN      229    241       CYTOPLASMIC.
FT   TRANSMEM    242    263
FT   DOMAIN      264    298       NON CYTOPLASMIC.
FT   TRANSMEM    299    321
FT   DOMAIN      322    336       CYTOPLASMIC.
FT   TRANSMEM    337    357
FT   DOMAIN      358    379       NON CYTOPLASMIC.
FT   TRANSMEM    380    400
FT   DOMAIN      401    426       CYTOPLASMIC.
FT   TRANSMEM    427    447
FT   DOMAIN      448    449       NON CYTOPLASMIC.
FT   TRANSMEM    450    471
FT   DOMAIN      472    475       CYTOPLASMIC.
//
ID   TcasOr17(AM689919)
FT   DOMAIN        1      3       NON CYTOPLASMIC.
FT   TRANSMEM      4     22
FT   DOMAIN       23     29       CYTOPLASMIC.
FT   TRANSMEM     30     47
FT   DOMAIN       48     59       NON CYTOPLASMIC.
FT   TRANSMEM     60     80
FT   DOMAIN       81     93       CYTOPLASMIC.
FT   TRANSMEM     94    112
FT   DOMAIN      113    127       NON CYTOPLASMIC.
FT   TRANSMEM    128    149
FT   DOMAIN      150    175       CYTOPLASMIC.
FT   TRANSMEM    176    195
FT   DOMAIN      196    197       NON CYTOPLASMIC.
FT   TRANSMEM    198    217
FT   DOMAIN      218    243       CYTOPLASMIC.
//
ID   TcasOr18(AM689920)
FT   DOMAIN        1     32       NON CYTOPLASMIC.
FT   TRANSMEM     33     58
FT   DOMAIN       59     66       CYTOPLASMIC.
FT   TRANSMEM     67     86
FT   DOMAIN       87    115       NON CYTOPLASMIC.
FT   TRANSMEM    116    136
FT   DOMAIN      137    157       CYTOPLASMIC.
```

```
FT   TRANSMEM    158    179
FT   DOMAIN      180    238        NON CYTOPLASMIC.
FT   TRANSMEM    239    259
FT   DOMAIN      260    282        CYTOPLASMIC.
FT   TRANSMEM    283    304
FT   DOMAIN      305    324        NON CYTOPLASMIC.
FT   TRANSMEM    325    346
FT   DOMAIN      347    410        CYTOPLASMIC.
//
ID   TcasOr19(AM689921)
FT   DOMAIN        1     38        NON CYTOPLASMIC.
FT   TRANSMEM     39     57
FT   DOMAIN       58     62        CYTOPLASMIC.
FT   TRANSMEM     63     81
FT   DOMAIN       82     93        NON CYTOPLASMIC.
FT   TRANSMEM     94    114
FT   DOMAIN      115    136        CYTOPLASMIC.
FT   TRANSMEM    137    159
FT   DOMAIN      160    219        NON CYTOPLASMIC.
FT   TRANSMEM    220    242
FT   DOMAIN      243    254        CYTOPLASMIC.
FT   TRANSMEM    255    276
FT   DOMAIN      277    310        NON CYTOPLASMIC.
FT   TRANSMEM    311    330
FT   DOMAIN      331    331        CYTOPLASMIC.
//
ID   TcasOr20(AM689922)
FT   DOMAIN        1     58        NON CYTOPLASMIC.
FT   TRANSMEM     59     80
FT   DOMAIN       81     90        CYTOPLASMIC.
FT   TRANSMEM     91    111
FT   DOMAIN      112    151        NON CYTOPLASMIC.
FT   TRANSMEM    152    169
FT   DOMAIN      170    186        CYTOPLASMIC.
FT   TRANSMEM    187    211
FT   DOMAIN      212    256        NON CYTOPLASMIC.
FT   TRANSMEM    257    279
FT   DOMAIN      280    290        CYTOPLASMIC.
FT   TRANSMEM    291    311
FT   DOMAIN      312    360        NON CYTOPLASMIC.
FT   TRANSMEM    361    381
FT   DOMAIN      382    386        CYTOPLASMIC.
//
ID   TcasOr21(AM689923)
FT   DOMAIN        1      5        NON CYTOPLASMIC.
FT   TRANSMEM      6     23
FT   DOMAIN       24     30        CYTOPLASMIC.
FT   TRANSMEM     31     48
FT   DOMAIN       49     58        NON CYTOPLASMIC.
FT   TRANSMEM     59     79
FT   DOMAIN       80     92        CYTOPLASMIC.
FT   TRANSMEM     93    112
FT   DOMAIN      113    127        NON CYTOPLASMIC.
```

```
FT   TRANSMEM    128    149
FT   DOMAIN      150    173        CYTOPLASMIC.
FT   TRANSMEM    174    193
FT   DOMAIN      194    195        NON CYTOPLASMIC.
FT   TRANSMEM    196    215
FT   DOMAIN      216    240        CYTOPLASMIC.
//
ID   TcasOr22(AM689924)
FT   DOMAIN        1     33        NON CYTOPLASMIC.
FT   TRANSMEM     34     55
FT   DOMAIN       56     63        CYTOPLASMIC.
FT   TRANSMEM     64     86
FT   DOMAIN       87    115        NON CYTOPLASMIC.
FT   TRANSMEM    116    136
FT   DOMAIN      137    154        CYTOPLASMIC.
FT   TRANSMEM    155    176
FT   DOMAIN      177    252        NON CYTOPLASMIC.
FT   TRANSMEM    253    276
FT   DOMAIN      277    292        CYTOPLASMIC.
FT   TRANSMEM    293    313
FT   DOMAIN      314    347        NON CYTOPLASMIC.
FT   TRANSMEM    348    368
FT   DOMAIN      369    382        CYTOPLASMIC.
//
ID   TcasOr23(AM689925)
FT   DOMAIN        1     33        NON CYTOPLASMIC.
FT   TRANSMEM     34     55
FT   DOMAIN       56     63        CYTOPLASMIC.
FT   TRANSMEM     64     86
FT   DOMAIN       87    112        NON CYTOPLASMIC.
FT   TRANSMEM    113    136
FT   DOMAIN      137    153        CYTOPLASMIC.
FT   TRANSMEM    154    174
FT   DOMAIN      175    187        NON CYTOPLASMIC.
FT   TRANSMEM    188    211
FT   DOMAIN      212    254        CYTOPLASMIC.
FT   TRANSMEM    255    276
FT   DOMAIN      277    278        NON CYTOPLASMIC.
FT   TRANSMEM    279    298
FT   DOMAIN      299    360        CYTOPLASMIC.
//
ID   TcasOr24(AM689926)
FT   DOMAIN        1     39        NON CYTOPLASMIC.
FT   TRANSMEM     40     60
FT   DOMAIN       61     69        CYTOPLASMIC.
FT   TRANSMEM     70     92
FT   DOMAIN       93    136        NON CYTOPLASMIC.
FT   TRANSMEM    137    160
FT   DOMAIN      161    182        CYTOPLASMIC.
FT   TRANSMEM    183    203
FT   DOMAIN      204    251        NON CYTOPLASMIC.
FT   TRANSMEM    252    277
FT   DOMAIN      278    288        CYTOPLASMIC.
```

```
FT   TRANSMEM    289    311
FT   DOMAIN      312    323        NON CYTOPLASMIC.
FT   TRANSMEM    324    342
FT   DOMAIN      343    343        CYTOPLASMIC.
//
```
ID   TcasOr25(AM689927)
```
FT   DOMAIN        1    383        NON CYTOPLASMIC.
FT   TRANSMEM    384    408
FT   DOMAIN      409    421        CYTOPLASMIC.
FT   TRANSMEM    422    440
FT   DOMAIN      441    469        NON CYTOPLASMIC.
FT   TRANSMEM    470    492
FT   DOMAIN      493    508        CYTOPLASMIC.
FT   TRANSMEM    509    530
FT   DOMAIN      531    551        NON CYTOPLASMIC.
FT   TRANSMEM    552    575
FT   DOMAIN      576    614        CYTOPLASMIC.
FT   TRANSMEM    615    637
FT   DOMAIN      638    752        NON CYTOPLASMIC.
FT   TRANSMEM    753    775
FT   DOMAIN      776    791        CYTOPLASMIC.
//
```
ID   TcasOr26(AM689928)
```
FT   DOMAIN        1     25        NON CYTOPLASMIC.
FT   TRANSMEM     26     46
FT   DOMAIN       47     53        CYTOPLASMIC.
FT   TRANSMEM     54     72
FT   DOMAIN       73     82        NON CYTOPLASMIC.
FT   TRANSMEM     83    101
FT   DOMAIN      102    114        CYTOPLASMIC.
FT   TRANSMEM    115    134
FT   DOMAIN      135    167        NON CYTOPLASMIC.
FT   TRANSMEM    168    191
FT   DOMAIN      192    248        CYTOPLASMIC.
FT   TRANSMEM    249    269
FT   DOMAIN      270    304        NON CYTOPLASMIC.
FT   TRANSMEM    305    324
FT   DOMAIN      325    338        CYTOPLASMIC.
```

C

```
TcasGr38        ------------------------------------------------------------
TcasGr46        ------------------------------------------------------------
TcasGr1         ------------------------------------------------------------
TcasGr123       ------------------------------------------------------------
TcasGr62        MMDRINHKKFINTTKLLLWCDQTIGLITFDINTPSFKLSKIRSFLNITASAVLLPFAIYH 60
TcasGr25        ------------------------------------------------------------
TcasGr59        ------------------------------------------------------------
AgamGr2         ------------------------------------------------------------
AaegGr66a       ------------------------------------------------------------
DmelGr66a       ------------------------------------------------------------
TcasGr11        ------------------------------------------------------------
TcasGr55        ------------------------------------------------------------
TcasGr21        ------------------------------------------------------------
TcasGr56        ------------------------------------------------------------
AaegGr          ------------------------------------------------------------
AaegGr28a       ------------------------------------------------------------
AgamGr33        ------------------------------------------------------------
DmelGr28bB      ------------------------------------------------------------
DmelGr28bC      ------------------------------------------------------------
DmelGr28bD      ------------------------------------------------------------
DmelGr28bA      ------------------------------------------------------------
DmelGr28bE      ------------------------------------------------------------
AgamGr25        ------------------------------------------------------------
AaegGr43a       ------------------------------------------------------------
DmelGr43a       ------------------------------------------------------------
AgamGr37b       ------------------------------------------------------------
AgamGr37d       ------------------------------------------------------------
AgamGr37a       ------------------------------------------------------------
AgamGr37c       ------------------------------------------------------------
AgamGr37e       ------------------------------------------------------------
AgamGr37f       ------------------------------------------------------------
AaegGr28c       ------------------------------------------------------------
AaegGr28b       ------------------------------------------------------------
AaegGr28e       ------------------------------------------------------------
TcasGr14        ------------------------------------------------------------
TcasGr49        ------------------------------------------------------------
AmelGr4         ------------------------------------------------------------
AmelGr5         ------------------------------------------------------------
TcasGr16        ------------------------------------------------------------
TcasGr22        ------------------------------------------------------------
TcasGr17        ------------------------------------------------------------
TcasGr150       ------------------------------------------------------------
TcasGr32        ------------------------------------------------------------
TcasGr54        ------------------------------------------------------------
TcasGr37        ------------------------------------------------------------
TcasGr53        ------------------------------------------------------------
TcasGr5         ------------------------------------------------------------
TcasGr12        ------------------------------------------------------------
TcasGr13        ------------------------------------------------------------
TcasGr104       ------------------------------------------------------------
TcasGr45        ------------------------------------------------------------
TcasGr98        ------------------------------------------------------------
TcasGr52        ------------------------------------------------------------
TcasGr105       ------------------------------------------------------------
TcasGr4         ------------------------------------------------------------
TcasGr44        ------------------------------------------------------------
AgamGr9a        ------------------------------------------------------------
AgamGr9c        ------------------------------------------------------------
AgamGr9b        ------------------------------------------------------------
AgamGr9d        ------------------------------------------------------------
AgamGr9e        ------------------------------------------------------------
AgamGr9f        ------------------------------------------------------------
AgamGr9g        ------------------------------------------------------------
AgamGr9i        ------------------------------------------------------------
AgamGr9h        ------------------------------------------------------------
AgamGr9j        ------------------------------------------------------------
AgamGr9l        ------------------------------------------------------------
AgamGr9k        ------------------------------------------------------------
```

```
AgamGr9m         ------------------------------------------------------------
AgamGr9n         ------------------------------------------------------------
AgamGr10         ------------------------------------------------------------
AgamGr11         ------------------------------------------------------------
DmelGr23aA       ------------------------------------------------------------
DmelGr23aB       ------------------------------------------------------------
DmelGr39aA       ------------------------------------------------------------
DmelGr39aB       ------------------------------------------------------------
DmelGr39aC       ------------------------------------------------------------
DmelGr39aD       ------------------------------------------------------------
DmelGr98c        ------------------------------------------------------------
DmelGr98d        ------------------------------------------------------------
DmelGr98b        ------------------------------------------------------------
AgamGr51         ------------------------------------------------------------
AgamGr52         ------------------------------------------------------------
DmelGr94a        ------------------------------------------------------------
DmelGr97a        ------------------------------------------------------------
DmelGr93b        ------------------------------------------------------------
DmelGr93c        ------------------------------------------------------------
DmelGr22d        ------------------------------------------------------------
DmelGr22e        ------------------------------------------------------------
DmelGr22a        ------------------------------------------------------------
DmelGr22b        ------------------------------------------------------------
DmelGr22c        ------------------------------------------------------------
DmelGr22f        ------------------------------------------------------------
DmelGr36a        ------------------------------------------------------------
DmelGr36c        ------------------------------------------------------------
DmelGr36b        ------------------------------------------------------------
DmelGr59d        ------------------------------------------------------------
DmelGr59a        ------------------------------------------------------------
DmelGr59b        ------------------------------------------------------------
AgamGr26         ------------------------------------------------------------
AgamGr27         ------------------------------------------------------------
AgamGr29         ------------------------------------------------------------
AgamGr31         ------------------------------------------------------------
AgamGr30         ------------------------------------------------------------
AgamGr32a        ------------------------------------------------------------
TcasGr2          ------------------------------------------------------------
TcasGr9          ------------------------------------------------------------
TcasGr7          ------------------------------------------------------------
TcasGr30         ------------------------------------------------------------
TcasGr29         ------------------------------------------------------------
AaegGr64f        ------------------------------------------------------------
AaegGr64a3       ------------------------------------------------------------
AgamGr14         ------------------------------------------------------------
AgamGr18         ------------------------------------------------------------
AaegGr64d        ------------------------------------------------------------
AgamGr17         ------------------------------------------------------------
AaegGr64c        ------------------------------------------------------------
DmelGr64c        ------------------------------------------------------------
DmelGr64d        ------------------------------------------------------------
AgamGr20         ------------------------------------------------------------
AaegGr61a        ------------------------------------------------------------
AgamGr21         ------------------------------------------------------------
AaegGr64a        ------------------------------------------------------------
DmelGr64a        ------------------------------------------------------------
DmelGr61a        ------------------------------------------------------------
AgamGr15         ------------------------------------------------------------
AaegGr1          ------------------------------------------------------------
DmelGr5a         ------------------------------------------------------------
DmelGr64f        ------------------------------------------------------------
DmelGr64e        ------------------------------------------------------------
AmelGr1          ------------------------------------------------------------
AmelGr2          ------------------------------------------------------------
AgamGr16         ------------------------------------------------------------
AaegGr64e        ------------------------------------------------------------
TCasGr6          ------------------------------------------------------------
TcasGr26         ------------------------------------------------------------
TcasGr33         ------------------------------------------------------------
AgamGr23         ------------------------------------------------------------
```

```
AaegGr21a       ------------------------------------------------------------
TcasGr10        ------------------------------------------------------------
TcasGr39        ------------------------------------------------------------
AgamGr22        ------------------------------------------------------------
AaegGr21b       ------------------------------------------------------------
DmelGr21a       ------------------------------------------------------------
AgamGr24        ------------------------------------------------------------
AaegGr63a       ------------------------------------------------------------
DmelGr63a       ------------------------------------------------------------
TcasGr3         ------------------------------------------------------------
DmelOr83b       ------------------------------------------------------------
AgamOr7         ------------------------------------------------------------
HvirOr2         ------------------------------------------------------------
TcasOr16        -----------------------------MSKIALNNPKSEYPSK------------QDP 19
TcasOr22        -----------------------------MSKIALNNPKSEYPSKYRPRPHRSIPAPQDP 31
AmelOr2         ------------------------------------------------------------
TcasGr31        ------------------------------------------------------------
TcasGr35        ------------------------------------------------------------
DmelGr59c       ------------------------------------------------------------
DmelGr92a       ------------------------------------------------------------
DmelGr28a       ------------------------------------------------------------
DmelGr64b       ------------------------------------------------------------
TcasGr71        ------------------------------------------------------------
AgamGr19        ------------------------------------------------------------
AaegGr64b       ------------------------------------------------------------
DmelGr93d       ------------------------------------------------------------
TcasGr34        ------------------------------------------------------------
TcasGr43        ------------------------------------------------------------
TcasGr47        ------------------------------------------------------------
AmelGr3         ------------------------------------------------------------
AgamGr32b       ------------------------------------------------------------
AgamGr50        ------------------------------------------------------------
TcasGr20        ------------------------------------------------------------
TcasGr79        ------------------------------------------------------------
TcasGr15        ------------------------------------------------------------
TcasGr60        ------------------------------------------------------------
AgamGr38        ------------------------------------------------------------
TcasGr51        ------------MNMILKLSLKDIVFINPLVKYLNIFFITPWYDFPTNQKYYPSLAKCYA 48
DmelGr39b       ------------------------------------------------------------
AgamGr28        ------------------------------------------------------------
TcasGr61        ------------------------------------------------------------
TcasGr50        ------------------------------------------------------------
AgamGr12        ------------------------------------------------------------
AgamGr36        ------------------------------------------------------------
AgamGr48        ------------------------------------------------------------
AgamGr49a       ------------------------------------------------------------
AgamGr49b       ------------------------------------------------------------
DmelGr2a        ------------------------------------------------------------
DmelGr32a       ------------------------------------------------------------
DmelGr68a       ------------------------------------------------------------
AmelGr7         ------------------------------------------------------------
AmelGr8         ------------------------------------------------------------
AmelGr9         ------------------------------------------------------------
TcasGr57        ------------------------------------------------------------
TcasGr41        ------------------------------------------------------------
AgamGr34        ------------------------------------------------------------
AgamGr35        ------------------------------------------------------------
AgamGr39        ------------------------------------------------------------
AgamGr40        ------------------------------------------------------------
AaegGr28d       ------------------------------------------------------------
AgamGr42        ------------------------------------------------------------
AgamGr41        ------------------------------------------------------------
AgamGr43        ------------------------------------------------------------
DmelGr33a       ------------------------------------------------------------
AgamGr44        ------------------------------------------------------------
AgamGr13        ------------------------------------------------------------
AgamGr45        ------------------------------------------------------------
AgamGr46        ------------------------------------------------------------
Dmelr93a        ------------------------------------------------------------
AmelGr6         ------------------------------------------------------------
```

```
AmelGr10            ------------------------------------------------------------
TcasGr28            ------------------------------------------------------------
TcasGr40            ------------------------------------------------------------
AgamGr3             ------------------------------------------------------------
AgamGr4             ------------------------------------------------------------
AgamGr6             ------------------------------------------------------------
AgamGr7             ------------------------------------------------------------
AgamGr47            ------------------------------------------------------------
DmelGr8a            ------------------------------------------------------------
AgamGr5             ------------------------------------------------------------
AgamGr8             ------------------------------------------------------------
DmelGr9a            ------------------------------------------------------------
DmelGr59f           ------------------------------------------------------------
DmelGr77a           ------------------------------------------------------------
DmelGr85a           ------------------------------------------------------------
TcasGr27            ------------------------------------------------------------
TcasGr48            ------------------------------------------------------------
AgamGr1             ------------------------------------------------------------
DmelGr10a           ------------------------------------------------------------
DmelGr59e           ------------------------------------------------------------
DmelGr10b           ------------------------------------------------------------
DmelGr89a           ------------------------------------------------------------
DmelGr57a           ------------------------------------------------------------
DmelGr47b           ------------------------------------------------------------
DmelGr58a           ------------------------------------------------------------
DmelGr58b           ------------------------------------------------------------
DmelGr98a           ------------------------------------------------------------
AaegGr93a           ------------------------------------------------------------
TcasGr19            ------------------------------------------------------------
DmelGr47a           ------------------------------------------------------------
DmelGr58c           ------------------------------------------------------------


TcasGr38            ------------------------------------------------------------
TcasGr46            ------------------------------------------------------------
TcasGr1             ------------------------------------------------------------
TcasGr123           ------------------------------------------------------------
TcasGr62            IFMHIVPTKLISFYKSTAILEIAFEVIFIVTVWMTGAVKHSKIAHFLNKMIRLDERFQSV 120
TcasGr25            ------------------------------------------------------------
TcasGr59            ------------------------------------------------------------
AgamGr2             ------------------------------------------------------------
AaegGr66a           ------------------------------------------------------------
DmelGr66a           ------------------------------------------------------------
TcasGr11            ------------------------------------------------------------
TcasGr55            ------------------------------------------------------------
TcasGr21            ------------------------------------------------------------
TcasGr56            ------------------------------------------------------------
AaegGr              ------------------------------------------------------------
AaegGr28a           ------------------------------------------------------------
AgamGr33            ------------------------------------------------------------
DmelGr28bB          ------------------------------------------------------------
DmelGr28bC          ------------------------------------------------------------
DmelGr28bD          ------------------------------------------------------------
DmelGr28bA          ------------------------------------------------------------
DmelGr28bE          ------------------------------------------------------------
AgamGr25            ------------------------------------------------------------
AaegGr43a           ------------------------------------------------------------
DmelGr43a           ------------------------------------------------------------
AgamGr37b           ------------------------------------------------------------
AgamGr37d           ------------------------------------------------------------
AgamGr37a           ------------------------------------------------------------
AgamGr37c           ------------------------------------------------------------
AgamGr37e           ------------------------------------------------------------
AgamGr37f           ------------------------------------------------------------
AaegGr28c           ------------------------------------------------------------
AaegGr28b           ------------------------------------------------------------
AaegGr28e           ------------------------------------------------------------
TcasGr14            ------------------------------------------------------------
TcasGr49            ------------------------------------------------------------
AmelGr4             ------------------------------------------------------------
```

```
AmelGr5         ------------------------------------------------------------
TcasGr16        ------------------------------------------------------------
TcasGr22        ------------------------------------------------------------
TcasGr17        ------------------------------------------------------------
TcasGr150       ------------------------------------------------------------
TcasGr32        ------------------------------------------------------------
TcasGr54        ------------------------------------------------------------
TcasGr37        ------------------------------------------------------------
TcasGr53        ------------------------------------------------------------
TcasGr5         ------------------------------------------------------------
TcasGr12        ------------------------------------------------------------
TcasGr13        ------------------------------------------------------------
TcasGr104       ------------------------------------------------------------
TcasGr45        ------------------------------------------------------------
TcasGr98        ------------------------------------------------------------
TcasGr52        ------------------------------------------------------------
TcasGr105       ------------------------------------------------------------
TcasGr4         ------------------------------------------------------------
TcasGr44        ------------------------------------------------------------
AgamGr9a        ------------------------------------------------------------
AgamGr9c        ------------------------------------------------------------
AgamGr9b        ------------------------------------------------------------
AgamGr9d        ------------------------------------------------------------
AgamGr9e        ------------------------------------------------------------
AgamGr9f        ------------------------------------------------------------
AgamGr9g        ------------------------------------------------------------
AgamGr9i        ------------------------------------------------------------
AgamGr9h        ------------------------------------------------------------
AgamGr9j        ------------------------------------------------------------
AgamGr9l        ------------------------------------------------------------
AgamGr9k        ------------------------------------------------------------
AgamGr9m        ------------------------------------------------------------
AgamGr9n        ------------------------------------------------------------
AgamGr10        ------------------------------------------------------------
AgamGr11        ------------------------------------------------------------
DmelGr23aA      ------------------------------------------------------------
DmelGr23aB      ------------------------------------------------------------
DmelGr39aA      ------------------------------------------------------------
DmelGr39aB      ------------------------------------------------------------
DmelGr39aC      ------------------------------------------------------------
DmelGr39aD      ------------------------------------------------------------
DmelGr98c       ------------------------------------------------------------
DmelGr98d       ------------------------------------------------------------
DmelGr98b       ------------------------------------------------------------
AgamGr51        ------------------------------------------------------------
AgamGr52        ------------------------------------------------------------
DmelGr94a       ------------------------------------------------------------
DmelGr97a       ------------------------------------------------------------
DmelGr93b       ------------------------------------------------------------
DmelGr93c       ------------------------------------------------------------
DmelGr22d       ------------------------------------------------------------
DmelGr22e       ------------------------------------------------------------
DmelGr22a       ------------------------------------------------------------
DmelGr22b       ------------------------------------------------------------
DmelGr22c       ------------------------------------------------------------
DmelGr22f       ------------------------------------------------------------
DmelGr36a       ------------------------------------------------------------
DmelGr36c       ------------------------------------------------------------
DmelGr36b       ------------------------------------------------------------
DmelGr59d       ------------------------------------------------------------
DmelGr59a       ------------------------------------------------------------
DmelGr59b       ------------------------------------------------------------
AgamGr26        ------------------------------------------------------------
AgamGr27        ------------------------------------------------------------
AgamGr29        ------------------------------------------------------------
AgamGr31        ------------------------------------------------------------
AgamGr30        ------------------------------------------------------------
AgamGr32a       ------------------------------------------------------------
TcasGr2         ------------------------------------------------------------
TcasGr9         ------------------------------------------------------------
```

```
TcasGr7         ------------------------------------------------------------
TcasGr30        ------------------------------------------------------------
TcasGr29        ------------------------------------------------------------
AaegGr64f       ------------------------------------------------------------
AaegGr64a3      ------------------------------------------------------------
AgamGr14        ------------------------------------------------------------
AgamGr18        ------------------------------------------------------------
AaegGr64d       ------------------------------------------------------------
AgamGr17        ------------------------------------------------------------
AaegGr64c       ------------------------------------------------------------
DmelGr64c       ------------------------------------------------------------
DmelGr64d       ------------------------------------------------------------
AgamGr20        ------------------------------------------------------------
AaegGr61a       ------------------------------------------------------------
AgamGr21        ------------------------------------------------------------
AaegGr64a       ------------------------------------------------------------
DmelGr64a       ------------------------------------------------------------
DmelGr61a       ------------------------------------------------------------
AgamGr15        ------------------------------------------------------------
AaegGr1         ------------------------------------------------------------
DmelGr5a        ------------------------------------------------------------
DmelGr64f       ------------------------------------------------------------
DmelGr64e       ------------------------------------------------------------
AmelGr1         ------------------------------------------------------------
AmelGr2         ------------------------------------------------------------
AgamGr16        ------------------------------------------------------------
AaegGr64e       ------------------------------------------------------------
TCasGr6         ------------------------------------------------------------
TcasGr26        ------------------------------------------------------------
TcasGr33        ------------------------------------------------------------
AgamGr23        ------------------------------------------------------------
AaegGr21a       ------------------------------------------------------------
TcasGr10        ------------------------------------------------------------
TcasGr39        ------------------------------------------------------------
AgamGr22        ------------------------------------------------------------
AaegGr21b       ------------------------------------------------------------
DmelGr21a       ------------------------------------------------------------
AgamGr24        ------------------------------------------------------------
AaegGr63a       ------------------------------------------------------------
DmelGr63a       ------------------------------------------------------------
TcasGr3         ------------------------------------------------------------
DmelOr83b       ------------------------------------------------------------
AgamOr7         ------------------------------------------------------------
HvirOr2         ------------------------------------------------------------
TcasOr16        NRTSIFPHVTPHQPIPHPLFHPLVHDFGDTVSPPSTHVPLIPITSRVRRFHLTTYRCGCE 79
TcasOr22        NRTSIFPHVTPHQPIPHPLFHPLVHDFGDTVSPPSTHVPLIPITSRVRRFHLTTYRCGCE 91
AmelOr2         ------------------------------------------------------------
TcasGr31        ------------------------------------------------------------
TcasGr35        ------------------------------------------------------------
DmelGr59c       ------------------------------------------------------------
DmelGr92a       ------------------------------------------------------------
DmelGr28a       ------------------------------------------------------------
DmelGr64b       ------------------------------------------------------------
TcasGr71        ------------------------------------------------------------
AgamGr19        ------------------------------------------------------------
AaegGr64b       ------------------------------------------------------------
DmelGr93d       ------------------------------------------------------------
TcasGr34        ------------------------------------------------------------
TcasGr43        ------------------------------------------------------------
TcasGr47        ------------------------------------------------------------
AmelGr3         ------------------------------------------------------------
AgamGr32b       ------------------------------------------------------------
AgamGr50        ------------------------------------------------------------
TcasGr20        ------------------------------------------------------------
TcasGr79        ------------------------------------------------------------
TcasGr15        ------------------------------------------------------------
TcasGr60        ------------------------------------------------------------
AgamGr38        ------------------------------------------------------------
TcasGr51        CLLMVVKILWVIYWLQDDALKSVYASLLCTEKIFLFAIYTNLSILTTVTIFKSAFWDVDK 108
DmelGr39b       ------------------------------------------------------------
```

```
AgamGr28        ------------------------------------------------------------
TcasGr61        ------------------------------------------------------------
TcasGr50        ------------------------------------------------------------
AgamGr12        ------------------------------------------------------------
AgamGr36        ------------------------------------------------------------
AgamGr48        ------------------------------------------------------------
AgamGr49a       ------------------------------------------------------------
AgamGr49b       ------------------------------------------------------------
DmelGr2a        ------------------------------------------------------------
DmelGr32a       ------------------------------------------------------------
DmelGr68a       ------------------------------------------------------------
AmelGr7         ------------------------------------------------------------
AmelGr8         ------------------------------------------------------------
AmelGr9         ------------------------------------------------------------
TcasGr57        ------------------------------------------------------------
TcasGr41        ------------------------------------------------------------
AgamGr34        ------------------------------------------------------------
AgamGr35        ------------------------------------------------------------
AgamGr39        ------------------------------------------------------------
AgamGr40        ------------------------------------------------------------
AaegGr28d       ------------------------------------------------------------
AgamGr42        ------------------------------------------------------------
AgamGr41        ------------------------------------------------------------
AgamGr43        ------------------------------------------------------------
DmelGr33a       ------------------------------------------------------------
AgamGr44        ------------------------------------------------------------
AgamGr13        ------------------------------------------------------------
AgamGr45        ------------------------------------------------------------
AgamGr46        ------------------------------------------------------------
Dmelr93a        ------------------------------------------------------------
AmelGr6         ------------------------------------------------------------
AmelGr10        ------------------------------------------------------------
TcasGr28        ------------------------------------------------------------
TcasGr40        ------------------------------------------------------------
AgamGr3         ------------------------------------------------------------
AgamGr4         ------------------------------------------------------------
AgamGr6         ------------------------------------------------------------
AgamGr7         ------------------------------------------------------------
AgamGr47        ------------------------------------------------------------
DmelGr8a        ------------------------------------------------------------
AgamGr5         ------------------------------------------------------------
AgamGr8         ------------------------------------------------------------
DmelGr9a        ------------------------------------------------------------
DmelGr59f       ------------------------------------------------------------
DmelGr77a       ------------------------------------------------------------
DmelGr85a       ------------------------------------------------------------
TcasGr27        ------------------------------------------------------------
TcasGr48        ------------------------------------------------------------
AgamGr1         ------------------------------------------------------------
DmelGr10a       ------------------------------------------------------------
DmelGr59e       ------------------------------------------------------------
DmelGr10b       ------------------------------------------------------------
DmelGr89a       ------------------------------------------------------------
DmelGr57a       ------------------------------------------------------------
DmelGr47b       ------------------------------------------------------------
DmelGr58a       ------------------------------------------------------------
DmelGr58b       ------------------------------------------------------------
DmelGr98a       ------------------------------------------------------------
AaegGr93a       ------------------------------------------------------------
TcasGr19        ------------------------------------------------------------
DmelGr47a       ------------------------------------------------------------
DmelGr58c       ------------------------------------------------------------


TcasGr38        ------------------------------------------------------------
TcasGr46        ------------------------------------------------------------
TcasGr1         ------------------------------------------------------------
TcasGr123       ------------------------------------------------------------
TcasGr62        GLQIDTVREKRRIKIHFFLRSLFAIMLPLGILLISQDVIANVLLFIFIVVKSGVAFQTIE 180
TcasGr25        ------------------------------------------------------------
```

```
TcasGr59        ----------MWSENITDVLKHVWFLSKLFLLCPRSIDEKIKRQERHSFYKIYKYTYNIV 50
AgamGr2         ------------------------------------------------------------
AaegGr66a       -------MAAKVGSSLLESIKVLFYSSSILGIIPYSLRDFYSRTVLRVSVVGNIWVLLSV 53
DmelGr66a       --MDNMAQAEDAVQPLLQQFQQLFFISKIAGILPQDLEKFRSRNLLEKSRNGMIYMLSTL 58
TcasGr11        ------------------------------------------------------------
TcasGr55        ------------------------------------------------------------
TcasGr21        ------------------------------------------------------------
TcasGr56        ------------------------------------------------------------
AaegGr          ------------------------------------------------------------
AaegGr28a       ------------------------------------------------------------
AgamGr33        ------------------------------------------------------------
DmelGr28bB      ------------------------------------------------------------
DmelGr28bC      ------------------------------------------------------------
DmelGr28bD      ------------------------------------------------------------
DmelGr28bA      ------------------------------------------------------------
DmelGr28bE      ------------------------------------------------------------
AgamGr25        ------------------------------------------------------------
AaegGr43a       ------------------------------------------------------------
DmelGr43a       ------------------------------------------------------------
AgamGr37b       ------------------------------------------------------------
AgamGr37d       ------------------------------------------------------------
AgamGr37a       ------------------------------------------------------------
AgamGr37c       ------------------------------------------------------------
AgamGr37e       ------------------------------------------------------------
AgamGr37f       ------------------------------------------------------------
AaegGr28c       ------------------------------------------------------------
AaegGr28b       ------------------------------------------------------------
AaegGr28e       ------------------------------------------------------------
TcasGr14        ------------------------------------------------------------
TcasGr49        ------------------------------------------------------------
AmelGr4         ------------------------------------------------------------
AmelGr5         ------------------------------------------------------------
TcasGr16        ------------------------------------------------------------
TcasGr22        ------------------------------------------------------------
TcasGr17        ------------------------------------------------------------
TcasGr150       ------------------------------------------------------------
TcasGr32        ------------------------------------------------------------
TcasGr54        ------------------------------------------------------------
TcasGr37        ------------------------------------------------------------
TcasGr53        ------------------------------------------------------------
TcasGr5         ------------------------------------------------------------
TcasGr12        ------------------------------------------------------------
TcasGr13        ----------------------------MRFLGESPNGIYLYHDGRVKLLEGRRVVDCDD 32
TcasGr104       ------------------------------------------------------------
TcasGr45        ------------------------------------------------------------
TcasGr98        ------------------------------------------------------------
TcasGr52        ------------------------------------------------------------
TcasGr105       ------------------------------------------------------------
TcasGr4         ------------------------------------------------------------
TcasGr44        ------------------------------------------------------------
AgamGr9a        ------------------------------------------------------------
AgamGr9c        ------------------------------------------------------------
AgamGr9b        ------------------------------------------------------------
AgamGr9d        ------------------------------------------------------------
AgamGr9e        ------------------------------------------------------------
AgamGr9f        ------------------------------------------------------------
AgamGr9g        ------------------------------------------------------------
AgamGr9i        ------------------------------------------------------------
AgamGr9h        ------------------------------------------------------------
AgamGr9j        ------------------------------------------------------------
AgamGr9l        ------------------------------------------------------------
AgamGr9k        ------------------------------------------------------------
AgamGr9m        ------------------------------------------------------------
AgamGr9n        ------------------------------------------------------------
AgamGr10        ------------------------------------------------------------
AgamGr11        ------------------------------------------------------------
DmelGr23aA      ------------------------------------------------------------
DmelGr23aB      ------------------------------------------------------------
DmelGr39aA      ------------------------------------------------------------
DmelGr39aB      ------------------------------------------------------------
```

```
DmelGr39aC      ------------------------------------------------------------
DmelGr39aD      ------------------------------------------------------------
DmelGr98c       ------------------------------------------------------------
DmelGr98d       ------------------------------------------------------------
DmelGr98b       ------------------------------------------------------------
AgamGr51        ------------------------------------------------------------
AgamGr52        ------------------------------------------------------------
DmelGr94a       ------------------------------------------------------------
DmelGr97a       ------------------------------------------------------------
DmelGr93b       ------------------------------------------------------------
DmelGr93c       ------------------------------------------------------------
DmelGr22d       ------------------------------------------------------------
DmelGr22e       ------------------------------------------------------------
DmelGr22a       ------------------------------------------------------------
DmelGr22b       ------------------------------------------------------------
DmelGr22c       ------------------------------------------------------------
DmelGr22f       ------------------------------------------------------------
DmelGr36a       ------------------------------------------------------------
DmelGr36c       ------------------------------------------------------------
DmelGr36b       ------------------------------------------------------------
DmelGr59d       ------------------------------------------------------------
DmelGr59a       ------------------------------------------------------------
DmelGr59b       ------------------------------------------------------------
AgamGr26        ------------------------------------------------------------
AgamGr27        ------------------------------------------------------------
AgamGr29        ------------------------------------------------------------
AgamGr31        ------------------------------------------------------------
AgamGr30        ------------------------------------------------------------
AgamGr32a       ------------------------------------------------------------
TcasGr2         ------------------------------------------------------------
TcasGr9         ------------------------------------------------------------
TcasGr7         -------------------------------------------MRVQPSNTPPEKSQLST 17
TcasGr30        -------------------------------------------MRVQPSNTPPEKSQLST 17
TcasGr29        -------------------------------------------MRVQPSNTPPEKSQLST 17
AaegGr64f       ------------------------------------------------------------
AaegGr64a3      ------------------------------------------------------------
AgamGr14        -----------------------------------------------------------M 1
AgamGr18        ------------------------------------------------------------
AaegGr64d       ---------------------------------------------------------MKL 3
AgamGr17        ------------------------------------------------------------
AaegGr64c       ------------------------------------------------------------
DmelGr64c       ------------------------------------------------------------
DmelGr64d       ------------------------------------------------------------
AgamGr20        ----------------------------------------------MIEVGFGFRMVPGP 14
AaegGr61a       ----------------------------------------------MIDPLWRDRYIL-- 12
AgamGr21        -----------------------------------------MCVRSNPDKENRAPVLESV 19
AaegGr64a       ------------------------------------------------------------
DmelGr64a       -------------------------------------------MKGPNLNFRKTPSKDNG 17
DmelGr61a       -------------------------------------------------MSRTSDDIRKH 11
AgamGr15        -------------------------------MG----FAVSENHDTKPLIQPTWHTC-LK 24
AaegGr1         -------------------------------MARKLLPQVLEPRQTGKIVRFNGELVPVQ 29
DmelGr5a        -------------------------------------MRQLKGRNRCNRAVR-----HLK 18
DmelGr64f       ---------------------------MKILPKLERKLRRLKKRVTRTSLFRKLDLVHER 33
DmelGr64e       ---------------------------MARTTGDPAKRRRCMSRIKFWRRSRVGSEATLG 33
AmelGr1         ------------------------------------------------------------
AmelGr2         ---------------------------MHSEDQIQLMMLKTKDGLGEIPKGKGRGSNLKI 33
AgamGr16        ---------------------------------------------------MFPLLT--- 6
AaegGr64e       ------------------------------------------MPGNFGRNDRHVILVRSK 18
TCasGr6         ------------------------------------MEISD-----------LAQLYGNE 13
TcasGr26        ------------------------------------MEISD-----------LAQLYGNE 13
TcasGr33        ------------------------------------MEISD-----------LAQLYGNE 13
AgamGr23        ---MRWNGCRYRYKTDQTPQRRVEKNFIRRHCPTKTMVIKESEFDDSLGYALLRRDMGTV 57
AaegGr21a       ---MTVIAIKVVEKAN---YRTVDYHVLR-----KKMVIKDSEFEDSLNYALLRGDMGTT 49
TcasGr10        --------------------------------MRNDHGSNT------------HLHPDDA 16
TcasGr39        --------------------------------MRNDHGSNT------------HLHPDDA 16
AgamGr22        -----------------MIHTQMED--AQYEIRHQVLNPNQ------------RQQLEDR 29
AaegGr21b       -----------------MIHSQMED--SQYQIRQQILNPNQ------------RQQLEDN 29
DmelGr21a       -----------------MSFWAVSRGLTPPSKVVPMLNPNQ------------RQFLEDE 31
AgamGr24        ------------------------------------------------------------
AaegGr63a       --------------------MNLNQDPIQYINLNNNARTVFLDVKPIYNE-------EKR 33
```

```
DmelGr63a       ----MRPSGEKVVKGHGQGNSGHSLSGMANYYRRKKGDAVFLNAKPLNSANAQAYLYGVR 56
TcasGr3         -------------------MYHQDQAVSILGEAIPKRRSVFLES-------------GVN 28
DmelOr83b       -------------------------------------------MTTSMQPSKYTGLVADL 17
AgamOr7         ---------------------------------------------MQVQPTKYVGLVADL 15
HvirOr2         ----------------------------------------------MMTKVKAQGLVSDL 14
TcasOr16        RISSNSR---------------------------NTLSQYQILTNFQMMKFKVTGLVADL 112
TcasOr22        RISSNSRSVTNFQPLVRNTLSQYQILTNFQPLVRNTLSQYQILTNFQMMKFKVTGLVADL 151
AmelOr2         -----------------------------------------------MMKFKQQGLIADL 13
TcasGr31        ------------------------------------------------------------
TcasGr35        ------------------------------------------------------------
DmelGr59c       ------------------------------------------------------------
DmelGr92a       ------------------------------------------------------------
DmelGr28a       ------------------------------------------------------------
DmelGr64b       ------------------------------------------------------------
TcasGr71        ------------------------------------------------------------
AgamGr19        ------------------------------------------------------------
AaegGr64b       ------------------------------------------------------------
DmelGr93d       ------------------------------------------------------------
TcasGr34        ------------------------------------------------------------
TcasGr43        ------------------------------------------------------------
TcasGr47        ------------------------------------------------------------
AmelGr3         ------------------------------------------------------------
AgamGr32b       ------------------------------------------------------------
AgamGr50        ------------------------------------------------------------
TcasGr20        ------------------------------------------------------------
TcasGr79        ------------------------------------------------------------
TcasGr15        ------------------------------------------------------------
TcasGr60        ------------------------------------------------------------
AgamGr38        ------------------------------------------------------------
TcasGr51        WRTLFTNLQYIDINLQNKGKKESKLMKNFYFWFVLKQVMFLCYSTYGIQVFGTMQRTSFF 168
DmelGr39b       ------------------------------------------------------------
AgamGr28        ------------------------------------------------------------
TcasGr61        -------------------MTKSTVYDTVSWLILLSLLLGLYPSYLQTVGKCQRLKTDKN 41
TcasGr50        ------------------------------------------------------------
AgamGr12        ------------------------------------------------------------
AgamGr36        ------------------------------------------------------------
AgamGr48        ------------------------------------------------------------
AgamGr49a       ------------------------------------------------------------
AgamGr49b       ------------------------------------------------------------
DmelGr2a        ------------------------------------------------------------
DmelGr32a       ----------------------------------------------------MSPNTWVI 8
DmelGr68a       ------------------------------------------------------------
AmelGr7         ------------------------------------------------------------
AmelGr8         ------------------------------------------------------------
AmelGr9         ------------------------------------------------------------
TcasGr57        ------------------------------------------------------------
TcasGr41        ------------------------------------------------------------
AgamGr34        ------------------------------------------------------------
AgamGr35        ------------------------------------------------------------
AgamGr39        ------------------------------------------------------------
AgamGr40        ------------------------------------------------------------
AaegGr28d       ------------------------------------------------------------
AgamGr42        ------------------------------------------------------------
AgamGr41        ------------------------------------------------------------
AgamGr43        ------------------------------------------------------------
DmelGr33a       ------------------------------------------------------------
AgamGr44        ------------------------------------------------------------
AgamGr13        ------------------------------------------------------------
AgamGr45        ------------------------------------------------------------
AgamGr46        ------------------------------------------------------------
Dmelr93a        ------------------------------------------------------------
AmelGr6         --------------------------------------------------MMKSIRKELA 10
AmelGr10        ------------------------------------------------------------
TcasGr28        ------------------------------------------------------------
TcasGr40        ------------------------------------------------------------
AgamGr3         ------------------------------------------------------------
AgamGr4         ------------------------------------------------------------
AgamGr6         ------------------------------------------------------------
AgamGr7         ------------------------------------------------------------
AgamGr47        ------------------------------------------------------------
```

```
DmelGr8a        ------------------------------------------------------------
AgamGr5         ------------------------------------------------------------
AgamGr8         ------------------------------------------------------------
DmelGr9a        ------------------------------------------------------------
DmelGr59f       ------------------------------------------------------------
DmelGr77a       ------------------------------------------------------------
DmelGr85a       ------------------------------------------------------------
TcasGr27        ------------------------------------------------------------
TcasGr48        ------------------------------------------------------------
AgamGr1         ------------------------------------------------------------
DmelGr10a       ------------------------------------------------------------
DmelGr59e       ------------------------------------------------------------
DmelGr10b       ------------------------------------------------------------
DmelGr89a       ------------------------------------------------------------
DmelGr57a       ------------------------------------------------------------
DmelGr47b       ------------------------------------------------------------
DmelGr58a       ------------------------------------------------------------
DmelGr58b       ------------------------------------------------------------
DmelGr98a       ------------------------------------------------------------
AaegGr93a       ------------------------------------------------------------
TcasGr19        ------------------------------------------------------------
DmelGr47a       ------------------------------------------------------------
DmelGr58c       ------------------------------------------------------------


TcasGr38        ------------------------MNNKLNMVLVKSMLQRIKMEEKLVVEKFLNSLQIYL 36
TcasGr46        ------------------------------------------MEEKLVVEKFLNSLQIYL 18
TcasGr1         -------------------------------------------MQHEVVKKFLHSVRIVF 17
TcasGr123       -----------------------------------------MSDFQKLYQKFVNSIRILL 19
TcasGr62        FVSIIRNRFVILNRYIEESISKYKHAELVMPLCKSCDLHHRLSKLIKQLNATYGLILLLM 240
TcasGr25        ------------------------------------------------------------
TcasGr59        AVSLTAYMVYITTNLR----ITQKLSIVTQLADLIFSASANASGVATVFFCLFYQNKLIE 106
AgamGr2         --------------------------------------MEPFMMAVDMVAGMINQKRLAQ 22
AaegGr66a       VSYSVSYHMVTDAYVG---VGGGGQGTLTNAIGIFIIYMEPLMMCIDMLAAMLNQKRLIE 110
DmelGr66a       ILYVVLYNILIYSFGEEDRSLKASQSTLTFVIGLFLTYIGLIMMVSDQLTALRNQGRIGE 118
TcasGr11        ------------------------MIKFSKVKSYFTTHHDTLHNIYTSMKPVYAICKLIG 36
TcasGr55        ------------------------MIKFSKVKSYFTTHHDTLHNIYTSMKPVYAICKLIG 36
TcasGr21        -----------------------------MLPKQFLKLFKDPCDVYTAIHPLFYVCTFFG 31
TcasGr56        -----------------------------MLPKQFLKLFKDPCDVYTAIHPLFYVCTFFG 31
AaegGr          -----------------------------MASSHWFRDCINPKNFYAAQAPVLKATFIGG 31
AaegGr28a       ------------------------------------------------------------
AgamGr33        -------------------------------MRKCLRTFLNPKDFYAAQRPVLRVSFLVG 29
DmelGr28bB      -----------------------------MSALRRVRKYFISSQVYEALRPLFFLTFLYG 31
DmelGr28bC      ----MDIEMAKEPVNPTDTPDIEVTPGLCQPLRRRFRRFVTAKQLYECLRPVFHVTYIHG 56
DmelGr28bD      -------------------------------MSFYFCEIFKPRDAFGAEQTLLLYTYLLG 29
DmelGr28bA      --------------------MIRCGLDIFRGCRGRFRYWLSARDCYDSISLMVAIAFALG 40
DmelGr28bE      ---------------------------MWLLRRSVGKSGNRPHDVYTCYRLTIFMALCLG 33
AgamGr25        ------------------------------------------MEISESTKVVFYVSRIFG 18
AaegGr43a       ------------------------------------------MEISESTKVVFFVSRIFG 18
DmelGr43a       ------------------------------------------MEISQPSIGIFYISKVLA 18
AgamGr37b       ---------------------------MMDSCLLVQ-------NVHDTFRPFYYLLKVTG 26
AgamGr37d       ----------------------------MR-WFSVT-------SVYETVQPFMFLLGCVG 24
AgamGr37a       ---------------------------MYRRSVAVVMLLNESLNVYSSMEVLYWILRLFG 33
AgamGr37c       -------------------------MSLFGE------------DALVAYETFSVLLKYLG 23
AgamGr37e       -------------------------MLTSNE------------DALKAFECFTLVVKCLG 23
AgamGr37f       --------------------------MVYKVLQKFN-----TSSSIEAFQPFLLLLKCLG 29
AaegGr28c       ---------------------------MSSAK----------VTVFETYSSVIVLLKCVG 23
AaegGr28b       --------------------------MALQWLKGHLRALSSSDSIYDVVYLNYLLSKIFG 34
AaegGr28e       --------------------------MALQWLKGHLRALSSSDSIYDVVYLNYLLSKIFG 34
TcasGr14        ------------------------------------------------------------
TcasGr49        -----------------------MCQIKNKLLLLDSSTQPNYLILSNKNNTYLVLNKYLL 37
AmelGr4         --------------------------------------MVDKLYWSRNMHLAIMLFLFFF 22
AmelGr5         --------------------------------------MVDKSYWSRNMDRLTLLFFFFF 22
TcasGr16        ---------------------------------------MPQVKLHKHYVPIILLSKILG 21
TcasGr22        ------------------------------------------------------------
TcasGr17        ------------------------------------------MNTFKIFRPIKILAEIFG 18
TcasGr150       ------------------------------------------MNTFKIFRPIKILAEIFG 18
TcasGr32        -------------------------------------------MSFKLLRLVLKVGHFFA 17
TcasGr54        -------------------------------------------MSFKLLRLVLKVGHFFA 17
TcasGr37        -----------------------------------------------MLKWVLKVGNVLA 13
```

```
TcasGr53        -----------------------------------------------MLKWVLKVGNVLA 13
TcasGr5         -------------------------------------------MTIQLLSLICKFGGWMG 17
TcasGr12        ------------------------------------------MSNLKKLKFLLKLGQLLG 18
TcasGr13        VSSVSQSEAGDEDRFSFTSSMVQSLDGNSSDLENSSEENCVSMSNLKKLKFLLKLGQLLG 92
TcasGr104       -----------------------------------MCYFNYSKKDIRSLSFFYKICNIFG 25
TcasGr45        -----------------------------------MCYFNYSKKDIRSLSFFYKICNIFG 25
TcasGr98        ------------------------------------MSYRLSRNDIRFLKVMYKLSHFLS 24
TcasGr52        ------------------------------------------------------------
TcasGr105       --------------------------------------MYFSRRDIRFLRPIFAVCRLFA 22
TcasGr4         --------------------------------------MVRPELDLNSLKCITKVLTILG 22
TcasGr44        ----------------------------------MTRAMVRPELDLNSLKCITKVLTILG 26
AgamGr9a        ------------------------------------------MHVLSNLRMLIRCLQFVG 18
AgamGr9c        --------------------------------------------MFENIHALLRSLQWVG 16
AgamGr9b        MINRVERGWEMVECITLVSLSLENGAHVRWRCKSFGKVVYITMHVFDNIHVLLRSLQFLG 60
AgamGr9d        ------------------------------------------MDVFDNIRGLIRCLRLLG 18
AgamGr9e        ---------------------------------------------MKWVQTILNNLSTVG 15
AgamGr9f        ---------------------------------------------MECLQIMFQSLSALG 15
AgamGr9g        ---------------------------------------------MWCLRIHLQCMEKLG 15
AgamGr9i        ---------------------------------------------MRCIRVQLAYMGRLG 15
AgamGr9h        ---------------------------------------MAASSFAELIRPMINTACTLG 21
AgamGr9j        -MLMQPVDIRPFSISLINLLPSISRIGPCVWLPQFCLLFNRMSNLLKRYRLLLAVASIGY 59
AgamGr9l        -MLEH---LFWYTFLSIILWYSFS----FFWK---CTTVDKMSSLVKRYQRLFAVATVMY 49
AgamGr9k        -----------MSWLKSPLYGQYQNDIRIVTFYHQTDTVDIMSMYIRQNHFTLRIVTAIY 49
AgamGr9m        ------------MWFISFYHEAYT---TFVIRGKAVVKSSTMSACARLNRYTFKIMSSVY 45
AgamGr9n        -----------------------------------------MSSLYRQHSRIFQISRLLY 19
AgamGr10        -----------------------------------------MSSLYRQHSRIFQISRLLY 19
AgamGr11        -----------------------------------------MSSYYRRHARIFQAAALFY 19
DmelGr23aA      -----------------------------------------MKTLECLTRRFLEVIFSVL 19
DmelGr23aB      ---------------------------------------MFPPTRVQASSRVVLKIFHFI 21
DmelGr39aA      -----------------------------------------MGTRNRKLLFFLHYQRYLG 19
DmelGr39aB      -----------------------------------------MDFQPGELCAYYRLCRYLG 19
DmelGr39aC      ----------------------------------------MKRNAFEELRVQLRTLKWLG 20
DmelGr39aD      -----------------------------------------MSKVCRDLRIYLRLLHIMG 19
DmelGr98c       -----------------------------------MEMEAKRSRLLTTARPYLQVLSLFG 25
DmelGr98d       -------------------------------------MEANRSRLLAAARPYIQIYSIFG 23
DmelGr98b       -------------------------------------MVAQKSRLLARAFPYLDIFSVFA 23
AgamGr51        --------------MCVCVCVCVWAVSPVRWSVRRFSCCTMQVPRWLIVLQSVVLKFLFA 46
AgamGr52        ---------------------MDYTDKSCVPSRDATLSVAMNVSKWSERLERCVWSFLLA 39
DmelGr94a       --------------------------------MDFTSDYAHRRMVKFLTIILIGFMTVFG 28
DmelGr97a       ------------MRFLRRQTRRLRSIWQRSLPVRFRRGKLHTQLVTICLYATVFLNILYG 48
DmelGr93b       -------------------MVYGFTMSGLLVMPRILRCLNVSRISAILLRSCFLYGTFFG 41
DmelGr93c       -------------------------------MIERLKKVSLPALSAFILFCSCHYGRILG 29
DmelGr22d       ------------------------------MFRPR-CGLRQK-FVYVILKSILYSSWLLG 28
DmelGr22e       ------------------------------MFRPSGSGYRQK-WTGLTLKGALYGSWILG 29
DmelGr22a       ------------------------------MSQPKRIHRICKGLARFTIRATLYGSWVLG 30
DmelGr22b       ------------------------------MFGSS-REIRPY-LARQMLKTTLYGSWLLG 28
DmelGr22c       ------------------------------MFASR-SDLQSR-LCWIILKATLYSSWFLG 28
DmelGr22f       ----------------------------MKMFQPR-RGFSCH-LAWFMLQTTLYASWLLG 30
DmelGr36a       ----------------------------------------MFDWVGLLLKVLYYYGQIIG 20
DmelGr36c       -----------------------------------------MDLESFLLGAVYYYGLFIG 19
DmelGr36b       ----------------------------------------MVDWVVLLLKAVHIYCYLIG 20
DmelGr59d       ----------------------------------------MADLLKLCLRIAYAYGRLTG 20
DmelGr59a       --------------------------------------------MKRIGQAYNVYAVFIG 16
DmelGr59b       -------------------------------------------MVYWMIKLYFRYSLAIG 17
AgamGr26        -----------------------------MVLELLSNRHLFRNEFLHSAHRLLRVCQVFG 31
AgamGr27        -----------------------------MAFATAAIQSFLQLNVKRSLYRLLTVGQVFG 31
AgamGr29        -----------------------------MSRIQTENRAHFRAILMDTFQPAIFLSQWSC 31
AgamGr31        -----------------------------MHRLTTEKCAYFRNLLVDTCRLAIRLSQWCC 31
AgamGr30        ------------------------------MCLVELDAAFYQYLLENQFRPLLNVAQCFG 30
AgamGr32a       ------------------------------MDPIKLDTTFYHKLLVKKFNLLLNIAQLAG 30
TcasGr2         MEKSTKIFPVPGD-----------------------------KNLHKSYSKMLILAQIFG 31
TcasGr9         MEKSTKIFPVPGD-----------------------------KNLHKSYSKMLILAQIFG 31
TcasGr7         ADTANLINKSLGK-----------------------------HNLQKSFAKVVILAQIFG 48
TcasGr30        ADTANLINKSLGK-----------------------------HNLQKSFAKVVILAQIFG 48
TcasGr29        ADTANLINKSLGK-----------------------------HNLQKSFAKVVILAQIFG 48
AaegGr64f       ------------------------------------------------------------
AaegGr64a3      ------------------------------------------------------------
AgamGr14        LNMTPPEVTPVKRFPGVSRPPSTPSTALAEETPPADGAAERECSTHEAVAAVIFMGQLFS 61
AgamGr18        -----------MKIASSLLLFYFFLP----------------ITFNIFRAKVLLLGQCIT 33
AaegGr64d       LTLTSRRAATAVAIGKESFFPNFHLPRWSKWIGRLKGKSTFDGSFHQAVAPVLFVGQCFT 63
```

```
AgamGr17       ---------MESP---------------------------AIDDFHRAVRPFLLISQLFS 24
AaegGr64c      ---------MQAPNQHCLAQLRKWHRHQFPSDNAVMDSNSKIDDFWGAVRPIIFVAQLFT 51
DmelGr64c      ----------------------------MQQSGQKGTR----NTLQHAIGPVLVIAQFFG 28
DmelGr64d      -----------------------MLRSHLSVHGLQMERSVQENTLHYTIGHVLIIARIFG 37
AgamGr20       VRSSNATRPTRSRWHWLRKRGNVTFVR---------------PAAEIDDRKERFYLAIAS 59
AaegGr61a      WKNSKILQPDEEPVNNEENVSEYDFFH---------------IAIAPVLR----FSQLFG 53
AgamGr21       RTLATSMCSSVTTTAMKLAAKGYTLRQRVRQKLCPKIVDDDRDTFLRGIRPVVILGQMFG 79
AaegGr64a      ------------------------------------------------------------
DmelGr64a      VKQVESLARPETPPPKFVEDSNLEFNVLASEKLPN---YTNLDLFHRAVFPFMFLAQCVA 74
DmelGr61a      LKVRRQKQRAILAMRWRCAQGGLEF--------------EQLDTFYGAIRPYLCVAQFFG 57
AgamGr15       RWC-ASWLHWPR---------VQRR--------ASREDWLFNGTFHEASRGVLMMAQLFS 66
AaegGr1        SKS-RAFLKHFK---------YPKR--------ATRENWIHDGSFHDAVSGLLITAQLFS 71
DmelGr5a       VQG-KMWLKNLKSGLEQIR-ESQVR--------GTRKNFLHDGSFHEAVAPVLAVAQCFC 68
DmelGr64f      ARK-KAFQESCETYKNQIENEYEIRNSLPKLSRSDKEAFLSDGSFHQAVGRVLLVAEFFA 92
DmelGr64e      IIKYRVVEKDTKRFKLSLIKAWLLR--------IRQEDYKYSGSFQEAIKPVLIIAQIFA 85
AmelGr1        ------------------------------------------------MRPIIMLAQFFS 12
AmelGr2        WSSVMYHKDDNNIEDISANQENDLSTKRPRAERNYFRNSEALENFHCAIGPVLKAAQIFG 93
AgamGr16       ----EDFYHPPNR--------------------PRFINGG--GYFCRSIRPVLIVGQMFG 40
AaegGr64e      VGDSSSFEREFYR--------------------QRQVNLDQEGSYLAVFRPVIILGQVFA 58
TCasGr6        LHIKQISKWLRG------SARAQEIQKRSELDSKDGHVIDEHDQFFRDHKLLLVLFRVLG 67
TcasGr26       LHIKQISKWLRG------SARAQEIQKRSELDSKDGHVIDEHDQFFRDHKLLLVLFRVLG 67
TcasGr33       LHIKQISKWLRG------SARAQEIQKRSELDSKDGHVIDEHDQFFRDHKLLLVLFRVLG 67
AgamGr23       WDTAKDERMVNGTMDPELIQRAKERAVRAQLNSADGDTCETHDQFYRDHKLLLVLFRGLA 117
AaegGr21a      WDINKDERMMNGTLDPELIQRAKERAIRAQLNSADGDTCELHDQFYRDHKLLLVLFRALA 109
TcasGr10       IRRAKIVKVAASPT-----SANPDE-------EPDPELLDRYDNFYQTTKSLLVLFQIMG 64
TcasGr39       IRRAKIVKVAASPT-----SANPDE-------EPDPELLDRYDNFYQTTKSLLVLFQIMG 64
AgamGr22       RRIKEQLHQLEQDN-----ESPTHMYRRKLKIASDVNLLDQHDSFYHTTKSLLVLFQIMG 84
AaegGr21b      RRIKEQMQQLQRDD-----ASPSHMYIRKLEFQADVNLLDKHDSFYHTTKSLLVLFQIMG 84
DmelGr21a      VRYREKLKLMARG------DAMEEVYVRKQETVDDPLELDKHDSFYQTTKSLLVLFQIMG 85
AgamGr24       -------------------------------------------MVFESSKPIYLVLRAIG 17
AaegGr63a      KVSNGFNNRIG---------FPPISSRRVFGLESEFN--TRSDIVYGTTKPIYNVLRMLG 82
DmelGr63a      KYSIGLAERLDADY-----EAPPLDRKKSSDSTASNNPEFKPSVFYRNIDPINWFLRIIG 111
TcasGr3        SADSFKASKVG--------PAPPIKFINKSSTDKFGN-----GAIYEVLKPIYALMRIVG 75
DmelOr83b      MPNIRAMKYSGLFMHNFTGG----SAFMKKVYSSVHLVFLLMQFTFILVNMALNAEEVNE 73
AgamOr7        MPNIRLMQASGHFLFRYVTG----PILIRKVYSWWTLAMVLIQFFAILGNLATNADDVNE 71
HvirOr2        MPNIKLMQMAGHFLFNYHSENAGMSNLLRKIYASTHAILIFIHYACMGINMAKYSDEVNE 74
TcasOr16       MPNIRLIQASGHFMLNYHADNSGALHTLRLGYCCMHLVFVLVQYGCNFVNLVLERGDVND 172
TcasOr22       MPNIRLIQASGHFMLNYHADNSGALHTLRLGYCCMHLVFVLVQYGCNFVNLVLERGDVND 211
AmelOr2        MPNINLMKATGHFMFNYYTDSS-TKHIHKI-YCIVHLVLILMQFGFCGINLMMESEDVDD 71
TcasGr31       ------------------------------------------------------------
TcasGr35       ------------------------------------------------------------
DmelGr59c      --------------------------------------------MVDLVKTILLIAYWYG 16
DmelGr92a      -------------------------------MFEFLHQMSAPKLSTSILRYIFRYAQFIG 29
DmelGr28a      -------------------------------MAFKLWERFSQADNVFQALRPLTFISLLG 29
DmelGr64b      --------------------------------------MPQGETFHRAVSNVLFISQIYG 22
TcasGr71       ------------------------------------------MTDHNLLRLIVTIGEFFA 18
AgamGr19       ------------------------------------------MNFHNTIRPILVIFLLFG 18
AaegGr64b      ---------------------------------------MATAEMRRIARTGINFS---N 18
DmelGr93d      --------------------------------------MKATKYSVGILRFMSFYARFLS 22
TcasGr34       -------------------------------------------MSSQLLKLTHDALKILG 17
TcasGr43       -------------------------------------------MDFQLLQIMFTIGKFFA 17
TcasGr47       -------------------------------------------MDLEVLEIYFTVANKFS 17
AmelGr3        ---------------------------MEVKRVEEKRKILFNNELCQAIFPIYYLGKFCG 33
AgamGr32b      ------------------------------MAWNDTEIDRLRHQLRQNISPTVRLSQCLS 30
AgamGr50       --------------------------------------MARKGVLTIGERFCIWLLMVGG 22
TcasGr20       ----------------------------------MTPHALISSDFHDVIKPLINLTKIFG 26
TcasGr79       -------------------------------------------MNSIPIETIFKFGKYTL 17
TcasGr15       -------------------------------------------MDIKSIKTCFYVAKFFA 17
TcasGr60       ---------------------------------------------MILVLLVIVNLGLNG 15
AgamGr38       ------------------------------MKAKSGNRELFESLLRFPKRDVFDCVPLHY 30
TcasGr51       KTFLNSCLMDVYYEFLLISLINILIRSFKSRYKDLNQKLETACEQSKSVEELMNIANYYR 228
DmelGr39b      --------------------------------------------MLYSFHPYLKYFALLG 16
AgamGr28       ------------------------------MFEGRDVQHLRKLQLYQSFQLYLRCAQLLS 30
TcasGr61       CTIVIIFHLILFAVLLYFASLEKNPLFDGQLYSFNTFAKLLIFVVSLSGNSAVFIMLIIS 101
TcasGr50       ------------------------------------------MSLYSSINSLVLISKIFA 18
AgamGr12       -----------------------------------------MARLCAKLRVLFFVSNVLY 19
AgamGr36       ----------------------------------MGCSYKNPRHVYDVLRPLLAFSKYFG 26
AgamGr48       ------------------------------------------MMVRYGLRALYYFHSILG 18
AgamGr49a      ----------------------------MQNSSRMMSFINYPRSIFNQLNHSRTQCDSAA 32
AgamGr49b      --------------------------------------MLYGYLTLLVLRLHFHTAQCIG 22
```

```
DmelGr2a        --------------------------------------MEFGMDTLRALEPLHRACQVCN 22
DmelGr32a       EMPTQKTRSHPYPRRISPYRPPVLNRDAFSRDAPPMPARNHDHPVFEDIRTILSVLKASG 68
DmelGr68a       ------------------------------------------MKIYQDIYPISKPSQIFA 18
AmelGr7         --------------------------------------MYEPKTLKETIKPLMVVNFVFG 22
AmelGr8         -------------------------------MIDRLKCNGKRVKNCKRQSSALCFGNYAS 29
AmelGr9         -------------------------MWLEKLKKHKIWKIFKATDFNSLMFPCFFICGIIG 35
TcasGr57        ---------------------------------------------MKEVPGWLTPPHSLG 15
TcasGr41        ------------------------------------MIEVGKTRVKRASNQLFRFGQFCA 24
AgamGr34        -----------------------------------MKNKVSLDSIISIVEMNVKFYRLLG 25
AgamGr35        ----------MSKWINKRKVSILNLAPPSKPGPALQATPVGEQEFEQLFHFAFKCFRLFA 50
AgamGr39        -----------------------------------MSRLFEAKSFVQTLRILYYIAKTFG 25
AgamGr40        ------------------------------------MKWFSVDSVYDSVRPVYRAGKLIG 24
AaegGr28d       ------------------------------------MRWFKVHNYFESLRPLYLAIKLLH 24
AgamGr42        -------------------------------MSTRLKYWFNIGSFFETMKPNIRVLKLFG 29
AgamGr41        ----------------------MDVFGLENSRELVVSWWPKINDVVHAVSPIWNTSRAFG 38
AgamGr43        ----------------------------------MCFVLFILPYSMLMLIAVFSFIQIIG 26
DmelGr33a       ------MIQIMNWFSMVIGLIPLNRQQSETNFILDYAMMCIVPIFYVACYLLINLSHIIG 54
AgamGr44        --------------------------------------MLNDKVVNGLLLFSTTIATLLG 22
AgamGr13        ------------------------------------------MLLLFSLYFNLLLSYAAG 18
AgamGr45        --------------MPRRKVFAWSISLQNAIIPLYIISKVGCLNAFTYLPLFHFLDTYAG 46
AgamGr46        ----------------------MNGINTGKPPSAASPPDYSRQQFLRCFRPLHILSSTFA 38
Dmelr93a        ------------------------------MFSSSSAMTGKRAESWSRLLLLWLYRCARG 30
AmelGr6         RYPAKRVLPLTKPRLKPFDVETTFAEVTLARKEHTRKYHGPDSLLYSAIYPVVCVMKVFG 70
AmelGr10        ------------------------------------------MVLVGDTNLIFNAGLILG 18
TcasGr28        --------------------------------------MRLIRKMKSDLVLTIEASDAVL 22
TcasGr40        ---------------------MVFAELWKKIRNRGTPPKFELPTTHNCLKKVLLLSQIVG 39
AgamGr3         ---------------------------------MASVRKLFILVTFGAFSWLLLLFQLFG 27
AgamGr4         --------MQFSHQHSSDVHFIVSRERSSPSTCTMNLVLPLLPNGQQLLSVAFGIFKCFG 52
AgamGr6         --------MFNSFSKSSLEFILTETQCTTMEQFSASSMSRHHAPYDRRFGSIYPLFQLLG 52
AgamGr7         --------------------MLVTLIFQLCALQTYASTDAPLGSSLALLWCIGNCILYVG 40
AgamGr47        -------------------------MSVMESNEKFYHPQKTKTGVVIVTINVVQYQKKWD 35
DmelGr8a        ----------------------------------------MSGHLGRVLQFHLRLYQVLG 20
AgamGr5         ---------MSRINEIMKIAQPIRDNYFARSPPWSKGGRFRVGTYLPNLDRARLVFSVRD 51
AgamGr8         ---------------------------------------------MECFSKVIVLYQILA 15
DmelGr9a        -------------------------------------------MSLWLEHFLTGYFQLCG 17
DmelGr59f       -------------------MRSSATKGAKLKNSPRERLSSFNPQYAERYKELYRTLFWLL 41
DmelGr77a       ------------MPLPLGDPLALAVSPQLGYIRITAMPRWLQLPGMSALGILYSLTRVFG 48
DmelGr85a       ----------------------MYSLIEAQLLGGKLVNRVMASLRRIIQRSLGYFCALNG 38
TcasGr27        ---------------------------------------MSHSNPLEAFKLNTFFLKALT 21
TcasGr48        --------------------------MHTLQHFTIDTRSTHSMKNFKYLKVLVTFAHFIC 34
AgamGr1         -----------------------------------MIGLKKHRAFLERLRWTLRAQHVFG 25
DmelGr10a       ------------------------------MTSPDERKSFWERHEFKFYRYGHVYALIYG 30
DmelGr59e       ------------------------------------------MDSSYWENLLLTINRFLG 18
DmelGr10b       --------------------------------------MRVGKLCRLALRFWMGLILVLG 22
DmelGr89a       ---------------------------------------MLRFPHVCGLCLLLKYWQILA 21
DmelGr57a       --------------------------------MAVLYFFREPETVFDCAAFICILQFLMG 28
DmelGr47b       --------------------------------------MQRDDGFVYCYGNLYSLLLYWG 22
DmelGr58a       ----------------------------------------------MLLKFMYIYGIGCG 14
DmelGr58b       ---------------------------------------MLHPKLGRVMNVVYYHSVVFA 21
DmelGr98a       ------------------------------------MEQMSGELHAASLLYMRRLMKCLG 24
AaegGr93a       ------------------------------------MVQSLQILWQFNLAVLHLMESLFG 24
TcasGr19        -----------------------------------MTSKARTFTIKCPVKKRKCTVSNVS 25
DmelGr47a       -------------------------------------------MAFTSSQLCSLLTKFTA 17
DmelGr58c       ------------------------------------------MNQYFLLHTYFQVSRLIG 18


TcasGr38        QHN------------------------------------------------------QIF 42
TcasGr46        QHN------------------------------------------------------QIF 24
TcasGr1         VQS------------------------------------------------------EIF 23
TcasGr123       FQS------------------------------------------------------RIF 25
TcasGr62        FTSHFIFIVVSIFYMSAYLISNPIMWDRVMLLTFWSAFFIVNVLYICNQCYNTVEESRIF 300
TcasGr25        ------------------------------------------------------------
TcasGr59        VISK--------------------------------------------------LHKLDN 116
AgamGr2         CFDR--------------------------------------------------LARLDN 32
AaegGr66a       CVER--------------------------------------------------LDKVDL 120
DmelGr66a       LYER--------------------------------------------------IRLVDE 128
TcasGr11        LNTLR---------------------------------------------------IGR- 44
TcasGr55        LNTLR---------------------------------------------------IGR- 44
TcasGr21        LAPYS---------------------------------------------------LVR- 39
TcasGr56        LAPYS---------------------------------------------------LVR- 39
```

```
AaegGr          LTPFT---------------------------------------------------VVN- 39
AaegGr28a       ------------------------------------------------------------
AgamGr33        MTPFT---------------------------------------------------VVK- 37
DmelGr28bB      LTPFH---------------------------------------------------VVR- 39
DmelGr28bC      LTSFY---------------------------------------------------ISCD 65
DmelGr28bD      LTPFR---------------------------------------------------LRG- 37
DmelGr28bA      ITPFL---------------------------------------------------VRR- 48
DmelGr28bE      IVPYY---------------------------------------------------VSI- 41
AgamGr25        LAPYV---------------------------------------------------VKRT 27
AaegGr43a       LAPYV---------------------------------------------------VKRT 27
DmelGr43a       LAPYA---------------------------------------------------TVRN 27
AgamGr37b       LAPF-----------------------------------------------------RLN 33
AgamGr37d       LAPFG---------------------------------------------------YRLG 33
AgamGr37a       YAPFR---------------------------------------------------LQLD 42
AgamGr37c       VNPPI---------------------------------------------------SCDR 32
AgamGr37e       LYPPV---------------------------------------------------TCSR 32
AgamGr37f       LTPFF---------------------------------------------------SNAS 38
AaegGr28c       LHPPL---------------------------------------------------TNPK 32
AaegGr28b       MFIYT---------------------------------------------------VTEN 43
AaegGr28e       MFIYT---------------------------------------------------VTDN 43
TcasGr14        ---------------------------------------------------------MPL 3
TcasGr49        VKHSK--------------------------------------------------PKMPL 47
AmelGr4         KFFGL--------------------------------------------------ATFSL 32
AmelGr5         KFAGL--------------------------------------------------ATFSL 32
TcasGr16        IAPIS--------------------------------------------------YKNSN 31
TcasGr22        ------------------------------------------------------------
TcasGr17        LSPMT--------------------------------------------------QKLTL 28
TcasGr150       LSPMT--------------------------------------------------QKLTL 28
TcasGr32        ITPGF------------------------------------------------------- 22
TcasGr54        ITPGF------------------------------------------------------- 22
TcasGr37        MTP--------------------------------------------------------- 16
TcasGr53        MTP--------------------------------------------------------- 16
TcasGr5         LTP-V------------------------------------------------------- 21
TcasGr12        LAP--------------------------------------------------------- 21
TcasGr13        LAP--------------------------------------------------------- 95
TcasGr104       IVPPY------------------------------------------------------- 30
TcasGr45        IVPPY------------------------------------------------------- 30
TcasGr98        ITPNY------------------------------------------------------- 29
TcasGr52        MQDSF------------------------------------------------------- 5
TcasGr105       LVPYY------------------------------------------------------- 27
TcasGr4         LLSCSFP----------------------------------------------------- 29
TcasGr44        LLSCSFP----------------------------------------------------- 33
AgamGr9a        QLSIQN----------------------------------------------------HA 26
AgamGr9c        QLSIQN----------------------------------------------------HA 24
AgamGr9b        LISVQKCQCS-----------------------------------------------DHR 73
AgamGr9d        LFSIHQ----------------------------------------------------YR 26
AgamGr9e        VALARY-----------------------------------------------------D 22
AgamGr9f        MTLVRY-----------------------------------------------------D 22
AgamGr9g        LTMVKY-----------------------------------------------------D 22
AgamGr9i        LTMVKY-----------------------------------------------------D 22
AgamGr9h        ISYVKH----------------------------------------------------CP 29
AgamGr9j        LIPCSY-----------------------------------------------------N 66
AgamGr9l        ILPCRY-----------------------------------------------------N 56
AgamGr9k        FLPVSY-----------------------------------------------------N 56
AgamGr9m        YLPVAY-----------------------------------------------------D 52
AgamGr9n        FTPCSY-----------------------------------------------------N 26
AgamGr10        FTPCSY-----------------------------------------------------N 26
AgamGr11        LVPCSY-----------------------------------------------------N 26
DmelGr23aA      ALVPLP---------------------------------------------------PIS 28
DmelGr23aB      LVAFSL---------------------------------------------------RSR 30
DmelGr39aA      LTNLDF---------------------------------------------------SKS 28
DmelGr39aB      IFCIDY---------------------------------------------------NPT 28
DmelGr39aC      VLRFTI---------------------------------------------------DFN 29
DmelGr39aD      MMCWHF---------------------------------------------------DSD 28
DmelGr98c       LTPPAE---------------------------------------------------FFT 34
DmelGr98d       LTPPIQ---------------------------------------------------FFT 32
DmelGr98b       LTPPPQ---------------------------------------------------SFG 32
AgamGr51        I--LPLSFD--------------------------------------------------R 54
AgamGr52        CGTFPLRFD--------------------------------------------------A 49
DmelGr94a       LLAN-------------------------------------------------------- 32
```

```
DmelGr97a       VYLG-------------------------------------------------------- 52
DmelGr93b       VITFRIERK--------------------------------------------------D 51
DmelGr93c       VICFDIGQR--------------------------------------------------T 39
DmelGr22d       IFPFKYEP---------------------------------------------------- 36
DmelGr22e       VFPFAYDS---------------------------------------------------- 37
DmelGr22a       LFPFTFDS---------------------------------------------------- 38
DmelGr22b       IFPFTLDSG--------------------------------------------------K 38
DmelGr22c       VFPYRFDS---------------------------------------------------- 36
DmelGr22f       LFPFTFDS---------------------------------------------------- 38
DmelGr36a       LINFEIDWQ--------------------------------------------------- 29
DmelGr36c       LSNFEFDWN--------------------------------------------------- 28
DmelGr36b       LSNFEFDCR--------------------------------------------------- 29
DmelGr59d       VINFKIDLK--------------------------------------------------- 29
DmelGr59a       MTSYETMG---------------------------------------------------- 24
DmelGr59b       ITSQQFSN---------------------------------------------------- 25
AgamGr26        TVPWEVT-L--------------------------------------------------F 40
AgamGr27        LVAWDLQPL--------------------------------------------------E 41
AgamGr29        TAPYPLDPY--------------------------------------------------Q 41
AgamGr31        TAPYPLDPY--------------------------------------------------Q 41
AgamGr30        FLPYPFD-F--------------------------------------------------Q 39
AgamGr32a       FLPFPAY-V--------------------------------------------------L 39
TcasGr2         FFP--VQGVRG-----------------------------------------------PD 42
TcasGr9         FFP--VQGVRG-----------------------------------------------PD 42
TcasGr7         FFP--AQGILG-----------------------------------------------RD 59
TcasGr30        FFP--AQGILG-----------------------------------------------RD 59
TcasGr29        FFP--AQGILG-----------------------------------------------RD 59
AaegGr64f       -MP--ISGYFR-----------------------------------------------TP 10
AaegGr64a3      -MP--ISGYFR-----------------------------------------------TP 10
AgamGr14        LIP--IDGYARS----------------------------------------------TD 73
AgamGr18        LLP--VVNIFS-----------------------------------------------AN 44
AaegGr64d       LMP--VVEIFS-----------------------------------------------HN 74
AgamGr17        LFP--LGGLYG-----------------------------------------------RT 35
AaegGr64c       LFP--VQGVFG-----------------------------------------------RD 62
DmelGr64c       VLP--VAGVWPS----------------------------------------------CR 40
DmelGr64d       VLP--LAGINPN----------------------------------------------GK 49
AgamGr20        VFP--LSNITA-----------------------------------------------TD 70
AaegGr61a       VFP--LNSVMN-----------------------------------------------RL 64
AgamGr21        IFP--IYGVTR-----------------------------------------------ND 90
AaegGr64a       ------------------------------------------------------------
DmelGr64a       IMP--LVGIRE-----------------------------------------------SN 85
DmelGr61a       IMP--LSNIRS-----------------------------------------------RD 68
AgamGr15        IMP--VCGILA-----------------------------------------------KD 77
AaegGr1         IMP--VCGIGQ-----------------------------------------------KD 82
DmelGr5a        LMP--VCGISA-----------------------------------------------PT 79
DmelGr64f       MMP--VKGVTG-----------------------------------------------KH 103
DmelGr64e       LMP--VRKVSS-----------------------------------------------KF 96
AmelGr1         LFP--VSGVNS-----------------------------------------------PD 23
AmelGr2         MFP--VSGIGS-----------------------------------------------SS 104
AgamGr16        LLP--LDGVWC-----------------------------------------------GR 51
AaegGr64e       IFP--VVGYGV-----------------------------------------------AL 69
TCasGr6         VMP--IQRGEIG------------------------------------------------ 77
TcasGr26        VMP--IQRGEIG------------------------------------------------ 77
TcasGr33        VMP--IQRGEIG------------------------------------------------ 77
AgamGr23        VMP--ITRSVPG------------------------------------------------ 127
AaegGr21a       VMP--ILRSSPG------------------------------------------------ 119
TcasGr10        VMP--IERSGKG------------------------------------------------ 74
TcasGr39        VMP--IERSGKG------------------------------------------------ 74
AgamGr22        VMP--IMRSPKG----------------------------------------------VD 96
AaegGr21b       VMP--IVRSPKG----------------------------------------------VN 96
DmelGr21a       VMP--IHRNPPE----------------------------------------------KN 97
AgamGr24        VLP--YTRLPSG----------------------------------------------G- 28
AaegGr63a       VFP--FSRPSPG----------------------------------------------V- 93
DmelGr63a       VLP--IVRHGPA----------------------------------------------R- 122
TcasGr3         IFP--IKNTEPG------------------------------------------------ 85
DmelOr83b       LSGNTITTLFFT----------------------------------------------HC 87
AgamOr7         LTANTITTLFFT----------------------------------------------HS 85
HvirOr2         LTANTITVLFFA----------------------------------------------HT 88
TcasOr16        LAANTITVLFFT----------------------------------------------HC 186
TcasOr22        LAANTITVLFFT----------------------------------------------HC 225
AmelOr2         LTANTITMLFFT----------------------------------------------HS 85
```

```
TcasGr31        ------------------------------------------------------------
TcasGr35        ------------------------------------------------------------
DmelGr59c       LAVGVSN---------------------------------------------------FE 25
DmelGr92a       VIFF-------------------------------------------------------- 33
DmelGr28a       LAPFRLN----------------------------------------------------- 36
DmelGr64b       LLPVSNVR-------------------------------------------------ALD 33
TcasGr71        MTP--------------------------------------------------------- 21
AgamGr19        QFP--------------------------------------------------------- 21
AaegGr64b       IIG--------------------------------------------------------- 21
DmelGr93d       LVCFRLRKQKDN-----------------------------------------------N 35
TcasGr34        IGP--------------------------------------------------------- 20
TcasGr43        LTP--------------------------------------------------------- 20
TcasGr47        G----------------------------------------------------------- 18
AmelGr3         LVPVRFFVHTS------------------------------------------------- 44
AgamGr32b       LAPYPLS---------------------------------------------------VF 39
AgamGr50        VLP--------------------------------------------------------- 25
TcasGr20        FLPVCS------------------------------------------------------ 32
TcasGr79        LTP--------------------------------------------------------- 20
TcasGr15        ITP--------------------------------------------------------- 20
TcasGr60        LID--------------------------------------------------------- 18
AgamGr38        LVLKMSG---------------------------------------------------MG 39
TcasGr51        ILGDTVEIFNSLFG------------------------YQIILVIFDCCLETVSALNGAF 264
DmelGr39b       LVP--------------------------------------------------------- 19
AgamGr28        NVP--------------------------------------------------------- 33
TcasGr61        FMHKKSFKNFVN-------------------------------------------NVAAL 118
TcasGr50        LLP--------------------------------------------------------- 21
AgamGr12        LVPCR------------------------------------------------------- 24
AgamGr36        LTAFS------------------------------------------------------- 31
AgamGr48        LVPYELN----------------------------------------------------- 25
AgamGr49a       ADRATTTLG--------------------------------------------------R 42
AgamGr49b       FVPLHFN----------------------------------------------------- 29
DmelGr2a        LWPWRLAPPPD------------------------------------------------S 34
DmelGr32a       LMPIYEQVS--------------------------------------------------- 77
DmelGr68a       ILPFYSG---------------------------------------------------DV 27
AmelGr7         MGLTGIEAK--------------------------------------------------- 31
AmelGr8         LIYYIAFIN--------------------------------------------------R 39
AmelGr9         YFPYKWR----------------------------------------------------- 42
TcasGr57        N----------------------------------------------------------- 16
TcasGr41        ICVP-------------------------------------------------------- 28
AgamGr34        LAPFELN----------------------------------------------------- 32
AgamGr35        LTPGLMD----------------------------------------------------- 57
AgamGr39        LISFTT------------------------------------------------------ 31
AgamGr40        IFT--------------------------------------------------------- 27
AaegGr28d       FHFET------------------------------------------------------- 29
AgamGr42        LFPYTISQAKGR----------------------------------------------SF 43
AgamGr41        IHVLR------------------------------------------------------- 43
AgamGr43        LQLHDS-----------------------------------------------------Y 33
DmelGr33a       LCLLDSCN-------------------------------------------------SVC 65
AgamGr44        YLTITYD----------------------------------------------------- 29
AgamGr13        LVPFRYN----------------------------------------------------- 25
AgamGr45        CKSDKCVKIGTADIP--------------------------------------------V 62
AgamGr46        LWPFGG------------------------------------------------------ 44
Dmelr93a        LLVLSSSLD--------------------------------------------------- 39
AmelGr6         LAPYDFTG---------------------------------------------------D 79
AmelGr10        CTALCAS----------------------------------------------------- 25
TcasGr28        AYP--------------------------------------------------------- 25
TcasGr40        VFP--------------------------------------------------------- 42
AgamGr3         FFNFPLK----------------------------------------------------- 34
AgamGr4         FIPFP------------------------------------------------------- 57
AgamGr6         FTP--------------------------------------------------------- 55
AgamGr7         VLTFCIIER--------------------------------------------------- 49
AgamGr47        YMELLDN----------------------------------------------------V 43
DmelGr8a        FHGLPLP----------------------------------------------------- 27
AgamGr5         MEPLEQWYVLSG------------------------------------------YVRFHQ 69
AgamGr8         LQWFS------------------------------------------------------- 20
DmelGr9a        LVCG-------------------------------------------------------- 21
DmelGr59f       LISVLAN----------------------------------------------------- 48
DmelGr77a       LMATAN------------------------------------------------------ 54
DmelGr85a       ILDFN------------------------------------------------------- 43
TcasGr27        VWHVEN------------------------------------------------------ 27
```

```
TcasGr48        LFP--------------------------------------------------------- 37
AgamGr1         VSSLIVD----------------------------------------------------- 32
DmelGr10a       QVVIDY------------------------------------------------------ 36
DmelGr59e       VYP--------------------------------------------------------- 21
DmelGr10b       FSSHYYN----------------------------------------------------- 29
DmelGr89a       LAPFRTS----------------------------------------------------- 28
DmelGr57a       CNGFGIR----------------------------------------------------- 35
DmelGr47b       LVTIRVRSP--------------------------------------------------D 32
DmelGr58a       LMPAPLK----------------------------------------------------- 21
DmelGr58b       LMSTTLR----------------------------------------------------I 29
DmelGr98a       MLPFG------------------------------------------------------- 29
AaegGr93a       LRCVRFDR---------------------------------------------------- 32
TcasGr19        LHSLKN------------------------------------------------------ 31
DmelGr47a       LNG--------------------------------------------------------- 20
DmelGr58c       LCNLHYD----------------------------------------------------- 25


TcasGr38        GFVTFTCTRSNFRSSKLLILYNIILQVLFVSFVSYWLYLVLEADDMLPIYKNTYLIILFA 102
TcasGr46        GFVTFTCTRSNFRSSKLLILYNIILQVLFVSFVSYWLYLVLEADDMLPIYKNTYLIILFA 84
TcasGr1         CLVNFNHRESYFRLSKAKSFCTFVAALVYCSVTFFALSELLTDLATSILLKVSSLLIGFC 83
TcasGr123       GLVTFTPDRSKFRPSSLRFCLNILSIFTYVPMILYCVYTTAT-YDSFMIYKTTNIIILGL 84
TcasGr62        GLVTFTPDRSKFRPSSLRFCLNILSIFTYVPMILYCVYTTAT-YDSFMIYKTTNIIILGL 359
TcasGr25        -----------------------------------------------------------M 1
TcasGr59        KLKHMLIWKSYKRTQIFITCELFFVILLWISFFLNFMLHCNNTTWRCLYRWIVLYTLSKM 176
AgamGr2         HLAGEGVLINNGRMRRYSIVLLLLMLLFEAIITVYSFVAFEEEFNAWSLIWFITTIPTAL 92
AaegGr66a       KLAGENIPVDNGRLRRYVVILLVLVFVFEAILTTYNFVEFSEAYTLLSLMWFITTFPTAI 180
DmelGr66a       RLYKEGCVMDNSTIGRRIRIMLIMTVIFELSILVSTYVKLVDYSQWMSLLWIVSAIPTFI 188
TcasGr11        ----KGELKQHKSDYFYFSFYITSYTLLSVYSLFRIATNENNSLVINKRLIFIECFVMMA 100
TcasGr55        ----KGELKQHKSDYFYFSFYITSYTLLSVYSLFRIATNENNSLVINKRLIFIECFVMMA 100
TcasGr21        VENGKKVFKFAWWPLTRNALLVLILLGALTYHAIFDLISFKDSDLQQKLRYFEEVFSSLL 99
TcasGr56        VENGKKVFKFAWWPLTRNALLVLILLGALTYHAIFDLISFKDSDLQQKLRYFEEVFSSLL 99
AaegGr          RRRHDSALECTIFGYVNSFIHAATFCTCYVITLVK-HESVTAHFFDSEISTLGDILQIIV 98
AaegGr28a       ------------------------------------------------------------
AgamGr33        GPTELMMLRCTPFGYINSSVHVILFCSCYVNALLQ-GDTITRFFFRTDISTLGDVLQFTI 96
DmelGr28bB      RKMGESYLKMSCFGVFNIFIYICLCGFCYISSLRQ-GESIVGYFFRTEISTIGDRLQIFN 98
DmelGr28bC      TKTGKKAIKKTIFGYINGIMHIAMFVFAYSLTIYNNCESVASYFFRSRITYFGDLMQIVS 125
DmelGr28bD      -QAGERQFHLSKIGYLNAFLQLSFFSYCFLAALIE-QQSIVGYFFKSEISQMGDSLQKFI 95
DmelGr28bA      NALGENSLEQSWYGFLNAIFRWLLLAYCYSYINLR-NESLIGYFMRNHVSQISTRVHDVG 107
DmelGr28bE      SSEGRGKLTSSYIGYINIIIRMAIYMVNSFYGAVN-RDTLMSNFFLTDISNVIDALQKIN 100
AgamGr25        PIGQIVDYKRSVFLIAYSVGLVFCLAGLTYKGIFFDVTSKKPIRMKTATSKVVTILDVSV 87
AaegGr43a       SKGQIVDYKRNLVLVIYGLGIVFCLAGLTYKGIFFDINSKKPIRMKTATSKVVTTLDVSV 87
DmelGr43a       SKG-RVEIGRSWLFTVYSATLTVVMVFLTYRGLLFDANSEIPVRMKSATSKVVTALDVSV 86
AgamGr37b       SQ-PSNDRKELYYAFGYSASFICVYSYAIYGYLFTTNTGH--LYI-SKIVAVMENGYMFC 89
AgamGr37d       MK-PVNRYLEFCYVLVYGG----MYTYALYSFLFVANVTD--FHL-SLIIGTIECINLSC 85
AgamGr37a       ADTPDRDAFGTWRTKLYTISFGLVYTLAFIKFISELKHTG--FSA-SMIEGKGERMYFTF 99
AgamGr37c       KRTRLPYRCVNILSFVLST---LFYGYLLFSISLDSGVLK--AGIKSLIVTRMHDLYFVL 87
AgamGr37e       KRTLLGYKLCNLLAIAFSA---TFYGFLLLFHSLDLETIK--LGSGSLITSRMHDVYFVS 87
AgamGr37f       NR-RLLSVLNVIILLLIGT----LYINGPCR-QIRKDMLR--FQS-SPLAVNSRIFTYLM 89
AaegGr28c       RQSQLHYRLINLISLTFIG---CFHGLLMIHFLLGNDFTR--YSTGSVIVQQMLYAFYVA 87
AaegGr28b       GFGKRSMIFTWSDIFPIIRLLCLQATSLYISYAMADHMES--TGS--NIMDFGLKYNLAI 99
AaegGr28e       GLGKRSMIFTWSDIFPIIRLLCLQATSLYISYAMADHMES--TGS--NIMDFGLKYNLAI 99
TcasGr14        LPKTPLPLTLLYKLLGIIQFPISAHFSFMSRFLCLPF-YSYFFYLSYIYTTYSRKLSGIF 62
TcasGr49        LPKTPLPLTLLYKLLGIIQFPISAHFSFMSRFLCLPF-YSYFFYLSYIYTTYSRKLSGIF 106
AmelGr4         NNRRNSKIWSSKNVVLFVNSKLGILYNLFVSFLIITLNFSLMPLIFYAEYAFRTNITIIL 92
AmelGr5         NNRANLNKKNSENTMFFISSKLGILYNLFGSCLIIALSFYSIPITMYADYVHKTIVTITI 92
TcasGr16        VNYSKIGIVQTVAYFVIFIILTIVFFEKRSALSPFELIQIRTINFVATFRTIANIILMAV 91
TcasGr22        -----------------------------------------------MFRDLG---STTM 10
TcasGr17        SPLKVLQTVILGTTCIYLTSLYIWFILTQMTFS--GVTSPTIMKFVLSFRTGSFTFLMVV 86
TcasGr150       SPLKVLQTVILGTTCIYLTSLYIWFILTQMTFS--GVTSPTIMKFVLSFRTGSFTFLMVV 86
TcasGr32        RKNRTTCPEKIYAYGVVAFITLGVAVSVFYRAKDY-------AKFIHIKAVVQITLDATL 75
TcasGr54        RKNRTTCPEKIYAYGVVAFITLGVAVSVFYRAKDY-------AKFIHIKAVVQITLDATL 75
TcasGr37        -TKEKRFPHRIHSYFMVAFYVVFSSVSTFFKIPFY-------STLRPINLLVQIILDVIL 68
TcasGr53        -TKEKRFPHRIHSYFMVAFYVVFSSVSTFFKIPFY-------STLRPINLLVQIILDVIL 68
TcasGr5         SVEPTGFSPKGYSLLWILLFTLGVTISGVYRTDFY-------KKLSPMRLIVQTCLDFLL 74
TcasGr12        ---VKFYVQKFYVVILAFLLTAGAIISLVYQAPVY-------KELTDIKLVLNILSDVTL 71
TcasGr13        ---VKFYVQKFYVVILAFLLTAGAIISLVYQAPVY-------KELTDIKLVLNILSDVTL 145
TcasGr104       SFEKPDSQKSLWKKIQGVVLVSIIAAGTAYSIYVR-HTYYRRLYTITH-LVLDYLDEFLI 88
TcasGr45        SFEKPDSQKSLWKKIQGVVLVSIIAAGTAYSIYVR-HTYYRRLYTITH-LVLDYLDEFLI 88
TcasGr98        DFENFVIISPRCDKISAICFLLSTILGTCWIIYVRIYCKEIRFFEISF-EILGSLDSLIL 88
TcasGr52        QYIN-------------------------------------RSELITS-KIVSFVNSIFL 27
```

```
TcasGr105       NFEKFSLEHQLWQKIQAWTYLILLTTWTVISASTR--IKSFKFLTIGVGITTDTIDRVFT 85
TcasGr4         ----------------KKKLVYCIIFGAIATLTCIEGLRDCPRKYSTPLAKITTILQRYC 73
TcasGr44        ----------------KKKLVYCIIFGAIATLTCIEGLRDCPRKYSTPLAKITTILQRYC 77
AgamGr9a        EEDGLFRSTRHATSKFVLSFTVSVLACSYLMHCSIDDYDKLSVN--GIIWPLVYVVHNAT 84
AgamGr9c        EEDGLFRSTRHATSKFTLSFIVSMVASSHLIYRGVKDYDQLSVD--GIIWPLIYVVHYAM 82
AgamGr9b        SGSNPFKSTNNAAPKFMILFALSLLTSGHSIFRGTIDNSVIDALDQGTIWPFVYLVHQIT 133
AgamGr9d        HTGNTFECSRHAALKFVIFLTITLACSGYSMYRNILDYPAMAAMNNTAIEPFFYIAHTIT 86
AgamGr9e        GEQCSYR--RPTTHQCVTHFLLVLFSIGFLLYCIWEQYSIF-AMAIYAFDDATFILSTAS 79
AgamGr9f        VRHRSYR--KPTPFQFSVQLLYSLCCITFTSFRVWVQFSVHSQIEHEMLENISFIVSTAS 80
AgamGr9g        PGKHTFISVPYAPLKFSCLFTAALLSSGYLLWRSPMDYIVY-KLDTRMFDITIFIINTTI 81
AgamGr9i        SEKHTFNSVQNARFKFSCLFAVALISSGYLIWRSPMDYSVY-KFDSKIFDITIFIINTII 81
AgamGr9h        STSDRFVVPKSLTIKFGFWIVTLLAIGCYSLIRTKQDVLER-SQKESYFDYVLFFVSNMM 88
AgamGr9j        IRTGLFDCSYRNTLVCVCNVVFFGGFVWYDFGMIL-KFYAT----LPIVLVGILTVDVTV 121
AgamGr9l        FRTHMFEKSWTNMVALVLNIVLHSLCLWLDLNAIK-ISIGY----MSFVMLGVIVIYLVT 111
AgamGr9k        QTTQRFVQQRTNLVSFGVGILLSFTFFYHDIFIQN-VFFAN----LPPFAFAIVLLELLL 111
AgamGr9m        AVEHRFIEKRSNLIPFAIGLLLSIGFIYHDFFIVLPNFNTD----QSAFTLAVFIVELTV 108
AgamGr9n        EELGRFEATKRNLAFLGAALVLTIPFWVYDIHLMATNFLHT----YTTVFAAVGGIELLI 82
AgamGr10        EELGHFVATKSNLTVFGVALVLTISFWVYDIHLMATNVLRT----YTTVFAAVGGIELLI 82
AgamGr11        TETDQFEQRTVNKLAFAVGIGMTVPFWYFDLKFMIAFYLEN----ISPIMVAVGSIEIAV 82
DmelGr23aA      QLGWLFLSLAIRCCWIVYFIYLLDVAISFSWVAIENVGNAVGTMLFVG---------NSV 79
DmelGr23aB      RLSRLVLWLQFLG-WLTWFISMWTQSVIYAQTIDCTLDCSLRHILTFF---------QTV 80
DmelGr39aA      LHIYWLHG--TWSSTAIQIVVVGVFMAALLGALAES-LYYMETKSQTG---------NTF 76
DmelGr39aB      KKKFRLRR--SVLCYIVHFALQAYLVGCISVMVTYWRRCFKSELTTTG---------NHF 77
DmelGr39aC      KCLVRENASEERSAWLYLIGVVGITCSLIVYSTYFPSHFIMGKHNTTG---------NCY 80
DmelGr39aD      HCQLVATSGSERYAVVYAGCILVSTTAGFIFALLHPSRFHIAIYNQTG---------NFY 79
DmelGr98c       RTLRKRRRFCWMAGYSLYLIAILLMVFYEFHANIVSLHLEIYKFHVEDFSKVMGRTQKFL 94
DmelGr98d       RTLHKRRRGIVILGYACYLISISLMVIYECYANIVALQKDIHKFHAEDSSKVMGNTQKVL 92
DmelGr98b       HTPHRRLRWYLMTGYVFYATAILATVFIVSYFNIIAIDEEVLEYNVSDFTRVMGNIQKSL 92
AgamGr51        HR-ERFRNRTKDVILSTVLLVLYAIVGPIVCVELYVMNISVEN-QITNALSAFQFAFVYF 112
AgamGr52        VGPGRFFTSRKCALYCSGITVVYAVLSPALMYLIYEHERVANV-YLVSMLNAAQLSFIYL 108
DmelGr94a       ----RYRAGRRERFRFSKANLAFASLWAIAFSLVYGRQIYKEYQEGQINLKDATTLYSYM 88
DmelGr97a       ----RFSF-RRKKFVFSKGLTIYSLFVATFFALFYIWNIYNEISTGQINLRDTIGIYCYM 107
DmelGr93b       SQ--LVAINRRGYLWICLVIRLLASCFYGYSYDAWSGQYEDMYLRAFFGFRLIGCLICSV 109
DmelGr93c       SDDSLVVRNRHQFKWFCLSCRLISVTAVCCFCAPYVADIEDPYERLLQCFRLSASLICGI 99
DmelGr22d       -KKRRLRRSMWLIPFGVVISSSLLILMVKQSAEDREHGIMLDVFQRNALLYQISSLMGVV 95
DmelGr22e       -WTRTLRRSKWLIAYGFVLNAAFILLVVTNDTES-ETPLRMEVFHRNALAEQINGIHDIQ 95
DmelGr22a       -RKRRLNRSKWLLAYGLVLNLTLLVLSMLPSTDD-HNSVKVEVFQRNPLVKQVEELVEVI 96
DmelGr22b       -RIRQLRRSRCLTLYGLVLN-YFLIFTLIRLAFEYRKHK-LEAFKRNPVLEMINVVIGII 95
DmelGr22c       -RNGQLKRSRFLLFYGLILN-FFLLLKMVCSGGQ-KLGI-PEAFARNSVLENTHYTTGML 92
DmelGr22f       -RRKQLKRSRWLLLYGFVLHSLAMCLAMSSHLASKQRRK-YNAFERNPLLEKIYMQFQVT 96
DmelGr36a       --RGRVVAAQRGILFAIAIN-VLICMVLLLQISKKFN-LDVYFGRANQLHQYVIIVMVSL 85
DmelGr36c       --TGRVFTKKWSTLYAIALD-SCIFALYIYHWTGNTNIVNAIFGRANMLHEYVVAILTGL 85
DmelGr36b       --TGRVFKSRRCTIYAFMAN-IFILITIIYNFTAHGD-TNLLFQSANKLHEYVIIIMSGL 85
DmelGr59d       --TGQALVTRGATLISVSTH-LLIFALLLYQTMRKSV-VNVMWKYANSLHEYVFLVIAGF 85
DmelGr59a       ---GKFRQSRITRIYCLLIN-AIFLTLLPSAFWKSAK-LLSTADWMPSYMRVTPYIMCTI 79
DmelGr59b       ---RKFFSTLFSRTYALIAN-IVTLIMLPIVMWQVQL-VFQQKKTFPKLILITNNVREAV 80
AgamGr26        AHVTQPLSSWRERLFRTLNALYSLALGLAVLSATVLQHAE----FDHHMPFVTHMLYISE 96
AgamGr27        PDRPTTKLHWFRKLIRATNQGYCFMIIVAIGTATVLHHSS----NTANAFFAIRTLYLTE 97
AgamGr29        RATRNYQAKCSRLVALFRRTLSLLLFTVILAAPFLLYHLYSGQIYAYTIPLSVKLMYYVQ 101
AgamGr31        HIKPYQQSKSFQTINILRRLLAALLFGIALSVPFLLYFLKADHVNAFKIPLSIRLMFYLQ 101
AgamGr30        RQAEVKNIVLQKMLTILNRTFGLAMIGTVLTCCVSLYVCYPSYMYEEDYPPVLNIAYHLE 99
AgamGr32a       RQDVERLHFRTLVLTCLNLTFAVLIFLTSFVCYLAMYIYYPDLMYKENLPAVLQIMYHVE 99
TcasGr2         FRSLRFSWKSARVVYALFTLLGTFLISGFQMQKIATKG-LDLLEANR--------LFFFL 93
TcasGr9         FRSLRFSWKSARVVYALFTLLGTFLISGFQMQKIATKG-LDLLEANR--------LFFFL 93
TcasGr7         FRAIHFTWASARVGYTIVTILGATFVTVLQLHKIFAKG-LNVIEANR--------LFFYC 110
TcasGr30        FRAIHFTWASARVGYTIVTILGATFVTVLQLHKIFAKG-LNVIEANR--------LFFYC 110
TcasGr29        FRAIHFTWASARVGYTIVTILGATFVTVLQLHKIFAKG-LNVIEANR--------LFFYC 110
AaegGr64f       ISKLKFTLKSVHFAYGCFTVFIMGAIMSMFFAFRIQRGTFGIGATTT--------CIYYA 62
AaegGr64a3      ISKLKFTLKSVHFAYGCFTVFIMGAIMFMFFAFRIQRGTFGIGATTT--------CIYYA 62
AgamGr14        PRDVRMRLRSVQFVYGCVTLFIMLTLIIMLCVHTAHEPSFGVQQATS--------LVYYA 125
AgamGr18        YRTARFKLRSFRCIYSLVYLALTGIYCTLFIRWYIRKG-LNLAYFAN--------CIYMV 95
AaegGr64d       PRNTRFKLLSVRFAYTAIYLIAAGFYSILACRRFILRG-LNVSSFAD--------MFYLM 125
AgamGr17        LQDIRFRWFGPGTVYSFYFFLSGLLTLVAHIYYSLTVETLGTSEISN--------IIYYV 87
AaegGr64c       LQQIRFQWLHLRTLYSLTFLVLAALIIIAQINHTV-VSTANTSMITS--------VLYYI 113
DmelGr64c       PERVRFRWISLSLLAALILFVFSIVDCALSSKVVFDHG-LKIYTIGS--------LSFSV 91
DmelGr64d       PENVRFRWFSPYILFFVVAFTFVIADFMLSTKIVLNDG-LQLYTMGS--------LSFSV 100
AgamGr20        PSAFRFHYFSPYIVLSALSIVGGLFIMAAALVRLNRVG-INAMNIAE--------PIFFG 121
AaegGr61a       PGNMLYKSVSFATALSMMAIFGGYAVSLLSLKRLARTG-LDAINMAE--------PFFFA 115
AgamGr21        PKRFRLKWFSLRVILNLTVVVTALLQAYYEYGRLKAIG-INAKNVSS--------LIFFI 141
AaegGr64a       ------------------------------MRLRMVG-VNAKNING--------LIFFV 20
```

```
DmelGr64a       PRRVRFAYKSIPMFVTLIFMIATSILFLSMFTHLLKIG-ITAKNFVG--------LVFFG 136
DmelGr61a       PQDVKFKVRSIGLAVTGLFLLLGGMKTLVGANILFTEG-LNAKNIVG--------LVFLI 119
AgamGr15        PRKLRFSYTAGRTFYAYFCAIGIGFLATMSVYFFASKR-YHFQKMVT--------AFFYC 128
AaegGr1         TTKLHFSWKSKRIFYSYAACMGTAFLAVTSTIRFVDRN-FNFSRLTG--------VFFYF 133
DmelGr5a        YRGLSFNRRSWRFWYSSLYLCSTSVDLAFSIRRVAHSV-LDVRSVEP--------IVFHV 130
DmelGr64f       PSDLSFSWRNIRTCFSLLFIASSLANFGLSLFKVLNNP-ISFNSIKP--------IIFRG 154
DmelGr64e       AEDLTFTWFSVRSYYALVTILFFGVSSGYMVAFVTSVS-FNFDSVET--------LVFYL 147
AmelGr1         SSYLRFTWRSPKFIYCTISFLSSSIMTIFNVLRIVTTG-ISSIKMTT--------FVFNG 74
AmelGr2         LSKLQFKIFSLLTMYSGFIALMISFMTIVSMIHMLKTFNASTFQIRGGLGAATVGAVFYG 164
AgamGr16        WWSIHWRLLSWRNLYALFVQLGALIMACFSFATFWYSG-VEFAKIMS--------WWFFT 102
AaegGr64e       AERIQFKWCSLRMLYTMMFQLGGAIMSGFSLATFWTTG-VEFSKILS--------WMFFT 120
TCasGr6         --RITFGWTSIPMLYAYVFYVVTTVLVVLVGYERFDILLN--KSK--------------- 118
TcasGr26        --RITFGWTSIPMLYAYVFYVVTTVLVVLVGYERFDILLN--KSK--------------- 118
TcasGr33        --RITFGWTSIPMLYAYVFYVVTTVLVVLVGYERFDILLN--KSK--------------- 118
AgamGr23        --RITFSWRSAASIYAFCFYLVSTVIVLVVGYERIKVFQT--TTKFDEYIYGILFVIFLV 183
AaegGr21a       --RITFDWRSWASIYAYCFYVVSTVIVLIVGYERLKILQD--TKKFDEYIYGVLFIIFLV 175
TcasGr10        --RTTFRWLSSTSIYAYFIFGAETIFVTMVFKERLYLILR-PGKRFDEYIYGIIFLSILI 131
TcasGr39        --RTTFRWLSSTSIYAYFIFGAETIFVTMVFKERLYLILR-PGKRFDEYIYGIIFLSILI 131
AgamGr22        MPRTTFTWCSKAFLWAYFIYACETVIVLVVARERINKFISTSDKRFDEVIYNIIFMSIMV 156
AaegGr21b       MPRTTFTWFSKAFIWAYFIYACETVLVVLVAKERIKRFISTSDKRFDEVIYNIIFMSLLV 156
DmelGr21a       LPRTGYSWGSKQVMWAIFIYSCQTTIVVLVLRERVKKFVTSPDKRFDEAIYNVIFISLLF 157
AgamGr24        ---TAFVLASPSMTYCVLFFLLLTVYIAFILLNRIEIVRT-LEGRFEESVIAYLFIVNIL 84
AaegGr63a       ---TLFACASPAMAYCGVLFVTLMAYVIYITILRVHIVRT-LEGRFEEAVIAYLFIVNIL 149
DmelGr63a       ---AKFEMNSASFIYSVVFFVLLACYVGYVANNRIHIVRS-LSGPFEEAVIAYLFLVNIL 178
TcasGr3         ----MFRVAPELLGYSVVVFVVVMGYIGFIEWDKVEIVRS-QEGRFEEAVIDYLFTVYLL 140
DmelOr83b       ITKFIYLAVNQKNFYRTLNIWNQVNTHPLFAESDARYHSIALAKMRKLFFLVMLTTVASA 147
AgamOr7         VTKFIYFAVNSENFYRTLAIWNQTNTHPLFAESDARYHSIALAKMRKLLVLVMATTVLSV 145
HvirOr2         IIKLAFFALNSKSFYRTLAVWNQSNSHPLFTESDARYHQIALTKMRRLLYFICGMTVLSV 148
TcasOr16        VTKFVYFAVRSKLFYRTLGIWNQPNSHPLFVESNNRYHGIALKKMRRLLYIIIIWTSFSA 246
TcasOr22        VTKFVYFAVRSKLFYRTLGIWNQPNSHPLFVESNNRYHGIALKKMRRLLYIIIIWTSFSA 285
AmelOr2         VVKLVYFAVRSKLFYRTLGIWNNPNSHPLFAESNARYHQIAVKKMRILLLAVIGTTVLSA 145
TcasGr31        -MGSVAINHSLKKRLKIITIVILVVATGNIIHTTSNKQ----------------LQFFAV 43
TcasGr35        ---MRLKWYYITISMAFIIFVSLEIYHIYTVVEESGFENFVKTYLVSDFENYVTFFCSLF 57
DmelGr59c       VDWLTGEAIATRRTTIYAAVHNASLITLLILFNLGNNSLKSEFISARYLHEYFFMLMTAV 85
DmelGr92a       --CLHTRKDDKTVFIRNWLKWLNVTHRIITFTRFFWVYIASISIKTNRVLQVLHGMRLVL 91
DmelGr28a       --LNPRKEVQTSKFSFFAGIVHFLFFVLCFGISVKEGDSIIGYFFQTNITRFSDGTLRLT 94
DmelGr64b       VADIRFRWCSPRILYSLLIGILNLSEFGAVINYVIKVTINFHTSST---------LSLYI 84
TcasGr71        -----SRPKTRAKIYALCVVTALVTPSALSIIYYRQPIYAKISETKSIVAMVMGTIVNAF 76
AgamGr19        -LYGVLTRKPLRWHFRWCSLQTVLSLGLIHVGLFLCFVEYDRLKAIGVNADNLIGPLFYL 80
AaegGr64b       ----TYFFVDTAIIVCLTVLMASGWRRFLHEVEKLELIWTRWPYNLRRDNLKLNCWILTM 77
DmelGr93d       VWLEEIWSNRSRWKWISVTLRIVPLCIYAFTYAEWISNRMLITEKFLHSCSLVVSIPCYL 95
TcasGr34        ---------KSNKIYSFLLLTTLTLLVILSSIDRPYLKSYTYIKLVVS---------VLM 62
TcasGr43        --RSIKLEKQTFIQKFYAVAFFLALSIGVVVSIYSKSFHRNEIHIKIVLMFFKEASLYCF 78
TcasGr47        --------------KLWPISIVLIYTVGVVYSFKRRIFYQDESPISRLVYIGTDLSYFIS 64
AmelGr3         -EGCQARLNIIDLIYSLCVLVLLLSAEIWGLWRDLKDGWEYSTRLKSRTAVIATCSDVLG 103
AgamGr32b       QRNCSTRSVRIRIIICFRYAFATCVTVAVVASQFTMFYYFPHIMYQPKVPIFIVILYYIV 99
AgamGr50        FQYVVSTNRFVQSTFHYRGCIVGTVLYAMVGPWLYWISVTRILHPDSQLNQYMIVVQFIC 85
TcasGr20        SQKGQKTCLNLSINYCICGYIFSAILFSLSILGLTEDLNSAPIRMKNPIVKYIILIDLGE 92
TcasGr79        KSRIEKKPSRQKQFYALFLIFFYTFGEVFTLVLRIRNMEYRSQTLMQTTLRLIRDLSLYF 80
TcasGr15        -SSIYDKPTKLYQKLYPVLMFLIYTITEIYSVYERRYMNAHLSGIQLVVRNLLDISLYLN 79
TcasGr60        --WYTVCLVPLNPYQFFLAVQYPRIVVSTFNIVFYMYTLILEERFKLINLIIKQEEFFSP 76
AgamGr38        CITPRAGVRNGSTMTTLIDIGLFVTNLILAVTMMIYGMFITQRHMQSPIMFIGLKMVNFT 99
TcasGr51        LYTINGQGQFNIEMFLCNMSLLTVIPIHVWTSKEKISFLRGFWLSPAVDMFYEFQIIELM 324
DmelGr39b       --WSESCAQSKFVQKVYSAILIILNAVHFGISIYFPQSAELFLSLMVNVIVFVARIVCVT 77
AgamGr28        --CCLARDLPSRAYFKQARCLLTAGTFIWVILCCYQATVKLLELPIPFFTAVLYVNEMVL 91
TcasGr61        DETLAKLGQYINYETDYYVCLAMTITGPLVIIGNIAMELWNMPRENIEPLPNVILACHML 178
TcasGr50        ----VKKHHRLEKLVPCVYLTSFSVVLSLGSFIVSVYLTTKIDNDGESISLASGWLDLYL 77
AgamGr12        -YCRKENKFKVNPYLSLLAFGINISLASVHLCFDWLQIWNGERRDASRIFNFLQMLHTLT 83
AgamGr36        -IVGEPPYVQVKVTGVEYVALLMNFVANLYCVYINVTNNRLSRWTGSAVMNLGLGFLFPL 90
AgamGr48        NQNGLRRSKCKRQWTLTIGCTTVVLVGFSFCTVWLQDTLSFATFVDALLIRSLVLVEFTI 85
AgamGr49a       ALVWWFTMSHDVAITKAVLVLIKCFGQLYGILPLASNAFTFERSRPVLWYSRTIALLLMV 102
AgamGr49b       KQLQTFQISRWLTVWSHALALTLLCVYPYVYVILLTGIRYPKLTLVNYLVWMQCSLHYVN 89
DmelGr2a        EGILLRRSRWLELYGWTVLIAATSFTVYGLFQESSVEEKQDSESTISSIGHTVDFIQLVG 94
DmelGr32a       -DYEVGPPTKTNEFYSFFVRGVVHALTIFNVYSLFTPISAQLFFSYRETDNVNQWIELLL 136
DmelGr68a       DDGFRFGGLGRWYGRLVALIILIGSLTLGEDVLFASKEYRLVASAQGDTEEINRTIETLL 87
AmelGr7         KPSKKLEYVYTLCNLVLFYFINKLTLPYYDKYYVISTFELSRFIFQWMFHANIWTITLLI 91
AmelGr8         FMGFLPYKLESSKFMYSKLYSVFSTIIIIIYVFLAIFSLYEINFYIVMNDGLPRILHRNS 99
AmelGr9         ---RPVYLFSKTRFVISTFILLLSIFIWFNLFYQLNFTDIIKKNTTEVIHENMYALLIGI 99
TcasGr57        ---TVTPVSLRFKLYTIVHIFIIIALYAHSSYGRENFIYGSMNMTVAITDKIANFMLTFF 73
TcasGr41        --KITSTNNEVPRWYICYTIGVIITIGCLCIWSIFNKWNQFRSYSNFSVFLFDIITIVTL 86
```

```
AgamGr34        ----TSRVRLSKPFCCAVGGFVTLYWTAMVSSIATSNHANDRISRISNYFQLITNAIMLT 88
AgamGr35        RQKDRYRVRNTRWMMLIVLLLVVVAWIALFETFFIERRTALITGIANHIQFLMNTIALTV 117
AgamGr39        --TFDTVGIRRSFKDVLVFVGFMFFNCYIVFKTGIPPKSDSPSYFDSSLIQFILGLFLTL 89
AgamGr40        ---HTIDFERQVLSTNTRDQVLLVVAILMDVYALTVSTRVSYSFSNSILLNVGIYCSVNL 84
AaegGr28d       --IDFSTQTLHRTLTDQFRFVFTLLLDCYLVYKGIQSTSTYFYLTDSNIVNVGCFSAFIL 87
AgamGr42        PSNSHHSNNRCNHSKTLKSEVKFIDIAIFTLWQTFFLQMLYVSWPKMFLNMPVSRIITMV 103
AgamGr41        ---FDTTTDRPTLLELVLSFAITVIRIYMIYIILVTDAWDLIFVSSSQLVEKGLDIVLKI 100
AgamGr43        ETETNGKTPSLSIVRILYSIILFGAYLVGLYKAVELTFVVEFAANSPSIFKFAYMTQIYS 93
DmelGr33a       KLSSHLFMHLGAFLYLTITLLSLYRRKEFFQQFDARLNDIDAVIQKCQRVAEMDKVKVTA 125
AgamGr44        -RKRQELRVSTVLVVYSIVLAILCTFTYQSAQFLSSDMRYRNGIKESAVNKAIGAIQGQV 88
AgamGr13        -FRKRRFDTCKPFVPVATVLLLLFAAVQYRLGRLPERMKIPKVGMQDIIHAVENLVLNGN 84
AgamGr45        KRFKGKDGKPLAFSFIGVLHALGCSLAYASYHILSSLSQSGKLTDSNLVRVAIDLKNQYI 122
AgamGr46        WSSRFNRCSTLYVLAVVTAYSAFHLYINYTDAYSVGTGEDGGKPGPDSTPTVDRQQQVEA 104
Dmelr93a        RDKLQLKATKQGSRNRFLHILWRCIVVMIYAGLWPMLTSAVIGKRLESYADVLALAQSMS 99
AmelGr6         EITPSNACLIFSFVFIGIYCHIIYIVYKRFLNVRRDKAILSVVETTKVIPTVLRDLMVTV 139
AmelGr10        ----KDNVKARRLRDIIFIVFSMTLYLVSLVVIYIYVFSYEDSDLKSTLIIIIRVFLIYL 81
TcasGr28        --------QPSFHQTFSFVVIFGQFFGIMPLHGVSRKNVQEIRLEWKSFRFVYAVYNIFG 77
TcasGr40        --LNHLNEEPEKLHFTFKSWKVLYTSLTSFGYLFCASLSFYKAFKIGILLNQLITPLFFF 100
AgamGr3         -LHHADGLAIGEHRWSSSVWSILHLASAVISGILAKRHYNYLFGGLMLTDAMNNYFKYVI 93
AgamGr4         FDCCTFALTPCSNVRSLLHLPILQVSFYLTLFCIIMSNRVNFFFTGLQILSLNDIIKYGT 117
AgamGr6         --VPLHGQALTSLRTLTIVFVSFYTTGEIIVLSYSLIKPEAVFFLSDATGTFADAIQFVI 113
AgamGr7         -RWLFADARTGTGINFIPFIKTSITIVTHLVVLLEALIARGVYRALDVRVASVDGALGHL 108
AgamGr47        TKVVEQEFHVRYPIRVVQTFIVIVISSELIYLVVILAGYYYLIDQTLNITTKYLYASYYM 103
DmelGr8a        --GDGNPARTRRRLMAWSLFLLISLSALVLACLFSGEEFLYRGDMFGCANDALKYVFAEL 85
AgamGr5         WLGFQPYRLASGRVSPFAVLTLAQFLGVLVNLALIIYRRRCILYHCEAIGMVVDFIKLLT 129
AgamGr8         ------PKRYRTFDCESLFFITLNFCIITGLFCWIYQHQSLVIYSDNALGYVVDFLKFLL 74
DmelGr9a        ------WSGSRLGRLLSSTFLVLILIELVGEIETYFTEENPDNESVPAYFAKVIMGVNMA 75
DmelGr59f       -TAPITILPGCPNRFYRLVHLSWMILWYGLFVLGSYWEFVLVTTQRVSLDRYLNAIESAI 107
DmelGr77a       WSPRGIKRVRQSLYLRIHGCVMLIFVGCFSPFAFWCIFQRMAFLRQNRILLMIGFNRYVL 114
DmelGr85a       -TDIGTGNLRRYRVLFMYRLLHNFAVISLTLKFLFDFTDHFKYIESSTLITVNFFTYFTL 102
TcasGr27        ---PTYRLYKIFVVFSFAVTFFSAWICALVNYNVSEISENFYYLPAMSTGPLKYAIFQKN 84
TcasGr48        -ITINIRKNGLNYNFTKKCYFLRMVLIDLLIVGCILKHIFNILMKNVTLNDVVFIFCSAP 96
AgamGr1         -KRGNVRSGSAAPKFILAMLGLAISLAVWFILYHNESPVICRSKANQVRLFYYFVIIETL 91
DmelGr10a       --VPQRALKRGVKVLLIAYGHLFSMLLIVVLPGYFCYHFRTLTDTLDRRLQLLFYVSFTN 94
DmelGr59e       --SGRVGVLRWLHTLWSLFLLMYIWTGSIVKCLEFTVEIPTIEKLLYLMEFPGNMATIAI 79
DmelGr10b       -PTRRRLVYSRILQTYDWLLMVINLGAFYLYYRYAMTYFLEGMFRRQGFVNQVSTCNVFQ 88
DmelGr89a       --EPMVARCQRWMTLIAVFRWLLLTSMAPFVLWKSAAMYEATNVRHSMVFKTIALATMTG 86
DmelGr57a       -RSTFRISWASRIYSMSVAIAAFCCLFGSLSVLLAEEDIRERLAKADNLVLSISALELLM 94
DmelGr47b       RGGAFSNRWTVCYALFTRSFMVICFMATVMTKLRDPEMSAAMFGHLSPLVKAIFTWECLS 92
DmelGr58a       --KGQFLLGYKQRWYLIYTACLHGGLLTVLPFTFPHYMYDDSYMSSNPVLKWTFNLTNIT 79
DmelGr58b       RSCRKCLRLEKVSRTYTIYSFFVGIFLFLNLYFMVPRIMEDGYMKYNIVLQWNFFVMLFL 89
DmelGr98a       ------QNLFSKGFCYVLLFVSLGFSSYWRFSFDYEFDYDFLNDRFSSTIDLSNFVALVL 83
AaegGr93a       MKFSICSKCSSFAFIKWLWLLAVVLALCLSSYAFYFWYCSEPVAGKYLKQWISIIIVWFL 92
TcasGr19        --KRSGASLQVGILDSLVFKCYNLRRIYYFYCVSITFNVHLLFLLCSGYFTVHLLFYCPF 89
DmelGr47a       -------LNTYYFDTKTNAFRVSSKLKIYCAIHHALCVLALAHMSYSTASNLRVSVTVLT 73
DmelGr58c       --SSNHRFILNHVPTVVYCVILNVVYLLVLPFALFVLTGNIYHCPDAGMFGVVYNVVALT 83


TcasGr38        DFAYLETTWICTLLKKDKLLELFKRLIHFDTKCQ-----------ENSTVIDYKRHKKRL 151
TcasGr46        DFAYLETTWICTLLKKDKLLELFKRLIHFDTKCQ-----------ENSTVIDYKRHKKRL 133
TcasGr1         ASIYVGTVWINTSINGTKFIEFINKLIEFDVKLQ-----------NVSLIINYENQRTRS 132
TcasGr123       NVVYVVTAWICAITKRDMFVEFLLKLVDFDSKLQ-----------TMNIKVNYHRVHRKI 133
TcasGr62        NVVYVVTAWICAITKRDMFVEFLLKLVDFDSKLQ-----------TMNIKVNYHRVHRKI 408
TcasGr25        SQVMLIQFCAFVVVLKQKFCVVNQYIKQVCKLNN-----------GHYKNFLS------- 43
TcasGr59        SQVMLIQFCAFVVVLKQKFCVVNQYIKQVCKLNN-----------GHYKNFLS------- 218
AgamGr2         NSVCRIWYVMLVSAIRQRFNAMNAHMNAIAHGIL-----------HYKDQYAGEPDAD-- 139
AaegGr66a       NSVSRIWFVVLVQSIRHRFHVMNHHMDELAALLE-----------EHNDRWEDDLEIE-- 227
DmelGr66a       NTLDKIWFAVSLYALKERFEAINATLEELVDTHE-----------KHKLWLRGNQEVPPP 237
TcasGr11        ---LTLIVTLFTFLARGTLIKSFDMLSHVDVS---FIKAGFRLEYKQLLKRSYLIISFVL 154
TcasGr55        ---LTLIVTLFTFLARGTLIKSFDMLSHVDVS---FIKAGFRLEYKQLLKRSYLIISFVL 154
TcasGr21        SCCSVIFGCIFALK----VIEVFKNIEEVDVA---FRSLAVWVPYK-HLYVNILIHLSGL 151
TcasGr56        SCCSVIFGCIFALK----VIEVFKNIEEVDVA---FRSLAVWVPYK-HLYVNILIHLSGL 151
AaegGr          GLVALIMTFGYSVFRRQTVIDAFHALARTDLH---FKEIGVETDYKSTLFYNYGLLFIQI 155
AaegGr28a       ------MTFLYSIAQRHKLIAAFHSLARTDEH---LAEIGVEISYKKTLFRNYMVVLFQL 51
AgamGr33        GITALFMTFFCSIFQRNKLIKCFHALASIDRR---FKEIGMETNYKSTLHYNLLVMLTKA 153
DmelGr28bB      GLIAGAVIYTSAILKRCKLLGTLTILHSLDTN---FSNIGVRVKYSRIFRYSLLVLIFKL 155
DmelGr28bC      GFIGVTVIYLTAFVPNHRLERCLQKFHTMDVQ---LQTVGVKIMYSKVLRFSYMVLISMF 182
DmelGr28bD      GMTGMSILFLCSSIRVRLLIHIWDRISYIDDR---FLNLGVCFNYPAIMRLRLLQIFLIN 152
DmelGr28bA      GIIAAVFTFILPLLLRKYFLKSVKNMVQVDTQ---LERLRSPVNFNTVVGQVVLVILAVV 164
DmelGr28bE      GMLGIFAILLISLLNRKELLKLLATFDRLETEA--FPRVGVAMHQVAANKKMNRLVIILV 158
```

```
AgamGr25        VVSACICGTLSGIGGLHFVRELNRRLTEADEILSLYVKESTRSRRQRRKGILMLGLVCFI 147
AaegGr43a       VVSACICGVFCGVFGLPFVRELNQRLNEADDMLSMYFAAGKQD-MEKRKGLSMLTVVGVI 146
DmelGr43a       VVMAIVSGVYCGLFSLNDTLELNDRLNKIDNTLNAYN----NFRRDRWRALGMAAVSLLA 142
AgamGr37b       EFMVTNVAIILGLVLRNRVLEIFVQLAEIDEQLNVLRLP------IKHNRQHRLLTVLCS 143
AgamGr37d       QYLTMVFAIVFAWIVKHRISFILQTLHECDVQLSKISSP------IDHRKLHLKVSMLAV 139
AgamGr37a       NFFTTMYGVVNGYAVRDKIEKMLYKLHAVDQKISQWKRN------VDHRRFYRNVWAGVY 153
AgamGr37c       RYITVIVVQVHVLNNGGEINRMLRALNSISNAVTLLANRKGVSSKLRFFGTVRFAKGLRV 147
AgamGr37e       RYLTVLAVLLHSYINQNSVDQLFGSLNEVSSALVTLNTNHASEGSIGYFGTQRFARHLRW 147
AgamGr37f       GTLVYHLTMLINFLHRHRLRDLFQAFVNIDRELQQVGVR------INYRMHRFLITAGMV 143
AaegGr28c       RYVMSIVVSFFAYNCLEHSHRMLVKLDQASEIICRMDPLN-----QAPFGTVGSRGRLRM 142
AaegGr28b       GSFYIFLLAISSYRSFGSFWIMLDKLNQFDEWARQINAP------VCHRTQKRRLWKAIV 153
AaegGr28e       GSFYIFLLAIGSYRSFGSFWIMLDKLNQFDEWARQINAP------VCHRTQKRRLWKAIA 153
TcasGr14        KYIDQMAGYTGFLAMLTSMVMFYKRSNDLKTLLS--------NLESIQIYSIKPKERNSN 114
TcasGr49        KYIDQMAGYTGFLAMLTSMVMFYKRSNDLKTLLS--------NLESIQIYSIKPKERNSN 158
AmelGr4         ETFQALLGSSVIILTLLSYCIFQSVIKEIGNYLI--------RIELILHRLQQP--INQK 142
AmelGr5         EIFLIILGCFVMTSTLLFYCIFESVIIRIGNYLI--------NIENVLRHLQQP--LNRK 142
TcasGr16        LFLGTFIGRAKFLAILKQMRNLEPEFLKLDQLG----------ATVRNNRQIRR----MV 137
TcasGr22        MVLNLFFSQMYFTKKLEQMRQVHEVDNQLKQMGC--------DLDIPNSVVQKH----LI 58
TcasGr17        AVLSSGTTFKLVAKTLENMTKIDKDLTELGQKK---------YLEKRNRHIFRT----SV 133
TcasGr150       AVLSSGTTFKLVAKTLENMTKIDKDLTELGQKK---------YLEKRNRHIFRT----SV 133
TcasGr32        YVQNIYTVLTA-LTKKPLWFKLLKNLKMV---------------------QNHNNIREKS 113
TcasGr54        YVQNIYTVLTA-LTKKPLWFKLLKNLKMV---------------------QNHNNIREKS 113
TcasGr37        LILNVHTILTT-ITKRNQWCKLLENLKLD---------------------RENNSS---- 102
TcasGr53        LILNVHTILTT-ITKRNQWCKLLENLKLD---------------------RENNSS---- 102
TcasGr5         LALNISTILTT-VRKQQQWAQLIQNLKVV---------------------ATTKTK---A 109
TcasGr12        WLSNVNSLTLT-LKRKK-WNLLFKTLTHD---------------------EPRKSS---- 104
TcasGr13        WLSNVNSLTLT-LKRKK-WNLLFKTLTHD---------------------EPRKSS---- 178
TcasGr104       VALAFQVILNSCFCDPAKWIRLNNNLQYIDEVLK--------------NRDSHETNLLRN 134
TcasGr45        VALAFQVILNSCFCDPAKWIRLNNNLQYIDEVLK--------------NRDSHETNLLRN 134
TcasGr98        LMVLVSIILGS--LKTKEWAKLNNKFQYIDEKLK--------------TRDQKERNLFKN 132
TcasGr52        VMSAVTIILSSLIFNQEQWSSLNKNFQFIDKKLN--------------NRNSKSRVFYEN 73
TcasGr105       VAIPFVGIANSCILNQDKWRLLNNNFQQIDKIFK--------------TRDDR-VKVYQA 130
TcasGr4         SVLLVFLTYFFNILFRKKLLNAVKILSKIDETLNSKPLKP--------VKIRTKVYLVYC 125
TcasGr44        SVLLVFLTYFFNILFRKKLLNAVKILSKIDETLNSKPLKP--------VKIRTKVYLVYC 129
AgamGr9a        FFAVFCALPWQTYRHRDRLAAALNTLRQNE--LSLIDLT--GSVTDYRVVKLLAVVIVCN 140
AgamGr9c        LCTVLCSLPLQTFRQRDRLAAALNTLHQNE--LSLIDLT--GSVTDYRVVKLLVFVILSN 138
AgamGr9b        LCVALCAFPWQTFWQRKRLSAAMNILAQNE--INLIART--GVRTDYRIVSWLAIAIVCN 189
AgamGr9d        VCVVLCVLPVQTVGRRHQLAYFMNILIKNE--EDLVKLTSGGRSTNYRLVTLLATVCLWN 144
AgamGr9e        FIVAWFVVPLVSCARGDRLIYTLNNLLENDTDLSRLHAIQSSVRLSYRQAKWFGRMMQYE 139
AgamGr9f        FCIIWMVVPLYAYARCSRLVHALNSLQANDAELG--HVIT----LSYRRVKCFACFIQLD 134
AgamGr9g        SCTLCLILPLQCYYKHRQLSSSLNALLKNDSILRQAILEMGGKPLCYKEATIFARIMRWD 141
AgamGr9i        TNTIYIILPLQCYCKHHQLANSLNALLINDSILRRLALLVGKEPPCYTEATYFSFIMRWD 141
AgamGr9h        LVLTVLQMVYYGYTRKHVQLELLNSTIDIERELTVLLKR----KINYGVIARFTKWLTIW 144
AgamGr9j        YNLLIFCIIINAVYNRDCFVQLLNSLFARDDWMLESVAMQ----GSSQQR-RSTQSTGGL 176
AgamGr9l        YALMLVVIIWNAFYHRDSFVHLFNSLFAKEDWLLQWVVMK----GIVTKLNLQTRHNGSM 167
AgamGr9k        YTIVPFCVVLNNLFYRKRWTDLLNVLFA-DDSVLDT-----------TNVTMSGAYFLYI 159
AgamGr9m        FATVPICVVCNSFVHRKEIVQLLNMLFA-DQNVLDLGGTY----G-NKSVQPFSIANRYV 162
AgamGr9n        YVSVVMCTILNVLVKRQRITRLMNVLFR-PDRILDRCSP-------TNSADARYNDNRKL 134
AgamGr10        YVSVVICTILNVFVKRQRITRLMNVLFR-PDRILDRCSP-------TNSADARYNDNRKL 134
AgamGr11        YVSIVSCTLLNTFGRRHRYTRLMNVLFH-DDWLLDR----------YESAEMEYDNRRPF 131
DmelGr23aA      LGFALLLESVLKQKTHSQLEDLRVQTELQLQRLGMFGRSR-----HAAYLLPLIGVQFTC 134
DmelGr23aB      SHAFIVVTSFLDG---FRIKQDQLDEPIAFEDSDPWLAFT-----VLAMLVPTLGVEYLV 132
DmelGr39aA      DNAVILTTSVTQLLANLWLRSQQKSQVNLLQRLSQVVELL-----QFEPYAVPQFRWLYR 131
DmelGr39aB      DRLVMVIALGILVVQNAWLIWLQAPHLRIVRQIEFYRRNH-----LANVRLLLPKRLLWL 132
DmelGr39aC      ALINIRSCSIVTMLIYTQLYIQRFRFVALLQSILRFNQIS-----GSHREEGRFAFYYYT 135
DmelGr39aD      EAVIFRSTCVVLFLVYVILYAWRHRYRDLVQHILRLNRRC-----ASSCTNQQFLHNIIL 134
DmelGr98c       IVAIATCNQLNILLNYGRLGLIYDEIANLDLGIDKSSKNF-----CGKSHWWSFRLRLTL 149
DmelGr98d       VVAMFVWNQLNILLNFRRLARIYDDIADLEIDLNNASSGF-----VGQRHWWRFRFRLAL 147
DmelGr98b       YSIMAIANHLNMLINYRRLGGIYKDIADLEMDMDEASQCF-----GGQRQRFSFRFRMAL 147
AgamGr51        FILIVQIIFIRKKACLHVLLNEMFQLKHVLERMVARSML---EFRLYRLFLVKVTAVDIL 169
AgamGr52        FMIVVNVRMLSKAQRLASTLNALLAIRNTILQQWNCRVL---SREYGQLLLYKVLAIDMA 165
DmelGr94a       NITVAVINYVSQMIISDH-VAKVLSKVPFFDTLKEFRLD---SRSLYISIVLALVKTVAF 144
DmelGr97a       NVCVCLFNYVTQWEKTLQ-IIRFQNSVPLFKVLDSLDIS---AMIVWRAFIYGLLKIVFC 163
DmelGr93b       IILVMQFWFGEELINLVNRFLQLFRRMQSLTNSPKNRFG---DRAEFLLMFSKVFSLLFV 166
DmelGr93c       CIIVVQVCYEKELLRMIISFLRLFRRVRRLSSLKRIGFG---GKREFFLLLFKFICLVYE 156
DmelGr22d       GVVSICTVHLRTLWRSKHLEEIYNGLMLLEAKYFCSN-----AVECPAFDGYVIQKGVVI 150
DmelGr22e       SLSMVSIMLLRSFWKSGDIERTLNELEDLQHRYFRNYS----LEECISFDRFVLYKGFSV 151
DmelGr22a       SLITTLVTHLRTFSRSSELVEILNELLVLDKNHFSKLM----LSECHTFNRYVIEKGLVI 152
DmelGr22b       NVLSALIVHFMNFWGSRKVGEICNELLILEYQDFEGLN----GRNCPNFNCFVIQKCLTI 151
DmelGr22c       AVFSCVVIHFLNFWGSTRVQDLANELLVLEYQQFASLN----ETKCPKFNSFVIQKWLSV 148
```

```
DmelGr22f       TFFTISVLLLMNVWKSNTVRKIANELLTLEGQVKDLLT----LKNCPNFNCFVIKKHVAA 152
DmelGr36a       RMASGISAILNRWRQRAQLMRLVECVLRLFLKKPH--------VKQMSRWAILVKFSVGV 137
DmelGr36c       RIVTGLFTLILRWYQRCKMMDLASKVVRMYVARPQ--------VRRMSRWGILTKFIFGS 137
DmelGr36b       KIVAGLITVLNRWLQRGQMMQLVKDVIRLYMINPQ--------LKSMIRWGILLKAFISF 137
DmelGr59d       RVVCVFLELVSRWSQRRTFVRLFNSFRRLYQRNPD--------IIQYCRRSIVSKFFCVT 137
DmelGr59a       NYAAIAYTLISRCYRDAMLMDLQRIVLEVNREMLRTGKK----MNSLLRRMFFLKTFTLT 135
DmelGr59b       SFLVILYTVLSRGFRDTAFKEMQPLLLTLFREEKRCGFKGIGGVRRSLRILLFVKFFTLS 140
AgamGr26        YSMENGVVMMVLIGCHYQRDCYRHYGEQLLAVAVDLSL-CGGTVDFPRIETIFNRVLACI 155
AgamGr27        NVIVNVIVLFAMLECQQSRPFYASILKELFELGKNLHD-CDASINFPHVEAITDRLLVCA 156
AgamGr29        TAIQTSTMGYILLVYQFRTNFHRYYFDRLVGVLEQFGQ-TDINIRLGALQTNIRRLLPII 160
AgamGr31        ALLQMGSLSYVLLVYQFRSSFHRFYFDRLVCVLEHFGR-RDIDKSLSALQTNMCRLLIAS 160
AgamGr30        NWLKIVTVLVAVLRSQTSTAYFRETVNSLVQIMVQYDQGWRIERKLASVATVSKRLSVVF 159
AgamGr32a       NWLRVVMVLIALVGPRLSGRYFRETIDTLVHIMKLFDRATKIESILTAISVITNRLLLLY 159
TcasGr2         TGVMASLLFLNLAKRWPKFVKDWCVVDATFASYGWP--------KGLNKKLNTLTVVFML 145
TcasGr9         TGVMASLLFLNLAKRWPKFVKDWCVVDATFASYGWP--------KGLNKKLNTLTVVFML 145
TcasGr7         SGLASGYLYLKLAMKWPRFMKDWSCVEVMMASYGWP--------AGLNRRLNVLLAVFMS 162
TcasGr30        SGLASGYLYLKLAMKWPRFMKDWSCVEVMMASYGWP--------AGLNRRLNVLLAVFMS 162
TcasGr29        SGLASGYLYLKLAMKWPRFMKDWSCVEVMMASYGWP--------AGLNRRLNVLLAVFMS 162
AaegGr64f       VIITAMIEFIILARNWPLIMQRWTADEDVFLSNPY---ETG-QYLPLESLVKRVAFTIIF 118
AaegGr64a3      VIITAMIEFIILARNWPLIMQRWTADEGVFLSNPY---ETG-QYLPLESLVKRVAFTIIF 118
AgamGr14        IIVFFMVELMLLARNWSQIMGRWYTDEAPFRTDPY---RPPSRTLPFRRKVHLIAFGVMF 182
AgamGr18        VVYLSAWLFFFIALRWQSILGAFSRCERSFLSDHYRRQSK--GRFALGWKIRFTGFAIFA 153
AaegGr64d       FNYIITVLFLLIAIQWQKVLKEFANCERLMLKDAYTKLTERVTRFNLAWRIRMVIAGIIV 185
AgamGr17        LNLSGAIVLLAIAARWRTIMEKWRSLEENFLHPPY----AERRFWSLKRVVAAIGSTMVV 143
AaegGr64c       LNFSGSVCFLIIATKWRNIMLNWKMYEEVFLHKPY----LMK-GRSLKFKVRMVGGGILL 168
DmelGr64c       ICIFCFGVFLLLSRRWPYIIRRTAECEQIFLEPEY----DCSYGRGYSSRLRLWGVCMLV 147
DmelGr64d       ICIFCFGSFIKLSRRWPHIIRETALCERIFLKPCY----ANQEGLNFTRFLRRWALILLV 156
AgamGr20        MCTLLQLLFVRLAQAWRGFMVYWAEREEMFFARPYG----AIN---LRRKVIGLAVCILT 174
AaegGr61a       VCATSAVLFWALAKEWQFVVTVWSETERVFLRKPFR----GKA---LRSSIRRTAFVVLT 168
AgamGr21        DACLINVLFLNLATKWRSVAMKWDEVDDTFNRPPYH----MQS-WSLRKRLGVVSFTLVF 196
AaegGr64a       DGCIINVLFLLMATKWNRVAVKWDSVDRIFLTESYR----IESKWTLKRRLWTATALLLG 76
DmelGr64a       CVLSAYVVFIRLAKKWPAVVRIWTRTEIPFTKPPYE----IPK-RNLSRRVQLAALAIIG 191
DmelGr61a       VGMVNWLNFVGFARSWSHIMLPWSSVDILMLFPPYK----RGK-RSLRSKVNVLALSVVV 174
AgamGr15        YNLYAMYRFGRLGQRWPALMVKWARVDDSLPPQKGL----FER-AVLAYRIKLCSIMVMA 183
AaegGr1         YNLYGMYCFVRVAQKWPVLMQKWFNVEQLLPQSSNI----IER-GKLANKIKLISILVIT 188
DmelGr5a        SILIASWQFLNLAQLWPGLMRHWAAVERRLPGYTCC----LQR-ARPARRLKLVAFVLLV 185
DmelGr64f       SVLLVLIVALNLARQWPQLMMYWHTVEKDLPQYKTQ----LTK-WKMGHTISMVMLLGMM 209
DmelGr64e       SIFLISLSFFQLARKWPEIAQSWQLVEAKLPPLKLP----KER-RSLAQHINMITIVATT 202
AmelGr1         TNLIASFLFLKLAMRWPCLMVTWEKLEKELSQRHRK----ISK-ISLSMKFKIVTIVVMT 129
AmelGr2         NSLVGSILFFSLSSRWVSLQYEWRAMERYIDS--NS----TEP-TRLRWKFFIISTMVLV 217
AgamGr16        LNLLISINFAVLARSWPQLMSRWVQLEQSLPDQPRL----SAACRRNARQVGLVATVLLT 158
AaegGr64e       INLSITIGFTVLARRWSAMMTEWENTEQSLPFRPQL----TASNRRVRRKVITIMVTLML 176
TCasGr6         ---------------------------------------NLQFPLLSTLIIIISLGCLI 138
TcasGr26        ---------------------------------------NLQFPLLSTLIIIISLGCLI 138
TcasGr33        ---------------------------------------NLQFPLLSTLIIIISLGCLI 138
AgamGr23        PHFWIPFVGWGVAKQVAIYKTMWGAFQVRYYRVTGT----SLQFPHLKLLIVFLSIGCLV 239
AaegGr21a       PHFWIPFVGWGVAKHVAVYKTMWGAFQVRYYRVTGT----NLQFPHLKILIVMFSIGCLV 231
TcasGr10        PHFLLPVAAWTNGTEVAKFKNMWTRFQLKYYQVTGT----PIIFHNLTLITYSLCVISWA 187
TcasGr39        PHFLLPVAAWTNGTEVAKFKNMWTRFQLKYYQVTGT----PIIFHNLTLITYSLCVISWA 187
AgamGr22        PHFLLPVASWRNGSEVAKFKNMWTDFQYKYLIVTGK----PIVFPKLYPITWTLCIVSWS 212
AaegGr21b       PHFLLPVASWRNGSEVAKFKNMWTDYQYKYLMVTGK----PIVFPKLYPITWVLCVVSWA 212
DmelGr21a       TNFLLPVASWRHGPQVAIFKNMWTNYQYKFFKTTGS----PIVFPNLYPLTWSLCVFSWL 213
AgamGr24        PILIIP-LMWYESRKVVSVVNGWVDFETVYRETTGR----ALELR-LRTKAQVIAILLPI 138
AaegGr63a       PVLIIP-LMWYETRKVSSLLNQWVDFEAIYRKTAGR----ELELS-FRTKALLIAILLPV 203
DmelGr63a       PIMIIP-ILWYEARKIAKLFNDWDDFEVLYYQISGH----SLPLK-LRQKAVYIAIVLPI 232
TcasGr3         PIIINP-LVLYEARKLANVVTDWVNFERIYYKLTKK----KLSVF-FGNKPVILTVVLPL 194
DmelOr83b       TAWTTITFFGDSVKMVVDHETN-SSIPVEIPRLPIKSFYPWNASHGMFYMISFAFQIYYV 206
AgamOr7         VAWVTITFFGESVKTVLDKATN-ETYTVDIPRLPIKSWYPWNAMSGPAYIFSFIYQIYFL 204
HvirOr2         ISWVTLTFFGESVRMITNKETN-ETLTEVVPRLPLKAWYPFNAMSGTMYIVAFAFQVYWL 207
TcasOr16        IAWTGITFVGDSVHNIKDPENENLTITEPIPRLLVKAWYPWDAMSGMPYYITLVFQIYYV 306
TcasOr22        IAWTGITFVGDSVHNIKDPENENLTITEPIPRLLVKAWYPWDAMSGMPYYITLVFQIYYV 345
AmelOr2         ISWTTITFIGDSVKKVIDPVTNETTYVE-IPRLMVRSWYPYDPSHGMAHILTLIFQFYWL 204
TcasGr31        EHLLIQCYIAVSIFGSSSFEADLRQFYKTAYSAIFT------------------------ 79
TcasGr35        IVVILKLILAKYRQQDVTLLQITKAKNLLYSKQ--------------------------- 90
DmelGr59c       RISAVLLSLITRWYQRSRFIRIWNQILALVRDRPQVVRG------RWYRRSIILKFVFCV 139
DmelGr92a       SIPNVAVILCYHIFRGPEIIDLINQFLRLFRQVSDLFKTKTPGFGGRRELILILLNLISF 151
DmelGr28a       GILAMSTIFGFAMFKRQRLVSIIQNNIVVDEIFVRLGMKLDYRRILLSSFLISLGMLLFN 154
DmelGr64b       VCLLEHLFFWRLAIQWPRIMRTWHGVEQLFLRVPYRFYG----EYRIKRRIYIVFTIVMS 140
TcasGr71        SCYTVLAPVLCKRQQYCQLMSKLLGNHQLVYNCHTFG-------RFLAPNLTYLLVALYS 129
AgamGr19        DVIIIMLLLLRVAYRWPTVVPKWEQIESLEVMQYNARQQNARSCRRIRAIALLLIVLGFA 140
```

```
AaegGr64b       FWMFFATVEHCLASSVQVYIQYQESLRCNWIYSNAMKN---------------------- 115
DmelGr93d       SIIHLKICHGPEVTKLVNQYLHIFRLGTLDIRRRSQFGGGRELFLLILSVCCQIHEYVFI 155
TcasGr34        DFIAYFFNFWTILFSRSDFFQQFECDNEPKLRHY-----------LIFIFVNVFFWVIIL 111
TcasGr43        NYYVIVVTTFYKRRTWTRLLKNLESCAKIKKTKR----------YCYYYYAFFGVHLAYG 128
TcasGr47        NVYQILAYNFWKKKYWSEFLANLKLLKGRKIRKN------------LYYLLLFVINVLYV 112
AmelGr3         VMSLTVVCIVGSPFRWKYLQLVINKLIEVDEKIGVSSAKVARRFTIVLTICSLSYLWFNS 163
AgamGr32b       SILQTLTTGNMMIGCEQRRAEYEGYFEEVLHLMKETAHQPDCKTTIWYRHVTKVLLALYC 159
AgamGr50        MYVVTLTSRLKTLANRERLCQLLNALLVLREQVLQGSTKAATFQQGLARSLLTKLIVFDL 145
TcasGr20        VAVTVLAGILTTPFKLPYFWKMLTGFGKIEQVLPPHHAPEEKKRSFWAVTIFLVLAASVI 152
TcasGr79        SVIYSLLRVKRMMRSWYYLLTNLTYNNTNRKYYWLN---------VCCLNLSIVTIFFNF 131
TcasGr15        AYHVFVTMMTLKRHKWSTLFEILLKLESKDLSRTFYG------FVIVAHLLFLVIFVLNI 133
TcasGr60        KIKRMVLLHKTLVRVAKSLNQVFAPQLLLWVLLG---------VMVVTVDLHVFFFTLFN 127
AgamGr38        CYASTIYGLISCFINRHDIWKFYNEAYAVDNMLEELG-------ERTNFTRSFALFVAYY 152
TcasGr51        TSVVKSLESRYADLNENFLQCQDEKFESFVYYHRILGETVDLFNSLFGYQTLLITFHIGS 384
DmelGr39b       VIILQVMVHYDDYFRFCREMKYLGLRLQCELKIHVG-------RLKWQSYAKILALGIGF 130
AgamGr28        NLCITVQTMLRGYSETVRYSQCLERIVTVAATVSSSE------WTELLLYLRLRLYWFGI 145
TcasGr61        SFLMVHQGETQFVAANVILKTRFKTINNILENLWAKKLIVVKDKKKSRSDEQTIDICMRC 238
TcasGr50        GITIQTLGIISNCINAKQITIFFDKIQRIDKQFGLLGYSINYRRISIFVNLAILGVFGEF 137
AgamGr12        LPVTIVYMHLIAFTKRVRITALFNALFTDRSWRFFGDRSNSWYESSRWLGRFATAITIGT 143
AgamGr36        GAIVMIVLAIDNFMRRDATCQIIGELFKVDRYLQRIGHQLDHRRQYVTLVRILFVVLMLI 150
AgamGr48        RYGTVVLCFYQLLRHEVSLHRYVQRFVVIGKSVIVRCNSRSYASVQRMVYALVAKMLIAD 145
AgamGr49a       IYPCAYIVLLTDHEFPILALSYYIIMLQFTLNYIIVGVLYLHVLFDPAPLLHVLNELIER 162
AgamGr49b       LALIYYHLLPARPVIRNIFNGFVQLFRTLNGAGCHRWKGKSTKKNVLKLALKMILCDTLL 149
DmelGr2a        MRVAHLAALLEALWQRQAQRGFFAELGEIDRLLSKALRVDVEAMRINMRRQTSRRAVWIL 154
DmelGr32a       CILTYTLTVFVCAHNTTSMLRIMNEILQLDEEVRRQFGANLSQNFGFLVKFLVGITACQA 196
DmelGr68a       CIISYTMVVLSSVQNASRHFRTLHDIAKIDEYLLANGFRETYSCRNLTILVTSAAGGVLA 147
AmelGr7         IITRIKAQQLRTIVSLVELSDQKMENIGLSPKHRCLMMYQIKRYIFLGIYTLIFVVLIYH 151
AmelGr8         IALFGPAVFICSYASNRSTLQAIQRVQHVSHALSTEIFSKLIKTLLIKDMSFFMPLGTFI 159
AmelGr9         GIITMHILSSTRLCMIQNLLKISSILSEKDFNDLAKR--------IHKKDILGFLFILIH 151
TcasGr57        NVSLRIILVFSKGKVIKSFFNQGYELSKQEIFNCCSKKSFRMNFAIFNLYMVLLLLFDAY 133
TcasGr41        TSTSILLIVNAVFVKEKRIRTLLIKLSEIKENTRLAEK-------QARISILELVAVHLV 139
AgamGr34        AILLMPAFRLRNFAEVTRALRKLEQDLQQDAIPTNCRQMARWNIGIVVGTVTVLLLATAF 148
AgamGr35        AWVVPQLKADELGSILDGFLLIDRELSSYNVHEVAGYKRVSFLLRYGVVLLALMSLTVYD 177
AgamGr39        QLIALIGIPITNYVQAYKYDRLIYCIAEVDKGLEELGFKHDYSVEYFYSTVYLSVTLGLQ 149
AgamGr40        GIIISLSISLCNRFVVGRMFRMFKTLDGVDQILKRDGYRLNHQLNHLLSCIYMAVPILVN 144
AaegGr28d       VFLFSLFMAPKYRLRTQGMFEILANIAELDGQLRSLGLHIDHQRHHFISTVSAGLWMTMG 147
AgamGr42        TLILYLLAGFNCSVSATLVLLFRKKFLHMVQLVEEADFLFSKYFYEIQHSKHHLVCVALL 163
AgamGr41        PFAMMLFVPWIFLFRKTKIFYISRDLDIFDALVNETFLCFVRTSCFLFSAIIFLFLQIAK 160
AgamGr43        GVLATIICYASLWRTDRMKCIYIEGIQGSNVKINDRYYQFRNMQIPIKYSILVPFSIGML 153
DmelGr33a       VKHSVAYHFTWLFLFCVFTFALYYDVRSLYLTFGNLAFIPFMVSSFPYLAGSIIQGEFIY 185
AgamGr44        VLQTFLSAVVRRYHKRYEIAQLMRDLFQLKRELFSATELSQRWIKGHILRKITISQLIVT 148
AgamGr13        CVLIAVQQLLFLETYRQLLTDLLAGVRALARRTTVALGEQVQLLVQLVLLPCATYTLHVS 144
AgamGr45        GCILVLGIVMVSLVRQPAFSRLIQVLLSVDRAIPEMVRTAGLSAITKVKVNNIVWLRNVL 182
AgamGr46        NFVSIVIDVYNRYSGLILFWLLHAVALANHRSLTAIVLGVLLLDEQIADRLSVVPKHGQW 164
Dmelr93a        VSILAVISFVIQARGENQFREVLNRYLALYQRICLTTRLRHLFPTKFVVFFLLKLFFTLC 159
AmelGr6         NYLVAMHDLIFTIFTRKTFSRIWNAQQDFDERLSQLGYPRKETKIKIAAWILLASQIVIW 199
AmelGr10        CLFTDASVTTLWNWKIRSVLSQLRNFDRATKFRDFSKG------NKLRIICHVTMFVSFS 135
TcasGr28        AFVMGLFCILKFALDGLMLDKTGICVSNVFDFVH-------------------------- 111
TcasGr40        HSFMTSVLFSQVASRWPLFLNEWTKIEINLLKHYQS-------TTDLHKKIRYSAFGMIF 153
AgamGr3         GLLTIFVTVADSWFEVETHRSIWLRYRALATRNGTILGLIGRDELARVLLRYFFAILTIV 153
AgamGr4         LTLSVFAIFIDTVLQRNTHRLVWEKIALIRLSARKVYVERFTRHYLWKFYGYLAVCAFLE 177
AgamGr6         PLAVPLVTLTLSLLKRSTQKRIALLMDQIDGSIEAHGAAPGCLERFNHQLADDIFSTMFV 173
AgamGr7         TDRAASSLARARTHFRNKLLLFCAFCCAIELGILALVYSDPVQLALWCLTVPSLVIIRLK 168
AgamGr47        MNISLLALINEYIYFTVHIRRLFMIVNRLLKQLLLGDTNESNETVLLDQNRKSKTPTIAY 163
DmelGr8a        GVLAIYLETLSSQRHLANFWWLHFKLGGQKTGLVSLRSEFQQFCRYLIFLYAMMAAEVAI 145
AgamGr5         IGLSILVMYVELFRTSDNICGCWRLLYKAHQTLQRYGMVDHRRLTRVMRLYWWFVLGSLL 189
AgamGr8         MVFTYYSLFLESGYQRGVLYKVWDELERLHNIFPSAKWALQPQAHLRTVALFVVYMSWWE 134
DmelGr9a        YKMIHAWIALSALFECRRFRYLLEELPPVKATS-------------FIYRHLILEIILFA 122
DmelGr59f       YVVHIFSIMLLTWQCRNWAPKLMTNIVTSDLNRAYTIDCNRTKRFIRLQLFLVGIFACLA 167
DmelGr77a       LLVCAFMTLWIHCFKQAEIIGCLNRLLKCRRRLRRLMHTRKLKDSMDCLATKGHLLEVVV 174
DmelGr85a       VFFALLSSMGSCYQWQNRILAVLKELKHQRDLSRHMGYRVPRSKQNSIDYLLFALTVLLI 162
TcasGr27        FTNIVNLTHLLETQYAKIRTENQKKIFDESVIFER----------KVMKNFAILIIPTCV 134
TcasGr48        IIITILDIEILGYVNKAKFGKLLINLYTINYNFKESE-------RNDYVSLLQLVFVFCY 149
AgamGr1         VAIAFNVICTIRIAWPASQHSLAQCWAQLIAKVQEIQIMFQCSIESKRPFQLFWLWIAVL 151
DmelGr10a       TAIKYATVIVTYVANTVHFEAINQRCTMQRTHLEFEFKNAPQEPKRPFEFFMYFKFCLIN 154
DmelGr59e       LVYYAVLNRPLAHGAELQIERIITGLKGKAKRLVYKRHGQRTLHLMATTLVFHGLCVLVD 139
DmelGr10b       QLLMAVTGTWLHFLFERHVCQTYNELSRILKHDLKLK---------EHSRFYCLAFLAKV 139
DmelGr89a       DVCISLALLGNHLWNRRELANLVNDLARLHRRRRLS---------WWSTLFLWLKLLLSL 137
DmelGr57a       STLVFGVTVISLQVFARRHLGIYQRLAALDARLMSDFGANLNYRKMLRKNIAVLGIVTTI 154
DmelGr47b       CSVTYIEYCLSLDLQKDRHLKLVARMQEFDRSVLMVFPHVQWNYRRARLKYWYGTVIVGF 152
```

```
DmelGr58a       RIMAMFSGVLLMWFRRKRILNLGENLILHCLKCKTLDNRSKKYSKLRKRVRNVLFQMLLV 139
DmelGr58b       RAIAVVSCYGTLWLKRHKIIQLYKYSLIYWKRFGHITRAIVDKKELLDLQESLARIMIRK 149
DmelGr98a       GHAIIVLELLWGNCSKDVDRQLQAIHSQIKLQLGTSNSTDRVRRYCNWIYGSLIIRWLIF 143
AaegGr93a       NLLVTAVIYWRQFMCNERIQQLNRNFIRFADEHMCSTAIKCQQLWYQTVLLAYLMVSNAG 152
TcasGr19        IIFTVHFLLCTYYFYYAFIILLCVYYFYYAFIIFTVHLLFLLCIYYFVVPLFFLLCIYYF 149
DmelGr47a       IGGTMACCVKSCWEKAQGIRNLARGLVTMEQKYFAGRPSGLLLKCRYYIKITFGSITLLR 133
DmelGr58c       KLLTMLFLMSSVWIQRRRLYKLGNDLMKMLHKFRFNLGNDCRNRCLCKGLLTSSRFVLLT 143


TcasGr38        LCYLLARY--------------------------------------VALALVILFSEILV 173
TcasGr46        LCYLLARY--------------------------------------VALALVILFSEILV 155
TcasGr1         RIHLFVRY--------------------------------------VFVTSYLLFDYFVQ 154
TcasGr123       MVQSLGRV--------------------------------------GVLVVIIGTLAIIA 155
TcasGr62        MVQSLGRV--------------------------------------GVLVVIIGTLAIIA 430
TcasGr25        ----QVEI--------------------------------------IHNEILVTHNEIQT 61
TcasGr59        ----QVEI--------------------------------------IHNEILVTHNEIQT 236
AgamGr2         IAEIPLDY--------------------------------------LEKEIFTVYTQRRK 161
AaegGr66a       KNDIPMNY--------------------------------------LEKEIFTIRSHKRG 249
DmelGr66a       LDSSQPPQ--------------------------------------YDSNLEYLYKELGG 259
TcasGr11        FSLLAR----------------------------------------VPVMLMTISADFIQ 174
TcasGr55        FSLLAR----------------------------------------VPVMLMTISADFIQ 174
TcasGr21        VTIVAT----------------------------------------LTVTIIFFASYQYG 171
TcasGr56        VTIVAT----------------------------------------LTVTIIFFASYQYG 171
AaegGr          TVNLAY----------------------------------------ITISIAIVTSSGVF 175
AaegGr28a       IVLFSY----------------------------------------FATTMSIVLISGFP 71
AgamGr33        IISTLY----------------------------------------LVVCLAVFISSDTY 173
DmelGr28bB      LILGVY----------------------------------------FVGVFRLLVSLDVT 175
DmelGr28bC      LVNVLF----------------------------------------TGGTFSVLYSSEVA 202
DmelGr28bD      GVQLGY----------------------------------------LISSNWMLLGNDVR 172
DmelGr28bA      LLDTVL----------------------------------------LTTGLVCLAKMEVY 184
DmelGr28bE      GSMVAY----------------------------------------ITCSFLMISLRDTT 178
AgamGr25        ITGALA----------------------------------------LDIWVWYRIADKIK 167
AaegGr43a       ITGALA----------------------------------------LDIWVWYRIAEKIK 166
DmelGr43a       ISILVG----------------------------------------LDVGTWMRIAQDMN 162
AgamGr37b       IVLG------------------------------------------SFLFLLLVTISTVT 161
AgamGr37d       GIVT------------------------------------------SYMLLLAIHLPLIL 157
AgamGr37a       LIAI------------------------------------------VLLIYIYGTIITCQ 171
AgamGr37c       FSLS------------------------------------------YPFVCLAITFTILK 165
AgamGr37e       LMLV------------------------------------------YPLMFVSMTGFVER 165
AgamGr37f       CFLSGIFV--------------------------------------TSALVYAYKITVLN 165
AaegGr28c       VLIVSIVS--------------------------------------PLIGSMVMNKYMVH 164
AaegGr28b       VIIL------------------------------------------LLTFVVISTGFLVS 171
AaegGr28e       VIIL------------------------------------------LLTFVVISTGFLVS 171
TcasGr14        HWVRSGLF--------------------------------------ALITGNVLFYPFLP 136
TcasGr49        HWVRSGLF--------------------------------------ALITGNVLFYPFLP 180
AmelGr4         YIFNLLFF--------------------------------------VCLFKFIIFVALLF 164
AmelGr5         HIFNVLFF--------------------------------------ICLFMLILLIILLI 164
TcasGr16        VFLIGVSL--------------------------------------SFNLFGDLLTLFIK 159
TcasGr22        LLMVLVNL--------------------------------------IFNTCGEVFSAWVR 80
TcasGr17        LLIFVMHL--------------------------------------MFDTLGGLYSTWSK 155
TcasGr150       LLIFVMHL--------------------------------------MFDTLGGLYSTWSK 155
TcasGr32        HYCLFVAS--------------------------------------NCFFWSYQSYMSYI 135
TcasGr54        HYCLFVAS--------------------------------------NCFFWSYQSYMSYI 135
TcasGr37        ----FVVA--------------------------------------NVLFQLIHVYYTVV 120
TcasGr53        ----FVVA--------------------------------------NVLFQLIHVYYTVV 120
TcasGr5         WFLPFVVT--------------------------------------NIIFVLFHTYEAFV 131
TcasGr12        -LVQLILT--------------------------------------QAIFLMFVSYNIYV 125
TcasGr13        -LVQLILT--------------------------------------QAIFLMFVSYNIYV 199
TcasGr104       VSVQFFVY--------------------------------------VILYILATVFLAYV 156
TcasGr45        VSVQFFVY--------------------------------------VILYILATVFLAYV 156
TcasGr98        AYFQLVLS--------------------------------------ISVYSSSVGYTQYV 154
TcasGr52        VNFQFGSI--------------------------------------LALFGVVQIYIFSG 95
TcasGr105       PVLQLFLY--------------------------------------IFTYLSVTCYVTYA 152
TcasGr4         SSALVLAA--------------------------------------MTTFHLIYEAHGFS 147
TcasGr44        SSALVLAA--------------------------------------MTTFHLIYEAHGFS 151
AgamGr9a        TVCFHIIF--------------------------------------QVYYITKTHSPDEL 162
AgamGr9c        GVIFHVFF--------------------------------------HAYYIIKNTGSDTY 160
AgamGr9b        GMCLHLFF--------------------------------------QVFYIVQYGGMGKP 211
AgamGr9d        GLLFHVFF--------------------------------------HFYYIDEFRHFGDR 166
AgamGr9e        SIACCLLL--------------------------------------SAGYTILYSFQP-E 160
```

```
AgamGr9f        GVLCCLLL--------------------------------------STGYTIAFWYQG-Q 155
AgamGr9g        GIVCVSFF--------------------------------------FCSYFIKSWSKC-N 162
AgamGr9i        GIACVSFF--------------------------------------FCSYFMKNWVID-E 162
AgamGr9h        TIFFYVII--------------------------------------MHTLYVFDHLRYNT 166
AgamGr9j        VCLIVLVL--------------------------------------LYAMYNALFVNDHS 198
AgamGr9l        GTLALLVI--------------------------------------LNSFYNFAYAANST 189
AgamGr9k        SVLSILLV--------------------------------------TLELCNLFALETLS 181
AgamGr9m        KVLYAIVA--------------------------------------LICFYNYINMIHTM 184
AgamGr9n        LVFVLFIL--------------------------------------FGMCMKLSYYKKVE 156
AgamGr10        LAFALFIL--------------------------------------FGIFIKLFHLKTAE 156
AgamGr11        AGLLFAML--------------------------------------TMMSINMFYHRDMN 153
DmelGr23aA      DLVRLATN--------------------------------------FGETVSPVFCISLP 156
DmelGr23aB      CSN--APE--------------------------------------YAFRIRIYHLKTLP 152
DmelGr39aA      IWLLVCLI--------------------------------------YGAMVTHFGINWLT 153
DmelGr39aB      IIATNVVY--------------------------------------MANFIKTCIFEWLT 154
DmelGr39aC      HLSLLIIC--------------------------------------MLNYAYGYWTAGVR 157
DmelGr39aD      YGMLTILC--------------------------------------FGNYLHGYTRAGLA 156
DmelGr98c       SIGLWMVI--------------------------------------IIGVIPRLTLGRAG 171
DmelGr98d       SVGLWIVL--------------------------------------LVGLTPRFTLVALG 169
DmelGr98b       CVGVWMIL--------------------------------------MVGSMPRLTMTAMG 169
AgamGr51        MQLLALYT--------------------------------------FEDSTYTGRVDSVG 191
AgamGr52        LLLFSLIM--------------------------------------YYVTQDEVPTG-AE 186
DmelGr94a       PLTIEVAF--------------------------------------ILQQRR--QHPEMS 164
DmelGr97a       PLITYITL--------------------------------------ILYHRRSISESQWT 185
DmelGr93b       FM----AF--------------------------------------RLMLSP------WF 178
DmelGr93c       LYSEICQL--------------------------------------WHLPDS------LS 172
DmelGr22d       VVGLLAPW--------------------------------------MVHFGMPD--SKLP 170
DmelGr22e       VLELVSML--------------------------------------VLELGMSPNYSAQF 173
DmelGr22a       ILEIGSSL--------------------------------------VLYFGIPN--SKIV 172
DmelGr22b       LGQLLSFF--------------------------------------TLNFALPG--LEFH 171
DmelGr22c       IGLLLSYL--------------------------------------SIAYGLPG--NNFS 168
DmelGr22f       IGQFVISI--------------------------------------YFCLCQE---NSYP 171
DmelGr36a       VSNFLQMA--------------------------------------ISMESLD-RLGFNE 158
DmelGr36c       ITDGLQMA--------------------------------------MVLSAMG-SVDSQF 158
DmelGr36b       AIELLQVT--------------------------------------LSVDALD-RQGTAE 158
DmelGr59d       MTETLHII--------------------------------------VTLAMMRNRLSIAL 159
DmelGr59a       YSCLSYIL--------------------------------------AVFIYQWKAQNWSN 157
DmelGr59b       WLCVTDVL---------------------------------------FLLYSTDALIWVN 161
AgamGr26        ALFFAGVL--------------------------------------TVDLLYNDLVVWRF 177
AgamGr27        VLYFSTSS--------------------------------------LVDWWADYDPSCTF 178
AgamGr29        PLHIALVL--------------------------------------AAYILHDQS--WGV 180
AgamGr31        LVFVTLVF--------------------------------------SALVVRDHS--WSN 180
AgamGr30        LLDDFTIA--------------------------------------TGLYFIVEG--APA 179
AgamGr32a       GLHALIIT--------------------------------------VTVWISTEH--PVS 179
TcasGr2         IALGEHWLSRYSWAKVFLVEHILVQTNKLVLALECNNTTA-EGFTYFLGNMSFAHIFALV 204
TcasGr9         IALGEHWLSRYSWAKVFLVEHILVQTNKLVLALECNNTTA-EGFTYFLGNMSFAHIFALV 204
TcasGr7         LAL---------------IEYILMQTNKLVLALECNNSTS-EGFDYFFGKMSYSHIFSLM 206
TcasGr30        LAL---------------IEYILMQTNKLVLALECNNSTS-EGFDYFFGKMSYSHIFSLM 206
TcasGr29        LAL---------------IEYILMQTNKLVLALECNNSTS-EGFDYFFGKMSYSHIFSLM 206
AaegGr64f       FAFVED-----------TINFISAYLLNVVHMKYCT-HAT--DFWRNFFRREHAYIVRFI 164
AaegGr64a3      FAFVED-----------TINFISAYLLNVVHMKYCT-HAT--DFWRNFFRREHAYIVRFI 164
AgamGr14        LAFVED-----------TLNFVSAYRLNELHIRYCP-HTA--GFWKNFFHREHPYVLRVI 228
AgamGr18        LALVED-----------WLNYYSAYQSNIVQIATCNRTNV--TVWQNFYLREHPHVFHWL 200
AaegGr64d       LAFFED-----------FLNFYSAYQDNYVQMDYCNRTEI--SFWENFYIRDHPQVFQYV 232
AgamGr17        LAFVED-----------TLHVASVYYTNLQYFKRCD-NST--PFWTLFYQREHPKFFHYL 189
AaegGr64c       LAFVED-----------LLHMLSCCRTIEVYIERCD-NSS--SFWETFYTREHSKTFDYI 214
DmelGr64c       AALCEH-----------STYVGSALYNNHLAIVECK-LDA--NFWQNYFQRERQQLFLIM 193
DmelGr64d       AALCEH-----------LTYVGSAAWSNYVQIRDCN-LKV--GFVENYFLRERQELFSVF 202
AgamGr20        SALVEH-----------VVYVINQAYNVYQESLTCQYNVT--NPLKLYGTLTFGSVYQSV 221
AaegGr61a       LALAEH-----------IFSVANNIANLRREVHHCNWTIS--SPVKYFCLKTFSSTFDSI 215
AgamGr21        LAAVEH-----------ILSIVSNVHNQMVEIKYCNWTE--PNYFQHYSLRRFANIYLNF 243
AaegGr64a       LACCEH-----------LLATINNLNDQWHEIEHCGWKENITDTFRHFSLRKFSNMYSIV 125
DmelGr64a       LSLGEH-----------ALYQVSAILSYTRRIQMCANITT-VPSFNNYMQTNYDYVFQLL 239
DmelGr61a       LAVGDH-----------MLYYASGYCSYSMHILQCHTNHS-RITFGLYLEKEFSDIMFIM 222
AgamGr15        LSLSEH-----------LLSIVA---AVHYSNNCPAVHD----PYEAFFKSNFAFVYYYF 225
AaegGr1         LSLMEH-----------MLSIVA---AVYYTPNCPNIKD----PVKMFFKSNFLFVFYYF 230
DmelGr5a        VSLMEH-----------LLSIIS---VVYYD-FCPRRSD----PVESYLLGASAQLFEVF 226
DmelGr64f       LSFAEH-----------ILSMVS---AINYASFCNRTAD----PIQNYFLRTNDEIFFVT 251
DmelGr64e       CSLVEH-----------IMSMLS---MGYYVNSCPRWPDR---PIDSFLYLSFSSVFYFV 245
AmelGr1         FALVEH-----------SLSIIH---GYFKAKECIEFHRE-QSILGVYFQMQFPQIFSRT 174
```

```
AmelGr2      LSLIEH-----------VLSIFNNIDGYEWNESNSTFHN----FLEIYTLRSHSFIFDTL 262
AgamGr16     SGLIEH-----------VLSKPA----GLHRAYRCPIPN----LLEAHYKQAFPEMFSFV 199
AaegGr64e    SALFEH-----------ALAKPS----GLYRAYKCGIKD----LLEAHLMQAFPEMFSFI 217
TCasGr6      LA-------------------------------------------VVFLLTLSALLEGFT 155
TcasGr26     LA-------------------------------------------VVFLLTLSALLEGFT 155
TcasGr33     LA-------------------------------------------VVFLLTLSALLEGFT 155
AgamGr23     CA-------------------------------------------IVFLLSLSFLLEGFA 256
AaegGr21a    CA-------------------------------------------IVFLLSLSFLLEGFA 248
TcasGr10     VG-------------------------------------------IGIMLAQYYLQADML 204
TcasGr39     VG-------------------------------------------IGIMLAQYYLQADML 204
AgamGr22     LS-------------------------------------------LVIILSQYYLQPDFQ 229
AaegGr21b    VS-------------------------------------------FVIIMSQYYLQPDFQ 229
DmelGr21a    LS-------------------------------------------IAINLSQYFLQPDFR 230
AgamGr24     LC-------------------------------------------SLSVAITHVTMVDFK 155
AaegGr63a    LS-------------------------------------------CLAVIITHVTMVEFQ 220
DmelGr63a    LS-------------------------------------------VLSVVITHVTMSDLN 249
TcasGr3      LA-------------------------------------------CGVMVVTHITMAHFK 211
DmelOr83b    LFSMIHSN----LCDVMFCSWLIFACEQLQHLKGIMKPLMELSASLDTYRPNSAALFRSL 262
AgamOr7      LFSMVQSN----LADVMFCSWLLLACEQLQHLKGIMRSLMELSASLDTYRPNSSQLFRAI 260
HvirOr2      LFSMAIAN----LMDVMFCSWLIFACEQLQHLKAIMKPLMELSASLDTYRPNTAELFRAS 263
TcasOr16     FFSLAHAN----LLDSLFCSWLIFACEQLQHLKEIMKPLMELSATLDTYVPKSADLFRAP 362
TcasOr22     FFSLAHAN----LLDSLFCSWLIFACEQLQHLKEIMKPLMELSATLDTYVPKSADLFRAP 401
AmelOr2      IFCMADAN----LLDVLFCSWLLFACEQIQHLKNIMKPLMEFSATLDTVVPNSGELFKAG 260
TcasGr31     -----------------------------------------------VIDFSLCKAILVH 92
TcasGr35     ----------------------------------------------IVTILKKTKSNIFI 104
DmelGr59c    LSDSLHTIS--------------------------------------DVSAQRKRITADL 161
DmelGr92a    AHEQTYLW--------------------------------------FTIRKGFSWRFLID 173
DmelGr28a    VIYLCVSYS------------------------------------LLVSATISPSFVTFT 178
DmelGr64b    SALVEHCLLLG--------------NSFHLSNMERTQCKINVTYFESIYKWERPHLYMIL 186
TcasGr71     GYVWT-----------------------------------------DILGFGYFREYLAE 148
AgamGr19     EHMLSIG-----------------------------KTVNARVYEARTCHWNYSNLPEYY 171
AaegGr64b    -----------------------------------------------YAHRNYAYIFNWM 128
DmelGr93d    LVIASRLCG-------------------------------------FQHIIWWVSYTYVF 178
TcasGr34     MSNYAFTR--------------------------------------IILLKSVLEIVIID 133
TcasGr43     VVIIYS----------------------------------------ICVQTEKSVFDFLC 148
TcasGr47     LTLCY-----------------------------------------AVYYWRQQYEDFWH 131
AmelGr3      IIDFYTWN-----------------------------------RKTKVDNKAMTGKGPIN 188
AgamGr32b    IASLTVPIV------------------------------------MTTILWDIATIPYVM 183
AgamGr50     GMMVLS----------------------------------------AAFFRTFIELKQSL 165
TcasGr20     FFDVVVWG--------------------------------------LSASDLGVFFKRFL 174
TcasGr79     ATKKLEQF--------------------------------------IVRFMKYHFFGTLK 153
TcasGr15     WCWIVEIGG-------------------------------------WLAFTKQLLIKFVE 156
TcasGr60     KLLGQYP----------------------------------------VGIVGVKNASLFI 147
AgamGr38     GTAIG-----------------------------------------MCVLVIAVMYSENL 171
TcasGr51     QLVLHTVKTVLKTTMSTFSDITFMRTVWKFFNIFLITPFYNFNENTIHSKLCKLYATFLI 444
DmelGr39b    LVTVLP---------------------------------------SIYVALSGSLLYFWS 151
AgamGr28     FFTLTSGVG-------------------------------------LAVAHLFFGGIWST 168
TcasGr61     HDQLCDMCGAINMLFGFPIIIGCLIQFNTIVFSFCYCYYNSKMTKDGVYNTLFFIVYVSL 298
TcasGr50     VLVFVPDY--------------------------------------VFFVENEPILYLIF 159
AgamGr12     MLYLS-----------------------------------------LLILSQYNSSTKFP 162
AgamGr36     ATGTVFALG-------------------------------------MSTISEFSIRNHLI 173
AgamGr48     VGLCTLFAMN------------------------------------SGIKRHETSAHVYR 169
AgamGr49a    TRALLRHIDP------------------------------------PAGESPVGRRLFFK 186
AgamGr49b    CALFVLYFG------------------------------------QFFIQDGDIQSQILW 173
DmelGr2a     WGYAVSQLLILG--------------------------------AKLLSRGDRFPIYWIS 182
DmelGr32a    YIIVLKIY-------------------------------------AVQGEITPTSYILLA 219
DmelGr68a    VAFYYIHYR-------------------------------------SGIGAKRQIILLLI 170
AmelGr7      CHYESTTP--------------------------------------ALIKLCLSILPNIP 173
AmelGr8      LYMIVNYD--------------------------------------FCVFLFICWYSFFG 181
AmelGr9      LPNCSKSD--------------------------------------IHVTLRNVTHLYIL 173
TcasGr57     LWISSVGVR------------------------------------MFQYYIGRSFTYYVC 157
TcasGr41     LAFLYS----------------------------------------FDLFANGITFGWHT 159
AgamGr34     DCYVTVFR--------------------------------------GFIRVDYWIITILP 170
AgamGr35     GFVSFVQLT------------------------------------TVEVWYWLSHQLPFI 201
AgamGr39     LSCLTGTAFTN------------------------------PFLVDYDTWCNFTALVGFI 179
AgamGr40     FFLMISTF--------------------------------------FIVQDQSSQQFTAT 166
AaegGr28d    LFVLIITGTN--------------------------------RTNEDWLDLSEMFPDWIS 175
AgamGr42     LGSFACQG--------------------------------------LLILNDYTAGKMLV 185
AgamGr41     YNYPAN----------------------------------------YQFFHMFSTLFATA 180
AgamGr43     IILSVCEAYADHG-------------------------------YYFTIITIVYPLWMFH 182
```

```
DmelGr33a      HVSVISQR-----------------------------------FEQINMLLEKINQEARH 210
AgamGr44       SSFVLAVL--------------------------------------AVLDAGEETAIGSE 170
AgamGr13       VYITLIEWAEIS--------------------------------IVQAVLFMVSLIPNIA 172
AgamGr45       LMLILGVG--------------------------------------VKAVLEVTNCLMYI 204
AgamGr46       CRFICLHMT------------------------------------FVFLCIGFAEWYNCI 188
Dmelr93a       GCFHEIIP--------------------------------------LFENSHFDDISQMV 181
AmelGr6        TAVNQS----------------------------------------GMFAFEETWTFNVS 219
AmelGr10       YWAIVG----------------------------------------YFTYRIEAKVPIFH 155
TcasGr28       ----------------------------------------------FYYPVATMSFYVLN 125
TcasGr40       VALLEHCFS-------------------------------------MLNYVYSSKCENNS 176
AgamGr3        AVCALVEF---------------------------------------TIYNQLTPGTQWH 174
AgamGr4        AQVLYLAWD------------------------------------DPSALAYWLVIMILH 201
AgamGr6        FNVIPAVS--------------------------------------EFFIISRISGNPVW 195
AgamGr7        HLHHAYHIDR------------------------------------LTARFDILREQLES 192
AgamGr47       DEIFTVYG--------------------------------------RGAGGPRGRKIGDA 185
DmelGr8a       HLGLWQFQ--------------------------------------ALTQHMLLFWSTYE 167
AgamGr5        YFVVNESY--------------------------------------LYLCAEQMQTKRFY 211
AgamGr8        LTYAYWIT-------------------------------------KSARSSNFTILFWVL 157
DmelGr9a       CN--------------------------------------------AFLVLSEYTIRGIY 138
DmelGr59f      IFFN-------------------------------------------IWTHKFVVYRSIL 184
DmelGr77a      LLSSYLLSMAQP----------------------------------IQILKDDPEVRRNF 200
DmelGr85a      LRLSIHLAT-------------------------------------FTLSARMGFNHPCN 185
TcasGr27       AMFIVP------------------------------------------YFQDRREMPLIV 152
TcasGr48       FIYLFT------------------------------------------VYYIYYSVHEAS 167
AgamGr1        SAAYGFLLT-------------------------------------FMVLTSKVLFVSQT 174
DmelGr10a      LMMMIQVCG-------------------------------------IFAQYGEVGKGSVS 177
DmelGr59e      VVNYDFEFWT------------------------------------TWSSNSVYNLPGLM 163
DmelGr10b      YNFFHN----------------------------------------FNFALSAIMHWGLR 159
DmelGr89a      YDLLCSVP---------------------------------------FLKGAGGRLPWSQ 158
DmelGr57a      YLMAINS---------------------------------------AAVQVASGHRALFL 175
DmelGr47b      CFFSFSIS--------------------------------------LIFDTTRCTCGIPS 174
DmelGr58a      ANLSILLG--------------------------------------ALILFRIHSVQRIS 161
DmelGr58b      IILLYSAF--------------------------------------LCSTVLQYQLLSVI 171
DmelGr98a      IVVTIYSN------------------------------------RALTINATYSELVFLA 167
AaegGr93a      LWLNMNPT--------------------------------------YRLYSNGDLFLSVV 174
TcasGr19       YCAFIIFT--------------------------------------VHLLFLLCIYHFFC 171
DmelGr47a      IHLIQP----------------------------------------IYMRRLLPSQFYLN 153
DmelGr58c      QQLLTRDS-------------------------------------VVNCESNSSLRQAMV 166


TcasGr38       IVSE----Q----------EWSFSTGLLVMIFNSALSYKASEIVVMLRSRFAILN----- 214
TcasGr46       IVSE----Q----------EWSFSTGLLVMIFNSALSYKASEIVVMLRSRFAILN----- 196
TcasGr1        RNQEYKYYQ----------ILSHLSGIFFTVFVNAQCYLTTELVLMLQTRFVILN----- 199
TcasGr123      NLNG---DT----------MIVEAMAYILLAMNSAVCHQTIELVKMLKIRFVILN----- 197
TcasGr62       NLNG---DT----------MIVEAMAYILLAMNSAVCHQTIELVKMLKIRFVILN----- 472
TcasGr25       IFSVPLLMK----------IASQFVGIFCSLYFCIFGYIYDDEMVVPQN----------- 100
TcasGr59       IFSVPLLMK----------IASQFVGIFCSLYFCIFGYIYDDEMVVPQN----------- 275
AgamGr2        QLTVPTPPK----------KTNFNATTKVITVKPYQPKQPASPPLKPSVTPPPPHRNGAI 211
AaegGr66a      NAVQPIMPM----------KPHGNQQGKIISVKPFEKRLTEEKVLAQKR----------- 288
DmelGr66a      MDIGSIGKS----------SVSGSGKNKVAPVAHSMNSFGEAIDAASRKPPPP------- 302
TcasGr11       Q------------------IMLFVS----ALIKAFSKYQFVVLVLQLQHRFGKIN----- 207
TcasGr55       Q------------------IMLFVS----ALIKAFSKYQFVVLVLQLQHRFGKIN----- 207
TcasGr21       TK---TYSL----------FIVFMTVILPYFINLLMELQYCHYLNILRVRYQLLN----- 213
TcasGr56       TK---TYSL----------FIVFMTVILPYFINLLMELQYCHYLNILRVRYQLLN----- 213
AaegGr         I----CGTT----------WAAFF---LPFMMMTMITSLFVCIVNQSKHRFYLLN----- 213
AaegGr28a      F----SITA----------WMSFM---LPFMMISMITVLFVSLLNQSRCRFQQLN----- 109
AgamGr33       P----SVTT----------WMAFL---LPYFMMSMVIVLFLCFVNQTKHRFHLLN----- 211
DmelGr28bB     P----SFCV----------CMTFF---LQHSVVSIAICLFCVIAFSFERRLSIIN----- 213
DmelGr28bC     P----TMAL----------HFTFL---IQHTVIAIAIALFSCFTYLVEMRLVMVN----- 240
DmelGr28bD     P----IYTA----------IVAFY---VPQIFLLSIVMLFNATLHRLWQHFTVLN----- 210
DmelGr28bA     A----SWQL----------TFIFV---YELLAISITICMFCLMTRTVQRRITCLH----- 222
DmelGr28bE     TF---SISA----------VISFF---SPHFIVCAVSFLAGNVMIKLRIYLSALN----- 217
AgamGr25       KENSNSSAN----------VLGYFPFYCLYYILMMFHVLFAQAALGIGNRFRHLN----- 212
AaegGr43a      KENQDSGAN----------VLGYCPFYALYYILMMFHVLFAQSALGINRRFHRLN----- 211
DmelGr43a      IAQSDTELN----------VHWYIPFYSLYFILTGLQVNIANTAYGLGRRFGRLN----- 207
AgamGr37b      SRLESFDTP----------ALDVAALAISSFCFLLQIVQFTVMVLLVLSRYQTIN----- 206
AgamGr37d      ELVPHIEPS----------LKEILPSAMFGLCFLLQICQFLLFLLVLHDRYCAIN----- 202
AgamGr37a      LHESSCNMQ----------ILFVFIYLLFVNSYALMMLQCTAFMEIIRTRYKLIN----- 216
AgamGr37c      WLNWNQLIG----------HVFQFVRLLYFTTYVHLWLQATLAAMLVLSRYEALK----- 210
AgamGr37e      WLPLFGVQV----------HAFNLVRFLYFYSYVHLWAQAILTLFLVLSRYIALN----- 210
```

```
AgamGr37f       NQPNFDGRW----------YYNMFSFVYYNAAFPTITSYFAIVLWFLLVRFQRLA----- 210
AaegGr28c       YRGLVDGLG----------VFYVFSRFLYSCGYVQVWLQSTVVLLLVWSRYVALN----- 209
AaegGr28b       EMSSGFGMP----------ISMALIFGCFGVPFSLFTHNLSMIGYLVSYRLNHLN----- 216
AaegGr28e       EMSSGFGMP----------ISMALIFGCFGVPFSLFTHNMSMIGYLVSYRLNHLN----- 216
TcasGr14        SDVSYNLFS----------FVPLVVNALDHLFLNDILSDICDKFEQINQHFRRQIKS--- 183
TcasGr49        SDVSYNLFS----------FVPLVVNALDHLFLNDILSDICDKFEQINQHFRRQIKS--- 227
AmelGr4         TEIIYFKPE----------PITLLGNLIPTIFAGLLFVQYFFVITLINEMFIKLNCI--- 211
AmelGr5         TEIKNFNPN----------PLVLMASIIPLIFVGLLFIQYFFVLNLIYAIFVKLNCI--- 211
TcasGr16        EELDLVWRE----------VCTFVVQVYPRLVVNTINLTFLTLLMILEGRFRVINDG--- 206
TcasGr22        -----CQDQ----------ILIFLVNWYPRLIIGIMNSTLNLIFLLIQTRFEMINN---- 121
TcasGr17        -----KSKQ----------VEYFLIHWYPRMLISTKLLAHFLILTVLKARFEAINN---- 196
TcasGr150       -----KSKQ----------VEYFLIHWYPRMLISTKLLAHFLILTVLKARFEAINN---- 196
TcasGr32        FANIMGLEF----------YKQFAIEYFQMYVLFFVNFAFFVVVKMLLVRYRNLT----- 180
TcasGr54        FANIMGLEF----------YKQFAIEYFQMYVLFFVNFAFFVVVKMLLVRYRNLT----- 180
TcasGr37        FTLLLGVDF----------VKEYAFEYIQLYSQFILYFLLFATLKILLGKYTQLK----- 165
TcasGr53        FTLLLGVDF----------VKEYAFEYIQLYSQFILYFLLFATLKILLGKYTQLK----- 165
TcasGr5         WTRIMGVAY----------YEQYAVEFFQFYAQFIVYFLIYAVLEMLLQKYKTVT----- 176
TcasGr12        CQTVSNFEY----------FQRKILLMIQLYYLYFDVIVISTFLQLFLEHYEVIN----- 170
TcasGr13        CQTVSNFEY----------FQRKILLMIQLYYLYFDVIVISTFLQLFLEHYEVIN----- 244
TcasGr104       WICEMGVVT----------LQAYTLHLFCVLYVVLLHFLTYNLSLGIKLRYDDVN----- 201
TcasGr45        WICEMGVVT----------LQAYTLHLFCVLYVVLLHFLTYNLSLGIKLRYDDVN----- 201
TcasGr98        RIAAKGLPT----------LKLFALHEFYGFCYLWVLILICNIALAFKQRYQLIN----- 199
TcasGr52        FIVPSGVVY----------KIGYWLHQICHICELHTIFLVCNVLTVFKIRYKDLN----- 140
TcasGr105       WSPID---H----------TESNIINDMCSLYYILQITLIVNYALALKNRFKELQ----- 194
TcasGr4         YKCGIQYHI----------CNTVISATVCFMMLLMLEIWRRFTILNNYLTIVLGETWTSL 197
TcasGr44        YKCGIQYHI----------CNTVISATVCFMMLLMLEIWRRFTILNNYLTIVLGETWTSL 201
AgamGr9a        FLSVYIVDC----------TFMYFDVVIELVLGLCDCLLLIAHLQLERLVWIVKNRD-QA 211
AgamGr9c        FL-VYMVSC----------TFMYFDLVIELVLGLCDCLLLIARLQLERLVWIVKNRN-DT 208
AgamGr9b        FLPLYITSC----------AFMYVDLAMELVLGLCDCLLLIVRLQLQRLVCSARNLE-RS 260
AgamGr9d        FVSMYIVSC----------FFMYIDLGMELLLGLCGCLLLVAQLQLGRLAQAAREFD-YG 215
AgamGr9e        AWYLYLTIG----------IYLYEDLCLSWLFGFYSTIMMHCVDQLSYAAHLLQED--HA 208
AgamGr9f        PWYMFLNIG----------IYIYEDLCLSWLFGIYSAIMLFCVAQLSCAAKLLQSDYYHS 205
AgamGr9g        FVHIQLSVC----------IYLATDLALDWTLGLCCVMILVGIAQLSHIFDLLKIASIRR 212
AgamGr9i        FVHLQLSVC----------IYLAMDLALGWTLGLCCVVMLVGIEQLSHIIDLLLKASDER 212
AgamGr9h        FILMYIETL----------LLLYANNCIEFTIIICCTTQMTLCRYLKDLMNALETKK--- 213
AgamGr9j        MVLMDMIIL----------LRFCFMFLILELYRVCVRIIRKRMKQLQVLLTQMEEIN--T 246
AgamGr9l        IFKLYWLIL----------LRFCGMFVMVELYRACVIVIEERMKQLHSLL-QLTING--P 236
AgamGr9k        HKMLTTTFV----------LRYTGMIHFLYLFHMCVSMVGLRMEQLKMLFHHKQT----- 226
AgamGr9m        HTFLELTLI----------SRFLLTTQYIYLYYLCVSMVRLRMQQLRVLFLDHQH----- 229
AgamGr9n        IKILAVMIA----------GRFLAIWVLIFVHRLHVRAIGQRMEQLRVLYAS-------- 198
AgamGr10        LKILRMMTA----------GRFFTIWMLIFVHRLHVRAIGQRMEQLRVMYAS-------- 198
AgamGr11        LRILSMSVA----------LKIFGIGYLCIIHRVCVGAIGVRMRQLSILGKLSCG----R 199
DmelGr23aA      LMWLLRYRY----------VQLVQHVMDLNQRSIHLRRSLLSMASGNDLWQP-------Y 199
DmelGr23aB      SFLALQVQI----------ISFILEVMKVNIRVRQTKLQLLILARELSCRWPQRKQKPQF 202
DmelGr39aA      TMQISRVLT----------LIGFVVYRCVLANFQFTCYTGMVVILKKLLQVQVKQLEHLVS 203
DmelGr39aB      DASRLFVIT----------SLGFPLRYLVTSFTMGTYFCMVHIVRLVLDWNQSQINAIID 204
DmelGr39aC      LTTIPIYLL----------QYGFSYLFLGQVVVLFACIQQILLSILKYYNQVVLK-NIKS 206
DmelGr39aD      TLPLALCML----------VYIFAFLVLCLLLMFFVSLKQVMTAGLIHYNQQLCQGDLIS 206
DmelGr98c       PFFHWVNQV----------LTQIILIMLQLKGPEYCLFVLLVYELILRTRHVLEQLKDDL 221
DmelGr98d       PYLHWTNKV----------LTEIILIMLQLKCTEYCVFVLLIYELILRGRHILQQISVEL 219
DmelGr98b       PFVSTLLKI----------LTEFVMIMQQLKSLEYCVFVLIIYELVLRLRRTLSQLQEEF 219
AgamGr51        LASWLTFYA----------MMYFIAVIENFILAGVLIGAVMQAMVNIGLKRLARSR---- 237
AgamGr52        VVVGVTFSV----------FRYVFTALVNLYLVGLMIVSFNQGSINSKLTSLVDR----- 231
DmelGr94a       LIWTLYRLF----------PLIISNFLNNCYFGAMVVVKEILYALNRRLEAQLQEVNLLQ 214
DmelGr97a       SVTTTKTML----------PLIVSNQINNCFFGGLVLANLIFAAVNRKLHGIVKEANMLQ 235
DmelGr93b       LLTLVCDLY----------TSVGTGMITHLCFVGYLSIGVLYRDLNNYVDCQLRAQ---L 225
DmelGr93c       LFATLCEIF----------LEIGSLMIIHIGFVGYLSVAALYSEVNSFARIELRRQ---L 219
DmelGr22d       VLNVLVVSM----------VKLGTLLLALHYHLGVVIIYRFVWLINRELLSLVCSLRGNH 220
DmelGr22e       FIGLGSLCL----------MLLAVLLGASHFHLAVVFVYRYVWIVNRELLKLVNKMAIGE 223
DmelGr22a       VYEAVCIYI----------VQLEVLMVVMHFHLAVIYIYRYLWIINGQLLDMASRLRRGD 222
DmelGr22b       ICLVLLSCL----------MEFSLNLNIMHYHVGVLLIYRYVWLINEQLKDLVSQLKLNP 221
DmelGr22c       VEMVLINSL----------VQFSFNCNIMHYYIGVLLIYRYLWLINGQLLEMVTNLKLDC 218
DmelGr22f       KILKILCCL----------PSVGLQLIIMHFHTEIILVYRYVWLVNETLED-------SH 214
DmelGr36a       FVGMASDFW----------MSAIINMAISQHYLVILFVRAYYHLLKTEVRQAIHESQMLS 208
DmelGr36c       YLGLGLQYW----------MFVILNMAMMQQHMIMLFVRTQFQLINTELRQVIDEAKDLL 208
DmelGr36b       MMGLLVKLC----------VSFIMNLAISQHFLVILLIRAQYRIMNAKLRMVIEESRRLS 208
DmelGr59d       ALRIWAVLS----------LTAIINVIITQYYVATACVRGRYALLNKDLQAIVTESQ--S 207
DmelGr59a       LCNGLLVNI----------SLTILFVNTFFYFTSLWHIARGYDFVNQQLNEIVACQSMDL 207
DmelGr59b       VLRFFFKCN----------TNNILEMVPMGYFLALWHIARGFDCVNRRLDQIVKSKST-- 209
AgamGr26        ARSSAVYTL----------SNVINVLALLQYAYVLYVLYFCYYDTNRLLASYNRHLLVRG 227
```

```
AgamGr27        LCHSVLYIL----------PNVINVLALYQYAALQLIVAACYRSVNSVLARYHDHQARP- 227
AgamGr29        LAKIVIFLA----------TQLMATSLTLLYLTIFGTVSVLLRQMNDTLEGILVGPIS-D 229
AgamGr31        LLKVVIYLA----------TQLMATSFTLQYLTVFGIVAVLLRQMNDTLELILYGTVVYD 230
AgamGr30        TLINLCYIP----------PFIAIVINVLHYYALFGTISGIMSCSNDTLYGITVEGKKDR 229
AgamGr32a       TLLNVSYLA----------PYVTIAVYILLYRALLASIAGIVGCLNDNLREITIQDRIDP 229
TcasGr2         EYDIFKALV----------LQTINLQITFIWTYNDLFVMLIS--TALAYRFGQIT----- 247
TcasGr9         EYDIFKALV----------LQTINLQITFIWTYNDLFVMLIS--TALAYRFGQIT----- 247
TcasGr7         DYNIVMALV----------LQFITLQHTFIWVFNDVFVMLLS--TALAYRFTQVT----- 249
TcasGr30        DYNIVMALV----------LQFITLQHTFIWVFNDVFVMLLS--TALAYRFTQVT----- 249
TcasGr29        DYNIVMALV----------LQFITLQHTFIWVFNDVFVMLLS--TALAYRFTQVT----- 249
AaegGr64f       PYHPALGVA----------IEVVMRVAKFTWHYIDVFIICVS--LVLQRRFQQYN----- 207
AaegGr64a3      PYHPALGVA----------IEVVMRVAKFTWHYIDVFIICVS--LVLQRRFQQYN----- 207
AgamGr14        PYHPVVGWT----------IELTMRIAKFTWHYVDVFIICLS--LGLQRRFVQFN----- 271
AgamGr18        PFNGFTIAI----------TEWINRCMRYTWTYLDIFIISFC--YGAQFRYEQIF----- 243
AaegGr64d       PVNIGSILF----------VEWINRCLRYTWTYLDLFIISFS--YAAQFRYTQIY----- 275
AgamGr17        PYSLPAVLL----------LELTHKIFLYVWTFMDLFIIFVA--LGLARRYEQFY----- 232
AaegGr64c       SYSLPLALL----------LEFVHKVYLFVWTFMDVFISVVS--IGLATRFEQLF----- 257
DmelGr64c       HFTAWWIPF----------IEWTTLSMTFVWNFVDIFLILIC--RGMQMRFQQMH----- 236
DmelGr64d       EYRAWMVFF----------IEWNTMAMTFVWNFGDIFLFLMC--RGLKIRFQQLH----- 245
AgamGr20        PYHHLLTMY----------LLYTTISLTFIWTFTDLFIMLVA--TGIACRFGQLN----- 264
AaegGr61a       PYNLPVALY----------NEYVVVAMTFAWNFVDLFIVLVS--IGLTTRFTQLN----- 258
AgamGr21        PYNSLSAVF----------FTYVSSALTMYWNYQDIFIIMIS--IGLATRFQQIN----- 286
AaegGr64a       PYSTVSAVF----------FSYVSFALTLYWNYLDVFIILIS--IAIATRFDQIN----- 168
DmelGr64a       PYSPIIAVL----------ILLINGACTFVWNYMDLFIMMIS--KGLSYRFEQIT----- 282
DmelGr61a       PFNIFSMCY----------GFWLNGAFTFLWNFMDIFIVMTS--IGLAQRFQQFA----- 265
AgamGr15        PYSTWRGFL----------TKFFNVICNFMWSYVDLFVIVIS--MGLSHAFRKYL----- 268
AaegGr1         EYSEIRGFV----------VKFINVISTFVWSYIDLFVIIVS--IGLSHTFRRIN----- 273
DmelGr5a        PYSNWLAWL----------GKIQNVLLTFGWSYMDIFLMMLG--MGLSEMLARLN----- 269
DmelGr64f       SYSTTLALW----------GKFQNVFSTFIWNYMDLFVMIVS--IGLASKFRQLN----- 294
DmelGr64e       DYTRFLGIV----------GKVVNVLSTFAWNFNDIFVMAVS--VALAARFRQLN----- 288
AmelGr1         SYSLWKGIL----------VDIINILSTFSWNFVDLFLILIS--IALTDQFRQLN----- 217
AmelGr2         NYNFVYGLY----------VFVVSKLATFTWNFTDLFIMLVA--TGLAERYKSLN----- 305
AgamGr16        PYNPYIGFL----------AQTITSLLTVYWNYVDLFLISVS--VGLRTNLAQVN----- 242
AaegGr64e       PYDIYVGFV----------AQVVTSVLTFYWNYVDLFLIVLS--IGLRQSVRHVN----- 260
TCasGr6         LYHTTAYLH----------IITMINMNCALWYINCRAVGNAS--TALAESFQNVLVPT-- 201
TcasGr26        LYHTTAYLH----------IITMINMNCALWYINCRAVGNAS--TALAESFQNVLVPT-- 201
TcasGr33        LYHTTAYLH----------IITMINMNCALWYINCRAVGNAS--TALAESFQNVLVPT-- 201
AgamGr23        LWHTSAYYH----------IITMLNMNSALWYINSRGIRVAS--SSLSRCFR-------- 296
AaegGr21a       LWHTSAYYH----------IITMLNMNSALWYINCRGIRVAS--SSLSDRFR-------- 288
TcasGr10        LWHTFGYYH----------ILAMLNCLCSLWFINCTAKGRVA--VWMCNNLH-------- 244
TcasGr39        LWHTFGYYH----------ILAMLNCLCSLWFINCTAKGRVA--VWMCNNLH-------- 244
AgamGr22        FCHTFAYYH----------IIAMLNGFCSLWFVNCTAFGTAS--KAFAKELT-------- 269
AaegGr21b       LTHTFAYYH----------ILAMLNGFCSLWFVNCTAFGTAS--KAFAQELS-------- 269
DmelGr21a       LWYTFAYYP----------IIAMLNCFCSLWYINCNAFGTAS--RALSDALQ-------- 270
AgamGr24        LLQVIPYCV----------LDTITYMMGGYWYMACETLSITA--KILAEDFQ-------- 195
AaegGr63a       LVQVIPYCI----------LDTLTYMMGGYWYMTCETLSITA--NILAEDFQ-------- 260
DmelGr63a       INQVVPYCI----------LDNLTAMLGAWWFLICEAMSITA--HLLAERFQ-------- 289
TcasGr3         IIQVVPYCY----------INCLIYLIGGFWFMQCDVVGKVA--SQLAEDFQ-------- 251
DmelOr83b       SANSKSELI----HNEEKDP-GTDMDMSGIYSSKADWGAQFR-APSTLQSFGGNGGGG-- 314
AgamOr7         SAGSKSELI----INEEKDPDVKDFDLSGIYSSKADWGAQFR-APSTLQTFDENGRNG-- 313
HvirOr2         STE------------KEKIPDTVDMDIRGIYSTQQDFGMTLRGAGGRLQNFGQQNPN--- 308
TcasOr16        SATSQDQ-------LIENDYNEKNEDLKGVYSTRQELGGHFR--GGALQNFGSGGVG--- 410
TcasOr22        SATSQDQ-------LIENDYNEKNEDLKGVYSTRQELGGHFR--GGALQNFGSGGVG--- 449
AmelOr2         SAEQPKEQEPLPPVTPPQGENMLDMDLRGIYSNRTDFTTTFR--PTAGMTFN-GGVG--- 314
TcasGr31        AITIRSTFS----------WTFIDVFIMLTSTAFVFRLKQLNAKVEMLKNARVKN----- 137
TcasGr35        LKESVDTFN----------DIFGWIILCNIFEAAAKSLIYIDMIIKKNVTQQNSD----- 149
DmelGr59c       IVKLSLLAT----------LTTIFNMIVCQYYLAMVQVIGLYKILLQDLRCLVRQAECIC 211
DmelGr92a       WWCDFYLVS---------ATNIFIHINSIGYLSLGVLYSELNKYVYTNLRIQLQKLN--- 221
DmelGr28a       TFALPHINIS---------LMVFKFLCTTDLARSRFSMLNEILQDILDAHIEQLSALELS 229
DmelGr64b       PYHFWMLPI----------LEWVNQTIAYPRSFTDCFIMCIGIGLAARFHQLYRR----- 231
TcasGr71        AVQLYFQFY----------YTYFLCVIVCIFRGKYRNINLLLTEQLQNRRFFAKK----- 193
AgamGr19        ALAHLRVLFP--KSIIQLSLLYIFRALTMAWTVQDVLLIMISDSIAGYFKRINSR----- 224
AaegGr64b       PYNVPVLFF----------VRYVTYSVTMAWTYQDILIMVISAYVLTRYRQFFWR----- 173
DmelGr93d       IICNSIMCFG---------FIWHLSLGVLYAELNDNLRFESGFQTAFLRKQQRIR----- 224
TcasGr34        IEIYSQFLYG---------FMIYLILDTIKSKYRQMHRLLANYKVITSDEFFYLVS---- 180
TcasGr43        RHTVEYFQN-----------YIHFFYQLLLYLITDMILMRYKELNRVILDNMSRN----- 192
TcasGr47        RYTLSFIEN--------------YCQFLYNCFHSTLLLMILSRYQRLKAQLWQNR----- 172
AmelGr3         YAPLYFMYT--------VIISTEIQYTVSTYNIGQRFIRLNTSLKDLFNANSNNNDNAID 240
AgamGr32b       AQTVPFVVS-----------SLILNQYFCVFVHLTSILRKMNERLARFLNMLPGSATEPP 232
AgamGr50        IYSILGVCN----------LLQISSMNVAVNLLMFVLYSGINIYARINAHCNDLVYG--- 212
```

```
TcasGr20        PVYVSYLLIFVQQIPFCFFVRVIRLKIEDLNGAFRAGLERVQNGENQWISVMKVNKQ--- 231
TcasGr79        LWSQLFSTFAG---------IEVLHVVKQCYHLQKINVQQSGIFINLPFLEHNLIR---- 200
TcasGr15        VYPLFVLLIVS---------NVVLKILVEKYRHQKNFINESNVETVKFNLFLLSQAVETF 207
TcasGr60        FDLIFLSFR-----------CTKLCKEANKTAFLWYHIKIDREDEEARDDFIKLG----- 191
AgamGr38        PWMLLLNLG----------YSYFSLTTFHILSLTCLDVVRARNVRLNKVFRKQFSLEG-- 219
TcasGr51        ALKLVWIVALFKNENLSEMVKQFLFTQKFTFVAGLATLSVLNLLSVIKSSFLDINTWKML 504
DmelGr39b       SLLSILIIR----------MQFVLVLLNVELLGHHVSLLGIRLQNVLECHLMGAN----- 196
AgamGr28        LFTMGAYVLP----------NMLASMSLAQYYAGTVLIYKLQHTVNQQLRTAIGKHR--- 215
TcasGr61        LLGPHPTYFGKTGRKTVLKTNKYCNFIILFYLSVFVILVYYASLDETPNPVTNGKLYNFN 358
TcasGr50        SYCPIVTTG---------IIKIQFATCAHLILQRVNHVKKILEKETQTVAFPKDN----- 205
AgamGr12        LYVTILEAVR----------VYVTMIAILIYIVCVLVVKMHFRQLQDRVEQFGAS----- 207
AgamGr36        NSFSYALTG----------IQFMIVNFHFVAAARLVSFRLDAIKCSLKKHLDTGSWYIE- 222
AgamGr48        TVNIYVVMMS---------SQFTNLLLLMLLFGSYVYGQINKQLDRTVHRLASFETQQS- 219
AgamGr49a       TVAIDLLQSFTWYGTFLWLQRKFGATYILAFWLNSIAVMQIVAALNILLATLLWG----- 241
AgamGr49b       TLNIFSVWQS----------AIFTNLIVVTLIIGVHFLEILNHRLTLYIRQMRAPACHLK 223
DmelGr2a        YLLPLLVCG----------LRYFQIFNATQLVRQRLDVLLVALQQLQLHQKGPAVDT--- 229
DmelGr32a       FYGIQNGLTAT--------YIVFASALLRIVYIRFHFINQLLNGYTYGQQHRRKEGGARA 271
DmelGr68a       YFLQLLYST---------LLALYLRTLMMNLAQRIGFLNQKLDTFNLQDCGHMEN----- 216
AmelGr7         FIVFGVSTIS---------FCFWVTCLKLKFRQLNELLRSMRIMESPIHKRVLEMTNN-- 222
AmelGr8         LLIISTLYLN---------NVYILNACFKFINNSLIKIKEIVINDEPHLLRREYHMK--- 229
AmelGr9         FINFSVDMFYVN-------CIWVLKVCFKKLNKSIHELKKFRSGNDLRVETIVQQKN--- 223
TcasGr57        NTTIFLIFHS---------VLPIKRFFTSLGIAFDNIMKNLICEIDGRHEFFLAEFKS-- 206
TcasGr41        YIFDVPNFVN---------EYVQAIEVLYICSYTVLIRNKISNLNTSLIHSTHLNG---- 206
AgamGr34        QFVNVIAVT---------QVVLLLLYINARFRKLNQLLEEEQHPVAGRKRFERASRLAGN 221
AgamGr35        IYAMAFLHAYVLIYWLHARFRRLNTLVEQYYRQGHIFAPARQTIISFATMVKLDEES--- 258
AgamGr39        MCNLVYIMTSCFCCVTLWAIVYRYHNINATFWYVSLHQPVCLTGGRNHVLLNSFASMYFH 239
AgamGr40        EMVVFLRSS----------LVFTIFGSYITVALTTIYMRFRALNKMIREKFPTTRTADP- 215
AaegGr28d       ILAMCRSATS---------LGIFTCYITMTLYSLKTGFDQLHQAIAKHLNTTPQAIN--- 223
AgamGr42        NTTLSLQSMP--------MQAITSYYYIIRSIHISCVVAFISGLYSFRDRFFAFNKQLR- 236
AgamGr41        AISIPLLSLASIRGLEFWMDHFFVLTFVGFSWSGGLLYNNICNVLLFHILYRIEVIN--- 237
AgamGr43        ITILEFTTLVKSTECILTKLNWMLDTFLESLQRQSTPSVTLKGEKNPTNKVYPLNRR--- 239
DmelGr33a       RHAPLTVFDIESEGKKERKTVTPITVMDGRTTTGFGNENKFAGEMKRQEGQQKNDDDDLD 270
AgamGr44        LLSLCIFYYP----------KLTIICSVSLYYAVMLFQQNLHYALNERLKQLIVEHTG-- 218
AgamGr13        HVNNFYALLFVQRLTLGEINDMLADLWTICIRPGANELLPPSRRLQLHLQQYQQCAG--- 229
AgamGr45        RDSGSPFSS-----------NCLLLCIIPQTLCVISELQFVAFALLISERLKLLNR---- 249
AgamGr46        MYMSDFIPAS---------EYCIFECFITMLTSSTVELQYVAFVQLIKNRLQLINDLLVE 239
Dmelr93a        GTGFGIYMWLG-------TLCVLDACFLGFLVSGILYEHMANNIIAMLKRMEPIESQD-- 232
AmelGr6         YMCTYIGTAT--------AVYKFFGMASFLGLRFHQLNQIAKENLPPRVGYKSSNVS--- 268
AmelGr10        GIIYFIMDAS---------MNTQILIFVCILFLIEERFRHLCSMIELSKADKIIEAHR-- 204
TcasGr28        FFGSIQFII----------ISKHWVTIMKEWSFMEMSMRNYGSSINMKKRFVVMT----- 170
TcasGr40        TGVEHFFKK----------QFHYIFTYMPYNIVFGLCLTTFGLLPVNGVTSEKCN----- 221
AgamGr3         WFWLHNFYP----------YTFSHVRHVFHLLHISLMASNLRQLQRKLVALHQTGER--- 221
AgamGr4         AFLRLRHLFH---------MFFIDILKIHLQKLHHDLVDAGEYMADLVEQPQDTAVFR-- 250
AgamGr6         HRNWLLKVW----------FFILIRLGDSFFLVHVLYLRNRYRVLNNELQQTASTVG--- 242
AgamGr7         LVTDGVSPGRP------KPTEATAVPPKAQTSLTRWKMNGPSRPAVIVDSWTGLSTMKS- 245
AgamGr47        NAGGMMKSKG---------PPKVTMVAPASAGFNKLSATTVYTTYITQLKIDNQSS---- 232
DmelGr8a        PLVWLTYLR-----------NLQFVLHLELLREQLTGLEREMGLLAEYSRFASETGR--- 213
AgamGr5         VYFFALQYILN--------IKLQQIIYPAILLDTYLRMTRAALEHHVGLLQCSERLG--- 260
AgamGr8         FLLLHLRQLQ---------ILLYTNVLGFCLKAINSELAWTIELSNGASRYGGRRSD--- 205
DmelGr9a        LENLRYAYS--------------LQAVRARYLQMMVLVDRLDGKLEQLHHRVISGSS--- 181
DmelGr59f       SINSYVMPN----------IISSISFAQYYLLLQGIAWRQRRLTEGLERELTHLHSP--- 231
DmelGr77a       MYACSLVFVS--------VCQAILQLSLGMYTMAILFLGHLVRHSNLLLAKILADAEHIF 252
DmelGr85a       CFLPECMIFS---------MNYLLFAILAEITRCWWSLQSGLKMVLLNRQLSTVAFN--- 233
TcasGr27        WFPFDYKQP----------VVFDLVYFILAFACISIAYTNVSTDAFFYTCLIQIET---- 198
TcasGr48        IINFGYTLA------------KFMIFTSTCIYTNLLRIIEADFSKLNHLLSGTEN----- 210
AgamGr1         NHLILYGLR--------IYAYLSDTTVCASFGLFALLLRILIVEMRQLVRKTTINEQ--- 223
DmelGr10a       QVRVHFAIY-------AFVLWNYTENMADYCYFINGSVLKYYRQFNLQLGSLRDEMDG-- 228
DmelGr59e       MSLGVLQYAQP---------VHFLWLVMDQMRMCLKELKLLQRPPQGSTKLDACYES--- 211
DmelGr10b       PFNVWDLLAN---------LYFVYNSLARDAILVAYVLLLLNLSEALRLNGQQEHDT--- 207
DmelGr89a       LVAYGVQLY----------FQHVASVYGNGIFGGILLMLECYNQLEREEPTNLAR----- 203
DmelGr57a       LFALCYTIVTG---------GPHFTGYVHMTLAEMLGIRFRLLQQLLQPEFLNWRFP--- 223
DmelGr47b       TLLMAFTYT----------LLTSSVGLLGFVHIGIMDFIRVRLRLVQQLLHQLYQAD--- 221
DmelGr58a       KTAMIVAHIT---------QFIYVVFMMTGICVILLVLHWQSERLQIALKDLCSFLN--- 209
DmelGr58b       NPQIFLAFCAR--------LTHFLHFLCVKMGFFGVLVLLNHQFLVIHLAINALHGR--- 220
DmelGr98a       RFSEFTLYC-----------AVILFIYQELIVGGSNVLDELYRTRYEMWSIRRLS----- 211
AaegGr93a       LMGPIIVCSLT-------ASRYCVIFLTVSCILDDINRDLSRQVDQFHRQHSSANIS--- 224
TcasGr19        AFIIFTMHL----------LFCCAFIFFNMHLLFLLCLDYFTLHLLFLPCIYYFYS---- 217
DmelGr47a       VGAYWLLYN----------MLLAAVLGFYFLLWEMCRIQKLINDQMTLILARSGQRN--- 200
DmelGr58c       PYQSAAIVYALIMILLMSYVDMTVYMVEVAGNWLLVNMTQGVREMVQDLEVLPERNG--- 223
```

```
TcasGr38        ------------------------------------------------------------
TcasGr46        ------------------------------------------------------------
TcasGr1         ------------------------------------------------------------
TcasGr123       ------------------------------------------------------------
TcasGr62        ------------------------------------------------------------
TcasGr25        ------------------------------------------------------------
TcasGr59        ------------------------------------------------------------
AgamGr2         QPWPAEA----------------------------------------------------- 218
AaegGr66a       ------------------------------------------------------------
DmelGr66a       ------------------------------------------------------------
TcasGr11        -------RTMRSFFSDNK------------------------------------------ 218
TcasGr55        -------RTMRSFFSDNK------------------------------------------ 218
TcasGr21        -------EYLETLVQETN------------------------------------------ 224
TcasGr56        -------EYLETLVQETN------------------------------------------ 224
AaegGr          -------KILRSLQEISV------------------------------------------ 224
AaegGr28a       -------KVLCLMRNKTV------------------------------------------ 120
AgamGr33        -------KVLKHLKQAAL------------------------------------------ 222
DmelGr28bB      -------QVLKNLAHQWD------------------------------------------ 224
DmelGr28bC      -------KVLKNLAHQWD------------------------------------------ 251
DmelGr28bD      -------QVLKNLAHQWD------------------------------------------ 221
DmelGr28bA      -------KVLKNLAHQWD------------------------------------------ 233
DmelGr28bE      -------EVLKNLAHQWD------------------------------------------ 228
AgamGr25        -------VALYEMFPECTTLAHTDSSSLDRTSHPLVIF---------------------- 243
AaegGr43a       -------ITLYHVFPECR------------------------------------------ 222
DmelGr43a       -------RMLSSSFLAENN----------------------------------------- 219
AgamGr37b       -------ELFR------------------------------------------------- 210
AgamGr37d       -------QAFR------------------------------------------------- 206
AgamGr37a       -------GCFR------------------------------------------------- 220
AgamGr37c       -------DLFG------------------------------------------------- 214
AgamGr37e       -------NLFR------------------------------------------------- 214
AgamGr37f       -------MAIRLASSTTTLPLIIKPKIHYRCIEQR------------------------- 238
AaegGr28c       -------RIVSLYVYAIITYVMRDSYTRKYSSVILGRAEIFFTVLFYSAIIISIVCAFAY 262
AaegGr28b       -------ATLT------------------------------------------------- 220
AaegGr28e       -------ATLT------------------------------------------------- 220
TcasGr14        ------------------------------------------------------------
TcasGr49        ------------------------------------------------------------
AmelGr4         ------------------------------------------------------------
AmelGr5         ------------------------------------------------------------
TcasGr16        ------------------------------------------------------------
TcasGr22        ------------------------------------------------------------
TcasGr17        ------------------------------------------------------------
TcasGr150       ------------------------------------------------------------
TcasGr32        ------------------------------------------------------------
TcasGr54        ------------------------------------------------------------
TcasGr37        ------------------------------------------------------------
TcasGr53        ------------------------------------------------------------
TcasGr5         ------------------------------------------------------------
TcasGr12        ------------------------------------------------------------
TcasGr13        ------------------------------------------------------------
TcasGr104       ------------------------------------------------------------
TcasGr45        ------------------------------------------------------------
TcasGr98        ------------------------------------------------------------
TcasGr52        ------------------------------------------------------------
TcasGr105       ------------------------------------------------------------
TcasGr4         SVYHLVEIS--------------------------------------------------- 206
TcasGr44        SVYHLVEIS--------------------------------------------------- 210
AgamGr9a        AMSIDRVLL--------------------------------------------------- 220
AgamGr9c        VDSIDGVLL--------------------------------------------------- 217
AgamGr9b        GNQEDDFLT--------------------------------------------------- 269
AgamGr9d        VDSPERFFF--------------------------------------------------- 224
AgamGr9e        HTLDERVR---------------------------------------------------- 216
AgamGr9f        DTLETRLR---------------------------------------------------- 213
AgamGr9g        TIALERIWP--------------------------------------------------- 221
AgamGr9i        AIAPELISP--------------------------------------------------- 221
AgamGr9h        -LPLASELY--------------------------------------------------- 221
AgamGr9j        TACVEHVVH--------------------------------------------------- 255
AgamGr9l        KLNVEHQVE--------------------------------------------------- 245
AgamGr9k        EDEFEYFLG--------------------------------------------------- 235
AgamGr9m        EHDFEQLLC--------------------------------------------------- 238
```

```
AgamGr9n         -EELEQHLD--------------------------------------------------- 206
AgamGr10         -EELEQHLD--------------------------------------------------- 206
AgamGr11         HPAQEKVVY--------------------------------------------------- 208
DmelGr23aA       GVQECLQLQ--------------------------------------------------- 208
DmelGr23aB       SDQQAHRVK--------------------------------------------------- 211
DmelGr39aA       TTT-ISMAG--------------------------------------------------- 211
DmelGr39aB       ESADLKMTSPNR------------------------------------------------ 216
DmelGr39aC       SKESREFYY--------------------------------------------------- 215
DmelGr39aD       GLRGR------------------------------------------------------- 211
DmelGr98c        EDFDCGARIQEL------------------------------------------------ 233
DmelGr98d        EGNQSRDSVQEL------------------------------------------------ 231
DmelGr98b        QDCEQQDMLQAL------------------------------------------------ 231
AgamGr51         ---QGSAAGRQ------------------------------------------------- 245
AgamGr52         -----SGEEKP------------------------------------------------- 237
DmelGr94a        RKDQLKLYTKY------------------------------------------------- 225
DmelGr97a        SPVQMNLHKPY------------------------------------------------- 246
DmelGr93b        RSLNGENNSFR------------------------------------------------- 236
DmelGr93c        RSLERPVGGPV------------------------------------------------- 230
DmelGr22d        KGSSSR------------------------------------------------------ 226
DmelGr22e        TVESER------------------------------------------------------ 229
DmelGr22a        SVDPDR------------------------------------------------------ 228
DmelGr22b        ETDFSR------------------------------------------------------ 227
DmelGr22c        SVDSSR------------------------------------------------------ 224
DmelGr22f        HLSSSR------------------------------------------------------ 220
DmelGr36a        EIYPRRAAFMTK------------------------------------------------ 220
DmelGr36c        LSPRHQGVFMTK------------------------------------------------ 220
DmelGr36b        FLQLRNGAFMTR------------------------------------------------ 220
DmelGr59d        LVPNGGGVFVTK------------------------------------------------ 219
DmelGr59a        ERKSKE------------------------------------------------------ 213
DmelGr59b        -RKHRE------------------------------------------------------ 214
AgamGr26         TDDTRYERRRG--AHEIV------------------------------------------ 243
AgamGr27         ------------------------------------------------------------
AgamGr29         PNTDKSAAYFT------------------------------------------------- 240
AgamGr31         EGTNGSHSRLR------------------------------------------------- 241
AgamGr30         HRSCS------------G------------------------------------------ 235
AgamGr32a        RRTYGKHTTISYIMLQEG------------------------------------------ 247
TcasGr2          ------------------------------------------------------------
TcasGr9          ------------------------------------------------------------
TcasGr7          ------------------------------------------------------------
TcasGr30         ------------------------------------------------------------
TcasGr29         ------------------------------------------------------------
AaegGr64f        ------------------------------------------------------------
AaegGr64a3       ------------------------------------------------------------
AgamGr14         ------------------------------------------------------------
AgamGr18         ------------------------------------------------------------
AaegGr64d        ------------------------------------------------------------
AgamGr17         ------------------------------------------------------------
AaegGr64c        ------------------------------------------------------------
DmelGr64c        ------------------------------------------------------------
DmelGr64d        ------------------------------------------------------------
AgamGr20         ------------------------------------------------------------
AaegGr61a        ------------------------------------------------------------
AgamGr21         ------------------------------------------------------------
AaegGr64a        ------------------------------------------------------------
DmelGr64a        ------------------------------------------------------------
DmelGr61a        ------------------------------------------------------------
AgamGr15         ------------------------------------------------------------
AaegGr1          ------------------------------------------------------------
DmelGr5a         ------------------------------------------------------------
DmelGr64f        ------------------------------------------------------------
DmelGr64e        ------------------------------------------------------------
AmelGr1          ------------------------------------------------------------
AmelGr2          ------------------------------------------------------------
AgamGr16         ------------------------------------------------------------
AaegGr64e        ------------------------------------------------------------
TCasGr6          ------------------------------------------------------------
TcasGr26         ------------------------------------------------------------
TcasGr33         ------------------------------------------------------------
AgamGr23         ------------------------------------------------------------
AaegGr21a        ------------------------------------------------------------
```

```
TcasGr10        ------------------------------------------------------------
TcasGr39        ------------------------------------------------------------
AgamGr22        ------------------------------------------------------------
AaegGr21b       ------------------------------------------------------------
DmelGr21a       ------------------------------------------------------------
AgamGr24        ------------------------------------------------------------
AaegGr63a       ------------------------------------------------------------
DmelGr63a       ------------------------------------------------------------
TcasGr3         ------------------------------------------------------------
DmelOr83b       ------------------------------------------------------------
AgamOr7         ------------------------------------------------------------
HvirOr2         ------------------------------------------------------------
TcasOr16        ------------------------------------------------------------
TcasOr22        ------------------------------------------------------------
AmelOr2         ------------------------------------------------------------
TcasGr31        ------------------------------------------------------------
TcasGr35        ------------------------------------------------------------
DmelGr59c       SIRNRR------------------------------------------------------ 217
DmelGr92a       ------------------------------------------------------------
DmelGr28a       PMHSVVNHRRYS------------------------------------------------ 241
DmelGr64b       ------------------------------------------------------------
TcasGr71        ------------------------------------------------------------
AgamGr19        ------------------------------------------------------------
AaegGr64b       ------------------------------------------------------------
DmelGr93d       ------------------------------------------------------------
TcasGr34        ------------------------------------------------------------
TcasGr43        ------------------------------------------------------------
TcasGr47        ------------------------------------------------------------
AmelGr3         YFRKCPETAAHDMDDKKIWN---------------------------------------- 260
AgamGr32b       VQLRGKPLIYN------------------------------------------------- 243
AgamGr50        ------------------------------------------------------------
TcasGr20        ------------------------------------------------------------
TcasGr79        ------------------------------------------------------------
TcasGr15        N----------------------------------------------------------- 208
TcasGr60        ------------------------------------------------------------
AgamGr38        ------------------------------------------------------------
TcasGr51        VKSFDAIDSKLQTGGKVETSICKNFCFG-------------------------------- 532
DmelGr39b       ------------------------------------------------------------
AgamGr28        ------------------------------------------------------------
TcasGr61        TFAKLLILVVSLSGTFGFFGTHILSYINKASFKKIVNTVATFDETWAKLGLEINHKEDFR 418
TcasGr50        ------------------------------------------------------------
AgamGr12        ------------------------------------------------------------
AgamGr36        ------------------------------------------------------------
AgamGr48        ------------------------------------------------------------
AgamGr49a       ------------------------------------------------------------
AgamGr49b       QLR--------------------------------------------------------- 226
DmelGr2a        ------------------------------------------------------------
DmelGr32a       RRQRGD------------------------------------------------------ 277
DmelGr68a       ------------------------------------------------------------
AmelGr7         ------------------------------------------------------------
AmelGr8         ------------------------------------------------------------
AmelGr9         ------------------------------------------------------------
TcasGr57        ------------------------------------------------------------
TcasGr41        ------------------------------------------------------------
AgamGr34        MPANEP------------------------------------------------------ 227
AgamGr35        ------------------------------------------------------------
AgamGr39        TE---------------------------------------------------------- 241
AgamGr40        ------------------------------------------------------------
AaegGr28d       ------------------------------------------------------------
AgamGr42        ------------------------------------------------------------
AgamGr41        ------------------------------------------------------------
AgamGr43        ------------------------------------------------------------
DmelGr33a       TSNDEDEDD--------------------------------------------------- 279
AgamGr44        ------------------------------------------------------------
AgamGr13        ------------------------------------------------------------
AgamGr45        ------------------------------------------------------------
AgamGr46        LTAYG------------------------------------------------------- 244
Dmelr93a        ------------------------------------------------------------
AmelGr6         ------------------------------------------------------------
AmelGr10        ------------------------------------------------------------
```

```
TcasGr28       ------------------------------------------------------------
TcasGr40       ------------------------------------------------------------
AgamGr3        ------------------------------------------------------------
AgamGr4        ------------------------------------------------------------
AgamGr6        ------------------------------------------------------------
AgamGr7        ------------------------------------------------------------
AgamGr47       ------------------------------------------------------------
DmelGr8a       ------------------------------------------------------------
AgamGr5        ------------------------------------------------------------
AgamGr8        ------------------------------------------------------------
DmelGr9a       ------------------------------------------------------------
DmelGr59f      ------------------------------------------------------------
DmelGr77a      ESSQKAGFWPNR------------------------------------------------ 264
DmelGr85a      ------------------------------------------------------------
TcasGr27       ------------------------------------------------------------
TcasGr48       ------------------------------------------------------------
AgamGr1        ------------------------------------------------------------
DmelGr10a      ------------------------------------------------------------
DmelGr59e      ------------------------------------------------------------
DmelGr10b      ------------------------------------------------------------
DmelGr89a      ------------------------------------------------------------
DmelGr57a      ------------------------------------------------------------
DmelGr47b      ------------------------------------------------------------
DmelGr58a      ------------------------------------------------------------
DmelGr58b      ------------------------------------------------------------
DmelGr98a      ------------------------------------------------------------
AaegGr93a      ------------------------------------------------------------
TcasGr19       ------------------------------------------------------------
DmelGr47a      ------------------------------------------------------------
DmelGr58c      ------------------------------------------------------------


TcasGr38       ------------------------------------------------------------
TcasGr46       ------------------------------------------------------------
TcasGr1        ------------------------------------------------------------
TcasGr123      ------------------------------------------------------------
TcasGr62       ------------------------------------------------------------
TcasGr25       ------------------------------------------------------------
TcasGr59       ------------------------------------------------------------
AgamGr2        ------------------------------------------------------------
AaegGr66a      ------------------------------------------------------------
DmelGr66a      ------------------------------------------------------------
TcasGr11       ------------------------------------------------------------
TcasGr55       ------------------------------------------------------------
TcasGr21       ------------------------------------------------------------
TcasGr56       ------------------------------------------------------------
AaegGr         ------------------------------------------------------------
AaegGr28a      ------------------------------------------------------------
AgamGr33       ------------------------------------------------------------
DmelGr28bB     ------------------------------------------------------------
DmelGr28bC     ------------------------------------------------------------
DmelGr28bD     ------------------------------------------------------------
DmelGr28bA     ------------------------------------------------------------
DmelGr28bE     ------------------------------------------------------------
AgamGr25       ------------------------------------------------------------
AaegGr43a      ------------------------------------------------------------
DmelGr43a      ------------------------------------------------------------
AgamGr37b      ------------------------------------------------------------
AgamGr37d      ------------------------------------------------------------
AgamGr37a      ------------------------------------------------------------
AgamGr37c      ------------------------------------------------------------
AgamGr37e      ------------------------------------------------------------
AgamGr37f      ------------------------------------------------------------
AaegGr28c      RNTNLQVLQKLNRMDQLLQEKLRTRIDHGKVHSLMIVGIACHFIMTFIICGSYETVHVTV 322
AaegGr28b      ------------------------------------------------------------
AaegGr28e      ------------------------------------------------------------
TcasGr14       ------------------------------------------------------------
TcasGr49       ------------------------------------------------------------
AmelGr4        ------------------------------------------------------------
AmelGr5        ------------------------------------------------------------
```

```
TcasGr16        ------------------------------------------------------------
TcasGr22        ------------------------------------------------------------
TcasGr17        ------------------------------------------------------------
TcasGr150       ------------------------------------------------------------
TcasGr32        ------------------------------------------------------------
TcasGr54        ------------------------------------------------------------
TcasGr37        ------------------------------------------------------------
TcasGr53        ------------------------------------------------------------
TcasGr5         ------------------------------------------------------------
TcasGr12        ------------------------------------------------------------
TcasGr13        ------------------------------------------------------------
TcasGr104       ------------------------------------------------------------
TcasGr45        ------------------------------------------------------------
TcasGr98        ------------------------------------------------------------
TcasGr52        ------------------------------------------------------------
TcasGr105       ------------------------------------------------------------
TcasGr4         ------------------------------------------------------------
TcasGr44        ------------------------------------------------------------
AgamGr9a        ------------------------------------------------------------
AgamGr9c        ------------------------------------------------------------
AgamGr9b        ------------------------------------------------------------
AgamGr9d        ------------------------------------------------------------
AgamGr9e        ------------------------------------------------------------
AgamGr9f        ------------------------------------------------------------
AgamGr9g        ------------------------------------------------------------
AgamGr9i        ------------------------------------------------------------
AgamGr9h        ------------------------------------------------------------
AgamGr9j        ------------------------------------------------------------
AgamGr9l        ------------------------------------------------------------
AgamGr9k        ------------------------------------------------------------
AgamGr9m        ------------------------------------------------------------
AgamGr9n        ------------------------------------------------------------
AgamGr10        ------------------------------------------------------------
AgamGr11        ------------------------------------------------------------
DmelGr23aA      ------------------------------------------------------------
DmelGr23aB      ------------------------------------------------------------
DmelGr39aA      ------------------------------------------------------------
DmelGr39aB      ------------------------------------------------------------
DmelGr39aC      ------------------------------------------------------------
DmelGr39aD      ------------------------------------------------------------
DmelGr98c       ------------------------------------------------------------
DmelGr98d       ------------------------------------------------------------
DmelGr98b       ------------------------------------------------------------
AgamGr51        ------------------------------------------------------------
AgamGr52        ------------------------------------------------------------
DmelGr94a       ------------------------------------------------------------
DmelGr97a       ------------------------------------------------------------
DmelGr93b       ------------------------------------------------------------
DmelGr93c       ------------------------------------------------------------
DmelGr22d       ------------------------------------------------------------
DmelGr22e       ------------------------------------------------------------
DmelGr22a       ------------------------------------------------------------
DmelGr22b       ------------------------------------------------------------
DmelGr22c       ------------------------------------------------------------
DmelGr22f       ------------------------------------------------------------
DmelGr36a       ------------------------------------------------------------
DmelGr36c       ------------------------------------------------------------
DmelGr36b       ------------------------------------------------------------
DmelGr59d       ------------------------------------------------------------
DmelGr59a       ------------------------------------------------------------
DmelGr59b       ------------------------------------------------------------
AgamGr26        ------------------------------------------------------------
AgamGr27        ------------------------------------------------------------
AgamGr29        ------------------------------------------------------------
AgamGr31        ------------------------------------------------------------
AgamGr30        ------------------------------------------------------------
AgamGr32a       ------------------------------------------------------------
TcasGr2         ------------------------------------------------------------
TcasGr9         ------------------------------------------------------------
TcasGr7         ------------------------------------------------------------
```

```
TcasGr30        ------------------------------------------------------------
TcasGr29        ------------------------------------------------------------
AaegGr64f       ------------------------------------------------------------
AaegGr64a3      ------------------------------------------------------------
AgamGr14        ------------------------------------------------------------
AgamGr18        ------------------------------------------------------------
AaegGr64d       ------------------------------------------------------------
AgamGr17        ------------------------------------------------------------
AaegGr64c       ------------------------------------------------------------
DmelGr64c       ------------------------------------------------------------
DmelGr64d       ------------------------------------------------------------
AgamGr20        ------------------------------------------------------------
AaegGr61a       ------------------------------------------------------------
AgamGr21        ------------------------------------------------------------
AaegGr64a       ------------------------------------------------------------
DmelGr64a       ------------------------------------------------------------
DmelGr61a       ------------------------------------------------------------
AgamGr15        ------------------------------------------------------------
AaegGr1         ------------------------------------------------------------
DmelGr5a        ------------------------------------------------------------
DmelGr64f       ------------------------------------------------------------
DmelGr64e       ------------------------------------------------------------
AmelGr1         ------------------------------------------------------------
AmelGr2         ------------------------------------------------------------
AgamGr16        ------------------------------------------------------------
AaegGr64e       ------------------------------------------------------------
TCasGr6         ------------------------------------------------------------
TcasGr26        ------------------------------------------------------------
TcasGr33        ------------------------------------------------------------
AgamGr23        ------------------------------------------------------------
AaegGr21a       ------------------------------------------------------------
TcasGr10        ------------------------------------------------------------
TcasGr39        ------------------------------------------------------------
AgamGr22        ------------------------------------------------------------
AaegGr21b       ------------------------------------------------------------
DmelGr21a       ------------------------------------------------------------
AgamGr24        ------------------------------------------------------------
AaegGr63a       ------------------------------------------------------------
DmelGr63a       ------------------------------------------------------------
TcasGr3         ------------------------------------------------------------
DmelOr83b       ------------------------------------------------------------
AgamOr7         ------------------------------------------------------------
HvirOr2         ------------------------------------------------------------
TcasOr16        ------------------------------------------------------------
TcasOr22        ------------------------------------------------------------
AmelOr2         ------------------------------------------------------------
TcasGr31        ------------------------------------------------------------
TcasGr35        ------------------------------------------------------------
DmelGr59c       ------------------------------------------------------------
DmelGr92a       ------------------------------------------------------------
DmelGr28a       ------------------------------------------------------------
DmelGr64b       ------------------------------------------------------------
TcasGr71        ------------------------------------------------------------
AgamGr19        ------------------------------------------------------------
AaegGr64b       ------------------------------------------------------------
DmelGr93d       ------------------------------------------------------------
TcasGr34        ------------------------------------------------------------
TcasGr43        ------------------------------------------------------------
TcasGr47        ------------------------------------------------------------
AmelGr3         ------------------------------------------------------------
AgamGr32b       ------------------------------------------------------------
AgamGr50        ------------------------------------------------------------
TcasGr20        ------------------------------------------------------------
TcasGr79        ------------------------------------------------------------
TcasGr15        ------------------------------------------------------------
TcasGr60        ------------------------------------------------------------
AgamGr38        ------------------------------------------------------------
TcasGr51        ------------------------------------------------------------
DmelGr39b       ------------------------------------------------------------
AgamGr28        ------------------------------------------------------------
```

```
TcasGr61        VCLVFTLTGPFFTFCNVLMEMWNIPRENIDPIPLVVVLTHLIPFMLIHQG---------- 468
TcasGr50        ------------------------------------------------------------
AgamGr12        ------------------------------------------------------------
AgamGr36        ------------------------------------------------------------
AgamGr48        ------------------------------------------------------------
AgamGr49a       ------------------------------------------------------------
AgamGr49b       ------------------------------------------------------------
DmelGr2a        ------------------------------------------------------------
DmelGr32a       ------------------------------------------------------------
DmelGr68a       ------------------------------------------------------------
AmelGr7         ------------------------------------------------------------
AmelGr8         ------------------------------------------------------------
AmelGr9         ------------------------------------------------------------
TcasGr57        ------------------------------------------------------------
TcasGr41        ------------------------------------------------------------
AgamGr34        ------------------------------------------------------------
AgamGr35        ------------------------------------------------------------
AgamGr39        ------------------------------------------------------------
AgamGr40        ------------------------------------------------------------
AaegGr28d       ------------------------------------------------------------
AgamGr42        ------------------------------------------------------------
AgamGr41        ------------------------------------------------------------
AgamGr43        ------------------------------------------------------------
DmelGr33a       ------------------------------------------------------------
AgamGr44        ------------------------------------------------------------
AgamGr13        ------------------------------------------------------------
AgamGr45        ------------------------------------------------------------
AgamGr46        ------------------------------------------------------------
Dmelr93a        ------------------------------------------------------------
AmelGr6         ------------------------------------------------------------
AmelGr10        ------------------------------------------------------------
TcasGr28        ------------------------------------------------------------
TcasGr40        ------------------------------------------------------------
AgamGr3         ------------------------------------------------------------
AgamGr4         ------------------------------------------------------------
AgamGr6         ------------------------------------------------------------
AgamGr7         ------------------------------------------------------------
AgamGr47        ------------------------------------------------------------
DmelGr8a        ------------------------------------------------------------
AgamGr5         ------------------------------------------------------------
AgamGr8         ------------------------------------------------------------
DmelGr9a        ------------------------------------------------------------
DmelGr59f       ------------------------------------------------------------
DmelGr77a       ------------------------------------------------------------
DmelGr85a       ------------------------------------------------------------
TcasGr27        ------------------------------------------------------------
TcasGr48        ------------------------------------------------------------
AgamGr1         ------------------------------------------------------------
DmelGr10a       ------------------------------------------------------------
DmelGr59e       ------------------------------------------------------------
DmelGr10b       ------------------------------------------------------------
DmelGr89a       ------------------------------------------------------------
DmelGr57a       ------------------------------------------------------------
DmelGr47b       ------------------------------------------------------------
DmelGr58a       ------------------------------------------------------------
DmelGr58b       ------------------------------------------------------------
DmelGr98a       ------------------------------------------------------------
AaegGr93a       ------------------------------------------------------------
TcasGr19        ------------------------------------------------------------
DmelGr47a       ------------------------------------------------------------
DmelGr58c       ------------------------------------------------------------


TcasGr38        ---------------------------------------------------KQIRFLNQY 223
TcasGr46        ---------------------------------------------------KQIRFLNQY 205
TcasGr1         ---------------------------------------------------KQLTKITVK 208
TcasGr123       ---------------------------------------------------KQINNLIEY 206
TcasGr62        ---------------------------------------------------KQINNLIEY 481
TcasGr25        ---------------------------------------------------------FHD 103
TcasGr59        ---------------------------------------------------------FHD 278
```

```
AgamGr2         ---------------------------------------------------DAVGYGPAQ 227
AaegGr66a       ---------------------------------------------------------AVF 291
DmelGr66a       ---------------------------------------------------------PLA 305
TcasGr11        ----------------------------------------QDDKIPQISDNLYILCR--- 235
TcasGr55        ----------------------------------------QDDKIPQISDNLYILCR--- 235
TcasGr21        ----------------------------------------RTSVEGWTDVSNVKRKSKEI 244
TcasGr56        ----------------------------------------RTSVEGWTDVSNVKRKSKEI 244
AaegGr          ----------------------------------------DKRRLSFDEKQNEVKIVKIQ 244
AaegGr28a       ----------------------------------------DKVLLQFTN-QHVNEQVKIQ 139
AgamGr33        ----------------------------------------EKR-ISPQRRPSYWHAIKIQ 241
DmelGr28bB      ----------------------------------------TRSLKAVNQKQRSLQCLDSF 244
DmelGr28bC      ----------------------------------------TRSLKAVNQKQRSLQCLDSF 271
DmelGr28bD      ----------------------------------------TRSLKAVNQKQRSLQCLDSF 241
DmelGr28bA      ----------------------------------------TRSLKAVNQKQRSLQCLDSF 253
DmelGr28bE      ----------------------------------------TRSLKAVNQKQRSLQCLDSF 248
AgamGr25        -----------------------------SVGRTPRIEISSINLDSDKNDPIDVDLRKCV 274
AaegGr43a       ------------------------------------------------------------
DmelGr43a       -----------------------------ATSAIKPQKVSTVKNVSVNRPAMPSALHASL 250
AgamGr37b       -----------------------------------------------MYFPTSPSAVGNP 223
AgamGr37d       -----------------------------------------------MYFPTSPSAVGNP 219
AgamGr37a       -----------------------------------------------MYFPTSPSAVGNP 233
AgamGr37c       -----------------------------------------------MYFPTSPSAVGNP 227
AgamGr37e       -----------------------------------------------MYFPTSPSAVGNP 227
AgamGr37f       --------------------------------------------ACRMYFPTSPSAVGNP 254
AaegGr28c       FAKLGIVKARILFHFICGFAFMLTIVVPIVQVILLVARFQALNNAFRMYFPTTPSCGGFL 382
AaegGr28b       ----------------------------------------------AQFVPPKKSSMVLL 234
AaegGr28e       ----------------------------------------------AHFVPPKKSSMVLL 234
TcasGr14        ------------------------------------------------VDLFVIFPLTKA 195
TcasGr49        ------------------------------------------------VDLFVIFPLTKA 239
AmelGr4         ------------------------------------------------MQNFYQNRLDDF 223
AmelGr5         ------------------------------------------------IQSFCRTRYDDI 223
TcasGr16        ------------------------------------------------IQALINDSRKVT 218
TcasGr22        ----------------------------------------------------IITRGDVT 129
TcasGr17        ---------------------------------------------------IIDSIRQNS 205
TcasGr150       ---------------------------------------------------IIDSIRQNS 205
TcasGr32        ---------------------------------------------------------KQL 183
TcasGr54        ---------------------------------------------------------KQL 183
TcasGr37        ---------------------------------------------------------ACL 168
TcasGr53        ---------------------------------------------------------ACL 168
TcasGr5         ---------------------------------------------------------YVM 179
TcasGr12        ---------------------------------------------------------RQT 173
TcasGr13        ---------------------------------------------------------RQT 247
TcasGr104       ---------------------------------------------------------NLF 204
TcasGr45        ---------------------------------------------------------NLF 204
TcasGr98        ---------------------------------------------------------EQM 202
TcasGr52        ---------------------------------------------------------KAL 143
TcasGr105       ---------------------------------------------------------KYL 197
TcasGr4         ------------------------------------------------------------
TcasGr44        ------------------------------------------------------------
AgamGr9a        ------------------------------------------------------------
AgamGr9c        ------------------------------------------------------------
AgamGr9b        ------------------------------------------------------------
AgamGr9d        ------------------------------------------------------------
AgamGr9e        ------------------------------------------------------------
AgamGr9f        ------------------------------------------------------------
AgamGr9g        ------------------------------------------------------------
AgamGr9i        ------------------------------------------------------------
AgamGr9h        ------------------------------------------------------------
AgamGr9j        ------------------------------------------------------------
AgamGr9l        ------------------------------------------------------------
AgamGr9k        ------------------------------------------------------------
AgamGr9m        ------------------------------------------------------------
AgamGr9n        ------------------------------------------------------------
AgamGr10        ------------------------------------------------------------
AgamGr11        ------------------------------------------------------------
DmelGr23aA      ------------------------------------------------------------
DmelGr23aB      ------------------------------------------------------------
DmelGr39aA      ------------------------------------------------------------
DmelGr39aB      ------------------------------------------------------------
DmelGr39aC      ------------------------------------------------------------
```

```
DmelGr39aD      ------------------------------------------------------------
DmelGr98c       ------------------------------------------------------------
DmelGr98d       ------------------------------------------------------------
DmelGr98b       ------------------------------------------------------------
AgamGr51        ------------------------------------------------------------
AgamGr52        ------------------------------------------------------------
DmelGr94a       ------------------------------------------------------------
DmelGr97a       ------------------------------------------------------------
DmelGr93b       ------------------------------------------------------------
DmelGr93c       ------------------------------------------------------------
DmelGr22d       ------------------------------------------------------------
DmelGr22e       ------------------------------------------------------------
DmelGr22a       ------------------------------------------------------------
DmelGr22b       ------------------------------------------------------------
DmelGr22c       ------------------------------------------------------------
DmelGr22f       ------------------------------------------------------------
DmelGr36a       ------------------------------------------------------------
DmelGr36c       ------------------------------------------------------------
DmelGr36b       ------------------------------------------------------------
DmelGr59d       ------------------------------------------------------------
DmelGr59a       ------------------------------------------------------------
DmelGr59b       ------------------------------------------------------------
AgamGr26        ------------------------------------------------------------
AgamGr27        ------------------------------------------------------------
AgamGr29        ------------------------------------------------------------
AgamGr31        ------------------------------------------------------------
AgamGr30        ------------------------------------------------------------
AgamGr32a       ------------------------------------------------------------
TcasGr2         ------------------------------------------------------------
TcasGr9         ------------------------------------------------------------
TcasGr7         ------------------------------------------------------------
TcasGr30        ------------------------------------------------------------
TcasGr29        ------------------------------------------------------------
AaegGr64f       ------------------------------------------------------------
AaegGr64a3      ------------------------------------------------------------
AgamGr14        ------------------------------------------------------------
AgamGr18        ------------------------------------------------------------
AaegGr64d       ------------------------------------------------------------
AgamGr17        ------------------------------------------------------------
AaegGr64c       ------------------------------------------------------------
DmelGr64c       ------------------------------------------------------------
DmelGr64d       ------------------------------------------------------------
AgamGr20        ------------------------------------------------------------
AaegGr61a       ------------------------------------------------------------
AgamGr21        ------------------------------------------------------------
AaegGr64a       ------------------------------------------------------------
DmelGr64a       ------------------------------------------------------------
DmelGr61a       ------------------------------------------------------------
AgamGr15        ------------------------------------------------------------
AaegGr1         ------------------------------------------------------------
DmelGr5a        ------------------------------------------------------------
DmelGr64f       ------------------------------------------------------------
DmelGr64e       ------------------------------------------------------------
AmelGr1         ------------------------------------------------------------
AmelGr2         ------------------------------------------------------------
AgamGr16        ------------------------------------------------------------
AaegGr64e       ------------------------------------------------------------
TCasGr6         ---------------------------------------------------ASNSKSTKV 210
TcasGr26        ---------------------------------------------------ASNSKSTKV 210
TcasGr33        ---------------------------------------------------ASNSKSTKV 210
AgamGr23        ------------------------------------------------------------
AaegGr21a       ------------------------------------------------------------
TcasGr10        ------------------------------------------------------------
TcasGr39        ------------------------------------------------------------
AgamGr22        ------------------------------------------------------------
AaegGr21b       ------------------------------------------------------------
DmelGr21a       ------------------------------------------------------------
AgamGr24        ------------------------------------------------------------
AaegGr63a       ------------------------------------------------------------
DmelGr63a       ------------------------------------------------------------
```

```
TcasGr3         ------------------------------------------------------------
DmelOr83b       ---------------------------------------------------NGLVNGAN- 322
AgamOr7         ---------------------------------------------------N-------- 314
HvirOr2         ------------------------------------------------------------
TcasOr16        ------------------------------------------------------------
TcasOr22        ------------------------------------------------------------
AmelOr2         ------------------------------------------------------------
TcasGr31        ------------------------------------------------------------
TcasGr35        ------------------------------------------------------------
DmelGr59c       ------------------------------------------------------------
DmelGr92a       ------------------------------------------------------------
DmelGr28a       -----------------------------------------------------HRLRNLI 248
DmelGr64b       ------------------------------------------------------------
TcasGr71        ------------------------------------------------------------
AgamGr19        ------------------------------------------------------------
AaegGr64b       ------------------------------------------------------------
DmelGr93d       ------------------------------------------------------------
TcasGr34        ------------------------------------------------------------
TcasGr43        ------------------------------------------------------------
TcasGr47        ------------------------------------------------------------
AmelGr3         -----------------------------------------------------LKPKRQI 267
AgamGr32b       ------------------------------------------------------------
AgamGr50        ------------------------------------------------------------
TcasGr20        ------------------------------------------------------------
TcasGr79        ------------------------------------------------------------
TcasGr15        ------------------------------------------------------------
TcasGr60        ------------------------------------------------------------
AgamGr38        ------------------------------------------------------------
TcasGr51        -FITKQLIFMIFVTYQLYCWSSFLKISVLQSLWASPTCDLMYEFQIFTFLMSLLQSIKRR 591
DmelGr39b       ------------------------------------------------------------
AgamGr28        ------------------------------------------------------------
TcasGr61        ------------------------------------ETQFVVANIILRRRFALINSILRK 492
TcasGr50        ------------------------------------------------------------
AgamGr12        ------------------------------------------------------------
AgamGr36        ------------------------------------------------------------
AgamGr48        ------------------------------------------------------------
AgamGr49a       ------------------------------------------------------------
AgamGr49b       ------------------------------------------------------------
DmelGr2a        ------------------------------------------------------------
DmelGr32a       ------------------------------------------------------------
DmelGr68a       ------------------------------------------------------------
AmelGr7         ------------------------------------------------------------
AmelGr8         ------------------------------------------------------------
AmelGr9         ------------------------------------------------------------
TcasGr57        ------------------------------------------------------------
TcasGr41        ------------------------------------------------------------
AgamGr34        --------------------------------------------------------KKMS 231
AgamGr35        ------------------------------------------------------------
AgamGr39        ------------------------------------------------------------
AgamGr40        ------------------------------------------------------------
AaegGr28d       ------------------------------------------------------------
AgamGr42        -----------------------------------------------------------I 237
AgamGr41        ----------------------------------------------------------DF 239
AgamGr43        ------------------------------------------------------------
DmelGr33a       ------------------------------------------------------------
AgamGr44        ------------------------------------------------------------
AgamGr13        ------------------------------------------------------------
AgamGr45        ------------------------------------------------------------
AgamGr46        ----------------------------------------------------------TD 246
Dmelr93a        ------------------------------------------------------------
AmelGr6         ------------------------------------------------------------
AmelGr10        ------------------------------------------------------------
TcasGr28        ------------------------------------------------------------
TcasGr40        ------------------------------------------------------------
AgamGr3         ------------------------------------------------------------
AgamGr4         ------------------------------------------------------------
AgamGr6         ------------------------------------------------------------
AgamGr7         ------------------------------------------------------------
AgamGr47        ------------------------------------------------------------
DmelGr8a        ------------------------------------------------------------
```

```
AgamGr5         ------------------------------------------------------------
AgamGr8         ------------------------------------------------------------
DmelGr9a        ------------------------------------------------------------
DmelGr59f       ------------------------------------------------------------
DmelGr77a       -----------------------------------------------------------Q 265
DmelGr85a       ------------------------------------------------------------
TcasGr27        ------------------------------------------------------------
TcasGr48        ------------------------------------------------------------
AgamGr1         ------------------------------------------------------------
DmelGr10a       ------------------------------------------------------------
DmelGr59e       ------------------------------------------------------------
DmelGr10b       ------------------------------------------------------------
DmelGr89a       ------------------------------------------------------------
DmelGr57a       ------------------------------------------------------------
DmelGr47b       ------------------------------------------------------------
DmelGr58a       ------------------------------------------------------------
DmelGr58b       ------------------------------------------------------------
DmelGr98a       ------------------------------------------------------------
AaegGr93a       ------------------------------------------------------------
TcasGr19        ------------------------------------------------------------
DmelGr47a       ------------------------------------------------------------
DmelGr58c       ------------------------------------------------------------


TcasGr38        LRLKPEGRISNR---RVFISFSKICYLHQHLSKSVKLFNEVFGV--SLLVLFGNSFLSIV 278
TcasGr46        LRLKPEGRISNR---RVFISFSKICYLHQHLSKSVKLFNEVFGV--SLLVLFGNSFLSIV 260
TcasGr1         N-------FATK---TQSAVLGKICTLHHHLSKLVTRFNEIFGL--GLLLMFAVSFLIIT 256
TcasGr123       FQKNKIGPVETKGTNKQLNTLNKICALHHHLSKLVKLFNETFGI--VLLLMFGVSFVVIV 264
TcasGr62        FQKNKIGPVETKGTNKQLNTLNKICALHHHLSKLVKLFNETFGI--VLLLMFGVSFVVIV 539
TcasGr25        IFLPLLCILT-------NTLEILITVTVCELTILEYKRTKKLLY--RIPVTKTDSMLIRN 154
TcasGr59        IFLPLLCILT-------NTLEILITVTVCELTILEYKRTKKLLY--RIPVTKTDSMLIRN 329
AgamGr2         EFLPTDRRLRPHIEQRLDNKLILFCRTHDELCEIGKVVNRMYSV--QMLVAMAHGFVAIT 285
AaegGr66a       EFLAFDKYMK--IDEKLDKKMILICRTHDELCEIGKSVNYMFSF--QMLVSMAHGFMAIT 347
DmelGr66a       TNMVHESELG--NAAKVEEKLNNLCQVHDEICEIGKALNELWSY--PILSLMAYGFLIFT 361
TcasGr11        --------------------------LHYKLTSVMQKINSAFSV--QLLVSIGVSLFDVL 267
TcasGr55        --------------------------LHYKLTSVMQKINSAFSV--QLLVSIGVSLFDVL 267
TcasGr21        SKLPKSMLAIS----DPVFIVDQVAALHIKLTDTAHMINYAFCV--QQLLRITVAFISIV 298
TcasGr56        SKLPKSMLAIS----DPVFIVDQVAALHIKLTDTAHMINYAFCV--QQLLRITVAFISIV 298
AaegGr          KPLNISSVFSN-TNRYMPDVINQVASIQSEISDACDHVEDYFKV--QMLTIVTISFLISV 301
AaegGr28a       NPFGVSSVFSVKTNRQMPNVINRVASIQSEICDACDCVEDYFKV--QMLTIVAISFLISV 197
AgamGr33        RPLGIASVYSN-NDKSMPDVVSTVAEIQDALCEACSYAEDYFTI--QMLTIVTIVFLIIV 298
DmelGr28bB      SMYTIVTKDPA-------EIIQESMEIHHLICEAAATANKYFTY--QLLTIISIAFLIIV 295
DmelGr28bC      SMYTIVTKDPA-------EIIQESMEIHHLICEAAATANKYFTY--QLLTIISIAFLIIV 322
DmelGr28bD      SMYTIVTKDPA-------EIIQESMEIHHLICEAAATANKYFTY--QLLTIISIAFLIIV 292
DmelGr28bA      SMYTIVTKDPA-------EIIQESMEIHHLICEAAATANKYFTY--QLLTIISIAFLIIV 304
DmelGr28bE      SMYTIVTKDPA-------EIIQESMEIHHLICEAAATANKYFTY--QLLTIISIAFLIIV 299
AgamGr25        ANVAHGFDRKRTLSSRK--IIEDLAVIHASLSYCIVLVSKTFGI--ALLAVLGSCLLHLV 330
AaegGr43a       --------KRTTIPPNR--IIEDLAYIHASLSTGVVLVSNTFGV--ALLAVLGSCLLHLV 270
DmelGr43a       TKLNGETLPSEAAAKNKGLLLKSLADSHESLGKCVHLLSNSFGI--AVLFILVSCLLHLV 308
AgamGr37b       S-----EFAICS-EKEQINVLKQIKILHDKLNDVVELVNYCFSV--QGK-RIGILVHKAI 274
AgamGr37d       S-----EFAICS-EKEQINVLKQIKILHDKLNDVVELVNYCFSV--QGK-RIGILVHKAI 270
AgamGr37a       S-----EFAICS-EKEQINVLKQIKILHDKLNDVVELVNYCFSV--QGK-RIGILVHKAI 284
AgamGr37c       S-----EFAICS-EKEQINVLKQIKILHDKLNDVVELVNYCFSV--QGK-RIGILVHKAI 278
AgamGr37e       S-----EFAICS-EKEQINVLKQIKILHDKLNDVVELVNYCFSV--QGK-RIGILVHKAI 278
AgamGr37f       S-----EFAICS-EKEQINVLKQIKILHDKLNDVVELVNYCFSV--QGK-RIGILVHKAI 305
AaegGr28c       SSTSHTETVICN-EKEQLAVLKQIKILHDKLNDVVELVNYCFSV--QITFCVGLCFVIGV 439
AaegGr28b       D-----ELPVPKSNYNKSVVLHQIQLQWTEICQVVELISKTYYR--QIIFIATASTIMST 287
AaegGr28e       D-----ELPVPKSNYNKSVVLHQIQLQWTEISQVVELISKTYYR--QIIFIATASTIMST 287
TcasGr14        EKVRNLKEDEVTFSVQKIQ---ELSHLHYKLANFTVKISGLFEI--TTITAMVMWFGYVI 250
TcasGr49        EKVRNLKEDEVTFSVQKIQ---ELSHLHYKLANFTVKISGLFEI--TTITAMVMWFGYVI 294
AmelGr4         NSNILYQNRRIFLNCSRIHLLLQIRNIHDHLCNISREISQFYAF--PTLTGLCFIFFTSL 281
AmelGr5         NFKILNQTRCVFMSYSKIQLFIQIRDIYDHLCDISREVSDFYSF--SILTALSFIFLVIL 281
TcasGr16        RFPTELTFCNKIK---------HLVNLHKNLVKVAKDHNSLYSL--HLLLWITVTFILLV 267
TcasGr22        --------STTIK---------KLFNLHKILVKVVREINGIFAF--QVLMCTSMNFVLLI 170
TcasGr17        QTRNQAVPTGTIK---------TLVQMHSNLVTTSEEVCDSFSI--FGLVWISLIFVVLI 254
TcasGr150       QTRNQAVPTGTIK---------TLVQMHSNLVTTSEEVCDSFSI--FGLVWISLIFVVLI 254
TcasGr32        RFKLRFFEKLGNRRLGFVQRLDQIQFDICLLKEGVDLVNDIFGW--QILFLITYATLQIL 241
TcasGr54        RFKLRFFEKLGNRRLGFVQRLDQIQFDICLLKEGVDLVNDIFGW--QILFLITYATLQIL 241
TcasGr37        ---------MEEKRP--MVSLQVIKAQIYSLRETVDVFNDIFGW--PFLLLITFTSLQIM 215
TcasGr53        ---------MEEKRP--MVSLQVIKAQIYSLRETVDVFNDIFGW--PFLLLITFTSLQIM 215
```

```
TcasGr5        T---------GQLKAQNSNLLKSVCADLCLLSECVDLFNSLFGF--LVLLLVALTTLQLL 228
TcasGr12       -----------LDTADVKTLTTVVEKECYKMKVTIDAFNDIFGW--SFLLFTLYSSLLLL 220
TcasGr13       -----------LDTADVKTLTTVVEKECYKMKVTIDAFNDIFGW--SFLLFTLYSSLLLL 294
TcasGr104      RIE-------ESEEKNIIVSLRQIGSLSQLLSETVALFNDVFGT--CLILITGKSIVQLL 255
TcasGr45       RIE-------ESEEKNIIVSLRQIGSLSQLLSETVALFNDVFGT--CLILITGKSIVQLL 255
TcasGr98       AVR-------INP-KNCASFVIEVRKLSRLLGEMVGLFNDIFGW--PLVFITGRFVIKIL 252
TcasGr52       ES-----------SKISVGLIRDTAVLYRLLADNVANFNKIFGW--SLIFLFGRIVLQLL 190
TcasGr105      KIE-------TS--------CRYVGTLYRILTTMMEMVNDIFGW--TLILILAKCITSSL 240
TcasGr4        ------------------------EIHFDLCQAADDLN-RYFEV--QVLTIFGVSFYFFI 239
TcasGr44       ------------------------EIHFDLCQAADDLN-RYFEV--QVLTIFGVSFYFFI 243
AgamGr9a       ---------------------TYVTTYNSIAAVLGDHLCSYFGP--LVLLHCSYTCLEAA 257
AgamGr9c       ---------------------TYVSMYNSITNVLRNNLTPYFGP--IILLHSSYVCLEAA 254
AgamGr9b       ---------------------FYGTMYCKIAVVLSDHLGPYFGL--IILMHCSYVCFEAA 306
AgamGr9d       ---------------------RYGVIHNRTAFAIRNHLSHYFGP--MLAVFCTYVSLEVA 261
AgamGr9e       ---------------------VFCKTYERVCRAVARSFFEYTGP--VIATFCTLVMFEGS 253
AgamGr9f       ---------------------LFCRTYDRVCTRVAQPFYEYCGP--VVATFCPLLIFEGS 250
AgamGr9g       ---------------------PLWQVYDRVTIDVRKDLSQYYGP--IVLLFSCMIGLESA 258
AgamGr9i       ---------------------ILWDVYDRVTIHVRKDLSQYCGP--IVVLFTSLFVLECA 258
AgamGr9h       ---------------------MYFKLIDRITVLINFHLSQIYGS--FVVFHCMYVLFESA 258
AgamGr9j       ---------------------VFLDRFQRYYLLID-SVNKCFSV--PVTHTLLLIVLERT 291
AgamGr9l       ---------------------LFIKRFEYYYELIE-CINRCFAI--PLIHVLLLILLERT 281
AgamGr9k       ---------------------LFMAKFERYVMLIE-QINRCFSL--PIVTMLALGMVELA 271
AgamGr9m       ---------------------LFLERFRQYIAQIE-RINQCLSA--PLLGMLLQALIELA 274
AgamGr9n       ---------------------YFLHRYDRYSRLIG-EVDDCYSF--PVVLVLLLVMLQLI 242
AgamGr10       ---------------------YFLHRYDRYSRQIG-EVDDCYSF--PVVLVLLLVMLQLI 242
AgamGr11       ---------------------YFVERFELYASQLR-QIDQCFSM--PLTMIVLLVLIEMV 244
DmelGr23aA     ----------------------TLRTTYERIFECYETFSDCYGW--GMLGLHLLTSFQFV 244
DmelGr23aB     ----------------------DLKRRYNDLHYLFVRINGYFGG--SLLTIIIVHFAIFV 247
DmelGr39aA     -------------------VAGCLRTHDEILLLGQRELIAVYGG--VILFLFIYQVMQCI 250
DmelGr39aB     -----------------LRLRVCLEMHDRLMLLCNDEISLVYGF--IAWLSWMFASLDVT 257
DmelGr39aC     ----------------------NFCKYNQVIWLSYTEINHCFGL--LLLLVTGLILLITP 251
DmelGr39aD     ---------------------------QQILKLCGGELNECFGL--LMLPIVALVLLMAP 242
DmelGr98c      -----------------CVTLKQNQLLIGRIWRLVDEIGAYFRW--SMTLLFLYNGLTIL 274
DmelGr98d      -----------------CVALKRNQLLAGRIWGLVNEVSLYFTL--SLTLLFLYNELTIL 272
DmelGr98b      -----------------CVALKRNQLLLGRIWRLEGDVGSYFTP--TMLLLFLYNGLTIL 272
AgamGr51       -----------------PSVLQLYMLHCKNEDMVKQFM-ETLNF--PTLMLTGWYFFMIV 285
AgamGr52       -----------------STIREVYMVHCENAQLEKQFM-AIMDL--PVLLLNGWYFYMIV 277
DmelGr94a      -----------------YRMQRFCALADELDQLAYRYR-LIYVH--SGKYLTPMSLSMIL 265
DmelGr97a      -----------------YRMRRFCELADLLDELARKYG-FTASR--SKNYLRFTDWSMVL 286
DmelGr93b      -----------------NNPQPTRQAISNLDKCLYLYD-EIHQV--SRSFQQLFDLPLFL 276
DmelGr93c      -----------------GRKQ-LRIVEYRVDECISVYD-EIERV--GRTFHRLLELPVLI 269
DmelGr22d      -------------------VRFLLKLYNKLVNLYSKLA-DCYDC--QTVLMMAIFLAANI 264
DmelGr22e      -------------------MDLLLYLYHRLLDLGQRLA-SIYDY--QMVMVMVSFLIANV 267
DmelGr22a      -------------------IQLLLWLYSRLLDLNHRLT-AIYDI--QVTLFMATLFSVNI 266
DmelGr22b      -------------------IHQFLSLYKRLLELNRKLV-IAYEY--QMTLFIIAQLSGNI 265
DmelGr22c      -------------------IRKYLSLYRRLLELKGYMV-ATYEY--HMTLVLTTGLASNF 262
DmelGr22f      -------------------IHALASLYDRLLKLSELVV-ACNDL--QLILMLIIYLIGNT 258
DmelGr36a      ------------CCYLADRIDNIAKLQNQLQSIVTQLN-QVFGI--QGIMVYGGYYIFSV 265
DmelGr36c      ------------CCSLADQIENIARIQSQLQTIMNQME-EVFGI--QGAMTYGGYYLSSV 265
DmelGr36b      ------------CCYLSDQLEDIGEVQSQLQSMVGQLD-EVFGM--QGLMAYSEYYLSIV 265
DmelGr59d      ------------CCYLADRLERIAKSQSDLQELVENLS-TAYEG--EVVCLVITYYLNML 264
DmelGr59a      -------------------LRGLWALHRNLSYTARRIN-KHYGP--QMLAMRFDYFIFSI 251
DmelGr59b      -------------------LQHLWLLHACLTKTALNIN-KIYAP--QMLASRFDNFVNGV 252
AgamGr26       -------LPAGALYDYAHLIDLLRRTHLRLAQLLEQAN-GCFGV--LIVFTTTASFVVLS 293
AgamGr27       -----------KVRDCAELVETLRRTHLKLGDLTGRVV-GRFGP--LIVCTVLSSFVVLN 273
AgamGr29       -------TPVRLTRDDDRTIEKIRLLQLQLLQIVRRTNGGEYGR--LVIMILLATFIYLN 291
AgamGr31       -------TPVRFTRDDEQTIEKIRLLQLKLVQIVFHTNGGEYGL--LLIVVLLTTFIFLN 292
AgamGr30       -------RKSNTKENDITTIDKLVRLHTALLHLTWKTN-AHYGV--LLLIIMLYSFIQIS 285
AgamGr32a      -------KERVTSPLDVATIVRLSTMHMALMRLARAAN-KHFGV--LMLIIVLSTFIQIN 297
TcasGr2        --RRIAAVASEKIKNEI--IWKKLREDYTRQCRLVRKVDKEIAY--IVLLTFASDLLFIL 301
TcasGr9        --RRIAAVASEKVTSEK--SWIALNDDYNRLCHLCFLLDEKLSY--IILTSFLNNFYFII 301
TcasGr7        --DRTQSMSESKNKSES--AWKNLREDYNRLCRLCKRVDEEISY--IVLMSFASDLLFIL 303
TcasGr30       --DRTQSMSESKNKSES--AWKNLREDYNRLCRLCKRVDEEISY--IVLMSFASDLLFIL 303
TcasGr29       --DRTQSMSESKNKSES--AWKNLREDYNRLCRLCKRVDEEISY--IVLMSFASDLLFIL 303
AaegGr64f      --DRIRTFNGNQQPEE---VWRTLRLDFLRLSELVTYLDTKLSR--IILLSCASDMFFIS 260
AaegGr64a3     --DRIRTFNGNQQPEE---VWRTLRLDFLRLSELVTYLDTKLSR--IILLSCASDMFFIS 260
AgamGr14       --ERLERLDGQPQSQG---VWRALRLDYVRLSELVTFVDERFSK--LILFCCANDMFFIT 324
AgamGr18       --RRLVAVQGIACPTN---FWHDVRMDYVAVSELVQVLDAQFGH--LILLACANDMYFIA 296
AaegGr64d      --QRLVSVEGTHYPTT---FWREIRTDYVAVSQLVAFLDEQFGH--LILLSCANDMYFIA 328
AgamGr17       --RHAAQYKGRHVMGP---VWQRLRLDYGRISSLVAYMEGIMAP--IIVCTTASDLYFIF 285
```

```
AaegGr64c       --HRIEHLKGKMLPES---FWAEIRLDYTKISNLVIYMDGVLSP--MIMITSASNIFFIT 310
DmelGr64c       --WRIRQHVRQQMPNE---FWQRIRCDLLDLSDLLGIYDKELSG--LIVLSCAHNMYFVC 289
DmelGr64d       --WRIRQNLGKPMAKE---FWQEIRSDFLDLDSLLKLYDKELSG--LILVCCAHNMYFIC 298
AgamGr20        --KRID-SNLQNG---SEAFWGEMRTHFVGLIELVERTNRIVGP--LLIASCANDMYFLC 316
AaegGr61a       --IRIS-ERIQSRKGTTEDFWEQIRIQYVSLCDLVVLLNRSINR--LVFVSYANDLYFIC 313
AgamGr21        --NYLKILSDG-VLIPGEDFWIRVRTNYVAVCELLDDVDRAISW--TMLISCATNLYYIC 341
AaegGr64a       --THLRTLAGGGVLIPNEPFWIRVRTHYVSLCELLDEVDQAVAW--IVLISCATNLYFIC 224
DmelGr64a       --TRIRKLEHEEVCES---VFIQIREHYVKMCELLEFVDSAMSS--LILLSCVNNLYFVC 335
DmelGr61a       --ARVGALEGRHVPEA---LWYDIRRDHIRLCELASLVEASMSN--IVFVSCANNVYVIC 318
AgamGr15        --SFVQEQMLRQKMTEQ--FWGEQRQKYRNVCDLVTTVDDHISA--ITMLSISNNLFFIC 322
AaegGr1         --NHLMNHK-REKMTEQ--FWGEQRQNYRNICDLVRFVDDAISI--ITMLSISNNLFFIC 326
DmelGr5a        --RSLEQQV-RQPMPEA--YWTWSRTLYRSIVELIREVDDAVSG--IMLISFGSNLYFIC 322
DmelGr64f       --DDLRNFK-GMNMAPS--YWSERRIQYRNICILCDKMDDAISL--ITMVSFSNNLYFIC 347
DmelGr64e       --DYMMREA-RLPTTVD--YWMQCRINFRNLCKLCEEVDDAIST--ITLLCFSNNLYFIC 341
AmelGr1         --SRLYSIR-GKAMPEW--WWAEARSDYNHLATLTRQLDSHISI--MVLLSFATDLYFIC 270
AmelGr2         --KKLAVTM-TKCQAAF--NWRELREDYAILSCIVKKVDDHISP--IILLSFANNVYFIC 358
AgamGr16        --DVIASSE-KLYHRGI--FWKDQCTHYRRVLGLIRHVNNHIGV--FIVISYASNLFFIC 295
AaegGr64e       --EIILNSK-AQYHSDT--FWHDYRKHHQRVCNLVHIVGQNVAY--LVVISFANNMFFIC 313
TCasGr6         LFKDVDRNCSAYIIAHYRVLWLSLSDLLQKMGNAYARTYSTYSL--FMMANITVAVYGFT 268
TcasGr26        LFKDVDRNCSAYIIAHYRVLWLSLSDLLQKMGNAYARTYSTYSL--FMMANITVAVYGFT 268
TcasGr33        LFKDVDRNCSAYIIAHYRVLWLSLSDLLQKMGNAYARTYSTYSL--FMMANITVAVYGFT 268
AgamGr23        --QDVAIECTAAMISRYRFLWLNLSELLQALGNAYARTYSTYCL--FMFVNITVAIYGAL 352
AaegGr21a       --KDVAIECTAAMISQYRFLWLNLSELLQALGNAYARTYSTYCL--FMFANITIAIYGAL 344
TcasGr10        --KALESRNPAKILGAYRDLWVDLSHMMQQLGKAYSGMYSMYCL--LILLTTIVASYGSV 300
TcasGr39        --KALESRNPAKILGAYRDLWVDLSHMMQQLGKAYSGMYSMYCL--LILLTTIVASYGSV 300
AgamGr22        --DVLATERPAAKLTEYRHLWVDLSHMMQQLGKAYSNMYGIYCL--VIFFTTIIATYGSL 325
AaegGr21b       --NILATEQPADKLTEYRHLWVDLSHMMQQLGKAYSNMYGIYCL--VIFFTTIIATYGAL 325
DmelGr21a       --TTIRGEKPAQKLTEYRHLWVDLSHMMQQLGRAYSNMYGMYCL--VIFFTTIIATYGSI 326
AgamGr24        --RALRHVGPAAKVSEYRSLWLRLSKLARDTGFSTCYTFTFICL--YLFFIITLSIYGLM 251
AaegGr63a       --RALRHVGPAAMVSEYRSLWLRLSKLARETGSSTCYTFTFLCL--YLFFIITLSIYGLM 316
DmelGr63a       --KALKHIGPAAMVADYRVLWLRLSKLTRDTGNALCYTFVFMSL--YLFFIITLSIYGLM 345
TcasGr3         --MALKHVGPSSQVADYRSLWMLLSKLIRDVGNASGYTVTFLCL--YLFLIITLTIYGLL 307
DmelOr83b       --PNGLTKKQEMMVRSAIKYWVERHKHVVRLVAAIGDTYGAALL--LHMLTSTIKLTLLA 378
AgamOr7         --PNGLTRKQEMMVRSAIKYWVERHKHVVRLVSAIGDTYGPALL--LHMLTSTIKLTLLA 370
HvirOr2         --PNGLTPKQEMLARSAIKYWVERHKHVVRLVASIGDTYGTALL--FHMLVSTITLTLLA 364
TcasOr16        --PNGLTKKQELMVRSAIKYWVERHKHVVRLVTAIGDAYGVALL--LHMLTSTIMLTLLA 466
TcasOr22        --PNGLTKKQELMVRSAIKYWVERHKHVVRLVTAIGDAYGVALL--LHMLTSTIMLTLLA 505
AmelOr2         --PNGLTKKQEMLVRSAIKYWVERHKHIVRLVTAIGDAYGVALL--LHMLTTTITLTLLA 370
TcasGr31        -----------------TALWKQLRYEHYRLYQLSVLIDNNMSY--IIIVSFATNLYFII 178
TcasGr35        ---------------------VFIFDNVWLFILWVGILSTIFLC--DTILKKVDDILSQA 186
DmelGr59c       ------GGVYSIQCCSLADQLDLIAERHYFLKDRLDEMSDLFQI--QSLSMSLVYFFSTM 269
DmelGr92a       ----------------TSGSKQKIRRVQNRLEKCISLYREIYHT--SIMFHKLFVPLLFL 263
DmelGr28a       STPMKRYSVTSVIRLNPEYAIKQVSNIHNLLCDICQTIEEYFTY--PLLGIIAISFLFIL 306
DmelGr64b       -------IAAVHRKVMPAVFWTEVREHYLALKRLVHLLDAAIAP--LVLLAFGNNMSFIC 282
TcasGr71        -----------------------MEGLVCSIKELNMIFNEFFGW--PIIMIILYSSLMVL 228
AgamGr19        -----IQFYTTVQVVAREKFWSEIHSDYVMVCELLEHVMSICSP--LLVVSCGTNLYLIC 277
AaegGr64b       -----IEVACNGTVLPTESFWVEIREHYVIISEFLVHVDKMYSP--LVLSSCCNDVFLIC 226
DmelGr93d       --------------------VQKSMALFKEISSVVTSLQDIFNV--HLFLSALLTLLQVL 262
TcasGr34        ----------------------KIESLACELKNTVDDFNDIFGI--SFLLIISYSTLHFV 216
TcasGr43        ----------------HLFLVRRTETLAQILNQTVNFYNSAFGW--PIAFLIFFATLDFL 234
TcasGr47        --------------------YETVAHGMTVQKTTLFFFNRTFGV--PIFLVLVFVQMQSV 210
AmelGr3         VLGSYRLSRKLDENKMYVNNISELIMVHSSLCDAVSLINSTFGV--VILAVTVTCLLHLV 325
AgamGr32b       -----VLGESLKMESSRLDQLEQLRLLHVRTVQTAGSLSEKFGI--VIILIVIAAFASVN 296
AgamGr50        --------------SKAPNEIVRLYLMHTEASLVVQSIVEVISI--PILLLSVWYFFIIV 256
TcasGr20        ------PKVIQGKSALSYERLNEFMRLVEEIEDVIEAINGFYGV--HTLLILFSCLLHLV 283
TcasGr79        --------------------LKNCVIYFNRIFGWNILFGHIFTV--CRTLIYIDDNVKGL 238
TcasGr15        DIFGWTILQTVFFTVPRVLMYFDIFITEHESKPNWLFFLANFLT--NLLFWEFDQIVNVA 266
TcasGr60        --------------------IKLSNDKLEITACRLFKIDNSLIL--DLLVFITINFVVLL 229
AgamGr38        --DVADPPALTVYDPKQTLILRQIMQIFDKISDLADKGSYYYGI--QIMVSIAGSFVYLM 275
TcasGr51        YVLLNEKVEAIKTSPKMRHELQKLAQVYRILGETIETWNKLFGY--QILLITFQSGLNVV 649
DmelGr39b       -----CTLDGNANRLCSLEFLLALKQSHMQLHYLFTHFNDLFGW--SILGTYVVLFSDST 249
AgamGr28        -----------------AVRLQEIKHLYLQLDHCVQLMTRSYEV--AIVTNVFLAINVTS 256
TcasGr61        LYKNKYRKLIVDGKKSEEQIVDICIRSHDKLCDVCDSVNRIFGF--IIIIGCLIQFNTIV 550
TcasGr50        ---------------HFLALLDIVHGVHNELCNLCQVANSIYGF--QNFLFVLSTFSICI 248
AgamGr12        --------------VYTARQWCLIVDRYGAAVNLVNEINHIFSE--LLLVMLVQVQVQLC 251
AgamGr36        ------QKQRWGRRVDPVDVVSELAEDFAALVDVVDRVNRIYSN--QIIALITGVGMFSI 274
AgamGr48        -YWSRRRVRVQQICCDASDTIDRLCTLHQELTEIVRAIVSIFQL--PVLLMNLNQFIVIV 276
AgamGr49a       ----------AFLYRLINERVRTVIGSLLQLDDTDRQVRDSATG--RERRFICNHIYLQL 289
AgamGr49b       -----RQISTYEALKSVQRNHHLVSSNVKSFIHLQRSTDTVSNK--LIVISFRYNAPTQI 279
DmelGr2a        -----VLEEQEDLEEAAMDRLIAVRLVYQRVWALVALLNRCYGL--SMLMQVGNDFLAIT 282
```

```
DmelGr32a       ----VNPNVNPALMEHFPEDSLFIYRMHNKLLRIYKGINDCCNL--ILVSFLGYSFYTVT 331
DmelGr68a       -----------------WRELSNLIEVLCKFRYITENINCVAGV--SLLFYFGFSFYTVT 257
AmelGr7         FENNRFALYRNEHVRRNTNTIRAVKQIHLEIIKIVSFLNQTFGI--QILIQMTVSVVFTT 280
AmelGr8         ------------KNPILLMELRILKKQYTELIEAVQLLNHSYSL--KNEMILLMLFFDIT 275
AmelGr9         --------------SLLLIKLKHLEEKHLEISDVVQLVNDTFII--HIIVLVITTFSTIT 267
TcasGr57        --------AKTPPNKLNCYDLRRVRKNYNSICELVDAFNNIYGL--AMLEIIIVVITYVL 256
TcasGr41        ------------------DIVKEHQNLHNKICNLIDEFNECFGL--QILGILFVGVIYTT 246
AgamGr34        LHVIEVHGCGIDAANLKHLQLPKVLYRYNDLYDICKLLDRYFGL--LFLLTFTSIFIVTT 289
AgamGr35        ----AVGEELHSVGRREISDDLQVLAIVSRTIDLGQKIESYFGP--LFLTVYTALFSVTT 312
AgamGr39        -----SGSNKQLYPLSENETIKTIAMIYMKLGTAVQVYNRCFFI--HIFYIIASAFGLNL 294
AgamGr40        ------YKHSNSSVTEVGGIVRCIGDIHERLSETIVDFNYCFAL--QILLMMASAFGYTL 267
AaegGr28d       --------RIPRTSRQTCQVVQRIASLHDLLSDTVELFNECYYT--QVVHALTAAFNFTI 273
AgamGr42        CFVEKHSGEQAASYRVLMNHIQGFTAIYSNLCDAIGIFCTIFAW--QPMMFCASLIVTVV 295
AgamGr41        LRKSITSDRLEDDLGSVLQLIKTLMRLHDKLSDISTNCSQCFGFSVGTSVWVKSCFAYYT 299
AgamGr43        -------PFVQEHELISGERLTYMFVIHENVAKLAEFINGAYST--QIIAIVTVTFVNTL 290
DmelGr33a       -FDYDNATIAENTGNTSEANLPDLFKLHDKILALSVITNGEFGP--QCVPYMAACFVVSI 336
AgamGr44        --GLVDRARGYARLQHAVYVRSKLNYLADARCRIVACCTETHNLYQQMLLSCSFLCMTFI 276
AgamGr13        -----------SVQKILPYYSLSVALYLLILFQELMSKVHISGESWLQFLITYRLTFNFF 278
AgamGr45        -WLLQLLPHLGRGSEAAYESVRHLVRIHGQLLKAIGLLNCCFGVQNTILLLYQFVTLVEL 308
AgamGr46        EEQAHYERFRRDAKPPDRGETVSTTLELMLWLGQWATPAELLPTFWEVLSTLSWAFISTF 306
Dmelr93a        ERYRMTKYRRMQLLCDFADELDECAAIYSELYHVTNSFRRILQW--QILFYIYLNFINIC 290
AmelGr6         -------------------RQKTIQDLHNDLMLSSETLGSLYSWS--LLFWLGNLSIHSV 307
AmelGr10        --------------SIRHSTLQQIWWLHCSLANATEIINSVYAIQLLFWISSMSFNLMSR 250
TcasGr28        ----------------------------SVIMTLALGNPYCFNS--SVYKVFPVEHLLFI 200
TcasGr40        --------------------LTFSWYSKKVFYSKIIISGSIFMTTTSFYRILNSGYNLTT 261
AgamGr3         ------------------ERLEEYRALYGELWQINEGINELFGFS--QACNIASSFAQMA 261
AgamGr4         -----------AMYAQSVDRLLSLKSVYGQLWELSDCINRNFGWS--QICNFTGNFVQLS 297
AgamGr6         ---------RAISPEAIHRRLVHLKAMQNLLKDLTGEVSDRFGW--QLFAVITMLFICTT 291
AgamGr7         ---PVSIGATSQVDRVPLRRMFVVKGTYLTLWHASKDLNGCCVY--SQLANLLQNFIQCT 300
AgamGr47        -------------METILKTVNRLSVLHYHLCDAIRLVNRISSLS--LMFHFGAMFVFLV 277
DmelGr8a        --------SFPGFESFLRRRLVQKQRIYSHVYDMLKCFQGAFNFS--ILAVLLTINIRIA 263
AgamGr5         ---------SMRYMEFLAGKLNALKALYSDLFQASVALNEAYGR--TYLIIYCKNYIHIL 309
AgamGr8         --------------GQICGYLRKLMEGFARVERLLELLNQAFGY--SLAIIKLINNIYIL 249
DmelGr9a        -------------------DYKTLRLDYAHLAKVTRSLSHLFGLSLLLLNVLCLGDWIIV 222
DmelGr59f       ----------------RISEVQKIRMHHANLIDFTKAVNRTFQY--SILLLFVGCFLNFN 273
DmelGr77a       ELYKGQQKWLALELWRLLHVHHQLLKLHRSICSLCAVQAVCFLG--FVPLECTIHLFFTY 323
DmelGr85a       -----------------LWEIERLHTRFQCLIDLTSEVCSIFRY--VTLAYMARNLWSGI 274
TcasGr27        ----------------------QCEIVSDTLRNLDKIVTNGFRN-----VAESRKIFIEC 231
TcasGr48        --------------------LDLVLPIYSQLVFMCKKINKLYGH--QLLLTILTYLIWTI 248
AgamGr1         -------------------QLRSMIQLYRHILQIIESFCAVYGW--VLILIFFEHFLILT 262
DmelGr10a       ------LRPGGMLLHHCCELSDRLEELRRRCREIHDLQRESFRMHQFQLIGLMLSTLINN 282
DmelGr59e       -----AFAVLVDAGGGSALMIEEMRYTCNLIEQVHSQFLLRFGL--YLVLNLLNSLVSIC 264
DmelGr10b       -----------------YSDLMKQLRRRERLLRIGRRVHRMFAWLVAIALIYLVFFNTAT 250
DmelGr89a       -----------------------LLQKEYSWLRLIQRFVKLFQLG--IFLLVLGSFVNIM 238
DmelGr57a       ------------QLHVQELRIRQVVSMIQELHYLIQEINRVYALSLWAAMAHDLAMSTSE 271
DmelGr47b       ------------DSSEVHERIAYLFEMSKRCSFLLAELNGVFGFAAAAGIFYDFTIMTCF 269
DmelGr58a       ---HEERNSLTLSENKANRSLGKLAKLFKLFAENQRLVREVFRTFDLPIALLLLKMFVTN 266
DmelGr58b       ---------------KARKKWKALRSVAAMHLKTLRLARRIFDMFDIANATVFINMFMTA 265
DmelGr98a       -----------------LQKLAKLQAIHNSLWQAIRCLECYFQLS--LITLLMKKFFIDTS 252
AaegGr93a       ------AYKLTNSELKLSDHLDRLIIQYDKLYDVVVAIIEIYSP--SILFLLVLDFGQVT 276
TcasGr19        --------------------AFIIFTIHLLFYCVLIILLCIYYFYYAFILFTVHLLLLVC 257
DmelGr47a       ----------------RLKKMQHCLRLYSKLLLLCDQFNSQLGH--VAIWVLACKSWCQI 242
DmelGr58c       -------IPREMGLMQILAAWRKLWRRCRRLDALLKQFVDIFQWQVLFNLLTTYIFSIAV 276


TcasGr38        LALFRTAAELQASQIKWTRIAYMALASVPFIFDSIHLCDVCYSTIGTVS----------- 327
TcasGr46        LALFRTAAELQASQIKWTRIAYMALASVPFIFDSIHLCDVCYSTIGTVSKAGELIHQIQT 320
TcasGr1         QVIFVICVLVQSEKIVWLHLVYISFLGIICAADVFYICHVCYATIQEVR----------- 305
TcasGr123       ITIFFFTANVQAGELYFMSLLNPILSNVTFVIDVVYVCDVCYSTIEEAN----------- 313
TcasGr62        ITIFFFTANVQAGELYFMSLLNPILSNVTFVIDVVYVCDVCYSTIEEAN----------- 588
TcasGr25        INLFSLQLAHQKLEFSACGFFLINGTLLHTIVGAVTVYLIMFIQFDIATTTKG------- 207
TcasGr59        INLFSLQLAHQKLEFSACGFFLINGTLLHTIVGAVTVYLIMFIQFDIATTTKG------- 382
AgamGr2         AEFYFLYCSLTQQDVPIL--FRTAEVFLLGLAYIVYTALKCIVPIFVCWRTKT------- 336
AaegGr66a       AQFYFLYCALSKQEVPIL--FRSATVLQISIIQIFYIAVKCVICIYVCWKTKT------- 398
DmelGr66a       AQLYFLYCATQYQSIPSL--FRSAKNPFITVIVLSYTSGKCVYLIYLSWKTSQ------- 412
TcasGr11        FQAYYLYYVATGKAS----FVTVPMIVCPIVWLMDEVVEIYLLVYACASTCEQA------ 317
TcasGr55        FQAYYLYYVATGKAS----FVTVPMIVCPIVWLMDEVVEIYLLVYACASTCEQA------ 317
TcasGr21        TALFLVAINFNKSSSEENEGKTTQLDYFFTFWAFSNACEVMAIVWITSETCEEI------ 352
TcasGr56        TALFLVAINFNKSSSEENEGKTTQLDYFFTFWAFSNACEVMAIVWITSETCEEV------ 352
AaegGr          FDSYYILETIFTNDSTDTPFTKVQFVTFFFCQGFIHVVGVLNIVYYVNSLTIHEN------ 355
```

```
AaegGr28a       FDWYYILETIFTDDHTDSRFSRLQFVAFFLYQGLMHVIVVIKIVIFASLAVQE------- 250
AgamGr33        FNSYYVLDALLGSTSLDSPFSKGQFAMFFLCQAMVYGFGVFNIVYGSSSLVQEN------ 352
DmelGr28bB      FDAYYVLETLLGKSKRESKFKTVEFVTFFSCQMILYLIAIISIVEGSNRAIKKS------ 349
DmelGr28bC      FDAYYVLETLLGKSKRESKFKTVEFVTFFSCQMILYLIAIISIVEGSNRAIKKS------ 376
DmelGr28bD      FDAYYVLETLLGKSKRESKFKTVEFVTFFSCQMILYLIAIISIVEGSNRAIKKS------ 346
DmelGr28bA      FDAYYVLETLLGKSKRESKFKTVEFVTFFSCQMILYLIAIISIVEGSNRAIKKS------ 358
DmelGr28bE      FDAYYVLETLLGKSKRESKFKTVEFVTFFSCQMILYLIAIISIVEGSNRAIKKS------ 353
AgamGr25        ATSYFLMVELVGDKD-------AVFAWIQALWLFIHIFRFLLTIEPCHITNVES------ 377
AaegGr43a       ATSYFLMVELVGERD-------AVFSWIQALWLFIHIFRFLLTVEPCHITNVEV------ 317
DmelGr43a       ATAYFLFLELLSKRD-------NGYLWVQMLWICFHFLRLLMVVEPCHLAARES------ 355
AgamGr37b       NCSS----SSAVINEVQCCEGANMMLPVCNVPCAWIILIL-----YLSR----------- 314
AgamGr37d       NCSS----SSAVINEVQCCEGANMMLPVCNVPCAWIILIL-----YLSR----------- 310
AgamGr37a       NCSS----SSAVINEVQCCEGANMMLPVCNVPCAWIILIL-----YLSR----------- 324
AgamGr37c       NCSS----SSAVINEVQCCEGANMMLPVCNVPCAWIILIL-----YLSR----------- 318
AgamGr37e       NCSS----SSAVINEVQCCEGANMMLPVCNVPCAWIILIL-----YLSR----------- 318
AgamGr37f       NCSS----SSAVINEVQCCEGANMMLPVCNVPCAWIILIL-----YLSR----------- 345
AaegGr28c       VCSFGLFRAFIYRNELFYMGVLNFVWYMYYLFFVLFFIAVG---SKITREGKRI------ 490
AaegGr28b       FSVFVF--YRAMISQDYSQKLNAIVYLMSNWYVMLLILVMIGVFDGIKQKGKRT------ 339
AaegGr28e       FSVFVF--YRAMISQDYSQKLNAIVYLMSNWYVMLLILVMIGVFDGIKQKGKRT------ 339
TcasGr14        DTMYLFIHIRSRQEDTDTLVVIYTFNLFYLCFCFYWLLVMVAMFSRTQQSANKT------ 304
TcasGr49        DTMYLFIHIRSRQEDTDTLVVIYTFNLFYLCFCFYWLLVMVAMFSRTQQSANKT------ 348
AmelGr4         YIIYFFLAIFLKNINVDLILVIN--GILWIILLLCPFGLLTSKITKIVNEIEKT------ 333
AmelGr5         YNFYYLFLHFVNDK-LNFLLITN--AIIWIMLPLSLLALLTSKVTNVINEIEKT------ 332
TcasGr16        GDSYIVMYVFFFHLSDVRYVMVVYLLKNVVLYGLELFILAK-IVTDLCQEANST------ 320
TcasGr22        GDLHTSIYIIFFDMFYQHHKIVLDMGKNCVTYVFDLFYLSK-RASDLCNEANKT------ 223
TcasGr17        GDAYAVLNSVLFQNVYKPLPIVLSIVKNCLSYCFNLYHLAS-KSNQLSREAKVT------ 307
TcasGr150       GDAYAVLNSVLFQNVYKPLPIVLSIVKNCLSYCFNLYHLAS-KSNQLSREAKVT------ 307
TcasGr32        VYLH-----LAVMLGFQDIYMIVYIIVVIFWYTVNASTNIFLCDEICNEGQTILG----- 291
TcasGr54        VYLH-----LAVMLGFQDIYMIVYIIVVIFWYTVNASTNIFLCDEICNEGQTILG----- 291
TcasGr37        VYLQ-----HIFVKSRPSPATIISNVTVISWQAVCTFYNILLCDSVAHTAGELLG----- 265
TcasGr53        VYLQ-----HIFVKSRPSPATIISNVTVISWQAVCTFYNILLCDSVAHTAGELLG----- 265
TcasGr5         IYIQ-----VIVIGTKNTIETVAYSVIFILWHIVGTFSGIFLCDLIRREIANVQV----- 278
TcasGr12        DYVS-----CVVA----------------------LIILILQCGRVRSEVVKILN----- 248
TcasGr13        DYVS-----CVVA----------------------LIILILQCGRVRSEVVKILN----- 322
TcasGr104       TCLNFITNNLKSENEEFKEKLLAANLCLVIYTLVSVSLVMVTCDACTIASKQTIS----- 310
TcasGr45        TCLNFITNNLKSENEEFKEKLLAANLCLVIYTLVSVSLVMVTCDACTIASKQTIS----- 310
TcasGr98        VALNFFTSTLEIDNLYLKHKLEISSLVQTAITLVAMCGLVLICDSAKSESQQTVF----- 307
TcasGr52        SSLGFLLYASK-KQFTFEETFAIIHLSVFVYTLVSVILIIMGCDVVASNSRKTAE----- 244
TcasGr105       SSL-----YTCIAGYEFNANFTISYILFALFNTFGTILLIMTCSGVHSESQKLVG----- 290
TcasGr4         STFFYFFTTGN---------------IIATYDKQFYIHNALIVRALFLFFQLWATVYTSS 284
TcasGr44        STFFYFFTTGN---------------IIATYDKQFYIHNALIVRALFLFFQLWATVYTSS 288
AgamGr9a        ICILDTNRHIINIDG---------IMSIVANILWPLSDLKKLSAVFLLGEGVNRMVEETA 308
AgamGr9c        VCILDVNSYLARKNRP-------SMMVILANILWPLSDVKKLAAIFLLGEGVNGMVEETA 307
AgamGr9b        ICILDMYDLLLNNNS--------SGLLYLVYIIWPLSDIKKVMILFMLSEQVNRLVEETA 358
AgamGr9d        ICLLAIVNSMTNPMGQ-------SKFYIVVVVLWPLTDVKKLFAVILLSERTKQVVEETA 314
AgamGr9e        LKLYHVYDVFIRGVNEIVW--T-EHMLNVIECMWLCFDVKKLFIVMYISGMLQQKVEETA 310
AgamGr9f        LKLFHAYDVFKRSTYRTVW--T-EQLLSASEWLWLSFDVKKLFVVLYVSEVLKQKVEETA 307
AgamGr9g        VKMIDVGGALNDGTG--------DLTILILGCLWLLFDIKKLIVLLLISERLKQKVEETA 310
AgamGr9i        ISLVDLGGLLYDGSDL-------DMSLIAVGALWLLFDFKKFIVPLLLSECLKQKVEETA 311
AgamGr9h        SIWFSFLSASSSMQFQIVN--EESNLMLVSYILWFLSDSKNVLLVAISSSLLQQKVEETA 316
AgamGr9j        VAAYDVFENLRGES--KMILWDF--YRLLYRQVWEITYIVLMVLLAINCNATSLQVEETA 347
AgamGr9l        VAAYDVYDNYNLLD--VMSVWDK--FGLLFRQIWQMIYPIIICMIGITGNLTSIQVEETA 337
AgamGr9k        YLVFECFYTYDAGS--PDSEMYNGFTDWAFSQFWQSMYCHFLVLTVSSCERTRKMVEETA 329
AgamGr9m        YFMYEWFRVISTGQ--VVNGNYVSVQQWITSQFWQLMYGNVLLLLVPSCEQASNEVEETA 332
AgamGr9n        YLAEHLYSTIETGT--MMPVADN-FFYSLFGQVWQTMYGALGYYCISACGTASEEVEETA 299
AgamGr10        YLAEHLYSTIETGR--MMPVADN-FFYSLFSQVWQTVYGALGYYCISACGTASEEVEETA 299
AgamGr11        YLVFDIYAVLALDRPVFMENLEIDYVQWALRQLWQTIYGAVVLLTVTGCQRTCGELQQTA 304
DmelGr23aA      TNAYWMIMGIYDG-GNVRS-------LIFNGATGIDFGTPIATLFWHGDSGAENGRQIGC 296
DmelGr23aB      SNSYWLFVDIRTRPWRIYA-------ILLNLGFIFNVALQMAAACWHCQQSYNLGRQIGC 300
DmelGr39aA      L--IFYISNLEGFHSSND-------LVLIF--CWLAPMLFYLILPLVVNDIHNQANKTAK 299
DmelGr39aB      G--VIYLTMVIQTKKSIV-------LKLITNVVWLSPTFMTCAASFMSNRVTIQANKTAK 308
DmelGr39aC      SGPFYLVSTIFEGRFRQNWQFS---LMSFTAILWSLPWIVLLVLAMGRNDVQKEANKTAK 308
DmelGr39aD      SGPFFLISTVLEGKFRPD-ECL---IMLLTSSTWDTPWMIMLVLMLRTNGISEEANKTAK 298
DmelGr98c       HVVNWAIIRSIDPNDCCQLN------RLGSITFLSFNLLLTCFFSECCVKTYNSISYILH 328
DmelGr98d       QIVNWALIKSVNPNECCQYR------RVGTCLLLSINIFLSCLYSEFCIQTYNSISRVLH 326
DmelGr98b       HMVNWAYINKFLYDSCCQYE------RFLVCSTLLVNLLLPCLLSQRCINAYNCFPRILH 326
AgamGr51        YSVR----PPASPVGNGM-ADDFKAYINPLIFFLYQCVQLYLLVLIPSVYTDHAKKMMRL 340
AgamGr52        VSVYYMYTSTMVEVRRGFGVEDVTKYFNSVTFFLYLAVQLYYMMSIPSQYTERSKKMLSI 337
DmelGr94a       SLICHLLGITVGFYSLYYAIADTLIMGKPYDGLGSLINLVFLSISLAEITLLTHLCNHLL 325
DmelGr97a       SMLMNLLGITMGCYNQYLAIADHYINEEPFDLFLAIVLVVFLAVPFLELVMVARISNQTL 346
```

```
DmelGr93b      SLAQSLLAMSMVSY---HAILR-RQYS--FNLWG-LVIKLLIDVVLLTMSVHSAVNGSRL 329
DmelGr93c      ILLGKIFATTILSY---EVIIRPELYARKIGMWG-LVVKSFADVILLTLAVHEAVSSSRM 325
DmelGr22d      IVCFYMIVYRISLSKMSFFVMLIMFPLAIANNFMDFWLS-----MKVCDLLQKTGRQTSM 319
DmelGr22e      LGIYFFIIYSISLNKSLDFKILVFVQALVIN-MLDFWLN-----VEICELAERTGRQTST 321
DmelGr22a      IVGHVLVICWINITRFSLLVIFLLFPQALIINFWDLWQG-----IAFCDLAESTGKKTSM 321
DmelGr22b      VVIYFLIVYGLSMR--TYSIFLVAFPNSLLINIWDFWLC-----IAACDLTEKAGDETAI 318
DmelGr22c      LAIYSWIVLDISMN--INFIYLLIFPLFLLVNVWNLWLS-----IAASDLAENAGKSTQT 315
DmelGr22f      VQIFFLIVLGVSMN--KRYIYLVASP-QLIINFWDFWLN-----IVVCDLAGKCGDQTSK 310
DmelGr36a      ATTYITYSLAINGI--EELHLSVRAAALVFSWFLFYYTSAILNLFVMLKLFDDHKEMERI 323
DmelGr36c      GTCYLAYSILKHGY--ENLSMTLSTVILAYSWCFFYYLDGMLNLSVMLHVQDDYWEMLQI 323
DmelGr36b      GTSYMSYSIYKYGP--HNLKLSAKTSIIVCILITLFYLDALVNCNNMLRVLDHHKDFLGL 323
DmelGr59d      GTSYLLFSISKYGN--FGNNLLVIITLCGIVYFVFYVVDCWINAFNVFYLLDAHDKMVKL 322
DmelGr59a      INACIGTIYSTTDQ--EPSLEKIFGSLIYWVRSFDFFLN-----DYICDLVSEYQMQPKF 304
DmelGr59b      IQAYWGAVFTFDLS--TPFFWVVYGSVQYHVRCLDYYLI-----DNMCDVAVEYHDSAKH 305
AgamGr26       LQFFEIYRAT---TVRPWTVTDTYLLVYTVLWIVLHAAKVLMILYPAHLVQRERDRTGPI 350
AgamGr27       LELFNVYKATGRGSKRVWSASEGFRLVHTLLWIGLHGAKILLILYPGHRARVECERTGPT 333
AgamGr29       TELLQLYQGVKAGAFTFDVIGS------KVLNSAQKILMLFLFAYPNRMIQTQNIRGLKL 345
AgamGr31       TELLQLYQGIKASAFTFDVIGA------KLTNSALKFAMMVAYAIANRTIQTQNLRGLKL 346
AgamGr30       VILAELYFDNYD--LPLVFVGI------CLTHAAVYFMFFLIIAHANHAIIMENERTLLL 337
AgamGr32a      MLLLELYHNISHPVMPEYCLWV------LFLHAIVHFTFFFVIATSNHAIQQENERTMLL 351
TcasGr2        IQLFN-SLRRMRN-DLERLYFYWSFALLITRIVCLCLFGAKVHDESIKPLLTLNSVPTEI 359
TcasGr9        FQVFE-SFYLHKATTLETVYYFISLGLLILRLVSVCFYGSWINEESKLSLDLLSYVPREV 360
TcasGr7        IQLFN-SLRQMKN-NLERIYFYWSFGFLIVRTVCLCLFGGKVNDESTQPMLVLNSVSADV 361
TcasGr30       IQLFN-SLRQMKN-NLERIYFYWSFGFLII------------------------------ 331
TcasGr29       IQLFN-SLRQMKN-NLERIYFYWSFGFLIVRTVCLCLFGGKVNDESTQPMLVLNSVSADV 361
AaegGr64f      VQLYN--IFDPKQTTVTTFYYWYSLLFLICRCFVMLYVTSSIYEASLKPLELLRDFATSS 318
AaegGr64a3     VQLYN--IFE-------------------------------------------------- 268
AgamGr14       VQLFN--SFDLKPTTVTTVYFWYSLGFLIGRCFLMLFVVSSISRASEKPLETLRRFPSTN 382
AgamGr18       TQLFN--GFQRRRVIANYVYFWYSLLLLMFRTIVMLYVGSGVYAASTSPLQLLRNVPSQH 354
AaegGr64d      TQLFN--GFQRRPAFMTMVYFWYSLALLIFRTLCMLYIGSGVHVASMSPLNILRNVPSKY 386
AgamGr17       YQMYN--AFQFSASLISELYFKFSLAFLIFRTLVMLLIASNIHVASLRPLDILRSVPMSC 343
AaegGr64c      YQLYM--SVQLDASSMTTFYYRFSLLFLILRTLVMLLTSSRVYVASRKPLEILRAVPMSS 368
DmelGr64c      VQIYH--SFQSKGNYADELYFWFCLSYVIIRVLNMMFAASSIPQEAKEISYTLYEIPTEF 347
DmelGr64d      VQVYH--SFQVKGAFMDELYFWFCLLYVISRLMNMMLAASSIPQEIKDISNTLYEVRSSP 356
AgamGr20       LQTLN--ALEDKPYDINDWYFRYSFTFLILRTSVKLWFAADVDENSVRTHKLVQKIRSEH 374
AaegGr61a      LQIMH--ATLEQPFLINRVYFFYSFSFLLLRTFLMFWYSSQVQDASHQPCRLILRVPNHE 371
AgamGr21       LQILH--VSKKLANTVEDAYYGFSLGFLIVRTVIVFLSAAHIHDCAKKPLDIIMKIPNVG 399
AaegGr64a      LQILN--VSQKLRYPMNDVYYWFSLLFLMGRTATLFLCAAHIHEAAKRPLDIVAKIPNNG 282
DmelGr64a      YQLLN--VFNKLRWPINYIYFWYSLLYLIGRTAFVFLTAADINEESKRGLGVLRRVSSRS 393
DmelGr61a      NQALA--IFTKLRHPINYVYFWYSLIFLLARTSLVFMTASKIHDASLLPLRSLYLVPSDG 376
AgamGr15       VQILN--SMNSRPTLVHTVYFWFNLIILIGRTLAVAMFAAEVNDESKRPIEVLRTIPREG 380
AaegGr1        ASILN--SLNTHPTLVHTVYFWFGLAFLIGRTLAVSMCTAAVNDESQRPFEVLRAIPRDG 384
DmelGr5a       LQLLK--SINTMPSSAHAVYFYFSLLFLLSRSTAVLLFVSAINDQAREPLRLLRLVPLKG 380
DmelGr64f      VQLLR--SLNTMPSVAHAVYFYFSLIFLIGRTLAVSLYSSSVHDESRLTLRYLRCVPKES 405
DmelGr64e      GKILK--SMQAKPSIWHALYFWFSLVYLLGRTLILSLYSSSINDESKRPLVIFRLVPREY 399
AmelGr1        IQLL--FSFNPMRGIIEKIYFGFSFGFLLARTTVVSLCAATIHDESLLPAPILYSVSSSS 328
AmelGr2        LQLLNGLSISDKNSVLSEAYFFGSFAFLICRTCAVTLLTARIHDQSKQALPYLYNCSTSS 418
AgamGr16       VQLVN--NSS----FIVTSYFWYSLFHLIGRIVAVSLYGSAIHDEYCRTRTLFYNLPDEY 349
AaegGr64e      IQLIG--VLKPYPGIIVAIYVWYSLAHLMTRMVMVAVYAAAIHDESRRILPTFRTLPTQY 371
TCasGr6        SEIVDHGIRFSFKEIGLLVDSTYCLFLLFVFCDCSHQASLNIARRVQVTLLQVNLSQVDP 328
TcasGr26       SEIVDHGIRFSFKEIGLLVDSTYCLFLLFVFCDCSHQASLNIARRVQVTLLQVNLSQVDP 328
TcasGr33       SEIVDHGIRFSFKEIGLLVDSTYCLFLLFVFCDCSHQASLNIARRVQVTLLQVNLSQVDP 328
AgamGr23       SEIIDHGFGFSFKEIGLIVDTVYCSTLLFIFCDCSHNATLQVAQGVQDTLLSINLLKVDQ 412
AaegGr21a      SEVIDHGFGFSFKEIGLIVDTVYCSTLLFIFCDCSHNATLQVAQGVQDTLLGINLLKVDH 404
TcasGr10       TEIMDQGI--SFKEAGLFMIAFYCMTLLYIICNEGHHATRKMGPEFRERLLNVNLSAVDQ 358
TcasGr39       TEIMDQGI--SFKEAGLFMIAFYCMTLLYIICNEGHHATRKMGPEFRERLLNVNLSAVDQ 358
AgamGr22       SEIIEHGA--TYKEVGLFVIVFYCMSLLFIICNEAHHASK------RERLLNVNLTAVDK 377
AaegGr21b      SEIIEHGA--TYKEVGLFVIVFYCMGLLFIICNEAHHASRRVGLNFQERLLNVNLTAVDK 383
DmelGr21a      SEIIDHGA--TYKEVGLFVIVFYCMGLLYIICNEAHYASRKVGLDFQTKLLNINLTAVDA 384
AgamGr24       SQISDGFG---VKDIGLAVTAFCSVGLLFYICDEAHYASFNVRTNFQKKLLMVELSWMNT 308
AaegGr63a      SQISEGFG---IKDIGLAVTAFCSVGLLFFICDEAHYASFNVRTKFQKKLLMAELSWMNS 373
DmelGr63a      SQLSEGFG---IKDIGLTITALWNIGLLFYICDEAHYASVNVRTNFQKKLLMVELNWMNS 402
TcasGr3        SQLQAGFS---TKDIGLTINAGLAIFILYFICDEAHYASNCLRVQFQKKLLLVELSWMND 364
DmelOr83b      YQATKINGVNVYAFTVVG-YLGYALAQVFHFCIFGNRLIEESSSVMEAAYSCHWYDGSEE 437
AgamOr7        YQATKIDGVNVYGLTVIG-YLCYALAQVFLFCIFGNRLIEESSSVMKAAYSCHWYDGSEE 429
HvirOr2        YQATKINGINVYAFSTIG-YLSYTLGQVFHFCIFGNRLIEESSSVMEAAYSCQWYDGSEE 423
TcasOr16       YQATKITGVDKYAATVLG-YLLFALAQVFHFCIFGNRLIEESSSVMEAAYSCHWYDGSEE 525
TcasOr22       YQATKITGVDKYAATVLG-YLLFALAQVFHFCIFGNRLIEESSSVMEAAYSCHWYDGSEE 564
AmelOr2        YQATKIHAVDTYAASVVG-YLLYSLGQVFMLCIFGNRLIEESSSVMEAAYSCHWYDGSEE 429
TcasGr31       IQLFG--SMKIVKGTLKTAYYLISFALLIMRLISVCLCGASVHSESS------------- 223
```

```
TcasGr35        YKLEASFDDLATYET--------------------------------------------- 201
DmelGr59c       GSIYFSVCSILYSSTGFGSTYWGLLLIVLSTASFYMDNWLSVNIGFHIRDQQDELFRVLA 329
DmelGr92a       ALIYKVLLIALIGFNVAVEFYLNSFIFWILLGKHVLDLFLVTVSVEGAVNQFLNIGMQFG 323
DmelGr28a       FDDFYILEAILNPKRLDVFEADEFFAFFLMQLIWYIVIIVLIVEGSSRTILHSSYTAAIV 366
DmelGr64b       FQLFN-SFKNIGVDFLVMLAFWYSLGFAVVRTLLTIFVASSINDYERKIVTALRDVPSR- 340
TcasGr71        NYIDEVFNNNFGYDKKQYIGVVVANVGVAFMINVGTVTLILSCDAVLEEVKTTLLLAYKI 288
AgamGr19        YQLFH-LVDRTDDFIIVTVFTYFSLFFIILRTFLTMHYCSAVHEVARKPLKLFRRVPTSN 336
AaegGr64b       YLLLH--SLKPHAYAISFVYFWYTLCFLIGRTLTTLWMAAELNREKRSALRVVQRISSDG 284
DmelGr93d       VVWYKMIIDLGFSDFRIWSFSLKNLIQTLLPVLAIQEAANQFKQTRERALDIFLVGKSK- 321
TcasGr34        NYIDDLFFFRFEKSKFEPFLISNISLVSLIFLFNCTLIIMCDCVRSEASKVVKN------ 270
TcasGr43        DNVDTIMYEKTNVTFVHLAVLNILRVGKNFVCLLSLIILCDCVVKEAQKSLVLTYEMRWY 294
TcasGr47        QDLSDIFYYKDTAQLVSLALLMQLTQLVPALFVIYLCGRVMEEQNKIRLITFEMNKSFER 270
AmelGr3         ITPYFLILQAGERHEWIFLIVQGGWCIFHITRMLIIVQPSYSAIAEAKKTAVLVSQLLSC 385
AgamGr32b       IELLEFYQSIKLGTLTPTTIFMKFLYAASKFSFYILIAYPNRLIQQENQKALFMLYRIKR 356
AgamGr50        FSVSRCRWRVCALYGILTTVLYRSVQIFYTYTSLVQDLEAGSTNALRNIINPLAFFISEV 316
TcasGr20        VTPYYLLIEVCNAGYLPFILLQVAWIVLHLIRLMYQVTTNLVLKLLTSPLLP-------- 335
TcasGr79        GKQYNIASSKTLKLSINIISLVQLWIFQLYLLILCDQILREFDQIVVILSKVKSILN--- 295
TcasGr15        YRMETWNVVFNGSRKNLDVMKSFVNFRPSFTAARFFEIDRINAESRKPIRILFDVSSEIY 326
TcasGr60        GDLYVTLYIILTNNQAKYCTTLIGLIKNCVIVIFELYYLSHVCQNVSTEVIGNILRLMHR 289
AgamGr38        FYTFAFTLVIRSVLPAEIVFNAGPIISYSMFAYNVFIVAFYGNALKQQGKITGQLIDKAI 335
TcasGr51        SCINFPLGVLDRGNFDIEIALCNGIFLLLTLGSLLQMVISMDSTVEEAKTFVDRCYKMQE 709
DmelGr39b       VNIYWTQQVLVEVYEYKYLYATFSVFVPSFFNILVFCRCGEFCQRQSVLIGSYLRNLSCH 309
AgamGr28        LLLLEIYQYLQANELQANSIYIVSNVMWLALYICLLLMVLYPCDMIKREQMRFGTILFEL 316
TcasGr61        FAFCYCYYSISIRPISTLGWFFWSLLRIYELARKAAFAHLNSNEARATLNWVSKLIIRSN 610
TcasGr50        TQFYYCYDSGFNSDNLTDVYFTLQWAFLQILEILALAYLCDKVQLKIGEIKYS------- 301
AgamGr12        CQYFILFCSTVDGLVPIVTGLTVLHTQLWESLFLLMLLVVGYACDSCHNQIQDVNVAVRN 311
AgamGr36        FVIYATSFSYYVGRSRETRLTLILLTACVVYIIMIGLIFFTGVDVENTSNDIVGLLHEAI 334
AgamGr48        SRIYFVYILSAQVKHRSDYMSYHRFFNSILYICFEAVQCFLLALGSSVIAQEARRPGTTM 336
AgamGr49a       TILRTHHSALTKCIQNIVEFYNTPLALIMLYQFLIIISEAEKTGVLLNEFLPTDPNP--- 346
AgamGr49b       FYAYTSIVQDIKNDVDVPLGTYMSSILFAVFEAVQFYYIVSASSEITEEVRKHAEKTGVL 339
DmelGr2a        SNCYWMFLNFRQSAASPFDILQIVASGVWSAPHLGNVLVLSLLCDRTAQCASRLALCLHQ 342
DmelGr32a       TNCYNLFVQITGKGMVSPNILQWCFAWLCLHVSLLALLSRSCGLTTTEANATSQILARVY 391
DmelGr68a       NQSYLAFATLTAGSLSSKTEVADTIGLSCIWVLAETITMIVICSACDGLASEVNGTAQIL 317
AmelGr7         NLLHLLYRVIWLNFTLPELLQELISVIFWILIYGSQILYVNHVCASTNSEAVNIGNIICE 340
AmelGr8         FNLYIYLILYNTEVGKITKINSTLGFAIFYIVYIVISISMIEIIRVQMKKIGSNIHKILV 335
AmelGr9         FNLYFFLLKMYSPKTENIKFWFIPNLAPALFFFIKFAMIIWICESTTNEAKKIKWTLYDA 327
TcasGr57        NLTDLFLVYGMSKSRNIEGVSFGTNLVILCALWITTLLLFTILLAYGCAGATSEAEKIAK 316
TcasGr41        YTIFLLLIFGSGSATPENNVSVVYVLILYILESLLYMVTKLVVVTFLKFIKKIQIFGIMI 306
AgamGr34        IQLYYSYTILYWFTGENGFTIWSLMVCLNTISINLGVLLTIVLLCEKISNKTKHANDLLA 349
AgamGr35        IQSYYCYLHLTAKGDRGLSIETLVLSGGIILYNVIAIVALPYICEQVESESKLLMSYLSK 372
AgamGr39        VSCFTIIHVYLASSSYSIVDPIMISQLFLSLFFILIIMQVMFAGNLLHKKAKQTAILLHK 354
AgamGr40        FSIFGLIHAFSQVEEDESHQVSMNNMVYGCIYLSFIIQVVVAGSLVTKECKRTVIFVHKA 327
AaegGr28d       FSIFSVIHSNAGQTDEVTLRVTWVNAMFDGFFVVYTLQLIVAASIVNETCKRSSVMIHKA 333
AgamGr42        FSILTIGHMLTNSAPIVLALALIYSALTILYTLLFLVLVKLGSDLKREGKQTAVLVHKAI 355
AgamGr41        STLFQVFITMIHVLISQLITTFAFIRVMFYHYNETELNDSIIYTIGTTSYCCLPIITIWI 359
AgamGr43        FGFFIVTKLLFWNYQMLDLILRACRYAFECIPACLLVYYMLIVCASTHSELFKSASLLHK 350
DmelGr33a       FGIFLETKVNFIVGGKSRLLDYMTYLYVIWSFTTMMVAYIVLRLCCNANNHSKQSAMIVH 396
AgamGr44        VTQLLYLHYRTRSELSFAALGHETFTCLLCYFEVYCIAEACELVREQSLKTQKLLLRLNL 336
AgamGr13        YQFTHFYTLSLGNPSPHILEAMGTMMILVYGMYTAQLIAECYAISEKSQKIKSTVHRLSL 338
AgamGr45        GYNTCMMSVRYAEAKVQQGDSTEDFLETVYWVVWFVVEIFVLCYFSHDTVEEARRTVHLL 368
AgamGr46        RILRICNVCNSAKNEVIEEKHSTHKGSLVRCWCDFVSYCFQFLHTCMCVCLILSLFCDTP 366
Dmelr93a        LMLYQYILHFLNDDEVVFVSIVMAFVKLANLVLLMMCADYTVRQSEVPKKLPLDIVCSDM 350
AmelGr6         SNLYFIIDWVILTPWTNIAWPLIINMWCWLIGFITQLLALHIACDYTINEIFNRKIPNSR 367
AmelGr10        IYSLKVFKLSDYGKIRESMLVTDCAWNLVLITTVCHMTAHQVDLSLNQTEISPISRRTYG 310
TcasGr28        ANAFITSQSCENATLYSGDEMYFRVAFPSVFTLIDYSLWKACFVEVRR------------ 248
TcasGr40        FGSFIFNANSAIEGIIFFNLAKSWPQLIEKWSRVEMALDNWKNDKSLKRKFYLTICTIMS 321
AgamGr3         FDLYWVYAMWQKQQRGVELQIFCFVPTPVIIGFLMHAAKKHQLEMDAVQGTVLDMNFG-- 319
AgamGr4         CDLYWLYMSMKWFEATEYKVVIVITLLPSTSIIVLLLSSAESCLGVAASLQSALLDIPMG 357
AgamGr6         IDGYWMYASLHYDGNLYKVESFLCGISPVLMFFVLFNTCQKCVDEGEITFYYLHSVLNRV 351
AgamGr7         CDLYSLYALLYLNQLDDIFAYILSIVATFTALGVVLAACENCKNQVGQMGQLLYKRLHG- 359
AgamGr47        FGLFTIYNAFNSGTWAFRIMAIANGAWIAFYLAAILSVITATTAAQTSGRMTGDIVHQII 337
DmelGr8a        VDCYFMYYSIYNNVINNDYYLIVPALLEIPAFIYASQSCMVVVPRIAHQLHNIVTDSGCC 323
AgamGr5         SNSYWVVFWLLNNQRHHAAGIISRLICRIILLGATFYINSCAMKSAQNLRHRLHTMDLGI 369
AgamGr8         TDTYWIVHGFMSGKVFDSVDLECCLSSKFICLMINLHSNERILSEFHRTRVLLHCIHLRW 309
DmelGr9a        CNVYFMVAYLQVLPATLFLFGQVMFVVCPTLIKIWSICAASHRCVSKSKHLQQLKDLPG 282
DmelGr59f       LVLFLVYQGIENPSMADFTKWVCMLLWLAMHVGKVCSILHFNQSIQNEHSTCLTLLSRVS 333
DmelGr77a       FMKYSKFILRKYGRSFPLNYFAIAFLVGLFTNLLLVILPTYYSERRFNCTREIIKGGGLA 383
DmelGr85a       VAGYLLVRFVIGNGLQDVELVYLVFSFITCIQPLMLSLLVNSMTSTTGSLVEVTRDILKI 334
TcasGr27        IEQYNVILRYTKIVSDTYQGILVVQFFCSLVALCLTMYKLSLTEDLYFAIYEMHWYDAS- 290
TcasGr48        YEMYHLAILWSCTSTNCPRFLIMLALSYTVIQEVMLFTILWNCQNTRVASEDFKAIWYSI 308
```

```
AgamGr1         DRSFFAIRMYRMPMGFSIQQVLSMMMTWVFPLALNDMLLVGACTATEKALQQFEQQLCEV 322
DmelGr10a       LTNFYTLFHMLAKQSLEEVSYPVVVGSVYATGFYIDTYIVALINEHIKLELEAVALTMRR 342
DmelGr59e       VELYLIFNFFETPLWEESVLLVYRLLWLAMHGGRIWFILSVNEQILEQKCNLCQLLNELE 324
DmelGr10b       IYLGYTMFIQKHDALGLRGRGLKMLLTVVSFLVILWDVVLLQVICEKLLAEENKICDCPE 310
DmelGr89a       VNIYAFMSYYVSLHGVPLTISNNCLVLAIQLYAVILAAHLCQVRSAKLRKKCLQLEYVPE 298
DmelGr57a       LYILFGQSVGIGQQNEEENGSCYRMLGYLALVMIPPLYKLLIAPFYCDRTIYEARRCLRL 331
DmelGr47b       VYVICQKLLEREPWDPEYVYMLLHVAIHTYKVVITSTYGYLLLREKRNCMHLLSQYSRYF 329
DmelGr58a       VNLVYHGVQFGNDTIETSSYTRIVGQWVVISHYWSAVLLMNVVDDVTRRSDLKMGDLLRE 326
DmelGr58b       INILYHAVQYSNSSIKSNGWGILFGNGLIVFNFWGTMALMEMLDSVVTSCNNTGQQLRQL 325
DmelGr98a       ALPYWLYLSRVEHTRVAVQHYVATVECIKLLEIVVPCYLCTRCDAMQRKFLSMFYTVTTD 312
AaegGr93a       FELYMFYCDLSHASLDDDDLHMIAFSFVSAIFSFTEILFIINGTSKFSDKLMQALEQLQR 336
TcasGr19        IYYFYYMHLLFCCAFIIFTMHLLFLLCIYYFYCALIILLCIYYFCRAFIIFP-------- 309
DmelGr47a       TFGYEIFQMVAAPKSIDLTMSMRVFVIFTYIFDAMNLFLGTDISELFSTFRADSQRILRE 302
DmelGr58c       LFRLWIYLEFDKNFHLWKGILYAIIFLTHHVEIVMQFSIFEINRCKWLGLLEDVGNLWDI 336


TcasGr38        ------------------------------------------------------------
TcasGr46        EDHDIIDEIEMFSLQIANEQVEFNAAGFFPINYTLVFSVLKQYVMQQQVKNFRHSVRSVF 380
TcasGr1         ------------------------------------------------------------
TcasGr123       ------------------------------------------------------------
TcasGr62        ------------------------------------------------------------
TcasGr25        ------------------------------------------------------------
TcasGr59        ------------------------------------------------------------
AgamGr2         ------------------------------------------------------------
AaegGr66a       ------------------------------------------------------------
DmelGr66a       ------------------------------------------------------------
TcasGr11        ------------------------------------------------------------
TcasGr55        ------------------------------------------------------------
TcasGr21        ------------------------------------------------------------
TcasGr56        ------------------------------------------------------------
AaegGr          ------------------------------------------------------------
AaegGr28a       ------------------------------------------------------------
AgamGr33        ------------------------------------------------------------
DmelGr28bB      ------------------------------------------------------------
DmelGr28bC      ------------------------------------------------------------
DmelGr28bD      ------------------------------------------------------------
DmelGr28bA      ------------------------------------------------------------
DmelGr28bE      ------------------------------------------------------------
AgamGr25        ------------------------------------------------------------
AaegGr43a       ------------------------------------------------------------
DmelGr43a       ------------------------------------------------------------
AgamGr37b       ------------------------------------------------------------
AgamGr37d       ------------------------------------------------------------
AgamGr37a       ------------------------------------------------------------
AgamGr37c       ------------------------------------------------------------
AgamGr37e       ------------------------------------------------------------
AgamGr37f       ------------------------------------------------------------
AaegGr28c       ------------------------------------------------------------
AaegGr28b       ------------------------------------------------------------
AaegGr28e       ------------------------------------------------------------
TcasGr14        ------------------------------------------------------------
TcasGr49        ------------------------------------------------------------
AmelGr4         ------------------------------------------------------------
AmelGr5         ------------------------------------------------------------
TcasGr16        ------------------------------------------------------------
TcasGr22        ------------------------------------------------------------
TcasGr17        ------------------------------------------------------------
TcasGr150       ------------------------------------------------------------
TcasGr32        ------------------------------------------------------------
TcasGr54        ------------------------------------------------------------
TcasGr37        ------------------------------------------------------------
TcasGr53        ------------------------------------------------------------
TcasGr5         ------------------------------------------------------------
TcasGr12        ------------------------------------------------------------
TcasGr13        ------------------------------------------------------------
TcasGr104       ------------------------------------------------------------
TcasGr45        ------------------------------------------------------------
TcasGr98        ------------------------------------------------------------
TcasGr52        ------------------------------------------------------------
TcasGr105       ------------------------------------------------------------
```

```
TcasGr4         QVTR-------------------------------------------------------- 288
TcasGr44        QVTR-------------------------------------------------------- 292
AgamGr9a        LCTR-------------------------------------------------------- 312
AgamGr9c        LCTR-------------------------------------------------------- 311
AgamGr9b        LCTR-------------------------------------------------------- 362
AgamGr9d        LCTR-------------------------------------------------------- 318
AgamGr9e        LCTR-------------------------------------------------------- 314
AgamGr9f        LCTR-------------------------------------------------------- 311
AgamGr9g        LCTR-------------------------------------------------------- 314
AgamGr9i        LCTR-------------------------------------------------------- 315
AgamGr9h        LCTR-------------------------------------------------------- 320
AgamGr9j        LCTR-------------------------------------------------------- 351
AgamGr9l        LCTR-------------------------------------------------------- 341
AgamGr9k        LCTR-------------------------------------------------------- 333
AgamGr9m        LCTR-------------------------------------------------------- 336
AgamGr9n        LCTR-------------------------------------------------------- 303
AgamGr10        LCTR-------------------------------------------------------- 303
AgamGr11        QLVR-------------------------------------------------------- 308
DmelGr23aA      LISK-------------------------------------------------------- 300
DmelGr23aB      LISK-------------------------------------------------------- 304
DmelGr39aA      MLTK-------------------------------------------------------- 303
DmelGr39aB      MLTK-------------------------------------------------------- 312
DmelGr39aC      MLTK-------------------------------------------------------- 312
DmelGr39aD      MLTK-------------------------------------------------------- 302
DmelGr98c       QIGC-------------------------------------------------------- 332
DmelGr98d       QMYC-------------------------------------------------------- 330
DmelGr98b       KIRC-------------------------------------------------------- 330
AgamGr51        LNY--------------------------------------------------------- 343
AgamGr52        LG---------------------------------------------------------- 339
DmelGr94a       VATRRSAVIL-------------------------------------------------- 335
DmelGr97a       VETRRTGELL-------------------------------------------------- 356
DmelGr93b       IRR----LSF-------------------------------------------------- 335
DmelGr93c       MRR----LSL-------------------------------------------------- 331
DmelGr22d       ILKL-------------------------------------------------------- 323
DmelGr22e       ILKL-------------------------------------------------------- 325
DmelGr22a       ILKL-------------------------------------------------------- 325
DmelGr22b       ILKI-------------------------------------------------------- 322
DmelGr22c       VLKL-------------------------------------------------------- 319
DmelGr22f       VLKL-------------------------------------------------------- 314
DmelGr36a       LEER-------------------------------------------------------- 327
DmelGr36c       LGKR-------------------------------------------------------- 327
DmelGr36b       LEER-------------------------------------------------------- 327
DmelGr59d       LNKR-------------------------------------------------------- 326
DmelGr59a       FAP--------------------------------------------------------- 307
DmelGr59b       SWS--------------------------------------------------------- 308
AgamGr26        LCHFNP------------------------------------------------------ 356
AgamGr27        LYEIASR----------------------------------------------------- 340
AgamGr29        LYQLNQP----------------------------------------------------- 352
AgamGr31        LFQLHSS----------------------------------------------------- 353
AgamGr30        LLDINCI----------------------------------------------------- 344
AgamGr32a       LHEFKCS----------------------------------------------------- 358
TcasGr2         ------------------------------------------------------------
TcasGr9         ------------------------------------------------------------
TcasGr7         ------------------------------------------------------------
TcasGr30        ------------------------------------------------------------
TcasGr29        ------------------------------------------------------------
AaegGr64f       ------------------------------------------------------------
AaegGr64a3      ------------------------------------------------------------
AgamGr14        ------------------------------------------------------------
AgamGr18        ------------------------------------------------------------
AaegGr64d       ------------------------------------------------------------
AgamGr17        ------------------------------------------------------------
AaegGr64c       ------------------------------------------------------------
DmelGr64c       ------------------------------------------------------------
DmelGr64d       ------------------------------------------------------------
AgamGr20        ------------------------------------------------------------
AaegGr61a       ------------------------------------------------------------
AgamGr21        ------------------------------------------------------------
AaegGr64a       ------------------------------------------------------------
DmelGr64a       ------------------------------------------------------------
```

```
DmelGr61a       ------------------------------------------------------------
AgamGr15        ------------------------------------------------------------
AaegGr1         ------------------------------------------------------------
DmelGr5a        ------------------------------------------------------------
DmelGr64f       ------------------------------------------------------------
DmelGr64e       ------------------------------------------------------------
AmelGr1         ------------------------------------------------------------
AmelGr2         ------------------------------------------------------------
AgamGr16        G----------------------------------------------------------- 350
AaegGr64e       ------------------------------------------------------------
TCasGr6         A----------------------------------------------------------- 329
TcasGr26        A----------------------------------------------------------- 329
TcasGr33        A----------------------------------------------------------- 329
AgamGr23        P----------------------------------------------------------- 413
AaegGr21a       P----------------------------------------------------------- 405
TcasGr10        K----------------------------------------------------------- 359
TcasGr39        K----------------------------------------------------------- 359
AgamGr22        A----------------------------------------------------------- 378
AaegGr21b       A----------------------------------------------------------- 384
DmelGr21a       A----------------------------------------------------------- 385
AgamGr24        D----------------------------------------------------------- 309
AaegGr63a       D----------------------------------------------------------- 374
DmelGr63a       D----------------------------------------------------------- 403
TcasGr3         E----------------------------------------------------------- 365
DmelOr83b       ------------------------------------------------------------
AgamOr7         ------------------------------------------------------------
HvirOr2         ------------------------------------------------------------
TcasOr16        ------------------------------------------------------------
TcasOr22        ------------------------------------------------------------
AmelOr2         ------------------------------------------------------------
TcasGr31        ------------------------------------------------------------
TcasGr35        ------------------------------------------------------------
DmelGr59c       DR---------------------------------------------------------- 331
DmelGr92a       N----------------------------------------------------------- 324
DmelGr28a       HKILN------------------------------------------------------- 371
DmelGr64b       ------------------------------------------------------------
TcasGr71        RQVFP------------------------------------------------------- 293
AgamGr19        ------------------------------------------------------------
AaegGr64b       ------------------------------------------------------------
DmelGr93d       ------------------------------------------------------------
TcasGr34        ------------------------------------------------------------
TcasGr43        CQTAT------------------------------------------------------- 299
TcasGr47        ------------------------------------------------------------
AmelGr3         TFEAN------------------------------------------------------- 390
AgamGr32b       ISCS-------------------------------------------------------- 360
AgamGr50        GQVYFMVSSSATFSR--------------------------------------------- 331
TcasGr20        ------------------------------------------------------------
TcasGr79        ------------------------------------------------------------
TcasGr15        N----------------------------------------------------------- 327
TcasGr60        ------------------------------------------------------------
AgamGr38        NSTNN------------------------------------------------------- 340
TcasGr51        QFTLN------------------------------------------------------- 714
DmelGr39b       PSIG-------------------------------------------------------- 313
AgamGr28        SHSG-------------------------------------------------------- 320
TcasGr61        P----------------------------------------------------------- 611
TcasGr50        ------------------------------------------------------------
AgamGr12        NVGS-------------------------------------------------------- 315
AgamGr36        QR---------------------------------------------------------- 336
AgamGr48        NVFVN------------------------------------------------------- 341
AgamGr49a       ------------------------------------------------------------
AgamGr49b       LN---------------------------------------------------------- 341
DmelGr2a        VSVDLR------------------------------------------------------ 348
DmelGr32a       AKSK-------------------------------------------------------- 395
DmelGr68a       ARIYG------------------------------------------------------- 322
AmelGr7         FYEP-------------------------------------------------------- 344
AmelGr8         HTFD-------------------------------------------------------- 339
AmelGr9         FSDS-------------------------------------------------------- 331
TcasGr57        ICFYWLN----------------------------------------------------- 323
TcasGr41        TKACMSASNLIQRTPN-------------------------------------------- 322
AgamGr34        DLQLR------------------------------------------------------- 354
```

```
AgamGr35        LS---------------------------------------------------------- 374
AgamGr39        ------------------------------------------------------------
AgamGr40        VCYG-------------------------------------------------------- 331
AaegGr28d       AAYG-------------------------------------------------------- 337
AgamGr42        NQSS-------------------------------------------------------- 359
AgamGr41        AGHIKKR----------------------------------------------------- 366
AgamGr43        LLQYKSN----------------------------------------------------- 357
DmelGr33a       EIMQ-------------------------------------------------------- 400
AgamGr44        SP---------------------------------------------------------- 338
AgamGr13        IPGQ-------------------------------------------------------- 342
AgamGr45        RMPN-------------------------------------------------------- 372
AgamGr46        ------------------------------------------------------------
Dmelr93a        DER--------------------------------------------------------- 353
AmelGr6         LILWAP------------------------------------------------------ 373
AmelGr10        DIT--------------------------------------------------------- 313
TcasGr28        ------------------------------------------------------------
TcasGr40        ------------------------------------------------------------
AgamGr3         ------------------------------------------------------------
AgamGr4         N----------------------------------------------------------- 358
AgamGr6         LP---------------------------------------------------------- 353
AgamGr7         ------------------------------------------------------------
AgamGr47        RKHQN------------------------------------------------------- 342
DmelGr8a        SCP--------------------------------------------------------- 326
AgamGr5         QIKC-------------------------------------------------------- 373
AgamGr8         QLR--------------------------------------------------------- 312
DmelGr9a        ------------------------------------------------------------
DmelGr59f       YAR--------------------------------------------------------- 336
DmelGr77a       FPSR-------------------------------------------------------- 387
DmelGr85a       SHKKS------------------------------------------------------- 339
TcasGr27        ------------------------------------------------------------
TcasGr48        LIKK-------------------------------------------------------- 312
AgamGr1         GKMAN------------------------------------------------------- 327
DmelGr10a       FAEP-------------------------------------------------------- 346
DmelGr59e       VCSS-------------------------------------------------------- 328
DmelGr10b       DVAS-------------------------------------------------------- 314
DmelGr89a       G----------------------------------------------------------- 299
DmelGr57a       VEKLD------------------------------------------------------- 336
DmelGr47b       SGQD-------------------------------------------------------- 333
DmelGr58a       FS---------------------------------------------------------- 328
DmelGr58b       SDLP-------------------------------------------------------- 329
DmelGr98a       RRSS-------------------------------------------------------- 316
AaegGr93a       IG---------------------------------------------------------- 338
TcasGr19        ------------------------------------------------------------
DmelGr47a       TS---------------------------------------------------------- 304
DmelGr58c       NYSGRQ------------------------------------------------------ 342


TcasGr38        ------------------------------------------WCELNFDWSS-------- 337
TcasGr46        FLSEIFGLVNLKYRETYFRLSKTKTFCTLVTALVYCSLAIFVLCELLIEGTTSILINVPS 440
TcasGr1         -------------------------------------------AKIYFFYLVK------- 315
TcasGr123       ------------------------------------------------------------
TcasGr62        ------------------------------------------------------------
TcasGr25        ------------------------------------------------------------
TcasGr59        ------------------------------------------------------------
AgamGr2         ------------------------------------------------------------
AaegGr66a       ------------------------------------------------------------
DmelGr66a       ------------------------------------------------------------
TcasGr11        ------------------------------------------------------------
TcasGr55        ------------------------------------------------------------
TcasGr21        ------------------------------------------------------------
TcasGr56        ------------------------------------------------------------
AaegGr          ------------------------------------------------------------
AaegGr28a       ------------------------------------------------------------
AgamGr33        ------------------------------------------------------------
DmelGr28bB      ------------------------------------------------------------
DmelGr28bC      ------------------------------------------------------------
DmelGr28bD      ------------------------------------------------------------
DmelGr28bA      ------------------------------------------------------------
DmelGr28bE      ------------------------------------------------------------
AgamGr25        ------------------------------------------------------------
```

```
AaegGr43a       ------------------------------------------------------------
DmelGr43a       ------------------------------------------------------------
AgamGr37b       ------------------------------------------------------------
AgamGr37d       ------------------------------------------------------------
AgamGr37a       ------------------------------------------------------------
AgamGr37c       ------------------------------------------------------------
AgamGr37e       ------------------------------------------------------------
AgamGr37f       ------------------------------------------------------------
AaegGr28c       ------------------------------------------------------------
AaegGr28b       ------------------------------------------------------------
AaegGr28e       ------------------------------------------------------------
TcasGr14        ------------------------------------------------------------
TcasGr49        ------------------------------------------------------------
AmelGr4         ------------------------------------------------------------
AmelGr5         ------------------------------------------------------------
TcasGr16        ------------------------------------------------------------
TcasGr22        ------------------------------------------------------------
TcasGr17        ------------------------------------------------------------
TcasGr150       ------------------------------------------------------------
TcasGr32        ------------------------------------------------------------
TcasGr54        ------------------------------------------------------------
TcasGr37        ------------------------------------------------------------
TcasGr53        ------------------------------------------------------------
TcasGr5         ------------------------------------------------------------
TcasGr12        ------------------------------------------------------------
TcasGr13        ------------------------------------------------------------
TcasGr104       ------------------------------------------------------------
TcasGr45        ------------------------------------------------------------
TcasGr98        ------------------------------------------------------------
TcasGr52        ------------------------------------------------------------
TcasGr105       ------------------------------------------------------------
TcasGr4         ------------------------------------------------------------
TcasGr44        ------------------------------------------------------------
AgamGr9a        ------------------------------------------------------------
AgamGr9c        ------------------------------------------------------------
AgamGr9b        ------------------------------------------------------------
AgamGr9d        ------------------------------------------------------------
AgamGr9e        ------------------------------------------------------------
AgamGr9f        ------------------------------------------------------------
AgamGr9g        ------------------------------------------------------------
AgamGr9i        ------------------------------------------------------------
AgamGr9h        ------------------------------------------------------------
AgamGr9j        ------------------------------------------------------------
AgamGr9l        ------------------------------------------------------------
AgamGr9k        ------------------------------------------------------------
AgamGr9m        ------------------------------------------------------------
AgamGr9n        ------------------------------------------------------------
AgamGr10        ------------------------------------------------------------
AgamGr11        ------------------------------------------------------------
DmelGr23aA      ------------------------------------------------------------
DmelGr23aB      ------------------------------------------------------------
DmelGr39aA      ------------------------------------------------------------
DmelGr39aB      ------------------------------------------------------------
DmelGr39aC      ------------------------------------------------------------
DmelGr39aD      ------------------------------------------------------------
DmelGr98c       ------------------------------------------------------------
DmelGr98d       ------------------------------------------------------------
DmelGr98b       ------------------------------------------------------------
AgamGr51        ------------------------------------------------------------
AgamGr52        ------------------------------------------------------------
DmelGr94a       ------------------------------------------------------------
DmelGr97a       ------------------------------------------------------------
DmelGr93b       ------------------------------------------------------------
DmelGr93c       ------------------------------------------------------------
DmelGr22d       ------------------------------------------------------------
DmelGr22e       ------------------------------------------------------------
DmelGr22a       ------------------------------------------------------------
DmelGr22b       ------------------------------------------------------------
DmelGr22c       ------------------------------------------------------------
DmelGr22f       ------------------------------------------------------------
```

```
DmelGr36a       ------------------------------------------------------------
DmelGr36c       ------------------------------------------------------------
DmelGr36b       ------------------------------------------------------------
DmelGr59d       ------------------------------------------------------------
DmelGr59a       ------------------------------------------------------------
DmelGr59b       ------------------------------------------------------------
AgamGr26        ------------------------------------------------------------
AgamGr27        ------------------------------------------------------------
AgamGr29        ------------------------------------------------------------
AgamGr31        ------------------------------------------------------------
AgamGr30        ------------------------------------------------------------
AgamGr32a       ------------------------------------------------------------
TcasGr2         ------------------------------------------------------------
TcasGr9         ------------------------------------------------------------
TcasGr7         ------------------------------------------------------------
TcasGr30        ------------------------------------------------------------
TcasGr29        ------------------------------------------------------------
AaegGr64f       ------------------------------------------------------------
AaegGr64a3      ------------------------------------------------------------
AgamGr14        ------------------------------------------------------------
AgamGr18        ------------------------------------------------------------
AaegGr64d       ------------------------------------------------------------
AgamGr17        ------------------------------------------------------------
AaegGr64c       ------------------------------------------------------------
DmelGr64c       ------------------------------------------------------------
DmelGr64d       ------------------------------------------------------------
AgamGr20        ------------------------------------------------------------
AaegGr61a       ------------------------------------------------------------
AgamGr21        ------------------------------------------------------------
AaegGr64a       ------------------------------------------------------------
DmelGr64a       ------------------------------------------------------------
DmelGr61a       ------------------------------------------------------------
AgamGr15        ------------------------------------------------------------
AaegGr1         ------------------------------------------------------------
DmelGr5a        ------------------------------------------------------------
DmelGr64f       ------------------------------------------------------------
DmelGr64e       ------------------------------------------------------------
AmelGr1         ------------------------------------------------------------
AmelGr2         ------------------------------------------------------------
AgamGr16        ------------------------------------------------------------
AaegGr64e       ------------------------------------------------------------
TCasGr6         ------------------------------------------------------------
TcasGr26        ------------------------------------------------------------
TcasGr33        ------------------------------------------------------------
AgamGr23        ------------------------------------------------------------
AaegGr21a       ------------------------------------------------------------
TcasGr10        ------------------------------------------------------------
TcasGr39        ------------------------------------------------------------
AgamGr22        ------------------------------------------------------------
AaegGr21b       ------------------------------------------------------------
DmelGr21a       ------------------------------------------------------------
AgamGr24        ------------------------------------------------------------
AaegGr63a       ------------------------------------------------------------
DmelGr63a       ------------------------------------------------------------
TcasGr3         ------------------------------------------------------------
DmelOr83b       ------------------------------------------------------------
AgamOr7         ------------------------------------------------------------
HvirOr2         ------------------------------------------------------------
TcasOr16        ------------------------------------------------------------
TcasOr22        ------------------------------------------------------------
AmelOr2         ------------------------------------------------------------
TcasGr31        ------------------------------------------------------------
TcasGr35        ------------------------------------------------------------
DmelGr59c       ------------------------------------------------------------
DmelGr92a       ------------------------------------------------------------
DmelGr28a       ------------------------------------------------------------
DmelGr64b       ------------------------------------------------------------
TcasGr71        ------------------------------------------------------------
AgamGr19        ------------------------------------------------------------
AaegGr64b       ------------------------------------------------------------
```

```
DmelGr93d         ------------------------------------------------------------
TcasGr34          ------------------------------------------------------------
TcasGr43          ------------------------------------------------------------
TcasGr47          ------------------------------------------------------------
AmelGr3           ------------------------------------------------------------
AgamGr32b         ------------------------------------------------------------
AgamGr50          ------------------------------------------------------------
TcasGr20          ------------------------------------------------------------
TcasGr79          ------------------------------------------------------------
TcasGr15          ------------------------------------------------------------
TcasGr60          ------------------------------------------------------------
AgamGr38          ------------------------------------------------------------
TcasGr51          ------------------------------------------------------------
DmelGr39b         ------------------------------------------------------------
AgamGr28          ------------------------------------------------------------
TcasGr61          ------------------------------------------------------------
TcasGr50          ------------------------------------------------------------
AgamGr12          ------------------------------------------------------------
AgamGr36          ------------------------------------------------------------
AgamGr48          ------------------------------------------------------------
AgamGr49a         ------------------------------------------------------------
AgamGr49b         ------------------------------------------------------------
DmelGr2a          ------------------------------------------------------------
DmelGr32a         ------------------------------------------------------------
DmelGr68a         ------------------------------------------------------------
AmelGr7           ------------------------------------------------------------
AmelGr8           ------------------------------------------------------------
AmelGr9           ------------------------------------------------------------
TcasGr57          ------------------------------------------------------------
TcasGr41          ------------------------------------------------------------
AgamGr34          ------------------------------------------------------------
AgamGr35          ------------------------------------------------------------
AgamGr39          ------------------------------------------------------------
AgamGr40          ------------------------------------------------------------
AaegGr28d         ------------------------------------------------------------
AgamGr42          ------------------------------------------------------------
AgamGr41          ------------------------------------------------------------
AgamGr43          ------------------------------------------------------------
DmelGr33a         ------------------------------------------------------------
AgamGr44          ------------------------------------------------------------
AgamGr13          ------------------------------------------------------------
AgamGr45          ------------------------------------------------------------
AgamGr46          ------------------------------------------------------------
Dmelr93a          ------------------------------------------------------------
AmelGr6           ------------------------------------------------------------
AmelGr10          ------------------------------------------------------------
TcasGr28          ------------------------------------------------------------
TcasGr40          ------------------------------------------------------------
AgamGr3           ------------------------------------------------------------
AgamGr4           ------------------------------------------------------------
AgamGr6           ------------------------------------------------------------
AgamGr7           ------------------------------------------------------------
AgamGr47          ------------------------------------------------------------
DmelGr8a          ------------------------------------------------------------
AgamGr5           ------------------------------------------------------------
AgamGr8           ------------------------------------------------------------
DmelGr9a          ------------------------------------------------------------
DmelGr59f         ------------------------------------------------------------
DmelGr77a         ------------------------------------------------------------
DmelGr85a         ------------------------------------------------------------
TcasGr27          ------------------------------------------------------------
TcasGr48          ------------------------------------------------------------
AgamGr1           ------------------------------------------------------------
DmelGr10a         ------------------------------------------------------------
DmelGr59e         ------------------------------------------------------------
DmelGr10b         ------------------------------------------------------------
DmelGr89a         ------------------------------------------------------------
DmelGr57a         ------------------------------------------------------------
DmelGr47b         ------------------------------------------------------------
DmelGr58a         ------------------------------------------------------------
```

```
DmelGr58b       ------------------------------------------------------------
DmelGr98a       ------------------------------------------------------------
AaegGr93a       ------------------------------------------------------------
TcasGr19        ------------------------------------------------------------
DmelGr47a       ------------------------------------------------------------
DmelGr58c       ------------------------------------------------------------


TcasGr38        ------------------------------------------------------------
TcasGr46        LIIHVSTSAYVATVWINSVINRWKFIEFIRKVLEFDVKCVSNYTKQQSKIHLIVRSVFVT 500
TcasGr1         ------------------------------------------------------------
TcasGr123       ------------------------------------------------------------
TcasGr62        ------------------------------------------------------------
TcasGr25        ------------------------------------------------------------
TcasGr59        ------------------------------------------------------------
AgamGr2         ------------------------------------------------------------
AaegGr66a       ------------------------------------------------------------
DmelGr66a       ------------------------------------------------------------
TcasGr11        ------------------------------------------------------------
TcasGr55        ------------------------------------------------------------
TcasGr21        ------------------------------------------------------------
TcasGr56        ------------------------------------------------------------
AaegGr          ------------------------------------------------------------
AaegGr28a       ------------------------------------------------------------
AgamGr33        ------------------------------------------------------------
DmelGr28bB      ------------------------------------------------------------
DmelGr28bC      ------------------------------------------------------------
DmelGr28bD      ------------------------------------------------------------
DmelGr28bA      ------------------------------------------------------------
DmelGr28bE      ------------------------------------------------------------
AgamGr25        ------------------------------------------------------------
AaegGr43a       ------------------------------------------------------------
DmelGr43a       ------------------------------------------------------------
AgamGr37b       ------------------------------------------------------------
AgamGr37d       ------------------------------------------------------------
AgamGr37a       ------------------------------------------------------------
AgamGr37c       ------------------------------------------------------------
AgamGr37e       ------------------------------------------------------------
AgamGr37f       ------------------------------------------------------------
AaegGr28c       ------------------------------------------------------------
AaegGr28b       ------------------------------------------------------------
AaegGr28e       ------------------------------------------------------------
TcasGr14        ------------------------------------------------------------
TcasGr49        ------------------------------------------------------------
AmelGr4         ------------------------------------------------------------
AmelGr5         ------------------------------------------------------------
TcasGr16        ------------------------------------------------------------
TcasGr22        ------------------------------------------------------------
TcasGr17        ------------------------------------------------------------
TcasGr150       ------------------------------------------------------------
TcasGr32        ------------------------------------------------------------
TcasGr54        ------------------------------------------------------------
TcasGr37        ------------------------------------------------------------
TcasGr53        ------------------------------------------------------------
TcasGr5         ------------------------------------------------------------
TcasGr12        ------------------------------------------------------------
TcasGr13        ------------------------------------------------------------
TcasGr104       ------------------------------------------------------------
TcasGr45        ------------------------------------------------------------
TcasGr98        ------------------------------------------------------------
TcasGr52        ------------------------------------------------------------
TcasGr105       ------------------------------------------------------------
TcasGr4         ------------------------------------------------------------
TcasGr44        ------------------------------------------------------------
AgamGr9a        ------------------------------------------------------------
AgamGr9c        ------------------------------------------------------------
AgamGr9b        ------------------------------------------------------------
AgamGr9d        ------------------------------------------------------------
AgamGr9e        ------------------------------------------------------------
AgamGr9f        ------------------------------------------------------------
```

```
AgamGr9g        ------------------------------------------------------------
AgamGr9i        ------------------------------------------------------------
AgamGr9h        ------------------------------------------------------------
AgamGr9j        ------------------------------------------------------------
AgamGr9l        ------------------------------------------------------------
AgamGr9k        ------------------------------------------------------------
AgamGr9m        ------------------------------------------------------------
AgamGr9n        ------------------------------------------------------------
AgamGr10        ------------------------------------------------------------
AgamGr11        ------------------------------------------------------------
DmelGr23aA      ------------------------------------------------------------
DmelGr23aB      ------------------------------------------------------------
DmelGr39aA      ------------------------------------------------------------
DmelGr39aB      ------------------------------------------------------------
DmelGr39aC      ------------------------------------------------------------
DmelGr39aD      ------------------------------------------------------------
DmelGr98c       ------------------------------------------------------------
DmelGr98d       ------------------------------------------------------------
DmelGr98b       ------------------------------------------------------------
AgamGr51        ------------------------------------------------------------
AgamGr52        ------------------------------------------------------------
DmelGr94a       ------------------------------------------------------------
DmelGr97a       ------------------------------------------------------------
DmelGr93b       ------------------------------------------------------------
DmelGr93c       ------------------------------------------------------------
DmelGr22d       ------------------------------------------------------------
DmelGr22e       ------------------------------------------------------------
DmelGr22a       ------------------------------------------------------------
DmelGr22b       ------------------------------------------------------------
DmelGr22c       ------------------------------------------------------------
DmelGr22f       ------------------------------------------------------------
DmelGr36a       ------------------------------------------------------------
DmelGr36c       ------------------------------------------------------------
DmelGr36b       ------------------------------------------------------------
DmelGr59d       ------------------------------------------------------------
DmelGr59a       ------------------------------------------------------------
DmelGr59b       ------------------------------------------------------------
AgamGr26        ------------------------------------------------------------
AgamGr27        ------------------------------------------------------------
AgamGr29        ------------------------------------------------------------
AgamGr31        ------------------------------------------------------------
AgamGr30        ------------------------------------------------------------
AgamGr32a       ------------------------------------------------------------
TcasGr2         ------------------------------------------------------------
TcasGr9         ------------------------------------------------------------
TcasGr7         ------------------------------------------------------------
TcasGr30        ------------------------------------------------------------
TcasGr29        ------------------------------------------------------------
AaegGr64f       ------------------------------------------------------------
AaegGr64a3      ------------------------------------------------------------
AgamGr14        ------------------------------------------------------------
AgamGr18        ------------------------------------------------------------
AaegGr64d       ------------------------------------------------------------
AgamGr17        ------------------------------------------------------------
AaegGr64c       ------------------------------------------------------------
DmelGr64c       ------------------------------------------------------------
DmelGr64d       ------------------------------------------------------------
AgamGr20        ------------------------------------------------------------
AaegGr61a       ------------------------------------------------------------
AgamGr21        ------------------------------------------------------------
AaegGr64a       ------------------------------------------------------------
DmelGr64a       ------------------------------------------------------------
DmelGr61a       ------------------------------------------------------------
AgamGr15        ------------------------------------------------------------
AaegGr1         ------------------------------------------------------------
DmelGr5a        ------------------------------------------------------------
DmelGr64f       ------------------------------------------------------------
DmelGr64e       ------------------------------------------------------------
AmelGr1         ------------------------------------------------------------
AmelGr2         ------------------------------------------------------------
```

```
AgamGr16        ------------------------------------------------------------
AaegGr64e       ------------------------------------------------------------
TCasGr6         ------------------------------------------------------------
TcasGr26        ------------------------------------------------------------
TcasGr33        ------------------------------------------------------------
AgamGr23        ------------------------------------------------------------
AaegGr21a       ------------------------------------------------------------
TcasGr10        ------------------------------------------------------------
TcasGr39        ------------------------------------------------------------
AgamGr22        ------------------------------------------------------------
AaegGr21b       ------------------------------------------------------------
DmelGr21a       ------------------------------------------------------------
AgamGr24        ------------------------------------------------------------
AaegGr63a       ------------------------------------------------------------
DmelGr63a       ------------------------------------------------------------
TcasGr3         ------------------------------------------------------------
DmelOr83b       ------------------------------------------------------------
AgamOr7         ------------------------------------------------------------
HvirOr2         ------------------------------------------------------------
TcasOr16        ------------------------------------------------------------
TcasOr22        ------------------------------------------------------------
AmelOr2         ------------------------------------------------------------
TcasGr31        ------------------------------------------------------------
TcasGr35        ------------------------------------------------------------
DmelGr59c       ------------------------------------------------------------
DmelGr92a       ------------------------------------------------------------
DmelGr28a       ------------------------------------------------------------
DmelGr64b       ------------------------------------------------------------
TcasGr71        ------------------------------------------------------------
AgamGr19        ------------------------------------------------------------
AaegGr64b       ------------------------------------------------------------
DmelGr93d       ------------------------------------------------------------
TcasGr34        ------------------------------------------------------------
TcasGr43        ------------------------------------------------------------
TcasGr47        ------------------------------------------------------------
AmelGr3         ------------------------------------------------------------
AgamGr32b       ------------------------------------------------------------
AgamGr50        ------------------------------------------------------------
TcasGr20        ------------------------------------------------------------
TcasGr79        ------------------------------------------------------------
TcasGr15        ------------------------------------------------------------
TcasGr60        ------------------------------------------------------------
AgamGr38        ------------------------------------------------------------
TcasGr51        ------------------------------------------------------------
DmelGr39b       ------------------------------------------------------------
AgamGr28        ------------------------------------------------------------
TcasGr61        ------------------------------------------------------------
TcasGr50        ------------------------------------------------------------
AgamGr12        ------------------------------------------------------------
AgamGr36        ------------------------------------------------------------
AgamGr48        ------------------------------------------------------------
AgamGr49a       ------------------------------------------------------------
AgamGr49b       ------------------------------------------------------------
DmelGr2a        ------------------------------------------------------------
DmelGr32a       ------------------------------------------------------------
DmelGr68a       ------------------------------------------------------------
AmelGr7         ------------------------------------------------------------
AmelGr8         ------------------------------------------------------------
AmelGr9         ------------------------------------------------------------
TcasGr57        ------------------------------------------------------------
TcasGr41        ------------------------------------------------------------
AgamGr34        ------------------------------------------------------------
AgamGr35        ------------------------------------------------------------
AgamGr39        ------------------------------------------------------------
AgamGr40        ------------------------------------------------------------
AaegGr28d       ------------------------------------------------------------
AgamGr42        ------------------------------------------------------------
AgamGr41        ------------------------------------------------------------
AgamGr43        ------------------------------------------------------------
DmelGr33a       ------------------------------------------------------------
```

```
AgamGr44        ------------------------------------------------------------
AgamGr13        ------------------------------------------------------------
AgamGr45        ------------------------------------------------------------
AgamGr46        ------------------------------------------------------------
Dmelr93a        ------------------------------------------------------------
AmelGr6         ------------------------------------------------------------
AmelGr10        ------------------------------------------------------------
TcasGr28        ------------------------------------------------------------
TcasGr40        ------------------------------------------------------------
AgamGr3         ------------------------------------------------------------
AgamGr4         ------------------------------------------------------------
AgamGr6         ------------------------------------------------------------
AgamGr7         ------------------------------------------------------------
AgamGr47        ------------------------------------------------------------
DmelGr8a        ------------------------------------------------------------
AgamGr5         ------------------------------------------------------------
AgamGr8         ------------------------------------------------------------
DmelGr9a        ------------------------------------------------------------
DmelGr59f       ------------------------------------------------------------
DmelGr77a       ------------------------------------------------------------
DmelGr85a       ------------------------------------------------------------
TcasGr27        ------------------------------------------------------------
TcasGr48        ------------------------------------------------------------
AgamGr1         ------------------------------------------------------------
DmelGr10a       ------------------------------------------------------------
DmelGr59e       ------------------------------------------------------------
DmelGr10b       ------------------------------------------------------------
DmelGr89a       ------------------------------------------------------------
DmelGr57a       ------------------------------------------------------------
DmelGr47b       ------------------------------------------------------------
DmelGr58a       ------------------------------------------------------------
DmelGr58b       ------------------------------------------------------------
DmelGr98a       ------------------------------------------------------------
AaegGr93a       ------------------------------------------------------------
TcasGr19        ------------------------------------------------------------
DmelGr47a       ------------------------------------------------------------
DmelGr58c       ------------------------------------------------------------


TcasGr38        ------------------------------------------------------------
TcasGr46        TYLMFDYCTVLRVQRFNNYQSLAHYLRVFFTVFNVVHCYLASELVLMLKNRFVTLNVQLT 560
TcasGr1         ------------------------------------------------------------
TcasGr123       ------------------------------------------------------------
TcasGr62        ------------------------------------------------------------
TcasGr25        ------------------------------------------------------------
TcasGr59        ------------------------------------------------------------
AgamGr2         ------------------------------------------------------------
AaegGr66a       ------------------------------------------------------------
DmelGr66a       ------------------------------------------------------------
TcasGr11        ------------------------------------------------------------
TcasGr55        ------------------------------------------------------------
TcasGr21        ------------------------------------------------------------
TcasGr56        ------------------------------------------------------------
AaegGr          ------------------------------------------------------------
AaegGr28a       ------------------------------------------------------------
AgamGr33        ------------------------------------------------------------
DmelGr28bB      ------------------------------------------------------------
DmelGr28bC      ------------------------------------------------------------
DmelGr28bD      ------------------------------------------------------------
DmelGr28bA      ------------------------------------------------------------
DmelGr28bE      ------------------------------------------------------------
AgamGr25        ------------------------------------------------------------
AaegGr43a       ------------------------------------------------------------
DmelGr43a       ------------------------------------------------------------
AgamGr37b       ------------------------------------------------------------
AgamGr37d       ------------------------------------------------------------
AgamGr37a       ------------------------------------------------------------
AgamGr37c       ------------------------------------------------------------
AgamGr37e       ------------------------------------------------------------
AgamGr37f       ------------------------------------------------------------
```

```
AaegGr28c       ------------------------------------------------------------
AaegGr28b       ------------------------------------------------------------
AaegGr28e       ------------------------------------------------------------
TcasGr14        ------------------------------------------------------------
TcasGr49        ------------------------------------------------------------
AmelGr4         ------------------------------------------------------------
AmelGr5         ------------------------------------------------------------
TcasGr16        ------------------------------------------------------------
TcasGr22        ------------------------------------------------------------
TcasGr17        ------------------------------------------------------------
TcasGr150       ------------------------------------------------------------
TcasGr32        ------------------------------------------------------------
TcasGr54        ------------------------------------------------------------
TcasGr37        ------------------------------------------------------------
TcasGr53        ------------------------------------------------------------
TcasGr5         ------------------------------------------------------------
TcasGr12        ------------------------------------------------------------
TcasGr13        ------------------------------------------------------------
TcasGr104       ------------------------------------------------------------
TcasGr45        ------------------------------------------------------------
TcasGr98        ------------------------------------------------------------
TcasGr52        ------------------------------------------------------------
TcasGr105       ------------------------------------------------------------
TcasGr4         ------------------------------------------------------------
TcasGr44        ------------------------------------------------------------
AgamGr9a        ------------------------------------------------------------
AgamGr9c        ------------------------------------------------------------
AgamGr9b        ------------------------------------------------------------
AgamGr9d        ------------------------------------------------------------
AgamGr9e        ------------------------------------------------------------
AgamGr9f        ------------------------------------------------------------
AgamGr9g        ------------------------------------------------------------
AgamGr9i        ------------------------------------------------------------
AgamGr9h        ------------------------------------------------------------
AgamGr9j        ------------------------------------------------------------
AgamGr9l        ------------------------------------------------------------
AgamGr9k        ------------------------------------------------------------
AgamGr9m        ------------------------------------------------------------
AgamGr9n        ------------------------------------------------------------
AgamGr10        ------------------------------------------------------------
AgamGr11        ------------------------------------------------------------
DmelGr23aA      ------------------------------------------------------------
DmelGr23aB      ------------------------------------------------------------
DmelGr39aA      ------------------------------------------------------------
DmelGr39aB      ------------------------------------------------------------
DmelGr39aC      ------------------------------------------------------------
DmelGr39aD      ------------------------------------------------------------
DmelGr98c       ------------------------------------------------------------
DmelGr98d       ------------------------------------------------------------
DmelGr98b       ------------------------------------------------------------
AgamGr51        ------------------------------------------------------------
AgamGr52        ------------------------------------------------------------
DmelGr94a       ------------------------------------------------------------
DmelGr97a       ------------------------------------------------------------
DmelGr93b       ------------------------------------------------------------
DmelGr93c       ------------------------------------------------------------
DmelGr22d       ------------------------------------------------------------
DmelGr22e       ------------------------------------------------------------
DmelGr22a       ------------------------------------------------------------
DmelGr22b       ------------------------------------------------------------
DmelGr22c       ------------------------------------------------------------
DmelGr22f       ------------------------------------------------------------
DmelGr36a       ------------------------------------------------------------
DmelGr36c       ------------------------------------------------------------
DmelGr36b       ------------------------------------------------------------
DmelGr59d       ------------------------------------------------------------
DmelGr59a       ------------------------------------------------------------
DmelGr59b       ------------------------------------------------------------
AgamGr26        ------------------------------------------------------------
AgamGr27        ------------------------------------------------------------
```

```
AgamGr29        ------------------------------------------------------------
AgamGr31        ------------------------------------------------------------
AgamGr30        ------------------------------------------------------------
AgamGr32a       ------------------------------------------------------------
TcasGr2         ------------------------------------------------------------
TcasGr9         ------------------------------------------------------------
TcasGr7         ------------------------------------------------------------
TcasGr30        ------------------------------------------------------------
TcasGr29        ------------------------------------------------------------
AaegGr64f       ------------------------------------------------------------
AaegGr64a3      ------------------------------------------------------------
AgamGr14        ------------------------------------------------------------
AgamGr18        ------------------------------------------------------------
AaegGr64d       ------------------------------------------------------------
AgamGr17        ------------------------------------------------------------
AaegGr64c       ------------------------------------------------------------
DmelGr64c       ------------------------------------------------------------
DmelGr64d       ------------------------------------------------------------
AgamGr20        ------------------------------------------------------------
AaegGr61a       ------------------------------------------------------------
AgamGr21        ------------------------------------------------------------
AaegGr64a       ------------------------------------------------------------
DmelGr64a       ------------------------------------------------------------
DmelGr61a       ------------------------------------------------------------
AgamGr15        ------------------------------------------------------------
AaegGr1         ------------------------------------------------------------
DmelGr5a        ------------------------------------------------------------
DmelGr64f       ------------------------------------------------------------
DmelGr64e       ------------------------------------------------------------
AmelGr1         ------------------------------------------------------------
AmelGr2         ------------------------------------------------------------
AgamGr16        ------------------------------------------------------------
AaegGr64e       ------------------------------------------------------------
TCasGr6         ------------------------------------------------------------
TcasGr26        ------------------------------------------------------------
TcasGr33        ------------------------------------------------------------
AgamGr23        ------------------------------------------------------------
AaegGr21a       ------------------------------------------------------------
TcasGr10        ------------------------------------------------------------
TcasGr39        ------------------------------------------------------------
AgamGr22        ------------------------------------------------------------
AaegGr21b       ------------------------------------------------------------
DmelGr21a       ------------------------------------------------------------
AgamGr24        ------------------------------------------------------------
AaegGr63a       ------------------------------------------------------------
DmelGr63a       ------------------------------------------------------------
TcasGr3         ------------------------------------------------------------
DmelOr83b       ------------------------------------------------------------
AgamOr7         ------------------------------------------------------------
HvirOr2         ------------------------------------------------------------
TcasOr16        ------------------------------------------------------------
TcasOr22        ------------------------------------------------------------
AmelOr2         ------------------------------------------------------------
TcasGr31        ------------------------------------------------------------
TcasGr35        ------------------------------------------------------------
DmelGr59c       ------------------------------------------------------------
DmelGr92a       ------------------------------------------------------------
DmelGr28a       ------------------------------------------------------------
DmelGr64b       ------------------------------------------------------------
TcasGr71        ------------------------------------------------------------
AgamGr19        ------------------------------------------------------------
AaegGr64b       ------------------------------------------------------------
DmelGr93d       ------------------------------------------------------------
TcasGr34        ------------------------------------------------------------
TcasGr43        ------------------------------------------------------------
TcasGr47        ------------------------------------------------------------
AmelGr3         ------------------------------------------------------------
AgamGr32b       ------------------------------------------------------------
AgamGr50        ------------------------------------------------------------
TcasGr20        ------------------------------------------------------------
```

```
TcasGr79        ------------------------------------------------------------
TcasGr15        ------------------------------------------------------------
TcasGr60        ------------------------------------------------------------
AgamGr38        ------------------------------------------------------------
TcasGr51        ------------------------------------------------------------
DmelGr39b       ------------------------------------------------------------
AgamGr28        ------------------------------------------------------------
TcasGr61        ------------------------------------------------------------
TcasGr50        ------------------------------------------------------------
AgamGr12        ------------------------------------------------------------
AgamGr36        ------------------------------------------------------------
AgamGr48        ------------------------------------------------------------
AgamGr49a       ------------------------------------------------------------
AgamGr49b       ------------------------------------------------------------
DmelGr2a        ------------------------------------------------------------
DmelGr32a       ------------------------------------------------------------
DmelGr68a       ------------------------------------------------------------
AmelGr7         ------------------------------------------------------------
AmelGr8         ------------------------------------------------------------
AmelGr9         ------------------------------------------------------------
TcasGr57        ------------------------------------------------------------
TcasGr41        ------------------------------------------------------------
AgamGr34        ------------------------------------------------------------
AgamGr35        ------------------------------------------------------------
AgamGr39        ------------------------------------------------------------
AgamGr40        ------------------------------------------------------------
AaegGr28d       ------------------------------------------------------------
AgamGr42        ------------------------------------------------------------
AgamGr41        ------------------------------------------------------------
AgamGr43        ------------------------------------------------------------
DmelGr33a       ------------------------------------------------------------
AgamGr44        ------------------------------------------------------------
AgamGr13        ------------------------------------------------------------
AgamGr45        ------------------------------------------------------------
AgamGr46        ------------------------------------------------------------
Dmelr93a        ------------------------------------------------------------
AmelGr6         ------------------------------------------------------------
AmelGr10        ------------------------------------------------------------
TcasGr28        ------------------------------------------------------------
TcasGr40        ------------------------------------------------------------
AgamGr3         ------------------------------------------------------------
AgamGr4         ------------------------------------------------------------
AgamGr6         ------------------------------------------------------------
AgamGr7         ------------------------------------------------------------
AgamGr47        ------------------------------------------------------------
DmelGr8a        ------------------------------------------------------------
AgamGr5         ------------------------------------------------------------
AgamGr8         ------------------------------------------------------------
DmelGr9a        ------------------------------------------------------------
DmelGr59f       ------------------------------------------------------------
DmelGr77a       ------------------------------------------------------------
DmelGr85a       ------------------------------------------------------------
TcasGr27        ------------------------------------------------------------
TcasGr48        ------------------------------------------------------------
AgamGr1         ------------------------------------------------------------
DmelGr10a       ------------------------------------------------------------
DmelGr59e       ------------------------------------------------------------
DmelGr10b       ------------------------------------------------------------
DmelGr89a       ------------------------------------------------------------
DmelGr57a       ------------------------------------------------------------
DmelGr47b       ------------------------------------------------------------
DmelGr58a       ------------------------------------------------------------
DmelGr58b       ------------------------------------------------------------
DmelGr98a       ------------------------------------------------------------
AaegGr93a       ------------------------------------------------------------
TcasGr19        ------------------------------------------------------------
DmelGr47a       ------------------------------------------------------------
DmelGr58c       ------------------------------------------------------------
```

```
TcasGr38        ------------------------------------------------------------
TcasGr46        KLTKNCATKAQSVVLGRICTLHHHLSKLVTRFNEIFGLGLLLMFGVSFLLITQTIFIICV 620
TcasGr1         ------------------------------------------------------------
TcasGr123       ------------------------------------------------------------
TcasGr62        ------------------------------------------------------------
TcasGr25        ------------------------------------------------------------
TcasGr59        ------------------------------------------------------------
AgamGr2         ------------------------------------------------------------
AaegGr66a       ------------------------------------------------------------
DmelGr66a       ------------------------------------------------------------
TcasGr11        ------------------------------------------------------------
TcasGr55        ------------------------------------------------------------
TcasGr21        ------------------------------------------------------------
TcasGr56        ------------------------------------------------------------
AaegGr          ------------------------------------------------------------
AaegGr28a       ------------------------------------------------------------
AgamGr33        ------------------------------------------------------------
DmelGr28bB      ------------------------------------------------------------
DmelGr28bC      ------------------------------------------------------------
DmelGr28bD      ------------------------------------------------------------
DmelGr28bA      ------------------------------------------------------------
DmelGr28bE      ------------------------------------------------------------
AgamGr25        ------------------------------------------------------------
AaegGr43a       ------------------------------------------------------------
DmelGr43a       ------------------------------------------------------------
AgamGr37b       ------------------------------------------------------------
AgamGr37d       ------------------------------------------------------------
AgamGr37a       ------------------------------------------------------------
AgamGr37c       ------------------------------------------------------------
AgamGr37e       ------------------------------------------------------------
AgamGr37f       ------------------------------------------------------------
AaegGr28c       ------------------------------------------------------------
AaegGr28b       ------------------------------------------------------------
AaegGr28e       ------------------------------------------------------------
TcasGr14        ------------------------------------------------------------
TcasGr49        ------------------------------------------------------------
AmelGr4         ------------------------------------------------------------
AmelGr5         ------------------------------------------------------------
TcasGr16        ------------------------------------------------------------
TcasGr22        ------------------------------------------------------------
TcasGr17        ------------------------------------------------------------
TcasGr150       ------------------------------------------------------------
TcasGr32        ------------------------------------------------------------
TcasGr54        ------------------------------------------------------------
TcasGr37        ------------------------------------------------------------
TcasGr53        ------------------------------------------------------------
TcasGr5         ------------------------------------------------------------
TcasGr12        ------------------------------------------------------------
TcasGr13        ------------------------------------------------------------
TcasGr104       ------------------------------------------------------------
TcasGr45        ------------------------------------------------------------
TcasGr98        ------------------------------------------------------------
TcasGr52        ------------------------------------------------------------
TcasGr105       ------------------------------------------------------------
TcasGr4         ------------------------------------------------------------
TcasGr44        ------------------------------------------------------------
AgamGr9a        ------------------------------------------------------------
AgamGr9c        ------------------------------------------------------------
AgamGr9b        ------------------------------------------------------------
AgamGr9d        ------------------------------------------------------------
AgamGr9e        ------------------------------------------------------------
AgamGr9f        ------------------------------------------------------------
AgamGr9g        ------------------------------------------------------------
AgamGr9i        ------------------------------------------------------------
AgamGr9h        ------------------------------------------------------------
AgamGr9j        ------------------------------------------------------------
AgamGr9l        ------------------------------------------------------------
AgamGr9k        ------------------------------------------------------------
AgamGr9m        ------------------------------------------------------------
AgamGr9n        ------------------------------------------------------------
```

```
AgamGr10        ------------------------------------------------------------
AgamGr11        ------------------------------------------------------------
DmelGr23aA      ------------------------------------------------------------
DmelGr23aB      ------------------------------------------------------------
DmelGr39aA      ------------------------------------------------------------
DmelGr39aB      ------------------------------------------------------------
DmelGr39aC      ------------------------------------------------------------
DmelGr39aD      ------------------------------------------------------------
DmelGr98c       ------------------------------------------------------------
DmelGr98d       ------------------------------------------------------------
DmelGr98b       ------------------------------------------------------------
AgamGr51        ------------------------------------------------------------
AgamGr52        ------------------------------------------------------------
DmelGr94a       ------------------------------------------------------------
DmelGr97a       ------------------------------------------------------------
DmelGr93b       ------------------------------------------------------------
DmelGr93c       ------------------------------------------------------------
DmelGr22d       ------------------------------------------------------------
DmelGr22e       ------------------------------------------------------------
DmelGr22a       ------------------------------------------------------------
DmelGr22b       ------------------------------------------------------------
DmelGr22c       ------------------------------------------------------------
DmelGr22f       ------------------------------------------------------------
DmelGr36a       ------------------------------------------------------------
DmelGr36c       ------------------------------------------------------------
DmelGr36b       ------------------------------------------------------------
DmelGr59d       ------------------------------------------------------------
DmelGr59a       ------------------------------------------------------------
DmelGr59b       ------------------------------------------------------------
AgamGr26        ------------------------------------------------------------
AgamGr27        ------------------------------------------------------------
AgamGr29        ------------------------------------------------------------
AgamGr31        ------------------------------------------------------------
AgamGr30        ------------------------------------------------------------
AgamGr32a       ------------------------------------------------------------
TcasGr2         ------------------------------------------------------------
TcasGr9         ------------------------------------------------------------
TcasGr7         ------------------------------------------------------------
TcasGr30        ------------------------------------------------------------
TcasGr29        ------------------------------------------------------------
AaegGr64f       ------------------------------------------------------------
AaegGr64a3      ------------------------------------------------------------
AgamGr14        ------------------------------------------------------------
AgamGr18        ------------------------------------------------------------
AaegGr64d       ------------------------------------------------------------
AgamGr17        ------------------------------------------------------------
AaegGr64c       ------------------------------------------------------------
DmelGr64c       ------------------------------------------------------------
DmelGr64d       ------------------------------------------------------------
AgamGr20        ------------------------------------------------------------
AaegGr61a       ------------------------------------------------------------
AgamGr21        ------------------------------------------------------------
AaegGr64a       ------------------------------------------------------------
DmelGr64a       ------------------------------------------------------------
DmelGr61a       ------------------------------------------------------------
AgamGr15        ------------------------------------------------------------
AaegGr1         ------------------------------------------------------------
DmelGr5a        ------------------------------------------------------------
DmelGr64f       ------------------------------------------------------------
DmelGr64e       ------------------------------------------------------------
AmelGr1         ------------------------------------------------------------
AmelGr2         ------------------------------------------------------------
AgamGr16        ------------------------------------------------------------
AaegGr64e       ------------------------------------------------------------
TCasGr6         ------------------------------------------------------------
TcasGr26        ------------------------------------------------------------
TcasGr33        ------------------------------------------------------------
AgamGr23        ------------------------------------------------------------
AaegGr21a       ------------------------------------------------------------
TcasGr10        ------------------------------------------------------------
```

```
TcasGr39        ------------------------------------------------------------
AgamGr22        ------------------------------------------------------------
AaegGr21b       ------------------------------------------------------------
DmelGr21a       ------------------------------------------------------------
AgamGr24        ------------------------------------------------------------
AaegGr63a       ------------------------------------------------------------
DmelGr63a       ------------------------------------------------------------
TcasGr3         ------------------------------------------------------------
DmelOr83b       ------------------------------------------------------------
AgamOr7         ------------------------------------------------------------
HvirOr2         ------------------------------------------------------------
TcasOr16        ------------------------------------------------------------
TcasOr22        ------------------------------------------------------------
AmelOr2         ------------------------------------------------------------
TcasGr31        ------------------------------------------------------------
TcasGr35        ------------------------------------------------------------
DmelGr59c       ------------------------------------------------------------
DmelGr92a       ------------------------------------------------------------
DmelGr28a       ------------------------------------------------------------
DmelGr64b       ------------------------------------------------------------
TcasGr71        ------------------------------------------------------------
AgamGr19        ------------------------------------------------------------
AaegGr64b       ------------------------------------------------------------
DmelGr93d       ------------------------------------------------------------
TcasGr34        ------------------------------------------------------------
TcasGr43        ------------------------------------------------------------
TcasGr47        ------------------------------------------------------------
AmelGr3         ------------------------------------------------------------
AgamGr32b       ------------------------------------------------------------
AgamGr50        ------------------------------------------------------------
TcasGr20        ------------------------------------------------------------
TcasGr79        ------------------------------------------------------------
TcasGr15        ------------------------------------------------------------
TcasGr60        ------------------------------------------------------------
AgamGr38        ------------------------------------------------------------
TcasGr51        ------------------------------------------------------------
DmelGr39b       ------------------------------------------------------------
AgamGr28        ------------------------------------------------------------
TcasGr61        ------------------------------------------------------------
TcasGr50        ------------------------------------------------------------
AgamGr12        ------------------------------------------------------------
AgamGr36        ------------------------------------------------------------
AgamGr48        ------------------------------------------------------------
AgamGr49a       ------------------------------------------------------------
AgamGr49b       ------------------------------------------------------------
DmelGr2a        ------------------------------------------------------------
DmelGr32a       ------------------------------------------------------------
DmelGr68a       ------------------------------------------------------------
AmelGr7         ------------------------------------------------------------
AmelGr8         ------------------------------------------------------------
AmelGr9         ------------------------------------------------------------
TcasGr57        ------------------------------------------------------------
TcasGr41        ------------------------------------------------------------
AgamGr34        ------------------------------------------------------------
AgamGr35        ------------------------------------------------------------
AgamGr39        ------------------------------------------------------------
AgamGr40        ------------------------------------------------------------
AaegGr28d       ------------------------------------------------------------
AgamGr42        ------------------------------------------------------------
AgamGr41        ------------------------------------------------------------
AgamGr43        ------------------------------------------------------------
DmelGr33a       ------------------------------------------------------------
AgamGr44        ------------------------------------------------------------
AgamGr13        ------------------------------------------------------------
AgamGr45        ------------------------------------------------------------
AgamGr46        ------------------------------------------------------------
Dmelr93a        ------------------------------------------------------------
AmelGr6         ------------------------------------------------------------
AmelGr10        ------------------------------------------------------------
TcasGr28        ------------------------------------------------------------
```

```
TcasGr40        ------------------------------------------------------------
AgamGr3         ------------------------------------------------------------
AgamGr4         ------------------------------------------------------------
AgamGr6         ------------------------------------------------------------
AgamGr7         ------------------------------------------------------------
AgamGr47        ------------------------------------------------------------
DmelGr8a        ------------------------------------------------------------
AgamGr5         ------------------------------------------------------------
AgamGr8         ------------------------------------------------------------
DmelGr9a        ------------------------------------------------------------
DmelGr59f       ------------------------------------------------------------
DmelGr77a       ------------------------------------------------------------
DmelGr85a       ------------------------------------------------------------
TcasGr27        ------------------------------------------------------------
TcasGr48        ------------------------------------------------------------
AgamGr1         ------------------------------------------------------------
DmelGr10a       ------------------------------------------------------------
DmelGr59e       ------------------------------------------------------------
DmelGr10b       ------------------------------------------------------------
DmelGr89a       ------------------------------------------------------------
DmelGr57a       ------------------------------------------------------------
DmelGr47b       ------------------------------------------------------------
DmelGr58a       ------------------------------------------------------------
DmelGr58b       ------------------------------------------------------------
DmelGr98a       ------------------------------------------------------------
AaegGr93a       ------------------------------------------------------------
TcasGr19        ------------------------------------------------------------
DmelGr47a       ------------------------------------------------------------
DmelGr58c       ------------------------------------------------------------


TcasGr38        --------------------------------------QVSKAGELIHQIQ--TEDHDII 357
TcasGr46        IVQSEQIAWLHLLYIFLVGIMYAADVFYICHVCCSTIHEVSKAGELIHKIE--TNDHEII 678
TcasGr1         ------------------------------------FYKVSKSGELIHKIE--TNEHEII 337
TcasGr123       -----------------------------------------KAGELIHKID--TEDHDIR 330
TcasGr62        -----------------------------------------KAGELIHKID--TEDHDIR 605
TcasGr25        --------------------------------------AAPAVSKARQFLPALPGYVPVY 229
TcasGr59        --------------------------------------AAPAVSKARQFLPALPGYVPVY 404
AgamGr2         --------------------------------------DSQRTGIELHYLANAVDEVHCY 358
AaegGr66a       --------------------------------------ESQRTGVFMHHLANMVDETHFY 420
DmelGr66a       --------------------------------------ASKRTGISLHKCGVVADDNLLY 434
TcasGr11        --------------------------------------NDTPSILHELRNNYF--HMDLE 337
TcasGr55        --------------------------------------NDTPSILHELRNNYF--HMDLE 337
TcasGr21        --------------------------------------VAGVTTYLVILIQFNNSDFVQR 374
TcasGr56        --------------------------------------SKKCYK---------------- 358
AaegGr          --------------------------------------KQIAVNVHKLINVNN-YDDELT 376
AaegGr28a       ----------------------------------------VMLRI--------------- 255
AgamGr33        --------------------------------------DNIGSNVHKLLNAAAGADSELA 374
DmelGr28bB      --------------------------------------EKTGGIVHSLLNKTK--SAEVK 369
DmelGr28bC      --------------------------------------EKTGGIVHSLLNKTK--SAEVK 396
DmelGr28bD      --------------------------------------EKTGGIVHSLLNKTK--SAEVK 366
DmelGr28bA      --------------------------------------EKTGGIVHSLLNKTK--SAEVK 378
DmelGr28bE      --------------------------------------EKTGGIVHSLLNKTK--SAEVK 373
AgamGr25        --------------------------------------NRTITIINSLRRTCK--DVEMI 397
AaegGr43a       ---------------------------------------RLISLL--------------- 323
DmelGr43a       --------------------------------------RKTIQIVCEIERKVH--EPILA 375
AgamGr37b       ------------------------------------------------------------
AgamGr37d       ------------------------------------------------------------
AgamGr37a       ------------------------------------------------------------
AgamGr37c       ------------------------------------------------------------
AgamGr37e       ------------------------------------------------------------
AgamGr37f       ------------------------------------------------------------
AaegGr28c       --------------------------------------GVLVHKAINCASSSA-----VI 507
AaegGr28b       --------------------------------------AALVHKALRETSNDR-----IR 356
AaegGr28e       --------------------------------------AALVHKALRETSNDR-----IR 356
TcasGr14        ---------------------------------------ATFVHEIWNKYALKNEVDKRV 325
TcasGr49        ---------------------------------------ATFVHEIWNKYALKNEVDKRV 369
AmelGr4         ---------------------------------------GCIVHILLNCVIDQ----KVK 350
AmelGr5         ---------------------------------------GCIIHVLLNCTIDR----ETK 349
TcasGr16        ---------------------------------------KKLIVKIKIDIDKE----DER 337
```

```
TcasGr22        ---------------------------------------KILLVGLKIDIDQE----EER 240
TcasGr17        ---------------------------------------KTVLLGVKIDICNE----EER 324
TcasGr150       ---------------------------------------KTVLLGVKIDICNE----EER 324
TcasGr32        --------------------------------------------ISYSLE-KLKTG-DEI 305
TcasGr54        --------------------------------------------ISYSLE-KLKTG-DEI 305
TcasGr37        --------------------------------------------AVYSLDLELKHI-ENF 280
TcasGr53        --------------------------------------------AVYSLDLELKHI-ENF 280
TcasGr5         --------------------------------------------LAYKIEAKRRD--NEI 292
TcasGr12        --------------------------------------------VLKKIALRRQTTNGDL 264
TcasGr13        --------------------------------------------VLKKIALRRQTTNGDL 338
TcasGr104       --------------------------------------------WCYKLQEQFVTNSEVR 326
TcasGr45        --------------------------------------------WCYKLQEQFVTNSEVR 326
TcasGr98        --------------------------------------------LCYKLMEKFPERSHEQ 323
TcasGr52        --------------------------------------------MCYKLQGFYPEEAPQR 260
TcasGr105       --------------------------------------------VCYEYQDRFSDESKKR 306
TcasGr4         ------------------------------------------------------------
TcasGr44        ------------------------------------------------------------
AgamGr9a        -----------------------------------------------HFDDYRLQNTRAA 325
AgamGr9c        -----------------------------------------------HFDDYRLQNTRAA 324
AgamGr9b        -----------------------------------------------HFDDYRLQNTRAA 375
AgamGr9d        -----------------------------------------------HFDDYRLQNTRAA 331
AgamGr9e        -----------------------------------------------HFDDYRLQNTRAA 327
AgamGr9f        -----------------------------------------------HFDDYRLQNTRAA 324
AgamGr9g        -----------------------------------------------HFDDYRLQNTRAA 327
AgamGr9i        -----------------------------------------------HFDDYRLQNTRAA 328
AgamGr9h        -----------------------------------------------HFDDYRLQNTRAA 333
AgamGr9j        -----------------------------------------------HFDDYRLQNTRAA 364
AgamGr9l        -----------------------------------------------HFDDYRLQNTRAA 354
AgamGr9k        -----------------------------------------------HFDDYRLQNTRAA 346
AgamGr9m        -----------------------------------------------HFDDYRLQNTRAA 349
AgamGr9n        -----------------------------------------------HFDDYRLQNTRAA 316
AgamGr10        -----------------------------------------------HFDDYRLQNTRAA 316
AgamGr11        -----------------------------------------------RLDDDRNRNSRVA 321
DmelGr23aA      -----------------------------------------------LVKP--QGSKLYN 311
DmelGr23aB      -----------------------------------------------LVKP--QGSKLYN 315
DmelGr39aA      -----------------------------------------------VPRT--GTG--LD 312
DmelGr39aB      -----------------------------------------------VPRT--GTG--LD 321
DmelGr39aC      -----------------------------------------------VPRT--GTG--LD 321
DmelGr39aD      -----------------------------------------------VPRT--GTG--LD 311
DmelGr98c       -----------------------------------------------LPTA--EEFQMLK 343
DmelGr98d       -----------------------------------------------LSAA--EDYLILK 341
DmelGr98b       -----------------------------------------------TSAD--PNFAMLT 341
AgamGr51        -----------------------------------------------VSVNQHHHRPGQE 356
AgamGr52        ------------------------------------------------AINHRSRGRRME 351
DmelGr94a       -----------------------------------------------QEMNLQHADSRYR 348
DmelGr97a       -----------------------------------------------QRFDLQHADARFK 369
DmelGr93b       -----------------------------------------------ENFYVTDSQS-YH 347
DmelGr93c       -----------------------------------------------ENFPITDHKA-WH 343
DmelGr22d       ------------------------------------------------FNDIENMDKDLE 335
DmelGr22e       ------------------------------------------------FNDIENIDEKLE 337
DmelGr22a       ------------------------------------------------FNDMENMDQETE 337
DmelGr22b       ------------------------------------------------FSDLEHRDDKLE 334
DmelGr22c       ------------------------------------------------FADLEVKDIELE 331
DmelGr22f       ------------------------------------------------FTDLEHDDEELE 326
DmelGr36a       ------------------------------------------------TLFTSALDVRLE 339
DmelGr36c       ------------------------------------------------TIFVG-LDVRLE 338
DmelGr36b       ------------------------------------------------TVFASSLDIRLE 339
DmelGr59d       ------------------------------------------------TLFQPGLDHRLE 338
DmelGr59a       -------------------------------------------------------ESSMS 312
DmelGr59b       -------------------------------------------------------EVRWT 313
AgamGr26        -------------------------------------------------AALDSALNSAL 367
AgamGr27        -------------------------------------------------YEAAGRSNSAL 351
AgamGr29        -------------------------------------------------GNDSRSNEITN 363
AgamGr31        -------------------------------------------------SSSSRCHEITN 364
AgamGr30        -------------------------------------------------WNSELNGMVDY 355
AgamGr32a       -------------------------------------------------WSSEQN-MT-- 366
TcasGr2         ----------------------------------------------------------YN 361
TcasGr9         ----------------------------------------------------------YN 362
TcasGr7         ----------------------------------------------------------YN 363
TcasGr30        ------------------------------------------------------------
```

```
TcasGr29        ----------------------------------------------------------YN 363
AaegGr64f       ----------------------------------------------------------WN 320
AaegGr64a3      ------------------------------------------------------------
AgamGr14        ----------------------------------------------------------WN 384
AgamGr18        ----------------------------------------------------------WG 356
AaegGr64d       ----------------------------------------------------------WG 388
AgamGr17        ----------------------------------------------------------WT 345
AaegGr64c       ----------------------------------------------------------WT 370
DmelGr64c       ----------------------------------------------------------WC 349
DmelGr64d       ----------------------------------------------------------WC 358
AgamGr20        ----------------------------------------------------------YN 376
AaegGr61a       ----------------------------------------------------------YC 373
AgamGr21        ----------------------------------------------------------WC 401
AaegGr64a       ----------------------------------------------------------WS 284
DmelGr64a       ----------------------------------------------------------WC 395
DmelGr61a       ----------------------------------------------------------WT 378
AgamGr15        ----------------------------------------------------------WC 382
AaegGr1         ----------------------------------------------------------WC 386
DmelGr5a        ----------------------------------------------------------YH 382
DmelGr64f       ----------------------------------------------------------WC 407
DmelGr64e       ----------------------------------------------------------WC 401
AmelGr1         ----------------------------------------------------------FS 330
AmelGr2         ----------------------------------------------------------YS 420
AgamGr16        ----------------------------------------------------------YT 352
AaegGr64e       ----------------------------------------------------------YS 373
TCasGr6         ----------------------------------------------------------TR 331
TcasGr26        ----------------------------------------------------------TR 331
TcasGr33        ----------------------------------------------------------TR 331
AgamGr23        ----------------------------------------------------------TQ 415
AaegGr21a       ----------------------------------------------------------TQ 407
TcasGr10        ----------------------------------------------------------TR 361
TcasGr39        ----------------------------------------------------------TR 361
AgamGr22        ----------------------------------------------------------TQ 380
AaegGr21b       ----------------------------------------------------------TQ 386
DmelGr21a       ----------------------------------------------------------TQ 387
AgamGr24        ----------------------------------------------------------AQ 311
AaegGr63a       ----------------------------------------------------------AQ 376
DmelGr63a       ----------------------------------------------------------AQ 405
TcasGr3         ----------------------------------------------------------AQ 367
DmelOr83b       -----------------------------------------------------------A 438
AgamOr7         -----------------------------------------------------------A 430
HvirOr2         -----------------------------------------------------------A 424
TcasOr16        -----------------------------------------------------------A 526
TcasOr22        -----------------------------------------------------------A 565
AmelOr2         -----------------------------------------------------------A 430
TcasGr31        ------------------------------------------------------------
TcasGr35        ------------------------------------------------------------
DmelGr59c       ------------------------------------------------TLFYRELDNRLE 343
DmelGr92a       ----------------------------------------------------VGDLSKFQ 332
DmelGr28a       ----------------------------------------------------ITDDPELR 379
DmelGr64b       ---------------------------------------------------------AWS 343
TcasGr71        ---------------------------------------------------------NEK 296
AgamGr19        ----------------------------------------------------------WC 338
AaegGr64b       ----------------------------------------------------------WC 286
DmelGr93d       ---------------------------------------------------------HWM 324
TcasGr34        ---------------------------------------------------------AQK 273
TcasGr43        --------------------------------------------------------MEEK 303
TcasGr47        ----------------------------------------------------------DK 272
AmelGr3         ----------------------------------------------------------IR 392
AgamGr32b       --------------------------------------------------------VELN 364
AgamGr50        --------------------------------------QARQIIPCLSMYTGRITDVPGD 353
TcasGr20        ---------------------------------------------------------DIK 338
TcasGr79        ----------------------------------------------------------KK 297
TcasGr15        -----------------------------------------------VEYKNVEFWRNIR 340
TcasGr60        -----------------------------------------------------------R 290
AgamGr38        ---------------------------AELIEMCAQLCFASTFDFVLVFGLISTLCESVK 373
TcasGr51        --------------------------------------------------------FRNS 718
DmelGr39b       ------------------------------------------------------RETSYK 319
AgamGr28        ---------------------------------------------------KAATVGEDQ 329
TcasGr61        ---------------------------------------------------------SLE 614
```

```
TcasGr50        ------------------------------------------------------------
AgamGr12        -------------------------------------------------LNTQRVEARKW 326
AgamGr36        -----------------------------------------------------VVDSSTK 343
AgamGr48        ----------------------------------------------------AWLDTRTE 349
AgamGr49a       ---------------------------------------------------------RVE 349
AgamGr49b       ------------------------------------------------EFLPTDPNPRVE 353
DmelGr2a        -------------------------------------------------------NESHN 353
DmelGr32a       ---------------------------------------------------------EYQ 398
DmelGr68a       ------------------------------------------------------KSKQFQ 328
AmelGr7         -----------------------------------------------------FATKEFQ 351
AmelGr8         --------------------------------------------------------DQII 343
AmelGr9         ------------------------------------------------------TDPMVR 337
TcasGr57        ---------------------------------------------EIPTMPVSIKDQTIK 338
TcasGr41        --------------------------------------ICYKLLSNLGIFPKTTNEQLLR 344
AgamGr34        ---------------------------------------------------GSRYMSSEE 363
AgamGr35        ------------------------------------------------------MKHSQV 380
AgamGr39        --------------------------------------------------------AAIY 358
AgamGr40        ------------------------------------------------------YYDMPI 337
AaegGr28d       -----------------------------------------------------RYDRRVV 344
AgamGr42        -------------------------------------------------------KTPAL 364
AgamGr41        ----------------------------------------NVITFKLVHRIINTTNVVEI 386
AgamGr43        -------------------------------------------------YFIDDQALFER 368
DmelGr33a       -----------------------------------------------KKPAFMLSNDLFY 413
AgamGr44        ------------------------------------------------------MDHKLK 344
AgamGr13        -------------------------------------------------------DRYFH 347
AgamGr45        -----------------------------------------------------VYGPCQR 379
AgamGr46        --------------------------------------------------------TPLS 370
Dmelr93a        ----------------------------------------------------------WD 355
AmelGr6         ----------------------------------------------FSSNGMQGQYKNFH 387
AmelGr10        ----------------------------------------------LLFVQANRVGELIF 327
TcasGr28        ------------------------------------------------------------
TcasGr40        --------------------------------------------------------AAAV 325
AgamGr3         ------------------------------------------------------LDAEMV 325
AgamGr4         -------------------------------------------------------DSTFR 363
AgamGr6         --------------------------------------------------------QTTI 357
AgamGr7         -----------------------------------------------------DEADLLA 366
AgamGr47        -----------------------------------------------------HFSVDVI 349
DmelGr8a        ---------------------------------------------------------DLS 329
AgamGr5         --------------------------------------------------------QALF 377
AgamGr8         -------------------------------------------------------CDQGW 317
DmelGr9a        ------------------------------------------------------QTPVER 288
DmelGr59f       --------------------------------------------------------KDIQ 340
DmelGr77a       -----------------------------------------------------ITVKQLR 394
DmelGr85a       --------------------------------------------------------VNLE 343
TcasGr27        -----------------------------------------------------------K 291
TcasGr48        ------------------------------------------------------ADSFCK 318
AgamGr1         -----------------------------------------------------TQNVECE 334
DmelGr10a       ----------------------------------------------------REMDERLT 354
DmelGr59e       ---------------------------------------------------------RLQ 331
DmelGr10b       -------------------------------------------------------SRTTY 319
DmelGr89a       --------------------------------------------------------LTQE 303
DmelGr57a       ------------------------------------------------------DWFPQK 342
DmelGr47b       ---------------------------------------------------------VAR 336
DmelGr58a       --------------------------------------------------HLELVKRDFH 338
DmelGr58b       -----------------------------------------------------KVGPKMQ 336
DmelGr98a       ---------------------------------------------------------QLN 319
AaegGr93a       -----------------------------------------------------IVCLDSR 345
TcasGr19        -----------------------------------------------------------T 310
DmelGr47a       ---------------------------------------------------------RLD 307
DmelGr58c       ----------------------------------------------CIKSSGTILSRKLE 356


TcasGr38        DEIEMFSLQIANEQ---VEFNAAGFFPINYTLVFS------------------------- 389
TcasGr46        DKIEMFSLQILNER---AGFSAAGFFPIDYSLVFS------------------------- 710
TcasGr1         DKIEMFSLQILNER---AEFNAAGFFPIDYTLVFS------------------------- 369
TcasGr123       DEIEMFSLQIANEQ---VEFNAAGFFAINYTLVFS------------------------- 362
TcasGr62        DEIEMFSLQIANEQ---VEFNAAGFFAINYTLVFS------------------------- 637
TcasGr25        IRPGDTPLEDINPD---LAEAFSSYARKNARLSFG------------------------- 261
TcasGr59        IRPGDTPLEDINPD---LAEAFSSYARKNARLSFG------------------------- 436
AgamGr2         EVVNHLSLKLLNHQ---LNFSACGFFDLDMTTLYA------------------------- 390
```

```
AaegGr66a       QIVNHLSLKLLNHQ---LNFSACGFFDLDMTTLYA------------------------- 452
DmelGr66a       EIVNHLSLKLLNHS---VDFSACGFFTLDMETLYG------------------------- 466
TcasGr11        NN---------------------------------------------------------- 339
TcasGr55        NNVQSYSLQLLHQK---VQFSVLGFFVVDYTLLYS------------------------- 369
TcasGr21        NSTEFDNATESY------------------------------------------------ 386
TcasGr56        ------------------------------------------------------------
AaegGr          KQLTNLSLQMTHRK---VAFTAYGFFKLDFTLLFT------------------------- 408
AaegGr28a       ------------------------------------------------------------
AgamGr33        GKLMHLSLQMLHRK---VRFSACGLFSLDFSLIFT------------------------- 406
DmelGr28bB      EKLQQFSMQLMHLK---INFTAAGLFNIDRTLYFT------------------------- 401
DmelGr28bC      EKLQQFSMQLMHLK---INFTAAGLFNIDRTLYFT------------------------- 428
DmelGr28bD      EKLQQFSMQLMHLK---INFTAAGLFNIDRTLYFT------------------------- 398
DmelGr28bA      EKLQQFSMQLMHLK---INFTAAGLFNIDRTLYFT------------------------- 410
DmelGr28bE      EKLQQFSMQLMHLK---INFTAAGLFNIDRTLYFT------------------------- 405
AgamGr25        ESINMFWQQLIASN---FSFTACGLCTIDRRIITS------------------------- 429
AaegGr43a       ------------------------------------------------------------
DmelGr43a       EAVKKFWQQLLVVD---ADFSACGLCRVNRTILTS------------------------- 407
AgamGr37b       -QLNLFSQQLLHRS---PVIT-CGLFVYDWTLWYT------------------------- 344
AgamGr37d       -QLNLFSQQLLHRS---PVIT-CGLFVYDWTLWYT------------------------- 340
AgamGr37a       -QLNLFSQQLLHRS---PVIT-CGLFVYDWTLWYT------------------------- 354
AgamGr37c       -QLNLFSQQLLHRS---PVIT-CGLFVYDWTLWYT------------------------- 348
AgamGr37e       -QLNLFSQQLLHRS---PVIT-CGLFVYDWTLWYT------------------------- 348
AgamGr37f       -QLNLFSQQLLHRS---PVIT-CGLFVYDWTLWYT------------------------- 375
AaegGr28c       NELNIFSQQLLHRS---PVIT-CGLFVYDWTLLYT------------------------- 538
AaegGr28b       DELMLFSSFLAHKK---IVVN-CYIFVCDWTLGMS------------------------- 387
AaegGr28e       DELMLFSSFLAHKK---IVVN-CYIFVCDWTLGMS------------------------- 387
TcasGr14        RHLQLVAIRLLNTK---LQFTAKDFFNLDWTFCHM------------------------- 357
TcasGr49        RHLQLVAIRLLNTK---LQFTAKDFFNLDWTFCHM------------------------- 401
AmelGr4         KELKQFSFQLLHQK---IIFSTNGYFTLDNKFFQS------------------------- 382
AmelGr5         IELEQFSLQLLHQK---VKFTANGYFTLDNTLFQS------------------------- 381
TcasGr16        NEVISSALKLSQNE---LEITACKFFSIDNALLFSANSTKKLIVKIRIDIDKEDERNEII 394
TcasGr22        NVVVTSVLKLMQNK---LEITACRLFSIDNALLFS------------------------- 272
TcasGr17        NS------HCLGGT---ITRTMW---------YFQ------------------------- 341
TcasGr150       NSVVASVLQLMENK---IEITACRLFNIDNALLFA------------------------- 356
TcasGr32        QDLISTIKDN---F---PRFYAARFFVINRGTILG------------------------- 334
TcasGr54        QDLISTIKDN---F---PRFYAARFFVINRGTILG------------------------- 334
TcasGr37        NDFVTALKDN---V---PVFAAARFYAINRSTIFR------------------------- 309
TcasGr53        NDFVTALKDN---V---PVFAAARFYAINRSTIFR------------------------- 309
TcasGr5         KMFVKIVDAS---C---RNFTAARFFELNRRTILG------------------------- 321
TcasGr12        DELIVTLSTHY--F---PVFSACDYFTIETKTVLD------------------------- 294
TcasGr13        DELIVTLSAHY--F---PVFSACDYFTIETKTVLD------------------------- 368
TcasGr104       TELFKLAQHVSANI---VHITAGNYFEINKATLCG------------------------- 358
TcasGr45        TELFKLAQHVSANI---VHITAGNYFEINKATLCG------------------------- 358
TcasGr98        QELYMAAQVIEKSV---ANFTAAGFFDVKRSTLFG------------------------- 355
TcasGr52        KELLRFASESRGSV---AKFTAADFFEIDRGTFFG------------------------- 292
TcasGr105       RELLRLAQQVNANI---AQITAANFFDISRSTFLG------------------------- 338
TcasGr4         --------EVQSRS---NRMTPLKLQPQNFSI---------------------------- 309
TcasGr44        --------EVQSRS---NRMTPLKLQPQNFSI---------------------------- 313
AgamGr9a        KQIQNFLLKNLHQK---KKFSACGFFDIDNTVIYM------------------------- 357
AgamGr9c        KQIQNFLLKNLHQK---KKFSACGFFDIDNTVIYM------------------------- 356
AgamGr9b        KQIQNFLLKNLHQK---KKFSACGFFDIDNTVIYM------------------------- 407
AgamGr9d        KQIQNFLLKNLHQK---KKFSACGFFDIDNTVIYM------------------------- 363
AgamGr9e        KQIQNFLLKNLHQK---KKFSACGFFDIDNTVIYM------------------------- 359
AgamGr9f        KQIQNFLLKNLHQK---KKFSACGFFDIDNTVIYM------------------------- 356
AgamGr9g        KQIQNFLLKNLHQK---KKFSACGFFDIDNTVIYM------------------------- 359
AgamGr9i        KQIQNFLLKNLHQK---KKFSACGFFDIDNTVIYM------------------------- 360
AgamGr9h        KQIQNFLLKNLHQK---KKFSACGFFDIDNTVIYM------------------------- 365
AgamGr9j        KQIQNFLLKNLHQK---KKFSACGFFDIDNTVIYM------------------------- 396
AgamGr9l        KQIQNFLLKNLHQK---KKFSACGFFDIDNTVIYM------------------------- 386
AgamGr9k        KQIQNFLLKNLHQK---KKFSACGFFDIDNTVIYM------------------------- 378
AgamGr9m        KQIQNFLLKNLHQK---KKFSACGFFDIDNTVIYM------------------------- 381
AgamGr9n        KQIQNFLLKNLHQK---KKFSACGFFDIDNTVIYM------------------------- 348
AgamGr10        KQIQKFLLKNLHQK---KKFSACGFFDIDNTVIYM------------------------- 348
AgamGr11        TQIHRFLLQNLNRK---INFSVCGMFDIDYAMIHM------------------------- 353
DmelGr23aA      DLVSEFSLQTLHQR---FVVTAKDFFSLNLHLLSS------------------------- 343
DmelGr23aB      DLVSEFSLQTLHQR---FVVTAKDFFSLNLHLLSS------------------------- 347
DmelGr39aA      RMIEKFLLKNLRQK---PILTAYGFFALDKSTLFK------------------------- 344
DmelGr39aB      RMIEKFLLKNLRQK---PILTAYGFFALDKSTLFK------------------------- 353
DmelGr39aC      RMIEKFLLKNLRQK---PILTAYGFFALDKSTLFK------------------------- 353
DmelGr39aD      RMIEKFLLKNLRQK---PILTAYGFFALDKSTLFK------------------------- 343
```

```
DmelGr98c      MGLKEYILQMQHLK---LLFTCGGLFDINIKLFGG------------------------- 375
DmelGr98d      MGLREYSLQMEHLK---LIFTCGGLFDINLKFFGG------------------------- 373
DmelGr98b      RGLREYSLQMEHLK---LRFTCGGLFDINLKYFGG------------------------- 373
AgamGr51       RLIEVLMVDCMQRN---NAINNYGMYAMNRALLFG------------------------- 388
AgamGr52       RMLDFITLDYMQRD---YDVRNYGLYDINRALLFG------------------------- 383
DmelGr94a      QAVHGFTLLVTVTK---YQIKPLGLYELDMRLISN------------------------- 380
DmelGr97a      QVVNAFWLQVVTIN---YKLMPLGLLELNTSLVNK------------------------- 401
DmelGr93b      QKLELFLGRLQHQE---LRVFPLGLFEVSNELTLF------------------------- 379
DmelGr93c      MKWEMFLSRLNFFE---FRVRPLGLFEVSNEVILL------------------------- 375
DmelGr22d      ISISDFALYCSHRR---FKFLHCGLFHVNREMGFK------------------------- 367
DmelGr22e      RSITDFALFCSHRR---LRFHHCGLFYVNYEMGFR------------------------- 369
DmelGr22a      RRVAEFTLFCSHRR---LKVCHLGLLDINYEMGFR------------------------- 369
DmelGr22b      MSVNEFAWLCSHRK---FRFQLCGLFSMNCRMGFK------------------------- 366
DmelGr22c      RSVNEFALLCGHCQ---FNFHVCGLFTINYKMGFQ------------------------- 363
DmelGr22f      RSLNEFAWLCTHRK---FRFQLCGLFSINHNMGFQ------------------------- 358
DmelGr36a      QSFESIQLQLIRNP---LKIEVLDIFTITRSSSAA------------------------- 371
DmelGr36c      EAFENLNLQLIRNP---LKITVVKLYDVTRSNTMA------------------------- 370
DmelGr36b      ESFESLQLQLARNP---LKINVMGMFPITRGSTAA------------------------- 371
DmelGr59d      MVFENFALNLVRNP---LKLHMYGLFEFGRGTSFA------------------------- 370
DmelGr59a      NELSSYLIYESSTR---LDLLVCGLYRVNKRKWLQ------------------------- 344
DmelGr59b      KEISSYVIYANSTK---LQLWSCGLFQANRSMWFA------------------------- 345
AgamGr26       AKFSSQLLHVQGPQ------TACGVINLEMTLIST------------------------- 396
AgamGr27       MKFAGQLLLLHGKR---HHCKACGLITLDLTLISK------------------------- 383
AgamGr29       RFITQTTLFLEKAH------EAYGMISIDMTLILS------------------------- 392
AgamGr31       RFISQTTLFLANGH------EAYSMISIDMTLILS------------------------- 393
AgamGr30       YFMQVS--NLQDTH------QACGMIKLDMQLVPN------------------------- 382
AgamGr32a      ------------------------------------------------------------
TcasGr2        LEIQRFIQQIGNSD---IAITGKNFFSITRGLILSFVDYFVFLLCSILVNIAFIKLATEW 418
TcasGr9        KEISRLVETL-------------KFQSITS------------------------------ 379
TcasGr7        LEIQRFIHQIGTLE---VAFTGKNFFSITRGLILSIAG---------------------- 398
TcasGr30       ---QRFIHQIGTLE---VAFTGKNFFSITRGLILSIAG---------------------- 363
TcasGr29       LEIQRFIHQIGTLE---VAFTGKNFFSITRGLILSIAG---------------------- 398
AaegGr64f      LDIQRLLDHASLKS---IAFSGKRFFFITRPLILAMAG---------------------- 355
AaegGr64a3     ------------------------------------------------------------
AgamGr14       LDLRRLCDAVATSE---NALSGKRFFFVRRPLILA------------------------- 416
AgamGr18       IDLQRLTDEVASGE---NVLSGKQFFFLKRQLILAMAG---------------------- 391
AaegGr64d      LDLQRLTDDVASGE---NTLSGKKFFYLKRQIILAMAG---------------------- 423
AgamGr17       IDVQRFTQELLSGR---NCLSGHGFFFLNRSVILAMAG---------------------- 380
AaegGr64c      TSVQRFTNEILNIE---NALSGHKFFFLKRGIILAMAG---------------------- 405
DmelGr64c      VELRRLNEIFLSDH---FALSGKGYFLLTRRLIFAMAA---------------------- 384
DmelGr64d      DELGRLSEMLRNET---FALSGMGYFYVTRRLIFAMAG---------------------- 393
AgamGr20       DELEILRICSSGG----VSISGMGFFTITRRIFLTMAG---------------------- 410
AaegGr61a      DELQRVQMYSRRG----VSLTGMGVFLVSRRIFLTIAG---------------------- 407
AgamGr21       VELERFSTQLKSEK---VALSGMGFFSLTRQLLFSMAG---------------------- 436
AaegGr64a      VELDRFSSQLKSET---VALSGMGFFHITRQLLFSMAG---------------------- 319
DmelGr64a      VEVERLIFQMTTQT---VALSGKKFYFLTRRLLFGMAG---------------------- 430
DmelGr61a      QEVQRFADQLTSEF---VGLSGYRLFCLTRKSLFGMLA---------------------- 413
AgamGr15       LEAKRFAEEVTTDT---VALTGLKFFSMTRQLVLNVTG---------------------- 417
AaegGr1        VEAKRFAEEVINDT---VALTGMKFFNMTRKLVLKVTG---------------------- 421
DmelGr5a       PEVFRFAAELASDQ---VALTGLKFFNVTRKLFLAMAG---------------------- 417
DmelGr64f      PEVKRFTEEVISDE---VALTGMKFFHLTRKLVLSVAG---------------------- 442
DmelGr64e      DELKRFSEEVQMDN---VALTGMKFFRLTRGVVISVAG---------------------- 436
AmelGr1        TEVMRFLSQVTTDN---ICLTGMKFFSVTRSLVLTVAG---------------------- 365
AmelGr2        VEVQRLQCQLATDD---IALTGLRFFSITRNFMLAVAG---------------------- 455
AgamGr16       DEVQRFHRQVEHDS---VALNGYGFFYLTRKLILKIAA---------------------- 387
AaegGr64e      KEVQRFHQQMENET---VALSGFRFFHLTRKLILKISG---------------------- 408
TCasGr6        KEIDIFLVAIQMNP---PKVSLKGYTVVNRELVTASVA---------------------- 366
TcasGr26       KEIDIFLVAIQMNP---PKVSLKGYTVVNRELVTASVA---------------------- 366
TcasGr33       KEIDIFLVAIQMNP---PKVSLKGYTVVNRELVTASVA---------------------- 366
AgamGr23       KEIDLFIQAIEMNP---AIVSLKGYAEVNRELLTSSIA---------------------- 450
AaegGr21a      KEIDLFIQAIEMNP---AIVSLKGYAEVNRELLTASIA---------------------- 442
TcasGr10       QEVHMFLMAIEKNP---PIMNLNGYANVNRKLISSTVT---------------------- 396
TcasGr39       QEVHMFLMAIEKNP---PIMNLNGYANVNRKLISSTVT---------------------- 396
AgamGr22       KEVEMFLVAIDKNP---PTMNLDGYANINRGLITSNIS---------------------- 415
AaegGr21b      KEVEMFLVAIDKNP---PTMNLDGYANINRGLITSNIS---------------------- 421
DmelGr21a      KEVEMLLVAINKNP---PIMNLDGYANINRELITTNIS---------------------- 422
AgamGr24       TEINMFLRATEMNP---SSINLGGFFDVNRTLFKSLLA---------------------- 346
AaegGr63a      TEINMFLRATEMNP---SSINLGGFFDVNRTLFKSLLA---------------------- 411
DmelGr63a      TEINMFLRATEMNP---STINCGGFFDVNRTLFKGLLT---------------------- 440
TcasGr3        QEINMFLKATEMSP---TDISLVGFFDVNRNLFKSLLA---------------------- 402
```

```
DmelOr83b       KTFVQIVCQQCQKA---MSISGAKFFTVSLDLFASVLG---------------------- 473
AgamOr7         KTFVQIVCQQCQKA---MTISGAKFFTVSLDLFASVLG---------------------- 465
HvirOr2         KTFVQIVCQQCQKA---MSISGAKFFTVSLDLFASVLG---------------------- 459
TcasOr16        KTFVQIVCQQCQKA---MSISGAKFFTISLDLFASVLG---------------------- 561
TcasOr22        KTFVQIVCQQCQKA---MSISGAKFFTISLDLFASVLG---------------------- 600
AmelOr2         KTFVQIVCQQCQKA---MSISGAKFFTVSLDLFASVLG---------------------- 465
TcasGr31        -KVLPLLFSVSSSS---YNCEVLFSFLIDQF----------------------------- 250
TcasGr35        DEVQIFIDVVQHNR---PEFKAARFFSIDRSTLFS------------------------- 233
DmelGr59c       AAFENFQLQLASNR---HEFYVMGLFKMERGRLIA------------------------- 375
DmelGr92a       TTLDTLFLHLRLGH---FRVSILGLFDVTQMQYLQ------------------------- 364
DmelGr28a       DRLFRLSLQLSHRK---VLFTAAGLFRLDRTLIFT------------------------- 411
DmelGr64b       IEVQRFSEQLGNDT---TALSGSGFFYLTRSLVLAMG----------------------- 377
TcasGr71        KNISEFINVVLNNY---PDFSAAGFFSINKTTLLQ------------------------- 328
AgamGr19        SELERFYSFIRKSS---IAINAMGLFRLTKKTMLTMLG---------------------- 373
AaegGr64b       TELERYYLQLRAEV---GALSGSRFFYLTHQTTFT------------------------- 318
DmelGr93d       KSVEIFVTHLNLSE---FRVNLLGLFNVSNELFLIIVS---------------------- 359
TcasGr34        LRHKFDLKTLVLNF---PKFTAGGFFHVKKSTIFS------------------------- 305
TcasGr43        QELYEFSNFVSQHL---PKFSAANFFDIERSTILS------------------------- 335
TcasGr47        NKLGDFVALIRDAQ---VKITAADFFILDKSTILK------------------------- 304
AmelGr3         RELEIFSLQLLHRP---LEFSACGLFSLDRNLITS------------------------- 424
AgamGr32b       EAIEHFISQISNLH---DVHQACGMLNLDMKLISN------------------------- 396
AgamGr50        RAIELLTIEYLNRD---YAIRIKGLFTIDNTMLFG------------------------- 385
TcasGr20        EQVEILAVQVSNKK---IKFSSFGMLKISRSLLTS------------------------- 370
TcasGr79        CCEKCGLKKIEFCR---PTFSAARFYGIDFSNTFLG------------------------ 330
TcasGr15        EDYDRLARLTQFLD---KELSYLILFSIGFNCFWVMKLLYNVLRFG-------------- 383
TcasGr60        FEITALRLFTIDNK---LLFGVLAYYSTTCDIATILDFWVR------------------- 328
AgamGr38        LTLLRMFLMQLGHR---SPMITCGLFPFDWTLIFS------------------------- 405
TcasGr51        DELIKFISIAKHNV---RTFSAGGFFDIRKNLIFS------------------------- 750
DmelGr39b       DLLMEFILQVEQNV---LAINAEGFMSTDNSLLMS------------------------- 351
AgamGr28        QMALLRLLTLGRKR---IVPTACGLLKLELSSLSS------------------------- 361
TcasGr61        EKLQIFALQLTHRA---PTFTALGLFPINGSFAFT------------------------- 646
TcasGr50        --VNKLIFRSNDPK---VTLELCGMISTYLIIMIQ------------------------- 331
AgamGr12        VDRFLLQKLSQTNQ---QRFMAGGLFVLDNKLVCT------------------------- 358
AgamGr36        RKLIWFSQQILFRR---PKLR-CIFYAYDWKTIYN------------------------- 374
AgamGr48        RSIELFTLAVLTTD---ARIKIGGMYVLNMAFVFS------------------------- 381
AgamGr49a       RCIESFAIELLHED---FKINNCGMYDVDYTLMFS------------------------- 381
AgamGr49b       RCIESFAIELLHED---FKINNCGMYDVDYTLMFS------------------------- 385
DmelGr2a        ALITQFSLQLLHQR---LHFSAAGFFNVDCTLLYT------------------------- 385
DmelGr32a       NIIDKFLTKSIKQE---VQFTAYGFFAIDNSTLFK------------------------- 430
DmelGr68a       NLIDKFLTKSIKQD---LQFTAYGFFSIDNSTLFK------------------------- 360
AmelGr7         AEIRDFTLQLIQNP---VVFTAYGFFNLDHSFIQG------------------------- 383
AmelGr8         TELELFSLEVLQKD---NKFIMFG-LEMDLTLVTD------------------------- 374
AmelGr9         REVHLFSLQIMHRN---NKFTAKTFDMNSVLLGQ-------------------------- 368
TcasGr57        EELALLAQQSTSRT---SKFSAAGFFPVDFTLLGF------------------------- 370
TcasGr41        KELLLFAEQMGQRQ---VAFKVGGFLNIDYGTLYS------------------------- 376
AgamGr34        VIKLTVPFQAPNKV---FKFSAMGFFQIDCNMLCG------------------------- 395
AgamGr35        AQHSSIWFPNLISS---VRFSAFGFFTINYNMLSG------------------------- 412
AgamGr39        NDCGSQIIEQLSHG---IPKASCYFYDIDWTFLLS------------------------- 390
AgamGr40        LLQIKFLSQQLRSV---APRVSCLLFDFEWPFLVS------------------------- 369
AaegGr28d       KELGTFSYQLYHHT---PKVS-CRLFDLDWTLLYT------------------------- 375
AgamGr42        VERLVLFSHHLQHQ---RPVVSCGLFCFDWTLALS------------------------- 396
AgamGr41        EDLLQQFSEQMNHR---SPSVDFRFFDVDWPLLVK------------------------- 418
AgamGr43        SKAIALQLLHRKRF---SLFDGSGLFRLDFTFIFS------------------------- 400
DmelGr33a       NKMKSFTLQFLHWEG-FFQFNGVGLFALDYTFIFS------------------------- 447
AgamGr44        QSIEVFALQTLHQP---IEFTACRMFTLDYTVLFS------------------------- 376
AgamGr13        ATINTFLLSLNHSM---LKINIAGMFTMDFELLTG------------------------- 379
AgamGr45        INFRTVTLALLFST--FIFYSHSNTLFIDFSRTTS------------------------- 412
AgamGr46        DHVRLLSLQLHQQR---IEFTASGLFTIDHGLMFN------------------------- 402
Dmelr93a        KSVETFLGQLQTQR---LEIKVLGFFHLNNEFILL------------------------- 387
AmelGr6         TKQDSFRTSLYLLNR-RLRFSAGGLFDIKLSLLCS------------------------- 421
AmelGr10        SPYSSVSLKRVHLQ---ENIEAAAYFQLRKVHLFT------------------------- 359
TcasGr28        ------------------------------------------------------------
TcasGr40        EHILSIVNTCSQIPS-EVEDKYTTYFLNTYPHLFN------------------------- 359
AgamGr3         KLRFYFLHQLLRNR---IKLTAKDIFDYDYTLIRT------------------------- 357
AgamGr4         KIIYRFGLQIAQQR---IRLTAHGLFEINYSLLKMFG----------------------- 397
AgamGr6         TLIENFSKQLDNEQ---INFSAANFFDIRLSSLTT------------------------- 389
AgamGr7         KMLENFSLLLQHTP---MSFSLGGFFDMDFVLLKE------------------------- 398
AgamGr47        EKLSTLSLQVTMRD----MSFSCGLFNFDWNLFGS------------------------- 380
DmelGr8a        LQIQNFSLQLLHQP---IRIDCLGLTILDCSLLTR------------------------- 361
AgamGr5         TRIESFLQQTQFES---IQLTAGDFFDLNFSPILT------------------------- 409
```

```
AgamGr8        QMVQHFLLKLESTQP--FTMTALSMYRLDYGTLMQ------------------------- 350
DmelGr9a       SQIEGFALQIMQDP---IQIDVCGIYHLNLQTLAG------------------------- 320
DmelGr59f      DTITHFIIQMRTNVR---QHVVCGVINLDLKFLTT------------------------- 372
DmelGr77a      HTMHFYGLYLKNVEH-VFAVSACGLFKLNNAILFC------------------------- 428
DmelGr85a      RSIEWLSLQLTWQH---THVTIFGVFRINRSLAFR------------------------- 375
TcasGr27       QIQNEVFIFMGQLEK-PIVFYVANIFSLDLDTFKK------------------------- 325
TcasGr48       KKLENYSLQLINHR---VVFTAMGLYVLNMEHFFSVKCSIQSE----------------- 358
AgamGr1        QMITSFSLYVAEQK---PKFRILKSINLSFNLIYAS------------------------ 367
DmelGr10a      REIEHLSLELLNYQ----PPMLCGLLHLDRRLVYL------------------------- 385
DmelGr59e      RTINRFLLQLQRSID--QPLEACGIVTLDTRSLGG------------------------- 364
DmelGr10b      RQWEMSALRRAITRS-SPENNVLGMFRMDMRCAFA------------------------- 353
DmelGr89a      QAMASTPFPVLTPTG-NVKFRILGVFILDNSFWLF------------------------- 337
DmelGr57a      SSLRPLVESLMSWRI-QAKIQFTSGLDVVLSRKVIG------------------------ 377
DmelGr47b      RKTEDFQHWRMHNRQ-AAMVGSTTLLSVSTIYLVYN------------------------ 371
DmelGr58a      LQLELFSDHLRCHP---STYKVCGLFIFNKQTSLA------------------------- 370
DmelGr58b      RELDVFTMQLRQNR---LVYKICGIVELDKPACLS------------------------- 368
DmelGr98a      AALRSLNLQLSQEK---YKFSAGGMVDINTEMLGK------------------------- 351
AaegGr93a      LTESIYGLSKVVQCM-QEETVMFYCFALNNSFLTT------------------------- 379
TcasGr19       HFYCAFSLYPLKST---FYLNVVRPFPERSMGITP------------------------- 342
DmelGr47a      RLLSMFALKLALHP---KRVVLLNVFTFDRKLTLT------------------------- 339
DmelGr58c      FSLLYMNRKLQLNPKRVRRLHIVGLFDLSNLTVHN------------------------- 391


TcasGr38       ----------------------------VRSVQVERI----------------------- 398
TcasGr46       ----------------------------ETKLLLFKITSVMIAVSGLVYIATVWICTLMN 742
TcasGr1        ----------------------------VSFG---------------------------- 373
TcasGr123      ----------------------------ILGGVTTYIIILIQLATSLAGQ---------- 384
TcasGr62       ----------------------------ILGGVTTYIIILIQLATSLAGQ---------- 659
TcasGr25       ----------------------------RAEPVFDGKVPSNGSLNQISDIDDVNFQEENL 293
TcasGr59       ----------------------------RAEPVFDGKVPSNGSLNQISDIDDVNFQEENL 468
AgamGr2        ----------------------------ITGAITSYLIILIQFNLAAIQKSNSNSTIASN 422
AaegGr66a      ----------------------------ITGAITSYLIILIQFNLAALQKAG-NATEAVN 483
DmelGr66a      ----------------------------VSGGITSYLIILIQFNLAAQQAKEAIQTFNSL 498
TcasGr11       -------------------------------------VIIIL------------------ 344
TcasGr55       ----------------------------IVGAVTTYLVIFIQFDQSSNSRNNYVLTNNST 401
TcasGr21       ------------------------------------------------------------
TcasGr56       ------------------------------------------------------------
AaegGr         ----------------------------LVGAATTYLVILVQFSLNQSQPCNQSMINYVT 440
AaegGr28a      ------------------------------------------------------------
AgamGr33       ----------------------------LVGAATTYLVILIQYELSMDESKHETIARAFL 438
DmelGr28bB     ----------------------------ISGALTTYLIILLQFTSNSPNNGYGNGSSCCE 433
DmelGr28bC     ----------------------------ISGALTTYLIILLQFTSNSPNNGYGNGSSCCE 460
DmelGr28bD     ----------------------------ISGALTTYLIILLQFTSNSPNNGYGNGSSCCE 430
DmelGr28bA     ----------------------------ISGALTTYLIILLQFTSNSPNNGYGNGSSCCE 442
DmelGr28bE     ----------------------------ISGALTTYLIILLQFTSNSPNNGYGNGSSCCE 437
AgamGr25       ----------------------------YCGAIATYLVILIQFKEADDNSGKTPH----- 456
AaegGr43a      ------------------------------------------------------------
DmelGr43a      ----------------------------FASAIATYLVILIQFQRTNG------------ 427
AgamGr37b      ----------------------------MIGATATYLIILIQFDVSFPNLVNVNATAVYR 376
AgamGr37d      ----------------------------MIGATATYLIILIQFDVSFPNLVNVNATAVYR 372
AgamGr37a      ----------------------------MIGATATYLIILIQFDVSFPNLVNVNATAVYR 386
AgamGr37c      ----------------------------MIGATATYLIILIQFDVSFPNLVNVNATAVYR 380
AgamGr37e      ----------------------------MIGATATYLIILIQFDVSFPNLVNVNATAVYR 380
AgamGr37f      ----------------------------MIGATATYLIILIQFDVSFPNLVNVNATAVYR 407
AaegGr28c      ----------------------------MIGATATYLIILIQFDVSFPNLVNVNGTQTAA 570
AaegGr28b      ----------------------------LISAIISYLVILIQFDASLVTTIPKNDTNSLL 419
AaegGr28e      ----------------------------LISAIISYLVILIQFDASLVTTIPKNDTNSLL 419
TcasGr14       ----------------------------MIAALTTYLVILIQFNF--------------- 374
TcasGr49       ----------------------------VSHKWLNYIN-QFEANFR-------------- 418
AmelGr4        ----------------------------MMSTMITYLVILLQFQTQTLNENVNSCNCMEC 414
AmelGr5        ----------------------------MINTVTTYMVILFQFQMEISNENDKFCNCTQC 413
TcasGr16       SSALKLSQSELEITACKFFSIDNALLLSICGASSSYLFIMIQLDLGNKKNETSTILVE-- 452
TcasGr22       ----------------------------ICGAASSYLFIMLQLDIGSKTQGTNSTLY--- 301
TcasGr17       ----------------------------ICGSAFSYLFIMLQFDLNNKKNQSLGNGTTA- 372
TcasGr150      ----------------------------ICGSAFSYLFIMLQFDLNNKKNQSLGNGTTA- 387
TcasGr32       ----------------------------ILDAIVTFLIVMIQFEMTQNSTNINFVSTFFK 366
TcasGr54       ----------------------------ILDAIVTFLIVMIQFEMTQNSTNINL------ 360
TcasGr37       ----------------------------MFNAIVTFLIVMVQFETNYSQP---------- 331
TcasGr53       ----------------------------MFNAIVTFLIVMVQFETNYSQP---------- 331
TcasGr5        ----------------------------VSNAVITFLIVMVQKASYEKLS---------- 343
```

```
TcasGr12        ----------------------------VLGVTVTYLIVIIQFDDKIVQV---------- 316
TcasGr13        ----------------------------VLGVTVTYLIVIIQFDDKIVQV---------- 390
TcasGr104       ----------------------------IFGTTTTYFIVILQFNQSSAK----------- 379
TcasGr45        ----------------------------IFGTTTTYFIVILQFNQSSAK----------- 379
TcasGr98        ----------------------------ILATTTTYLIVTIQFNQGLNK----------- 376
TcasGr52        ----------------------------ILSTTTSYFIIMIQFNMSL------------- 311
TcasGr105       ----------------------------ILATITTYFIVIIEFGF--------------- 355
TcasGr4         ------------------------------------------------------------
TcasGr44        ------------------------------------------------------------
AgamGr9a        ----------------------------VFSSIVTYLVILIQFKQLETD-LTQAGDGYNV 388
AgamGr9c        ----------------------------VFSSIVTYLVILIQFKQLETD-LTQAGDGYNV 387
AgamGr9b        ----------------------------VFSSIVTYLVILIQFKQLETD-LTQAGDGYNV 438
AgamGr9d        ----------------------------VFSSIVTYLVILIQFKQLETD-LTQAGDGYNV 394
AgamGr9e        ----------------------------VFSSIVTYLVILIQFKQLETD-LTQAGDGYNV 390
AgamGr9f        ----------------------------VFSSIVTYLVILIQFKQLETD-LTQAGDGYNV 387
AgamGr9g        ----------------------------VFSSIVTYLVILIQFKQLETD-LTQAGDGYNV 390
AgamGr9i        ----------------------------VFSSIVTYLVILIQFKQLETD-LTQAGDGYNV 391
AgamGr9h        ----------------------------VFSSIVTYLVILIQFKQLETD-LTQAGDGYNV 396
AgamGr9j        ----------------------------VFSSIVTYLVILIQFKQLETD-LTQAGDGYNV 427
AgamGr9l        ----------------------------VFSSIVTYLVILIQFKQLETD-LTQAGDGYNV 417
AgamGr9k        ----------------------------VFSSIVTYLVILIQFKQLETD-LTQAGDGYNV 409
AgamGr9m        ----------------------------VFSSIVTYLVILIQFKQLETD-LTQAGDGYNV 412
AgamGr9n        ----------------------------VFSSIVTYLVILIQFKQLETD-LTQAGDGYNV 379
AgamGr10        ----------------------------VFSSIVTYLVILIQFKQLETD-LTQSGDGYNV 379
AgamGr11        ----------------------------VLSSIFTYLVILVQFDQLQPERLSYLNTNLNT 385
DmelGr23aA      ----------------------------MFAAVVTYLVILIQFMFAERS--STRGSG--- 370
DmelGr23aB      ----------------------------MFAAVVTYLVILIQFMFAERS--STRGSG--- 374
DmelGr39aA      ----------------------------LFTAIFTYMVILVQFKEMENS--TKSINKF-- 372
DmelGr39aB      ----------------------------LFTAIFTYMVILVQFKEMENS--TKSINKF-- 381
DmelGr39aC      ----------------------------LFTAIFTYMVILVQFKEMENS--TKSINKF-- 381
DmelGr39aD      ----------------------------LFTAIFTYMVILVQFKEMENS--TKSINKF-- 371
DmelGr98c       ----------------------------MLVTLCGYVIIIVQFKIQDFA--LIGYRQNTS 405
DmelGr98d       ----------------------------MVVTLFGYIIILVQFKIQFFA--QSNFMQNIN 403
DmelGr98b       ----------------------------LLVTIFGYIIILIQFKVQAIA--ANRYKKVVN 403
AgamGr51        ----------------------------MIATMTSYLIILIQFHIQAYE----------- 409
AgamGr52        ----------------------------TIATVTSYVIILVQFHMQQYG----------- 404
DmelGr94a       ----------------------------VFSAVASFLLILVQADLSQRFKMQ-------- 404
DmelGr97a       ----------------------------VFSSAIGSLLILIQSDLTLRFSLK-------- 425
DmelGr93b       ----------------------------FLSAMVTYLVFLVQYGMQSQQI---------- 401
DmelGr93c       ----------------------------FLSSMITYFTYVVQYGIQTNRL---------- 397
DmelGr22d       ----------------------------MFVASVLYLLYLVQFDYMNL------------ 387
DmelGr22e       ----------------------------MAITSFLYLLFLIQFDYWNL------------ 389
DmelGr22a       ----------------------------MIITNILYVVFLVQFDYMNLKFKTD------- 394
DmelGr22b       ----------------------------MIITTFLYLVYLVQFDYMNL------------ 386
DmelGr22c       ----------------------------MIITSFLYLIYMIQFDFMNL------------ 383
DmelGr22f       ----------------------------MIITSFLYLVYLLQFDFMNL------------ 378
DmelGr36a       ----------------------------MIGSIITNSIFLIQYDMEYF------------ 391
DmelGr36c       ----------------------------MFGNLITHSIFLIQYDIEHF------------ 390
DmelGr36b       ----------------------------MCASVIVNSIFLIQFDMEFF------------ 391
DmelGr59d       ----------------------------VFNSLLTHSLLLIQYDVQNF------------ 390
DmelGr59a       ----------------------------MVGSIVVHSSMLFQFHLVMRGGL--------- 367
DmelGr59b       ----------------------------MISSVLYYILVLLQFHLVMRK----------- 366
AgamGr26        ----------------------------MVGALTTYLVILIQFDTAITQGQTADSNGPSS 428
AgamGr27        ----------------------------IVAGLTTYLMILIQFDSAFTQGSR-------- 407
AgamGr29        ----------------------------NVGGLTNILVVLVQFADAKPSPN--------- 415
AgamGr31        ----------------------------IVAGLTNILVVLVQFSDGKPSCN--------- 416
AgamGr30        ----------------------------VIAVITSILFILMQFSDADRNQDIKTVYRLDN 414
AgamGr32a       ------------------------------------------------------------
TcasGr2         PQ--------------------------LMKAWLKIELLVGNLGMRRNFRKKLDFIFTFI 452
TcasGr9         -------------------------------AVVTYELIV------IQFRKKME------ 396
TcasGr7         -------------------------------AIVSYELVL------MQFNDSLL------ 415
TcasGr30        -------------------------------AIVSYELVL------MQFNDSLL------ 380
TcasGr29        -------------------------------AIVSYELVL------MQFNDSLL------ 415
AaegGr64f       -------------------------------TIITYELVL------LDQVAKEQ------ 372
AaegGr64a3      ------------------------------------------------------------
AgamGr14        ------------------------------------------------------------
AgamGr18        -------------------------------TLVTYELVL------LDQVKKIP------ 408
AaegGr64d       -------------------------------TLVTYELVL------MDQVKQAP------ 440
AgamGr17        -------------------------------TLITYELVM------LKEVNPSS------ 397
AaegGr64c       -------------------------------TMITYELVM------LSEVR-HS------ 421
```

```
DmelGr64c       -------------------------------TLMVYELVL------INQMAGSE------ 401
DmelGr64d       -------------------------------ALMGYELVL------FRQMQGAV------ 410
AgamGr20        -------------------------------SILTYELVL------MRFHR--------- 424
AaegGr61a       -------------------------------TIITYELVL------LSFRKRIM------ 424
AgamGr21        -------------------------------TIVTYELVM------LKFDQ--------- 450
AaegGr64a       -------------------------------TIVTYELVM------LKFDK--------- 333
DmelGr64a       -------------------------------TIVTYELVL------LQFDE--------- 444
DmelGr61a       -------------------------------TLVTYELML------LQIDA--------- 427
AgamGr15        -------------------------------AIITYELVL------IQFHKD-E------ 433
AaegGr1         -------------------------------SIITYELVL------IQFHQD-E------ 437
DmelGr5a        -------------------------------TVATYELVL------IQFHEDKK------ 434
DmelGr64f       -------------------------------TIVTYELVL------IQFHEDND------ 459
DmelGr64e       -------------------------------TIVTYELIL------LQFNGEEK------ 453
AmelGr1         -------------------------------TIVTYELVL------VQFNTTQQ------ 382
AmelGr2         -------------------------------AIITYEVVL------LQFNGK-------- 470
AgamGr16        -------------------------------TVVTYELVL------TQVNEAEA------ 404
AaegGr64e       -------------------------------TIVTYELVL------LQVNDAEE------ 425
TCasGr6         -------------------------------TIAIYLIVL------LQFKISLLNMR--- 386
TcasGr26        -------------------------------TIAIYLIVL------LQFKISLLNMR--- 386
TcasGr33        -------------------------------TIAIYLIVL------LQFKISLLNMR--- 386
AgamGr23        -------------------------------TIAIYLIVL------LQFKLSLISQQ--- 470
AaegGr21a       -------------------------------TIAIYLVVL------LQFKLSLISQQ--- 462
TcasGr10        -------------------------------SIATYLVML------MQFRLTLMRN---- 415
TcasGr39        -------------------------------SIATYLVML------MQFRLTLMRN---- 415
AgamGr22        -------------------------------FMATYLVVL------MQFKLTLLRQS--- 435
AaegGr21b       -------------------------------FMATYLVVL------MQFKLTLLRQS--- 441
DmelGr21a       -------------------------------FMATYLVVL------LQFKITEQRR---- 441
AgamGr24        -------------------------------TMVTYLVVL------LQFQISIPDE---- 365
AaegGr63a       -------------------------------TMVTYLVVL------LQFQISIPDD---- 430
DmelGr63a       -------------------------------TMVTYLVVL------LQFQISIPTDKGDS 463
TcasGr3         -------------------------------TMVTYLVVL------LQFQISIPEE---- 421
DmelOr83b       -------------------------------AVVTYFMVL------VQLK---------- 486
AgamOr7         -------------------------------AVVTYFMVL------VQLK---------- 478
HvirOr2         -------------------------------AVVTYFMVL------VQLK---------- 472
TcasOr16        -------------------------------AVVTYFMVL------VQLK---------- 574
TcasOr22        -------------------------------AVVTYFMVL------VQLK---------- 613
AmelOr2         -------------------------------AMVTYFMVL------VQLK---------- 478
TcasGr31        ------------------------------------------------------------
TcasGr35        ----------------------------VLNSLTTFLLVMIQFKEN-------------- 251
DmelGr59c       ----------------------------MLSSVITHTMVLVQWEIQNDES---------- 397
DmelGr92a       ----------------------------FLSALLSGLAFIAQYRMQVGNG---------- 386
DmelGr28a       ----------------------------ITGAATCYLIILIQFRFTHHMDDTSSNSTNNL 443
DmelGr64b       ------------------------------TTIITYELMISDVINQGSIRQKTQYCREY- 406
TcasGr71        ----------------------------IIGNVTTFFIIIIQFNNKH------------- 347
AgamGr19        -------------------------------AVITYELVMLHFAQTTANQGIVRACSPEQ 402
AaegGr64b       ----------------------------IVAVIFTYELVMIKYSRMTAADVGIPENCSAM 350
DmelGr93d       -------------------------------AMFCYLVFVTQCVIVYRRRYVI------- 381
TcasGr34        ----------------------------ILNTVSTLLIVMVQFDKER------------- 324
TcasGr43        ----------------------------VLGTACTFLIIIVQFRNS-------------- 353
TcasGr47        ----------------------------VLDTVVAFLMVVAQFQD--------------- 321
AmelGr3         ----------------------------IAGVVTTYLVILIQFQNADDTKDDFDIIRNAT 456
AgamGr32b       ------------------------------------------------------------
AgamGr50        ----------------------------IVASTTSYMIILVQYYLQE------------- 404
TcasGr20        ----------------------------FGGALTTFLVILFQYGGIHGNVY--------- 393
TcasGr79        ----------------------------LVGAVVTFLIVLLQFQIN-------------- 348
TcasGr15        ------------------------AESQTINFMFSFGFILLRFLSVFESCTRLNNESRKP 419
TcasGr60        -----------------------CVLSLHCAAVGGSANIMSGCSTSTSTNINTKLFWLY- 364
AgamGr38        ----------------------------ILSTAATYIIILLQFESAGSSSG--------- 428
TcasGr51        ----------------------------LIANVAMYFVISVQFNQNQKE----------- 771
DmelGr39b       ----------------------------ILAAKVTYLIVLMQFSSV-------------- 369
AgamGr28        ----------------------------IFVALLSFMIILIQFDSAKLNKRDGRVTAIDQ 393
TcasGr61        ----------------------------VVGAATTYITIIYQFQVNKPTCGP-------- 670
TcasGr50        ---------------------------------LDLARTVPKFENKNKETEQ-------- 350
AgamGr12        ----------------------------ALTSMITYLVILIQFRKYDFEDYVVTFRNDSN 390
AgamGr36        ----------------------------MFGFVVTYLIILLQFDKFSDDASKKNFLTS-- 404
AgamGr48        ----------------------------LATTVNMYLIVLVQFQLNID------------ 401
AgamGr49a       ----------------------------MIATITSYLIMLVQFQLAEI------------ 401
AgamGr49b       ----------------------------MIATITSYLIMLVQFQLAEI------------ 405
DmelGr2a        ----------------------------IVGATTTYLIILIQFHMSESTIGSDSNGQ--- 414
DmelGr32a       ----------------------------IFSAVTTYLVILIQFKQLEDSKVEDPVPEQT- 461
```

```
DmelGr68a      ----------------------------IFSAVTTYLVILIQFKQLEDSKVEDISQA--- 389
AmelGr7        ----------------------------VIGTITTYLVVMIQVGDLSNSDKSILS----- 410
AmelGr8        ----------------------------MTCKITTFLLMLIQFLFVPPC----------- 395
AmelGr9        ----------------------------ITKGVLIYILILFQFLLNYRLCS--------- 391
TcasGr57       ----------------------------IFGSVTSYIIISIQFIE--------------- 387
TcasGr41       ----------------------------LFGSTAMNVIILLQFQKQGKYN---------- 398
AgamGr34       ----------------------------MIGAITTYLVIYIQFYILYADEVKKSSFVSRF 427
AgamGr35       ----------------------------LVAGMVTYLIIFIQFNSMVPAGKDDTHHTRKQ 444
AgamGr39       ----------------------------MISSFATYLNILVQFDIMQLRASGANNNDTLL 422
AgamGr40       ----------------------------VAATLVMHVVILVQFDLSIIANKV-------- 393
AaegGr28d      ----------------------------MAGSFATYLVVLLQFDLANLNGI--------- 398
AgamGr42       ----------------------------IISALATYSVILIQFELGVPKFFISAILQQNG 428
AgamGr41       ----------------------------ACSEAATYLIIMIQFETKI------------- 437
AgamGr43       ----------------------------VFSAATSYLIVLLQFDLSKDLEKSTITA---- 428
DmelGr33a      ----------------------------TVSAATSYLIVLLQFDMTAILRNEGLMS---- 475
AgamGr44       ----------------------------IAAAVTNYLIILIQFEMAIEQ----------- 397
AgamGr13       ----------------------------MLAAISNFVVLLMQFHIDYSKSKISYWHGNNS 411
AgamGr45       ----------------------------IIGATTTYLIILIQFDQSFNGD---------- 434
AgamGr46       ----------------------------IVGSLATYLLILIQFDIAQNGAKWKTELRAPL 434
Dmelr93a       ----------------------------ILSAIISYLFILIQFGITGGFEASEDIKNRFD 419
AmelGr6        ----------------------------IAHVMSTYLIILLQFPSS-------------- 439
AmelGr10       ----------------------------VAGLIRVDLPLLLSIFSALTTYLVILT----- 386
TcasGr28       ------------------------------------------------------------
TcasGr40       --------------------------------FFEFSVPLAIFAVV-------------- 373
AgamGr3        ----------------------------LVIVILTYVIIFIEIAD--------------- 374
AgamGr4        ------------------------------TGITTYMIIFITFSKDIKLEDIDDE----- 422
AgamGr6        ----------------------------IFGSITSYLVIYINFIPKTDTFNDYNKEHNLV 421
AgamGr7        ----------------------------IAAAITTYMVIFIQFMPKEDTPTDATTASNST 430
AgamGr47       ----------------------------ILSALAMYLVFLIQFDVAPPVGLNSPNLTIPS 412
DmelGr8a       ----------------------------MACSVGTYMIYSIQFIPKFSNTYM-------- 385
AgamGr5        ----------------------------IIFSVGAYISIFIQQAINQIDELKCNDYEKQC 441
AgamGr8        ----------------------------VLKIVTTYKILFDCYTKAKTSTKPPSAAQNPY 382
DmelGr9a       ----------------------------MFFFILEALVIFLQFVSLVRT----------- 341
DmelGr59f      ----------------------------LLVASADFFIFLLQYDVTYEALSKSVQGNVTR 404
DmelGr77a      ----------------------------IVGAILEYLMILIQFDKVLNK----------- 449
DmelGr85a      ----------------------------SASLILVHVLYMVQSDYISITN---------- 397
TcasGr27       ----------------------------IMQKAWSFFTALKNMHDIRNN----------- 346
TcasGr48       --------------------------SLLNDILFPVAGFISDFDCDINSIQYFCLMARCL 392
AgamGr1        -----------------------------CGVIATYLTVFLQFDNGDSL----------- 387
DmelGr10a      ----------------------------IAVTAFSYFITLVQFDLYLRKKS--------- 408
DmelGr59e      ----------------------------FIGVLMAIVIFLIQIGLGNKSLMGVALNRSNW 396
DmelGr10b      ----------------------------LISCSLSYGIIIIQIGYIPG------------ 373
DmelGr89a      ----------------------------LVSYAMNFIVVILQTSFEHINHGEI------- 362
DmelGr57a      ---------------------------LFTSILVNYLLILIQFAMTQKMGEQIEQQKIAL 410
DmelGr47b      -------------------------------GMANYVIILVQLLFQQQQIKDHQLTSGKD 400
DmelGr58a      ----------------------------YFFYVLVQVLVLVQFDLKNKVEKRN------- 395
DmelGr58b      ----------------------------YIGSILSNVIILMQFDLRRQRQPINDRQYLIH 400
DmelGr98a      ----------------------------FFFGMISYIVICIQFSINFRAKKMSNEQMSQN 383
AaegGr93a      ----------------------------IIAASCSYLILLIQTA---------------- 395
TcasGr19       ----------------------------MIWNSTRRLFVVW------------------- 355
DmelGr47a      ----------------------------LLAKSTLYTICCLQNDYNKLKA---------- 361
DmelGr58c      ----------------------------MTRSIITNVLVLCQIAYKKYG----------- 412


TcasGr38       ------------------------------------------------------------
TcasGr46       RCKFSEFMTKLLEFDVKLQSDRLMIDYKTQKTGSKIHLFVKCVFLMGYQIFDWVQKEQSL 802
TcasGr1        ------------------------------------------------------------
TcasGr123      ------------------------------------------------------------
TcasGr62       ------------------------------------------------------------
TcasGr25       ALTS------TEHSLSQSQHIQKIPKN--------------------------------- 314
TcasGr59       ALTS------TEHSLSQSQHIQKIPKN--------------------------------- 489
AgamGr2        GSTT---AMAVVEGVVTTALTTYVSN---------------------------------- 445
AaegGr66a      VTAA---TVAPLLNLSTTALSTYVANP--------------------------------- 507
DmelGr66a      NDTAGLVGAATDMDNISSTLRDFVTTTMTPAV---------------------------- 530
TcasGr11       ------------------------------------------------------------
TcasGr55       C----------------------------------------------------------- 402
TcasGr21       ------------------------------------------------------------
TcasGr56       ------------------------------------------------------------
AaegGr         TPANTTLSP--------------------------------------------------- 449
AaegGr28a      ------------------------------------------------------------
```

```
AgamGr33        G--NETW----------------------------------------------------- 443
DmelGr28bB      TFNNMTNHTL-------------------------------------------------- 443
DmelGr28bC      TFNNMTNHTL-------------------------------------------------- 470
DmelGr28bD      TFNNMTNHTL-------------------------------------------------- 440
DmelGr28bA      TFNNMTNHTL-------------------------------------------------- 452
DmelGr28bE      TFNNMTNHTL-------------------------------------------------- 447
AgamGr25        ------------------------------------------------------------
AaegGr43a       ------------------------------------------------------------
DmelGr43a       ------------------------------------------------------------
AgamGr37b       GST--------------------------------------------------------- 379
AgamGr37d       GST--------------------------------------------------------- 375
AgamGr37a       GST--------------------------------------------------------- 389
AgamGr37c       GST--------------------------------------------------------- 383
AgamGr37e       GST--------------------------------------------------------- 383
AgamGr37f       GST--------------------------------------------------------- 410
AaegGr28c       SGV--------------------------------------------------------- 573
AaegGr28b       TTTVKPKQ---------------------------------------------------- 427
AaegGr28e       TTTAKPKQ---------------------------------------------------- 427
TcasGr14        ------------------------------------------------------------
TcasGr49        ------------------------------------------------------------
AmelGr4         L----------------------------------------------------------- 415
AmelGr5         R----------------------------------------------------------- 414
TcasGr16        ------------------------------------------------------------
TcasGr22        ------------------------------------------------------------
TcasGr17        ------------------------------------------------------------
TcasGr150       ------------------------------------------------------------
TcasGr32        IPFYSTLRPINLLVQIILDVILLILNVHTILTTITKRNQWCKLLENLKLDRENN------ 420
TcasGr54        ------------------------------------------------------------
TcasGr37        ------------------------------------------------------------
TcasGr53        ------------------------------------------------------------
TcasGr5         --------PVVLFIQVAADTVLFILNISTIIITARKKQQWNSLIKILKTVSNRNDKGDIF 395
TcasGr12        ------------------------------------------------------------
TcasGr13        ------------------------------------------------------------
TcasGr104       ------------------------------------------------------------
TcasGr45        ------------------------------------------------------------
TcasGr98        ------------------------------------------------------------
TcasGr52        ------------------------------------------------------------
TcasGr105       ------------------------------------------------------------
TcasGr4         ------------------------------------------------------------
TcasGr44        ------------------------------------------------------------
AgamGr9a        TSNVSTVQP--------------------------------------------------- 397
AgamGr9c        TSNVSTVQP--------------------------------------------------- 396
AgamGr9b        TSNVSTVQP--------------------------------------------------- 447
AgamGr9d        TSNVSTVQP--------------------------------------------------- 403
AgamGr9e        TSNVSTVQP--------------------------------------------------- 399
AgamGr9f        TSNVSTVQP--------------------------------------------------- 396
AgamGr9g        TSNVSTVQP--------------------------------------------------- 399
AgamGr9i        TSNVSTVQP--------------------------------------------------- 400
AgamGr9h        TSNVSTVQP--------------------------------------------------- 405
AgamGr9j        TSNVSTVQP--------------------------------------------------- 436
AgamGr9l        TSNVSTVQP--------------------------------------------------- 426
AgamGr9k        TSNVSTVQP--------------------------------------------------- 418
AgamGr9m        TSNVSTVQP--------------------------------------------------- 421
AgamGr9n        TSNVSTVQP--------------------------------------------------- 388
AgamGr10        TSNVSTVQP--------------------------------------------------- 388
AgamGr11        TANGSDPNLKVH------------------------------------------------ 397
DmelGr23aA      ------------------------------------------------------------
DmelGr23aB      ------------------------------------------------------------
DmelGr39aA      ------------------------------------------------------------
DmelGr39aB      ------------------------------------------------------------
DmelGr39aC      ------------------------------------------------------------
DmelGr39aD      ------------------------------------------------------------
DmelGr98c       DTS--------------------------------------------------------- 408
DmelGr98d       STELKAYTA--------------------------------------------------- 412
DmelGr98b       ------------------------------------------------------------
AgamGr51        ------------------------------------------------------------
AgamGr52        ------------------------------------------------------------
DmelGr94a       ------------------------------------------------------------
DmelGr97a       ------------------------------------------------------------
DmelGr93b       ------------------------------------------------------------
```

```
DmelGr93c       ------------------------------------------------------------
DmelGr22d       ------------------------------------------------------------
DmelGr22e       ------------------------------------------------------------
DmelGr22a       ------------------------------------------------------------
DmelGr22b       ------------------------------------------------------------
DmelGr22c       ------------------------------------------------------------
DmelGr22f       ------------------------------------------------------------
DmelGr36a       ------------------------------------------------------------
DmelGr36c       ------------------------------------------------------------
DmelGr36b       ------------------------------------------------------------
DmelGr59d       ------------------------------------------------------------
DmelGr59a       ------------------------------------------------------------
DmelGr59b       ------------------------------------------------------------
AgamGr26        SPSLPNDTNTPGNGTTI------------------------------------------- 445
AgamGr27        ------------------------------------------------------------
AgamGr29        ------------------------------------------------------------
AgamGr31        ------------------------------------------------------------
AgamGr30        IYNISVET---------------------------------------------------- 422
AgamGr32a       ------------------------------------------------------------
TcasGr2         TLLTIGLYFQLCEFSRLNFSVEHLLMELSRAIDSVACSKTVSDGIRHYYVNVTFPHLFNG 512
TcasGr9         ------------------------RDEKPYSRNSIC------------------------ 408
TcasGr7         -------------------------ETISEQIDSCPVYL--------------------- 429
TcasGr30        -------------------------ETISEQIDSCPVYL--------------------- 394
TcasGr29        -------------------------ETISEQIDSCPVYL--------------------- 429
AaegGr64f       -------------------------DTTRDCDF--------------------------- 380
AaegGr64a3      ------------------------------------------------------------
AgamGr14        ------------------------------------------------------------
AgamGr18        -------------------------DTSTDCSYF-------------------------- 417
AaegGr64d       -------------------------DPTTDCSFY-------------------------- 449
AgamGr17        -------------------------EKSDFCDGVKRLY---------------------- 410
AaegGr64c       -------------------------DNTQFCDGGHRLY---------------------- 434
DmelGr64c       -------------------------VQKSFCEGGVGSSKSIFS----------------- 419
DmelGr64d       -------------------------VQKSICSRGPGSSMSIFFS---------------- 429
AgamGr20        -------------------------SSKGVGEDLPCGYID-------------------- 439
AaegGr61a       -------------------------DEPDDNNDVSCEPLHLD------------------ 441
AgamGr21        -------------------------ESESKGNIPLCTKFRRFEHVSV------------- 472
AaegGr64a       -------------------------ESEGKGYIRPCS----------------------- 345
DmelGr64a       -------------------------PNRRKGLQPLCA----------------------- 456
DmelGr61a       -------------------------KSHKG---LRCA----------------------- 436
AgamGr15        -------------------------ASDVDLCKLKRMDTL-------------------- 448
AaegGr1         -------------------------TADYDLCTFRRT----------------------- 449
DmelGr5a        -------------------------TWDCSPFNLD------------------------- 444
DmelGr64f       -------------------------LWDCDQSYYS------------------------- 469
DmelGr64e       -------------------------VPGCFEN---------------------------- 460
AmelGr1         -------------------------TDASNATIVCEVK---------------------- 395
AmelGr2         ------------------------------------------------------------
AgamGr16        -------------------------KNGDDNPCTWVAYKTVCQRVYGVSFSGPDRT---- 435
AaegGr64e       -------------------------KNGDQNPCT-------------------------- 434
TCasGr6         -----------------------G------------------------------------ 387
TcasGr26        -----------------------G------------------------------------ 387
TcasGr33        -----------------------G------------------------------------ 387
AgamGr23        -----------------------IPVEIIENVKLLQKQ---------------------- 485
AaegGr21a       -----------------------MPIELME-IKHSHKG---------------------- 476
TcasGr10        --------------------------AQLAARRAIANVSVSSGNTTMS------------ 437
TcasGr39        --------------------------AQLAARRAIAN----------------------- 426
AgamGr22        -----------------------AKNAFISALKANLSRIRSLDADKVNT----------- 461
AaegGr21b       -----------------------ARKALIPALRANLTKLKEN------------------ 460
DmelGr21a       ----------------------------IGQQQA-------------------------- 447
AgamGr24        -----------------------PSAMLMHSNS-SHS----------------------- 378
AaegGr63a       -----------------------SSMLVMHNMTGSYRE---------------------- 445
DmelGr63a       EGANNITVVDFV------MDSLDNDMSLMGASTLSTTTVGTTLPPPIMKLKGRKG----- 512
TcasGr3         -------------------------ASPTNSTTITTQTPN-------------------- 436
DmelOr83b       ------------------------------------------------------------
AgamOr7         ------------------------------------------------------------
HvirOr2         ------------------------------------------------------------
TcasOr16        ------------------------------------------------------------
TcasOr22        ------------------------------------------------------------
AmelOr2         ------------------------------------------------------------
TcasGr31        ------------------------------------------------------------
TcasGr35        ------------------------------------------------------------
```

```
DmelGr59c       ------------------------------------------------------------
DmelGr92a       ------------------------------------------------------------
DmelGr28a       HSIHLGD----------------------------------------------------- 450
DmelGr64b       ------------------------------------------------------------
TcasGr71        ------------------------------------------------------------
AgamGr19        FLFQPKMQITTN------------------------------------------------ 414
AaegGr64b       AFSQD------------------------------------------------------- 355
DmelGr93d       ------------------------------------------------------------
TcasGr34        ------------------------------------------------------------
TcasGr43        ------------------------------------------------------------
TcasGr47        ------------------------------------------------------------
AmelGr3         QILKNASPLQNFTGLKTIV----------------------------------------- 475
AgamGr32b       ------------------------------------------------------------
AgamGr50        ------------------------------------------------------------
TcasGr20        ------------------------------------------------------------
TcasGr79        ------------------------------------------------------------
TcasGr15        SFVIQFSHIPVDNIEFAGALVTYELTLFQYHAFYLT------------------------ 455
TcasGr60        ------------------------------------------------------------
AgamGr38        ------------------------------------------------------------
TcasGr51        ------------------------------------------------------------
DmelGr39b       ------------------------------------------------------------
AgamGr28        LYTPTNS----------------------------------------------------- 400
TcasGr61        ------------------------------------------------------------
TcasGr50        ------------------------------------------------------------
AgamGr12        ISF--------------------------------------------------------- 393
AgamGr36        ------------------------------------------------------------
AgamGr48        ------------------------------------------------------------
AgamGr49a       ------------------------------------------------------------
AgamGr49b       ------------------------------------------------------------
DmelGr2a        ------------------------------------------------------------
DmelGr32a       ------------------------------------------------------------
DmelGr68a       ------------------------------------------------------------
AmelGr7         ------------------------------------------------------------
AmelGr8         ------------------------------------------------------------
AmelGr9         ------------------------------------------------------------
TcasGr57        ------------------------------------------------------------
TcasGr41        ------------------------------------------------------------
AgamGr34        QI---------------------------------------------------------- 429
AgamGr35        HVTEERF----------------------------------------------------- 451
AgamGr39        SFERS------------------------------------------------------- 427
AgamGr40        ------------------------------------------------------------
AaegGr28d       ------------------------------------------------------------
AgamGr42        DMTTTNSQP--------------------------------------------------- 437
AgamGr41        ------------------------------------------------------------
AgamGr43        ------------------------------------------------------------
DmelGr33a       ------------------------------------------------------------
AgamGr44        ------------------------------------------------------------
AgamGr13        SKPLTVRS---------------------------------------------------- 419
AgamGr45        ------------------------------------------------------------
AgamGr46        V----------------------------------------------------------- 435
Dmelr93a        ------------------------------------------------------------
AmelGr6         ------------------------------------------------------------
AmelGr10        ------------------------------------------------------------
TcasGr28        ------------------------------------------------------------
TcasGr40        ------------------------------------------------------------
AgamGr3         ------------------------------------------------------------
AgamGr4         ------------------------------------------------------------
AgamGr6         TT---------------------------------------------------------- 423
AgamGr7         LANSTATATPE------------------------------------------------- 441
AgamGr47        IELIDFEV---------------------------------------------------- 420
DmelGr8a        ------------------------------------------------------------
AgamGr5         ------------------------------------------------------------
AgamGr8         RRNHRVKRTLECVEAEAKSFLTAKKII--------------------------------- 409
DmelGr9a        ------------------------------------------------------------
DmelGr59f       YK---------------------------------------------------------- 406
DmelGr77a       ------------------------------------------------------------
DmelGr85a       ------------------------------------------------------------
TcasGr27        ------------------------------------------------------------
TcasGr48        NTLIFYYHISTYEYSI-------------------------------------------- 408
AgamGr1         ------------------------------------------------------------
```

```
DmelGr10a       ------------------------------------------------------------
DmelGr59e       VYV--------------------------------------------------------- 399
DmelGr10b       ------------------------------------------------------------
DmelGr89a       ------------------------------------------------------------
DmelGr57a       QEWIGF------------------------------------------------------ 416
DmelGr47b       VDIVGPMGPITHMD---------------------------------------------- 414
DmelGr58a       ------------------------------------------------------------
DmelGr58b       LMKNKTKV---------------------------------------------------- 408
DmelGr98a       ITSTSAPI---------------------------------------------------- 391
AaegGr93a       ------------------------------------------------------------
TcasGr19        ------------------------------------------------------------
DmelGr47a       ------------------------------------------------------------
DmelGr58c       ------------------------------------------------------------


TcasGr38        ------------------------------------------------------------
TcasGr46        AHSVGVFLTIFNAAHCYLATELVLMLKNRYIILNGRLIKMETKAQSVALGKICTLHHHLS 862
TcasGr1         ------------------------------------------------------------
TcasGr123       ------------------------------------------------------------
TcasGr62        ------------------------------------------------------------
TcasGr25        ------------------------------------------------------------
TcasGr59        ------------------------------------------------------------
AgamGr2         ------------------------------------------------------------
AaegGr66a       ------------------------------------------------------------
DmelGr66a       ------------------------------------------------------------
TcasGr11        ------------------------------------------------------------
TcasGr55        ------------------------------------------------------------
TcasGr21        ------------------------------------------------------------
TcasGr56        ------------------------------------------------------------
AaegGr          ------------------------------------------------------------
AaegGr28a       ------------------------------------------------------------
AgamGr33        ------------------------------------------------------------
DmelGr28bB      ------------------------------------------------------------
DmelGr28bC      ------------------------------------------------------------
DmelGr28bD      ------------------------------------------------------------
DmelGr28bA      ------------------------------------------------------------
DmelGr28bE      ------------------------------------------------------------
AgamGr25        ------------------------------------------------------------
AaegGr43a       ------------------------------------------------------------
DmelGr43a       ------------------------------------------------------------
AgamGr37b       ------------------------------------------------------------
AgamGr37d       ------------------------------------------------------------
AgamGr37a       ------------------------------------------------------------
AgamGr37c       ------------------------------------------------------------
AgamGr37e       ------------------------------------------------------------
AgamGr37f       ------------------------------------------------------------
AaegGr28c       ------------------------------------------------------------
AaegGr28b       ------------------------------------------------------------
AaegGr28e       ------------------------------------------------------------
TcasGr14        ------------------------------------------------------------
TcasGr49        ------------------------------------------------------------
AmelGr4         ------------------------------------------------------------
AmelGr5         ------------------------------------------------------------
TcasGr16        ------------------------------------------------------------
TcasGr22        ------------------------------------------------------------
TcasGr17        ------------------------------------------------------------
TcasGr150       ------------------------------------------------------------
TcasGr32        --SSFVVANVLFQLIHVYYTVVFTLLLGVDFVKEYAFEYIQLYSQFILYFLLFATLKILL 478
TcasGr54        ------------------------------------------------------------
TcasGr37        ------------------------------------------------------------
TcasGr53        ------------------------------------------------------------
TcasGr5         WFSPFLVANLAFVTIVTYETFVWTQIMGAEFFKLYAVEYFQMYAQFIVG----------- 444
TcasGr12        ------------------------------------------------------------
TcasGr13        ------------------------------------------------------------
TcasGr104       ------------------------------------------------------------
TcasGr45        ------------------------------------------------------------
TcasGr98        ------------------------------------------------------------
TcasGr52        ------------------------------------------------------------
TcasGr105       ------------------------------------------------------------
TcasGr4         ------------------------------------------------------------
```

```
TcasGr44        ------------------------------------------------------------
AgamGr9a        ------------------------------------------------------------
AgamGr9c        ------------------------------------------------------------
AgamGr9b        ------------------------------------------------------------
AgamGr9d        ------------------------------------------------------------
AgamGr9e        ------------------------------------------------------------
AgamGr9f        ------------------------------------------------------------
AgamGr9g        ------------------------------------------------------------
AgamGr9i        ------------------------------------------------------------
AgamGr9h        ------------------------------------------------------------
AgamGr9j        ------------------------------------------------------------
AgamGr9l        ------------------------------------------------------------
AgamGr9k        ------------------------------------------------------------
AgamGr9m        ------------------------------------------------------------
AgamGr9n        ------------------------------------------------------------
AgamGr10        ------------------------------------------------------------
AgamGr11        ------------------------------------------------------------
DmelGr23aA      ------------------------------------------------------------
DmelGr23aB      ------------------------------------------------------------
DmelGr39aA      ------------------------------------------------------------
DmelGr39aB      ------------------------------------------------------------
DmelGr39aC      ------------------------------------------------------------
DmelGr39aD      ------------------------------------------------------------
DmelGr98c       ------------------------------------------------------------
DmelGr98d       ------------------------------------------------------------
DmelGr98b       ------------------------------------------------------------
AgamGr51        ------------------------------------------------------------
AgamGr52        ------------------------------------------------------------
DmelGr94a       ------------------------------------------------------------
DmelGr97a       ------------------------------------------------------------
DmelGr93b       ------------------------------------------------------------
DmelGr93c       ------------------------------------------------------------
DmelGr22d       ------------------------------------------------------------
DmelGr22e       ------------------------------------------------------------
DmelGr22a       ------------------------------------------------------------
DmelGr22b       ------------------------------------------------------------
DmelGr22c       ------------------------------------------------------------
DmelGr22f       ------------------------------------------------------------
DmelGr36a       ------------------------------------------------------------
DmelGr36c       ------------------------------------------------------------
DmelGr36b       ------------------------------------------------------------
DmelGr59d       ------------------------------------------------------------
DmelGr59a       ------------------------------------------------------------
DmelGr59b       ------------------------------------------------------------
AgamGr26        ------------------------------------------------------------
AgamGr27        ------------------------------------------------------------
AgamGr29        ------------------------------------------------------------
AgamGr31        ------------------------------------------------------------
AgamGr30        ------------------------------------------------------------
AgamGr32a       ------------------------------------------------------------
TcasGr2         LVDYSLWKALIFQISNLQTTFGGTFGDTFIILLSMAFATRMKQSRTKIEALVKSHIAGAI 572
TcasGr9         ------------------------------------------------------------
TcasGr7         ------------------------------------------------------------
TcasGr30        ------------------------------------------------------------
TcasGr29        ------------------------------------------------------------
AaegGr64f       ------------------------------------------------------------
AaegGr64a3      ------------------------------------------------------------
AgamGr14        ------------------------------------------------------------
AgamGr18        ------------------------------------------------------------
AaegGr64d       ------------------------------------------------------------
AgamGr17        ------------------------------------------------------------
AaegGr64c       ------------------------------------------------------------
DmelGr64c       ------------------------------------------------------------
DmelGr64d       ------------------------------------------------------------
AgamGr20        ------------------------------------------------------------
AaegGr61a       ------------------------------------------------------------
AgamGr21        ------------------------------------------------------------
AaegGr64a       ------------------------------------------------------------
DmelGr64a       ------------------------------------------------------------
DmelGr61a       ------------------------------------------------------------
```

```
AgamGr15        ------------------------------------------------------------
AaegGr1         ------------------------------------------------------------
DmelGr5a        ------------------------------------------------------------
DmelGr64f       ------------------------------------------------------------
DmelGr64e       ------------------------------------------------------------
AmelGr1         ------------------------------------------------------------
AmelGr2         ------------------------------------------------------------
AgamGr16        ------------------------------------------------------------
AaegGr64e       ------------------------------------------------------------
TCasGr6         ------------------------------------------------------------
TcasGr26        ------------------------------------------------------------
TcasGr33        ------------------------------------------------------------
AgamGr23        ------------------------------------------------------------
AaegGr21a       ------------------------------------------------------------
TcasGr10        ------------------------------------------------------------
TcasGr39        ------------------------------------------------------------
AgamGr22        ------------------------------------------------------------
AaegGr21b       ------------------------------------------------------------
DmelGr21a       ------------------------------------------------------------
AgamGr24        ------------------------------------------------------------
AaegGr63a       ------------------------------------------------------------
DmelGr63a       ------------------------------------------------------------
TcasGr3         ------------------------------------------------------------
DmelOr83b       ------------------------------------------------------------
AgamOr7         ------------------------------------------------------------
HvirOr2         ------------------------------------------------------------
TcasOr16        ------------------------------------------------------------
TcasOr22        ------------------------------------------------------------
AmelOr2         ------------------------------------------------------------
TcasGr31        ------------------------------------------------------------
TcasGr35        ------------------------------------------------------------
DmelGr59c       ------------------------------------------------------------
DmelGr92a       ------------------------------------------------------------
DmelGr28a       ------------------------------------------------------------
DmelGr64b       ------------------------------------------------------------
TcasGr71        ------------------------------------------------------------
AgamGr19        ------------------------------------------------------------
AaegGr64b       ------------------------------------------------------------
DmelGr93d       ------------------------------------------------------------
TcasGr34        ------------------------------------------------------------
TcasGr43        ------------------------------------------------------------
TcasGr47        ------------------------------------------------------------
AmelGr3         ------------------------------------------------------------
AgamGr32b       ------------------------------------------------------------
AgamGr50        ------------------------------------------------------------
TcasGr20        ------------------------------------------------------------
TcasGr79        ------------------------------------------------------------
TcasGr15        ------------------------------------------------------------
TcasGr60        ------------------------------------------------------------
AgamGr38        ------------------------------------------------------------
TcasGr51        ------------------------------------------------------------
DmelGr39b       ------------------------------------------------------------
AgamGr28        ------------------------------------------------------------
TcasGr61        ------------------------------------------------------------
TcasGr50        ------------------------------------------------------------
AgamGr12        ------------------------------------------------------------
AgamGr36        ------------------------------------------------------------
AgamGr48        ------------------------------------------------------------
AgamGr49a       ------------------------------------------------------------
AgamGr49b       ------------------------------------------------------------
DmelGr2a        ------------------------------------------------------------
DmelGr32a       ------------------------------------------------------------
DmelGr68a       ------------------------------------------------------------
AmelGr7         ------------------------------------------------------------
AmelGr8         ------------------------------------------------------------
AmelGr9         ------------------------------------------------------------
TcasGr57        ------------------------------------------------------------
TcasGr41        ------------------------------------------------------------
AgamGr34        ------------------------------------------------------------
AgamGr35        ------------------------------------------------------------
```

```
AgamGr39        ------------------------------------------------------------
AgamGr40        ------------------------------------------------------------
AaegGr28d       ------------------------------------------------------------
AgamGr42        ------------------------------------------------------------
AgamGr41        ------------------------------------------------------------
AgamGr43        ------------------------------------------------------------
DmelGr33a       ------------------------------------------------------------
AgamGr44        ------------------------------------------------------------
AgamGr13        ------------------------------------------------------------
AgamGr45        ------------------------------------------------------------
AgamGr46        ------------------------------------------------------------
Dmelr93a        ------------------------------------------------------------
AmelGr6         ------------------------------------------------------------
AmelGr10        ------------------------------------------------------------
TcasGr28        ------------------------------------------------------------
TcasGr40        ------------------------------------------------------------
AgamGr3         ------------------------------------------------------------
AgamGr4         ------------------------------------------------------------
AgamGr6         ------------------------------------------------------------
AgamGr7         ------------------------------------------------------------
AgamGr47        ------------------------------------------------------------
DmelGr8a        ------------------------------------------------------------
AgamGr5         ------------------------------------------------------------
AgamGr8         ------------------------------------------------------------
DmelGr9a        ------------------------------------------------------------
DmelGr59f       ------------------------------------------------------------
DmelGr77a       ------------------------------------------------------------
DmelGr85a       ------------------------------------------------------------
TcasGr27        ------------------------------------------------------------
TcasGr48        ------------------------------------------------------------
AgamGr1         ------------------------------------------------------------
DmelGr10a       ------------------------------------------------------------
DmelGr59e       ------------------------------------------------------------
DmelGr10b       ------------------------------------------------------------
DmelGr89a       ------------------------------------------------------------
DmelGr57a       ------------------------------------------------------------
DmelGr47b       ------------------------------------------------------------
DmelGr58a       ------------------------------------------------------------
DmelGr58b       ------------------------------------------------------------
DmelGr98a       ------------------------------------------------------------
AaegGr93a       ------------------------------------------------------------
TcasGr19        ------------------------------------------------------------
DmelGr47a       ------------------------------------------------------------
DmelGr58c       ------------------------------------------------------------


TcasGr38        ------------------------------------------------------------
TcasGr46        KLIRLFNEIFGHGLLLMFGISFLLVTQTIFALCVVLQLQENDWLQLGYLIFVSTLYTANV 922
TcasGr1         ------------------------------------------------------------
TcasGr123       ------------------------------------------------------------
TcasGr62        ------------------------------------------------------------
TcasGr25        ------------------------------------------------------------
TcasGr59        ------------------------------------------------------------
AgamGr2         ------------------------------------------------------------
AaegGr66a       ------------------------------------------------------------
DmelGr66a       ------------------------------------------------------------
TcasGr11        ------------------------------------------------------------
TcasGr55        ------------------------------------------------------------
TcasGr21        ------------------------------------------------------------
TcasGr56        ------------------------------------------------------------
AaegGr          ------------------------------------------------------------
AaegGr28a       ------------------------------------------------------------
AgamGr33        ------------------------------------------------------------
DmelGr28bB      ------------------------------------------------------------
DmelGr28bC      ------------------------------------------------------------
DmelGr28bD      ------------------------------------------------------------
DmelGr28bA      ------------------------------------------------------------
DmelGr28bE      ------------------------------------------------------------
AgamGr25        ------------------------------------------------------------
AaegGr43a       ------------------------------------------------------------
```

```
DmelGr43a       ------------------------------------------------------------
AgamGr37b       ------------------------------------------------------------
AgamGr37d       ------------------------------------------------------------
AgamGr37a       ------------------------------------------------------------
AgamGr37c       ------------------------------------------------------------
AgamGr37e       ------------------------------------------------------------
AgamGr37f       ------------------------------------------------------------
AaegGr28c       ------------------------------------------------------------
AaegGr28b       ------------------------------------------------------------
AaegGr28e       ------------------------------------------------------------
TcasGr14        ------------------------------------------------------------
TcasGr49        ------------------------------------------------------------
AmelGr4         ------------------------------------------------------------
AmelGr5         ------------------------------------------------------------
TcasGr16        ------------------------------------------------------------
TcasGr22        ------------------------------------------------------------
TcasGr17        ------------------------------------------------------------
TcasGr150       ------------------------------------------------------------
TcasGr32        GKYTQLKACLMEEKRPMVSLQVIKAQIYSLRETVDVFNDIFGWPFLLLITFTSLQIMVYL 538
TcasGr54        ------------------------------------------------------------
TcasGr37        ------------------------------------------------------------
TcasGr53        ------------------------------------------------------------
TcasGr5         ------------------------------------------------------------
TcasGr12        ------------------------------------------------------------
TcasGr13        ------------------------------------------------------------
TcasGr104       ------------------------------------------------------------
TcasGr45        ------------------------------------------------------------
TcasGr98        ------------------------------------------------------------
TcasGr52        ------------------------------------------------------------
TcasGr105       ------------------------------------------------------------
TcasGr4         ------------------------------------------------------------
TcasGr44        ------------------------------------------------------------
AgamGr9a        ------------------------------------------------------------
AgamGr9c        ------------------------------------------------------------
AgamGr9b        ------------------------------------------------------------
AgamGr9d        ------------------------------------------------------------
AgamGr9e        ------------------------------------------------------------
AgamGr9f        ------------------------------------------------------------
AgamGr9g        ------------------------------------------------------------
AgamGr9i        ------------------------------------------------------------
AgamGr9h        ------------------------------------------------------------
AgamGr9j        ------------------------------------------------------------
AgamGr9l        ------------------------------------------------------------
AgamGr9k        ------------------------------------------------------------
AgamGr9m        ------------------------------------------------------------
AgamGr9n        ------------------------------------------------------------
AgamGr10        ------------------------------------------------------------
AgamGr11        ------------------------------------------------------------
DmelGr23aA      ------------------------------------------------------------
DmelGr23aB      ------------------------------------------------------------
DmelGr39aA      ------------------------------------------------------------
DmelGr39aB      ------------------------------------------------------------
DmelGr39aC      ------------------------------------------------------------
DmelGr39aD      ------------------------------------------------------------
DmelGr98c       ------------------------------------------------------------
DmelGr98d       ------------------------------------------------------------
DmelGr98b       ------------------------------------------------------------
AgamGr51        ------------------------------------------------------------
AgamGr52        ------------------------------------------------------------
DmelGr94a       ------------------------------------------------------------
DmelGr97a       ------------------------------------------------------------
DmelGr93b       ------------------------------------------------------------
DmelGr93c       ------------------------------------------------------------
DmelGr22d       ------------------------------------------------------------
DmelGr22e       ------------------------------------------------------------
DmelGr22a       ------------------------------------------------------------
DmelGr22b       ------------------------------------------------------------
DmelGr22c       ------------------------------------------------------------
DmelGr22f       ------------------------------------------------------------
DmelGr36a       ------------------------------------------------------------
```

```
DmelGr36c       ------------------------------------------------------------
DmelGr36b       ------------------------------------------------------------
DmelGr59d       ------------------------------------------------------------
DmelGr59a       ------------------------------------------------------------
DmelGr59b       ------------------------------------------------------------
AgamGr26        ------------------------------------------------------------
AgamGr27        ------------------------------------------------------------
AgamGr29        ------------------------------------------------------------
AgamGr31        ------------------------------------------------------------
AgamGr30        ------------------------------------------------------------
AgamGr32a       ------------------------------------------------------------
TcasGr2         VTYELVVIQFNLRL---------------------------------------------- 586
TcasGr9         ------------------------------------------------------------
TcasGr7         ------------------------------------------------------------
TcasGr30        ------------------------------------------------------------
TcasGr29        ------------------------------------------------------------
AaegGr64f       ------------------------------------------------------------
AaegGr64a3      ------------------------------------------------------------
AgamGr14        ------------------------------------------------------------
AgamGr18        ------------------------------------------------------------
AaegGr64d       ------------------------------------------------------------
AgamGr17        ------------------------------------------------------------
AaegGr64c       ------------------------------------------------------------
DmelGr64c       ------------------------------------------------------------
DmelGr64d       ------------------------------------------------------------
AgamGr20        ------------------------------------------------------------
AaegGr61a       ------------------------------------------------------------
AgamGr21        ------------------------------------------------------------
AaegGr64a       ------------------------------------------------------------
DmelGr64a       ------------------------------------------------------------
DmelGr61a       ------------------------------------------------------------
AgamGr15        ------------------------------------------------------------
AaegGr1         ------------------------------------------------------------
DmelGr5a        ------------------------------------------------------------
DmelGr64f       ------------------------------------------------------------
DmelGr64e       ------------------------------------------------------------
AmelGr1         ------------------------------------------------------------
AmelGr2         ------------------------------------------------------------
AgamGr16        ------------------------------------------------------------
AaegGr64e       ------------------------------------------------------------
TCasGr6         ------------------------------------------------------------
TcasGr26        ------------------------------------------------------------
TcasGr33        ------------------------------------------------------------
AgamGr23        ------------------------------------------------------------
AaegGr21a       ------------------------------------------------------------
TcasGr10        ------------------------------------------------------------
TcasGr39        ------------------------------------------------------------
AgamGr22        ------------------------------------------------------------
AaegGr21b       ------------------------------------------------------------
DmelGr21a       ------------------------------------------------------------
AgamGr24        ------------------------------------------------------------
AaegGr63a       ------------------------------------------------------------
DmelGr63a       ------------------------------------------------------------
TcasGr3         ------------------------------------------------------------
DmelOr83b       ------------------------------------------------------------
AgamOr7         ------------------------------------------------------------
HvirOr2         ------------------------------------------------------------
TcasOr16        ------------------------------------------------------------
TcasOr22        ------------------------------------------------------------
AmelOr2         ------------------------------------------------------------
TcasGr31        ------------------------------------------------------------
TcasGr35        ------------------------------------------------------------
DmelGr59c       ------------------------------------------------------------
DmelGr92a       ------------------------------------------------------------
DmelGr28a       ------------------------------------------------------------
DmelGr64b       ------------------------------------------------------------
TcasGr71        ------------------------------------------------------------
AgamGr19        ------------------------------------------------------------
AaegGr64b       ------------------------------------------------------------
DmelGr93d       ------------------------------------------------------------
```

```
TcasGr34         ------------------------------------------------------------
TcasGr43         ------------------------------------------------------------
TcasGr47         ------------------------------------------------------------
AmelGr3          ------------------------------------------------------------
AgamGr32b        ------------------------------------------------------------
AgamGr50         ------------------------------------------------------------
TcasGr20         ------------------------------------------------------------
TcasGr79         ------------------------------------------------------------
TcasGr15         ------------------------------------------------------------
TcasGr60         ------------------------------------------------------------
AgamGr38         ------------------------------------------------------------
TcasGr51         ------------------------------------------------------------
DmelGr39b        ------------------------------------------------------------
AgamGr28         ------------------------------------------------------------
TcasGr61         ------------------------------------------------------------
TcasGr50         ------------------------------------------------------------
AgamGr12         ------------------------------------------------------------
AgamGr36         ------------------------------------------------------------
AgamGr48         ------------------------------------------------------------
AgamGr49a        ------------------------------------------------------------
AgamGr49b        ------------------------------------------------------------
DmelGr2a         ------------------------------------------------------------
DmelGr32a        ------------------------------------------------------------
DmelGr68a        ------------------------------------------------------------
AmelGr7          ------------------------------------------------------------
AmelGr8          ------------------------------------------------------------
AmelGr9          ------------------------------------------------------------
TcasGr57         ------------------------------------------------------------
TcasGr41         ------------------------------------------------------------
AgamGr34         ------------------------------------------------------------
AgamGr35         ------------------------------------------------------------
AgamGr39         ------------------------------------------------------------
AgamGr40         ------------------------------------------------------------
AaegGr28d        ------------------------------------------------------------
AgamGr42         ------------------------------------------------------------
AgamGr41         ------------------------------------------------------------
AgamGr43         ------------------------------------------------------------
DmelGr33a        ------------------------------------------------------------
AgamGr44         ------------------------------------------------------------
AgamGr13         ------------------------------------------------------------
AgamGr45         ------------------------------------------------------------
AgamGr46         ------------------------------------------------------------
Dmelr93a         ------------------------------------------------------------
AmelGr6          ------------------------------------------------------------
AmelGr10         ------------------------------------------------------------
TcasGr28         ------------------------------------------------------------
TcasGr40         ------------------------------------------------------------
AgamGr3          ------------------------------------------------------------
AgamGr4          ------------------------------------------------------------
AgamGr6          ------------------------------------------------------------
AgamGr7          ------------------------------------------------------------
AgamGr47         ------------------------------------------------------------
DmelGr8a         ------------------------------------------------------------
AgamGr5          ------------------------------------------------------------
AgamGr8          ------------------------------------------------------------
DmelGr9a         ------------------------------------------------------------
DmelGr59f        ------------------------------------------------------------
DmelGr77a        ------------------------------------------------------------
DmelGr85a        ------------------------------------------------------------
TcasGr27         ------------------------------------------------------------
TcasGr48         ------------------------------------------------------------
AgamGr1          ------------------------------------------------------------
DmelGr10a        ------------------------------------------------------------
DmelGr59e        ------------------------------------------------------------
DmelGr10b        ------------------------------------------------------------
DmelGr89a        ------------------------------------------------------------
DmelGr57a        ------------------------------------------------------------
DmelGr47b        ------------------------------------------------------------
DmelGr58a        ------------------------------------------------------------
DmelGr58b        ------------------------------------------------------------
```

```
DmelGr98a       ------------------------------------------------------------
AaegGr93a       ------------------------------------------------------------
TcasGr19        ------------------------------------------------------------
DmelGr47a       ------------------------------------------------------------
DmelGr58c       ------------------------------------------------------------


TcasGr38        ------------------------------------------------------------
TcasGr46        VCICHVCYSTIQEVSKSGELIHKIATNDHEIIDKIEMFSLQILNERAGFTAAGFFPIDYT 982
TcasGr1         ------------------------------------------------------------
TcasGr123       ------------------------------------------------------------
TcasGr62        ------------------------------------------------------------
TcasGr25        ------------------------------------------------------------
TcasGr59        ------------------------------------------------------------
AgamGr2         ------------------------------------------------------------
AaegGr66a       ------------------------------------------------------------
DmelGr66a       ------------------------------------------------------------
TcasGr11        ------------------------------------------------------------
TcasGr55        ------------------------------------------------------------
TcasGr21        ------------------------------------------------------------
TcasGr56        ------------------------------------------------------------
AaegGr          ------------------------------------------------------------
AaegGr28a       ------------------------------------------------------------
AgamGr33        ------------------------------------------------------------
DmelGr28bB      ------------------------------------------------------------
DmelGr28bC      ------------------------------------------------------------
DmelGr28bD      ------------------------------------------------------------
DmelGr28bA      ------------------------------------------------------------
DmelGr28bE      ------------------------------------------------------------
AgamGr25        ------------------------------------------------------------
AaegGr43a       ------------------------------------------------------------
DmelGr43a       ------------------------------------------------------------
AgamGr37b       ------------------------------------------------------------
AgamGr37d       ------------------------------------------------------------
AgamGr37a       ------------------------------------------------------------
AgamGr37c       ------------------------------------------------------------
AgamGr37e       ------------------------------------------------------------
AgamGr37f       ------------------------------------------------------------
AaegGr28c       ------------------------------------------------------------
AaegGr28b       ------------------------------------------------------------
AaegGr28e       ------------------------------------------------------------
TcasGr14        ------------------------------------------------------------
TcasGr49        ------------------------------------------------------------
AmelGr4         ------------------------------------------------------------
AmelGr5         ------------------------------------------------------------
TcasGr16        ------------------------------------------------------------
TcasGr22        ------------------------------------------------------------
TcasGr17        ------------------------------------------------------------
TcasGr150       ------------------------------------------------------------
TcasGr32        QHIFVKSRPSPATIISNVTVISWQAVCTFYNILLCDSVAHTAGELLGAVYSLDLELKHIE 598
TcasGr54        ------------------------------------------------------------
TcasGr37        ------------------------------------------------------------
TcasGr53        ------------------------------------------------------------
TcasGr5         ---------------------------TFNSVFICDLIEQKVKNIQMLVYQN------EA 471
TcasGr12        ------------------------------------------------------------
TcasGr13        ------------------------------------------------------------
TcasGr104       ------------------------------------------------------------
TcasGr45        ------------------------------------------------------------
TcasGr98        ------------------------------------------------------------
TcasGr52        ------------------------------------------------------------
TcasGr105       ------------------------------------------------------------
TcasGr4         ------------------------------------------------------------
TcasGr44        ------------------------------------------------------------
AgamGr9a        ------------------------------------------------------------
AgamGr9c        ------------------------------------------------------------
AgamGr9b        ------------------------------------------------------------
AgamGr9d        ------------------------------------------------------------
AgamGr9e        ------------------------------------------------------------
AgamGr9f        ------------------------------------------------------------
AgamGr9g        ------------------------------------------------------------
```

```
AgamGr9i        ------------------------------------------------------------
AgamGr9h        ------------------------------------------------------------
AgamGr9j        ------------------------------------------------------------
AgamGr9l        ------------------------------------------------------------
AgamGr9k        ------------------------------------------------------------
AgamGr9m        ------------------------------------------------------------
AgamGr9n        ------------------------------------------------------------
AgamGr10        ------------------------------------------------------------
AgamGr11        ------------------------------------------------------------
DmelGr23aA      ------------------------------------------------------------
DmelGr23aB      ------------------------------------------------------------
DmelGr39aA      ------------------------------------------------------------
DmelGr39aB      ------------------------------------------------------------
DmelGr39aC      ------------------------------------------------------------
DmelGr39aD      ------------------------------------------------------------
DmelGr98c       ------------------------------------------------------------
DmelGr98d       ------------------------------------------------------------
DmelGr98b       ------------------------------------------------------------
AgamGr51        ------------------------------------------------------------
AgamGr52        ------------------------------------------------------------
DmelGr94a       ------------------------------------------------------------
DmelGr97a       ------------------------------------------------------------
DmelGr93b       ------------------------------------------------------------
DmelGr93c       ------------------------------------------------------------
DmelGr22d       ------------------------------------------------------------
DmelGr22e       ------------------------------------------------------------
DmelGr22a       ------------------------------------------------------------
DmelGr22b       ------------------------------------------------------------
DmelGr22c       ------------------------------------------------------------
DmelGr22f       ------------------------------------------------------------
DmelGr36a       ------------------------------------------------------------
DmelGr36c       ------------------------------------------------------------
DmelGr36b       ------------------------------------------------------------
DmelGr59d       ------------------------------------------------------------
DmelGr59a       ------------------------------------------------------------
DmelGr59b       ------------------------------------------------------------
AgamGr26        ------------------------------------------------------------
AgamGr27        ------------------------------------------------------------
AgamGr29        ------------------------------------------------------------
AgamGr31        ------------------------------------------------------------
AgamGr30        ------------------------------------------------------------
AgamGr32a       ------------------------------------------------------------
TcasGr2         ------------------------------------------------------------
TcasGr9         ------------------------------------------------------------
TcasGr7         ------------------------------------------------------------
TcasGr30        ------------------------------------------------------------
TcasGr29        ------------------------------------------------------------
AaegGr64f       ------------------------------------------------------------
AaegGr64a3      ------------------------------------------------------------
AgamGr14        ------------------------------------------------------------
AgamGr18        ------------------------------------------------------------
AaegGr64d       ------------------------------------------------------------
AgamGr17        ------------------------------------------------------------
AaegGr64c       ------------------------------------------------------------
DmelGr64c       ------------------------------------------------------------
DmelGr64d       ------------------------------------------------------------
AgamGr20        ------------------------------------------------------------
AaegGr61a       ------------------------------------------------------------
AgamGr21        ------------------------------------------------------------
AaegGr64a       ------------------------------------------------------------
DmelGr64a       ------------------------------------------------------------
DmelGr61a       ------------------------------------------------------------
AgamGr15        ------------------------------------------------------------
AaegGr1         ------------------------------------------------------------
DmelGr5a        ------------------------------------------------------------
DmelGr64f       ------------------------------------------------------------
DmelGr64e       ------------------------------------------------------------
AmelGr1         ------------------------------------------------------------
AmelGr2         ------------------------------------------------------------
AgamGr16        ------------------------------------------------------------
```

```
AaegGr64e       ------------------------------------------------------------
TCasGr6         ------------------------------------------------------------
TcasGr26        ------------------------------------------------------------
TcasGr33        ------------------------------------------------------------
AgamGr23        ------------------------------------------------------------
AaegGr21a       ------------------------------------------------------------
TcasGr10        ------------------------------------------------------------
TcasGr39        ------------------------------------------------------------
AgamGr22        ------------------------------------------------------------
AaegGr21b       ------------------------------------------------------------
DmelGr21a       ------------------------------------------------------------
AgamGr24        ------------------------------------------------------------
AaegGr63a       ------------------------------------------------------------
DmelGr63a       ------------------------------------------------------------
TcasGr3         ------------------------------------------------------------
DmelOr83b       ------------------------------------------------------------
AgamOr7         ------------------------------------------------------------
HvirOr2         ------------------------------------------------------------
TcasOr16        ------------------------------------------------------------
TcasOr22        ------------------------------------------------------------
AmelOr2         ------------------------------------------------------------
TcasGr31        ------------------------------------------------------------
TcasGr35        ------------------------------------------------------------
DmelGr59c       ------------------------------------------------------------
DmelGr92a       ------------------------------------------------------------
DmelGr28a       ------------------------------------------------------------
DmelGr64b       ------------------------------------------------------------
TcasGr71        ------------------------------------------------------------
AgamGr19        ------------------------------------------------------------
AaegGr64b       ------------------------------------------------------------
DmelGr93d       ------------------------------------------------------------
TcasGr34        ------------------------------------------------------------
TcasGr43        ------------------------------------------------------------
TcasGr47        ------------------------------------------------------------
AmelGr3         ------------------------------------------------------------
AgamGr32b       ------------------------------------------------------------
AgamGr50        ------------------------------------------------------------
TcasGr20        ------------------------------------------------------------
TcasGr79        ------------------------------------------------------------
TcasGr15        ------------------------------------------------------------
TcasGr60        ------------------------------------------------------------
AgamGr38        ------------------------------------------------------------
TcasGr51        ------------------------------------------------------------
DmelGr39b       ------------------------------------------------------------
AgamGr28        ------------------------------------------------------------
TcasGr61        ------------------------------------------------------------
TcasGr50        ------------------------------------------------------------
AgamGr12        ------------------------------------------------------------
AgamGr36        ------------------------------------------------------------
AgamGr48        ------------------------------------------------------------
AgamGr49a       ------------------------------------------------------------
AgamGr49b       ------------------------------------------------------------
DmelGr2a        ------------------------------------------------------------
DmelGr32a       ------------------------------------------------------------
DmelGr68a       ------------------------------------------------------------
AmelGr7         ------------------------------------------------------------
AmelGr8         ------------------------------------------------------------
AmelGr9         ------------------------------------------------------------
TcasGr57        ------------------------------------------------------------
TcasGr41        ------------------------------------------------------------
AgamGr34        ------------------------------------------------------------
AgamGr35        ------------------------------------------------------------
AgamGr39        ------------------------------------------------------------
AgamGr40        ------------------------------------------------------------
AaegGr28d       ------------------------------------------------------------
AgamGr42        ------------------------------------------------------------
AgamGr41        ------------------------------------------------------------
AgamGr43        ------------------------------------------------------------
DmelGr33a       ------------------------------------------------------------
AgamGr44        ------------------------------------------------------------
```

```
AgamGr13        ------------------------------------------------------------
AgamGr45        ------------------------------------------------------------
AgamGr46        ------------------------------------------------------------
Dmelr93a        ------------------------------------------------------------
AmelGr6         ------------------------------------------------------------
AmelGr10        ------------------------------------------------------------
TcasGr28        ------------------------------------------------------------
TcasGr40        ------------------------------------------------------------
AgamGr3         ------------------------------------------------------------
AgamGr4         ------------------------------------------------------------
AgamGr6         ------------------------------------------------------------
AgamGr7         ------------------------------------------------------------
AgamGr47        ------------------------------------------------------------
DmelGr8a        ------------------------------------------------------------
AgamGr5         ------------------------------------------------------------
AgamGr8         ------------------------------------------------------------
DmelGr9a        ------------------------------------------------------------
DmelGr59f       ------------------------------------------------------------
DmelGr77a       ------------------------------------------------------------
DmelGr85a       ------------------------------------------------------------
TcasGr27        ------------------------------------------------------------
TcasGr48        ------------------------------------------------------------
AgamGr1         ------------------------------------------------------------
DmelGr10a       ------------------------------------------------------------
DmelGr59e       ------------------------------------------------------------
DmelGr10b       ------------------------------------------------------------
DmelGr89a       ------------------------------------------------------------
DmelGr57a       ------------------------------------------------------------
DmelGr47b       ------------------------------------------------------------
DmelGr58a       ------------------------------------------------------------
DmelGr58b       ------------------------------------------------------------
DmelGr98a       ------------------------------------------------------------
AaegGr93a       ------------------------------------------------------------
TcasGr19        ------------------------------------------------------------
DmelGr47a       ------------------------------------------------------------
DmelGr58c       ------------------------------------------------------------


TcasGr38        ------------------------------------------------------------
TcasGr46        SAFSSEKIVWLHLVYISFLGIICAADVFYICHVCYATIQEVRAKIYFFYLVKFYKVSKSG 1042
TcasGr1         ------------------------------------------------------------
TcasGr123       ------------------------------------------------------------
TcasGr62        ------------------------------------------------------------
TcasGr25        ------------------------------------------------------------
TcasGr59        ------------------------------------------------------------
AgamGr2         ------------------------------------------------------------
AaegGr66a       ------------------------------------------------------------
DmelGr66a       ------------------------------------------------------------
TcasGr11        ------------------------------------------------------------
TcasGr55        ------------------------------------------------------------
TcasGr21        ------------------------------------------------------------
TcasGr56        ------------------------------------------------------------
AaegGr          ------------------------------------------------------------
AaegGr28a       ------------------------------------------------------------
AgamGr33        ------------------------------------------------------------
DmelGr28bB      ------------------------------------------------------------
DmelGr28bC      ------------------------------------------------------------
DmelGr28bD      ------------------------------------------------------------
DmelGr28bA      ------------------------------------------------------------
DmelGr28bE      ------------------------------------------------------------
AgamGr25        ------------------------------------------------------------
AaegGr43a       ------------------------------------------------------------
DmelGr43a       ------------------------------------------------------------
AgamGr37b       ------------------------------------------------------------
AgamGr37d       ------------------------------------------------------------
AgamGr37a       ------------------------------------------------------------
AgamGr37c       ------------------------------------------------------------
AgamGr37e       ------------------------------------------------------------
AgamGr37f       ------------------------------------------------------------
AaegGr28c       ------------------------------------------------------------
```

```
AaegGr28b       ------------------------------------------------------------
AaegGr28e       ------------------------------------------------------------
TcasGr14        ------------------------------------------------------------
TcasGr49        ------------------------------------------------------------
AmelGr4         ------------------------------------------------------------
AmelGr5         ------------------------------------------------------------
TcasGr16        ------------------------------------------------------------
TcasGr22        ------------------------------------------------------------
TcasGr17        ------------------------------------------------------------
TcasGr150       ------------------------------------------------------------
TcasGr32        NFNDFVTALKDNVPVFAAARFYAINRSTIFRMFNAIVTFLIVMVQFETNYSQP------- 651
TcasGr54        ------------------------------------------------------------
TcasGr37        ------------------------------------------------------------
TcasGr53        ------------------------------------------------------------
TcasGr5         EEVKILLDVINHFPHFTAARFFDLNRKTILGVLNALFTFLIVVVQFENLTS--------- 522
TcasGr12        ------------------------------------------------------------
TcasGr13        ------------------------------------------------------------
TcasGr104       ------------------------------------------------------------
TcasGr45        ------------------------------------------------------------
TcasGr98        ------------------------------------------------------------
TcasGr52        ------------------------------------------------------------
TcasGr105       ------------------------------------------------------------
TcasGr4         ------------------------------------------------------------
TcasGr44        ------------------------------------------------------------
AgamGr9a        ------------------------------------------------------------
AgamGr9c        ------------------------------------------------------------
AgamGr9b        ------------------------------------------------------------
AgamGr9d        ------------------------------------------------------------
AgamGr9e        ------------------------------------------------------------
AgamGr9f        ------------------------------------------------------------
AgamGr9g        ------------------------------------------------------------
AgamGr9i        ------------------------------------------------------------
AgamGr9h        ------------------------------------------------------------
AgamGr9j        ------------------------------------------------------------
AgamGr9l        ------------------------------------------------------------
AgamGr9k        ------------------------------------------------------------
AgamGr9m        ------------------------------------------------------------
AgamGr9n        ------------------------------------------------------------
AgamGr10        ------------------------------------------------------------
AgamGr11        ------------------------------------------------------------
DmelGr23aA      ------------------------------------------------------------
DmelGr23aB      ------------------------------------------------------------
DmelGr39aA      ------------------------------------------------------------
DmelGr39aB      ------------------------------------------------------------
DmelGr39aC      ------------------------------------------------------------
DmelGr39aD      ------------------------------------------------------------
DmelGr98c       ------------------------------------------------------------
DmelGr98d       ------------------------------------------------------------
DmelGr98b       ------------------------------------------------------------
AgamGr51        ------------------------------------------------------------
AgamGr52        ------------------------------------------------------------
DmelGr94a       ------------------------------------------------------------
DmelGr97a       ------------------------------------------------------------
DmelGr93b       ------------------------------------------------------------
DmelGr93c       ------------------------------------------------------------
DmelGr22d       ------------------------------------------------------------
DmelGr22e       ------------------------------------------------------------
DmelGr22a       ------------------------------------------------------------
DmelGr22b       ------------------------------------------------------------
DmelGr22c       ------------------------------------------------------------
DmelGr22f       ------------------------------------------------------------
DmelGr36a       ------------------------------------------------------------
DmelGr36c       ------------------------------------------------------------
DmelGr36b       ------------------------------------------------------------
DmelGr59d       ------------------------------------------------------------
DmelGr59a       ------------------------------------------------------------
DmelGr59b       ------------------------------------------------------------
AgamGr26        ------------------------------------------------------------
AgamGr27        ------------------------------------------------------------
AgamGr29        ------------------------------------------------------------
```

```
AgamGr31        ------------------------------------------------------------
AgamGr30        ------------------------------------------------------------
AgamGr32a       ------------------------------------------------------------
TcasGr2         ------------------------------------------------------------
TcasGr9         ------------------------------------------------------------
TcasGr7         ------------------------------------------------------------
TcasGr30        ------------------------------------------------------------
TcasGr29        ------------------------------------------------------------
AaegGr64f       ------------------------------------------------------------
AaegGr64a3      ------------------------------------------------------------
AgamGr14        ------------------------------------------------------------
AgamGr18        ------------------------------------------------------------
AaegGr64d       ------------------------------------------------------------
AgamGr17        ------------------------------------------------------------
AaegGr64c       ------------------------------------------------------------
DmelGr64c       ------------------------------------------------------------
DmelGr64d       ------------------------------------------------------------
AgamGr20        ------------------------------------------------------------
AaegGr61a       ------------------------------------------------------------
AgamGr21        ------------------------------------------------------------
AaegGr64a       ------------------------------------------------------------
DmelGr64a       ------------------------------------------------------------
DmelGr61a       ------------------------------------------------------------
AgamGr15        ------------------------------------------------------------
AaegGr1         ------------------------------------------------------------
DmelGr5a        ------------------------------------------------------------
DmelGr64f       ------------------------------------------------------------
DmelGr64e       ------------------------------------------------------------
AmelGr1         ------------------------------------------------------------
AmelGr2         ------------------------------------------------------------
AgamGr16        ------------------------------------------------------------
AaegGr64e       ------------------------------------------------------------
TCasGr6         ------------------------------------------------------------
TcasGr26        ------------------------------------------------------------
TcasGr33        ------------------------------------------------------------
AgamGr23        ------------------------------------------------------------
AaegGr21a       ------------------------------------------------------------
TcasGr10        ------------------------------------------------------------
TcasGr39        ------------------------------------------------------------
AgamGr22        ------------------------------------------------------------
AaegGr21b       ------------------------------------------------------------
DmelGr21a       ------------------------------------------------------------
AgamGr24        ------------------------------------------------------------
AaegGr63a       ------------------------------------------------------------
DmelGr63a       ------------------------------------------------------------
TcasGr3         ------------------------------------------------------------
DmelOr83b       ------------------------------------------------------------
AgamOr7         ------------------------------------------------------------
HvirOr2         ------------------------------------------------------------
TcasOr16        ------------------------------------------------------------
TcasOr22        ------------------------------------------------------------
AmelOr2         ------------------------------------------------------------
TcasGr31        ------------------------------------------------------------
TcasGr35        ------------------------------------------------------------
DmelGr59c       ------------------------------------------------------------
DmelGr92a       ------------------------------------------------------------
DmelGr28a       ------------------------------------------------------------
DmelGr64b       ------------------------------------------------------------
TcasGr71        ------------------------------------------------------------
AgamGr19        ------------------------------------------------------------
AaegGr64b       ------------------------------------------------------------
DmelGr93d       ------------------------------------------------------------
TcasGr34        ------------------------------------------------------------
TcasGr43        ------------------------------------------------------------
TcasGr47        ------------------------------------------------------------
AmelGr3         ------------------------------------------------------------
AgamGr32b       ------------------------------------------------------------
AgamGr50        ------------------------------------------------------------
TcasGr20        ------------------------------------------------------------
TcasGr79        ------------------------------------------------------------
```

```
TcasGr15        ------------------------------------------------------------
TcasGr60        ------------------------------------------------------------
AgamGr38        ------------------------------------------------------------
TcasGr51        ------------------------------------------------------------
DmelGr39b       ------------------------------------------------------------
AgamGr28        ------------------------------------------------------------
TcasGr61        ------------------------------------------------------------
TcasGr50        ------------------------------------------------------------
AgamGr12        ------------------------------------------------------------
AgamGr36        ------------------------------------------------------------
AgamGr48        ------------------------------------------------------------
AgamGr49a       ------------------------------------------------------------
AgamGr49b       ------------------------------------------------------------
DmelGr2a        ------------------------------------------------------------
DmelGr32a       ------------------------------------------------------------
DmelGr68a       ------------------------------------------------------------
AmelGr7         ------------------------------------------------------------
AmelGr8         ------------------------------------------------------------
AmelGr9         ------------------------------------------------------------
TcasGr57        ------------------------------------------------------------
TcasGr41        ------------------------------------------------------------
AgamGr34        ------------------------------------------------------------
AgamGr35        ------------------------------------------------------------
AgamGr39        ------------------------------------------------------------
AgamGr40        ------------------------------------------------------------
AaegGr28d       ------------------------------------------------------------
AgamGr42        ------------------------------------------------------------
AgamGr41        ------------------------------------------------------------
AgamGr43        ------------------------------------------------------------
DmelGr33a       ------------------------------------------------------------
AgamGr44        ------------------------------------------------------------
AgamGr13        ------------------------------------------------------------
AgamGr45        ------------------------------------------------------------
AgamGr46        ------------------------------------------------------------
Dmelr93a        ------------------------------------------------------------
AmelGr6         ------------------------------------------------------------
AmelGr10        ------------------------------------------------------------
TcasGr28        ------------------------------------------------------------
TcasGr40        ------------------------------------------------------------
AgamGr3         ------------------------------------------------------------
AgamGr4         ------------------------------------------------------------
AgamGr6         ------------------------------------------------------------
AgamGr7         ------------------------------------------------------------
AgamGr47        ------------------------------------------------------------
DmelGr8a        ------------------------------------------------------------
AgamGr5         ------------------------------------------------------------
AgamGr8         ------------------------------------------------------------
DmelGr9a        ------------------------------------------------------------
DmelGr59f       ------------------------------------------------------------
DmelGr77a       ------------------------------------------------------------
DmelGr85a       ------------------------------------------------------------
TcasGr27        ------------------------------------------------------------
TcasGr48        ------------------------------------------------------------
AgamGr1         ------------------------------------------------------------
DmelGr10a       ------------------------------------------------------------
DmelGr59e       ------------------------------------------------------------
DmelGr10b       ------------------------------------------------------------
DmelGr89a       ------------------------------------------------------------
DmelGr57a       ------------------------------------------------------------
DmelGr47b       ------------------------------------------------------------
DmelGr58a       ------------------------------------------------------------
DmelGr58b       ------------------------------------------------------------
DmelGr98a       ------------------------------------------------------------
AaegGr93a       ------------------------------------------------------------
TcasGr19        ------------------------------------------------------------
DmelGr47a       ------------------------------------------------------------
DmelGr58c       ------------------------------------------------------------


TcasGr38        ------------------------------------------------------------
```

```
TcasGr46        ELIHKIETNEHEIIDKIEMFSLQILNERAEFNAAGFFPIDYTLVFSKFTSSIRMLLIQGQ 1102
TcasGr1         ------------------------------------------------------------
TcasGr123       ------------------------------------------------------------
TcasGr62        ------------------------------------------------------------
TcasGr25        ------------------------------------------------------------
TcasGr59        ------------------------------------------------------------
AgamGr2         ------------------------------------------------------------
AaegGr66a       ------------------------------------------------------------
DmelGr66a       ------------------------------------------------------------
TcasGr11        ------------------------------------------------------------
TcasGr55        ------------------------------------------------------------
TcasGr21        ------------------------------------------------------------
TcasGr56        ------------------------------------------------------------
AaegGr          ------------------------------------------------------------
AaegGr28a       ------------------------------------------------------------
AgamGr33        ------------------------------------------------------------
DmelGr28bB      ------------------------------------------------------------
DmelGr28bC      ------------------------------------------------------------
DmelGr28bD      ------------------------------------------------------------
DmelGr28bA      ------------------------------------------------------------
DmelGr28bE      ------------------------------------------------------------
AgamGr25        ------------------------------------------------------------
AaegGr43a       ------------------------------------------------------------
DmelGr43a       ------------------------------------------------------------
AgamGr37b       ------------------------------------------------------------
AgamGr37d       ------------------------------------------------------------
AgamGr37a       ------------------------------------------------------------
AgamGr37c       ------------------------------------------------------------
AgamGr37e       ------------------------------------------------------------
AgamGr37f       ------------------------------------------------------------
AaegGr28c       ------------------------------------------------------------
AaegGr28b       ------------------------------------------------------------
AaegGr28e       ------------------------------------------------------------
TcasGr14        ------------------------------------------------------------
TcasGr49        ------------------------------------------------------------
AmelGr4         ------------------------------------------------------------
AmelGr5         ------------------------------------------------------------
TcasGr16        ------------------------------------------------------------
TcasGr22        ------------------------------------------------------------
TcasGr17        ------------------------------------------------------------
TcasGr150       ------------------------------------------------------------
TcasGr32        ------------------------------------------------------------
TcasGr54        ------------------------------------------------------------
TcasGr37        ------------------------------------------------------------
TcasGr53        ------------------------------------------------------------
TcasGr5         ------------------------------------------------------------
TcasGr12        ------------------------------------------------------------
TcasGr13        ------------------------------------------------------------
TcasGr104       ------------------------------------------------------------
TcasGr45        ------------------------------------------------------------
TcasGr98        ------------------------------------------------------------
TcasGr52        ------------------------------------------------------------
TcasGr105       ------------------------------------------------------------
TcasGr4         ------------------------------------------------------------
TcasGr44        ------------------------------------------------------------
AgamGr9a        ------------------------------------------------------------
AgamGr9c        ------------------------------------------------------------
AgamGr9b        ------------------------------------------------------------
AgamGr9d        ------------------------------------------------------------
AgamGr9e        ------------------------------------------------------------
AgamGr9f        ------------------------------------------------------------
AgamGr9g        ------------------------------------------------------------
AgamGr9i        ------------------------------------------------------------
AgamGr9h        ------------------------------------------------------------
AgamGr9j        ------------------------------------------------------------
AgamGr9l        ------------------------------------------------------------
AgamGr9k        ------------------------------------------------------------
AgamGr9m        ------------------------------------------------------------
AgamGr9n        ------------------------------------------------------------
AgamGr10        ------------------------------------------------------------
```

```
AgamGr11        ------------------------------------------------------------
DmelGr23aA      ------------------------------------------------------------
DmelGr23aB      ------------------------------------------------------------
DmelGr39aA      ------------------------------------------------------------
DmelGr39aB      ------------------------------------------------------------
DmelGr39aC      ------------------------------------------------------------
DmelGr39aD      ------------------------------------------------------------
DmelGr98c       ------------------------------------------------------------
DmelGr98d       ------------------------------------------------------------
DmelGr98b       ------------------------------------------------------------
AgamGr51        ------------------------------------------------------------
AgamGr52        ------------------------------------------------------------
DmelGr94a       ------------------------------------------------------------
DmelGr97a       ------------------------------------------------------------
DmelGr93b       ------------------------------------------------------------
DmelGr93c       ------------------------------------------------------------
DmelGr22d       ------------------------------------------------------------
DmelGr22e       ------------------------------------------------------------
DmelGr22a       ------------------------------------------------------------
DmelGr22b       ------------------------------------------------------------
DmelGr22c       ------------------------------------------------------------
DmelGr22f       ------------------------------------------------------------
DmelGr36a       ------------------------------------------------------------
DmelGr36c       ------------------------------------------------------------
DmelGr36b       ------------------------------------------------------------
DmelGr59d       ------------------------------------------------------------
DmelGr59a       ------------------------------------------------------------
DmelGr59b       ------------------------------------------------------------
AgamGr26        ------------------------------------------------------------
AgamGr27        ------------------------------------------------------------
AgamGr29        ------------------------------------------------------------
AgamGr31        ------------------------------------------------------------
AgamGr30        ------------------------------------------------------------
AgamGr32a       ------------------------------------------------------------
TcasGr2         ------------------------------------------------------------
TcasGr9         ------------------------------------------------------------
TcasGr7         ------------------------------------------------------------
TcasGr30        ------------------------------------------------------------
TcasGr29        ------------------------------------------------------------
AaegGr64f       ------------------------------------------------------------
AaegGr64a3      ------------------------------------------------------------
AgamGr14        ------------------------------------------------------------
AgamGr18        ------------------------------------------------------------
AaegGr64d       ------------------------------------------------------------
AgamGr17        ------------------------------------------------------------
AaegGr64c       ------------------------------------------------------------
DmelGr64c       ------------------------------------------------------------
DmelGr64d       ------------------------------------------------------------
AgamGr20        ------------------------------------------------------------
AaegGr61a       ------------------------------------------------------------
AgamGr21        ------------------------------------------------------------
AaegGr64a       ------------------------------------------------------------
DmelGr64a       ------------------------------------------------------------
DmelGr61a       ------------------------------------------------------------
AgamGr15        ------------------------------------------------------------
AaegGr1         ------------------------------------------------------------
DmelGr5a        ------------------------------------------------------------
DmelGr64f       ------------------------------------------------------------
DmelGr64e       ------------------------------------------------------------
AmelGr1         ------------------------------------------------------------
AmelGr2         ------------------------------------------------------------
AgamGr16        ------------------------------------------------------------
AaegGr64e       ------------------------------------------------------------
TCasGr6         ------------------------------------------------------------
TcasGr26        ------------------------------------------------------------
TcasGr33        ------------------------------------------------------------
AgamGr23        ------------------------------------------------------------
AaegGr21a       ------------------------------------------------------------
TcasGr10        ------------------------------------------------------------
TcasGr39        ------------------------------------------------------------
```

```
AgamGr22         ------------------------------------------------------------
AaegGr21b        ------------------------------------------------------------
DmelGr21a        ------------------------------------------------------------
AgamGr24         ------------------------------------------------------------
AaegGr63a        ------------------------------------------------------------
DmelGr63a        ------------------------------------------------------------
TcasGr3          ------------------------------------------------------------
DmelOr83b        ------------------------------------------------------------
AgamOr7          ------------------------------------------------------------
HvirOr2          ------------------------------------------------------------
TcasOr16         ------------------------------------------------------------
TcasOr22         ------------------------------------------------------------
AmelOr2          ------------------------------------------------------------
TcasGr31         ------------------------------------------------------------
TcasGr35         ------------------------------------------------------------
DmelGr59c        ------------------------------------------------------------
DmelGr92a        ------------------------------------------------------------
DmelGr28a        ------------------------------------------------------------
DmelGr64b        ------------------------------------------------------------
TcasGr71         ------------------------------------------------------------
AgamGr19         ------------------------------------------------------------
AaegGr64b        ------------------------------------------------------------
DmelGr93d        ------------------------------------------------------------
TcasGr34         ------------------------------------------------------------
TcasGr43         ------------------------------------------------------------
TcasGr47         ------------------------------------------------------------
AmelGr3          ------------------------------------------------------------
AgamGr32b        ------------------------------------------------------------
AgamGr50         ------------------------------------------------------------
TcasGr20         ------------------------------------------------------------
TcasGr79         ------------------------------------------------------------
TcasGr15         ------------------------------------------------------------
TcasGr60         ------------------------------------------------------------
AgamGr38         ------------------------------------------------------------
TcasGr51         ------------------------------------------------------------
DmelGr39b        ------------------------------------------------------------
AgamGr28         ------------------------------------------------------------
TcasGr61         ------------------------------------------------------------
TcasGr50         ------------------------------------------------------------
AgamGr12         ------------------------------------------------------------
AgamGr36         ------------------------------------------------------------
AgamGr48         ------------------------------------------------------------
AgamGr49a        ------------------------------------------------------------
AgamGr49b        ------------------------------------------------------------
DmelGr2a         ------------------------------------------------------------
DmelGr32a        ------------------------------------------------------------
DmelGr68a        ------------------------------------------------------------
AmelGr7          ------------------------------------------------------------
AmelGr8          ------------------------------------------------------------
AmelGr9          ------------------------------------------------------------
TcasGr57         ------------------------------------------------------------
TcasGr41         ------------------------------------------------------------
AgamGr34         ------------------------------------------------------------
AgamGr35         ------------------------------------------------------------
AgamGr39         ------------------------------------------------------------
AgamGr40         ------------------------------------------------------------
AaegGr28d        ------------------------------------------------------------
AgamGr42         ------------------------------------------------------------
AgamGr41         ------------------------------------------------------------
AgamGr43         ------------------------------------------------------------
DmelGr33a        ------------------------------------------------------------
AgamGr44         ------------------------------------------------------------
AgamGr13         ------------------------------------------------------------
AgamGr45         ------------------------------------------------------------
AgamGr46         ------------------------------------------------------------
Dmelr93a         ------------------------------------------------------------
AmelGr6          ------------------------------------------------------------
AmelGr10         ------------------------------------------------------------
TcasGr28         ------------------------------------------------------------
TcasGr40         ------------------------------------------------------------
```

```
AgamGr3         ------------------------------------------------------------
AgamGr4         ------------------------------------------------------------
AgamGr6         ------------------------------------------------------------
AgamGr7         ------------------------------------------------------------
AgamGr47        ------------------------------------------------------------
DmelGr8a        ------------------------------------------------------------
AgamGr5         ------------------------------------------------------------
AgamGr8         ------------------------------------------------------------
DmelGr9a        ------------------------------------------------------------
DmelGr59f       ------------------------------------------------------------
DmelGr77a       ------------------------------------------------------------
DmelGr85a       ------------------------------------------------------------
TcasGr27        ------------------------------------------------------------
TcasGr48        ------------------------------------------------------------
AgamGr1         ------------------------------------------------------------
DmelGr10a       ------------------------------------------------------------
DmelGr59e       ------------------------------------------------------------
DmelGr10b       ------------------------------------------------------------
DmelGr89a       ------------------------------------------------------------
DmelGr57a       ------------------------------------------------------------
DmelGr47b       ------------------------------------------------------------
DmelGr58a       ------------------------------------------------------------
DmelGr58b       ------------------------------------------------------------
DmelGr98a       ------------------------------------------------------------
AaegGr93a       ------------------------------------------------------------
TcasGr19        ------------------------------------------------------------
DmelGr47a       ------------------------------------------------------------
DmelGr58c       ------------------------------------------------------------


TcasGr38        ------------------------------------------------------------
TcasGr46        IFGLITFGCSNRCFFPSKIRICWNVLLNCVYLFLCGFCVYEFASDERIKLVIKAIIIMIL 1162
TcasGr1         ------------------------------------------------------------
TcasGr123       ------------------------------------------------------------
TcasGr62        ------------------------------------------------------------
TcasGr25        ------------------------------------------------------------
TcasGr59        ------------------------------------------------------------
AgamGr2         ------------------------------------------------------------
AaegGr66a       ------------------------------------------------------------
DmelGr66a       ------------------------------------------------------------
TcasGr11        ------------------------------------------------------------
TcasGr55        ------------------------------------------------------------
TcasGr21        ------------------------------------------------------------
TcasGr56        ------------------------------------------------------------
AaegGr          ------------------------------------------------------------
AaegGr28a       ------------------------------------------------------------
AgamGr33        ------------------------------------------------------------
DmelGr28bB      ------------------------------------------------------------
DmelGr28bC      ------------------------------------------------------------
DmelGr28bD      ------------------------------------------------------------
DmelGr28bA      ------------------------------------------------------------
DmelGr28bE      ------------------------------------------------------------
AgamGr25        ------------------------------------------------------------
AaegGr43a       ------------------------------------------------------------
DmelGr43a       ------------------------------------------------------------
AgamGr37b       ------------------------------------------------------------
AgamGr37d       ------------------------------------------------------------
AgamGr37a       ------------------------------------------------------------
AgamGr37c       ------------------------------------------------------------
AgamGr37e       ------------------------------------------------------------
AgamGr37f       ------------------------------------------------------------
AaegGr28c       ------------------------------------------------------------
AaegGr28b       ------------------------------------------------------------
AaegGr28e       ------------------------------------------------------------
TcasGr14        ------------------------------------------------------------
TcasGr49        ------------------------------------------------------------
AmelGr4         ------------------------------------------------------------
AmelGr5         ------------------------------------------------------------
TcasGr16        ------------------------------------------------------------
TcasGr22        ------------------------------------------------------------
```

```
TcasGr17        ------------------------------------------------------------
TcasGr150       ------------------------------------------------------------
TcasGr32        ------------------------------------------------------------
TcasGr54        ------------------------------------------------------------
TcasGr37        ------------------------------------------------------------
TcasGr53        ------------------------------------------------------------
TcasGr5         ------------------------------------------------------------
TcasGr12        ------------------------------------------------------------
TcasGr13        ------------------------------------------------------------
TcasGr104       ------------------------------------------------------------
TcasGr45        ------------------------------------------------------------
TcasGr98        ------------------------------------------------------------
TcasGr52        ------------------------------------------------------------
TcasGr105       ------------------------------------------------------------
TcasGr4         ------------------------------------------------------------
TcasGr44        ------------------------------------------------------------
AgamGr9a        ------------------------------------------------------------
AgamGr9c        ------------------------------------------------------------
AgamGr9b        ------------------------------------------------------------
AgamGr9d        ------------------------------------------------------------
AgamGr9e        ------------------------------------------------------------
AgamGr9f        ------------------------------------------------------------
AgamGr9g        ------------------------------------------------------------
AgamGr9i        ------------------------------------------------------------
AgamGr9h        ------------------------------------------------------------
AgamGr9j        ------------------------------------------------------------
AgamGr9l        ------------------------------------------------------------
AgamGr9k        ------------------------------------------------------------
AgamGr9m        ------------------------------------------------------------
AgamGr9n        ------------------------------------------------------------
AgamGr10        ------------------------------------------------------------
AgamGr11        ------------------------------------------------------------
DmelGr23aA      ------------------------------------------------------------
DmelGr23aB      ------------------------------------------------------------
DmelGr39aA      ------------------------------------------------------------
DmelGr39aB      ------------------------------------------------------------
DmelGr39aC      ------------------------------------------------------------
DmelGr39aD      ------------------------------------------------------------
DmelGr98c       ------------------------------------------------------------
DmelGr98d       ------------------------------------------------------------
DmelGr98b       ------------------------------------------------------------
AgamGr51        ------------------------------------------------------------
AgamGr52        ------------------------------------------------------------
DmelGr94a       ------------------------------------------------------------
DmelGr97a       ------------------------------------------------------------
DmelGr93b       ------------------------------------------------------------
DmelGr93c       ------------------------------------------------------------
DmelGr22d       ------------------------------------------------------------
DmelGr22e       ------------------------------------------------------------
DmelGr22a       ------------------------------------------------------------
DmelGr22b       ------------------------------------------------------------
DmelGr22c       ------------------------------------------------------------
DmelGr22f       ------------------------------------------------------------
DmelGr36a       ------------------------------------------------------------
DmelGr36c       ------------------------------------------------------------
DmelGr36b       ------------------------------------------------------------
DmelGr59d       ------------------------------------------------------------
DmelGr59a       ------------------------------------------------------------
DmelGr59b       ------------------------------------------------------------
AgamGr26        ------------------------------------------------------------
AgamGr27        ------------------------------------------------------------
AgamGr29        ------------------------------------------------------------
AgamGr31        ------------------------------------------------------------
AgamGr30        ------------------------------------------------------------
AgamGr32a       ------------------------------------------------------------
TcasGr2         ------------------------------------------------------------
TcasGr9         ------------------------------------------------------------
TcasGr7         ------------------------------------------------------------
TcasGr30        ------------------------------------------------------------
TcasGr29        ------------------------------------------------------------
```

```
AaegGr64f     ------------------------------------------------------------
AaegGr64a3    ------------------------------------------------------------
AgamGr14      ------------------------------------------------------------
AgamGr18      ------------------------------------------------------------
AaegGr64d     ------------------------------------------------------------
AgamGr17      ------------------------------------------------------------
AaegGr64c     ------------------------------------------------------------
DmelGr64c     ------------------------------------------------------------
DmelGr64d     ------------------------------------------------------------
AgamGr20      ------------------------------------------------------------
AaegGr61a     ------------------------------------------------------------
AgamGr21      ------------------------------------------------------------
AaegGr64a     ------------------------------------------------------------
DmelGr64a     ------------------------------------------------------------
DmelGr61a     ------------------------------------------------------------
AgamGr15      ------------------------------------------------------------
AaegGr1       ------------------------------------------------------------
DmelGr5a      ------------------------------------------------------------
DmelGr64f     ------------------------------------------------------------
DmelGr64e     ------------------------------------------------------------
AmelGr1       ------------------------------------------------------------
AmelGr2       ------------------------------------------------------------
AgamGr16      ------------------------------------------------------------
AaegGr64e     ------------------------------------------------------------
TCasGr6       ------------------------------------------------------------
TcasGr26      ------------------------------------------------------------
TcasGr33      ------------------------------------------------------------
AgamGr23      ------------------------------------------------------------
AaegGr21a     ------------------------------------------------------------
TcasGr10      ------------------------------------------------------------
TcasGr39      ------------------------------------------------------------
AgamGr22      ------------------------------------------------------------
AaegGr21b     ------------------------------------------------------------
DmelGr21a     ------------------------------------------------------------
AgamGr24      ------------------------------------------------------------
AaegGr63a     ------------------------------------------------------------
DmelGr63a     ------------------------------------------------------------
TcasGr3       ------------------------------------------------------------
DmelOr83b     ------------------------------------------------------------
AgamOr7       ------------------------------------------------------------
HvirOr2       ------------------------------------------------------------
TcasOr16      ------------------------------------------------------------
TcasOr22      ------------------------------------------------------------
AmelOr2       ------------------------------------------------------------
TcasGr31      ------------------------------------------------------------
TcasGr35      ------------------------------------------------------------
DmelGr59c     ------------------------------------------------------------
DmelGr92a     ------------------------------------------------------------
DmelGr28a     ------------------------------------------------------------
DmelGr64b     ------------------------------------------------------------
TcasGr71      ------------------------------------------------------------
AgamGr19      ------------------------------------------------------------
AaegGr64b     ------------------------------------------------------------
DmelGr93d     ------------------------------------------------------------
TcasGr34      ------------------------------------------------------------
TcasGr43      ------------------------------------------------------------
TcasGr47      ------------------------------------------------------------
AmelGr3       ------------------------------------------------------------
AgamGr32b     ------------------------------------------------------------
AgamGr50      ------------------------------------------------------------
TcasGr20      ------------------------------------------------------------
TcasGr79      ------------------------------------------------------------
TcasGr15      ------------------------------------------------------------
TcasGr60      ------------------------------------------------------------
AgamGr38      ------------------------------------------------------------
TcasGr51      ------------------------------------------------------------
DmelGr39b     ------------------------------------------------------------
AgamGr28      ------------------------------------------------------------
TcasGr61      ------------------------------------------------------------
TcasGr50      ------------------------------------------------------------
```

```
AgamGr12        ------------------------------------------------------------
AgamGr36        ------------------------------------------------------------
AgamGr48        ------------------------------------------------------------
AgamGr49a       ------------------------------------------------------------
AgamGr49b       ------------------------------------------------------------
DmelGr2a        ------------------------------------------------------------
DmelGr32a       ------------------------------------------------------------
DmelGr68a       ------------------------------------------------------------
AmelGr7         ------------------------------------------------------------
AmelGr8         ------------------------------------------------------------
AmelGr9         ------------------------------------------------------------
TcasGr57        ------------------------------------------------------------
TcasGr41        ------------------------------------------------------------
AgamGr34        ------------------------------------------------------------
AgamGr35        ------------------------------------------------------------
AgamGr39        ------------------------------------------------------------
AgamGr40        ------------------------------------------------------------
AaegGr28d       ------------------------------------------------------------
AgamGr42        ------------------------------------------------------------
AgamGr41        ------------------------------------------------------------
AgamGr43        ------------------------------------------------------------
DmelGr33a       ------------------------------------------------------------
AgamGr44        ------------------------------------------------------------
AgamGr13        ------------------------------------------------------------
AgamGr45        ------------------------------------------------------------
AgamGr46        ------------------------------------------------------------
Dmelr93a        ------------------------------------------------------------
AmelGr6         ------------------------------------------------------------
AmelGr10        ------------------------------------------------------------
TcasGr28        ------------------------------------------------------------
TcasGr40        ------------------------------------------------------------
AgamGr3         ------------------------------------------------------------
AgamGr4         ------------------------------------------------------------
AgamGr6         ------------------------------------------------------------
AgamGr7         ------------------------------------------------------------
AgamGr47        ------------------------------------------------------------
DmelGr8a        ------------------------------------------------------------
AgamGr5         ------------------------------------------------------------
AgamGr8         ------------------------------------------------------------
DmelGr9a        ------------------------------------------------------------
DmelGr59f       ------------------------------------------------------------
DmelGr77a       ------------------------------------------------------------
DmelGr85a       ------------------------------------------------------------
TcasGr27        ------------------------------------------------------------
TcasGr48        ------------------------------------------------------------
AgamGr1         ------------------------------------------------------------
DmelGr10a       ------------------------------------------------------------
DmelGr59e       ------------------------------------------------------------
DmelGr10b       ------------------------------------------------------------
DmelGr89a       ------------------------------------------------------------
DmelGr57a       ------------------------------------------------------------
DmelGr47b       ------------------------------------------------------------
DmelGr58a       ------------------------------------------------------------
DmelGr58b       ------------------------------------------------------------
DmelGr98a       ------------------------------------------------------------
AaegGr93a       ------------------------------------------------------------
TcasGr19        ------------------------------------------------------------
DmelGr47a       ------------------------------------------------------------
DmelGr58c       ------------------------------------------------------------


TcasGr38        ------------------------------------------------------------
TcasGr46        SGCIIFTEGIWICSLIHRKKIVKFLDGIMAFDIQLKQVVNYKKKKFQRMVVARYVYYATI 1222
TcasGr1         ------------------------------------------------------------
TcasGr123       ------------------------------------------------------------
TcasGr62        ------------------------------------------------------------
TcasGr25        ------------------------------------------------------------
TcasGr59        ------------------------------------------------------------
AgamGr2         ------------------------------------------------------------
AaegGr66a       ------------------------------------------------------------
```

```
DmelGr66a       ------------------------------------------------------------
TcasGr11        ------------------------------------------------------------
TcasGr55        ------------------------------------------------------------
TcasGr21        ------------------------------------------------------------
TcasGr56        ------------------------------------------------------------
AaegGr          ------------------------------------------------------------
AaegGr28a       ------------------------------------------------------------
AgamGr33        ------------------------------------------------------------
DmelGr28bB      ------------------------------------------------------------
DmelGr28bC      ------------------------------------------------------------
DmelGr28bD      ------------------------------------------------------------
DmelGr28bA      ------------------------------------------------------------
DmelGr28bE      ------------------------------------------------------------
AgamGr25        ------------------------------------------------------------
AaegGr43a       ------------------------------------------------------------
DmelGr43a       ------------------------------------------------------------
AgamGr37b       ------------------------------------------------------------
AgamGr37d       ------------------------------------------------------------
AgamGr37a       ------------------------------------------------------------
AgamGr37c       ------------------------------------------------------------
AgamGr37e       ------------------------------------------------------------
AgamGr37f       ------------------------------------------------------------
AaegGr28c       ------------------------------------------------------------
AaegGr28b       ------------------------------------------------------------
AaegGr28e       ------------------------------------------------------------
TcasGr14        ------------------------------------------------------------
TcasGr49        ------------------------------------------------------------
AmelGr4         ------------------------------------------------------------
AmelGr5         ------------------------------------------------------------
TcasGr16        ------------------------------------------------------------
TcasGr22        ------------------------------------------------------------
TcasGr17        ------------------------------------------------------------
TcasGr150       ------------------------------------------------------------
TcasGr32        ------------------------------------------------------------
TcasGr54        ------------------------------------------------------------
TcasGr37        ------------------------------------------------------------
TcasGr53        ------------------------------------------------------------
TcasGr5         ------------------------------------------------------------
TcasGr12        ------------------------------------------------------------
TcasGr13        ------------------------------------------------------------
TcasGr104       ------------------------------------------------------------
TcasGr45        ------------------------------------------------------------
TcasGr98        ------------------------------------------------------------
TcasGr52        ------------------------------------------------------------
TcasGr105       ------------------------------------------------------------
TcasGr4         ------------------------------------------------------------
TcasGr44        ------------------------------------------------------------
AgamGr9a        ------------------------------------------------------------
AgamGr9c        ------------------------------------------------------------
AgamGr9b        ------------------------------------------------------------
AgamGr9d        ------------------------------------------------------------
AgamGr9e        ------------------------------------------------------------
AgamGr9f        ------------------------------------------------------------
AgamGr9g        ------------------------------------------------------------
AgamGr9i        ------------------------------------------------------------
AgamGr9h        ------------------------------------------------------------
AgamGr9j        ------------------------------------------------------------
AgamGr9l        ------------------------------------------------------------
AgamGr9k        ------------------------------------------------------------
AgamGr9m        ------------------------------------------------------------
AgamGr9n        ------------------------------------------------------------
AgamGr10        ------------------------------------------------------------
AgamGr11        ------------------------------------------------------------
DmelGr23aA      ------------------------------------------------------------
DmelGr23aB      ------------------------------------------------------------
DmelGr39aA      ------------------------------------------------------------
DmelGr39aB      ------------------------------------------------------------
DmelGr39aC      ------------------------------------------------------------
DmelGr39aD      ------------------------------------------------------------
DmelGr98c       ------------------------------------------------------------
```

```
DmelGr98d       ------------------------------------------------------------
DmelGr98b       ------------------------------------------------------------
AgamGr51        ------------------------------------------------------------
AgamGr52        ------------------------------------------------------------
DmelGr94a       ------------------------------------------------------------
DmelGr97a       ------------------------------------------------------------
DmelGr93b       ------------------------------------------------------------
DmelGr93c       ------------------------------------------------------------
DmelGr22d       ------------------------------------------------------------
DmelGr22e       ------------------------------------------------------------
DmelGr22a       ------------------------------------------------------------
DmelGr22b       ------------------------------------------------------------
DmelGr22c       ------------------------------------------------------------
DmelGr22f       ------------------------------------------------------------
DmelGr36a       ------------------------------------------------------------
DmelGr36c       ------------------------------------------------------------
DmelGr36b       ------------------------------------------------------------
DmelGr59d       ------------------------------------------------------------
DmelGr59a       ------------------------------------------------------------
DmelGr59b       ------------------------------------------------------------
AgamGr26        ------------------------------------------------------------
AgamGr27        ------------------------------------------------------------
AgamGr29        ------------------------------------------------------------
AgamGr31        ------------------------------------------------------------
AgamGr30        ------------------------------------------------------------
AgamGr32a       ------------------------------------------------------------
TcasGr2         ------------------------------------------------------------
TcasGr9         ------------------------------------------------------------
TcasGr7         ------------------------------------------------------------
TcasGr30        ------------------------------------------------------------
TcasGr29        ------------------------------------------------------------
AaegGr64f       ------------------------------------------------------------
AaegGr64a3      ------------------------------------------------------------
AgamGr14        ------------------------------------------------------------
AgamGr18        ------------------------------------------------------------
AaegGr64d       ------------------------------------------------------------
AgamGr17        ------------------------------------------------------------
AaegGr64c       ------------------------------------------------------------
DmelGr64c       ------------------------------------------------------------
DmelGr64d       ------------------------------------------------------------
AgamGr20        ------------------------------------------------------------
AaegGr61a       ------------------------------------------------------------
AgamGr21        ------------------------------------------------------------
AaegGr64a       ------------------------------------------------------------
DmelGr64a       ------------------------------------------------------------
DmelGr61a       ------------------------------------------------------------
AgamGr15        ------------------------------------------------------------
AaegGr1         ------------------------------------------------------------
DmelGr5a        ------------------------------------------------------------
DmelGr64f       ------------------------------------------------------------
DmelGr64e       ------------------------------------------------------------
AmelGr1         ------------------------------------------------------------
AmelGr2         ------------------------------------------------------------
AgamGr16        ------------------------------------------------------------
AaegGr64e       ------------------------------------------------------------
TCasGr6         ------------------------------------------------------------
TcasGr26        ------------------------------------------------------------
TcasGr33        ------------------------------------------------------------
AgamGr23        ------------------------------------------------------------
AaegGr21a       ------------------------------------------------------------
TcasGr10        ------------------------------------------------------------
TcasGr39        ------------------------------------------------------------
AgamGr22        ------------------------------------------------------------
AaegGr21b       ------------------------------------------------------------
DmelGr21a       ------------------------------------------------------------
AgamGr24        ------------------------------------------------------------
AaegGr63a       ------------------------------------------------------------
DmelGr63a       ------------------------------------------------------------
TcasGr3         ------------------------------------------------------------
DmelOr83b       ------------------------------------------------------------
```

```
AgamOr7         ------------------------------------------------------------
HvirOr2         ------------------------------------------------------------
TcasOr16        ------------------------------------------------------------
TcasOr22        ------------------------------------------------------------
AmelOr2         ------------------------------------------------------------
TcasGr31        ------------------------------------------------------------
TcasGr35        ------------------------------------------------------------
DmelGr59c       ------------------------------------------------------------
DmelGr92a       ------------------------------------------------------------
DmelGr28a       ------------------------------------------------------------
DmelGr64b       ------------------------------------------------------------
TcasGr71        ------------------------------------------------------------
AgamGr19        ------------------------------------------------------------
AaegGr64b       ------------------------------------------------------------
DmelGr93d       ------------------------------------------------------------
TcasGr34        ------------------------------------------------------------
TcasGr43        ------------------------------------------------------------
TcasGr47        ------------------------------------------------------------
AmelGr3         ------------------------------------------------------------
AgamGr32b       ------------------------------------------------------------
AgamGr50        ------------------------------------------------------------
TcasGr20        ------------------------------------------------------------
TcasGr79        ------------------------------------------------------------
TcasGr15        ------------------------------------------------------------
TcasGr60        ------------------------------------------------------------
AgamGr38        ------------------------------------------------------------
TcasGr51        ------------------------------------------------------------
DmelGr39b       ------------------------------------------------------------
AgamGr28        ------------------------------------------------------------
TcasGr61        ------------------------------------------------------------
TcasGr50        ------------------------------------------------------------
AgamGr12        ------------------------------------------------------------
AgamGr36        ------------------------------------------------------------
AgamGr48        ------------------------------------------------------------
AgamGr49a       ------------------------------------------------------------
AgamGr49b       ------------------------------------------------------------
DmelGr2a        ------------------------------------------------------------
DmelGr32a       ------------------------------------------------------------
DmelGr68a       ------------------------------------------------------------
AmelGr7         ------------------------------------------------------------
AmelGr8         ------------------------------------------------------------
AmelGr9         ------------------------------------------------------------
TcasGr57        ------------------------------------------------------------
TcasGr41        ------------------------------------------------------------
AgamGr34        ------------------------------------------------------------
AgamGr35        ------------------------------------------------------------
AgamGr39        ------------------------------------------------------------
AgamGr40        ------------------------------------------------------------
AaegGr28d       ------------------------------------------------------------
AgamGr42        ------------------------------------------------------------
AgamGr41        ------------------------------------------------------------
AgamGr43        ------------------------------------------------------------
DmelGr33a       ------------------------------------------------------------
AgamGr44        ------------------------------------------------------------
AgamGr13        ------------------------------------------------------------
AgamGr45        ------------------------------------------------------------
AgamGr46        ------------------------------------------------------------
Dmelr93a        ------------------------------------------------------------
AmelGr6         ------------------------------------------------------------
AmelGr10        ------------------------------------------------------------
TcasGr28        ------------------------------------------------------------
TcasGr40        ------------------------------------------------------------
AgamGr3         ------------------------------------------------------------
AgamGr4         ------------------------------------------------------------
AgamGr6         ------------------------------------------------------------
AgamGr7         ------------------------------------------------------------
AgamGr47        ------------------------------------------------------------
DmelGr8a        ------------------------------------------------------------
AgamGr5         ------------------------------------------------------------
AgamGr8         ------------------------------------------------------------
```

```
DmelGr9a        ------------------------------------------------------------
DmelGr59f       ------------------------------------------------------------
DmelGr77a       ------------------------------------------------------------
DmelGr85a       ------------------------------------------------------------
TcasGr27        ------------------------------------------------------------
TcasGr48        ------------------------------------------------------------
AgamGr1         ------------------------------------------------------------
DmelGr10a       ------------------------------------------------------------
DmelGr59e       ------------------------------------------------------------
DmelGr10b       ------------------------------------------------------------
DmelGr89a       ------------------------------------------------------------
DmelGr57a       ------------------------------------------------------------
DmelGr47b       ------------------------------------------------------------
DmelGr58a       ------------------------------------------------------------
DmelGr58b       ------------------------------------------------------------
DmelGr98a       ------------------------------------------------------------
AaegGr93a       ------------------------------------------------------------
TcasGr19        ------------------------------------------------------------
DmelGr47a       ------------------------------------------------------------
DmelGr58c       ------------------------------------------------------------


TcasGr38        ------------------------------------------------------------
TcasGr46        FTIITATSSFVTIHNVIGQFLGYFIGLITFVMSQHCVELVSMIKARFVVVNQQIGGIVTY 1282
TcasGr1         ------------------------------------------------------------
TcasGr123       ------------------------------------------------------------
TcasGr62        ------------------------------------------------------------
TcasGr25        ------------------------------------------------------------
TcasGr59        ------------------------------------------------------------
AgamGr2         ------------------------------------------------------------
AaegGr66a       ------------------------------------------------------------
DmelGr66a       ------------------------------------------------------------
TcasGr11        ------------------------------------------------------------
TcasGr55        ------------------------------------------------------------
TcasGr21        ------------------------------------------------------------
TcasGr56        ------------------------------------------------------------
AaegGr          ------------------------------------------------------------
AaegGr28a       ------------------------------------------------------------
AgamGr33        ------------------------------------------------------------
DmelGr28bB      ------------------------------------------------------------
DmelGr28bC      ------------------------------------------------------------
DmelGr28bD      ------------------------------------------------------------
DmelGr28bA      ------------------------------------------------------------
DmelGr28bE      ------------------------------------------------------------
AgamGr25        ------------------------------------------------------------
AaegGr43a       ------------------------------------------------------------
DmelGr43a       ------------------------------------------------------------
AgamGr37b       ------------------------------------------------------------
AgamGr37d       ------------------------------------------------------------
AgamGr37a       ------------------------------------------------------------
AgamGr37c       ------------------------------------------------------------
AgamGr37e       ------------------------------------------------------------
AgamGr37f       ------------------------------------------------------------
AaegGr28c       ------------------------------------------------------------
AaegGr28b       ------------------------------------------------------------
AaegGr28e       ------------------------------------------------------------
TcasGr14        ------------------------------------------------------------
TcasGr49        ------------------------------------------------------------
AmelGr4         ------------------------------------------------------------
AmelGr5         ------------------------------------------------------------
TcasGr16        ------------------------------------------------------------
TcasGr22        ------------------------------------------------------------
TcasGr17        ------------------------------------------------------------
TcasGr150       ------------------------------------------------------------
TcasGr32        ------------------------------------------------------------
TcasGr54        ------------------------------------------------------------
TcasGr37        ------------------------------------------------------------
TcasGr53        ------------------------------------------------------------
TcasGr5         ------------------------------------------------------------
TcasGr12        ------------------------------------------------------------
```

```
TcasGr13        ------------------------------------------------------------
TcasGr104       ------------------------------------------------------------
TcasGr45        ------------------------------------------------------------
TcasGr98        ------------------------------------------------------------
TcasGr52        ------------------------------------------------------------
TcasGr105       ------------------------------------------------------------
TcasGr4         ------------------------------------------------------------
TcasGr44        ------------------------------------------------------------
AgamGr9a        ------------------------------------------------------------
AgamGr9c        ------------------------------------------------------------
AgamGr9b        ------------------------------------------------------------
AgamGr9d        ------------------------------------------------------------
AgamGr9e        ------------------------------------------------------------
AgamGr9f        ------------------------------------------------------------
AgamGr9g        ------------------------------------------------------------
AgamGr9i        ------------------------------------------------------------
AgamGr9h        ------------------------------------------------------------
AgamGr9j        ------------------------------------------------------------
AgamGr9l        ------------------------------------------------------------
AgamGr9k        ------------------------------------------------------------
AgamGr9m        ------------------------------------------------------------
AgamGr9n        ------------------------------------------------------------
AgamGr10        ------------------------------------------------------------
AgamGr11        ------------------------------------------------------------
DmelGr23aA      ------------------------------------------------------------
DmelGr23aB      ------------------------------------------------------------
DmelGr39aA      ------------------------------------------------------------
DmelGr39aB      ------------------------------------------------------------
DmelGr39aC      ------------------------------------------------------------
DmelGr39aD      ------------------------------------------------------------
DmelGr98c       ------------------------------------------------------------
DmelGr98d       ------------------------------------------------------------
DmelGr98b       ------------------------------------------------------------
AgamGr51        ------------------------------------------------------------
AgamGr52        ------------------------------------------------------------
DmelGr94a       ------------------------------------------------------------
DmelGr97a       ------------------------------------------------------------
DmelGr93b       ------------------------------------------------------------
DmelGr93c       ------------------------------------------------------------
DmelGr22d       ------------------------------------------------------------
DmelGr22e       ------------------------------------------------------------
DmelGr22a       ------------------------------------------------------------
DmelGr22b       ------------------------------------------------------------
DmelGr22c       ------------------------------------------------------------
DmelGr22f       ------------------------------------------------------------
DmelGr36a       ------------------------------------------------------------
DmelGr36c       ------------------------------------------------------------
DmelGr36b       ------------------------------------------------------------
DmelGr59d       ------------------------------------------------------------
DmelGr59a       ------------------------------------------------------------
DmelGr59b       ------------------------------------------------------------
AgamGr26        ------------------------------------------------------------
AgamGr27        ------------------------------------------------------------
AgamGr29        ------------------------------------------------------------
AgamGr31        ------------------------------------------------------------
AgamGr30        ------------------------------------------------------------
AgamGr32a       ------------------------------------------------------------
TcasGr2         ------------------------------------------------------------
TcasGr9         ------------------------------------------------------------
TcasGr7         ------------------------------------------------------------
TcasGr30        ------------------------------------------------------------
TcasGr29        ------------------------------------------------------------
AaegGr64f       ------------------------------------------------------------
AaegGr64a3      ------------------------------------------------------------
AgamGr14        ------------------------------------------------------------
AgamGr18        ------------------------------------------------------------
AaegGr64d       ------------------------------------------------------------
AgamGr17        ------------------------------------------------------------
AaegGr64c       ------------------------------------------------------------
DmelGr64c       ------------------------------------------------------------
```

```
DmelGr64d       ------------------------------------------------------------
AgamGr20        ------------------------------------------------------------
AaegGr61a       ------------------------------------------------------------
AgamGr21        ------------------------------------------------------------
AaegGr64a       ------------------------------------------------------------
DmelGr64a       ------------------------------------------------------------
DmelGr61a       ------------------------------------------------------------
AgamGr15        ------------------------------------------------------------
AaegGr1         ------------------------------------------------------------
DmelGr5a        ------------------------------------------------------------
DmelGr64f       ------------------------------------------------------------
DmelGr64e       ------------------------------------------------------------
AmelGr1         ------------------------------------------------------------
AmelGr2         ------------------------------------------------------------
AgamGr16        ------------------------------------------------------------
AaegGr64e       ------------------------------------------------------------
TCasGr6         ------------------------------------------------------------
TcasGr26        ------------------------------------------------------------
TcasGr33        ------------------------------------------------------------
AgamGr23        ------------------------------------------------------------
AaegGr21a       ------------------------------------------------------------
TcasGr10        ------------------------------------------------------------
TcasGr39        ------------------------------------------------------------
AgamGr22        ------------------------------------------------------------
AaegGr21b       ------------------------------------------------------------
DmelGr21a       ------------------------------------------------------------
AgamGr24        ------------------------------------------------------------
AaegGr63a       ------------------------------------------------------------
DmelGr63a       ------------------------------------------------------------
TcasGr3         ------------------------------------------------------------
DmelOr83b       ------------------------------------------------------------
AgamOr7         ------------------------------------------------------------
HvirOr2         ------------------------------------------------------------
TcasOr16        ------------------------------------------------------------
TcasOr22        ------------------------------------------------------------
AmelOr2         ------------------------------------------------------------
TcasGr31        ------------------------------------------------------------
TcasGr35        ------------------------------------------------------------
DmelGr59c       ------------------------------------------------------------
DmelGr92a       ------------------------------------------------------------
DmelGr28a       ------------------------------------------------------------
DmelGr64b       ------------------------------------------------------------
TcasGr71        ------------------------------------------------------------
AgamGr19        ------------------------------------------------------------
AaegGr64b       ------------------------------------------------------------
DmelGr93d       ------------------------------------------------------------
TcasGr34        ------------------------------------------------------------
TcasGr43        ------------------------------------------------------------
TcasGr47        ------------------------------------------------------------
AmelGr3         ------------------------------------------------------------
AgamGr32b       ------------------------------------------------------------
AgamGr50        ------------------------------------------------------------
TcasGr20        ------------------------------------------------------------
TcasGr79        ------------------------------------------------------------
TcasGr15        ------------------------------------------------------------
TcasGr60        ------------------------------------------------------------
AgamGr38        ------------------------------------------------------------
TcasGr51        ------------------------------------------------------------
DmelGr39b       ------------------------------------------------------------
AgamGr28        ------------------------------------------------------------
TcasGr61        ------------------------------------------------------------
TcasGr50        ------------------------------------------------------------
AgamGr12        ------------------------------------------------------------
AgamGr36        ------------------------------------------------------------
AgamGr48        ------------------------------------------------------------
AgamGr49a       ------------------------------------------------------------
AgamGr49b       ------------------------------------------------------------
DmelGr2a        ------------------------------------------------------------
DmelGr32a       ------------------------------------------------------------
DmelGr68a       ------------------------------------------------------------
```

```
AmelGr7         ------------------------------------------------------------
AmelGr8         ------------------------------------------------------------
AmelGr9         ------------------------------------------------------------
TcasGr57        ------------------------------------------------------------
TcasGr41        ------------------------------------------------------------
AgamGr34        ------------------------------------------------------------
AgamGr35        ------------------------------------------------------------
AgamGr39        ------------------------------------------------------------
AgamGr40        ------------------------------------------------------------
AaegGr28d       ------------------------------------------------------------
AgamGr42        ------------------------------------------------------------
AgamGr41        ------------------------------------------------------------
AgamGr43        ------------------------------------------------------------
DmelGr33a       ------------------------------------------------------------
AgamGr44        ------------------------------------------------------------
AgamGr13        ------------------------------------------------------------
AgamGr45        ------------------------------------------------------------
AgamGr46        ------------------------------------------------------------
Dmelr93a        ------------------------------------------------------------
AmelGr6         ------------------------------------------------------------
AmelGr10        ------------------------------------------------------------
TcasGr28        ------------------------------------------------------------
TcasGr40        ------------------------------------------------------------
AgamGr3         ------------------------------------------------------------
AgamGr4         ------------------------------------------------------------
AgamGr6         ------------------------------------------------------------
AgamGr7         ------------------------------------------------------------
AgamGr47        ------------------------------------------------------------
DmelGr8a        ------------------------------------------------------------
AgamGr5         ------------------------------------------------------------
AgamGr8         ------------------------------------------------------------
DmelGr9a        ------------------------------------------------------------
DmelGr59f       ------------------------------------------------------------
DmelGr77a       ------------------------------------------------------------
DmelGr85a       ------------------------------------------------------------
TcasGr27        ------------------------------------------------------------
TcasGr48        ------------------------------------------------------------
AgamGr1         ------------------------------------------------------------
DmelGr10a       ------------------------------------------------------------
DmelGr59e       ------------------------------------------------------------
DmelGr10b       ------------------------------------------------------------
DmelGr89a       ------------------------------------------------------------
DmelGr57a       ------------------------------------------------------------
DmelGr47b       ------------------------------------------------------------
DmelGr58a       ------------------------------------------------------------
DmelGr58b       ------------------------------------------------------------
DmelGr98a       ------------------------------------------------------------
AaegGr93a       ------------------------------------------------------------
TcasGr19        ------------------------------------------------------------
DmelGr47a       ------------------------------------------------------------
DmelGr58c       ------------------------------------------------------------


TcasGr38        ------------------------------------------------------------
TcasGr46        FSTNLPKRETEVRKKSLDFGKLCSLHHHLSKLIKSFNEIYGVPLLLFFGLNFLIITQAMF 1342
TcasGr1         ------------------------------------------------------------
TcasGr123       ------------------------------------------------------------
TcasGr62        ------------------------------------------------------------
TcasGr25        ------------------------------------------------------------
TcasGr59        ------------------------------------------------------------
AgamGr2         ------------------------------------------------------------
AaegGr66a       ------------------------------------------------------------
DmelGr66a       ------------------------------------------------------------
TcasGr11        ------------------------------------------------------------
TcasGr55        ------------------------------------------------------------
TcasGr21        ------------------------------------------------------------
TcasGr56        ------------------------------------------------------------
AaegGr          ------------------------------------------------------------
AaegGr28a       ------------------------------------------------------------
AgamGr33        ------------------------------------------------------------
```

```
DmelGr28bB      ------------------------------------------------------------
DmelGr28bC      ------------------------------------------------------------
DmelGr28bD      ------------------------------------------------------------
DmelGr28bA      ------------------------------------------------------------
DmelGr28bE      ------------------------------------------------------------
AgamGr25        ------------------------------------------------------------
AaegGr43a       ------------------------------------------------------------
DmelGr43a       ------------------------------------------------------------
AgamGr37b       ------------------------------------------------------------
AgamGr37d       ------------------------------------------------------------
AgamGr37a       ------------------------------------------------------------
AgamGr37c       ------------------------------------------------------------
AgamGr37e       ------------------------------------------------------------
AgamGr37f       ------------------------------------------------------------
AaegGr28c       ------------------------------------------------------------
AaegGr28b       ------------------------------------------------------------
AaegGr28e       ------------------------------------------------------------
TcasGr14        ------------------------------------------------------------
TcasGr49        ------------------------------------------------------------
AmelGr4         ------------------------------------------------------------
AmelGr5         ------------------------------------------------------------
TcasGr16        ------------------------------------------------------------
TcasGr22        ------------------------------------------------------------
TcasGr17        ------------------------------------------------------------
TcasGr150       ------------------------------------------------------------
TcasGr32        ------------------------------------------------------------
TcasGr54        ------------------------------------------------------------
TcasGr37        ------------------------------------------------------------
TcasGr53        ------------------------------------------------------------
TcasGr5         ------------------------------------------------------------
TcasGr12        ------------------------------------------------------------
TcasGr13        ------------------------------------------------------------
TcasGr104       ------------------------------------------------------------
TcasGr45        ------------------------------------------------------------
TcasGr98        ------------------------------------------------------------
TcasGr52        ------------------------------------------------------------
TcasGr105       ------------------------------------------------------------
TcasGr4         ------------------------------------------------------------
TcasGr44        ------------------------------------------------------------
AgamGr9a        ------------------------------------------------------------
AgamGr9c        ------------------------------------------------------------
AgamGr9b        ------------------------------------------------------------
AgamGr9d        ------------------------------------------------------------
AgamGr9e        ------------------------------------------------------------
AgamGr9f        ------------------------------------------------------------
AgamGr9g        ------------------------------------------------------------
AgamGr9i        ------------------------------------------------------------
AgamGr9h        ------------------------------------------------------------
AgamGr9j        ------------------------------------------------------------
AgamGr9l        ------------------------------------------------------------
AgamGr9k        ------------------------------------------------------------
AgamGr9m        ------------------------------------------------------------
AgamGr9n        ------------------------------------------------------------
AgamGr10        ------------------------------------------------------------
AgamGr11        ------------------------------------------------------------
DmelGr23aA      ------------------------------------------------------------
DmelGr23aB      ------------------------------------------------------------
DmelGr39aA      ------------------------------------------------------------
DmelGr39aB      ------------------------------------------------------------
DmelGr39aC      ------------------------------------------------------------
DmelGr39aD      ------------------------------------------------------------
DmelGr98c       ------------------------------------------------------------
DmelGr98d       ------------------------------------------------------------
DmelGr98b       ------------------------------------------------------------
AgamGr51        ------------------------------------------------------------
AgamGr52        ------------------------------------------------------------
DmelGr94a       ------------------------------------------------------------
DmelGr97a       ------------------------------------------------------------
DmelGr93b       ------------------------------------------------------------
DmelGr93c       ------------------------------------------------------------
```

```
DmelGr22d       ------------------------------------------------------------
DmelGr22e       ------------------------------------------------------------
DmelGr22a       ------------------------------------------------------------
DmelGr22b       ------------------------------------------------------------
DmelGr22c       ------------------------------------------------------------
DmelGr22f       ------------------------------------------------------------
DmelGr36a       ------------------------------------------------------------
DmelGr36c       ------------------------------------------------------------
DmelGr36b       ------------------------------------------------------------
DmelGr59d       ------------------------------------------------------------
DmelGr59a       ------------------------------------------------------------
DmelGr59b       ------------------------------------------------------------
AgamGr26        ------------------------------------------------------------
AgamGr27        ------------------------------------------------------------
AgamGr29        ------------------------------------------------------------
AgamGr31        ------------------------------------------------------------
AgamGr30        ------------------------------------------------------------
AgamGr32a       ------------------------------------------------------------
TcasGr2         ------------------------------------------------------------
TcasGr9         ------------------------------------------------------------
TcasGr7         ------------------------------------------------------------
TcasGr30        ------------------------------------------------------------
TcasGr29        ------------------------------------------------------------
AaegGr64f       ------------------------------------------------------------
AaegGr64a3      ------------------------------------------------------------
AgamGr14        ------------------------------------------------------------
AgamGr18        ------------------------------------------------------------
AaegGr64d       ------------------------------------------------------------
AgamGr17        ------------------------------------------------------------
AaegGr64c       ------------------------------------------------------------
DmelGr64c       ------------------------------------------------------------
DmelGr64d       ------------------------------------------------------------
AgamGr20        ------------------------------------------------------------
AaegGr61a       ------------------------------------------------------------
AgamGr21        ------------------------------------------------------------
AaegGr64a       ------------------------------------------------------------
DmelGr64a       ------------------------------------------------------------
DmelGr61a       ------------------------------------------------------------
AgamGr15        ------------------------------------------------------------
AaegGr1         ------------------------------------------------------------
DmelGr5a        ------------------------------------------------------------
DmelGr64f       ------------------------------------------------------------
DmelGr64e       ------------------------------------------------------------
AmelGr1         ------------------------------------------------------------
AmelGr2         ------------------------------------------------------------
AgamGr16        ------------------------------------------------------------
AaegGr64e       ------------------------------------------------------------
TCasGr6         ------------------------------------------------------------
TcasGr26        ------------------------------------------------------------
TcasGr33        ------------------------------------------------------------
AgamGr23        ------------------------------------------------------------
AaegGr21a       ------------------------------------------------------------
TcasGr10        ------------------------------------------------------------
TcasGr39        ------------------------------------------------------------
AgamGr22        ------------------------------------------------------------
AaegGr21b       ------------------------------------------------------------
DmelGr21a       ------------------------------------------------------------
AgamGr24        ------------------------------------------------------------
AaegGr63a       ------------------------------------------------------------
DmelGr63a       ------------------------------------------------------------
TcasGr3         ------------------------------------------------------------
DmelOr83b       ------------------------------------------------------------
AgamOr7         ------------------------------------------------------------
HvirOr2         ------------------------------------------------------------
TcasOr16        ------------------------------------------------------------
TcasOr22        ------------------------------------------------------------
AmelOr2         ------------------------------------------------------------
TcasGr31        ------------------------------------------------------------
TcasGr35        ------------------------------------------------------------
DmelGr59c       ------------------------------------------------------------
```

```
DmelGr92a       ------------------------------------------------------------
DmelGr28a       ------------------------------------------------------------
DmelGr64b       ------------------------------------------------------------
TcasGr71        ------------------------------------------------------------
AgamGr19        ------------------------------------------------------------
AaegGr64b       ------------------------------------------------------------
DmelGr93d       ------------------------------------------------------------
TcasGr34        ------------------------------------------------------------
TcasGr43        ------------------------------------------------------------
TcasGr47        ------------------------------------------------------------
AmelGr3         ------------------------------------------------------------
AgamGr32b       ------------------------------------------------------------
AgamGr50        ------------------------------------------------------------
TcasGr20        ------------------------------------------------------------
TcasGr79        ------------------------------------------------------------
TcasGr15        ------------------------------------------------------------
TcasGr60        ------------------------------------------------------------
AgamGr38        ------------------------------------------------------------
TcasGr51        ------------------------------------------------------------
DmelGr39b       ------------------------------------------------------------
AgamGr28        ------------------------------------------------------------
TcasGr61        ------------------------------------------------------------
TcasGr50        ------------------------------------------------------------
AgamGr12        ------------------------------------------------------------
AgamGr36        ------------------------------------------------------------
AgamGr48        ------------------------------------------------------------
AgamGr49a       ------------------------------------------------------------
AgamGr49b       ------------------------------------------------------------
DmelGr2a        ------------------------------------------------------------
DmelGr32a       ------------------------------------------------------------
DmelGr68a       ------------------------------------------------------------
AmelGr7         ------------------------------------------------------------
AmelGr8         ------------------------------------------------------------
AmelGr9         ------------------------------------------------------------
TcasGr57        ------------------------------------------------------------
TcasGr41        ------------------------------------------------------------
AgamGr34        ------------------------------------------------------------
AgamGr35        ------------------------------------------------------------
AgamGr39        ------------------------------------------------------------
AgamGr40        ------------------------------------------------------------
AaegGr28d       ------------------------------------------------------------
AgamGr42        ------------------------------------------------------------
AgamGr41        ------------------------------------------------------------
AgamGr43        ------------------------------------------------------------
DmelGr33a       ------------------------------------------------------------
AgamGr44        ------------------------------------------------------------
AgamGr13        ------------------------------------------------------------
AgamGr45        ------------------------------------------------------------
AgamGr46        ------------------------------------------------------------
Dmelr93a        ------------------------------------------------------------
AmelGr6         ------------------------------------------------------------
AmelGr10        ------------------------------------------------------------
TcasGr28        ------------------------------------------------------------
TcasGr40        ------------------------------------------------------------
AgamGr3         ------------------------------------------------------------
AgamGr4         ------------------------------------------------------------
AgamGr6         ------------------------------------------------------------
AgamGr7         ------------------------------------------------------------
AgamGr47        ------------------------------------------------------------
DmelGr8a        ------------------------------------------------------------
AgamGr5         ------------------------------------------------------------
AgamGr8         ------------------------------------------------------------
DmelGr9a        ------------------------------------------------------------
DmelGr59f       ------------------------------------------------------------
DmelGr77a       ------------------------------------------------------------
DmelGr85a       ------------------------------------------------------------
TcasGr27        ------------------------------------------------------------
TcasGr48        ------------------------------------------------------------
AgamGr1         ------------------------------------------------------------
DmelGr10a       ------------------------------------------------------------
```

```
DmelGr59e       ------------------------------------------------------------
DmelGr10b       ------------------------------------------------------------
DmelGr89a       ------------------------------------------------------------
DmelGr57a       ------------------------------------------------------------
DmelGr47b       ------------------------------------------------------------
DmelGr58a       ------------------------------------------------------------
DmelGr58b       ------------------------------------------------------------
DmelGr98a       ------------------------------------------------------------
AaegGr93a       ------------------------------------------------------------
TcasGr19        ------------------------------------------------------------
DmelGr47a       ------------------------------------------------------------
DmelGr58c       ------------------------------------------------------------


TcasGr38        ------------------------------------------------------------
TcasGr46        LVVGQLQASQIHWQKIIIIVMSSVTYGIDTVAVCDACYSTIEEKNLQVNESGKLIHKIDA 1402
TcasGr1         ------------------------------------------------------------
TcasGr123       ------------------------------------------------------------
TcasGr62        ------------------------------------------------------------
TcasGr25        ------------------------------------------------------------
TcasGr59        ------------------------------------------------------------
AgamGr2         ------------------------------------------------------------
AaegGr66a       ------------------------------------------------------------
DmelGr66a       ------------------------------------------------------------
TcasGr11        ------------------------------------------------------------
TcasGr55        ------------------------------------------------------------
TcasGr21        ------------------------------------------------------------
TcasGr56        ------------------------------------------------------------
AaegGr          ------------------------------------------------------------
AaegGr28a       ------------------------------------------------------------
AgamGr33        ------------------------------------------------------------
DmelGr28bB      ------------------------------------------------------------
DmelGr28bC      ------------------------------------------------------------
DmelGr28bD      ------------------------------------------------------------
DmelGr28bA      ------------------------------------------------------------
DmelGr28bE      ------------------------------------------------------------
AgamGr25        ------------------------------------------------------------
AaegGr43a       ------------------------------------------------------------
DmelGr43a       ------------------------------------------------------------
AgamGr37b       ------------------------------------------------------------
AgamGr37d       ------------------------------------------------------------
AgamGr37a       ------------------------------------------------------------
AgamGr37c       ------------------------------------------------------------
AgamGr37e       ------------------------------------------------------------
AgamGr37f       ------------------------------------------------------------
AaegGr28c       ------------------------------------------------------------
AaegGr28b       ------------------------------------------------------------
AaegGr28e       ------------------------------------------------------------
TcasGr14        ------------------------------------------------------------
TcasGr49        ------------------------------------------------------------
AmelGr4         ------------------------------------------------------------
AmelGr5         ------------------------------------------------------------
TcasGr16        ------------------------------------------------------------
TcasGr22        ------------------------------------------------------------
TcasGr17        ------------------------------------------------------------
TcasGr150       ------------------------------------------------------------
TcasGr32        ------------------------------------------------------------
TcasGr54        ------------------------------------------------------------
TcasGr37        ------------------------------------------------------------
TcasGr53        ------------------------------------------------------------
TcasGr5         ------------------------------------------------------------
TcasGr12        ------------------------------------------------------------
TcasGr13        ------------------------------------------------------------
TcasGr104       ------------------------------------------------------------
TcasGr45        ------------------------------------------------------------
TcasGr98        ------------------------------------------------------------
TcasGr52        ------------------------------------------------------------
TcasGr105       ------------------------------------------------------------
TcasGr4         ------------------------------------------------------------
TcasGr44        ------------------------------------------------------------
```

```
AgamGr9a        ------------------------------------------------------------
AgamGr9c        ------------------------------------------------------------
AgamGr9b        ------------------------------------------------------------
AgamGr9d        ------------------------------------------------------------
AgamGr9e        ------------------------------------------------------------
AgamGr9f        ------------------------------------------------------------
AgamGr9g        ------------------------------------------------------------
AgamGr9i        ------------------------------------------------------------
AgamGr9h        ------------------------------------------------------------
AgamGr9j        ------------------------------------------------------------
AgamGr9l        ------------------------------------------------------------
AgamGr9k        ------------------------------------------------------------
AgamGr9m        ------------------------------------------------------------
AgamGr9n        ------------------------------------------------------------
AgamGr10        ------------------------------------------------------------
AgamGr11        ------------------------------------------------------------
DmelGr23aA      ------------------------------------------------------------
DmelGr23aB      ------------------------------------------------------------
DmelGr39aA      ------------------------------------------------------------
DmelGr39aB      ------------------------------------------------------------
DmelGr39aC      ------------------------------------------------------------
DmelGr39aD      ------------------------------------------------------------
DmelGr98c       ------------------------------------------------------------
DmelGr98d       ------------------------------------------------------------
DmelGr98b       ------------------------------------------------------------
AgamGr51        ------------------------------------------------------------
AgamGr52        ------------------------------------------------------------
DmelGr94a       ------------------------------------------------------------
DmelGr97a       ------------------------------------------------------------
DmelGr93b       ------------------------------------------------------------
DmelGr93c       ------------------------------------------------------------
DmelGr22d       ------------------------------------------------------------
DmelGr22e       ------------------------------------------------------------
DmelGr22a       ------------------------------------------------------------
DmelGr22b       ------------------------------------------------------------
DmelGr22c       ------------------------------------------------------------
DmelGr22f       ------------------------------------------------------------
DmelGr36a       ------------------------------------------------------------
DmelGr36c       ------------------------------------------------------------
DmelGr36b       ------------------------------------------------------------
DmelGr59d       ------------------------------------------------------------
DmelGr59a       ------------------------------------------------------------
DmelGr59b       ------------------------------------------------------------
AgamGr26        ------------------------------------------------------------
AgamGr27        ------------------------------------------------------------
AgamGr29        ------------------------------------------------------------
AgamGr31        ------------------------------------------------------------
AgamGr30        ------------------------------------------------------------
AgamGr32a       ------------------------------------------------------------
TcasGr2         ------------------------------------------------------------
TcasGr9         ------------------------------------------------------------
TcasGr7         ------------------------------------------------------------
TcasGr30        ------------------------------------------------------------
TcasGr29        ------------------------------------------------------------
AaegGr64f       ------------------------------------------------------------
AaegGr64a3      ------------------------------------------------------------
AgamGr14        ------------------------------------------------------------
AgamGr18        ------------------------------------------------------------
AaegGr64d       ------------------------------------------------------------
AgamGr17        ------------------------------------------------------------
AaegGr64c       ------------------------------------------------------------
DmelGr64c       ------------------------------------------------------------
DmelGr64d       ------------------------------------------------------------
AgamGr20        ------------------------------------------------------------
AaegGr61a       ------------------------------------------------------------
AgamGr21        ------------------------------------------------------------
AaegGr64a       ------------------------------------------------------------
DmelGr64a       ------------------------------------------------------------
DmelGr61a       ------------------------------------------------------------
AgamGr15        ------------------------------------------------------------
```

```
AaegGr1         ------------------------------------------------------------
DmelGr5a        ------------------------------------------------------------
DmelGr64f       ------------------------------------------------------------
DmelGr64e       ------------------------------------------------------------
AmelGr1         ------------------------------------------------------------
AmelGr2         ------------------------------------------------------------
AgamGr16        ------------------------------------------------------------
AaegGr64e       ------------------------------------------------------------
TCasGr6         ------------------------------------------------------------
TcasGr26        ------------------------------------------------------------
TcasGr33        ------------------------------------------------------------
AgamGr23        ------------------------------------------------------------
AaegGr21a       ------------------------------------------------------------
TcasGr10        ------------------------------------------------------------
TcasGr39        ------------------------------------------------------------
AgamGr22        ------------------------------------------------------------
AaegGr21b       ------------------------------------------------------------
DmelGr21a       ------------------------------------------------------------
AgamGr24        ------------------------------------------------------------
AaegGr63a       ------------------------------------------------------------
DmelGr63a       ------------------------------------------------------------
TcasGr3         ------------------------------------------------------------
DmelOr83b       ------------------------------------------------------------
AgamOr7         ------------------------------------------------------------
HvirOr2         ------------------------------------------------------------
TcasOr16        ------------------------------------------------------------
TcasOr22        ------------------------------------------------------------
AmelOr2         ------------------------------------------------------------
TcasGr31        ------------------------------------------------------------
TcasGr35        ------------------------------------------------------------
DmelGr59c       ------------------------------------------------------------
DmelGr92a       ------------------------------------------------------------
DmelGr28a       ------------------------------------------------------------
DmelGr64b       ------------------------------------------------------------
TcasGr71        ------------------------------------------------------------
AgamGr19        ------------------------------------------------------------
AaegGr64b       ------------------------------------------------------------
DmelGr93d       ------------------------------------------------------------
TcasGr34        ------------------------------------------------------------
TcasGr43        ------------------------------------------------------------
TcasGr47        ------------------------------------------------------------
AmelGr3         ------------------------------------------------------------
AgamGr32b       ------------------------------------------------------------
AgamGr50        ------------------------------------------------------------
TcasGr20        ------------------------------------------------------------
TcasGr79        ------------------------------------------------------------
TcasGr15        ------------------------------------------------------------
TcasGr60        ------------------------------------------------------------
AgamGr38        ------------------------------------------------------------
TcasGr51        ------------------------------------------------------------
DmelGr39b       ------------------------------------------------------------
AgamGr28        ------------------------------------------------------------
TcasGr61        ------------------------------------------------------------
TcasGr50        ------------------------------------------------------------
AgamGr12        ------------------------------------------------------------
AgamGr36        ------------------------------------------------------------
AgamGr48        ------------------------------------------------------------
AgamGr49a       ------------------------------------------------------------
AgamGr49b       ------------------------------------------------------------
DmelGr2a        ------------------------------------------------------------
DmelGr32a       ------------------------------------------------------------
DmelGr68a       ------------------------------------------------------------
AmelGr7         ------------------------------------------------------------
AmelGr8         ------------------------------------------------------------
AmelGr9         ------------------------------------------------------------
TcasGr57        ------------------------------------------------------------
TcasGr41        ------------------------------------------------------------
AgamGr34        ------------------------------------------------------------
AgamGr35        ------------------------------------------------------------
AgamGr39        ------------------------------------------------------------
```

```
AgamGr40        ------------------------------------------------------------
AaegGr28d       ------------------------------------------------------------
AgamGr42        ------------------------------------------------------------
AgamGr41        ------------------------------------------------------------
AgamGr43        ------------------------------------------------------------
DmelGr33a       ------------------------------------------------------------
AgamGr44        ------------------------------------------------------------
AgamGr13        ------------------------------------------------------------
AgamGr45        ------------------------------------------------------------
AgamGr46        ------------------------------------------------------------
Dmelr93a        ------------------------------------------------------------
AmelGr6         ------------------------------------------------------------
AmelGr10        ------------------------------------------------------------
TcasGr28        ------------------------------------------------------------
TcasGr40        ------------------------------------------------------------
AgamGr3         ------------------------------------------------------------
AgamGr4         ------------------------------------------------------------
AgamGr6         ------------------------------------------------------------
AgamGr7         ------------------------------------------------------------
AgamGr47        ------------------------------------------------------------
DmelGr8a        ------------------------------------------------------------
AgamGr5         ------------------------------------------------------------
AgamGr8         ------------------------------------------------------------
DmelGr9a        ------------------------------------------------------------
DmelGr59f       ------------------------------------------------------------
DmelGr77a       ------------------------------------------------------------
DmelGr85a       ------------------------------------------------------------
TcasGr27        ------------------------------------------------------------
TcasGr48        ------------------------------------------------------------
AgamGr1         ------------------------------------------------------------
DmelGr10a       ------------------------------------------------------------
DmelGr59e       ------------------------------------------------------------
DmelGr10b       ------------------------------------------------------------
DmelGr89a       ------------------------------------------------------------
DmelGr57a       ------------------------------------------------------------
DmelGr47b       ------------------------------------------------------------
DmelGr58a       ------------------------------------------------------------
DmelGr58b       ------------------------------------------------------------
DmelGr98a       ------------------------------------------------------------
AaegGr93a       ------------------------------------------------------------
TcasGr19        ------------------------------------------------------------
DmelGr47a       ------------------------------------------------------------
DmelGr58c       ------------------------------------------------------------


TcasGr38        -------------------------------------------------
TcasGr46        QDCDFVDEIEMFSMFERFHVNKLHFLLQIIGGVTTYIIILIQLSASLKQ 1451
TcasGr1         -------------------------------------------------
TcasGr123       -------------------------------------------------
TcasGr62        -------------------------------------------------
TcasGr25        -------------------------------------------------
TcasGr59        -------------------------------------------------
AgamGr2         -------------------------------------------------
AaegGr66a       -------------------------------------------------
DmelGr66a       -------------------------------------------------
TcasGr11        -------------------------------------------------
TcasGr55        -------------------------------------------------
TcasGr21        -------------------------------------------------
TcasGr56        -------------------------------------------------
AaegGr          -------------------------------------------------
AaegGr28a       -------------------------------------------------
AgamGr33        -------------------------------------------------
DmelGr28bB      -------------------------------------------------
DmelGr28bC      -------------------------------------------------
DmelGr28bD      -------------------------------------------------
DmelGr28bA      -------------------------------------------------
DmelGr28bE      -------------------------------------------------
AgamGr25        -------------------------------------------------
AaegGr43a       -------------------------------------------------
DmelGr43a       -------------------------------------------------
```

```
AgamGr37b       --------------------------------------------------
AgamGr37d       --------------------------------------------------
AgamGr37a       --------------------------------------------------
AgamGr37c       --------------------------------------------------
AgamGr37e       --------------------------------------------------
AgamGr37f       --------------------------------------------------
AaegGr28c       --------------------------------------------------
AaegGr28b       --------------------------------------------------
AaegGr28e       --------------------------------------------------
TcasGr14        --------------------------------------------------
TcasGr49        --------------------------------------------------
AmelGr4         --------------------------------------------------
AmelGr5         --------------------------------------------------
TcasGr16        --------------------------------------------------
TcasGr22        --------------------------------------------------
TcasGr17        --------------------------------------------------
TcasGr150       --------------------------------------------------
TcasGr32        --------------------------------------------------
TcasGr54        --------------------------------------------------
TcasGr37        --------------------------------------------------
TcasGr53        --------------------------------------------------
TcasGr5         --------------------------------------------------
TcasGr12        --------------------------------------------------
TcasGr13        --------------------------------------------------
TcasGr104       --------------------------------------------------
TcasGr45        --------------------------------------------------
TcasGr98        --------------------------------------------------
TcasGr52        --------------------------------------------------
TcasGr105       --------------------------------------------------
TcasGr4         --------------------------------------------------
TcasGr44        --------------------------------------------------
AgamGr9a        --------------------------------------------------
AgamGr9c        --------------------------------------------------
AgamGr9b        --------------------------------------------------
AgamGr9d        --------------------------------------------------
AgamGr9e        --------------------------------------------------
AgamGr9f        --------------------------------------------------
AgamGr9g        --------------------------------------------------
AgamGr9i        --------------------------------------------------
AgamGr9h        --------------------------------------------------
AgamGr9j        --------------------------------------------------
AgamGr9l        --------------------------------------------------
AgamGr9k        --------------------------------------------------
AgamGr9m        --------------------------------------------------
AgamGr9n        --------------------------------------------------
AgamGr10        --------------------------------------------------
AgamGr11        --------------------------------------------------
DmelGr23aA      --------------------------------------------------
DmelGr23aB      --------------------------------------------------
DmelGr39aA      --------------------------------------------------
DmelGr39aB      --------------------------------------------------
DmelGr39aC      --------------------------------------------------
DmelGr39aD      --------------------------------------------------
DmelGr98c       --------------------------------------------------
DmelGr98d       --------------------------------------------------
DmelGr98b       --------------------------------------------------
AgamGr51        --------------------------------------------------
AgamGr52        --------------------------------------------------
DmelGr94a       --------------------------------------------------
DmelGr97a       --------------------------------------------------
DmelGr93b       --------------------------------------------------
DmelGr93c       --------------------------------------------------
DmelGr22d       --------------------------------------------------
DmelGr22e       --------------------------------------------------
DmelGr22a       --------------------------------------------------
DmelGr22b       --------------------------------------------------
DmelGr22c       --------------------------------------------------
DmelGr22f       --------------------------------------------------
DmelGr36a       --------------------------------------------------
DmelGr36c       --------------------------------------------------
```

```
DmelGr36b       -------------------------------------------------
DmelGr59d       -------------------------------------------------
DmelGr59a       -------------------------------------------------
DmelGr59b       -------------------------------------------------
AgamGr26        -------------------------------------------------
AgamGr27        -------------------------------------------------
AgamGr29        -------------------------------------------------
AgamGr31        -------------------------------------------------
AgamGr30        -------------------------------------------------
AgamGr32a       -------------------------------------------------
TcasGr2         -------------------------------------------------
TcasGr9         -------------------------------------------------
TcasGr7         -------------------------------------------------
TcasGr30        -------------------------------------------------
TcasGr29        -------------------------------------------------
AaegGr64f       -------------------------------------------------
AaegGr64a3      -------------------------------------------------
AgamGr14        -------------------------------------------------
AgamGr18        -------------------------------------------------
AaegGr64d       -------------------------------------------------
AgamGr17        -------------------------------------------------
AaegGr64c       -------------------------------------------------
DmelGr64c       -------------------------------------------------
DmelGr64d       -------------------------------------------------
AgamGr20        -------------------------------------------------
AaegGr61a       -------------------------------------------------
AgamGr21        -------------------------------------------------
AaegGr64a       -------------------------------------------------
DmelGr64a       -------------------------------------------------
DmelGr61a       -------------------------------------------------
AgamGr15        -------------------------------------------------
AaegGr1         -------------------------------------------------
DmelGr5a        -------------------------------------------------
DmelGr64f       -------------------------------------------------
DmelGr64e       -------------------------------------------------
AmelGr1         -------------------------------------------------
AmelGr2         -------------------------------------------------
AgamGr16        -------------------------------------------------
AaegGr64e       -------------------------------------------------
TCasGr6         -------------------------------------------------
TcasGr26        -------------------------------------------------
TcasGr33        -------------------------------------------------
AgamGr23        -------------------------------------------------
AaegGr21a       -------------------------------------------------
TcasGr10        -------------------------------------------------
TcasGr39        -------------------------------------------------
AgamGr22        -------------------------------------------------
AaegGr21b       -------------------------------------------------
DmelGr21a       -------------------------------------------------
AgamGr24        -------------------------------------------------
AaegGr63a       -------------------------------------------------
DmelGr63a       -------------------------------------------------
TcasGr3         -------------------------------------------------
DmelOr83b       -------------------------------------------------
AgamOr7         -------------------------------------------------
HvirOr2         -------------------------------------------------
TcasOr16        -------------------------------------------------
TcasOr22        -------------------------------------------------
AmelOr2         -------------------------------------------------
TcasGr31        -------------------------------------------------
TcasGr35        -------------------------------------------------
DmelGr59c       -------------------------------------------------
DmelGr92a       -------------------------------------------------
DmelGr28a       -------------------------------------------------
DmelGr64b       -------------------------------------------------
TcasGr71        -------------------------------------------------
AgamGr19        -------------------------------------------------
AaegGr64b       -------------------------------------------------
DmelGr93d       -------------------------------------------------
TcasGr34        -------------------------------------------------
```

```
TcasGr43        --------------------------------------------------
TcasGr47        --------------------------------------------------
AmelGr3         --------------------------------------------------
AgamGr32b       --------------------------------------------------
AgamGr50        --------------------------------------------------
TcasGr20        --------------------------------------------------
TcasGr79        --------------------------------------------------
TcasGr15        --------------------------------------------------
TcasGr60        --------------------------------------------------
AgamGr38        --------------------------------------------------
TcasGr51        --------------------------------------------------
DmelGr39b       --------------------------------------------------
AgamGr28        --------------------------------------------------
TcasGr61        --------------------------------------------------
TcasGr50        --------------------------------------------------
AgamGr12        --------------------------------------------------
AgamGr36        --------------------------------------------------
AgamGr48        --------------------------------------------------
AgamGr49a       --------------------------------------------------
AgamGr49b       --------------------------------------------------
DmelGr2a        --------------------------------------------------
DmelGr32a       --------------------------------------------------
DmelGr68a       --------------------------------------------------
AmelGr7         --------------------------------------------------
AmelGr8         --------------------------------------------------
AmelGr9         --------------------------------------------------
TcasGr57        --------------------------------------------------
TcasGr41        --------------------------------------------------
AgamGr34        --------------------------------------------------
AgamGr35        --------------------------------------------------
AgamGr39        --------------------------------------------------
AgamGr40        --------------------------------------------------
AaegGr28d       --------------------------------------------------
AgamGr42        --------------------------------------------------
AgamGr41        --------------------------------------------------
AgamGr43        --------------------------------------------------
DmelGr33a       --------------------------------------------------
AgamGr44        --------------------------------------------------
AgamGr13        --------------------------------------------------
AgamGr45        --------------------------------------------------
AgamGr46        --------------------------------------------------
Dmelr93a        --------------------------------------------------
AmelGr6         --------------------------------------------------
AmelGr10        --------------------------------------------------
TcasGr28        --------------------------------------------------
TcasGr40        --------------------------------------------------
AgamGr3         --------------------------------------------------
AgamGr4         --------------------------------------------------
AgamGr6         --------------------------------------------------
AgamGr7         --------------------------------------------------
AgamGr47        --------------------------------------------------
DmelGr8a        --------------------------------------------------
AgamGr5         --------------------------------------------------
AgamGr8         --------------------------------------------------
DmelGr9a        --------------------------------------------------
DmelGr59f       --------------------------------------------------
DmelGr77a       --------------------------------------------------
DmelGr85a       --------------------------------------------------
TcasGr27        --------------------------------------------------
TcasGr48        --------------------------------------------------
AgamGr1         --------------------------------------------------
DmelGr10a       --------------------------------------------------
DmelGr59e       --------------------------------------------------
DmelGr10b       --------------------------------------------------
DmelGr89a       --------------------------------------------------
DmelGr57a       --------------------------------------------------
DmelGr47b       --------------------------------------------------
DmelGr58a       --------------------------------------------------
DmelGr58b       --------------------------------------------------
DmelGr98a       --------------------------------------------------
```

```
AaegGr93a       --------------------------------------------------
TcasGr19        --------------------------------------------------
DmelGr47a       --------------------------------------------------
DmelGr58c       --------------------------------------------------
```

D

```
TcasOr16      ------------------------------------MMKFKVTGLVADLMPNIRLIQASG 24
AmelOr2       ------------------------------------MMKFKQQGLIADLMPNINLMKATG 24
AgamOr7       ----------------------------------MQVQPTKYVGLVADLMPNIRLMQASG 26
AaegOr7       -------------------------------------------------MPNIRLMQGFG 11
DmelOr83b     --------------------------------MTTSMQPSKYTGLVADLMPNIRAMKYSG 28
BmorOr2       -----------------------------------MMTKVKTQGLVTDLMPCIRLLQAAG 25
BmorOr2a      -----------------------------------MMTKVKTQGLVTDLMPCIRLLQAAG 25
HvirOr2       -----------------------------------MMTKVKAQGLVSDLMPNIKLMQMAG 25
AgamOr56      ------------------------------------MDIVSKQGEFPLVKLSTKLLRIIR 24
AgamOr57      ------------------------------------MDIVSKQGEFPLVKLSTKLLRIIR 24
AgamOr26      ------------------------------------MDSNSKKDTFPLIQISTKMLQIIR 24
AgamOr27      ------------------------------------MKPSCKEGVFPLINLSIKLLKIVG 24
HvirOr9       -------------------------------------MVDQFQKCLKSVNLYLKFIGL-- 21
HvirOr7       ------------------------------------------------------------
DmelOr85b     -----------------------------------------MEKLMKYASFFYTAVG--- 16
DmelOr85c     ------------------------------------------MKFMKYAVFFYTSVG--- 15
DmelOr85d     ---------------------MLTKKDTQSAKEQEKLKAIPLHSFLKYANVFYLSIG--- 36
DmelOr67a     ---------------------------MDNVAEMPEEKYVEVDDFLRLAVKFYNTLG--- 30
DmelOr67c     ------------------------------METAKDNTARTFMELMRVPVQFYRTIG--- 27
DmelOr92a     -------------------------MLFRKRKPKSDDEVITFDELTRFPMTFYKTIG--- 32
DmelOr69a     ---------------------------------------MQLHDHMKYIDLGCKMACIPR 21
DmelOr69b     ---------------------------------------MQLEDFMRYPDLVCQAAQLPR 21
TcasOr6       ------------------------------------------------------------
TcasOr11      ---------------------------------------------------------MGK 3
AgamOr41      --------------------------------------------------MSEHPIHTFD 10
AgamOr42      ---------------------------------------------METHIGKQHSLERFQ 15
AgamOr43      -------------------------------------------------MTFTLSHDLYV 11
AgamOr44      -------------------------------------------------MTFTLSHDLYV 11
AgamOr66      ----------------------------------------------------MEASEKFS 8
AgamOr67      ----------------------------------------------------MEASEKFC 8
AgamOr72      ----------------------------------------------------MANVEGFR 8
AgamOr73      ----------------------------------------------------MANVEEFR 8
AgamOr71      ----------------------------------------------------MANVKDFR 8
AgamOr74      ----------------------------------------------------MANVEGFR 8
AgamOr70      ----------------------------------------------------MATIESFR 8
AgamOr69      ----------------------------------------------------MATVESFR 8
TcasOr9       ------------------------------------------------------------
TcasOr18      -------------------------------------------------------MAKLE 5
DmelOr83a     ------------------------MKSTFKEERIKDDSKRRDLFVFVRQTMCIAAMYPFG 36
DmelOr85e     ---------------MASLQFHGNVDADIRYDISLDPARESNLFRLLMGLQLANGTKPSP 45
TcasOr24      ----------------------------------------------MTEEKQLRICLSSC 14
TcasOr25      ----------------------------------------------MTEEKELRLCLWSC 14
TcasOr19      ------------------------------------------------------MVLLPT 6
TcasOr22      -----------------------------------------------MAKDSSPVLQESI 13
TcasOr23      -----------------------------------------------MAKDTSPVLRESI 13
AmelOr168     -----------------------------------------------MNFQNLNRLNALA 13
AmelOr170     -----------------------------------------------MNFQNLNRLNAFA 13
AmelOr169     ---------------------------------------------MKMNFQNLNRLNTFV 15
DmelOr65b     ---------------------------------------------MEASHSSIYYWREQM 15
DmelOr65c     ---------------------------------------------MESSYSAVYYWREQM 15
DmelOr65a     --------------MTELRSERKNGNWDRLFGPFFESWAVFKAPQAKSRHIIAYWTRDQL 46
TcasOr17      -------------------------------------------MSNFSWKAAVETNITTL 17
TcasOr21      -------------------------------------------MEKYDWMQAIKTNILIL 17
TcasOr56      --------------------------------------MTATKSLKEIPPIYLRVHLTVL 22
TcasOr15      ----------------------------------------MSKNLKEIPPVYLKVHLTVL 20
AgamOr9       ----------------------MVRLF-FSKTRVT-KLFTRKDERPEMFLPSLRMILFIF 36
AgamOr65      ----------------------MVKFFRCCKARVSPELLSKKNEKPEAFLPSLRMILFIF 38
BmorOr6       ---------------------------------------MTMRTNAKSFLFVPSKVLTLC 21
BmorOr10      ----------------------------------------MMGNSTDLFLDRTKRILNFF 20
AgamOr34      ------------------------------------------MYDPGRFIFPMRFSMWAW 18
AgamOr37      -----------------------------MEAEKQDTPTSDDPYRWGDYIRPVRITVWTW 31
DmelOr94a     ------------------------------------------MDKHKDRIESMRLILQVM 18
DmelOr94b     ------------------------------------------MES-TNRLSAIQTLLVIQ 17
DmelOr71a     --------------------------------------------MDYDRIRPVRFLTGVL 16
DmelOr46a     -----------------------------------------MSKGVEIFYKGQKAFLNIL 19
DmelOr46b     -----------------------------------------MVT--EDFYKYQVWYFQIL 17
HvirOr8       --------------------------------------------MVFRQIDCFKINMKFL 16
BmorOr8       ------------------------------------------MMTLVYQTDIFKPNVFFW 18
```

```
AmelOr68        -------------------------------------------------MTILQPIFNIL 11
AmelOr69        -------------------------------------------------MQLLRTIYHLL 11
AmelOr70        -------------------------------------------------MQALQWTRFLL 11
AmelOr71        -------------------------------------------------MRILRWTFLLF 11
AmelOr72        -------------------------------------------------MHLLRWTFKLF 11
DmelOr19a       ------------------------------------------MDIS--KVDSTRALVNHW 16
DmelOr19b       ------------------------------------------MDIS--KVDSTRALVNHW 16
DmelOr33a       ------------------------------------------MDSR-RKVRSENLYKTYW 17
DmelOr33b       ------------------------------------------MDLKPRVIRSEDIYRTYW 18
DmelOr22a       --------------------------------MLSKFFPHIKEKPLSERVKSRDAFIYLD 28
DmelOr22b       --------------------------------MLSQFFPHIKEKPLSERVKSRDAFVYLD 28
DmelOr42b       -----------------------------------MVFELIRPAPLTEQKRSRDGCIYLY 25
DmelOr59b       ----------------------------------MAVFKLIKPAPLTEKVQSRQGNIYLY 26
DmelOr59c       --------------------------------MTKFFFKRLQTAPLDQEVSSLDASDYYY 28
DmelOr98a       -----------------------------------MLFNYLRKPNPTNLLTSPDSFRYFE 25
DmelOr42a       -------------------------------MDLRRWFPTLYTQSKDSPVRSRDATLYLL 29
DmelOr85a       -----------------------------------MIFKYIQEPVLGSLFRSRDSLIYLN 25
DmelOr43b       ---------------------------------MFGHFKLVYPAPISEPIQSRDSNAYMM 27
AgamOr2         -----------------------------------------------MLIEECPIIGVNV 13
AgamOr10        -----------------------------------------------MEVLNCPLLSVNV 13
DmelOr30a       ------------------------------------------MELKSMDPVEMPIFGSTL 18
DmelOr49b       ----------------------------------------------MFEDIQLIYMN--I 12
DmelOr43a       -----------------------------------------------MTIEDIGLVGINV 13
AgamOr32        ------------------------------------------------MSQLNEFPAYQF 12
AgamOr35        ------------------------------------------------MSCVEDFLDAQM 12
DmelOr47a       ------------------------------------------------MDSFLQVQKSTI 12
DmelOr98b       ----------------------------------------------MLTDKFLRLQSALF 14
DmelOr9a        ------------------------------------MSDKVKGKKQEEKDQSLRVQILVY 24
AgamOr38        ------------------------------MIITVAPVTDAR-PQTPEDCGMFKFQRKIL 29
AgamOr39        ------------------------------MVSFGAAASNPRDAETTPHWDIFKLQRKIL 30
DmelOr24a       -----------------------------------------------MERHYFMVPKFAL 13
DmelOr45b       ------------------------------------MYPRFLSRNYPLAKHLFFVTRYSF 24
AmelOr74        ------------------------------------------MQGEGYTDVSLKVSQFLL 18
AmelOr86        ------------------------------------------MHATPYSDVSIVVSQFLL 18
AmelOr75        ------------------------------------------MRRRGSKDVSIIWTSFLM 18
AmelOr84        --------------------------------------------MRSTRDISIIWTSFLM 16
AmelOr87        ---------------------------MGAKAVAKVVAHSKLVARHSNKDFALSMTAFLM 33
AmelOr76        -----------------------------------------MKSKEVR-DLSITVTAFYM 18
AmelOr85        -----------------------------------------MSSNKVGGDLSITVMTFYM 19
AmelOr88        -----------------------------------------MNGRNVR-NLSITVTAFYM 18
AmelOr91        -----------------------------------------MS--FLESDVSVSLTSIFM 17
AmelOr92        -----------------------------------------MN--FLENDVAVSLTSIFM 17
AmelOr95        ---------------------------------------------MKNDDFSINLSSIFI 15
AmelOr93        -----------------------------------------MN--FLESDVSX----IFM 13
AmelOr77        -----------------------------------------MNGLLNGGDASMTMTAAFM 19
AmelOr94        ------------------------------------------MTLKYRGDVSFSLATFFL 18
AmelOr96        ------------------------------------------MNLKYRKDLAFTVASFYL 18
AmelOr79        ------------------------------------------MQAEYRLDISINLSTFLL 18
AmelOr83        ------------------------------------------MQTDNQLDISISLSTFFL 18
AmelOr80        ------------------------------------------MQTESQLDISINLSTFFL 18
AmelOr81        ------------------------------------------MQTESQVDISMNLSTFFL 18
AmelOr82        ------------------------------------------MQTESQVDISMNLSTFFL 18
AmelOr78        ------------------------------------------MQSENQLDVSITLSTFFL 18
AmelOr89        ------------------------------------------MQREVELDVSVNLAAFFL 18
AmelOr90        --------------------------------------------MEKELDISVNLSSFFL 16
AmelOr142       ------------------------------------------MKNRLTPEKAILFTKLSV 18
AmelOr146       ------------------------------------------MFRNATPEKAIAFTQFIV 18
AmelOr148       ------------------------------------------MLKYVTPEKGIYIVWLSV 18
AmelOr158       ------------------------------------------MLKQITPEKSIYIIWLSV 18
AmelOr147       ------------------------------------------MLKQVSPEKGIYIIWLSV 18
AmelOr150       ------------------------------------------MLKQLTPEKAIHITWISV 18
AmelOr151       ------------------------------------------MLKQLTPEKVIYITWVSV 18
AmelOr152       ------------------------------------------MLKQIISEKTIQIIWFSV 18
AmelOr154       ------------------------------------------MLKKVTPENVIYIIRLSV 18
AmelOr155       ------------------------------------------MLKKATPEKIIDIIRFSV 18
AmelOr157       -------------------------------------------MRRARPEKSVYLVWLSV 17
AmelOr156       ------------------------------------------MIEQVMLKRVIYITWLSV 18
AmelOr153       ------------------------------------------MVKEMIPEKTIHITWLSV 18
AmelOr144       -----------------------------------------MGMLNMDIRQVLHILELTG 19
AmelOr145       -----------------------------------------MGMLNMNIRQVFYILELTG 19
```

```
AmelOr143   ----------------------------------------------MNIRQILYILELIG 14
AmelOr1     ---------------------------------MENTTNYRNIHYKSDAEYTVHVAKTLL 27
AmelOr3     ----------------------------MSVKTARNIRDYHNIHYRSDAEYTVRVAKILL 32
AmelOr6     ---------------------------------------METK--EKDLKQAFY-AQPFL 18
AmelOr7     ---------------------------------------METK--EKDLKQAFY-AQPFL 18
AmelOr4     ---------------------------------------METKHTEKDLKQAFY-VQTFL 20
AmelOr5     ---------------------------------------METKHTEKDLKQAFY-AQSFL 20
AmelOr8     --------------------------------------MVQIRNAREGINHTFWFAYPLS 22
AmelOr9     --------------------------------------MARIRNAREGINHTLWFAYPLS 22
AmelOr11    --------------------------------------MVQIRNAKEGLKHTFWFAYPFS 22
AmelOr12    --------------------------------------MARIRNAKDGIRHTFWFAYPFS 22
AmelOr10    --------------------------------------MVQIRNAKEGLRHTFWFAYPFS 22
AmelOr14    --------------------------------------MSRVGKAENGMRHTVWFAYPLL 22
AmelOr15    --------------------------------------MSRIGNAEDGMRHTIWFAYMLL 22
AmelOr13    ----------------------------------------------MGQPYSLKLVYPLL 14
AmelOr16    -----------------------------------MENISGIAKAEEDLKYATRFVKPIL 25
AmelOr17    -----------------------------------MENISGIAKAEEDLKYATRFVKPIM 25
AmelOr18    ----------------------------MNAEKLMIEGKPPNANYKNDLSFNVRLNVWTL 32
AmelOr19    ----------------------------MNMEHFIVEKKSYNASYKNDLFFNVQLNVWTL 32
AmelOr22    --------------------------MEKSKINS-ISCIQTNHDYKRNVNLSIQWSRWIL 33
AmelOr24    -----------------------------------MSYTKTDHDYKRNVNLSIQWSRWIL 25
AmelOr20    --------------------------MEKSKDKANQKFYLTDYEYQKNVNLSIQYNRWLL 34
AmelOr25    --------------------------MEK------QQYVIAQDDGK-KANLSIQWNRWLL 27
AmelOr29    --------------------------MKN------QQVVITQDDYKRKTNLSIQWNRWLL 28
AmelOr30    --------------------------MEK------NRSIIGHDDYERNVNLSIRWNRFLL 28
AmelOr21    -----------------------------------MSSVKIDQDYKSNVNLSIKYSRRIS 25
AmelOr23    -----------------------------------MSSVKINQDIKNNINFSIKYSRLIL 25
AmelOr35    ---------------------------------MLVLKDSSSVSYSKDWIYSVQINRWLL 27
AmelOr27    ---------------------------------MMN-QTAITEEIKTNSDYSLQLNRWFL 26
AmelOr28    ---------------------------------MSNRSVAIKTDPDTNSDYCLQLNRWFL 27
AmelOr33    --------------------------------MMTSKSVPIEQDNHSLSNYSVQLNRWFL 28
AmelOr34    --------------------------------MMIDKFVPIEQDNHSLSNHSVQLNRWLL 28
AmelOr32    ---------------------------------MIDKFASIQQTNNNLSNYSIQLNRWFL 27
AmelOr31    ---------------------------------MTSKSVISEESFDSLCDYSLQLNRWLL 27
AmelOr36    ---------------------------------MTDDISAIQKKFGSLNEYSIQVNRWLS 27
AmelOr37    --------------------------------MMADDIATVQKEFENLNEYSIQFNKWFS 28
AmelOr38    --------------------------------MMADDIATVQKEFNNLNEYSIQFNKWFS 28
AmelOr39    --------------------------------MMADDIATVQKEFDNLNEYSIQFNKWFA 28
AmelOr47    ---------------------------------MADDIVAIQKKFGSLNEYSIQLNRWFS 27
AmelOr48    ---------------------------------MADDIVAVQKKFGSLNEYSIQLNRWLS 27
AmelOr40    ---------------------------------MADDITAIQKKFGSLNEYSIQLNRWLS 27
AmelOr43    --------------------------------MMADDIAAIQKKFGSLNEYSIQLNRWFS 28
AmelOr45    ---------------------------------MADDIAAIQKKFGSLNEYSIQLNRWLS 27
AmelOr41    ---------------------------------MADDIVAIQKKFGSLNEYSIQVNRWLS 27
AmelOr42    ---------------------------------MADDIVAIQKKFGSLNEYSIQVNRWLS 27
AmelOr44    ---------------------------------MADDIVAIQKKFGSLNEYSIQVNRWLS 27
AmelOr46    ---------------------------------MSDDLVEVEKKFGSLNEYSIQFNRWIL 27
AmelOr49    ---------------------------------MADDLAKIEKKFGDLSEYSIQFNRWIL 27
AmelOr50    ---------------------------------MTNDINVAKQRSDNLSEYSIKLSRWYL 27
AmelOr26    -----------------------------MMNQLNEQSVLMPVSYARDYEYSIQVNRWLL 31
AmelOr53    ---------------------------MHDRSHDNINGQLKNSHYKSDIHYTLQMCQWLL 33
AmelOr54    ---------------------------MHDRSYHDIESQLKNSYYKSDIHYTLQMCQWLL 33
AmelOr52    ---------------------------MFDRSYN--NSQLKNIHYENDIHYTLQMCQWLL 31
AmelOr51    -----------------------------MRSTNNIDNLPLNDRYESDIQYTFQFCHWIL 31
AmelOr55    ---------------------------MHFSVRNLIN-KPRNPNYEKDITYVMKHNKWVL 32
AmelOr57    ---------------------------MHVSVRDPIN-ELRNPNYEKDIAYVTKHNKWVL 32
AmelOr56    ---------------------------MYLSIQNPIN-EPRNPNYEKDIAYVTKYNKWVL 32
AmelOr58    ---------------------------MHLFVRDQTN-QPRNLNYEKDIVYVTKHNKWIL 32
AmelOr59    ---------------------------MHPITLNESDCKARNLKYKEDIAYVTKHNKWIL 33
AmelOr60    ---------------------------MHLTILNESDYRARNLKYKEDIAYVTKHSKWIL 33
AmelOr61    ---------------------------MHLTTLNKNNCKVRNLKYKEDIAYITKHNKWIL 33
AmelOr63    ------------------------------------MLKKMKTTSNKDFAYAMTPLKFLA 24
AmelOr64    ----------------------------------------MKTTSNKDFAYAMTPLKFLS 20
AmelOr66    ----------------------------------------MKTTLNKEFAYAMTPLKFLS 20
AmelOr65    ----------------------------------------MKTTSNKDFTYAMTLLKFLS 20
AmelOr67    ----------------------------------------MKTTSNKDFTYAMIPLKFLS 20
AmelOr114   -----------------------------------------------------------M 1
AmelOr115   ------------------------------------------------MDFAMGWNRFNL 12
AmelOr118   --------------------------------------------------MINRPLEYSL 10
AmelOr164   ---------------------------------------------MAIKSIINRPVEISL 15
```

```
AmelOr163       ----------------------------------------------MFKTIITYPVEVCL 14
AmelOr165       ---------------------------------------------MLVLNTLSPSVKFGL 15
AmelOr166       ---------------------------------------------MTSINTISRSVKYGL 15
AmelOr167       ---------------------------------------------MIPIRSISHPIVIGL 15
DmelOr35a       -----------------------------------MVRYVPRFADGQKVKLAWPLAVFR- 24
DmelOr74a       ---------------------------------MSFHRYRPRLPGGELAPMPWPVSLYRV 27
AgamOr3         --------------------MPSERLRLITSFGTPQDKRTMVLPKLKDETAVMPFLLQIQ 40
AgamOr5         ----------------------------------------MVLPKLSEPYAVMPLLLRLQ 20
AgamOr13        ----------------------------------------MNFLRQEQPAGMPHISIKLL 20
AgamOr15        ----------------------------------------MNFLRQEQPAGMPHISIKLL 20
AgamOr17        ----------------------------------------MNFLRQEHPAGMPHISIKLL 20
AgamOr16        ----------------------------------------MRFFLIEKPPGVPHIALKLL 20
AgamOr55        ---------------------------------------MIRFFTIHKPPGVPHLALKLL 21
AgamOr18        ----------------------------------------MTSFKPTDDTDVMPLVLRLL 20
AgamOr14        ----------------------------------------MLFQELPNDRAVLPLLLYIQ 20
AgamOr51        ----------------------------------------MLFQALPNDRAVLPLLLYIQ 20
AgamOr12        ----------------------------------------MEKLRPEDPKAVMPYAKRLL 20
AgamOr19        ----------------------------------------MLRLSPEDPKAVMPVVKRLL 20
AgamOr20        ----------------------------------------MLRLSPEDPKAVMPVVKRLL 20
AgamOr21        ----------------------------------------MLRLSPEDPKAVMPFAKRLL 20
AgamOr50        ----------------------------------------MLSLRPEDPKAVMPFAKRLL 20
AgamOr29        ----------------------------------------MKLFQIDDPREVVPIGCRLL 20
AgamOr53        ----------------------------------------MKLLELDNPREAIAIGCRLL 20
AgamOr30        ----------------------------------------MKFFQIDDTREILPIGCRLL 20
AgamOr46        ---------------------------------------MVVFEPLDDPLKVLPLPLKLL 21
AgamOr47        ---------------------------------------MVVFDPLDDPLKVLPLPLKLL 21
AgamOr48        ---------------------------MVN----------LFARTPADNFRVMPYNLRLF 23
AgamOr49        ---------------------------MFRRLTHPTSPKQQHHRDRACDFTVMPHGLWLL 33
AgamOr61        -------------------------MWHHQVQL-WFRFLLGRFTTFTDSSDYFGLYKTLA 34
AgamOr62        ------------------------MVRSQPFRLSLVRKYWDKYFTFTDTVDYFNLLNTFG 36
AgamOr63        -------------------------MYLTQLFASMRTQFLHRITHYTKHSDFFIMQRYFE 35
AgamOr60        ---------------------------MIDLVTRIRRKLLALERKLANDLDQFVLLHHLT 33
AgamOr75        -------------------------MTVVHRIVSFGYNLLQRHFNVGHPTEQFFLLRCLD 35
AgamOr76        -------------------------MTVVHRIVSFGYNLLQRHFNVGHPTEQFFLLRCLD 35
AgamOr78        -------------------------MTVVHRIVSFGYNLLQRHFNVGHPTEQFFLLRCLD 35
AgamOr77        ----------------------------------------MGTANQPTSICFLRWIGWMD 20
AgamOr79        ----------------------------------------MGTANQPSSICFLRWIGWMD 20
AmelOr99        --------------------------------MDIFQKIEKQHNEIYDIPY-YKMMEKYI 27
AmelOr101       --------------------------------MDIFQKTKKQHNEIYDIPY-YKMMEKYI 27
AmelOr102       --------------------------------MNIFQKTRRQCPDIFDIPY-YKMVEKYF 27
AmelOr103       --------------------------------MDIFQKTERQYPEIFDIPY-YKMVEKYF 27
AmelOr104       --------------------------------MDVFQKTRNKCINIFDIPY-YKLLEKYM 27
AmelOr98        --------------------------------MDMFQKTGKEYSNIFDIPY-YKVLKKYL 27
AmelOr100       ------------------------------------------------------------
AmelOr97        ------------------------------------------------------------
AmelOr105       --------------------------------MSMLQKSNEQEYNAFDIAY-YKTLKLYL 27
AmelOr109       -----------------------------------------MDERAIEDQY-LKINKFFG 18
AmelOr110       -----------------------------------------MDKQTIENQY-LKINKFFG 18
AmelOr111       -----------------------------------------MDKRVIEDQY-LKINKFFG 18
AmelOr108       -----------------------------------------MNKQAIEDQY-LRINKFFG 18
AmelOr107       -----------------------------------------MDQQAMEELY-LKDNKFFG 18
AmelOr112       -----------------------------------------MDARTVEKNF-LKVNKIFG 18
AmelOr113       -----------------------------------------MDSDILEKRF-LKITKRFA 18
AmelOr106       -----------------------------------------MDVR-LEERY-LKINKIYS 17
AmelOr122       -----------------------------------------MNMDVFDKQ--YRIYRIIL 17
AmelOr125       -----------------------------------------MNMDVFDKQ--YRIYRIIL 17
AmelOr123       -------------------------------------------MNVFDNQ--YRTYRIIL 15
AmelOr126       -------------------------------------------MNVFDNQ--YRTYRIIL 15
AmelOr127       -------------------------------------------MNVFDNQ--YRTYRIIL 15
AmelOr128       -------------------------------------------MNVFDNQ--YRTYRTVL 15
AmelOr129       -------------------------------------------MNVFDNQ--YRTYRIIL 15
AmelOr133       ------------------------------------------------------------
AmelOr134       -------------------------------------------MDIFDKH--YYSYRTVL 15
AmelOr131       -------------------------------------------MDVFDKY--YHTYRIVL 15
AmelOr132       -------------------------------------------MDVFDKY--YHSYRTVL 15
AmelOr135       -------------------------------------------MDVFDKQ--YHSYRTVM 15
AmelOr136       -------------------------------------------MNVFDKH--YHTYRTLM 15
AmelOr138       ------------------------------------------------------------
AmelOr130       -------------------------------------------MNVFDNQ--YRIYRIIL 15
AmelOr137       -------------------------------------------MDIFDKR--YCTYRTML 15
```

```
AmelOr139       -------------------------------------------MDIFDKHQSYHSYRTIM 17
BmorOr20        ----------------------------MIFVDDAVIG-----IKDPREYRHLRVLRTSL 27
BmorOr3         ----------------------------MIFVDDAVIG-----IKDPREYRHLRVLRTSL 27
BmorOr18        ----------------------------MIFVDDAVIG-----IKDPREYRHLRVLRTSL 27
HvirOr6         -----------------------MNLRKFLFENEAVEG-----INSPADYLYTRILRFNL 32
BmorOr23        --------------------------MLLYYPNTQVK----EKVNNVEEFTYIKFLKSFC 30
BmorOr16        --------------------------MLLYYPNTQVK----EKVNNVEEFTYIKFLKSFC 30
BmorOr4         --------------------------MLLYYPNTQVK----EKVNNVEEFTYMKFLKSFC 30
BmorOr5         --------------------------MLLYHPNTQVE----EKVNNVEEFTYMKFLKSFC 30
BmorOr22        --------------------------MFKIIKNIIVENDALKQVEKPQEFQYMKWVQYHL 34
BmorOr17        --------------------------MFKIIKNIIVENDALKQVEKPQEFQYMKWVQYHL 34
BmorOr1         --------------------------MLLSFKDDSRS----PDIQKPQNFQYMKILRFNL 30
BmorOr1a        --------------------------MLLSFKDDSRS----PDIQKPQNFQYMKILRFNL 30
BmorOr1b        --------------------------MLLSFKDDSRS----PDIQKPQNFQYMKILRFNL 30
BmorOr19        -------------------------MKEEYYLQHPRT------QLFYKVLAHVSTIESTI 29
BmorOr15        -------------------------MKEEYYLQHPRT------QLFYKVLAHVSTIESTI 29
TcasOr5         ---------------------------------------MVWSPKDVYDLTLPILPAWKY 21
TcasOr7         ----------------------------------------MYRLVASFVNYMGAESAQQV 20
HvirOr4         ------------------------------------------------MCVYSYILVTVL 12
BmorOr13        MTMSIKPRLQCMVPPSLALALRVSRLAGIAPLKFVAKQSNIMIRLSTSLCVYSYLLVTAL 60
DmelOr10a       ----------------------------------MSEWLRFLKRDQQLDVYFFAVPRLSL 26
DmelOr33c       ----------------------------------------------MVIIDSLSFYRPFW 14
DmelOr7a        --------------------------------MAVSTRVATKQEVPESRRAFRNLFNCFY 28
DmelOr22c       ---------------------------------------MTDSGQPAIADHFYRIPRISG 21
AgamOr1         ----------------------------------MKLNKLNPRWDAYDRRDSFWLQLLCL 26
AgamOr4         -------------------------------------MKFELFQKYSSPDTVLSFVLRLL 23
DmelOr47b       ------------------------MNDSGYQSNLSLLRVFLDEFRSVLRQESPGLIPRLA 36
AmelOr73        -------------------------------------------------MHKLSLSFALL 11
AgamOr68        -----------------------------------------MYTAIEFYNASFNRLKLSS 19
DmelOr59a       --------------------------------------------MAEVRVDSLEFFKSHW 16
DmelOr23a       -----------------------------------------MKLSETLKIDYFRVQLNAW 19
AgamOr31        -------------------------------------------MLAAETVDFFRVQSICL 17
DmelOr2a        -----------------------------------------MEKQEDFKLNTHSAVYYHW 19
AmelOr62        --------------------------------------MGKRKESIDERIRNFMVQKMVL 22
AmelOr121       ------------------------------------MHTSESKKYSKDYEWAVRLNRFSL 24
AmelOr140       ----------------------------------------LRGLYSVWGVNYDAVIECMP 20
TcasOr12        -------------------------------------------MAHNLDVDLTEFVRFNI 17
AgamOr8         --------------------------------------MDPPTDELVRFESFIRVPEIFF 22
BmorOr9         -------------------------------------------MDIPKFEELLKQIKMNF 17
AgamOr40        -------------------------------------MLRPTSEDDQTLLINFRLLERVL 23
TcasOr63        ---------------------------------------MGFMIQDYDLRNAFSLERKLM 21
TcasOr4         -------------------------------------------MDEEFLIGTFETEKKFL 17
TcasOr13        ------------------------------------MSAMSTVIVSGEMFDLFSPVLYLS 24
AgamOr24        -------------------------------------------------MDLTAGIESFN 11
AgamOr54        --------------------------MYYLEQLRALVRRYLERRSPDPRIQHTFIVQSIN 34
AgamOr23        -------------------------------------------MRWRKDETGLHTPQCTV 17
AgamOr64        -------------------------MFHPSRWMAKCRVWYAATFKFSADADYFFLVQPLC 35
DmelOr82a       ------------------------------------------------MGRLFQLQEYCL 12
AmelOr116       ---------------------------------MTNHLEKQIKLKKINSNKHLQNNLSII 27
AmelOr160       -----------------------------------MRRPISSYVELFYDKNVISWSKRLL 25
TcasOr10        --------------------------------------------MATFLLYKTEMLDLIK 16
TcasOr26        ---------------------------------------------------MMESTVTRL 9
AmelOr117       ----------------------------------------MKKPFNKSIDYYILPNKIFC 20
AmelOr120       ---------------------------------------MSNQTNTMNIRNYIFINQLVL 21
BmorOr7         ---------------------------------------------MSLSGSSVFRHLFLL 15
AgamOr6         -------------------------------------------MVRLSFEETLRNTNLML 17
DmelOr45a       -----------------------------------------------MDASYFAVQRRAL 13
DmelOr67d       ---------------------------------------MLKMAKVEPVERYCKVIRMIR 21
DmelOr83c       -----------------------------------------MSTSESPSSRFRELSKYIN 19
AmelOr162       -----------------------------------------MKRLMDIMQEDWKFHARLR 19
HvirOr3         ------------------------------------------------------------
TcasOr20        ----------------------------------------------MMDETYLQFFVKSF 14
AgamOr11        ----------------------------------MELKEEWILPDVVYANPLLKRTLLGL 26
DmelOr56a       ----------------------------------MFKVKDLLLSPTTFEDPIFGTHLRYF 26
AgamOr28        ----------------------------MARLVLHEVRYVLMAMLYISRGMATKIQNSPI 32
AgamOr58        --------------------------------MSALVHLAKDHIQRIADGGQFVIVNGMD 28
AgamOr59        ----------------------------------MFSASAEMEWFRQQRHAICHLREGSD 26
DmelOr1a        -----------------------------------------MSKLIEVFLGNLWTQRFTF 19
DmelOr13a       -----------------------------------------MFYSYPYKALSFPIQCVWL 19
AmelOr161       ----------------------------------------MGEFRNEEYDQLIKPIMITG 20
```

```
AmelOr141       ---------------------------------------MIDEKTKREFDKTIDLNLFLL 21
AgamOr52        --------------------------------------------SLTPTDAGVQLQTERL 16
AgamOr36        -----------------------------MQTGGVLSRMRRALGRFYVERDFFRPYEILL 31
AgamOr25        --------------------------------------------MEQEINQIISFLRRPL 16
AgamOr45        -------------------------------------------MPRATSNSAANLERLVI 17
TcasOr3         ---------------------------------------MSKSEKIHTLATYFDSNIAFL 21
DmelOr88a       -----------------------------------MKPTEIKKPYRMEEFLRPQMFQEVA 25
AgamOr22        ------------------------------------------MFSLGKSHQYLELSYNRI 18
DmelOr49a       ------------------------------------------MEKLRSYEDFIFMANMMF 18
DmelOr85f       -----------------------------------------MEPVQYSYEDFARLPTTVF 19
AmelOr119       ----------------------------------MHTQRDTSEITYSHDTATSRKLFYLL 26
DmelOr63a       -----------------------------------MYSPEEAAELKRRNYRSIREMIRLS 25
DmelOr67b       --------------------------------------MQDQLDHELERIDKLPKLGLLW 22
BmorOr21        ----------------------------------------MSNYVFKPFHETYRIITFTM 20
BmorOr11        -----------------------------------------MSVSNLKFEALFKPTTMSL 19
BmorOr12        ----------------------------------MHEFVINVQNETTKLYDQLNIILYIL 26
AgamOr33        -----------------------------------------MCILAGLLLTLAKKWHCVL 19
HvirOr1         -----------------------------------------MGPTSLKRNMFFWIPVKKN 19
HvirOr5         --------------------------------------------MSGHRSVTARLAGTIF 16
TcasOr1         --------------------------------------------MNNSRPANCTHRPKLS 16
TcasOr2         -----------------------------------MFKTVTREKVFQKFTSSIRMLLIQG 25


TcasOr16        HFMLNYHADNSGALHTLRL--GYCCMHLVFVLVQYGCNFVNLVL------ERGDVNDLAA 76
AmelOr2         HFMFNYYTDSS-TKHIHKI---YCIVHLVLILMQFGFCGINLMM------ESEDVDDLTA 74
AgamOr7         HFLFRYVTGPI----LIRK--VYSWWTLAMVLIQFFAILGNLAT------NADDVNELTA 74
AaegOr7         HFLFRYVNGPV----LIRK--LYSWWNLIMILLQYFAIMGNLVM------NTGDVNELTA 59
DmelOr83b       LFMHNFTGGSA----FMKK--VYSSVHLVFLLMQFTFILVNMAL------NAEEVNELSG 76
BmorOr2         HFLFNYHADTSGMNMLLRK--IYSSAHAVLIVVHYICMGINMAQ------YKDEVNELTA 77
BmorOr2a        HFLFNYHADTSGMNMLLRK--IYSSAHAVLIVVHYICMGINMAQ------YKDEVNELTA 77
HvirOr2         HFLFNYHSENAGMSNLLRK--IYASTHAILIFIHYACMGINMAK------YSDEVNELTA 77
AgamOr56        --FWNDSPGQR-----IPS--LGLLVTAIYPLIWLIPSWLFLVS------SQDNITRFMK 69
AgamOr57        --FWNDSPGQR-----IPS--FGLLVTAIYPLIWLIPSWLFLVS------SQDNITRFMK 69
AgamOr26        --LWNESSGQR-----MST--FGVLLIAVFPIMWLIPSWLFIVS------SQDNIKRLMK 69
AgamOr27        --FWSKTTGQR-----ISV--IGLLAVILYLLAWLIPNWLFIVS------SQDNITRLIK 69
HvirOr9         HLESKDTTKTFIERSRSHR--LYFAHFFSLNLEVVAQILWVLEA----VITRKSFVEITR 75
HvirOr7         -MNIKNKIVTPLDTIKYRW--LYTLNFLVVFSAIIGSVYYVILG----IKQGKNFIEVTS 53
DmelOr85b       IRPYTNGEESKMNKLIFHI--VFWSNVINLSFVGLFESIYVYSA----FMDN-KFLEAVT 69
DmelOr85c       IEPYTIDSRSKKASLWSHL--LFWANVINLSVIVFGEILYLGVA----YSDG-KFIDAVT 68
DmelOr85d       MMAYDHKYSQKWKEVLLHW--TFIAQMVNLNTVLISELIYVFLA----IGKGSNFLEATM 90
DmelOr67a       IDPYETG---RKRTIWFQI--YFALNMFNMVFSFYAEVATLVDR----LRDNENFLESCI 81
DmelOr67c       EDIYAHRSTNPLKSLLFKI--YLYAGFINFNLLVIGELVFFYNS----IQDFETIRLAIA 81
DmelOr92a       EDLYSDRDPNVIRRYLLRF--YLVLGFLNFNAYVVGEIAYFIVH----IMSTTTLLEATA 86
DmelOr69a       YQWKGRPTERQFYASEQRI--VFLLGTICQIFQITGVLIYWYCNGRLATETGTFVAQLSE 79
DmelOr69b       YTWNGRRSLEVKRNLAKRI--IFWLGAVNLVYHNIGCVMYGYFGDGRTKDPIAYLAELAS 79
TcasOr6         --------------MSPHD--SGTFARIRKIFSILVYTSTVVLSMAELFFNYKDLETVIR 44
TcasOr11        VKFTEPLEFLNVVGLNPEN--CSNFSLFRRVISLGFFLVVITLGLLELLLHFEGLETCSR 61
AgamOr41        RLIKRQRLLLKLIGIDSYD--PAFRIHSLTFMLVCLALTFFVISLYDLYLFRDDLFNFVY 68
AgamOr42        AMVAWQNRVLALFGCYMYL--GKERVSYRIVPICFMACSFVLLSLYSAVQARGNMGQAVL 73
AgamOr43        YNLHLIRWFASFVGLDIMA--PNYKPNILTFLAFFGLSISLIGEVYTVWYFWPILDKLME 69
AgamOr44        YNLHLIRWFASFVGLDIMA--PNYKPNILTFLAFFGLSISLIGEVYTVWYYWPNLVKLME 69
AgamOr66        QFERYIRTLCNIIGFDVMT--ETWKKSYRTYMSIFLCSQYLILMVYSLIIAS-DTFELFK 65
AgamOr67        QFERYLRTLCKIIGFDVMT--ETWKKSYQTYMSILLCSQYFILMVYSLIIAS-DTFELFK 65
AgamOr72        KLMKSLIVYSKVAGVEIWT--APGKFVPASYYVSFHITVYFVSTVWTLRKYIDDPIHMMK 66
AgamOr73        KLMKSLIVCSKVAGVEMWT--APGKLKPASYYVSFHITVYFVSTVWTLRKYIDDPIHMMK 66
AgamOr71        KLMKSLIVYSKVAGVELWT--APGKFTPASYYVSFHITVYFASTVWTLRKYIDDPIRMMK 66
AgamOr74        KLMKSLIVYSKVAGVEIWT--APGKFTPASYYVSFHITVYFASTVWTLRKYIDDPIHTMK 66
AgamOr70        KLLKYLIVYSKVPGVEMWT--APGKFKPASYYVSFHIIVYFVSTVWTLKKYSDDPIHMMK 66
AgamOr69        QLLQPLIFYSKGVGVEIWT--APGKFVPASYYLSLHVAIYFSSTVFTLIKYSDDTLHMMK 66
TcasOr9         ------------------------------------------------------------
TcasOr18        YLTGATFTLKCAVLYPIDS--NNPKIKKILYAVWAI-----FFILTFVTGFIQCFVFVCI 58
DmelOr83a       YYVNGS-GVLAVLVRFCDL--TYELFNYFVSVHIAG-----LYICTIYINYGQGDLDFFV 88
DmelOr85e       RLPKWWPKRLEMIGKVLPK--AYCSMVIFTSLHLGV-----LFTKTTLDVLPTGELQAIT 98
TcasOr24        FFLKWSFMWPTKSEEFRTS--KGLYFRLLAFVIISGLTFTAMIVMHLLKSVEAGDYDISE 72
TcasOr25        YYLKLSLMWPLKREEFKSS--KGLYLRLLVFVIISGSTFTAMIFMHLYKSLKVGSYDVSE 72
TcasOr19        DFGRFRHHPHIRSAGFDAP----------------------------LEAMEEG-MDISE 37
TcasOr22        EIMKYLQLWPQNEHTNLRR--RFCFVILLCSPLTLG-----LAAHLIVCIKDNLDVELSA 66
TcasOr23        EVMKYLQLWPQNERTNLRR--RYFIVIFLCSPLHLG-----LATHLVVCLKDNLDVDLSA 66
AmelOr168       NVVSGNFLPMTNINEKSSV--ISKIYFVIVWIIQLM-----YLASCTLGLFNVSWERALK 66
AmelOr170       NMVSGNFLPMTNINEKLST--ILKIYFVVAWIIELI-----YVAASFLGLFNVSGEKALK 66
```

```
AmelOr169       NAVSGNILPITDMKKRLSI--VLKIYSILVWTIELS-----YLAACILGLFNVSRERALK 68
DmelOr65b       KAMALFTTTE-------ER--LLPYRSKWHTLVYIQMVIFFASMSFGLTESMGDHVQMGR 66
DmelOr65c       KAMFLYTTSK-------ER--QMPYRSSWHTLVIIQATVCFLTMCYGVTESLGDKVQMGR 66
DmelOr65a       KALGFYMNSE-------QR--RLPRIVAWQYFVSIQLATALASLFYGISESIGDIVNLGR 97
TcasOr17        KILGLWPKG-----DETYK--LNFYTLYAVFGVIGLLCAHSFVQIFNIYFIVDDLEAFTS 70
TcasOr21        KIVGLWPD------SEDYK--FDFYALHASVWLSTLLVASTFFQGINIIFILDDVKALTG 69
TcasOr56        QILGIDILP-----VESVP--QNLFYTYTALIIS-TMCLFTIAEFLDMVLNYEDIYRLTF 74
TcasOr15        QILGIDILP-----NERIP--QTLFYTYSVLLIA-TMVVFTTAECLDLVLNYEDIYKLTF 72
AgamOr9         RMFYAWPDEH-----LEKS--ALWWYRAKGVLFRVFFIYLSAATQLAYNFTVTSREELFA 89
AgamOr65        RMFYAWPDEQS----LAPN--ALWWYRAKGVLFRTFFIYLSAAAQLAYNFTVTSREELFA 92
BmorOr6         GVWPVEKTSLF----SVIY--RSIMLSSQFCFLVFNGIYIGLMWG--------DLKAVSD 67
BmorOr10        AMWRSFEKPIP----LKVY--MAFIMTTQYLFLIFEIIYIVNVWG--------DMAEVSE 66
AgamOr34        KVCGFFNAP-------VPK--SVAYRVYCYAFYSCIMAVYLFVLLLNVFVPQPFEQRVFF 69
AgamOr37        KICGLYNG--------KPQ--TTRYRVYRAVFNIFLMVVYLFTLSLNVFVMQTFEQLVLY 81
DmelOr94a       QLFGLWPWSLKSEEEWTFT--GFVKRNYRFLLHLPITFTFIGLMWLEAFISSNLEQAG-Q 75
DmelOr94b       RWIGLLKWENEGED--GVL--TWLKRIYPFVLHLPLTFTYIALMWYEAITSSDFEEAG-Q 72
DmelOr71a       KWWRLWPR----KESVSTP--DWTN-WQAYALHVPFTFLFVLLLWLEAIKSRDIQHTA-D 68
DmelOr46a       SLWPQIERR-------WRI--IHQVNYVHVIVFWVLLFDLLLVL--HVMANLSYMSEVVK 68
DmelOr46b       GVWQLPTWA-------ADH--QRRFQSMRFGFILVILFIMLLLFSFEMLNNISQVREILK 68
HvirOr8         KFLAIWPGK--------DF--TRRYKYYTVAFLTAYFIIFMILFTINLFFLPKQLDIFIE 66
BmorOr8         KMFGIWADR--------KS--SKTYKYYSFVFLFITLIMYNSLLAINLLYTPLKIELLIR 68
AmelOr68        TICGCRMPSS----CRTSY--KRMLYILYATFVLLLLYSFCISQFLNVIINVRTADELCN 65
AmelOr69        TSCACWRPP-----FLSPL--KNFAYTVYYCYVILLIYGATFCQFVDLLLIVETEDEFCD 64
AmelOr70        SVCGCMPPTS----WKSSF--KKSLYNIYTCVIWLLILSLVSTQILDIIINVKNKNEFIE 65
AmelOr71        ALCGCFPPSS----WTTRL--KRYLYKIYAVFSFVALNSFLLSQILDMVYNVKGTDDFSD 65
AmelOr72        VATGYFLSPK----IKSPR--KRFLYNVYTVVVTLFLLSFLLTLIMQIVFNVRTADELSE 65
DmelOr19a       RIFRIMGIHPPGKRTFWGR--HYTAYSMVWNVTFHICIWVSFSVN---LLQSNSLETFCE 71
DmelOr19b       RIFRIMGIHPPGKRTFWGR--HYTAYSMVWNVTFHICIWVSFSVN---LLQSNSLETFCE 71
DmelOr33a       LYWRLLGVEG----DYPFR--RLVDFTITSFIT--ILFPVHLILG---MYKKPQI-QVFR 65
DmelOr33b       LYWHLLGLES----NFFLN--RLLDLVITIFVT--IWYPIHLILG---LFMERSLGDVCK 67
DmelOr22a       RVMWSFGW-TEPENKRWIL--PYKLWLAFVNIVMLILLPISISIEYLHRFKTFSAGEFLS 85
DmelOr22b       RVMWSFGW-TVPENKRWDL--HYKLWSTFVTLLIFILLPISVSVEYIQRFKTFSAGEFLS 85
DmelOr42b       RAMKFIGW-LPPKQGVLRY--VYLTWTLMTFVWCTTYLPLGFLGSYMTQIKSFSPGEFLT 82
DmelOr59b       RAMWLIGW-IPPKEGVLRY--VYLFWTCVPFAFGVFYLPVGFIISYVQEFKNFTPGEFLT 83
DmelOr59c       RIAFFLGW-TPPKGALLRW--IYSLWTLTTMWLGIVYLPLGLSLTYVKHFDRFTPTEFLT 85
DmelOr98a       YGMFCMGW-HTP--ATHKI--IYYITSCLIFAWCAVYLPIGIIISFKTDINTFTPNELLT 80
DmelOr42a       RCVFLMGV-RKPP-AKFFV--AYVLWSFALNFCSTFYQPIGFLTGYISHLSEFSPGEFLT 85
DmelOr85a       RSIDQMGWRLPPRTKPYWW--LYYIWTLVVIVLVFIFIPYGLIMTGIKEFKNFTTTDLFT 83
DmelOr43b       ETLRNSGL---NLKNDFGI--GRKIWRVFSFTYNMVILPVSFPINYVIHLAEFPPELLLQ 82
AgamOr2         RVWLFWSYLRRP------------RLSRFLVGCIPVAVLNVFQFLKLYSSWGDMSELII 60
AgamOr10        RVWRFWSFVLVH------------NWRRYIS-IIPVTALNVFMFADLYRAWGNIEEVII 59
DmelOr30a       KLMKFWSYLFVH------------NWRRYVA-MTPYIIINCTQYVDIYLSTESLDFIIR 64
DmelOr49b       KILRFWALLYDK------------NLRRYVC-IGLASFHIFTQIVYMMSTNEGLTGIIR 58
DmelOr43a       RMWRHLAVLYPTPG----------SSWRKFAFVLPVTAMNLMQFVYLLRMWGDLPAFIL 62
AgamOr32        RLIRFCGVVEQPSLKNN-------VRCLFAFILLCSFGPL--HVWYLAKTPVLDLVVTCE 63
AgamOr35        WYLRYCGVFREKSALGG-------IRLSVCLGVLVVFMVL--QSIYIFQH-VHHFAMICD 62
DmelOr47a       ALLGFDLFSENREMWKR-------PYRAMN-VFSIAAIFP--FILAAVLHNWKNVLLLAD 62
DmelOr98b       RLLGLELLHEQDVGHRY-------PWRSICCILSVASFMP--LTIAFGLQNVQNVEQLTD 65
DmelOr9a        RCMGIDLWSP-TMANDR-------PWLTFVTMGPLFLFMV--PMFLAAHEYITQVSLLSD 74
AgamOr38        LIFGCWPPDRLTRRWYV-------KVLIAVNLITLAICIVGEFRYCLYAYRNGSLIETIE 82
AgamOr39        LIFGVWPADRLVRPWYV-------KVLIAINLATLAICMVGEFLHGLYAYQEGDLSESIE 83
DmelOr24a       SLIGFYPEQ--KRTVLV-------KLWSFFNFFILTYGCYAEAYYGIHYIPIN-IATALD 63
DmelOr45b       GLLGLRFGK--EQSWLH-------LLWLVFNFVNLAHCCQAEFVFGWSHLRTS-PVDAMD 74
AmelOr74        KSAGIWVIGNDAEERQ---------RKFAVFYTLAALIYGIYVNAVDIYHNLDNLAHCVF 69
AmelOr86        KLTGVWMTVNDGEKRR---------RRIAMAYTFVIQVYGLYLNIGDIYHSWDDLSHCIF 69
AmelOr75        KIVGLWLATDRNEQRQ---------RDFALIYTVGTLFISICIAFRDIYYSWGNFSNSVF 69
AmelOr84        KIVGLWLAADRDEQRR---------RDFALIYTVGALFIIVCIGFRDIYFTWGNFSDSVY 67
AmelOr87        KIVGLWLAKNEQEQRK---------RRLTLMYTVIAILFGVWVQFRDFYYSWPNFGNCAY 84
AmelOr76        KIAGFWTSTNYVEERR---------RNVTMSYTLFAILFAATTEARDLYFSWGNFSDSIY 69
AmelOr85        KIVGFWIASNYVEERR---------RNLTISYTFFAIFFAMATEARDLYFSWGNFGDSIL 70
AmelOr88        KVAGFWVANNYAEKRR---------RNVAMFVTIFFAFMGISIEGRDLYFAWGDFEDSIF 69
AmelOr91        KLVGLWMAADQYEQRL---------RNISVTYNLVAILFALYLQTTDIYYSWGNFSACLF 68
AmelOr92        KFVGIWMX--QYQQRM---------RNIMVAYNVIAIFFALWIQTMDMYHSWGNIRACLF 66
AmelOr95        KLMGIWMANDQSEKYV---------RNVTILYSIIALLFGLWLQITDMYYSWGDFSECIF 66
AmelOr93        KLVGIWMAGNGZEX-------------ITLFTAIIFSGYNLZ-----MYFILGDFSACLF 55
AmelOr77        KLVGLWTAKNRREQRA---------RKFALIYTVAAMLFALWIEFTDFYYSFGDFSTCLF 70
AmelOr94        RVVGFWLASSRLEEWF---------GNATVMYSIITIIFSMWVQMRGLYFSWGDFGVCTF 69
AmelOr96        RVVGFWLTTSRLEEWF---------RIGVVGYTILAITFSAWVQIRGLYFNWGDFSACTY 69
AmelOr79        KNVGVWMSHDPGEQRR---------MRMLLVCTVWMLLLGIVINTRDLYFTMLYNGDILY 69
AmelOr83        KNVGVWMPDNSDEQRR---------MKMLFLYTIWMLFCGTIISTRDLYFTLLYNGDILY 69
```

```
AmelOr80        KNVGIWMSDNPNEQRR---------IKMLFLYTIWNLLFGTVVNSRDLYFTLLYDGDILY 69
AmelOr81        KNVGVWISDNPSEQRW---------RNMLLGYTTWILLSGIIINGRDLYFTLLYNGDILY 69
AmelOr82        KNVGVWMSDNPNEQRQ---------IKMLLINTTWILLSGIVINGRDLYFTLLYHGDILY 69
AmelOr78        RNIGLWMSDDPGEQRR---------MRILLVYTVWILLLGMIINGRDLYFTFLYNGDILY 69
AmelOr89        KNVGLWASNDPGHERR---------RKVILVYTMWCVTLSSVVIIRDVYFTWFYNGDILY 69
AmelOr90        RSIGLWIGDGSTNERR---------RKGMLAYTIWCTFFSTIISSRDLYFTWIYNGDILY 67
AmelOr142       ALTCSWPPSPLATKAQ---------HLFFNALWCIAFLTSVMLFLPLLAAIYVYRKHPVI 69
AmelOr146       SLSCCWPLPSTATKLQ---------TRCFKIIRSLLFLNSLLLFFPLLYFVYVNRNDNTT 69
AmelOr148       ALSLCWPLPASSTRKQ---------IVCIKILQIGAIISAFMVLLPLIYAIHLNIHNLIN 69
AmelOr158       ALSFCWPLHINSTRKQ---------IMYIKILQISAVVNAFMVLLPLIYTIHLNMHNLIN 69
AmelOr147       ALSLCWPLPINSTRKQ---------IVCMKILQIGAIISAFMILLPLIYTIYLNLDNLNI 69
AmelOr150       AITFCWPLPANSTKIQ---------VFMFKTLQIISIINAFILLLPLLYSVYLHFDDIVI 69
AmelOr151       ALTLCWPLPANNGKIQ---------VFMFKALQIISIINAFILLLPLLYSVYLHFDDVII 69
AmelOr152       AITFCWPISLNSSKTQ---------VFIFKILQIISIINVFMLLLPLLYSVYLHFNDIII 69
AmelOr154       AICCCWPRPFNSTKNQ---------IFAFKVLQISTIISAFMVFLPLLYSIYLHNDNIIH 69
AmelOr155       AICFCWPYPLNSSRNQ---------IFGFKVLQISTMVSACIMLLPLLYSIYLHNDDVIH 69
AmelOr157       AMTFCWPLPPDTARKR---------IVGMKVLLIISIVNGCAVILPMLYWIHLHLDDIIS 68
AmelOr156       ALCFCWPVSANTSRNQ---------IIVFRFFQIFTIISSCLGSLPMFHSIYLHQDDIVI 69
AmelOr153       AL--CWPLSVNSGKTQ---------VFIFKMLQIISIVSACMLLLLSSYSIYFX-HGQCR 66
AmelOr144       TFTCTWPINPKDSKKY---------IIIRNILWTFTILNVIFLTISLMLAIFHFRSNIPK 70
AmelOr145       TFTCAWPINPNDSKTY---------IIIRNILWIFTILNVIFLAISMIFAIFHFRSDIPK 70
AmelOr143       TFTCTWPINPNISKRR---------IIFRNIFWIFSILNVILLMTSLMLAVVYFRNDILM 65
AmelOr1         TLIGIWPRRNTFID-------NVKFYVQIGIVFFLMCFLLLPHVIYTYFDCE-NLTKYMK 79
AmelOr3         TMVGIWPRRNTFSN-------NVKFYVQTTIVFFLMCFLLLPHVIYTYFDCE-NLTKYMK 84
AmelOr6         KIIGAWPILIESSL-SS----KIQKWFIISFSISLQMCIVVPCILVMFLKEK-NGRRKIN 72
AmelOr7         KIIGAWPIVIESSL-SS----KIRKWFIISFSISLQMCIVVPCILVMFLKEK-NGRRKIN 72
AmelOr4         KIIGAWPIAIESSL-GS----KIQKWFIISFYLFLQICIVAPCILDVFLKEK-NGSRRIN 74
AmelOr5         KIVGVWPIPIGSPL-SS----KIRNWFITFFSLFLQICIVGPCILVMFLKEK-NGKRKIN 74
AmelOr8         RMLGYWPLNVPSSA-FS----KILNSFTIFFSYLLPLIVLIPGLLYVFLKER-NGRRKVK 76
AmelOr9         KMVGCWPLNIPSST-FS----KIFNAFIIFISYLLSLIVLVPGLLYLFLKEK-NGRRKIK 76
AmelOr11        RTLGYWPL-VSPSA-FT----KFFNSFTIFTLYFLELIVLIPGLLYV-LQVK-NPRTKIK 74
AmelOr12        RMLGYWPLSVSSSA-FA----KISNYFIIFLSYLLTLIFMVPGLLYIFLKVK-NGRSRIK 76
AmelOr10        RMLGHWPLSVSSSA-FS----KILNSFIIFISYLLQMIVVIPSLLYVILKEK-NPKKKIK 76
AmelOr14        RILGAWPNRVSSST-LS----KIFNWYLIFTCYTLQLIVLVPGFLHVFLKEK-NGRKKMK 76
AmelOr15        GKLGAWPNRATSSTSFS----RTRNCILIFMCYSVQLIILIPGLLHFFLKEK-DSRKKVK 77
AmelOr13        KILGAWP-KSSPSSVLS----TILKCCLISICYLIQLMVLIPGILYIFLKEA-NLGGKIK 68
AmelOr16        ATIGAWPISSSTSF-FL----KALQRLGHIFTYFLFFLIMIPTLAYVFLKEK-NSKVRLK 79
AmelOr17        GMIGAWPISPSTSF-LL----KVLQRLRHIFTYFLFFLIMIPTLMYVFLKEK-NNKVRLK 79
AmelOr18        RTIGTWPRSPDHSWLE-----TLEHVCLNLFCYELLAFILIPCSIYIILEIK-DFYNQLK 86
AmelOr19        RTIGTWPKSLDRSWLE-----TIEHVCLCFLNYVLLAFILIPGVMYFLLEMK-DFYDQMK 86
AmelOr22        KPIGLWPNSSTISTTG-----KYLYRLINVICYSLISFLSIPCSLYVILEVE-DIYNRIK 87
AmelOr24        KPIGLWPNSSTISTTG-----KYLYRLINVICYSLISFLSIPCSLYVILEVE-DIYNRIK 79
AmelOr20        KPMGLWPNS---YTSK-----DYPYWLINIVCYCLISFLFIPCTLYLFLEIE-DFYGKLK 85
AmelOr25        TPIGAWPNLRK-SRIG-----KCYSLLISIICYGLIGFMLVSCSMFLMVEIK-KVYNRIK 80
AmelOr29        TPIGAWPNLRK-SRIG-----KCYSLLISIICYSLIGFMLVSCSIFLMVEIN-NIYNKLK 81
AmelOr30        KSLGTWPNLRE-SRIG-----KCYSVLIGIVCYGLISFMLTSSNMFLVVEVK-DTYNRIK 81
AmelOr21        KMIGLWPIFDKISTIH-----KFLRMLYNTICYCLLMFMIVLGWMYIAFEVK-NIYDGLK 79
AmelOr23        KMIGLWPIFDKSSTIH-----KYLQWLYNVICYSLIMFIIISGWIYISLEVE-NIYDRLK 79
AmelOr35        KAIGIWPLSLCVTTTE-----KIHSVILTLISIFLIGFLLVPCTLCTLLDKTGDLDTKIK 82
AmelOr27        KPIGAWPLFSTTTKFE-----KTVSLILNIICYAIVILCATPSLMQIILAEE-SFYLKLK 80
AmelOr28        KPIGAWPSFPSTTKHE-----RIISFLLNVSCYSSLLFTLIPCLLHMLLEDE-SFYLKMK 81
AmelOr33        KSIGTWPLSPSTTKLE-----KTISFLLIICCYCFICFTVIPCLLHIILGDD-SFREKLK 82
AmelOr34        KSIGAWPSFSSTTKLE-----KIISFVLIICCYCFICFTVIPCLLHVILEDD-SFHEKLK 82
AmelOr32        KPIGAWPPSPSTTKLE-----KIISIVLIICCYSSICFTVIPCLLHVMLEDE-SFRDKLK 81
AmelOr31        KPIGAWPSSSSSSKLE-----RIVSFFLIVLCYGFILFTVIPSLFHIVLEDE-NLHMKLK 81
AmelOr36        KTIGVWPLPSSTSKFE-----KITTRILILFCWTIAVLDTTSGLLHFVLVKE-DIIIKLK 81
AmelOr37        KTIGVWPLPSSTSKFE-----KIMTRILILFCWIIALFDAISGLLHFVLVKE-DIIIKLK 82
AmelOr38        KTIGVWPLPSSTSKFE-----KIVTRILIIVCSIITLFVIIPSMLHFILVKE-DIISKLK 82
AmelOr39        KTIGVWPLPSSTSKLE-----KIMTRILILFCWITTLFVTISSLLHFTLVKE-DIIIKLK 82
AmelOr47        KTIGVWPLPSSSSKLE-----KIITKILIFLYWIIVLFIIITSLLHFILVKE-DIVSKLK 81
AmelOr48        KMIGVWPLPSFTSKFE-----KIMTKILIFFYWIILLFIILASSLHFLFVKE-DIVSKLK 81
AmelOr40        KTIGVWPLPSSTTKFE-----KIMTKILIFLCWIIALFVITSSLLHFTLVKE-DIISKLK 81
AmelOr43        KTIGVWPLPSSTSKLE-----KIMTKILIFLCWIIALFVIISSLLYFALVKE-DIISKLK 82
AmelOr45        KTIGVWPLSSSSSKFE-----KIMTKILIFLCCIIALFVIIPSLLHFTLVKE-DIISKLK 81
AmelOr41        KTIGVWPFTSTTSKFE-----KIMTKILIIVCSIIALFVTVPSMLHFILVKE-DIITKLK 81
AmelOr42        KTIGVWPFTSTTSKFE-----KIMTKILIIVCSIIALFVTIPSMLHFILVKE-DIITKLK 81
AmelOr44        KTIGLWPLTSTTSKFE-----KIMTKILILLCWIIALFVTTLSLLHFILVKE-DIITKLK 81
AmelOr46        KPIGAWPISLCTTRNE-----KIISKILIIVCWSLSLFTLIPGLLHFILEKE-DTYLKLK 81
AmelOr49        KPIGAWPASFYKSRIE-----KIVSKILIVICWISSLFTLIPGVLHFFLEKE-DIYVKLK 81
AmelOr50        KPLGAWPASSSTTKME-----RIISQILIVICWCIILFTVIPGILYILFVKQ-DIYVKLK 81
```

```
AmelOr26        KPIGAWPNLTKATRTE-----KLLVKLLNFICHSLIIFTVMPCIMYIFYEDE-SLKTRMK 85
AmelOr53        KPIGVWPLIYNQTSRFE----QLISIILMGTCFSSLLFIILPSGHHILFVEK-NLHMKVK 88
AmelOr54        KPIGVWPLISNQTNKFE----QLVSIILMITCFSSLLFIILPSGHHYFFVEK-NLNMKVK 88
AmelOr52        KPIGVWPFVYDRTSRFE----QLISIILMATCFSSLLFIILPSGHHIFFVEK-DMHLKVK 86
AmelOr51        KPLGIYYFIYNQANKFE----KILSIILILICFFIIQFVIVPFGYYILFYEK-DMNTKIK 86
AmelOr55        ISIGIWPTVLKNIG-------KFLPKIVIGINNLMCFFILIQSALHIILEQK-DTLLRLK 84
AmelOr57        ASIGIWPTVLKNIG-------KILPKIVIGFNNLLCFFTLTQSALHIILEQK-DTLLRLK 84
AmelOr56        TCIGIWPIILKNIN-------KILPKIVIGINNLLCSFILIQSALHIIYEEK-DVLLRLK 84
AmelOr58        NSIGIWPTVLKGID-------EYLPKIAIALSNLVLSFTVIQCVLHILLEQK-DPILRLK 84
AmelOr59        KSIGIWPSIFKDVS-------KFLPKIMFGLCNFVLFFAIIPCILYIVIEEN-DTMIRFK 85
AmelOr60        KSIGIWPIILKDVA-------KFLPKIVIGISNFVLLFAIIPCILYIILEEK-NNLIKLK 85
AmelOr61        KSIGIWPSVLKSVS-------RFLPKIMFGFNNFVLLFSVIPCILYIVYEEK-NIMIKFK 85
AmelOr63        WPVGTWPLQVF----------NTFSIIRATFSTFLLLLMLTILQVELYLDSS-NPEYNLD 73
AmelOr64        WPLGTWPLQVF----------NTFSIIRAMFSTFLVLLMLAILQVELYLDRS-NAENNLD 69
AmelOr66        WPVGTWPFQVY----------DIFSLTRTIFSISLLLLMIAIVQVELYLDRT-DAENNLD 69
AmelOr65        WPVGTWPFQVY----------DTFSLTRTIFSISLLLLMIIIVQVELYLDRT-NAENNLD 69
AmelOr67        WPVGTWPFQVH----------EIFSISRTIFSISLLLLMVVILQVELYLDRS-NAENNLD 69
AmelOr114       KFIGIWPEERKWNQAS------NYLVLIPFLMILCFICAPQTINLTIISNDFNLVIENLS 55
AmelOr115       TLLGVYPEPRKMSRNSRLM--SSLIFWFTTLVTFTFICAPQTANLILKSTSLDEVIENLS 70
AmelOr118       RIFGIWPDSPYPKLKI---------ITWIIILPTFLVFQYWYCITHIKLG-LIDLLDGLS 60
AmelOr164       RLIGAWPNSSCQILKY---------IMWTIVMSIFLIFQYSYCIIHIKTATLIDILDCLS 66
AmelOr163       RLIGVWPYSSYRIMQR---------IFWTIIMGNSTVFQLWYCISYFKTADLFDLLDGIT 65
AmelOr165       HFAGIWPGTPFPYLHK---------LGWLAAIAALQSYQYRYIVMHYKSDNLMSIIDNLS 66
AmelOr166       YFAASWPGASFSILHK---------FFWTIIFCTLHISQYSYLIMHYKYDALTEIIDNIS 66
AmelOr167       RLIGIWPKSSYEIIVR---------FMWVIIMMCAQIFQYQYIINHIGFDNLADLIDSVS 66
DmelOr35a       LNHIFWPLDPSTGKWGR----YLDKVLAVAMSLVFMQHNDAELRYLRFEASNRNLDAFLT 80
DmelOr74a       LNHVAWPLEAESGRWTV----FLDRLMIFLGFLVFCEHNEVDFHYL--IANRQDMDNMLT 81
AgamOr3         TIAGLWGDRSQRY--------------RFYLI-FSYFCAMVVLPKVLFGYPD--LEVAVR 83
AgamOr5         RFVGLWGERRYRY--------------KFRLA-FLSFCLLVVIPKVAFGYPD--LETMVR 63
AgamOr13        QLFGVTGSPEERF--------------RILPV-MLTYFFFIVVPKCFFGYPD--LEITII 63
AgamOr15        RVFGVTNHSEERF--------------RIVPV-MLAYFFFIVVPKCFFGYPD--LEIMII 63
AgamOr17        RVFGVTGGPDVRF--------------RILPV-MLTYVFCIVVPKCFYGYPN--QEIAII 63
AgamOr16        RLVGVSQTQSERY--------------RYVPM-FVLFLLTIAIPKIAFGYPN--FETSII 63
AgamOr55        HAVGIANGETFSH--------------RFTAV-YLLFIFIIAIPKLFCGYTS--FEASVV 64
AgamOr18        RVIGVWDVPQYRY--------------RYAAV-FLSYCFGILIPKLCFGYPT--LEASIR 63
AgamOr14        ERLGFWGDRTTRV--------------RYPVA-FVAFWITVAIPKLATDYSD--VELFIC 63
AgamOr51        ERLGLWG-ESYRA--------------RFLFV-VVAFSITVCIPKFTTTYTN--LETFIC 62
AgamOr12        QLSGFRQDTEQLG--------------TRIYLNLFIFVAAILIPKVCFPYPD--TEAMVR 64
AgamOr19        KLSGLTQETTRFG--------------AINFLNLFIYIAAVLVPKVCFPYPN--TEAMIR 64
AgamOr20        KLSGLTQETTRFG--------------AINFLNLFIYIAAVLVPKVCFPYPN--TEAMIR 64
AgamOr21        RLSGFRQETEQLE--------------KQIFFNLFVYVAALLIPKVCSPYPD--SEAIIR 64
AgamOr50        RLSGFSPGTEQLG--------------RCIYVNLSIYITVILVPKVCFPYPN--TEAMIR 64
AgamOr29        KLFGLGRDEKLKL--------------LYWVQ-CVFYLVFSIIPRVLVQIDD--TIMVLR 63
AgamOr53        KLFGLGRDERFKL--------------VYWLQ-CVAYLAFSIVPRLLVEIED--MVALMR 63
AgamOr30        MLCGCPRSARVNR--------------RFWLL-GVFFFVFGQIPRFLIKIDE--PIALVR 63
AgamOr46        ALLGVNKNPSERF--------------RLYAI-YIYLWVAMFIPKLCLGYET--IPQCFR 64
AgamOr47        ALLGVNKNPSERF--------------RLYAI-YAYLCIALFIPKLCLGYET--ILQCFR 64
AgamOr48        AMLGLWGDRRKLY--------------RLYAL-LLLAYVAIIFPKPVRITDRHPFESIVR 68
AgamOr49        EHSGLWGDPRRKR--------------SFALM-LLGTVLLLIVPKIVLGTGSDSFDSIAR 78
AgamOr61        TISAIHYDAS---CWFD----------RAIWIVYRSLPILVNISYFYKAYRLILFPEDNT 81
AgamOr62        TLFALHYHTRQPRWTVK----------KLLWTVYRTLYLLSYLSYCYKVYWLFSHWQYST 86
AgamOr63        KIYAIHYTPHG---WHD----------RILWYLYRALYGLIYLSYIHKTHWVLHHPQDSF 82
AgamOr60        FLFAIRNDAPRG--VGQ----------FMRWCFHLMAPVFVLSSYCYKAYWYMSHSEYSF 81
AgamOr75        VVSPAMLLQRP-----R----------SNLEVALKTLCLSVMVAHTIALAYDFSQQTDVR 80
AgamOr76        VVSPAMLLQRP-----R----------SNLEVALKTLCLSVMVAHTIALAYDFSQQMDVR 80
AgamOr78        VVSPAMLLQRP-----R----------SNLEVALKTLCLSVMVAHSIALAYDFSQQTDVR 80
AgamOr77        TINGIHLHDDSR---LA----------RAFEYTFYALQLVQLLNGYNFAVLCSNDTISLE 67
AgamOr79        TINGIHLHDDSR---LA----------RAFEYTFYALQLVQLLNGYNFAVLCSNDTISLE 67
AmelOr99        RFLGQDPRQKDEFRNIIV----------FILIISIASIVIPTTLELYISLRNKDVDGVIE 77
AmelOr101       RFLGQDPRQKNEFRNIIV----------FILVISIASILIPTTLELYISLRNKDMDGVIE 77
AmelOr102       QLLGQDPRLKNEFRNFIV----------TVVVISISGNIVPTSIELYTSLCDKNMDAVIE 77
AmelOr103       QLLGQDPRLKNEFRNFIV----------TVVVISISGNIVPTSIELYTSLCDKNMDAVIE 77
AmelOr104       KFLGQDPRQRDGFRNIIV----------IVMVASISGILIPTSLELYTSLRDKNMDAVIE 77
AmelOr98        QFLGQDPYQECKYRNIIT----------IIMLISMIAIFIPTTFEIYVSIHDKNTDAVME 77
AmelOr100       ---------------------------------------------------------MFE 3
AmelOr97        ------------------------------MICSLISFLIPGLSRVYISIVEKNLNALME 30
AmelOr105       TICGINPYQNNSISIIII----------IMIISVCMSFLCPTSIQLWEAISNKDFDNIIQ 77
AmelOr109       QLVGVWPYQKKFFKTCIR----------FITFTIMIFSLATQISRVIVFYS---LDVLSD 65
AmelOr110       QLVGVWPFQERFIKTCMR----------FIVSVIMLLDLATQISRVIVFYS---FDVFSD 65
AmelOr111       QLVGVWPYQQRFIKFCIR----------FITSVIVVLTLAAQISRVIIFYS---IDVLSD 65
```

```
AmelOr108       QLVGVWPYQERFTKFCIR----------LTIFAIIILTLTTQIYQVIVFCT---LDALSN 65
AmelOr107       QLVGVWPDQGKFMKFLMR----------FIILIVMIIAFIAQISRVAVFYS---VDVLSD 65
AmelOr112       LITGVWPYQNYRSKMAER----------FISVTVMMSGFVTQFAYLVLNPT---MDKIAT 65
AmelOr113       KLSGIWPDQNKYLKYISW----------IIIYVISIPSIVVQIARIVHIST---ANVIVE 65
AmelOr106       IIVGMWPNQKR--KTIPR----------IFVELIAILAHLTQGGNMVLFFS---LTLAMD 62
AmelOr122       KIVGLWPYDKSIYVWIQR----------ICLSMYFLIGVIFQIILLVKSEI--TLRNYIV 65
AmelOr125       KIVGLWPYDKSIYVWIQR----------ICLSMYFLIGVIFQIIVLVKSEI--TLRNYIV 65
AmelOr123       KIVGLWPYDNSIYVRIQR----------ICVLIYFLIGILIQIFSLVKSEI--SLRNCIV 63
AmelOr126       KIVGLWPYDNSIYVRIQR----------ICVLIYFLIGVLVQIFSFVKSEI--SLRNCIV 63
AmelOr127       KIVGLWPYDNSIYVRIQR----------ICVLIYFLIGILIQIFSFVKSEI--SLRNCIV 63
AmelOr128       KIVGLWPYDNSIYVRIQR----------ICVLIYFLIVVLVQIFSLVKSEI--SLRNCIV 63
AmelOr129       KIIGLWPYDNSIYVWIYR----------LCLLIYFLVVVLVQIFSLAKSEI--SLRNCIV 63
AmelOr133       ------------------------------------------------------------
AmelOr134       KIIGLWPYNNSIYVWIQR----------LWISALFLGNIIFQIVLLLRSKI--TVRNCIL 63
AmelOr131       KIIGLWPYNNSVYVWIQR----------LCISALFLGNIIFQILSLIRSEI--TLRNCIL 63
AmelOr132       KIIGLWPYNNSIYVWIQR----------LLLLTLFLGNIIFQIMSLLRSEI--TLRNCIL 63
AmelOr135       KIVGLWPYNNSIYIWIQR----------LLLLTFFLGNVIFQIVSLLKSEI--TLRNCIL 63
AmelOr136       KIVGLWPYNNSIYVWIQR----------LWFLMFFFGNIIFQIMSLLTSAI--TLQNCVL 63
AmelOr138       ------------------------------------------------------------
AmelOr130       KIIGLWPYDNSIYVWIQR----------LCLLSYFFANIIFQIVSLLRSEI--TLQNSIL 63
AmelOr137       KIVGLWPYNNSIYVWIQR----------LWLLIFFLGNIIFQVVSLS-SEI--TLRNCIL 62
AmelOr139       KIIGLWPYNNSIYVYIQK----------LZLLIFFLGQIIFQX----------------- 50
BmorOr20        RLLGAWPGHYLGEETGS-----KYECAPMFLLMFIKIACLYLTIVYLRNNADVLGFFELG 82
BmorOr3         RLLGAWPGHYLGEETGS-----KYECAPMFLLMFIKIACLYLTIVYLRNNADVLGFFELG 82
BmorOr18        RLLGAWPGHYLGEETGS-----KYECAPMFLLMFIKIACLYLTIVYLRNNADVLGFFELG 82
HvirOr6         DFIRTWPRKELGEPENL-----AFTVFMQYFYLILNIVTVMGSTSYIVVRGSELSFIEAG 87
BmorOr23        KIMDFWP--EREEKNSK-----TRIFRLRYILVLQFCFTLVAGVLYLTNSVGKQTFYDLG 83
BmorOr16        KIMDFWP--EREEKNSK-----TRIFRLRYILVLQFCFTLVAGVLYLTNSVGKQTFYDLG 83
BmorOr4         KIMDFWP--EREEKNSK-----TRIFRLRYILVLQFCFTLVAGVLYLKNSVGKQTFYDLG 83
BmorOr5         KIMDFWP--EREEKNSK-----TRIFRLRYILVLQFCFTLVAGVLYLKNNFGKKTFYDLG 83
BmorOr22        KYIDGWPNMDMNKKNVS-----KIRFHKRHLLVVEQTITFLSQMFYIVKNYGKLSFFEIG 89
BmorOr17        KYIDGWPNMDMNKKNVS-----KIRFHKRHLLVVEQTITFLSQMFYIVKNYGKLSFFEIG 89
BmorOr1         KIICAWPEKQLNEIRSL-----GHSIH-RVILPIQSVVCLACGILYIHFHFNEIPFFILA 84
BmorOr1a        KIICAWPEKQLNEIRSL-----GHSIH-RVILPIQSVVCLACGILYIHFHFNEIPFFILA 84
BmorOr1b        KIICAWPEKQLNEIRSL-----GHSIH-RVILPIQSVVCLACGILYIHFHFNEIPFFILA 84
BmorOr19        DLT--WWGYTFPKYVGW-----FYHLQCNVVRLFGKCVVVSQILFIILNYQTIDKSVFII 82
BmorOr15        DLT--WWGYTFPKYVGW-----FYHLQCNVVRLFGKCVVVSQILFIILNYQTIDKSVFII 82
TcasOr5         FGLPAYKIIKTPNFKSLTH--NNSKLNYIMVIISTTAYLYYFKVGDKNTNSFEKHMTMYR 79
TcasOr7         KELG--KIHKDLNKLAMEV--NET-FGELMLLRFSGDFIYLIVGLSSNAVLNAEYETVLD 75
HvirOr4         VICTLFGLVAEINVGVELS--VRMSSRMSQVVSTCDVLVVVATAGAGVYGAPRRMRNMLK 70
BmorOr13        NVCTLIAVMIDFSVPVKLS--IRMQTETKRFVWIADVVIMGILSGVGVYTAPIQMRRLIA 118
DmelOr10a       DIMGYWPGKTGDTWPWR----------SLIHFAILAIGVATELHAGMCFLDRQQITLALE 76
DmelOr33c       ICMRLLVPTFFKDSSRP--------VQLYVVLLHILVTLWFPLHLLLHLLLLPSTAEFFK 66
DmelOr7a        ALGMQAPDGSRPTTSSTWQ--RIYACFSVVMYVWQLLLVPTFFVISYRYMGGMEITQVLT 86
DmelOr22c       LIVGLWPQRIRGGGGRP------WHAHLLFVFAFAMVVVGAVGEVSYGCVHLDNLVVALE 75
AgamOr1         KYLGLWPPEDTDQATRN-------RYIAYGWALRIMFLHLYALTQALYFKDVKDINDIAN 79
AgamOr4         HIVGMNGAGFRSRIRVGG---------IFLFYLIFLVIPPLTGGYTDGHQRVRTSVEFLF 74
DmelOr47b       FYYVRAFLSLLCQYPNKKL--ASLPLYRWINLFIMCNVMTIFWTMFVALPESKNVIEMGD 94
AmelOr73        TYGGYWRPTKWPASSYK------YHLYNIYSAFMIFLLYFITFCTCVDSLISKNLKTMSE 65
AgamOr68        RLIGAGLWEKQEGILWG------------RVASVMQIVLFLTLHAWTGYKYRYDALQMLE 67
DmelOr59a       TAWRYLGVAHFRVENWKN------LYVFYSIVSNLLVTLCYPVHLGISLFRNRTITEDIL 70
DmelOr23a       RICGALDLSEGR--------------YWSWSMLLCILVYLPTPMLLRGVYSFEDPVENNF 65
AgamOr31        RAIGIARTDSFRG-------------RVLFAVSFFTVLVMMLGTVMFAFKHIDQIMLLCD 64
DmelOr2a        RVWELTGLMRPPGVSS-------LLYVVYSITVNLVVTVLFPLSLLARLLFTTNMAGLCE 72
AmelOr62        KIIGIWPTNGERSFFG------------RWIFAVTTQIGIIYILSLEIYRHCLDIDDTMD 70
AmelOr121       NVICLWPVEEQNMRKQS-------WTKLHIMTCFMLITFVCTIPCLCALKQCNNLMEVTD 77
AmelOr140       PIISIFQSASMYFNGIFN---------TKKIKNILLFIKNDHKYYINRPENIILQKYDLQ 71
TcasOr12        KCIQFFGYFSPDFRNNSQR--KLLFRIYAALFVGFAFILSLLSQIANMVDAFGDIEKMTE 75
AgamOr8         AMIGVARYGEPKRTLRAY---LKHLLFWSSCINTGFCLVIEHIYFVKAAGNFTNFLQLTA 79
BmorOr9         WLMGIPFDNPKIQIR------------YYVLLLPLSLMLIEEIAFFGSRMSSENFLELTQ 65
AgamOr40        RLMAVWPTDFNPYLPQYLR--GRFFLSELFDFGYQLFWYFICVHIAAFHIMSVLTGDLSY 81
TcasOr63        LVVGFYPKRDNKHEILY---------WLSAFFNLLISYGQLTTMIIQMVFDRSDLSKLTE 72
TcasOr4         RYGSFYPCGKRIK------------FIFLGLFMFVYSWTEFLSMITVLFVERDNLTKLSE 65
TcasOr13        RIFCLQPLKWIKTDYSYVISKSGPYMAYSILTSLFLITASIYGLTQVYSMEAVYLIRLSS 84
AgamOr24        KSMGVSFFKRKHSFVN-----------FFFIFGISNLVLYVVSVLISSYKSRHDIIKLIY 60
AgamOr54        RVGGMLGIDIFTPGYSSTN--------LLLRMVLLNTFTFFWINLYSLTTTYGNLVDFMY 86
AgamOr23        DLPGDGSHSVSVRKPLLDR-------DRLGIADGCCVLLCYAGNRYTGELMKFYFEHSIT 70
AgamOr64        RSLQLFGNPVRCLQEFGTVR---GLFVQLCRFLFLLPYASFALKSYWQLNHPLDTNNSIL 92
DmelOr82a       RAMGHKDDMDSTDSTALS---------LKHISSLIFVISAQYPLISYVAYNRNDMEKVTA 63
AmelOr116       YYIGLWPDRVKYKYLYN---------LYTICSLIFLVGIIIVSEIIYIIINWGKIEIMMT 78
```

```
AmelOr160       GLSGLWPDNRND---------------VRFFLYITYVVIFTWLEIVTLVQNIHDLEKTLK 70
TcasOr10        KSERFWKLDRFGDLQAKN-----------LHSTYPIFQIFFYVYVVILFLTCAMFALVNW 65
TcasOr26        KRMYLWPTASVTSR--------------KPAFFLITFSCFLLYGSVMHLIVNDISMEEVH 55
AmelOr117       SIAGMWPIDEKSSIFSK------IFAYVRLIFGLIIVNSFFIPQIIIIVMNWKNIKIIAG 74
AmelOr120       KFVGFYPIN-----------------ILRYVICISCIMFIVIPQIIMIYINWNDLNIVME 64
BmorOr7         RCCGFCRLSRSSTARRGLS----VAHEVYRALTLTLTVVYLLQECIYAYQERTDMDKLSR 71
AgamOr6         LMMGIPPCEEPYPPGVLPS-LKRNAGFIASFLLLAYTTIGELIYLKQMFERDVTFLEVTF 76
DmelOr45a       EIVGFDPSTPQLSLKHP----------IWAGILILSLISHNWPMVVYALQDLSDLTRLTD 63
DmelOr67d       FCVGFCGNDVADPNFR------MWWLTYAVMAAIAFFFACTGYTIYVGVVINGDLTIILQ 75
DmelOr83c       SLTNLLGVDFLSPKLKFN---------YRTWTTIFAIANYTGFTVFTILNNGGDWRVGLK 70
AmelOr162       NEYEILCEHYAIARKITTS-------FVAFLLGLTTPFGAMPLLLNIGDALGLCNISDDR 72
HvirOr3         -----------------------------------------MSGDKVTEERKKLMANYVM 19
TcasOr20        TYLNMLPEKTTFCTTIQQY--------YVSVIITITTFPILADLVSQFYEESISFTSVNE 66
AgamOr11        RYYGILLGQSQSYKKVH---------CFRGMVFTLSMVLFNCTQYIDLWQVWGSVSDMTA 77
DmelOr56a       QWYGYVASKDQNRPLLS---------LIRCTILTASIWLSCALMLARVFRGYENLNDGAT 77
AgamOr28        DLYVYWFLTFIPIASLCVP--QIIFRVKMLKQTKQIIHFYLSLQFTYLVVDTKSLIDFIS 90
AgamOr58        RFIGFFAWDVKLNATWLK---------MTLIVFAVGYEITAIAAMALASVKGVFTERSFT 79
AgamOr59        YLRYLRPFQLIAGYPINPRPALSKLATVVRLLAYLTYLGTLLHKISYVLYRPEDINYVSF 86
DmelOr1a        ARMGLDLQPDKKGNVLRSP--------LLYCIMCLTTSFELCTVCAFMVQNRNQIVLCSE 71
DmelOr13a       KLNGSWPLTESSRPWRSQS-LLATAYIVWAWYVIASVGITISYQTAFLLNNLSDIIITTE 78
AmelOr161       KIISIWPLAENSSRITITFR------RFHLFCMFFLVIVMSVAVTADVVHNIDDLDEATE 74
AmelOr141       KLCGIVPCGDGFARN------------ILAWLAFSCLTIYSISYVHEFITNTTNLTTALE 69
AgamOr52        WLRALF---------------------LLYQLLLPTQAIIWLWRTWAVAYIEHNKPLAIS 55
AgamOr36        ALPGFHLVEEFRTSSWKR-----ALFLLSRTVQLLQYALWADRFYLALVDPSSPPGKALH 86
AgamOr25        KWLGFDVIDPNWKVTP------------LTMFTIVMFALQHYTTYLYLSTHLDMFDMFTE 64
AgamOr45        RISSHMAVLKLNIIDPAWR------PTLRFGIVLFLTALVPVYIWQGIKVYRTRFETLLE 71
TcasOr3         KLTAFWIYDDETTRRKK-----YLQHAYNIFWIFYLFVAYQPAELLYVYYSFNDLSVFLR 76
DmelOr88a       QMVHFQWRRNPVDNSMVN------ASMVPFCLSAFLNVLFFGCNGWDIIGHFWLGHPANQ 79
AgamOr22        YEVFHWFLKISLLRIFDDDFLVAPVPTTLFQFHETELVVSLGVILLHAYCYRNDLDTIIL 78
DmelOr49a       KTLGYDLFHTPKPWWRYLL---VRGYFVLCTISNFYEASMVTTRIIEWESLAGSPSKIMR 75
DmelOr85f       WIMGYDMLGVPKTRSRR-----ILYWIYRFLCLASHGVCVGVMVFRMVEAKTIDNVSLIM 74
AmelOr119       VVVGGQMAHASGHEWMKSIRTLTSIKINYLKYSGLGEIDSSCSRILKYAYFVYKVWMLVS 86
DmelOr63a       YTVGFNLLDPSRCGQVLRIWTIVLSVSSLASLYGHWQMLARYIHDIPRIGETAGTALQFL 85
DmelOr67b       VEYSAYALGVNIAPRKRSS---KYCRLTRILVLIVNLSIIYSLVAFIMENYMISFETYVE 79
BmorOr21        IAAMIYPNPATEKRRLIYIG---------LMLLSVIPLAFMIVTEMYEFFMASDLNNTIR 71
BmorOr11        HMNRSHPSIKRNKIWLLQ--------FISLMTLTAFCATGLITSLLFHDLKFGKYMEASK 71
BmorOr12        GLQGIWVDEIKLSRRFHVF-----FKVVTFILHIMCGMFAGLQFFAIFTQNSLNSQQKSD 81
AgamOr33        YIDELWECVEQMQTYHEH---------YLLQGEWFVRRMRRQNLQERMLQDASKLLATLL 70
HvirOr1         KVDVAKPKVKNITTFQDALRATLIIGQVFSLLPFVGVFTNVASNVKFIKTSWKCGYSLLS 79
HvirOr5         YSISILS---------------------LVLVSKLYYNWRTNIAGVWGKVERSVGVKIP 54
TcasOr1         YLFQMRNVPQS---------------YCFVTILFDSVNKLGTQIVNPYSVKQYVMQQQVK 61
TcasOr2         QIFGLITFGCSNRCFFPSK------IRICWNVLLNCVYLFLCGFCVYEFASDERIKLVIK 79


TcasOr16        NTITVLFFTHCVTKFVYFAVRSKLFYRTLGIWNQPNSHPLFVESNN---------RYHGI 127
AmelOr2         NTITMLFFTHSVVKLVYFAVRSKLFYRTLGIWNNPNSHPLFAESNA---------RYHQI 125
AgamOr7         NTITTLFFTHSVTKFIYFAVNSENFYRTLAIWNQTNTHPLFAESDA---------RYHSI 125
AaegOr7         NTITTLFFTHSVTKFIYVAVNSEHFYRTLGIWNQPNSHSLFAESDA---------RYHSI 110
DmelOr83b       NTITTLFFTHCITKFIYLAVNQKNFYRTLNIWNQVNTHPLFAESDA---------RYHSI 127
BmorOr2         NTITVLFFAHSIIKLAFFAFNSKSFYRTLAVWNQSNSHPLFTESDA---------RYHQI 128
BmorOr2a        NTITVLFFAHSIIKLAFFAFNSKSFYRTLAVWNQSNSHPLFTESDA---------RYHQI 128
HvirOr2         NTITVLFFAHTIIKLAFFALNSKSFYRTLAVWNQSNSHPLFTESDA---------RYHQI 128
AgamOr56        AANEQIVFSLICCKLCFYAIHFRRWEKLYYDLQRSFSTVLNNPN--------------- 113
AgamOr57        AANELIVFSLIFCKLCFYAIHFRRWEKLFYDLQRSFSSVLNNPS--------------- 113
AgamOr26        AINEQIVFCIVFLKFSFYAIHFRRWEQLFYDLQRSFSTVVNNPS--------------- 113
AgamOr27        ATNEQILYFIAFFKLYFYVIHYRRWEKLFYDLQIAFSSVMTNPS--------------- 113
HvirOr9         LIPCLILTLISDFKTLSLLYYARHNNEFIVTMKSLLLNQKQLEEKE---------TRF-- 124
HvirOr7         VAPCLTFSILSMIKSLYHLMYEEHIQELIELLRELELRENNREKCI---------E---- 100
DmelOr85b       ALSYIGFVTVGMSKMFFIRWKKTAITELINELKEIYPN-GLIREER---------YN--- 116
DmelOr85c       VLSYIGFVIVGMSKMFFIWWKKTDLSDLVKELEHIYPN-GKAEEEM---------YR--- 115
DmelOr85d       NLSFIGFVIVGDFKIWNISRQRKRLTQVVSRLEELHPQ-GLAQQEP---------YN--- 137
DmelOr67a       LLSYVSFVVMGLSKIGAVMKKKPKMTALVRQLETCFPSPSAKVQEE---------YA--- 129
DmelOr67c       VAPCIGFSLVADFKQAAMIRGKKTLIMLLDDLENMHPK-TLAKQME---------YK--- 128
DmelOr92a       VAPCIGFSFMADFKQFGLTVNRKRLVRLLDDLKEIFPL-DLEAQRK---------YN--- 133
DmelOr69a       MCSSFCLTFVGFCNVYAISTNRNQIETLLEELHQIYPR---YRKNH---------YR--- 124
DmelOr69b       VASMLGFTIVGTLNLWKMLSLKTHFENLLNEFEELFQL---IKHRA---------YR--- 124
TcasOr6         ATESFFTQYGLAWKIAVFVVYKTELAQIIR-LCDNLWPLDEFGTGH---------NFQF- 93
TcasOr11        ASEAMIVQYQLFIKIAVLLKHRKNLVVLMQ-KTRKFWPLDKFGQDA---------KIER- 110
AgamOr41        VLITIFFATIGIGHISVFLLCS-KLLAELLEQSYRTYRLVVD-DQR---------EQRI- 116
AgamOr42        SIVVLFYGIVGVTRLAVAISNP-AGCYRSIQIAEEMYQRANGSNPA---------ECNV- 122
```

```
AgamOr43        SAAIFGVFIQGLAKFYTALRYRKFFEAMYNRLDLFHYEYRNHE--K---------NNAT- 117
AgamOr44        SAAVYGVFIQGLAKFYTALRYRKFFEAMYNRLDLFHYEYRNHE--K---------NNAT- 117
AgamOr66        SLSFLGFFFQCSLKMYYTIQQAPQYAVNFCCLKEAIYERHSKGTLA---------QKTT- 115
AgamOr67        SLSFLGFFFQCSLKIYYTIQQAPQYAVNFSCLKEAIYERHSKGTLA---------QKTT- 115
AgamOr72        VLITMGTAVQLYIKFFVGHSKAQEIMLVTAELEQQVLKRYQNGSEQ---------EIAV- 116
AgamOr73        VLITMGTAVQLYIKFFVAHSKAQEVKLFTVELEQEVLKRYQNGSEE---------ETAV- 116
AgamOr71        VLITLGTAVQLYIKFFVWHSKPQEVKLFTAELEQEVLKRYQDGSEE---------ETAV- 116
AgamOr74        VLITLGTAVQLYIKFFVGHSKAREINLFTAKLEQEVLQRYQNGSEE---------ETDV- 116
AgamOr70        VLITLGTAVQLYIKFFVGHSKSKEVIVFSNKIEQEVFEQNKNRTAG---------ETVL- 116
AgamOr69        ALITLGTCVQLYVKFIIGHKKASELKLLSDNIEQAILQRYENGKAE---------EIAV- 116
TcasOr9         ------------------------------------------------------------
TcasOr18        NPFDLVQEAMIIMSLVFYKN---WQNMVALVTNINKNFHRATDNVI---------EKIS- 105
DmelOr83a       NCLIQTIIYLWTIAMKLYFRRFRPGLLNTILSNINDEYETRSAVGF---------SFVT- 138
DmelOr85e       DALTMTIIYFFTGYGTIYWC-LRSRRLLAYMEHMNREYRHHSLAGV---------TFVS- 147
TcasOr24        DIAILATNTGYILMMLLYII--RQKDLESLLVDLSSFKKYQKPPKF---------D---- 117
TcasOr25        DLAILASNIGYVLMMTMYVS--RQKDLELLLLDLSDFKTYGKPPNF---------D---- 117
TcasOr19        DLGIFAAIIGLVTMMVLYVV--KQKDLSSLLQDLSNFEKFGKPPNF---------D---- 82
TcasOr22        NIAVLSAVTGLTYMLIVFVW--SQDKVVHLLAKLGTHEIFGTPDNF---------K---- 111
TcasOr23        NIAVLSAVTGLTYMLIVFVW--SQDKLVHLLAKLDTHEIFGTPDNL---------T---- 111
AmelOr168       DGTVNMVLLLEVIILNVYLH--SRKKLLRELIGKLNQILINEDEIF---------RNV-- 113
AmelOr170       DGTVNIAISFEVIVFNIYLH--SRKKLLHKLIGKLNHLLITEDEIF---------RSV-- 113
AmelOr169       DSTVNIVISLEVFVLIVYLH--NRENLLRELIGKLNCLLIVDDETL---------RDV-- 115
DmelOr65b       DLAFILGAFFIIFKTYYFCWYGDELDQVISDLDALHPWAQK--------------GPNP 111
DmelOr65c       DIAFIIGFFYIAFKIYYFQWYGDELDEVVEALETFHPWAQK--------------GPGA 111
DmelOr65a       DLVFIITIIFICFRLVFFAQYAGELDVIIDALEDIYHWSIK--------------GPAT 142
TcasOr17        SIFVTLSCLGTVAKTYYLLQNMQMLKELFISIN-KDIFQPK----N---------NKQIL 116
TcasOr21        TAYVLLTEILAVIKTYFVVKNMKMLKHLMQSLNNNKLFQPR----S---------HEQIK 116
TcasOr56        GLCYCVTHVLGTVKMFLMLYLRKKLWGNLTTLE-EGIFKPNPTRGG---------PEELQ 124
TcasOr15        GLCCCVTHVLGAAKMFLMLYLRKKLWGYFTTLE-NGIFKPNPCRGG---------AEEFE 122
AgamOr9         GMFVLLTQLVMILKMEFFYKNVFKIQQ-LIRRLNGKLYQ-S----R---------NAEED 134
AgamOr65        GMFVLLTQLVLILKMEFFYKNVSKIQQ-LIRRLNGKRYQQS----G---------NAEED 138
BmorOr6         ALYMFFTQTTCCSKAIGFYFNFLKIKR-IVASMDDVLFTAM----S---------IEDQA 113
BmorOr10        ASILLFTQASVCYKMTAFISKTNNFVI-LLGLIESEIFSAQ----T---------ELHEK 112
AgamOr34        IMYIFLTETAMILKTLTIYRHFNIVWS-LYETTLGVSFQPR----D---------EQERE 115
AgamOr37        IMYIVFTEIVMALKAIVTYYKFDQLCD-LYRQTLGSDFKPL----D---------AEEEQ 127
DmelOr94a       VLYMSITEMALVVKILSIWHYRTEAWRLMYELQHAPDYQLH----N---------QEEVD 122
DmelOr94b       VLYMSITELALVTKLLNIWYRRHEAASLIHELQHDPAFNLR----N---------SEEIK 119
DmelOr71a       VLLICLTTTALGGKVINIWKYAHVAQGILSEWSTWDLFELR----S---------KQEVD 115
DmelOr46a       AIFILATSAGHTTKLLSIKANNVQMEE-LFRRLDNEEFRPR----G---------ANEEL 114
DmelOr46b       VFFMFATEISCMAKLLHLKLKSRKLAG-LVDAMLSPEFGVK----S---------EQEMQ 114
HvirOr8         NMVFYFTDSATLSKVMTIGFMRKKILQ-LFEMLESDIFQPD----N---------AEGLA 112
BmorOr8         EVIFCFTEITVSTKVLMILFKRNKILD-AFDLLNKNEFRGN----S---------EESSA 114
AmelOr68        SFYMFIASLLSCCKIVALLMNHKAIKI-FRRKLEEEPCKPT----N---------TKEVT 111
AmelOr69        NFYLTLAIFISCHKMYSMLVNRENIIL-VNRMLESEPFQPE----T---------EEEMD 110
AmelOr70        NFYITLVVFVTSCKMTIILRYRKNILS-LMDDLQHEPFSPM----T---------HEENE 111
AmelOr71        NFSVTVVVFVTCFKLITILTRRENILL-LCNTLKQEPLSPI----N---------TEEFE 111
AmelOr72        NFGITITVFTTICKFINLLFRRGIIIS-LLDLLQKEPFLPM----D---------IEEIK 111
DmelOr19a       SLCVTMPHTLYMLKLINVRRMRGQMISSHWLLRLLDKRLGC-------------DDERQ 117
DmelOr19b       SLCVTMPHTLYMLKLINVRRMRGEMISSHWLLRLLDKRLGC-------------ADERQ 117
DmelOr33a       SLHFTSECLFCSYKFFCFRWKLKEIKTIEGLLQDLDSRVES-------------EEERN 111
DmelOr33b       GLPITAACFFASFKFICFRFKLSEIKEIEILFKELDQRALS-------------REECE 113
DmelOr22a       SLEIGVNMYGSSFKCAFTLIGFKKRQEAKVLLDQLDKRCLS-------------DKERS 131
DmelOr22b       SIQIGVNMYGSSFKSYLTMMGYKKRQEAKMSLDELDKRCVC-------------DEERT 131
DmelOr42b       SLQVCINAYGSSVKVAITYSMLWRLIKAKNILDQLDLRCTA-------------MEERE 128
DmelOr59b       SLQVCINVYGASVKSTITYLFLWRLRKTEILLDSLDKRLAN-------------DSDRE 129
DmelOr59c       SLQVDINCIGNVIKSCVTYSQMWRFRRMNELISSLDKRCVT-------------TTQRR 131
DmelOr98a       VMQLFFNSVGMPFKVLFFNLYISGFYKAKKLLSEMDKRCTT-------------LKERV 126
DmelOr42a       SLQVAFNAWSCSTKVLIVWALVKRFDEANNLLDEMDRRITD-------------PGERL 131
DmelOr85a       YVQVPVNTNASIMKGIIVLFMRRRFSRAQKMMDAMDIRCTK-------------MEEKV 129
DmelOr43b       SLQLCLNTWCFALKFFTLIVYTHRLELANKHFDELDKYCVK-------------PAEKR 128
AgamOr2         NGYFTVLYFNLVLRTSFLVINRRKFETFFEGVAAEYALLEKND--D-----------IRP 107
AgamOr10        NAYFAVLYFNAVLRTLILVYNRDKYESFLAGAASVYEEIRAIN--D---------DVITK 108
DmelOr30a       NVYLAVLFTNTVVRGVLLCVQRFSYERFINILKSFYIELLQSD--D---------PIINI 113
DmelOr49b       NSYMLVLWINTVLRAYLLLADHDRYLALIQKLTEAYYDLLNLN--D---------SYISE 107
DmelOr43a       NMFFFSAIFNALMRTWLVIIKRRQFEEFLGQLATLFHSILDST--D---------EWGRG 111
AgamOr32        EIMLIQLCFVMLLKFNLFITYRSGMYNLVEAFKRILKCIDKA---------------EFA 108
AgamOr35        AIPTLVVCMVAISKFYIFVFHPAALFALIDSFKALQRRASRE---------------DLL 107
DmelOr47a       AMVALLITILGLFKFSMILYLRRDFKRLIDKFRLLMSNEAEQG-----------EEYAE 110
DmelOr98b       SLCSVLVDLLALCKIGLFLWLYKDFKFLIGQFYCVLQTETHT------------AVAEM 112
DmelOr9a        TLGSTFASMLTLVKFLLFCYHRKEFVGLIYHIRAILAKEIEVW-----------PDARE 122
```

```
AgamOr38        SICPTVARFSGLLRMCWFLRNEHKIKSALNSVVHLIKNEHPR---------------ETG 127
AgamOr39        SICPTVARISGFLRMVFYLANEEKIHRVLNNISKTLQDEHPR---------------EHT 128
DmelOr24a       ALCPVASSILSLVKMVAIWWYQDELRSLIERVRFLTEQQKSK--------------RKLG 109
DmelOr45b       AFCPLACSFTTLFKLGWMWWRRQEVADLMDRIRLLIGEQEKRE--D---------SRRKV 123
AmelOr74        LTCNMMCILLGLFKCFVISFFRIEFSRIVSYAQKHFWR-LDY---D---------YDEKI 116
AmelOr86        LTCNTLCIVLTMFKFSILFIRRTEFKNLILFARKNFWH-LDY---D---------RHETI 116
AmelOr75        ICCNILYVAIVLLKISVLYAHREEFFNLIAFTQKNFWR--LYD--D---------PQELL 116
AmelOr84        ISCNNLYLMIVVLKVGVLYAHKMEFFDLVTFTRNNFWR--SYP--D---------PEEEL 114
AmelOr87        TACNILCLIMVLLKLFVLFVHRKEFIDLLVYTHENFWH--TNY--T---------NNELL 131
AmelOr76        VACNIITVSLVLIKLLTSFIYNEELLGIIRYAKTNFWH-SNYD--T---------C-EKS 116
AmelOr85        IICNLVTVILVLFKISISLMYRNKLHKIIQYAKTNFWN-LKYD--L---------HDEQI 118
AmelOr88        AGCNVITIVLVLLKIFVLYINNEELLNVVNYAKTNFWRESNYE--P---------H-EKK 117
AmelOr91        SVSNTLSLILPLLKIFILLSNKEDFFRLIVYMQRNFLQ-GNYD--D---------H-ERK 115
AmelOr92        STSNTLSLILPLLKIFILLCHKQDFFRLVLYMKRNFLX--NYD--D---------H-ERK 112
AmelOr95        SMCNMLSIAAPLLKLITLVVHREDFFYLILYLQRKFLH-GDYN--D---------Y-ERN 113
AmelOr93        FISIILSSIMLLLKIIILFSHREDFFHLILYMKRNFLX--NYD--D---------H-ERK 101
AmelOr77        NTCNIIYITMPLLKIFVIVLNKKDFFHLIFYTEKHFYK-DNYD--E---------H-EQR 117
AmelOr94        IVCNSLGLVMDLLKILVVFVHKKKFLGLIAYMQKNFWH-LDYD--Q---------R-ENS 116
AmelOr96        IACDGLGLVMDFFKIFSLFIYEKKFLGLMVYMQKNFWH-YNYD--E---------K-EDL 116
AmelOr79        VVTNNITLIISLVKICNIIIYKGKFLNLIVDMQENFWN-VDY---D---------YHEKE 116
AmelOr83        AMTNTITTIMALIKICIILTYKGKFLNLIVYMQQNFWN-VDY---D---------CQEKE 116
AmelOr80        VTTNNITMIMGMVKICIILIYKKKFLNLIVYMQQNFWN-VNY---D---------HREKQ 116
AmelOr81        ATTNNITMIMGLVKICIILMYKKKFLNLIVYMQQNFWN-LNY---D---------HCEKR 116
AmelOr82        SITNNITMIMALIKISIIIIYKGKFLNLIACMQQNFWK-VNY---D---------YREKE 116
AmelOr78        ALTNNVTMVMGLIKIYIILLYKGKFLNLIVHMQQNFWN-VNY---D---------YHEKE 116
AmelOr89        VVTNALSMMMITVKVCVIVVHKEEFINLIVYMQENFWN-DNYH--D---------LRERE 117
AmelOr90        ALTNYMSVMMILLKICVIVVHKSEFINLILYMQRYFWN-VNY---D---------SREKE 114
AmelOr142       LGKTVSLTAAVAQVTIKMIICRLQQKRFQMLYSEMENFCKQAT--N---------EEKII 118
AmelOr146       FCKAMSLSLAVVQVPLLSSFCITQYDRFQRLIKEMKFCCENAN--S---------YERQV 118
AmelOr148       LFKCICLLICVFQNIIQTIICFIKYDVLQRVVEEMMTCVKEEQ--L---------Y--KV 116
AmelOr158       LFQCICLLICIFKHIIQTVICFIKYNALQRVVEEMMICVKEEQ--L---------Y--DI 116
AmelOr147       FFKSICLLMGVFQHIVQTITCFIKYDSLQRVVEEMMICIKEMQ--L---------N--EI 116
AmelOr150       VFKSIALCVGLSQMIIQTAICFVKYNTLQRVIEEMITYVKEAQ--Q---------YERKI 118
AmelOr151       VSKCVAVSIGLTQVITQTIICFAKYDSLQHVIEEMIICIKAAQ--Q---------YEEKI 118
AmelOr152       VSKSIALSVGLIQVIVQTIICFIKYDSLQHVVEEMIIYVKEAQ--Q---------YEKKI 118
AmelOr154       VFKCICLSIGITQLIVQTLICFIKHNSLQRVVEEMVNCVKQAQ--Q---------SEIEI 118
AmelOr155       ISKCICISIGVTQLIVQTLVCFIKHNSLQRVVGEMMKCVKEAQ--Q---------NEIEI 118
AmelOr157       LFKCICVALCLVQYVAQTIVCLVKYDTLQRVVDEMMGLIEERR--M---------Y--EI 115
AmelOr156       VAKSISIMVVLIQLIVQTTICAIKHDTLQHIIEEMITYMKEAK--Q---------YEKKI 118
AmelOr153       IFKNYHRFIDVAQNIIQTVICFY-------IIKEMKICIKETQ--E---------YEIEI 108
AmelOr144       SMKTASEMAALLEVVLDLVLCKWNNSELQVLIEEVKSFLEMAS--E---------YEIKI 119
AmelOr145       SMKTASEMAALLEVALDLALFKWNNSELQILIEEVKSFLEIAD--E---------YEIKI 119
AmelOr143       SLKTASEMAALLEVVLDLILCKWNNSEFQVLIEEVKSFVEMAN--E---------YEIKI 114
AmelOr1         VIAAQIFSLLAIIKFWTIIINREEIRFWLMEMEIQYRDVECE--------------EDRL 125
AmelOr3         VIAAQVFSLLAIIKIWTILINRNEIRFCLMEMEVQYRDVECE--------------EDRL 130
AmelOr6         LFMLLTNILNQVFKYVITLNRANELRIAIHEIKKDWLTATPE---------------DRF 117
AmelOr7         LFMLLTNILNQVFKYVITLNRANELRIAIHEIKKDWLTATPE---------------DRF 117
AmelOr4         LFMLLISTLNQVFKYVITLNRANELRIAIHEIKKDWLTATPE---------------DRF 119
AmelOr5         LFKLLTNTLNQLFKYIITLNRANELAIAMNEIKNDWLTATSE---------------DRW 119
AmelOr8         MLMPHINSIAQMTKYTIILRRTKELGKLLDEIKKDWSTATQE---------------NRR 121
AmelOr9         MLMPLMSTIAQMTKYTILLRRMKEFNKLLDEIKKDWSTATQE---------------NRQ 121
AmelOr11        LLMPHLNSIAQMAKYTIILQRAKEFSKLLDEIKKDWLLATEE---------------NRQ 119
AmelOr12        LLMSHINGIVQMAKYTILLRKTKEIAKLLDEIKKDWMTASEE---------------NRQ 121
AmelOr10        LLMPHLNSIVQMIKYTILLRQMKLIDKLLDEIKKDWSIATEE---------------NRR 121
AmelOr14        MMIPQVNGYLQLCKYSLVLRWTNKLRVLLNEMKEDWLNTTEE---------------DQL 121
AmelOr15        ILIPLINGYLQLCRYSLVLRSANKLCHLLNEMKKDWMNISEE---------------DRL 122
AmelOr13        MFVPHMNGITQVSKYTILLRQIKEFNIILKEVKRDYSLATDK---------------NMW 113
AmelOr16        LMGPIINCSMQFFKYTIIIWRRKEIQEGLHAIRHDWIQATEE---------------ERL 124
AmelOr17        LMPPIINCSIQCFKYTIILWRRKEIQEGLYAIKHDWIKATEE---------------ERL 124
AmelOr18        LGSALSFFLMAVMKYCVFIIREDDIRKCVELIENDWKNVRYQ--------------EDRK 132
AmelOr19        LGSALSFFLMAVMKMCVFIIRENDIRKCIECIEDDWKNVKYQ--------------EDRK 132
AmelOr22        LFGPLSFCVMAFLKYHLLILHKDNISECIKRIEWDWKNITYS--------------KDIE 133
AmelOr24        LFGPLSFCVMAFLKYHLLILHKDNISECIKRIEWDWKNITYS--------------KDRE 125
AmelOr20        QFGPLIFCMMAFVKYYYLIFHKTDIRECVERIKWDWRNITYA--------------KDRE 131
AmelOr25        MIGPLSFFLMTFMKYYLLLLHENDIREGIECIEWDWKNMKHQ--------------EDRN 126
AmelOr29        MVGPLSFFVMTIMKYYFLLFHENDIREGIERIEWDWKNVKHQ--------------EDRN 127
AmelOr30        MIGPLSFFAMTLIKYYFLTFHEENIRKGIEHIEWDWKNVKHE--------------EDKR 127
AmelOr21        FVSLMSFCMLSITKYHLINIHKDDVRECVKRIEWDWKNISYS--------------EDRE 125
AmelOr23        FVSLMSFCMLSITKYHLINIHKDDVRECVKRIEWDWKNISYS--------------EDRE 125
AmelOr35        MIGPFSFCIMAAIKYYVLLSRGSHIGKCIEDIRVDWFRVSSHN--C---------LEDRK 131
```

```
AmelOr27        TLGPVSHWFVSTVNYTALLMKSKDIRYCFEHMEADWQTIKRM--------------EDQQ 126
AmelOr28        VLGSLAHWFVGTMNYTTLLLRGKEIRLCVEHIRTDWQTVTRE--------------EDQQ 127
AmelOr33        VLGPLSHWFIGGINYTTLLLRKKEIRYCIKHVQRDWRIVTRM--------------EDQQ 128
AmelOr34        VLGPLSHWLVGGINYTTLLLRNKEIRYCIEHMQRDWEIVTKT--------------EDQQ 128
AmelOr32        VLGPLSHWFIGAINYTTLLLRSKEIRYCIEHMQRDWRIVTRT--------------EDQQ 127
AmelOr31        VFGPLSHWFIGGINYTTLLLQNKEIQYCVEHMQTDWKIVNRA--------------KDQQ 127
AmelOr36        SLAPISYILGGGLNYAVLLLRKNDIRYCIDRIEADWKVITRM--------------ADRQ 127
AmelOr37        SLAPISYIFGGGLNYAVLLLRKDDILYCIEHMETDWKTITRM--------------TDRQ 128
AmelOr38        SLGPISYCFGGGLNYAVLLLRKNDIRYCIDHIETDWKVITRM--------------TDRQ 128
AmelOr39        SLAPISYCFGGGLNYAVLLYRKSDILYCIEHMEVDWKAITKT--------------ADRQ 128
AmelOr47        SLGPISYCFGGGLNYAVLLLRKNDIRYCIDHIEADWKVITRM--------------GDRQ 127
AmelOr48        TLGPISYCFGGGFNYAVLLLRKNDIRYCIEHIETDWKIIKRM--------------EDQQ 127
AmelOr40        TLGPISYCFGGGLNYAVLLLRKDDIRYCIDHIETDWKAITRT--------------GDRQ 127
AmelOr43        TLGPISYCFGGGLNYAVLLLRKNDIRYCIDHIETDWKAITRT--------------GDRQ 128
AmelOr45        TLGPIGYCFGGGLNYAILLLRKNDIRYCIEHMKADWKAITRT--------------DDQQ 127
AmelOr41        MTGPIIYCIGGGLNYAILLFLRDDIRYCIEHIEADWKTITRT--------------GDRQ 127
AmelOr42        MTGPIIYCIGGGLNYAILLFLRDDIRYCIEHIEADWKTITRT--------------GDRQ 127
AmelOr44        MIGPISYCVGGGLNYAVLLFLRDDIRYCIDHIETDWNAITRT--------------QDRQ 127
AmelOr46        TIGPLSHWVIGGFNYAVLLLRKNDILHCIEHIRVDWNIITKK--------------QDQQ 127
AmelOr49        ILGPLTHWLVGGFNYAVLLLRKDDIYYCIKQICADWNIITKK--------------QDQQ 127
AmelOr50        IFGPLSHWCIDGFNYAILLLRKNDILHCIEHLRADWKLITRT--------------QDQQ 127
AmelOr26        AIGPTSHWLMGELNYCCLLMRAKEIVYCIEHIKYDWKTVRRA--------------RDRE 131
AmelOr53        AFGPAGFCLSSTIKYCYLGLKGSSFERCIEHMRKDWMMVQDP--------------NHRT 134
AmelOr54        ALGPVSFCVSSTIKYCYLALKGSSFERCIEHMKRDWMMVQDP--------------NHRT 134
AmelOr52        LLGPVGFCLSSTIKYCYLGVKGVFFEQCIKHVKNDWKMVQDP--------------SYRI 132
AmelOr51        FLGPLTFCLSALFKYSYLGIKSSELGHCIKHVEKDWKMLQNE--------------DHRV 132
AmelOr55        FFGLIFFSFMSLMKYWALTIRKPEIEHCIQQVQSDWKQVKME--------------NDRE 130
AmelOr57        FLGLIFFSFMSMMKYWALMIRKPEIEHCIEQVQLDWKQVEIE--------------NDRE 130
AmelOr56        ILGLIFFSFISLMKYWALTIHKPEIKYCIEQVQSDWKQVEME--------------NDRE 130
AmelOr58        ILGLTFFSFISLMKYWVLTMRKPKIKLCIEQIQHDWKQVEFE--------------RDRK 130
AmelOr59        LFGLLSFCLVALIKYWTLLYRKSRIKNCVEQIWIDWEQVELY--------------EDRE 131
AmelOr60        LFGLLMFCSIALMKHWALAYRKPKIKNCIEQIQNDWEQVKLY--------------EDRE 131
AmelOr61        LVGLLSFSLIALIKYWTLTYRKPRIKDCIEQIWIDWEQVELH--------------EDRK 131
AmelOr63        ALILINAGILAVTKVICFHVRSLGLVSNFTSAVKDYKELNSE--------------ENRV 119
AmelOr64        ALVLINGGILAVAKVMCFHIRPLGLISNFTSAVKDYNELNSE--------------ENRV 115
AmelOr66        ALLLINCGILAVAKVMCFRIRPVGLVSNFSSAIKDYNELNSE--------------ENRV 115
AmelOr65        ALLLINCGILAVGKVMSFRVRSTGLVFNFTSAVKDYNESNDE--------------ENRM 115
AmelOr67        ALLLINCGILAVAKVMCFRIRPIGLVSNFSSAIKDYNELNSE--------------ENRV 115
AmelOr114       MG--NITITLSLLKTIAFWINGKPLKSLLNCMANDWIKVTSKT--E------------QE 99
AmelOr115       I---NIPIVFALIKQIVLRYYKKALTELLGEMLADWSGPIGDQ--D------------RE 113
AmelOr118       L---TLSNTLVFIKLIVIWFHKRTFYEILMSMKEDLNNNKHSA--T----------ENKR 105
AmelOr164       I---TCSNTLLLLKFIIIWFHKRVLFESLIIIAEDWDNCKFEW--N------------ME 109
AmelOr163       L---TLSNTVTFFKLIILWFNYRTIHNILTIVFEDWNNRALTD--K----------K-KQ 109
AmelOr165       I---AMPFSLVFIKLIVTWINYGVFCDILSTMEKDCQKYAVID--------------INN 109
AmelOr166       I---CLPHSLVCIKLFTAWTQNTLIRNILLSMEEECQKYAIMD--------------TDN 109
AmelOr167       T---TLPYSLLCFKLISFWTKREIFENILIGMYHDWTNAFATD--F----------IVED 111
DmelOr35a       GMPTYLILVEAQFRSLHILLHFEKLQKFLEIFYANIYIDPRK--------------EPEM 126
DmelOr74a       GLPTYLILVEMQIRCFQLAWHKDRFRALLQRFYAEIYVSEEM--------------EPHL 127
AgamOr3         GTAELMFESNAFFGMLMFSFQRDNYERLVHQLQDLAALVL-QDLPT---------E-LGE 132
AgamOr5         GTAELIFEWNVLFGMLLFSLKLDDYDDLVYRYKDISKIAFRKDVPS---------Q-MGD 113
AgamOr13        GTAELIFQSNTFCGMFWLFLNRHKLAQFITQVRTFSLTVFRE-SPP---------A-VVQ 112
AgamOr15        GTAELIFQTNSFCGMFLLFLNRRKLAEFIQHARSFSQTVICA-SPP---------A-VVR 112
AgamOr17        GIAELFFLTNSFCGMFLLFLNRHKLAEFIRHVRTFSLTVLQE-SPP---------A-VVQ 112
AgamOr16        GLAELFFQTNRFVGVLLLVLYSDSIFELVRQSEFFAKKVLSE-TSP-----------VAE 111
AgamOr55        GLAELFFQLNNFTGLLLVLVSSKQLQCLVRVGQTIADEVFQC-AQT---------D-MRQ 113
AgamOr18        GYTELILETNVFAGMLQLYLCYNHFVPLVDELRAFAAIVFQDNQPM---------L-LRT 113
AgamOr14        SMAELAFVGNVFCGGMALWSVYNSFRQFIEQVIRLTKYLYRDHHPL---------QTVRE 114
AgamOr51        SMAELVFIGNVFGGAMLLWTEYDAFRQFIEQVNSLTKHFYREDP-------------LKE 109
AgamOr12        GLSELIFFTNIFVGLFCFIAQHRPYRELLNAIESFVNIVYRTSSQQPE-------SLSER 117
AgamOr19        GLSELIFFTNVYVGFFCFISQHRHYRDLLNAIDSFVDVVYPTAAHPSD-------SPSEQ 117
AgamOr20        GLSELIFFTNVYVGFFCFISQHRHYRDLLNAIDSFVDVVYPTAAHPSD-------SPSEQ 117
AgamOr21        GLSELIFFTNVYVGYYCFVVQHRHYRDLLDEIQSFVNVVYPTSQQP-E-------SPSER 116
AgamOr50        GLGELIYFTNVYVGFFCFVVQHRHYRDLLDEIQSFVNIVYPTSQQP-E-------SLSER 116
AgamOr29        LGSELAFVSYLYCQILGLYFRRSYLYRLVDLLQTCINKQYSESID--------------Q 109
AgamOr53        LIAELVFVVYLCLQIMALYCRRRQLYRLVDMLQQCIDIPYSEQIE--------------S 109
AgamOr30        VGAEIVYSTYMFLQLIALYARRSDLYRLIDTLQKCVENPYPDDVR--------------A 109
AgamOr46        SIAEGMFSFNTTVTFIMLPLKMDNFEGLLQNLQRFTQIVTVEEDY--------------K 110
AgamOr47        SIAEGMLSFNTTITFIMLPLKMDNLEDLLKNLKRFTEIVIFNEDY--------------E 110
AgamOr48        SVSELIFAALCYLTIIILAIKSEPFRAVIRKMEQALELFRDKQDQ------------CSQ 116
AgamOr49        STAEFIFCYNNYLMMAIFAIRRQPFEQLIGTVQLLFDKERKFQTTD-----------SSR 127
```

```
AgamOr61        SAASVIASVWGFTEGTLRICLIELRYGTLASIMSFLNERS-YRQQD-------------S 127
AgamOr62        AAANLLGALGLCSGALLRLLLIEQNYPTVHRLQQFLNDRT-YLRED-------------P 132
AgamOr63        STANILGVMWFFSVVILRVAILEWYYPLMLRMQTFLNNHTSYQRTD-------------P 129
AgamOr60        ALFNVVGTIWIMAGAFVRRLLFDR--GLLTRLEQFLNDRS-FREDE-------------P 125
AgamOr75        LAMDMLCMLSLFVSIILRGTCMRQYLAHINALDRLERRPT-FRIGT-------------P 126
AgamOr76        LALDMLCMLSLFVSIILRSTCMRQYLSHIDALDRLERRPT-FRVGT-------------P 126
AgamOr78        LAMDMLCMLSLFVSIILRGTCMRQYLAHIDALDRLERWPT-FRVGT-------------P 126
AgamOr77        TFAEQFNQFGGILLTLSRVIAIVGSFPRLKDTAEFINACK-FHHLN-------------E 113
AgamOr79        TFAEQFNQFGGILLTLSRVIAIVGSFPRLKDTAEFINAYK-FHHLN-------------E 113
AmelOr99        CIPHFIASSISAVKLLNLHFNRQNYNILFHFVTKKWQQLKSTYELNAL-------DETIM 130
AmelOr101       CIPHFIASSISAVKLLNLHFNRQNYNILFHFVIKKWQQLKSIYELNAL-------DETIM 130
AmelOr102       GLPHFIAATISAVKILNVYFYRENFDKLFQFVTNEWNKLKLNNELHIL-------DKTII 130
AmelOr103       GLPHFIAATISAVKILNIYFYRENFDKLFQFVASEWDKLKLNNELHIL-------DNTII 130
AmelOr104       CLPHLIAAATSVVKLLNIHFNRENFKKLFEFITKEWEKFELNNQFHVL-------EEITI 130
AmelOr98        CLPNLCASLSSVVKILNVHFNRENFNKLLEFVVKEWDELKLN-ELHIL-------EEITI 129
AmelOr100       CLPSLGVCIVAMFKLQNIYNNSENFKKLFTFVAKQWYQLKLNNEIRIL-------EEIIM 56
AmelOr97        IIPIVFATISCAIKLLNHRINKKNFDKLFDLMSKEWEMKNDRNQTCIL-------DEFTK 83
AmelOr105       NIPQVITVIASMIKILNIYSNKMQFKNLFYSLAQDWKLLESKEELIML-------DKFTQ 130
AmelOr109       QLPYINAGIVTLFKQYNYILNEDKLRELLHDIVSDRLIERSKEELEIL-------EMYSR 118
AmelOr110       QIPYLNAAIICLFKEYNYVLNENKLRELLNDIISDRLIRRSKKELEIL-------ELYSR 118
AmelOr111       QLPYLDVGFVLLFKQYNYILNEDKLRELLNEIISDRLIKRSKEELEIL-------EIYLK 118
AmelOr108       QLPYLNALFILLFKQYNYILNEDKLRDLLNDIIFDRLMVRSKKELEIL-------NMYSR 118
AmelOr107       QIPYIDLGFALMLKQYNYILNEKKLRELLHNIISDRLVKRSKEEEEIF-------EIYFK 118
AmelOr112       NLPYSIASFGTFVKMGNYFLDETKLTTILNHIFEDWATIKSKEEYEIM-------YKYSR 118
AmelOr113       QSGIATAIFLSLLKEANYILNATKVKSLFNDMYMDWRMDRPKKEFEIM-------STYAQ 118
AmelOr106       QIPFLIAAILLMIKYNNFIINEQKFKELFVSILNDWQKKKTHEEEMIL-------EKYAD 115
AmelOr122       TLSAIFPLLLFFIRYIYYITMFPYVEILFDNIRTEENLLQDTTEIQIQ-------TKYLD 118
AmelOr125       TLSAIFPLLLFFIRYIYYITMFPYAKLLFDDIRTEEYLLEDETEIQIQ-------TRYLD 118
AmelOr123       TFSTTFPIVLFCLRYIYCLTLFSYAKVLFDDICIEEHLLQDTTEIQIQ-------TKYLD 116
AmelOr126       TFSMTFPTVLFCLRYIYCLTLFSYAKLLFDDICTEEHLLQDTTEIQIQ-------TKYLD 116
AmelOr127       TFSTTFPTLLFCLRYIYCLTLFSYAKLLFDDICTEEHLLQDTTEIQIQ-------TKYLD 116
AmelOr128       TFSTTFPTLLFCLRYIYCLTLFSYAELLFDNVHTEEHLLEDTTEIQIQ-------TKYLD 116
AmelOr129       TLSTTFPTLLYCLRYIYCLTLFSYTELLFDNIRTEEHILQDMTEIQIQ-------TKYLD 116
AmelOr133       ---------------------------------MEENIIQDSIEAQIR-------TKYIS 20
AmelOr134       ILSTTCPLIIISLRYICFILFFPMIKYLFHHMRMEENIIQDSIEAQIR-------MKYIG 116
AmelOr131       ILSTTCPLIIILLRYISFIIFFPMVKLLFHHICVEENAVQDLIEIQIR-------MKYIG 116
AmelOr132       ILSTTCPLIIISLRYICFILFFPMIKYLFHHMRMEENIVQDSIETRIR-------TKCIN 116
AmelOr135       ILSITCPFIIVSLRYVCFIVFFPTIKLLFHHMRVEENIVQDLIEIQIR-------TKYIN 116
AmelOr136       IFSTTCPLIIVLFRYIGLILFFPTIKLLFHHMCMEEAMIQDSIEAQIR-------RKYID 116
AmelOr138       -------------------------------MRMERNMIQDSTEIKIR-------KKYIN 22
AmelOr130       ILSITCPLVLFLLRYIGSIACFPTIKIVFKHIRTEENIVQDSIESQIR-------MKLID 116
AmelOr137       ILSLIFPLTIILVRYVSCVIFFSMIKLLFHHMRMGGNIIQDSTEIKIR-------KKYIN 115
AmelOr139       ------------------------------------------------------------
BmorOr20        HVYLTIFMTFVTLSRGFSLTWNPNYHKVVKKFITEMHLLYFK---DNS-------EYAMK 132
BmorOr3         HVYLTIFMTFVTLSRGFSLTWNPNYHKVVKKFITEMHLLYFK---DNS-------EYAMK 132
BmorOr18        HVYLTIFMTFVTLSRGFSLTWNPNYHKVVKKFITEMHLLYFK---DNS-------EYAMK 132
HvirOr6         LMYLIFLIGIVDTLTVVCLTFSEKFRVLAKDFLTKTHLFYYK---DRS-------KHAME 137
BmorOr23        HTIITVLMNVVSLSRLILR-CFKKYDVVGQQFINKIHLYHYR---NDS-------EYAMK 132
BmorOr16        HTIITVLMNVVSLSRLILR-CFKKYDVVGQQFINKIHLYHYR---NDS-------EYAMK 132
BmorOr4         HTIITVLMNVVS-SRLILR-CFKKYDVVGQQFINKIHLYHYR---NDS-------EYAMK 131
BmorOr5         HTIITVVMNVVSVSRLILR-CFKKYDVVGQQFINKIHLYHFR---NDS-------EYSMK 132
BmorOr22        HSYITALMTIVIFSRSVVT-ALGRYRKIARYFVSSLHLYHYK---DIS-------EYALQ 138
BmorOr17        HSYITALMTIVIFSRSVVT-ALGRYRKIARYFVSSLHLYHYK---DIS-------EYALQ 138
BmorOr1         STFITVMMNLVTCSRTALVMLFERYLVLTGRFITVMHLFNFQ---KNS-------DYAYK 134
BmorOr1a        STFITVMMNLVTCSRTALVMLFERYLVLTGRFITVMHLFNFQ---KNS-------DYAYK 134
BmorOr1b        STFITVMMNLATCSRTALVMLFERYLVLTGRFITVMHLFNFQ---KNS-------DYAYK 134
BmorOr19        AITITPLGALVGIKAESAK--AECYVNLMKNFMDKVHIHSIYRKNENN-------EFVKK 133
BmorOr15        AITITPLGALVGIKAESAK--AECYVNLMKNFMDKVHIHSIYRKNENN-------EFVKK 133
TcasOr5         LVASFVNYMG----AVIFCHKHKNKFLSNIEKCALLQQRLN------------------K 117
TcasOr7         FVWTFTVYVLWIPMALGFIALTVWYFEDILHHDARINENLTNL---------------IE 120
HvirOr4         FMENIASVDTSIGGQYSRVTERKLCGIILAILIFS-VLIADDFTFYAL-------QAKKL 122
BmorOr13        YLHRIHKINSDLGTYSSSLTDKMLHRLTIGMLLITSVIIVTDFTFVMY-------LADLN 171
DmelOr10a       TLCPAGTSAVTLLKMFLMLRFRQDLSIMWNRLRGLLFDPNWER------------PEQRD 124
DmelOr33c       NLTMSLTCVACSLKHVAHLYHLPQIVEIESLIEQLDTFIASEQ-------------EHRY 113
DmelOr7a        SAQVAIDAVILPAKIVALAWNLPLLRRAEHHLAALDARCREQ--------------EEFQ 132
DmelOr22c       AFCPGTTKAVCVLKLWVFFRSNRRWAELVQRLRAILWESRRQE--------------AQR 121
AgamOr1         ALFVLMTQVTLIYKLEKFNYNIARIQACLRKLNCTLYHPKQR--------------EEFS 125
AgamOr4         NCNIYGGSMFFAYDVATFQAFIQELKSLSVLCSHSYRLKYKLTRFN----------RRAD 124
DmelOr47b       DLVWISGMALVFTKIFYMHLRCDEIDELISDFEYYNRELRPHN--------------IDE 140
AmelOr73        KFSLCISVFGVSLKVANLFLQRGKIINIMNSLTKENSIPRDE---------------QEE 110
```

```
AgamOr68        TQSLICTGAALMIKYFTMIRNPAPVRELSGNIETQMYTKYQEMS------------PEYP 115
DmelOr59a       NLTTFATCTACSVKCLLYAYNIKDVLEMERLLRLLDERVVGP--------------EQRS 116
DmelOr23a       SLSLTVTSLSNLMKFCMYVAQLTKMVEVQSLIGQLDARVSGESQSE----------RHRN 115
AgamOr31        CLGPTFTAYLGLVRQYNLLLHRSELWSIVDEFAALKHSLQSS---------------EIR 109
DmelOr2a        NLTITITDIVANLKFANVYMVRKQLHEIRSLLRLMDARARLVGD-----------PEEIS 121
AmelOr62        AFVMDLSAVISLAKLFILRLNSKHAWVLINSVVEDWSAVHDSRHEYIM-------TEYLK 123
AmelOr121       NLAYSIPLIITTIKFIVVSSKKKVLSLIVNMVAKDWAKLKTDH--------------EKD 123
AmelOr140       GKKITFYYILYVYTTLFVYLLLPTIPLIIDFITSSNHSQKRN------------------ 113
TcasOr12        ASFLLFTNLVQCFKIYTFANHGKKVWNLVYSMNRSDFKPNNLA--------------QYW 121
AgamOr8         LAPCMGFTALSFVKIMTIKLTETKLTDMLHRLDALFPSTVALQ--------------QRY 125
BmorOr9         LAPCICIGVLSVLKILALTAKRQKIYELTQNLECLHKIILNDTR-------------KTE 112
AgamOr40        DELFLTLITTSIYSILVPLSLYLRLYESNIRQLYEFSQRHFRK------------RSAAG 129
TcasOr63        SLLYFFTHFTFLCKLLNFQYYSKDLIEIENFLTDPIFYGYSF--------------EQLD 118
TcasOr4         TLLFCMTQAAFLFKLVNFLYHNKTMLRIESILKNPILNCLDQ--------------FEKN 111
TcasOr13        NTDRFVTFSDVVVVLIPCIIGVPIASHNIEKTIKYFSFLRQFD--------------CCL 130
AgamOr24        CIATFGFCCQAIMKIYSFIITRQRVVDLYEINHRYYQHMMGQS------------IAVKR 108
AgamOr54        SFETLLYVGIACIKMYVFIKNKTLILQMHQYMIDFFDHFHGDR-------------EQDE 133
AgamOr23        HHGPSAQGLAKIEAFTCPELNDLHLYNVARFKAPPRFPEVDEA----------------- 113
AgamOr64        TYGSFVIYMLWAILIWVLNHYEHEMCELRLFFKNPTYQEQSD--------------WAHR 138
DmelOr82a       CLSVVFTNMLTVIKISTFLANRKDFWEMIHRFRKMHEQSSHIPRYR----------EGLD 113
AmelOr116       GLTILMTNSTYAAKVIYIICRYERIKNLVDITNSEIFNRDNDK--------------YKH 124
AmelOr160       NITLSFPTILIVLKAVMFRMNMHLVLPLLTVVKRDVNEGLYRS------------AEERR 118
TcasOr10        IFDTGKPISLCYGESEGLETPWVEFYIVLQSVEVTIIFLGITG----------------- 108
TcasOr26        VIETTAGQFGVLYYLTLFTIYRKGILEIYADLSNFTKFGKPYN--------------FDK 101
AmelOr117       IGCVLTTITQVLFKMIYLIARREKTYSLYYKIRNLWNSSNDSK---------------ER 119
AmelOr120       TGSTLLTILLAALKSIVWIFNRKKLEFFIEFMLTDYWKIIETN--------------VFE 110
BmorOr7         VMFLLLCHITSVAKQLVFYLDADRIDCLIATLDDPSYNEMSH----------------QR 115
AgamOr6         QAPCIGYCTIGVLKMVILARGRNTIAELVGLFRAKWTSAIVTG-------------AHWA 123
DmelOr45a       NFAVFMQGSQSTFKFLVMMAKRRRIGSLIHRLHKLNQAASATP-------------NHLE 110
DmelOr67d       ALAMVGSAVQGLTKLLVTANNASHMREVQNTYEDIYREYGSKG------------DEYAK 123
DmelOr83c       ASLMTGGLFHGLGKFLTCLLKHQDMRRLVLYSQSIYDEYETRGD-----------SYHRT 119
AmelOr162       PLAFRVEYFVDVDKYYYLLLVHSSIGTLGYTVIVLAINSIIIVYVLHE-------CGLCE 125
HvirOr3         FITGMTAAVIGAFSLLALLEGTMSVEAWLPFDP--------------------------- 52
TcasOr20        NFVALSALFAVIYVSVCFINRKHKIRALIADLALFETFSSKA------------------ 108
AgamOr11        NAATTLLFSTTIFRIIFFYFHRARFTSIIKAAHDGIERILKDG----W-------TDEQS 126
DmelOr56a       SYATAVQYFAVSIAMFNAYVQRDKVISLLRVAHSDIQNLMHEAD-----------NREME 126
AgamOr28        VLVPITEILLTNGKMIICNVKRGKIINLINQVQVAWDEGAKSEHLEIQ-------TLITT 143
AgamOr58        MSFVTMTGAMCIVIWLSLAAFRRDLTGTVKFLQHRQCTIHRQS----------------- 122
AgamOr59        VSGGITVLVAVLLLMLIFTVHYDAFQQLGTFLNDRAFARDHP--------------LATG 132
DmelOr1a        ALMHGLQMVSSLLKMAIFLAKSHDLVDLIQQIQSPFTEEDLVG----------------T 115
DmelOr13a       NCCTTFMGVLNFVRLIHLRLNQRKFRQLIENFSYEIWIPNSS----------------KN 122
AmelOr161       CALICTAFYLCVVRLLVYSFHQKDMLYVVNTMKEDWLSSSDQD---------------RL 119
AmelOr141       SVAMIISIVGGHARYTILLWFRDICQTMLNVCEIFWSNLKPH---------------EKK 114
AgamOr52        LLCGQFALTSIVARYMLLLRSYAQLQPIQRHLNEQRFLRGHPR--------------AHA 101
AgamOr36        YGNTFGVLTMMLARMLVVRWYLPHVRQLMEYLRRQRGRIEPAP----------------- 129
AgamOr25        CFSTSAVALEIAIRMGLLVHQRELRNETIEIIRQQQKVTARFH----------------- 107
AgamOr45        VLSVAGCGWQMFFRMYFYLFQQDRCRQIVQEVRDQRTVYGADR------------NPRME 119
TcasOr3         ALRDIGNHVSLAYKAFNYFIMRRDILKLMETLQHGNYHYEDCGDFQPK-------LIVDE 129
DmelOr88a       NPPVLSITIYFSIRGLMLYLKRKEIVEFVNDLDRECPRDLVSQLDMQM-------DETYR 132
AgamOr22        SVSAFVSAFELMLKINGMVYRRKQIAAMLRTVLSDRSSLNGP--------------IEAV 124
DmelOr49a       QGLHFFYMLSSQLKFITFMINRKRLLQLSHRLKELYPHKEQNQ------------RKYEV 123
DmelOr85f       RYATLVTYIINSDTKFATVLQRSAIQSLNSKLAELYPKTTLDR------------IYHRV 122
AmelOr119       MCILAITVFADIYTNMDNLSITTDDGCIFAGIFVVIFKAMNLQ------------IQLES 134
DmelOr63a       TSIAKMWYFLFAHRQIYELLRKARCHELLQKCELFERMSDLPVIKEIR-------QQVES 138
DmelOr67b       AVLLTFQLSVGVVKMFHFQNKVESCSQLVFSTETGEVLKSLGLFQLDLPRKKELLSSVSL 139
BmorOr21        HSTVIGPFIGGFVKVALMYYKRRQANELVSEINRDHLAYNGLKG-----------EDREI 120
BmorOr11        NGTIAMLSFTTTFKYSLLLYLQKSLNRLIAKIDMDYEIAKGLP------------PQEKA 119
BmorOr12        VIVIGISNPMAYIFCINFIRNRNEIKDLFYHLAVVLKIYYND----------------VE 125
AgamOr33        ASCLMVNVRIYGILVQEFTIFEDGLRLIFYSTQCSLGTLFQRR---------------NA 115
HvirOr1         LIGQMFMAVLCVNKLAKSNVSLNGTSPVIFYVTTCVTMMLFFQVARRWPALVQHISKAED 139
HvirOr5         VDRTLKCRMTFVAGLMTFFSIFEHAMSILSSVGLDCPPSLILKR---------------- 98
TcasOr1         NFRHSVRSVFFLSEIFGLVNLKYRETYFRLSKTKTFCTLVTA------------------ 103
TcasOr2         AIIIMILSGCIIFTEGIWICSLIHRKKIVKFLDGIMAFDIQLK---------------QV 124


TcasOr16        ALKKMRRLLYIIIIWTSFSAIAWTGITFVGDSVHNIKDPENENLTITEP-----IPRLLV 182
AmelOr2         AVKKMRILLLAVIGTTVLSAISWTTITFIGDSVKKVIDPVTNETTYVE------IPRLMV 179
AgamOr7         ALAKMRKLLVLVMATTVLSVVAWVTITFFGESVKTVLDKATNETYTVD------IPRLPI 179
AaegOr7         ALAKMRKLLVMVMVTTVLSVVAWITITFFGDSVKNVFDKETNETYTVE------IPRLPI 164
DmelOr83b       ALAKMRKLFFLVMLTTVASATAWTTITFFGDSVKMVVDHETNSSIPVE------IPRLPI 181
```

```
BmorOr2      SLSKMRRLLYFICGMTVFSVISWVTLTFFGESVRMIASKETNETLTEP------APRLPL 182
BmorOr2a     SLSKMRRLLYFICGMTVFSVISWVTLTFFGESVRMIASKETNETLTEP------APRLPL 182
HvirOr2      ALTKMRRLLYFICGMTVLSVISWVTLTFFGESVRMITNKETNETLTEV------VPRLPL 182
AgamOr56     --LEIQTILGHVTKSTHKLTKYYCSTVSFNCAAYGLFP--------------------ML 151
AgamOr57     --LEIQTILGHVTKSTHNLTKYYCSTVSFNCAAYGLFP--------------------ML 151
AgamOr26     --LEIQTILGHVTKSTHKLTKYYCSIVSFNCAAYGLFP--------------------ML 151
AgamOr27     --PEIQGILGHVTKSTHNLTKYYSYVVNFNCAWYGVFR--------------------ML 151
HvirOr9      ----REDLIDKHVLMLTSITKKISYLIGMGLLMFALAP--------------------AF 160
HvirOr7      ----KEEIIASETGFLNKVINVLYVLNCSMIVVFDMTP--------------------MI 136
DmelOr85b    --------LPMYLGTCSRISLIYSLLYSVLIWTFNLFC--------------------VM 148
DmelOr85c    --------LDRYLRSCSRISITYALLYSVLIWTFNLFS--------------------IM 147
DmelOr85d    --------IGHHLSGYSRYSKFYFGMHMVLIWTYNLYW--------------------AV 169
DmelOr67a    --------VKSWLKRCHIYTKGFGGLFMIMYFAHALIP--------------------LF 161
DmelOr67c    --------LPDFEKTMKRVINIFTFLCLAYTTTFSFYP--------------------AI 160
DmelOr92a    --------VSFYRKHMNRVMTLFTILCMTYTSSFSFYP--------------------AI 165
DmelOr69a    --------CQHYFDMAMTIMRIEFLFYMILYVYYNSAP--------------------LW 156
DmelOr69b    --------IHHYQEKYTRHIRNTFIFHTSAVVYYNSLP--------------------IL 156
TcasOr6      --------LHKFLRRFFLLYTGNLALLCTQFAVTAFFDD----------------QFKSV 129
TcasOr11     --------PHKLLKAFFFAYKLIMILMALQYILRKFVSK----------------NGKPL 146
AgamOr41     --------LQWYTRLFQRAVDGYTLVFIGTSVAAGLL---------------------PL 147
AgamOr42     --------LAKYTDLFCKSVQLYTVCFVLSVVVLTLM---------------------PF 153
AgamOr43     --------LLLLMNRICLLRKLITSQFSISILTLMVT---------------------PV 148
AgamOr44     --------LLLLMNRICLLRKFITLQIIGCILVLSLN---------------------PV 148
AgamOr66     --------VARIIDLLVLVTKAISVLYTSSLFIFSLY---------------------PA 146
AgamOr67     --------ITRIIDLLVLMTKAISVLYTSSLFIFSLY---------------------PA 146
AgamOr72     --------LRRTGRILWIVYRMMSASYIFAAVAFGLY---------------------PA 147
AgamOr73     --------LRRTGRILWIVHRLMSASYIFVAVAFGLY---------------------PA 147
AgamOr71     --------LRHTGRILWIVYRIMSASYIFAAVAFGVY---------------------PA 147
AgamOr74     --------LRHTGRILWLVYRMISASYIFLAVAFGMY---------------------PA 147
AgamOr70     --------LKQTGYILWFFFRFVTTLATIVVLAFGLY---------------------PL 147
AgamOr69     --------LQRTGRILWFIFRFMRTSVSSAAFGFFLY---------------------PV 147
TcasOr9      --------MEKSSKLSDKFVFYWTFNCIASSLMPMIAS------------------LLGG 34
TcasOr18     --------MDQASELSDKLAYVWTSSLAVGSVVPVVLA------------------IATG 139
DmelOr83a    --------MAGSYRMSKLWIKTYVYCCYIGTIFWLALP------------------IAYR 172
DmelOr85e    --------SHAAFRMSRNFTVVWIMSCLLGVISWGVSP------------------LMLG 181
TcasOr24     ----------EVNRKLEWCTRMVFGYCVFGSVFYNLVK------------------ILAI 149
TcasOr25     ----------KVRKRMDLYAHLIFFYSMFGSFVYNMDK------------------IILI 149
TcasOr19     ----------KVNKRLDFLVKFVFSYAIFGAVVYNLMR------------------IIEI 114
TcasOr22     ----------KRSRRLDFYAKLYSYYCYFGIVIYSLLQ------------------IIEM 143
TcasOr23     ----------KRSRRLNFYAKLYSYYCYFGIVIYSLVQ------------------IIEM 143
AmelOr168    --------TISTTKMLEKPSRIYIIVNVISIIVWISSP------------------LIKL 147
AmelOr170    --------IIDTVKPLEMPLKIYVIASVASLMIWILSP------------------LIKL 147
AmelOr169    --------TIGTVKPLEKPLRVYIIASVGSLMIWASLP------------------LAKI 149
DmelOr65b    VEYQTGKRWYFVMAFFLATSWSFFLCILLLLLITSP------------MWVHQQNLPFHA 159
DmelOr65c    VDYRTAKRWYFTLAFFLASSWLVFLCIFILLLITSP------------LWVHQQILPLHA 159
DmelOr65a    KEVQETKRLHFLLFMALIITWFSFLILFMLIKISTP------------FWIESQTLPFHV 190
TcasOr17     LVEPSIKFWQRFYLIFRVLCYCTTFFWSSYPILDKW---------------TKDHRLPFL 161
TcasOr21     LIQPSLKFWKLLYNLFHSLVGGATLFWILFPIVDK-----------------KEKRLPFL 159
TcasOr56     IVNDAITMCNRQGYVFYTLVFLIIGARLLYASLANWPYDKHNYFDGNVTVIVNTKEMPYT 184
TcasOr15     IVTSAINMCKRQGYVFYVLTVGVTGGQGLYAALANLPYDKHNYFDGNVTVVVNTKQMPYA 182
AgamOr9      IPLASARKKSTIFWVLYFMFSDGLVTEWLVISLLL-------------------TTLIVP 175
AgamOr65     MPLVSARKKSTIFWTLYFMFSVGLATEWLVISLSL-------------------TTLIVP 179
BmorOr6      TIFSHSR-TVNKLYKGVLGFTGFTLVQWTVLSLIG----------------SERTLPFNE 156
BmorOr10     ILILKAR-KIKRLCMFFLVNAVTTCSLWAVIPLLDI---------------SSKMLPF-K 155
AgamOr34     LQQRRLAVFNRWYYAYIFVSH-MAAFGTGS---HLL---------------SAEYRMPFF 156
AgamOr37     LHRKGVASINRYLYPYLVTTN-LAVASSCL---YLL---------------QDDYRLPYF 168
DmelOr94a    FWRREQRFFKWFFYIYILISLGVVYSGCTG---VLF---------------LEGYELPFA 164
DmelOr94b    FWQQNQRNFKRIFYWYIWGSLFVAVMGYIS---VFF---------------QEDYELPFG 161
DmelOr71a    MWRFEHRRFNRVFMFYCLCSAGVIPFIVIQ---PLF---------------DIPNRLPFW 157
DmelOr46a    IFAAACERSRKLRDFYGALSFAALSMILIP---QFA---------------LDWSHLPLK 156
DmelOr46b    MLELDRVAVVRMRNSYGIMSLGAASLILIV---PCF---------------DNFGELPLA 156
HvirOr8      VIEKAKKFNKLYWNIILAVSFASCASNLFPPIIAHF---------------ILGTELVLP 157
BmorOr8      IIQKNNSAYKTYWKLYAILSNFAYSSQVLGPLIVKL---------------IWKTKLELP 159
AmelOr68     IQKSFDKNIGSITIYYTVMVEFTVFCMIVSS-LVTD---------------FRNQRLAYE 155
AmelOr69     MRDKCDKQARLNAIYYAILVELSVMSLSFGG-LLK----------------AESHKLPYR 153
AmelOr70     IRTKFNKMNERTSICYTILVLVSATWIFVRS-FFTD---------------FKKRKLTFR 155
AmelOr71     IFLKFEKLTDWNTLGYFILLMSSSLCILMGS-LLAN---------------FKIRKLAFR 155
AmelOr72     IHTKYNKLIEKVSIFYTLQNVSCLVALIGAT-LITD---------------FKKKKLTFE 155
DmelOr19a    IIMAG-IERAEFIFRTIFRGLACTVVLGIIYISASS-----------------EPTLMYP 159
DmelOr19b    IIMAG-IERAEFIFRTIFRGLACTVVLGIIYISASS-----------------EPTLMYP 159
```

```
DmelOr33a       YFNQNPSRVARMLSKSYLVAAISAIITATVAGLFST-----------------GRNLMYL 154
DmelOr33b       FFNQNTRREANFIWKSFIVAYGLSNISAIASVLFGG-----------------GHKLLYP 156
DmelOr22a       TVHRYVAMGNFFDILYHIFYSTFVVMNFPYFLLERR-----------------HAWRMYF 174
DmelOr22b       IVHRHVALGNFCYIFYHIAYTSFLISNFLSFIMKRI-----------------HAWRMYF 174
DmelOr42b       KIHLVVARSNHAFLIFTFVYCGYAGSTYLSSVLSGR-----------------PPWQLYN 171
DmelOr59b       RIHNMVARCNYAFLIYSFIYCGYAGSTFLSYALSGR-----------------PPWSVYN 172
DmelOr59c       IFHKMVARVNLIVILFLSTYLGFCFLTLFTSVFAGK-----------------APWQLYN 174
DmelOr98a       EVHQGVVRCNKAYLIYQFIYTAYTISTFLSAALSGK-----------------LPWRIYN 169
DmelOr42a       QIHRAVSLSNRIFFFFMAVYMVYATNTFLSAIFIGR-----------------PPYQNYY 174
DmelOr85a       QVHRAAALCNRVVVIYHCIYFGYLSMALTGALVIGK-----------------TPFCLYN 172
DmelOr43b       KVRDMVATITRLYLTFVVVYVLYATSTLLDGLLHHR-----------------VPYNTYY 171
AgamOr2         VLERYTRRGRMLSISNLWLGAFISACFVTYPLFVPGR----------------GLPYGVT 151
AgamOr10        LVSTYTKRARFLSISNLALGAFISGCFVVYPLFTGQR----------------GLPYGMF 152
DmelOr30a       LVKETTRLSVLISRINLLMGCCTCIGFVTYPIFGSER----------------VLPYGMY 157
DmelOr49b       ILDQVNKVGKLMARGNLFFGMLTSMGFGLYPLSSSER----------------VLPFGSK 151
DmelOr43a       ILRRAEREARNLAILNLSASFLDIVGALVSPLFREER----------------AHPFGLA 155
AgamOr32        RFVKCSELHAKLLRAYVIGTSIVLLLYELNAIVASIT----------------LSLQQN- 151
AgamOr35        LFRRSGAFHAKLTKIYMTSALIVGWFYILSAIVSGIS----------------RSLAEG- 150
DmelOr47a       ILNAANKQDQRMCTLFRTCFLLAWALNSVLPLVRMGL----------------SYWLAG- 153
DmelOr98b       IVTRESRRDQFISAMYAYCFITAGLSACLMSPLSMLI----------------SYHEQV- 155
DmelOr9a        IIEVENQSDQMLSLTYTRCFGLAGIFAALKPFVGIIL----------------SSIRGD- 165
AgamOr38        YTNRITARDQTITKVLFHSSFITAILYGIIPYFMMAY----------------NWFQGQY 171
AgamOr39        ITKQLTTLGQQFTFYLFLMMFFAACLYGVTPFFIMIY----------------NWVQGQR 172
DmelOr24a       YKKRFYTLATQLTFLLLCCGFCTSTSYSVRHLIDNIL----------------RRTHGK- 152
DmelOr45b       AQRSYYLMVTRCGMLVFTLGSITTGAFVLRSLWEMWV----------------RRHQ--- 164
AmelOr74        LFGECQKFCRLWIIVVSMISQS-SLAFYIITPIYENI----------------GKNKSER 159
AmelOr86        LFTKCRKFCTLWTLTVFSFTQA-SLTFYIITPICANI----------------GKNKSER 159
AmelOr75        IITGCKKLCNFSIVLIIFCAQG-TCAGYMVTPLIENI----------------GKNESDR 159
AmelOr84        ILAECKRICTIFVVVISFCAQG-TCTGYMITPIIANV----------------GRNESDR 157
AmelOr87        LLQNCKRISMLCITLINVCAQG-TIVSYVLTPIVENI----------------GRNHSDR 174
AmelOr76        IMNKCQRTCNYLVFVFTFFAQG-TVLGFILRPILVNR----------------GKNESDR 159
AmelOr85        IINTCKRYSTFFVCIFTFFSQG-TVFSFVIRSLKENI----------------GKNETER 161
AmelOr88        IIDDYRRLCSFLVCSFTFFAQG-TVVCFVITPVFVNN----------------GKNESDR 160
AmelOr91        IVFGCKRKCTFFICFFTFFTMA-TIVSYIAGPIIGNI----------------GKNESDR 158
AmelOr92        IVIGCNQKCTFFICFFTFLTIA-TTASYMVIPLIVNI----------------GKNESDR 155
AmelOr95        IVLNCKRKCTFFTCSLTFTTLA-TVVSYVINPLVANI----------------GRNESDR 156
AmelOr93        MIG-CNZKCTSSSVSSRFSRWRPLLLTSSVRLLVKNI----------------GKNESDR 144
AmelOr77        IFTNCRRQCIIFVCFLTFSTKG-TLVCYIVSPLVENI----------------GKNQSER 160
AmelOr94        IIADARQLCVYFVCVFSFFSQS-TVFSYMFMPMISNI----------------GKNESDR 159
AmelOr96        IVKDTKRITAYFVCILTFSSLS-SIFTYMFRPLLTNI----------------GRNETDR 159
AmelOr79        ILDDCKKICIFFISSVTTIGIC-AIISYLMTPFVAQS----------------GSNESER 159
AmelOr83        ILDDCRKTCIFFISSVTTIGMC-TVMSYLTTPVITQS----------------GSNESER 159
AmelOr80        ILDDCRKTCIFFVSCVTIMAIC-AMICYIMIPFIAQS----------------GSNESER 159
AmelOr81        ILDDCRKTCTFFVSSVTSMAIC-AMICYLMIPFIVQS----------------GKNESER 159
AmelOr82        ILNDCRKTCIFFVSCLTTMVIC-AMISYLIIPFIAK-----------------GNNESER 158
AmelOr78        ILDDCRKTCIFFVSSLTTIGIC-AMLSYLMTPFAIRS----------------GNNESER 159
AmelOr89        ILENCKRTCAFFVSLVTAIGIC-AILSYLATPLIVQT----------------ASNNSER 160
AmelOr90        ILNGCKKTCAFFVSTVTFIGIC-AILSYLTTPFTARI----------------GNNESER 157
AmelOr142       LQRYVDRYKYFHSFYILWSFLT---------TIFVIC----------------GPLYTVQ 153
AmelOr146       FQGYAKSYATFYGVSAIWFYWC---------ALIVVV----------------GTLFISD 153
AmelOr148       LCIYVKKCNIFYGGTIVLTYGA---------ATVFVL----------------GPTFLPI 151
AmelOr158       LCMYVKKCNIFYGGTIVLIYGT---------ATVFVL----------------GPIFLPI 151
AmelOr147       MCAYVAKCNIFYGGTIVLIYTT---------ATVFIL----------------GPTFLPI 151
AmelOr150       FHKYIKKCYTFYGCSIICMYLT---------GLAFII----------------GPAFSPA 153
AmelOr151       FHKYIEKCYTFYACSITCMYLT---------ATAFII----------------GPAFSPA 153
AmelOr152       FHKYIEKCHIFYGCSIACIYLT---------ATVFVI----------------GPVFSSA 153
AmelOr154       FYKYIEKCKIFYGSSIAFSYLA---------ATAFML----------------GPAILPI 153
AmelOr155       FSKYIEKCKIFYGSSIIFSYLT---------STAFML----------------GPIILPI 153
AmelOr157       LRAYASKCNTLYGASIASIYVC---------GTSFIF----------------APLFLPN 150
AmelOr156       IQKYVSKCYILYGSAIIISYLT---------TTIFIL----------------GPIFLPI 153
AmelOr153       FQKYIAKFKTVWGCNITCMYLT---------ALAFTI----------------GSVFIST 143
AmelOr144       LQGYINRYKKFFSTVSMGYILP---------ASSFIL----------------MPLFSAQ 154
AmelOr145       LQGYINRYKKFFSTVSMGYILP---------ASSFIL----------------TPLLSDK 154
AmelOr143       LQGYVNRYKKFFSTVSMGYIST---------AISFSL----------------MPLFSAQ 149
AmelOr1         VMMNTAKIGRFFTIVYLSLSYTGALPYHIILPLIS------------------ERIVKED 167
AmelOr3         VMMNTAKIGRIFTIVYLFLGYGGALPYHVILPLIS------------------ERIVKAD 172
AmelOr6         IFVTNSRIGQRIMLIIAVITYSSGLGYRMVLPLLK------------------GKIVLAN 159
AmelOr7         IFVTNSRIGQRIMLIIAVIMYSSGLGYRMVLPLLK------------------GKIVLPN 159
AmelOr4         IFVMNSRIGQRIMLIMAFIMYISGLGYRMVLPLLK------------------GKIVLPN 161
AmelOr5         IFTANSKMGQKVMLIVAVTVYSSGLGYRMLLPILK------------------GKIVLPN 161
```

```
AmelOr8         IFSERASIEHKLTMIVAITIYGGGFLYRAILPLSK------------------GRIVLPN 163
AmelOr9         IFSAKASIEHKLTTVIAITIYGGGIFYRMILPLSK------------------GRIVLPN 163
AmelOr11        IFSERASIEHKLTTVIVVTMYGGGFFYRTILPLSK------------------GKILLPN 161
AmelOr12        IFSTRASIEHKLTMVVVVTMYGGGFFYRAILPLSK------------------GKIVLSN 163
AmelOr10        IFSRTASVEHKLTSIIAITIYSGGFFYRMILPFSK------------------NKIVS-N 162
AmelOr14        IFRAKASFGHRVMSMIAIVTYSAGLGYRTILPLSK------------------GRILLPN 163
AmelOr15        IFRRKASIGHRLMSVVAIIMYSAGLGYRTFIPLSK------------------GRILLPD 164
AmelOr13        IFTTRAYIGHKMMIAIAIAMYSSGVGYRMILPFLK------------------GRILLPD 155
AmelOr16        IFRSKMKIGRRVVLIAAFTMYGGGLCYRTILPLLK------------------GTVITAD 166
AmelOr17        IFRSKAKIGRRVVLVVAFTMYGGGLCYRMILPLLK------------------GTIVTAN 166
AmelOr18        IMLENASFSRRLIVICGTFMYGGVIFYYIALPLTRA-----------------KIVEEGG 175
AmelOr19        IMLENASFSRRLIVICGAFMYGGVVFYYIALPFTRA-----------------KVVEEGG 175
AmelOr22        IMITNANFGRRLVIICTFFMYSGFAFYYIAVPISVG-----------------KILAEDD 176
AmelOr24        IMITNANFGRRLVVICTFFMYSGFAFYYIAVPISVG-----------------KIPAEDD 168
AmelOr20        IMIMYANFGRKLVMVCTFFMYSGFAFYYIAIPISVG-----------------RVKT--D 172
AmelOr25        IMIEYANYGRKLVLICTFFMYSAFAFYYLVLPFSVG-----------------KIED--G 167
AmelOr29        IMITYANYGRKLAFICFFFMLCAFIFYFLIQPFGGG-----------------KIVD--G 168
AmelOr30        IMIEYANYGKKLALISIFFVYSAFVFYYFVVPISVG-----------------KIRD--E 168
AmelOr21        IMLMNANFGKRLIIVTTTVTYSGFVFFYIAIPMKIG-----------------KIPAPDA 168
AmelOr23        IMLMNANFGKRLIIVTTTVTYSGFVFFYIAVPMKIG-----------------KIPAPDA 168
AmelOr35        IMMENARIGRSLAIFCAGFMYSGGFFYTTVMPLCT------------------KRTEIID 173
AmelOr27        TMLKNAKFGRYVAASCAIFMQGGILCFCFVTILTTE-----------------TIQVGNE 169
AmelOr28        VMLKNAKFGRYVAAVSAAILQSGVNCXCCMTISRTE-----------------LIQIGNE 170
AmelOr33        VMIKHAKIGRYISMMCAAFMQGGVLSYCAVTAFSTQ-----------------TIEIGNE 171
AmelOr34        VMIKHAKIGRYITMFCAAFMQGGVLSYCAVTAFSTQ-----------------TIEIGNE 171
AmelOr32        IMMKHAKIGRYIAVFSAAFMQGGVLSNCAVTAFSTQ-----------------TIEIGNV 170
AmelOr31        VMMKYAKIGRYIAALCAIFMQTGVLTYCVVTAFSTR-----------------IIEIGNE 170
AmelOr36        VMLKNAKIGRIISCCIVGFMQLGTFCFCTILGVFKR-----------------TIKIGND 170
AmelOr37        IMLKNAKIGRIISCCILAFMQVSAVCFCTVLGVFKR-----------------TIKIGNE 171
AmelOr38        VMLKNAKIGRIISCCIVGFLQIGTFCFCTILGVFKR-----------------TIKIGNN 171
AmelOr39        IMFKNAKIGRIISCCIAAFVQISAVCFCTVLGVFKR-----------------TIKIGNE 171
AmelOr47        VMLKNAKIGRIISCCIVCFMQIGTLCFCTILGVFKR-----------------TIKIGND 170
AmelOr48        VMLKSAKIGRIISGCIAGFMHIGTFCFCIVLGVLKR-----------------TIKIGND 170
AmelOr40        VMFKNAKIGRIISGCIASFMQVSTICFGIVFGVFKQ-----------------KIKIGNE 170
AmelOr43        VMFKNAKIGRIISGCVAGFLQLSTISFCTVFGVFKR-----------------RIKIGNE 171
AmelOr45        IMLKNAKIGRIISCCFAAFMQFSTVIFCAVFGVFKR-----------------TIKISNE 170
AmelOr41        VMFKNAKIGRIISGCIGSFLQFSTISYCTVFGVFKQ-----------------TIKIGNE 170
AmelOr42        VMFKNAKIGRIISGCIGSFLQFSTISYCTVFGVFKQ-----------------TIKIGNE 170
AmelOr44        VMLKNAKIGRIISGCIAGFMQLDSICFCTVLGVFKQ-----------------TIKVGNE 170
AmelOr46        VMLKYAKIGRYIAAFCTAFLQGGVLCTCIALGAFKT-----------------TIKNGNE 170
AmelOr49        VMLKNAKIGRYVAVFCTVFLQGGVFCTCLALGAFKK-----------------TIKVDNE 170
AmelOr50        VMLRNAKMGRYIAAFCAIFMQVIIFFTCFILGIFKR-----------------SIHIDNK 170
AmelOr26        LMIKNAKLGRFIACIAALCMHSGIMSYTVITGFKKI-----------------TFQIGND 174
AmelOr53        IMLKYATISRKLITMCAVFLYTGGMSYHTIMQFLS------------------KGKNK-D 175
AmelOr54        IMLKYATISRRLITICAVFLYSGGMSYHTVMQFLS------------------KGKN--- 173
AmelOr52        IMLKYATISRKLIIMCAVFLYTGGMSYHTVMQFLS------------------KEKD--N 172
AmelOr51        IMSRYVIMGRNLITLCAAFMYTGGLSYHTIMPLLS------------------KRKV--E 172
AmelOr55        LMLKYGIIGRNLTIYSILFMYISGIMYISFMQYAM------------------RLQINND 172
AmelOr57        LMLKYGIIGRNLTIYSILFMYLSGIIYVSIMQYAM------------------GSQINEH 172
AmelOr56        LMLKYGILGRNLTIYSILFMYMGSITYMSITQYAM------------------GLQFNEH 172
AmelOr58        LMLKYGIIGRNLSMYSIVFMYSGGIIYHTVMHYKL------------------GSYVDEY 172
AmelOr59        MMLKYGQMGRNLMIICAMFTYTGGTIFHTILQYKV------------------GTFIDEY 173
AmelOr60        MMLKYGQVGRNLTIICAVFMYTGGIIYHTILQYEI------------------GTFIDEY 173
AmelOr61        VMLKYGQIGRNLTIICAVFIYTGGSIFHTILQYKI------------------GTFIDEH 173
AmelOr63        IVRRHAYMGRAACISLIFCSYVGCTLFMIVPIVAGDK----------------EEVINVT 163
AmelOr64        IVRRHAYMGRVACASLIFCSYVGSTLFMTVPMLAGDE----------------EEVINVT 159
AmelOr66        IVRRHAYMGRVACASLIFCSYAGSTLFMTVPMLAGDE----------------EEVINVT 159
AmelOr65        IMRRHAYMGRVACTSLISCSYVCSTLFITVPMLAGDE----------------IQVINAT 159
AmelOr67        IMRRHAYMSRVACASLISCSFIASTLFMTVPMLTGDK----------------KDIINVT 159
AmelOr114       TMARIASITRNTIIKSTVMCHTVVAFYVFLRYISMKY----------------NENK--- 140
AmelOr115       TMLRNARLSRAISIVCSTLTYFMLLAFVSLQVWSNAE----------------NASETDL 157
AmelOr118       IIMDKSMLSSRISNFLISYFAITFFLYS-----GVAL----------------VIFDED- 143
AmelOr164       IMMQKAILSYRIAKLMLIIFICSIFMYAVSTFFGPDI----------------GASHSD- 152
AmelOr163       LMVDNTRLSSRISNFLFGIYSVTCILYS----ASIAL----------------ISDDIDN 149
AmelOr165       LISKTGQISFYTTTIVMSSYLVSAAFYITGTLAF---------------------QRTNS 148
AmelOr166       LISKTAYLSYRLTSTIICTCVASTVCYAIGIFSH---------------------QEVNV 148
AmelOr167       MIKKT-ELAYYCSNLILSIYAIAVFLYVGVFLELSHD----------------HDQENRS 154
DmelOr35a       FRKVDGKMIINRLVSAMYGAVISLYLIAPVFSIINQS----------------KDFLYSM 170
DmelOr74a       FASIQRQMLATRVNSTVYLLALLNFFLVPVTNVIYHR----------------REMLYKQ 171
AgamOr3         YLISVNRRVDRFSKIYCCCHFSMATFFWFMPVWTTYSA-------YFA-----VRNSTEP 180
```

```
AgamOr5         YLVRINHRIDRFSKIYCCSHLCLAIFYWVAPSSSTYLA-------YLG-----ARNRSVP 161
AgamOr13        HLTTQHDFIHKITRIYCIVVMFAAHFYVLTPLLSTFYA-------FYG----TVRNDNVT 161
AgamOr15        HLTTQHDMIHKITRIFCIVVMFAAHFYALAPFLSTLYT-------FYG----TVRNKNAT 161
AgamOr17        HLTTQHDFIHKITRMYCIVVMFAAHFYVLTPFLSTLYA-------FYG----TVRNENAT 161
AgamOr16        YLTKMDTQVTKITKLYLTALLVPANFYSYSPIVATLWK-------YYN----TYEN-DTV 159
AgamOr55        LLTSHHKRMHTWTRYYCLVILYTVSIFATAPICATFWS-------YVR----AAHR-NTT 161
AgamOr18        NLTDLNLRIHKYTLCYCLYMCCVCTVYCVAPLGSNIAG-------YTAAMADTASNSSTT 166
AgamOr14        QLLQFNHRIHRYTNRYCFSMMILIVFYLVAPVITSFGV-------YFQSVWQSHHDTNDT 167
AgamOr51        HVLQFNYLIHRYTNRYCFSVMILIMFYLAAPVITSFVV-------YFQSLWKSYHLANGT 162
AgamOr12        TLIKLNVKINQISVVYCYYVAVAAFTYWIAPCLITYLS-------IHKEQDIAGNGTMNA 170
AgamOr19        ILIKFNVKIQKLSVVFCWYIAVTATFYWLAPCLMTYRS-------IYMASVSVENGSVQS 170
AgamOr20        ILIKFNVKIQKLSVVFCWYIAVTATFYWLAPCLMTYRS-------IYMASVSVENGSVQS 170
AgamOr21        TLIKLNVKINKISVLYCWYLAAAGLIYWSTPCLMTYHS-------VLKAKAEYGPN--HP 167
AgamOr50        TLIKLNVKINILSVLYCRYIVVAAFIYWMVPCVVTYSS-------IHKAEVSMGNESIQS 169
AgamOr29        FVIKSNTKINKLSVTCCKYFTIAYVLYCAMPPIASTVV-------YVRNQR-NKTAEPEE 161
AgamOr53        FLIRSNVKINQSSAAYARFFMCVYVLYCTMSPLASGFV-------YIRNQR-NATGVQED 161
AgamOr30        FILITRDTINKSSVMYSKCFFAVCISYIIMPFMATSAV-------VVRNRR-NQTGEREE 161
AgamOr46        QILISLNTTIHKFTKFYFIFTNGVVFSITGSTIAGMLY-------TYYTKD-SEYSAAFP 162
AgamOr47        QILIRLNTAIHKFTKYYFIFTNGIVFAMTSSTIAGMFY-------TYYAKD-SEYSAAFP 162
AgamOr48        LIIEVNANIHRFSLGYAKLNLLYVLLFNVIPPIYNYPR-------YFLQWRMPEDNRTVE 169
AgamOr49        YVVYYVNRQIMRYSRLYIIVQGVYFLIFNLLPAIVTYSA-------YFASS---GDGEPVE 177
AgamOr61        LVRQQRATLFGENNRIQLILVATMLMEAIWFMTTQLFS-------RDAFMLQVNGHVVDS 180
AgamOr62        WARTERSKLYRHNNRFLVVLISAISVESLCFLARLLLT-------RPEFMLQYGGAVLGG 185
AgamOr63        WAVGRRARFYRRTNRVILTVMGINLAETVCFTATNVMK-------LDEFMLQFRGAIVGG 182
AgamOr60        LVRAARQHVKVQNNRYLFAVCLTLVLEASIFSGTNLML-------QPEFMLLYQGHAVGG 178
AgamOr75        YADESRRNVALQNSRYLGVALVFHSLTVTVYVIQNMVR-------ENSFVKIITSFPIDL 179
AgamOr76        YADESRRNVALQNSRYLGVALVMHSLTVTMYVIQNMVR-------ENSFVKIITSFPIDL 179
AgamOr78        YADESRRNVALQNSRYLGVALVMHLLTVTVYVIQNMVR-------ENSFVKIITSFPIDL 179
AgamOr77        RAEEIRAGSIHNAGRFLSTLLAVQIVTLIFWFVLTELQ-------AHRQDVLLPIFIYLP 166
AgamOr79        RAEEIRAGSIHNAGRFLSTLLAVQIVTLIFWFVLTELQ-------AHRQDVLLPIFIYLP 166
AmelOr99        QGKKMAQLYRNTLFSFLILFLVVPLVSPILDIVHPLN-----------------QTRSR 172
AmelOr101       QGKRMAKLYRNTLFSFLILFLLVPLVSPILDIVHPLN-----------------QTRSR 172
AmelOr102       RGNRTAHLYRSALLIALVLFLLIPLISPMLDVFLPLN-----------------ETRPR 172
AmelOr103       QGNKMAQLYRSALLTALILFLLIPLLSPILDIVLPLN-----------------ETRPR 172
AmelOr104       KGSKMAQLYRNTLLSFMVLFLLVPLIFPFLDIVHPLN-----------------ETRPR 172
AmelOr98        QGSKIAHLYRNTLLSFLILFLLVPMYFPILDMIDALN-----------------QTRSR 171
AmelOr100       QGNKMAQIYKNTLLLSMTIFFFVPLIFPILDIVYPLN-----------------ETRPR 98
AmelOr97        QGNKFAEIYKNVLLSALLLFLLLPLFPSFLDIVFPLN-----------------ETRQQ 125
AmelOr105       YGSKLALLYRRTLLTFLVIFLFLPLCNPILDVILPLN-----------------ETRSR 172
AmelOr109       RTTALCALYKVMVYSCAFMFLVIPTIPPILNIVAPLN-----------------VSRSR 160
AmelOr110       KATTLCILYKVMVYSCAFMFLVIPTIPPILNIVAPLN-----------------VSRSR 160
AmelOr111       RARVLSTVYEVSIFFCGFMFLLIPSIPPILNIISPLN-----------------ESRGR 160
AmelOr108       RATTLCIFYEVIVIFSAIMFIMIPTIPPILNIIMPLN-----------------ESRDR 160
AmelOr107       RAMFFCSFYEVSIYSCGFMFLSMPSIPLIMNVIMPLN-----------------ESRSR 160
AmelOr112       RGLFITISYFLHIGVTETFMLILPMVPPILDIIVPLN-----------------VSRKR 160
AmelOr113       RGSFLAMFYFINAYCCSLLFLQVPWTARLLYMIKSQN-----------------TSPPM 160
AmelOr106       KSLFFILIYVVNAYFCTVLFLILPLTPILLDIFIPLN-----------------ESRPR 157
AmelOr122       ISSHIIYIFCCMTFAFIVAAIIFLVNPVILDLRNPLN-----------------ESRIF 160
AmelOr125       ISSHIIYIFCCMTFAFIAAAIIFLVNLIILDLRNSLN-----------------EFR-F 159
AmelOr123       ISSHIIYIFCWLSFICVASTWIFILNPVILDVIMPLN-----------------KFR-L 157
AmelOr126       ISSHIIYIFCWLSFICAAASCILILNPVILDVIMPLN-----------------KFR-L 157
AmelOr127       ISSHIIYIFCWLSFICAAASCIFIVNPVILDVIMPLN-----------------KFR-L 157
AmelOr128       ISSHIIDIFCWMSFICVASTCIFMLNPVILDVIMPLN-----------------KFR-L 157
AmelOr129       ISSHIIDIFCWLSFICVAATWIFILNPVTLDVIMPLN-----------------KSR-I 157
AmelOr133       DSRHMIEILLWMAYATITLYSILGLCPII--FIILLN-----------------ESPIR 60
AmelOr134       DSRHMIEIFLWMAYANITLYSILGLYLII--FIMPLN-----------------ESPIR 156
AmelOr131       NSRHMIEILLRVTFLTITLFSIFLLYFVTMDFIMPLN-----------------EFHRH 158
AmelOr132       DSCHMIDIFLWMIYAIFAFCIILLLCPIILDFIMPLN-----------------ESRIY 158
AmelOr135       DSCHIIDIFFWVACTNITLSSISLLYFITLNFIMPLN-----------------EFR-- 156
AmelOr136       DSCYMIDIFFWMTYVGIALCSILLLCPITLDFIMPLN-----------------ESRTR 158
AmelOr138       DSCHMINIFFZIIYGIAVLSIIFILYPMTLDFIMPLN-----------------KTRIH 64
AmelOr130       DSHHIINIFFWMTYTTIVIFIIYVSYPIILDFMIPLN-----------------ESRTH 158
AmelOr137       DSCHMMNIFFWIIYGIAALSIIFILYPMTLDFIMPLN-----------------RTRIR 157
AmelOr139       -------------DAVIAFFSISLLYPIILDFINSLN-----------------ESRTR 79
BmorOr20        THRRVHKISHFYTVFLKVQMIAGLTLFNVIPMYNNYRQG-------------NYASDRPA 179
BmorOr3         THRRVHKISHFYTVFLKVQMIAGLTLFNVIPMYNNYRQG-------------NYASDRPA 179
BmorOr18        THRRVHKISHFYTVFLKVQMIAGLTLFNVIPMYNNYRQG-------------NYASDRPA 179
HvirOr6         IHKKIHLISHLFSLWILFQMLSGLSLFNIIPMYSNLAAG-------------KYRKGGLQ 184
BmorOr23        IHTVVHKISHNMTYIFSFCIIFGTVTFNLTPIFNNIGSD-------------AYKNPRPD 179
BmorOr16        IHTVVHKISHNMTYIFSFCIIFGTVTFNLTPIFNNIGSD-------------AYKNPRPD 179
```

```
BmorOr4         IHTVVHKISHNMTYIFSFCIIFGTVTFNLTPIFNNIGSG-------------AYKNPRPD 178
BmorOr5         TYKAVHKISNNMTYIFSFSIFVCVVTFNLNPVFNNIGSG-------------AYKNPRPD 179
BmorOr22        THLLVHRLSHYYTVYLISLVVTGMLLFNITPLYNNISSG-------------VFNSPRPE 185
BmorOr17        THLLVHRLSHYYTVYLISLVVTGMLLFNITPLYNNISSG-------------VFNSPRPE 185
BmorOr1         LCTFVNRMSHFYTLYVLFSMFMGLGLFNLLPLYNNYVSG-------------AFSDPYGP 181
BmorOr1a        LCTFVNRMSHFYTLYVLFSMFMGLGLFNLLPLYNNYVSG-------------AFSDPYGP 181
BmorOr1b        LCTFVNRMSHFYTLYVLFSMFMGLGLFNLLPLYNNYVSG-------------AFSDPYGP 181
BmorOr19        KVIQIERVSRFTAYFLVILIAINCLSWMLKPTLHNIK---------------HFEEIMNK 178
BmorOr15        KVIQIERVSRFTAYFLVILIAINCLSWMLKPTLHNIK---------------HFEEIMNK 178
TcasOr5         LGKKEHSFNKMAKILLTIY-TTKLSSMIVVTVNDIVK----------------------- 153
TcasOr7         ATKQDYIFRYCFYTGFVFYGASDFTILVLITPVEKLYG---------------------- 158
HvirOr4         DREWDVVTNYLGFYLLWFVVLILELQFAFTALSVRAR-----------------FSAVND 165
BmorOr13        HRQLLIAIMYWCYYCSYFIAHLLEMQFVLIAALALSS-----------------LKLVNN 214
DmelOr10a       IRLKHSAMAARINFWPLSAGFFTCTTYNLKPILIAMILY-------------LQNRYEDF 171
DmelOr33c       YRDHVHCHARRFTRCLYISFGMIYALFLFGVFVQVISG--------------------NW 153
DmelOr7a        LILDAVRFCNYLVWFYQICYAIYSSSTFVCAFLLGQP--------------------PYA 172
DmelOr22c       MLVGLATTANRLSLLLLSSGTATNAAFTLQPLIMGLYR-------------WIVQLPGQT 168
AgamOr1         PVLQSMSGVFWLMIFLMFVAIFTIIMWVMSPAFDNER---------------------RL 164
AgamOr4         IIAKVQTTCMGAVTLFYWIAPIPSICAHYYRSTNSTEP----------------VRFVQH 168
DmelOr47b       EVLGWQRLCYVIESGLYINCFCLVNFFSAAIFLQPLLG---------------EGKLPFH 185
AmelOr73        IIQRRNDNYARKVTIYCEILNESAVFFATVGQYKRFIN--------------------TR 150
AgamOr68        VVLKYGRVLYIAGHIMIGGYFGSLFIIWINPLIMYFKE---------------GRVMLLF 160
DmelOr59a       IYGQVRVQLRNVLYVFIGIYMPCALFAELSFLFKEER---------------------GL 155
DmelOr23a       MTEHLLRMSKLFQITYAVVFIIAAVPFVFETELSLPMP---------------------- 153
AgamOr31        IVQKYNRIDRFLAWAYLITAMSTGVLFVGVALVLVFLS-----------------EKSDW 152
DmelOr2a        ALRKEVNIAQGTFRTFASIFVFGTTLSCVRVVVRPDR---------------------EL 160
AmelOr62        KGRIVSLMILYLGYASGFSFIVKALPFGDILPFQMFQNS--------------RNSSMNP 169
AmelOr121       IMIRRARIARIINIFGYILICILIWLLMILPRFGITIR--------------YVTNETDA 169
AmelOr140       FLFELDYGMDKQQYFYYISIHSYIGTAIVANLIASCD----------------------- 150
TcasOr12        IVVNEIKTSKIISKLFLLACTLTCVSWAISPLLDKRGS-----------------DELRL 164
AgamOr8         GVYQYNRESQVVMKSFSILYMILIWMFNLLPLVSMVAG---------------YVSDGTW 170
BmorOr9         LVRKNLVLIKFITKYFFVLNAVLIFVYNFSSPVIIAYN---------------YIVSNEV 157
AgamOr40        VHYISISSSYRFTNKYQFWWIASCLLGTMHWAVYPILS-------------------QER 170
TcasOr63        IIKAKIRSCAFISNAFRICCTFTCSFYCLVPFIDESR-------------------KKIL 159
TcasOr4         IIEKYMIRVKYLARLFRILCILTVSFYGLFPFIDEDPD-------------------HML 152
TcasOr13        QKQPPKTWKSFFVPIITIVFTATILLFDAVMWLTLIAR-----------------NQSIF 173
AgamOr24        VLCDNASLIYIVIKVTVLAYLLLVITAMSIPGLSSIF-------------------LADR 149
AgamOr54        LLVRTLNNTTLLSSLFAVCSSSAPGLLFVG------------------------------ 163
AgamOr23        -LFHTATMCMVFIRIFAVAFSIVAIAIYSYAILMPLIEH-------------------EL 153
AgamOr64        IRAGNYRRWNWMILMFYLFNVINVSIFTLTNAHNRQFH----------------FQTRGE 182
DmelOr82a       YVAEANKLASFLGRAYCVSCGLTGLYFMLGPIVKIGVCR-------------WHGTTCDK 160
AmelOr116       IISYYNWQGIFHHIAYQGFASICIFSYSCIPLQSAFSG-----------------KSKQL 167
AmelOr160       TVVWYNVAATLFSTSSALSLFFVPTLFYAKPIIGCLLS---------------KYNNCTL 163
TcasOr10        ----YDMVFLYYAGSVCIQFQMLKMAFAERKMNE-------------------------- 138
TcasOr26        RNKQLNQWSRWFSVVLYFFVISVFAWPGIFTQSCEDLN-----------------VALNK 144
AmelOr117       PYEEFAYWARIFSIIFYSSCMCNVFTFSIAAAIDYFKFEYN-------------ANNTEN 166
AmelOr120       YLQEYAIYAKNITKGYFFSMCNALLFFFSLPIIETLTK-----------------NENLN 153
BmorOr7         LLVDASRWASRFVWAYSGCAVVTCTLWIVFPIIYHVQG-------------------QTV 156
AgamOr6         VCEDTMRPAIRVTSVTALANVVMGIAFTILPIAEMIYT-------------HHYTGRWNR 170
DmelOr45a       KIERENQLDRYVARSFRNAAYGVICASAIAPMLLGLWG---------------YVETGVF 155
DmelOr67d       CLEKRIRITWTLLIGFMLVYIILLGLVITFPIFYLLILH---------------QKVLVM 168
DmelOr83c       LNSNIDRLLGIMKIIRNGYVFAFCLMELLPLAMLMYDG----------------TRVTAM 163
AmelOr162       ILRESNNMICKIFLKDSLDIYTLDKIMNQFIIVQGETR-----------------KLCGN 168
HvirOr3         ------------------------------------------------------------
TcasOr20        VITETDKSVKFYTKLFIVYGIVGNLCYGLLPILGYKKCHE-------------SKSVHMT 155
AgamOr11        IVGSNVRYLKRLAVVFWSCALVTANMMCVYSLVQYLTYE---------------EGPPDG 171
DmelOr56a       LLVATQAYTRTITLLIWIPSVIAGLMAYSDCIYRSLFLP--------------KSVFNVP 172
AgamOr28        TAKKSKIFVIIYTTSFLLICVEYSSMPLFKLIYHSAVYG----------------KQSNY 187
AgamOr58        -AARKALMDRVNRYLWLFYLQNIAQVFFWINLLRNCSP------------------LAVF 163
AgamOr59        IRDRWYRWSNGLILGPQCCIVLILLQTWISRQHLKKHT-----------------MLVVR 175
DmelOr1a        EWRSQNQRGQLMAAIYFMMCAGTSVSFLLMPVALTMLK----------------YHSTGE 159
DmelOr13a       NVAAECRRRMVTFSIMTSLLACLIIMYCVLPLVEIFFG----------------PAFDAQ 166
AmelOr161       IYAEKTMFAFRLAKYFITTVAITIVMFMSVPILEIYVIG-----------------NSDK 162
AmelOr141       IVQSYTRKTTRLTRWYLASCVLTIAFYAFLVLFGSLFDQ------------SKDFEHMRN 162
AgamOr52        LRQQAFRTNNVLMLALMVYGVLNFVVYEASGLHWQEIFR-----------------MPDY 144
AgamOr36        ---DTHRLSYRRIVNIAITFQLIGLADRLVAGFSQTYR------------------QELY 168
AgamOr25        -------KLISVTLHALAACYMSTMMFELIPIVSPNPR------------------KSNL 142
AgamOr45        KLFRAGTKRMLLAYRVIHLMYGTSYFFQLGPLIMPDP----------------HKCNLPL 163
TcasOr3         EKKEALKWTKYFLNFCNAICLSMFANGVFTFIFLSDKQ--------------YVERNGQR 175
DmelOr88a       NFWQRYRFIRIYSHLGGPMFCVVPLALFLLTHEGKDTP-----------------VAQHE 175
```

```
AgamOr22       ICGKYQRLARKLLLVTILSYLTTGAMLLIYPVVSGG--------------------LADR 164
DmelOr49a      NKYYLSCSTRNVLYVYYFVMVVMALEPLVQSCIMYLIG------------------FGKA 165
DmelOr85f      NDHYWTKSFVYLVIIYIGSSIMVVIGPIITSIIAYFTHN-----------------VFTY 165
AmelOr119      VKKIIDKYHTRNKVMFFGFCVIGACLGFALLCFTPMEN----------------GLPIRA 178
DmelOr63a      TMNRYWASTRRQILIYLYSCICITTNYFINSFVINLYRYFTK-----------PKGSYDI 187
DmelOr67b      ILLNNWMIIDRQVMFFFKIVCMPVLYYCVRPYFQYIFDCYIK-----------DKDTCEM 188
BmorOr21       AASSIRNCQIYCELGWTLIVMSCGLSFPVIAILLNIHS--------------FTFKFDST 166
BmorOr11       TVLNYAKKGVIVSKFWLFTAFAITFCFPLKAFIIMGYR-----------------FFIKN 162
BmorOr12       IEKSMVNKIKSYLSTYVFASITILVSNGIIAFFQTINS---------------DEPFLGI 170
AgamOr33       YPACHLSLQHGHHAWLRVCVRLSGLSDRLRTVQHGADR---------------------- 153
HvirOr1        MDPNFDCSLTRKCNITCAVVLILALLEHILSLLSAFAG---------------ASACYTG 184
HvirOr5        --------YVLVSHGFIFMGQDYSEWFAMPLVIIS------------------------- 125
TcasOr1        -------LVYCSLAIFVLCELLIEGTTSILINVPS------------------------- 131
TcasOr2        VNYKKKKFQRMVVARYVYYATIFTIITATSSFVTIHN----------------------- 161


TcasOr16       KAWYPWDAMSGMPYYITLVFQIYYVFFSLAHANLLDSLFCSWLIFACEQLQHLKEIMKPL 242
AmelOr2        RSWYPYDPSHGMAHILTLIFQFYWLIFCMADANLLDVLFCSWLLFACEQIQHLKNIMKPL 239
AgamOr7        KSWYPWNAMSGPAYIFSFIYQIYFLLFSMVQSNLADVMFCSWLLLACEQLQHLKGIMRSL 239
AaegOr7        KAWYPWDAMSGVPY-FSFIYQAYFLLFSMCQANLADVMFCSWLLFTCEQLQHLKGIMRPL 223
DmelOr83b      KSFYPWNASHGMFYMISFAFQIYYVLFSMIHSNLCDVMFCSWLIFACEQLQHLKGIMKPL 241
BmorOr2        KAWYPFKTMSGGGYVFAFIYQIYFLLFSMALANLLDVIFCSWLIFACEQLQHLKAIMKPL 242
BmorOr2a       KAWYPFKTMSGGGYVFAFIYQIYFLLFSMALANLLDVIFCSWLIFACEQLQHLKAIMKPL 242
HvirOr2        KAWYPFNAMSGTMYIVAFAFQVYWLLFSMAIANLMDVMFCSWLIFACEQLQHLKAIMKPL 242
AgamOr56       FIVVKYAVTGSYDVPLSTPIEAKYFIPGLRTN------FWVWLPVDLTLNAILELHGFAL 205
AgamOr57       FIVVKYAVTGSYDVPLSTPIEANYFIPGLRTN------FWVWLPVNITLSAMLELHGFAL 205
AgamOr26       FIVVKYAVTGSYDVPLSTPIEGKYSIPGFRTN------FWVWLPFNLAQNVVLQCHSFAV 205
AgamOr27       FIVVKYAVTGSYDVPLSTPIEAKYFIPGLHTN------FWVWLPVTLGLNVLLEWHSLAL 205
HvirOr9        IIIPHYFKT--NEVKLEMPFIAYYPFNEFDS-RIYPWVYLHQVWTACVAMIMVYGPDCFF 217
HvirOr7        MIAVKYYKT--NEFEMLLPYLDVFSFIPYEL-KYWPFAYIHQIWSECVVLLDMAAADYLF 193
DmelOr85b      EYWVYDKWLNIRVVGKQLPYLMYIPWKWQDN-WSYYPLLFSQNFAGYTSAAGQISTDVLL 207
DmelOr85c      QFLVYEKLLKIRVVGQTLPYLMYFPWNWHEN-WTYYVLLFCQNFAGHTSASGQISTDLLL 206
DmelOr85d      YYLVCDFWLGMRQFERMLPYYCWVPWDWSTG-YSYYFMYISQNIGGQACLSGQLAADMLM 228
DmelOr67a      IYFIQRVLLHYPDAKQIMPFYQLEPWEFRDS-WLFYPSYFHQSSAGYTATCGSIAGDLMI 220
DmelOr67c      KASVKFNFLGYDTFDRNFGFLIWFPFDATRNNLIYWIMYWDIAHGAYLAGIAFLCADLLL 220
DmelOr92a      KSTIKYYLMGSEIFERNYGFHILFPYDAETDLTVYWFSYWGLAHCAYVAGVSYVCVDLLL 225
DmelOr69a      VLLWEHLHEE-YDLSFKTQTNTWFPWKVHGSALGFGMAVLSITVGSFVGVGFSIVTQNLI 215
DmelOr69b      LMIREHFSNS-QQLGYRIQSNTWYPWQVQGSIPGFFAAVACQIFSCQTNMCVNMFIQFLI 215
TcasOr6        MVYYGEKESRSQIYDNFVFTLQVIYLYVGCFVVAGFDCFFFYLLGHAVTELKMLTISFSC 189
TcasOr11       AIAFGESKGLSPKVDHLYFVLHSTSTFVVLHAVTGFDRLFFFLIGHVLTELKLVKKSYRL 206
AgamOr41       AIYLLSGDRVLPYGVVLPFVDPDSLVGYELNYIYQVSCIIWTPPGLVASVCMLFGLVLNI 207
AgamOr42       ALYLLRGERYLPLGIVLPFTAETDDFGFWCTLVVQLAYMYTGPFGLIPSQNIYFAFVFNI 213
AgamOr43       AQYILKGERFLVYTIILPFTDPEITSHYLLNLVVQYYLLIVGLAGFSAAENVLILFVTSV 208
AgamOr44       IQYIIKRERVLVYAILLPFSDPEITSHYLLNLIVQYYLMIAGIGGFAAAESVLILFVTSV 208
AgamOr66       YMYFVVHVKVPIFPLYIPGINIYSAYGYGITNSFHMLIAVYGLFGALTSDIVFIMFVVHF 206
AgamOr67       YMYFVVHVKVPIFPLYIPGINIYSAYGYGITNSLHMVIAVYGLLGALTSDIAFIMFVVHF 206
AgamOr72       FYYFATGVVVPLFLYELPFFDLSSSLGYAVTMCFQINLLAIGVLGAILSDFVFFMYAMYA 207
AgamOr73       FYYFATGKVMPLFLYELPFFNVSSSLGYAVTMGFQINLLAIGVLGAILSDFMFFMYAMYA 207
AgamOr71       FYYFVTGVVMPLFVYELPFCDLSSSLGYAVTMCFQINLLVIGVLGAILSDFVFFMYAIYA 207
AgamOr74       FYCFATGKVMPLFLYELPFCDWSSSLGYAVTICFQINLLAIGVLGAILSDFVFFMYAMYA 207
AgamOr70       FAYNVNGVVMPLFLYELPYYDWSTTIGYVVNMMFQVNLLVIGTIGAMLFDFLYFMYAMYT 207
AgamOr69       IAYYTTGELMPLFMIELPYYGWTTTIGLAMNMFCQANILVIGTMGAIMSDFLFFMYAMYA 207
TcasOr9        NKNLPMVVWYPYDPNKTPYFHLTYIWEIFCISNLGLIYAVLDLVFPCIAIVLGQQFKILA 94
TcasOr18       NLEMPMPAWFPYDYNKSPVFEITYLWQVFCLITLAIIYGASDMFFPCITIIIGQQFKILA 199
DmelOr83a      DRSLPLACWYPFDYTQPGVYEVVFLLQAMGQIQVAASFASSSGLHMVLCVLISGQYDVLF 232
DmelOr85e      IRMLPLQCWYPFDALGPGTYTAVYATQLFGQIMVGMTFGFGGSLFVTLSLLLLGQFDVLY 241
TcasOr24       PSCKKS----RRINEVCGVAIPYWVWFDTENWSIKLPLILHTFLVIIIVDKVTLLVSLQV 205
TcasOr25       DKCKEA----RRINEVCGSAIPFWTPFETEDLFTLTLVITYVLINIFVVVKVAMTVSVQV 205
TcasOr19       PHCKRN----RKIREVCGVFVPLWTPFDADYPPVLVLVASVVFIVVILIDKVTLLVSLQV 170
TcasOr22       PECRKIN-EEKGLSEICGMIVPFWAPFDIDWFPLKQIFWFDQLLGIYIIIKGGAAVSITT 202
TcasOr23       PQCRKMN-EEKGLSEICGMIVPFWAPFDIDWFPLKQIFWLNQLLGIYIIIKGGAAVSITT 202
AmelOr168      FQKDEFYHEDFVMPAVFSNQ-PFSTGVFISGVFLQLFGGEYLLFRKISLDLYTMHLNLLI 206
AmelOr170      FQKDEFYYEDFIMPAVFSKQQPFSNDVFICGIFLQLLGGEDTIIRKISLDIYTIYLCLLI 207
AmelOr169      FRKSEFYYTDYQVPAVISNE-PFPIGVFIGGVALQIFGSAYTLLRKVSLDLYTMHLILLI 208
DmelOr65b      AFPFQ-----------------WHEKSLHPISHAIIYLFQ--SYFAVYCLTWLLCIEGLS 200
DmelOr65c      AFPFQ-----------------WHEKSIHPISHAFIYLFQ--TWNVMYFLTWLVCIEGLS 200
DmelOr65a      SWPFQ-----------------LHDPSKHPIAYIIIFVSQ--STTMLYFLIWLGVVENMG 231
TcasOr17       AWYP--------------------YDSTKSPFYELTYIHQ--VVSIWYLVSASLNIDMLI 199
TcasOr21       GWYI--------------------VDTKVSPYYEIVYGFQ--FCSCCYMSALIINIDTLI 197
TcasOr56       TWMP--------------------FDYNDSPLYETIFAFQ--IFSTTVYGFYIGAADAVI 222
```

```
TcasOr15        TWTP--------------------FDYNDSPLYEIMFAFQ--IFSTTLYGFYIGAADAVI 220
AgamOr9         VWPR-------------------VDHTTPYWVVLMVILYQ--YMAIVLNASFNISWDSLV 214
AgamOr65        VWPV-------------------VDHTTPYWVYLMVIFHQ--YVAIALNASFNISWDSLV 218
BmorOr6         MWVP-------------------TDISK-TPNYEITFVVE--LWMMVISAALFMSVDTIT 194
BmorOr10        IWMP-------------------VSTGE-SPHYELGYLYQ--MITIYISAXLFISVDSVP 193
AgamOr34        PWFFG-----------------VPYWEDAHVAYYTICAYQ--SFGMYFHMLLNTAGDTQL 197
AgamOr37        PWILG-----------------IEYGPTKRLNYGLLFAYQ--VIGMYLHMLINVAIDVQL 209
DmelOr94a       YYVP--------------------FEWQNERRYWFAYGYD--MAGMTLTCISNITLDTLG 202
DmelOr94b       YYVP--------------------FEWRTRERYFYAWGYN--VVAMTLCCLSNILLDTLG 199
DmelOr71a       MWTP--------------------FDWQQPVLLWYAFIYQ--ATTIPIACACNVTMDAVN 195
DmelOr46a       TYNPL-----------------GENTGS--PAYWLLYCYQ--CLALSVSCITNIGFDSLC 195
DmelOr46b       ML-EV-----------------CSIEGW--ICYWSQYLFH--SICLLPTCVLNITYDSVA 194
HvirOr8         ICNYG-----------------FLSEDFRQMFGVPLYLYQ--GSAMMFDMLYSVNIDTLF 198
BmorOr8         ICNYY-----------------FLNEELRHDFFSGWYIYQ--SFGMYGHMMYNVNIDTFI 200
AmelOr68        AWLP------------------FNCSAPNYYYYYIAYVHQ--IIALIGTSLLNVACDVTI 195
AmelOr69        MWLP------------------YNYTS--LSAHIFIYTQQ--VVSLIVSAMIHVACDSFI 191
AmelOr70        AWLP------------------YDYS--ELLPFALSYAHQ--ATTSMFCSCQNISCDTLF 193
AmelOr71        TWLP------------------YDYS--TASAFLLAFAYQ--VVVATVCTFACVASDTLY 193
AmelOr72        AWIP------------------FNYTA-SWFLFSLTFIHQ--CGCAVVTSFGISIFDTLF 194
DmelOr19a       TWIP-------------------WNWRDSTSAYLATAMLH--TTALMANATLVLNLSSYP 198
DmelOr19b       TWIP-------------------WNWKDSTSAYLATAMLH--TTALMANATLVLNLSSYP 198
DmelOr33a       GWFP-------------------YDFQATAAIYWISFSYQ--AIGSSLLILENLANDSYP 193
DmelOr33b       AWFP-------------------YDVQATELIFWLSVTYQ--IAGVSLAILQNLANDSYP 195
DmelOr22a       PYI---------------------DSDE---QFYISSIAE--CFLMTEAIYMDLCTDVCP 208
DmelOr22b       PYV---------------------DPEK---QFYISSIAE--VILRGWAVFMDLCTDVCP 208
DmelOr42b       PFI---------------------DWHDGTLKLWVASTLE--YMVMSGAVLQDQLSDSYP 208
DmelOr59b       PFI---------------------DWRDGMGSLWIQAIFE--YITMSFAVLQDQLSDTYP 209
DmelOr59c       PLV---------------------DWRKGHWQLWIASILE--YCVVSIGTMQELMSDTYA 211
DmelOr98a       PFV---------------------DFRESRSSFWKAALNE--TALMLFAVTQTLMSDIYP 206
DmelOr42a       PFL---------------------DWRSSTLHLALQAGLE--YFAMAGACFQDVCVDCYP 211
DmelOr85a       PLV---------------------NPDD---HFYLATAIE--SVTMAGIILANLILDVYP 206
DmelOr43b       PFI---------------------NWRVDRTQMYIQSFLE--YFTVGYAIYVATATDSYP 208
AgamOr2         IPGVDVL---------------------ATPTYQVVFVLQ--VYLTFPACCMYIPFTSFY 188
AgamOr10        IPGVNNF---------------------DSPQYEIFYITQ--LVLTFPGCCMYIPYTSFF 189
DmelOr30a       LPTIDEYK-------------------YASPYYEIFFVIQ--AIMAPMGCCMYIPYTNMV 196
DmelOr49b       IPGLNEY---------------------ESPYYEMWYIFQ--MLITPMGCCMYIPYTSLI 188
DmelOr43a       LPGVSMT---------------------SSPVYEVIYLAQ--LPTPLLLSMMYMPFVSLF 192
AgamOr32        KVCFVTP-------------FRFPFDYQHPIVFALTFLHN--FDAMLVTVCTSVTVDSCF 196
AgamOr35        RVHFVAPMAYVSVRTRGRSRFHFPHNYQHPLMFALTFLFN--CDSIHMSIFIIGSVDTCF 208
DmelOr47a       HAEPELPFPC-----------LFPWNIHIIRNYVLSFIWS--AFASTGVVLPAVSLDTIF 200
DmelOr98b       NCSRNFHFP------------VYPWDNMKLSNYIISYFWN--VCAALGVALPTVCVDTLF 201
DmelOr9a        EIHLELPHNG-----------VYPYDLQVVMFYVPTYLWN--VMASYSAVTMALCVDSLL 212
AgamOr38        PLVKLLPFKV-----------VLPFDSQDPTLFVLTTIFL--NYASVPTITAMTSTDALF 218
AgamOr39        PLVKLLPFKL-----------ALPFDSQNLFPFVVTTLFL--NYASAPTITSQSGSDALF 219
DmelOr24a       DWIYETPFKM-----------MFPDLLLRLPLYPITYILV--HWHGYITVVCFVGADGFF 199
DmelOr45b       EFKFDMPFRM-----------LFHDFAHRMPWFPVFYLYS--TWSGQVTVYAFAGTDGFF 211
AmelOr74        ILPFK--------------MWVDLP-LSVTPYYEIMFVIQ--LLAVEQIGIAYVCSDFFL 202
AmelOr86        ILPFK--------------MWVDFP-LSETPYYEIMFVIQ--LLTVQQIGIAYTCNDNFL 202
AmelOr75        ALPFN--------------LWIDFP-VGLSPYFELLFILQ--ILCVYHVATCYICFDNLL 202
AmelOr84        ELPFN--------------LWVDLP-VGLSPYFEILFTVQ--ILCVYHVGVCYICFDNLL 200
AmelOr87        VLPFN--------------MWVDLPTLFLSPYYEILFVLQ--VLSLYHVGVCYICFDNLL 218
AmelOr76        ILPFN--------------MWLELP-LSITPYFEVMFFVQ--VVFVYHVCVCYHCFDSLL 202
AmelOr85        IHPFN--------------LWLDES-WYMTPYFEMVFIIE--ILSLYHVGVCYLYFDNFM 204
AmelOr88        IHPFN--------------MWFDRS-LSLSPYYEIIYTIQ--VLSAYEIGICYHCFDNLL 203
AmelOr91        VLPFN--------------MWINLP-LSMTPYFEITFTLQ--VLSLYQIGVSYFCFDNFL 201
AmelOr92        VLPFN--------------MWVNLP-LSMTPYFEISFVLZ--VLSLYQIAVSYFCFDNFL 198
AmelOr95        VLPFN--------------IWIDLP-LTITPYYEITFVLE--VISLYHIGVSYFCFDNFL 199
AmelOr93        ILPFN--------------MWVNLL-LNITSYFEITYTLQXFSLSLYHIGVSYFCFDNFL 189
AmelOr77        ALPFN--------------MWVNLP-LSTSPYYEIIFTIQ--VLSLYHIGVGYFCFDNLL 203
AmelOr94        ILIFN--------------MWLDLP-LSMSPYFEIIYVIQ--ALCLYQVGICYLCVDNMF 202
AmelOr96        ILIFN--------------MYLDLP-LSISPYYEIAYTIQ--IAALNQAGSCYFCFDNIF 202
AmelOr79        MLPFN--------------VWITFPVTR-TPYYEIIFFIQ--AICLYYIGISSFCFDNIF 202
AmelOr83        MFPFN--------------IWINLPITQRTPYYQIIFFVQ--GVSVYYIGISYFCFDNIF 203
AmelOr80        MLPFN--------------MWINLPISR-TPYYQITFLIQ--ATCVYYVGISYFCFDNIF 202
AmelOr81        MLPFN--------------MWINLPVSR-TPYYEIIFFIQ--AMCVYYVGISTFCFDNIF 202
AmelOr82        MLPFN--------------MWINLPLSK-TPYYEIMFLIQ--AMCVYYIGVASFCFDNIF 201
AmelOr78        MLPFN--------------MWLDMPLSK-TPYYEITFLIQ--AMCVYYIGISNFCFDTVF 202
AmelOr89        MLPFN--------------MWLKLPLSE-SPYYELMFVYQ--IMTFYFIGISYFCFDNIF 203
AmelOr90        ILPFN--------------MWVNLPLSQ-TPYYELLFLIQ--IITLYYIGICYFCFDNVF 200
AmelOr142       TFPTH--------------AIYPFS-VRRHLYKGLIFFHQ--SLVGFQVSSGMAIDTQI- 195
```

```
AmelOr146       PFPTN--------------AEYPFP-VHFEPVRSIVFVQQ--ALVGFQCSAHLCVNIFC- 195
AmelOr148       SFPWE--------------TEYPFQ-INDTSRN-IIYIHQ--FFFTYQCAAHICLSLFG- 192
AmelOr158       SFPWG--------------TEYPFQ-VNYTTINVIIYAHQ--FFLVYQCAAHTCLSLFG- 193
AmelOr147       TFPWE--------------TEYPFQ-VNYTSRNFIIYMHQ--FFFTYQCAAHICVSMFV- 193
AmelOr150       SFPAD--------------AEYPFQ-INYKSIKVIIYLQQ--TLVGFQCTAHICLSVFG- 195
AmelOr151       SFPID--------------AEYPFQ-INYTSVKIIIYLQQ--TLVGFQCAAHVCLSIFG- 195
AmelOr152       SFPAD--------------AEYPFQ-VNSTSMKIIIYLQQ--SLIAFQCAGHACLSIFG- 195
AmelOr154       SFPLE--------------AEYPFH-VNESLITIIIYMHQ--SLVSYQCSANVCVSIFG- 195
AmelOr155       SFPFD--------------AEYPFH-VNHSLVTIIIYIHQ--SLVGYQCSANVCASVFG- 195
AmelOr157       PFPFE--------------TEYPFH-VNTTTRIFIIYASH--VLVIFQGTAHMCLCMFG- 192
AmelOr156       SLPFY--------------TEFPLS-LNNTAVYIIIYFHQ--CFFAYQCSATVCLSIFG- 195
AmelOr153       SLSCD--------------AEYPFQ-LNYTLVFAIRYQS----FLSYQC-AYACADHX-- 181
AmelOr144       ELPAE--------------GWLPFS-IEPLGIYCVVYVNH--IYCILQT-SFCIFVDFTI 196
AmelOr145       ELPTE--------------GWLPFS-IEPLGIYCAVYVNH--VYCILQTLSFCIFVDFTI 197
AmelOr143       KLPAD--------------GWLPFS-TEPFGIYCIIYFNH--VVCILQT-AFCIFVDFTI 191
AmelOr1         NTTQI----PLPYLS----DYVFFVIEDSPI-YEMTFVLQ--IFISSIILSTNCGTYSLI 216
AmelOr3         NSTQI----PLPYLS----DYVFFVIEDSPT-YEITFVVQ--MFTSFLIMSLNYGIYSLI 221
AmelOr6         NVTIR----LLPCPT----YFTFFNELVSPY-YEMIFMLQ--ILAGVFVYTVLSGTIGIS 208
AmelOr7         NVTIR----LLPCPT----YFTFFNELVSPY-YEIIFMLQ--ILAGFFIYTVLSGTIGIS 208
AmelOr4         NVTIR----LLPCPT----YFTFFNELVSPY-YEMIFMLQ--LLARFFIYTVLNSTVGIS 210
AmelOr5         NVTIR----LLPCPT----YFTFFNELVSPY-YEMIFMLQ--LLAGFFSYTVLNGTVGIS 210
AmelOr8         NVTIR----LLPCPG----YFGSLDEQVTPN-YEIIFTLQ--VLGGFVTHTAVCGIKSAC 212
AmelOr9         NVTIR----LLPCPG----YFGSLNVQITPN-YEIIFTLQ--ILGGFVIYTALCGVKSSC 212
AmelOr11        NMTVR----LLPCPS----YFGSLNEQATPN-YEIIFTLQ--VLGGFIIYTVLCGTKSAC 210
AmelOr12        NVTIR----LLPCPG----YFGFLDEQVSPN-YEIIFTLQ--VLGGFVIYTAVCGTKSIC 212
AmelOr10        NMTIR----LLPCPG----YFGSLDEQVSPN-YEIIFILQ--VFGGFVIYTAVCSTKSIC 211
AmelOr14        NTTKR----LLPCPG----YFVFFNEQVSPI-YEIIFIIQ--VLGGLLTYTIMCGTIGMC 212
AmelOr15        NTTIR----LLPCPG----YYIFFNEQITPN-YEIVFTLQ--VIGGLLSYTIMCGTTSMC 213
AmelOr13        NTTVR----LLPCPG----YYMFLNEQVTPN-YEIIFTIQ--VLGGFLNYTTLCGTTGIT 204
AmelOr16        NITIR----PLPCPS----YFIIINEQQSPI-YEILFVLQ--VMAGMAIYAVISGTCGIS 215
AmelOr17        NTMIR----ALPCPS----YFFILNEQQSPI-YEILFVLQ--IIAGIAIYAVICGFCGIF 215
AmelOr18        NLTYR----RLVYPF----PKVLLDARHSPI-NEICYTIQ--LLSGFVAHNITVAACGLA 224
AmelOr19        NLTYR----RLVYPF----PKALLDARRTPA-NELLYTIQ--LLSGFVAHNITVAACGLA 224
AmelOr22        NITFI----PLVFPF----SRFIIDTRYSFI-NEIVFSIQ--LIAGALMHTITTAACSLA 225
AmelOr24        NITFI----PLVFPF----SRFIIDTRYSFT-NEIVFCIQ--LVAGVLLHTITTAACSLA 217
AmelOr20        NLTFV----PLVFPF----SRFIVDTRYSPT-NEIVFSIQ--LMAGALMHGITSAACSLV 221
AmelOr25        NLTFI----QLPFPS----SSLIADIRYSPY-NEIVLSVQ--ILTGVVMHAITSAACSIA 216
AmelOr29        NLTFI----QLPFPI----SILIADVRDSPY-NEIMLSIQ--ILTGIVMNAIRSAICSVA 217
AmelOr30        NLTFI----PLPFPS----SKLIADMRQSPA-NEILFSVQ--VLSGVIIHAITATAVSIA 217
AmelOr21        NISFI----PTMFPF----PKYIADVRYSPI-NEIVFFFQ--FMCGFLVHGVTSSACSLA 217
AmelOr23        NISFI----PTMFPF----PKYIADVRYSPI-NEIVFLAQ--FICGFLLHGITSSVCSLA 217
AmelOr35        NEIVR----SQAFPI----YRGLLDPRTSPS-FEIVQLMQ--CLAGFVIYSVTVGSCSLA 222
AmelOr27        TRVLH----VLPCAV----YKKLVNVEENSI-NIFMLCFQ--FVAAAIANSSTVGIFSLA 218
AmelOr28        TRIVH----VLPCAV----YRKLIDVTHSPN-SELIIASQ--FLSGFIVNSSTAGIFSLA 219
AmelOr33        TRIVH----MIPCIV----YKKLIATDTSPT-NEIVIASQ--FVSGFIVNSSAVGAVSIA 220
AmelOr34        TRIVH----MIPCVV----YKKLIASDTSPT-NEIVIASQ--FVSGFIVNSSAVGAVSIA 220
AmelOr32        TKTIH----MIPCTA----YKKLIAVDTSPT-NEIVIASQ--FLSGFIVNSSAVGAVSIA 219
AmelOr31        TRIVH----MLPCPV----YKELISIDTSPT-NEIVLISQ--FVSGFIVNSIAVGAISIG 219
AmelOr36        SMEIY----VLPSPT----YK--IPVDTNPG-HDIVLGFQ--YVAAYITSATVISAFSFA 217
AmelOr37        SMEIY----VLPSPI----YK--IPVDTNPG-HDIVLGFQ--YLAAYITSATVVSAFSFA 218
AmelOr38        SMEIY----VLPSPA----YK--IPVDTNPG-HDIVLCFQ--YLAAYITSATVVSAFSFA 218
AmelOr39        SMEIH----VLPSPT----YK--IPVDTNPG-YGIILGLQ--FLTGYIMSATVVIAFSFA 218
AmelOr47        SMEIY----VLPFPT----YK--IPVDTNPG-HAIILGLQ--YLTSFIMSATVVIAFSLA 217
AmelOr48        SMEMY----VLPFPT----YK--IPVDTNPG-HGIILSLQ--YLTSYTSSATVVIAFSLA 217
AmelOr40        SMEIH----VLPFPT----YK--IPVDTNLE-HSIVLGFQ--FLTGCIMSATVVIAFSLA 217
AmelOr43        SMEIY----VLPFPT----YK--IPVDTNPG-HNIVLGFQ--FLAAYIMSATVVIAFSLA 218
AmelOr45        SMEIY----VLPFPT----YK--IPVDVNPG-HNIVLGFQ--FLAGYITTGTVIIAFSFA 217
AmelOr41        SMEIH----VLPFPT----YK--IPVDTNLE-HGIVLGFQ--YLTACIMTATIIIAFSLA 217
AmelOr42        SMEIH----VLPFPT----YK--IPVDTNLE-HGIVLGFQ--YLTACIMTATIIIAFSLA 217
AmelOr44        SIRVY----ILPYPT----YK--VPVDTNPG-HSILLLLQ--FLTTCIMSTTVVIAFSLA 217
AmelOr46        TIEIY----SLLCPA----YK--LPVQTNPT-HDIILGTQ--LLSAFITSSSAAGAFSLA 217
AmelOr49        TVNIY----NLPCPA----YN--MPVDTNPT-HDIILGTQ--LLSAFICSSSTAGAFSFV 217
AmelOr50        TVELY----NLPCPA----YK--IPFDTDPTIHDIMLGTQ--FLSAFVVSSSASASFTLA 218
AmelOr26        SYSMY----RLPCPF----YTNLLDVRFSPM-NEIVFALQ--LLSGFISTSVTVGACGLA 223
AmelOr53        NYTIR----PLPYIG----YDPFFDTQSSPT-YEIVYCIH--CFTAMIMYSISTVAYSLT 224
AmelOr54        NYTIR----PLPYIG----YDPFFDTQSSPT-YEIVYCIH--CFTAMIMYSISTVAYSLA 222
AmelOr52        NNTFK----PLTYLG----YDPFFDTQSSPI-YEIVFCMH--CFAAMIMYSVTTVAYSLA 221
AmelOr51        NFTIR----PLTYPG----YEAFLNIQKSPT-YEIIYCMH--CIYVIVVGNITMAAYSLT 221
AmelOr55        NQTNK----VLIFPA----YS--NSIQKSPI-YEITYGIQ--CICGYVLDSVTSGACGLA 219
AmelOr57        NQTIK----MLIYPA----YGG-YNIQKSPT-YEIIYGVQ--CICEYVFDTIASGACGLA 220
```

```
AmelOr56        NQTIR----VLIYPT----YG--YNIQKSPI-YEIIYGVQ--FMCGYVVDTITSGACGLA 219
AmelOr58        NRTIK----LLIYPT----YSRLYDVQKSPV-YELVYILQ--CICGYMFDAVTVGACGLA 221
AmelOr59        NRTIK----PVIYPT----YNGLFNVQRSPI-YEFVYILH--CMCGYVMHSVTAGACGLT 222
AmelOr60        NHTIK----PVIYPT----YSGLFNVQKSPI-YELIYVLH--CTCGYVMYSITAGACGLA 222
AmelOr61        NRTIK----PVVYPT----YNALFDVQKSPI-YELVYLLH--SICGYIMYSVTAGSCGLT 222
AmelOr63        EESAM----KYPVPF----ENTLILINMPENMYFLIFIVE--YLMLLLTTTGNLGSDSLF 213
AmelOr64        EESAI----KYPMPS----ENTLTLINMPEKMYFVIFIVE--YLMLLLTSTGNLGSDSLF 209
AmelOr66        VESAM----KYPIPS----KNILAIINMPENMYFVVFIIE--YIMLLLTSTGNLGSDSLF 209
AmelOr65        EENAI----KYPIPS----KNALEIINMPDNLYFVVFIVE--YMMLLFTSIGNLGSDSVF 209
AmelOr67        EKSII----KYPIPS----KNALAIINMPENLSFMVFIVE--YMMLLFTSTGNLGSDSLF 209
AmelOr114       -----------LLFR----AYFPYDTTVSPN-YELTILGQ--FVAALYAATSYTAVDTFV 182
AmelOr115       GG---------LLHP----ATFPYETSKSPN-YEITWLGQ--LMGTVLTAICYSCFDTFL 201
AmelOr118       --------QGKFLVR----MEFPFIATISPR-YEIILITQ--FIFESFIVYGAATSIALI 188
AmelOr164       --------QKKFLLK----MEFPFEATVSPL-YEIIITIQ--LVMQFMFATMAGMFMTII 197
AmelOr163       TNNELILNNKKLLLK----MKLPFDFTIFPL-YEFVIVAQ--FVFECFVALTAGMLMAFS 202
AmelOr165       SI------SRELLFK----MDLPFETNESPN-YEFVVTSQ--LLIHVSAAFTFGTFSALL 195
AmelOr166       TS------SRELLLK----MNLPFDTNKSPI-YEFVVIIQ--YFYQVSAAFVFGVFAAFL 195
AmelOr167       NL------SPELLIK----MDLPFTYDESPI-YEYVFIVQ--FIQLFFIASSIAVLDALI 201
DmelOr35a       IFPFD---------------------SDPLYIFVPLLLTN--VWVGIVIDTMMFGETNLL 207
DmelOr74a       VYPFD---------------------NTQLHFFIPLLVLN--FWVGFIITSMLFGELNVM 208
AgamOr3         VE-----------HVLHLEEELYFLNIRTSMAHYTFYVAIMWPTIYTLGFTGGTKLLTI 228
AgamOr5         VE-----------HVLHLEEELYWFHTRVSLVDYSIFTAIMLPTIFMLAYFGGLKLLTI 209
AgamOr13        VH-----------YTLQMEENFYGLQTRTTTSHYLIFGMFMTPTIYLCAFTGTVKILSI 209
AgamOr15        MH-----------YTLQMEENFYGLQTRTSATHYLIFGIVMTPTAYLCAFTGTVKTLTI 209
AgamOr17        MH-----------FALQIEENFYGLQTRTTTSHYLLFGMIMTPTVYLCAFTGTVKTVTI 209
AgamOr16        PE-----------FTLHMEESFYRLNVREKLDHYLIFGAIMVPISFLCALVGTAKLVSI 207
AgamOr55        AQ-----------YVLHMEEDFYGLQIRSSLRSCLMFAIPMVPTSYVCAYVGCAKIMIV 209
AgamOr18        FA-----------FTLYLEQGFYWLDNRTSLLGYCVCTVFMLPLMYLCAYTATVKVVAV 214
AgamOr14        IRIWEISQVAPHRQFSLHMEQDFYGLQHRTNILHYTLYIAVIVPMMFVTACTVHMKVLTI 227
AgamOr51        VRTWDIS----HRQFSLHMEQSFYGLQHRTNILHYTLYIAVIVPMMFVTACTVHMKVLTI 218
AgamOr12        IQY-----------YPNLEESFYWLDNRTSVRGYAVLSVVAFITFIFAVYSQTTKLLTI 218
AgamOr19        IQY-----------YPNLEESFYWLDNRTSVRGYAVLSVVAFITFIFAVYSQTTKLLTI 218
AgamOr20        IQY-----------YPNLEESFYWLDNRSSVYGYATFSIVALLVFTVASYNNATKLLTI 218
AgamOr21        IRF-----------YPNLEGSFYGLDNRTSVYGYAAFSIVALLVFAFASYNNATKLLTI 215
AgamOr50        IQY-----------YPNLEESFYWLDNRSSVSGYAAFSAVALIVFAFASYNHVTKLLTI 217
AgamOr29        FII----------SSEMKCV-LYYLNIRFNLLHFSFYTIVICLLTITSAGSLCIKDIMD 209
AgamOr53        ---------------------LYDLDIRYNPLHYSIYAGLIFVLSAISSLSLCTKDVID 199
AgamOr30        YVM----------PTEMKSVTFYYLDIRFNLLHYSLYFGAVSGLSVIGSLALCTKDVMD 210
AgamOr46        LVM----------ENR-----FYVLDSHYNLGHCFVHQALMFFALYILLVMFTAKAGTL 206
AgamOr47        LIM----------ENR-----LYLIDSHYNLGHCFVHQALMFFALYILLVMFTAKAGTL 206
AgamOr48        FML----------PLMQD---LYGLDVHHNIVHYTISWVAITPFCVFCALILWFKGALF 215
AgamOr49        FLL----------PVESR---FFFLDIRHSIVHYTVFSLLACPAFLFTAYLTVVKGLVF 223
AgamOr61        IA----------------VQILYGLLSNVWGLIYVLSFAIFYIIMNTLHLEMSILLDGI 223
AgamOr62        PS----------------VQLAYGMVTACWGIIYVLSFIVFYTLLTGFRLEMQLLARSF 228
AgamOr63        WP----------------VQIVYGVLTMGFGGMYCMGFMMCYLLLSIFKLEVDILIRSL 225
AgamOr60        FL----------------IQLLYGWATCLWGSLYVLIFAFIYVLLNVFRGEMSLLVESF 221
AgamOr75        SER---------APVLERVADLCYSLVGYVWGWYHGATQLTIIVLLRYAITEFRVFLHSL 230
AgamOr76        SER---------APVLERVADLCYSLVGYVWGWYHGATQLTIIVLLRYAITEFRVFLHSL 230
AgamOr78        SER---------APVLERVANLCYSLVGYVWGWYHGATQLTIIVLLRYAITEFRVFLHSL 230
AgamOr77        FD-----------------ASHWPLVAKVAFRLYAYVAFAYAQLMLTFFGSYIITSSYL 208
AgamOr79        FD-----------------ASHWPLVAKVAFRLYAY--LAYAQLMLTFFGSYIITSSYL 206
AmelOr99        QQLLR------------------VNYIVFDIDDYFFYVYLQLAWGSIIVVLTIIAADWFY 214
AmelOr101       QQLLR------------------VNYIIFDTDDYFFYIYLQLAWGSIIVVLTIIAADWFY 214
AmelOr102       QQLLK------------------VNYLVFNDDDYFFYVYLQLAWGSIIVVVTSVAVDSLL 214
AmelOr103       QQLLK------------------VNYLFFNDDNYFFYVYLQLAWGSIMVVVTIVAVDSLL 214
AmelOr104       QQLFR------------------VNYLIFNHNDYFFYIYLQLAWGSIIVVMIIVTVDSLY 214
AmelOr98        QQLLR------------------VNYMVFNADDYFFYVYLQLAWGAIVIVMIVITVDSLY 213
AmelOr100       QQLYR------------------VNYFIFNHEDYFFYVYFQLVWSSFVCVIVIIIFDWLY 140
AmelOr97        FQIFK------------------MKYFVNE-DEYFYPIYFHSVWSSFVIIMITVTIDSLY 166
AmelOr105       QNIFN------------------VNYIILDNYEYFYIVYMHLSCSAVIIVIIIISVDSLY 214
AmelOr109       EFIYP------------------TYYFVDEQK-YYYPILTHMIAVILVLSSVYLACDTNL 201
AmelOr110       EFIYP------------------TYYFVDEQK-YYYPILMHMIAAILVLSSIYLACDTNL 201
AmelOr111       ELIYP------------------SYYFVDEEK-YYYPILMHMIAVALILTSVYVACDTYL 201
AmelOr108       EFIYP------------------TYFFIDEEK-YYYPILTYMATVILIVSSVYLACDTNL 201
AmelOr107       ELVYP------------------SYYFVDEQK-YYYLITGHMLAVCLGHVFVYIACDINL 201
AmelOr112       VFLYP------------------AYFWLDDEK-YYVLLLGHMIITLLMICFIFCACDTNY 201
AmelOr113       LYVIP------------------GYYFVDDDRDYYYFIQLHMSLSIIMVANVYVAYDTCY 202
AmelOr106       VQMYP------------------AYYYIENEADYYYPILIFSIVSLLTAMCVYIATDTTL 199
AmelOr122       YFDLL------------------FLLDDQSAYIKIFLILNFMLNILFGLLSITSTESFTN 202
AmelOr125       YFDLL------------------FFFDDQSAYIKIFLILNFMLNTLFGLLSITSTESLTN 201
```

```
AmelOr123      HYSVI------------------FLSNDRRKFIDIFLVLNSIIIFTFGLLSLICSELFTN 199
AmelOr126      HYSLI------------------FLSNDRRKCIDIFLVLNSIIIFTFGLLSLICSELLTN 199
AmelOr127      HYSVI------------------FLSNDRRKCIDIFLVLNSIIIFIFGLLSLICSELFTN 199
AmelOr128      HFSLI------------------FLSNDRRTYIDIFMVLNLIIILIFGLLSIVCSESLTN 199
AmelOr129      HFSLI------------------FLSNDRRTYIDIFMVLNLIIILIFGLLSLICSESLTN 199
AmelOr133      MLHYV------------------TLLSVNGTIYFYILCLDFLFIIIFGLLSMICTETIVG 102
AmelOr134      MLHYV------------------TLFSVNGTIYFYILCLDFLFVIIFGFLSIICTETIIG 198
AmelOr131      ILLYV------------------TLFSVNRTIYFYILYLNFLFVITFGLLSLICTESIVG 200
AmelOr132      IAHYI------------------TIFSDKRIIYVDILCLNYMFLMIFVVLSIMSTESILG 200
AmelOr135      IIHYI------------------TLFSVNRTMYFNILCLDFIFVVIFALLSVICTESIIG 198
AmelOr136      IVHYV------------------TIFSDKSIIYMDILCLNYMLLAILVILSATCTESILG 200
AmelOr138      IIHYI------------------TIFPYNRTIYLDILSLNFMFVGIFGSLSLACTESIFG 106
AmelOr130      FIYYI------------------TTFSHNQSIYLDILDFNFMFTGIFGLLSVACSESITG 200
AmelOr137      IIHYI------------------TIFPYNRTMYLDILSLNFMFVGIFGSLSLACTESIFG 199
AmelOr139      IIHYF------------------TIFFHSRIIYIDILCLNYIFLAIISLLSIICIESMIG 121
BmorOr20       NITYDLS------------IYYET-FDILNTPNGYIFICVFNWFASYICCSFFCSFDLIL 226
BmorOr3        NITYDLS------------IYYET-FDILNTPNGYIFICVFNWFASYICCSFFCSFDLIL 226
BmorOr18       NITYDLS------------IYYET-FDILNTPNGYIFICVFNWFASYICCSFFCSFDLIL 226
HvirOr6        NSTFEHS------------LYYLYPFNTSTDITGYIIACILHWIISYLCSCWFCIINLFL 232
BmorOr23       NVTLQQC------------VYYALPFDYTGNFKWYLLVAIFNVQKTFFCTSLFILFELSL 227
BmorOr16       NVTLQQC------------VYYALPFDYTGNFKWYLLVAIFNVQKTFFCTSLFILFELSL 227
BmorOr4        NVTLQQC------------VYYALPFDYTGNFKWYLLVAIFNVQKTFFCTSLFILFELSL 226
BmorOr5        NVTLQQC------------VYYALPFDYTGDFKWYMLVAIFNVQKTFFCTSLFILFDLLL 227
BmorOr22       NMTFQHA------------VYLGLPFDYTTDIKGYFVVFILNWHLSHIAASYFCTFDLFL 233
BmorOr17       NMTFQHA------------VYLGLPFDYTTDIKGYFVVFILNWHLSHIAASYFCTFDLFL 233
BmorOr1        NVTFFHS------------VYFAFPFDYSHNFRGYIIMALFNSYVSVTCSIGLVMFDLLM 229
BmorOr1a       NVTFFHS------------VYFAFPFDYSHNFRGYIIMALFNSYVSVTCSIGLVMFDLLM 229
BmorOr1b       NVTFFHS------------VYFAFPFDYSHNFRGYIIMALFNSYVSVTCSIGLVMFDLLM 229
BmorOr19       SMEFQYY------------IYFWTPLDYKYNLRDYIIIHTLCIYLGATAVTVIVTFDIFN 226
BmorOr15       SMEFQYY------------IYFWTPLDYKYNLRDYIIIHTLCIYLGATAVTVIVTFDIFN 226
TcasOr5        -------------------------ISTSESHSTVKLGWYCNFITEMFLFYFLIVLRSLY 188
TcasOr7        -----------------------HFLNNLENHNLEESVRVFFMLQDLAQSTFNIIEEFML 195
HvirOr4        ALA--------------------LTARQVSIPVEKPKTSSPLNIYAIRVAPVDSQRSANV 205
BmorOr13       GLR--------------------TLLHQSGIESLTEIPNSNEQHTANAILPQPPKKSVNN 254
DmelOr10a      VWFTP---------------FNMTMPKVLLNYPFFPLTYIFIAYTGYVTIFMFGGCDGFY 216
DmelOr33c      ELLYP----------------AYFPFDLESNRFLGAVALGYQVFSMLVEGFQGLGNDTYT 197
DmelOr7a       LYLPG--------------------LDWQRSQMQFCIQAWIEFLIMNWTCLHQASDDVYA 212
DmelOr22c      ELPFN-----------------IILPSFAVQPGVFPLTYVLLTASGACTVFAFSFVDGFF 211
AgamOr1        PVP------------------AWFPVDYHHSDIVYGVLFLYQTIGIVMSATYNFSTDTMF 206
AgamOr4        LEVK------------------FYWLENRTSVEDYITFVLIMLPVVVMCGYVCNLKVMTI 210
DmelOr47b      SVYP------------------FQWHRLDLHPYTFWFLYIWQSLTSQHNLMSILMVDMVG 227
AmelOr73       TLPVS-----------------DWIPYDLSSTELYIISLLYQTVGLLICANASVGNETLI 193
AgamOr68       FCEIP-------------YVDWTVMKGYWTTVILQLFFYITGTCGLILVDYLCAYFTING 207
DmelOr59a      MYP------------------AWFPFDWLHSTRNYYIANAYQIVGISFQLLQNYVSDCFP 197
DmelOr23a      ---------------------MWFPFDWKNSMVAYIGALVFQEIGYVFQIMQCFAADSFP 192
AgamOr31       KLP-----------------LLMDFPFDVKHPVTFTIFFVWCSVAIFWVVLDCVACDSTF 195
DmelOr2a       LYP------------------AWFGVDWMHSTRNYVLINIYQLFGLIVQAIQNCASDSYP 202
AmelOr62       DIPLKLN-----------YFLASYCVFGSLPLLHHVCVLLLQGIFIFVNAVAHCGNDGLF 218
AmelOr121      KKLFP---------------LPSYYIFDVSETPYFEIMYALQSISLLIAAFCYAGVDNFF 214
AmelOr140      ---------------------------TMYMLYAQHAYALFAIVSYELKTIHILNTNNLI 183
TcasOr12       PLSG-------------------WYPFSTEKSPAFEFAYTYQIFTTWVGGLGDISMDTFM 205
AgamOr8        HKQLP---------------YFMWYWYDWHRPGYFAVTFLHQNWGGFVSAVFYLSTDLMF 215
BmorOr9        QFVLP---------------YAVLLPFKTDSWIPWLIVYVYSIFCGFTCVLYYATVDVLY 202
AgamOr40       TLPFP-----------------CWYPVDVHESPMYELAYIFQVLGQLQVSLVFGLASALF 213
TcasOr63       PLPG------------------WFPYDTTNYYYSTFFVQS---LSLFISAYCNTAIDILT 198
TcasOr4        PLPG------------------WFPFDVKTHQIELVIAQT---CGIAIGAFLNSTLDILP 191
TcasOr13       ILSLP------------------YYVCYCFTMIIEIMFWQFVNSIKIRLILLNERLEQIG 215
AgamOr24       ILPFG----------------FVMPFIDANTLAGYYANYGIQLIMAIYYWIITVGSDITT 193
AgamOr54       ---------------------------------SLLWSIAVEYVLPFGFFIPTVGMDTLQ 190
AgamOr23       SLAFG----------------FYLPFVDYRTPVGFAINWVYQFVQVLEGCIGLMACDSCL 197
AgamOr64       VVG-----------------PLYFQIIVEIFTGYLSLGYFVPSCITLMTLQLFRTEMHIL 225
DmelOr82a      ELPMP-----------------MKFPFNDLESPGYEVCFLYTVLVTVVVVAYASAVDGLF 203
AmelOr116      PIAG-------------------WYPYNVTSTPIFEIACLHQVLVILINCINNIAIDTLI 208
AmelOr160      PFELP---------------MKVNNVYEITKLQTYALFCVYLIPTSTLLTIGATGADSLL 208
TcasOr10       -----------------------RQFLKAVKHHEFLLQYVEQLGDIYSMWFLLQYFSSLF 175
TcasOr26       TEVCG-------------VVSPVWLPFRFDYKPMKQFVYFWQSFCCLYSNGGAGTISFAM 191
AmelOr117      NRHLP---------------FIVWYGTDISASPSFEIVFFYQIISSSICASVISGLDTSL 211
AmelOr120      NFTIKN------------FPFAASYPITFYKFPFYEIAYISQILATSICCLMMLAIDSLI 201
BmorOr7        EFP-------------------FWIQIDYTKSSMFVVVLLYSYYVTTLVGIANTTMDAFM 197
AgamOr6        QLAFN----------------IWWPFDVLGGVKYYWFVYPLYVVIGFTGIIIHMAFDCLF 214
```

```
DmelOr45a       TPTTP---------------MEFNFWLDERKPHFYWPIYVWGVLGVAAAAWLAIATDTLF 200
DmelOr67d       QFLIP--------------------FLDHTTDGGHLILTAAHVILITFGGFGNYGGDMYL 208
DmelOr83c       QYLIPG--------------------LPLENNYCYVVTYMIQTVTMLVQGVGFYSGDLFV 203
AmelOr162       G---------------------CNGYRITPPQZKGZMVSKCQGLCTLAQTHNRVSIKLIT 207
HvirOr3         ----------------------------MESLMNQILALEVLAFCVFPGLCRAFAMQGLV 84
TcasOr20        RYGIPCG-----------LVVRFLFPFKFDYSPLAELVALYEILVCILGTSVVIVVTTLI 204
AgamOr11        TGTSAANITRKGQPIPPTPILRSWYPTADGKDKHFLEIYLIQLYIMYVGQLIVPSWHMFM 231
DmelOr56a       AVRRGEE----------HPILLFQLFPFGELCDNFVVGYLGPWYALGLGITAIPLWHTFI 222
AgamOr28        TIALP---------------YLSRFAYSTESTTSFAWTYFFILIGVYLLALTLSGFDSLF 232
AgamOr58        ELS----------------------LLDSVNVLLYPLAMTLMSLMFIHTIMIVSTLLSAL 201
AgamOr59        GEPVG------------------TEFDHFLYMSCLYFPTVGFFMGCSIVNAMLVGFLGEM 217
DmelOr1a        FAPVS--------------SFRVLLPYDVTQPHVYAMDCCLMVFVLSFFCCSTTGVDTLY 205
DmelOr13a       NKPFP--------------YKMIFPYDAQSSWIRYVMTYIFTSYAGICVVTTLFAEDTIL 212
AmelOr161       VLPFRG-----------------YFFINQTVSPIFEFLYLFNVTAGGFGGSMIAGATSFN 205
AmelOr141       DSSLVPSEAGNILESMSKRHLPYAFFLDVQKTPWYEIVYAVQLIGMFNVGFTCVGVDTVG 222
AgamOr52        LLATN---------------RPLTWTLHLIMRPMTFNGLGAFIATFVSIHTMLTVLHAEL 189
AgamOr36        EVPAN-----------------LAELQWPMVVAVHVLSFDFASRWGAAYNVSLTGMNSIM 211
AgamOr25        PLP------------------MYLPYMPHDVTPYWHLNYTFITVMNLMCILFLVGIDGLL 184
AgamOr45        ALQLP-------------------FLPPDRNMVYYCINYAHHLLLNTIGVFILLPMDGVL 204
TcasOr3         VYHQEQP-------------VNTVSPFGSGTKLRFFVTFIYTMIALTFYAWTIVALDSLF 222
DmelOr88a       QLLGG-----------------WLPCGVRKDPNFYLLVWSFDLMCTTCGVSFFVTFDNLF 218
AgamOr22        TLPLG----------------YSIPFADYRTHPWYLINYLLQIVQVQWVALVFVGLDGPF 208
DmelOr49a       DFTYKR-------------IFPTRLTFDSEKPLGYVLAYVIDFTYSQFIVNVSLGTDLWM 212
DmelOr85f       MHCYP----------------YFLYDPEKDPVWIYISIYALEWLHSTQMVISNIGADIWL 209
AmelOr119       KYPLN----------------TTVSPWHEISFFVETCAVSGGLLGIIVMDSMTTFKCSLI 222
DmelOr63a       MLPLP--------------SLYPAWEHKGLEFPYYHIQMYLETCSLYICGMCAVSFDGVF 233
DmelOr67b       TLTYP--------------AIVPYLQLGNYEFPSYVIRFFLLQSGPLWCFFAVFGFNSLF 234
BmorOr21        KHMIHDIN-----------NPFTDDPEDRFESPFFEIMFVYTFFSSFIYIINYVGYDGFF 215
BmorOr11        EFRLEP---------MFDMTYPEPIESYKTSFPVYFILFVVFFLFGCYASSLFVAFDPLV 213
BmorOr12        ITAWP--------------DKTDTSKTASYARIGFYLFWCIHFFRISTVFAVIVCILISI 216
AgamOr33        ----------------------LYRRASAQPDGATVLHAAQTVRADRGRSSAAPRGPPPR 191
HvirOr1         MDTYQG-------------FVTHFYPWVFNYLPYSIVLGVITQFLHFQSTFIWNFSDLFV 231
HvirOr5         -----------------------------TIATLLWNFQDQVIVLISMGLTSRYRRLNEC 156
TcasOr1         ------------------------LIIHVSTSAYVATVWINSVINRWKFIEFIRKVLEFD 167
TcasOr2         ----------------------------VIGQFLGYFIGLITFVMSQHCVELVSMIKARF 193


TcasOr16        MELSATLDTYVPKSADLFRAPSATSQDQ-------LIENDYNEKNEDLKGVYSTRQELGG 295
AmelOr2         MEFSATLDTVVPNSGELFKAGSAEQPKEQEPLPPVTPPQGENMLDMDLRGIYSNRTDFTT 299
AgamOr7         MELSASLDTYRPNSSQLFRAISAGSKSE----LIINEEKDPDVKDFDLSGIYSSKADWGA 295
AaegOr7         MELSATLDTYRPNSAALFRVASAGSKSE----LILNEEKDPDTKDFDLNGIYNSKADWGA 279
DmelOr83b       MELSASLDTYRPNSAALFRSLSANSKSE----LIHNEEKDPGT-DMDMSGIYSSKADWGA 296
BmorOr2         MELSAALDTYRPNTAELFRVSSTDKT-----------EKVPDAVDMDIRGIYSTQQDFGM 291
BmorOr2a        MELSAALDTYRPNTAELFRVSSTDKT-----------EKVPDAVDMDIRGIYSTQQDFGM 291
HvirOr2         MELSASLDTYRPNTAELFRASSTEK------------EKIPDTVDMDIRGIYSTQQDFGM 290
AgamOr56        VFVETFTWNLVYATSCMFRILQIQVN--------------------ELSHQSRNEKEWNG 245
AgamOr57        FFVETFTWNLVYATSCLFRILQIQAN--------------------ELSHQCRNEKEWNG 245
AgamOr26        ITIECFTWNLVYATSCMFRILQIQAT--------------------ELLDRSRDKKEWNI 245
AgamOr27        VFVETFIWNLVYATSCMFRILQILAN--------------------ELSDRKRNEGIWTV 245
HvirOr9         FTCCTFIHIQFSLLNNDMERIVNEDTPR--------------------YD--------KT 249
HvirOr7         FTCCTYIRVQFKLLQYDFERMIPDRGIS--------------------KGKFYEENELRN 233
DmelOr85b       CAVATQLVMHFDFLSNSMERHELSG----------------------------DWKKDSR 239
DmelOr85c       CAVATQVVMHFDYLARVVEKQVLDR----------------------------DWSENSR 238
DmelOr85d       CALVTLVVMHFIRLSAHIESHVAGIG---------------------------SFQHDLE 261
DmelOr67a       FAVVLQVIMHYERLAKVLREFKIQAHNA--------------------PN---GAKEDIR 257
DmelOr67c       VVVITQICMHFNYISMRLEDHPCNSN---------------------------EDKENIE 253
DmelOr92a       IATITQLTMHFNFIANDLEAYEGGDH---------------------------TDEENIK 258
DmelOr69a       CLLTFQLKLHYDGISSQLVSLDCRRP------------------------------GAHK 245
DmelOr69b       NFFGIQLEIHFDGLARQLETIDARNP------------------------------HAKD 245
TcasOr6         KEIGRNWGYEERFKCSVKHHIHVLELLDKI------------------------------ 219
TcasOr11        TQNRR-----EKFLETVQHHAFALEIPPDL------------------------------ 231
AgamOr41        CIQYDILAVKLQDLDELIRSPHPHRDAM-------------------------------- 235
AgamOr42        CLQYELLIERLKQLDERIRSSN-NLKLT-------------------------------- 240
AgamOr43        AGYADVLKNKINEMNTLLLDAQNSRDR--------------------------------- 235
AgamOr44        AGYADVLKNKIDEMNIILLDAQNSRDR--------------------------------- 235
AgamOr66        VTYGGLFKIECEQFDQDLSGVFQHCEWRTA------------------------------ 236
AgamOr67        VTYGGLFKIECEQFDQDLSGVLQHCEWRTV------------------------------ 236
AgamOr72        MARADISIVHLRELENILNNPSKNE----------------------------------- 232
AgamOr73        MARADISIVHLSELENILNDSTKNE----------------------------------- 232
AgamOr71        MARTEISILHLGELENMLIDPNKNE----------------------------------- 232
```

```
AgamOr74        MARADISIVHLGELKNMLNDPTKNE----------------------------------- 232
AgamOr70        MVKADIFIIHLGELEKMLNDPLTMK----------------------------------- 232
AgamOr69        MTCIDIFIVHLCELETLLKEIHIHEKE-CA------------------------------ 236
TcasOr9         SNFKNNVYRALVDSEVSEKIVQTFSKNLHN------------------------------ 124
TcasOr18        SNFKNNFYTSLIKLGAEESIVQNFSKDIKT------------------------------ 229
DmelOr83a       CSLKNVLASSYVLMGANMTELNQLQAEQSAA----------------------------- 263
DmelOr85e       CSLKNLDAHTKLLGGESVNGLSSLQEELLLG----------------------------- 272
TcasOr24        LEIACNIKLRLDQLNCMLVSCFDG-DVEAS------------------------------ 234
TcasOr25        LEISSHINLRIEQLKIFIAGCFDR-DFKAS------------------------------ 234
TcasOr19        LEISCHIRLRIEQLNAMILNCFNG-DLQSS------------------------------ 199
TcasOr22        FEVAQYICLKIKHLNSLLRQAFDDPHEEVI------------------------------ 232
TcasOr23        FEVAQYICLKIKHLNRLLREAFDDPCDVVV------------------------------ 232
AmelOr168       TSQYKYLRIKFATILKENGESAKDNDK--------------------------------- 233
AmelOr170       TAQYKYLRIKFAIILKEEREITKDHYKNIIW----------------------------- 238
AmelOr169       TAQYKYLRIKFAAILEQ--ETPKDFFYGGII----------------------------- 237
DmelOr65b       ICIYAEITFGIEVLCLELRQIHRHNYG--------------------------------- 227
DmelOr65c       VSIYVEITFAIEVLCLELRHLHQRCHG--------------------------------- 227
DmelOr65a       VSLFFELTSALRVLCIELRNLQELCLGD-------------------------------- 259
TcasOr17        AALNMFVGAQCDLLCDNLRNIGQ------------------------------------- 222
TcasOr21        AALNVYIGNQIDILCNNLRNLKA------------------------------------- 220
TcasOr56        CGFMMLIKAQFLIVKRELETLIERAQKAAIAENPDNEDNFGRE----------------- 265
TcasOr15        CGFLMLIKAQFLIVKRELETLVERAQRAG---NPDRGD-FGGG----------------- 259
AgamOr9         AALLALTNAHLHRLQIQLLKVDANKRMITVSPIDDDAR---------------------- 252
AgamOr65        AALFALTNAHLHRLQIQLMK-DAKRRMIAVSSFDDNAR---------------------- 255
BmorOr6         VATMMFSCAQLDIIMKKTQQ-------IQEIPLSPELS---------------------- 225
BmorOr10        LSMIMFGCAQLEIIMDKIGK-------VKSWSLDQQPM---------------------- 224
AgamOr34        CYMMHMIGIQLELLGKRFRDLN------NCE----------------------------- 222
AgamOr37        SYLLGMISIQLDLIGKRFRSLH------TSE----------------------------- 234
DmelOr94a       CYFLFHISLLYRLLGLRLRETKNMK---NDT----------------------------- 230
DmelOr94b       CYFMFHIASLFRLLGMRLEALKNAA---EEK----------------------------- 227
DmelOr71a       WYLMLHLSLCLRMLGQRLSKLQ--H---DDK----------------------------- 221
DmelOr46a       SSLFIFLKCQLDILAVRLDKIGRLITTSGG------------------------------ 225
DmelOr46b       YSLLCFLKVQLQMLVLRLEKLGPVIEPQDNE----------------------------- 225
HvirOr8         AGLMVLAIAQLDILGIKLRRVTDKEVLEETDSE----T---------------------- 232
BmorOr8         SGLLMMAVTQLKIIQTKLLSLK----LNPRERK----M---------------------- 230
AmelOr68        CGLFVHMYSQQEILKHRLKESV-------------------------------------- 217
AmelOr69        WALLMHICSQIEIFNCRLRKIK-------------------------------------- 213
AmelOr70        AGFLVQIYCQFEILEERLKNVQ-------------------------------------- 215
AmelOr71        SGLLIHISCQFEILEHRLKNIG-------------------------------------- 215
AmelOr72        AGLLLQVCCQLDTLVYRLQNIK-------------------------------------- 216
DmelOr19a       GTYLILVSVHTKALALRVSKLGYGAPLPAVR----------------------------- 229
DmelOr19b       GTYLILVSVHTKALALRVSKLGYGAPLPAVR----------------------------- 229
DmelOr33a       PITFCVVSGHVRLLIMRLSRIGHDVKLSSSE----------------------------- 224
DmelOr33b       PMTFCVVAGHVRLLAMRLSRIGQGPEETIYL----------------------------- 226
DmelOr22a       LISMLMARCHISLLKQRLRNLRSKPGRTEDE----------------------------- 239
DmelOr22b       LISMVIARCHITLLKQRLRNLRSEPGRTEDE----------------------------- 239
DmelOr42b       LIYTLILRAHLDMLRERIRRLRSDENLSEAE----------------------------- 239
DmelOr59b       LMFTIMFRAHMEVLKDHVRSLRMDPERSEAD----------------------------- 240
DmelOr59c       IVFISLFRCHLAILRDRIANLRQDPKLSEME----------------------------- 242
DmelOr98a       LLYGLILRVHLKLLRLRVESLCTDSGKSDAE----------------------------- 237
DmelOr42a       VNFVLVLRAHMSIFAERLRRLGTYPYESQEQ----------------------------- 242
DmelOr85a       IIYVVVLRIHMELLSERIKTLRTDVEKGDDQ----------------------------- 237
DmelOr43b       VIYVAALRTHILLLKDRIIYLGDPSNEGSSDPSY-------------------------- 242
AgamOr2         ATCTLFALVQIAALKQRLGRLGRHSGTMAST----------------------------- 219
AgamOr10        ASSTLFGLVQIKTLQHQLKTFR--SSEMLN------------------------------ 217
DmelOr30a       VTFTLFAILMCRVLQHKLRSLEKLK----------------------------------- 221
DmelOr49b       VGLIMFGIVRCKALQHRLRQVALKHPYGDR------------------------------ 218
DmelOr43a       AGLAIFGKAMLQILVHRLGQIGGEEQ---------------------------------- 218
AgamOr32        SEMASNLTIHFDIVRERFEKL--------------------------------------- 217
AgamOr35        SELATSVTIHFQLIQRQFQAV--------------------------------------- 229
DmelOr47a       CSFTSNLCAFFKIAQYKVVRF--------------------------------------- 221
DmelOr98b       CSLSHNLCALFQIARHKMMHF--------------------------------------- 222
DmelOr9a        FFFTYNVCAIFKIAKHRMIHL--------------------------------------- 233
AgamOr38        SGVCLYMDGQFQAIRLELEALAETVDKY-------TLK---------------------- 249
AgamOr39        SGVCLYIYGQFQAIRLEVEALAETVDRN-------TLK---------------------- 250
DmelOr24a       LGFCLYFTVLLLCLQDDVCDLLEVENI--------EKS---------------------- 229
DmelOr45b       FGFTLYMAFLLQALRYDIQDALKPI-----------RD---------------------- 238
AmelOr74        CILNLHALYQFRMMQQELSKIWSAIEQQ--------TT---------------------- 232
AmelOr86        CVLNMHVVCQFRILQHRLTKLWSIIDER--------AD---------------------- 232
AmelOr75        CIVNLHVAGQFRILQHRLKNLG---------NAIRDET---------------------- 231
```

```
AmelOr84        CIVNLHVAGQFRILQHRLRNLN---------VAVTGDR---------------------- 229
AmelOr87        CLMNLHAATQFRILQHRLSDLGSGWDTRRSFNKIDRET---------------------- 256
AmelOr76        CILNLHTASQFRILQHRFANTCNEKRG---K---RDED---------------------- 234
AmelOr85        CIINLHIAGQFRILQHRFSNVCNEMCE---KCCYQLSR---------------------- 239
AmelOr88        FVINLYTAGQFRILRYRFENICGKNDD---KNYYKVSK---------------------- 238
AmelOr91        CIMNLHLAGQFKVLQYRISTIADRVIEK-EEKKEKLII---------------------- 238
AmelOr92        CIMNFHVAGQFKVLQHRISTIADLTIKT-EEKKEKLII---------------------- 235
AmelOr95        CIMNLHVAGQFQVLQYRISNIIDSIDK---EKKEKLIM---------------------- 234
AmelOr93        CIMNLHVAGQFQVLQYRISNIIYXRFN---EKKEKLIV---------------------- 224
AmelOr77        CVLNLQLAGQFQILQYKMANIVDLLKE----KNEKRII---------------------- 237
AmelOr94        CIMCLHLASQFRILQYRLANVSNVEDE-------EGVE---------------------- 233
AmelOr96        CILCLNVACQFRILQYRIANVPILKMK-------GNPD---------------------- 233
AmelOr79        CIMAVHLAGQFRILRYRLTKLC--EQEI---YEKDS----------------------- 233
AmelOr83        CIMAVHLAGQFRILRYRLMTLCDTEPET---REKDSRS---------------------- 238
AmelOr80        CIMAVHLAGQFRILRYRFTKLCDMEYGI---KENSQS----------------------- 236
AmelOr81        CIMAVHLAGQFRILRYRFTKLCDVEY------ENSQS----------------------- 233
AmelOr82        CIMAVHLAGQFRILQYRLTKLYDVECIE---MHKKDS----------------------- 235
AmelOr78        CIMAVHLAGQFRILQYRFTKLCDTDNQI---CKKNL------------------------ 235
AmelOr89        CIMTVHLAGQFQILRHRFDRLCNAEDRI---AEKG------------------------- 235
AmelOr90        CIMAIHLTGQFRILGYRFAKLCNIEHEM---REKDTV----------------------- 234
AmelOr142       ALLLRYATARFEILGIQFNNAKS------------------------------------- 218
AmelOr146       ALLLLFAAARFEILMNELRAVEN------------------------------------- 218
AmelOr148       ALLLWFAAARFECLVEELQKITN------------------------------------- 215
AmelOr158       ALLLWFATARFECLIKELQKITS------------------------------------- 216
AmelOr147       ALLLWFTSARFECLVKELQKTTN------------------------------------- 216
AmelOr150       ALLLWFTAARFECLIVELKKITN------------------------------------- 218
AmelOr151       ALLLWFTAARFECLAVELQKITN------------------------------------- 218
AmelOr152       ALLLWFVSARFECLAVELQKTTD------------------------------------- 218
AmelOr154       ALLLWFTVARFECLIEEFQKCSN------------------------------------- 218
AmelOr155       ALLLWFTVARFECLIVEFQKCTD------------------------------------- 218
AmelOr157       ALLLWFTTARFECLIGELRGVTS------------------------------------- 215
AmelOr156       ALLLWFVVIKFECLIMKIQNISN------------------------------------- 218
AmelOr153       --LLWFTAPRFECLCVELQNVTN------------------------------------- 202
AmelOr144       VILFSFPAAKLDVLRSKLRHVNN------------------------------------- 219
AmelOr145       VILFSFPAAKLDVLGSKLQNVNN------------------------------------- 220
AmelOr143       VILFSFPAAKLDVLRSKLRHVNN------------------------------------- 214
AmelOr1         ASITMHCCGLFEVTNRKIKTLCKWN----------------------------------- 241
AmelOr3         ASITMHCCGLFEVTNRRIETILK------------------------------------- 244
AmelOr6         LMLSLHMCSLLKILRRKMIDLADGSIT--------------------------------- 235
AmelOr7         LMLSLHMCSLLKILRRKMIDLADGSIT--------------------------------- 235
AmelOr4         LMLSLHMCSLLKILTRKMADLTDGSII--------------------------------- 237
AmelOr5         LMLSLHMCSLLKILTRKMANLTDRSIT--------------------------------- 237
AmelOr8         LMVCMHMCGLLRILTNKLTDLTNDND---------------------------------- 238
AmelOr9         LMLCMHMCGLLRILTNKVMELTSDKD---------------------------------- 238
AmelOr11        LMLCLHMCGLLKILTNKVMDLTNDSD---------------------------------- 236
AmelOr12        LMLCLHMCGLLKILTNKVMELTNDKD---------------------------------- 238
AmelOr10        LMLCMHMCGLLRILTNKVMELTNDND---------------------------------- 237
AmelOr14        VMFCLHSSSLLRILLNKIYQLTKQLDV--------------------------------- 239
AmelOr15        AMLCLHATSLLRILVKKINELTKQPDI--------------------------------- 240
AmelOr13        TMLCLHMCSLLEILINKMNDLTCQSDE--------------------------------- 231
AmelOr16        ALLVLHACSMLRILVNKIKKLVNKSDM--------------------------------- 242
AmelOr17        ALLVLHAWSMLRILVNKIKKLVDKSDM--------------------------------- 242
AmelOr18        ALLAIHACGQLQILMSWLEKLVDGRK---------------------------------- 250
AmelOr19        ALLAMHACGQLQILMSWLEKLVDGRE---------------------------------- 250
AmelOr22        AIFAVHACGQMQVLSNWLKHLINGRS---------------------------------- 251
AmelOr24        AIFAVHACGQMQVLSSWLKHLINGRS---------------------------------- 243
AmelOr20        ATFAVHACGQMQVLMNWLQHLIDGRL---------------------------------- 247
AmelOr25        AVFAVHACGQMQVLMNWLDHLVDGRS---------------------------------- 242
AmelOr29        AVFAIHACGQMQVLMNWLNHLVEGRS---------------------------------- 243
AmelOr30        AVFAVHACGQMQMLMNWLECLVDGRS---------------------------------- 243
AmelOr21        AIFTVHACGQIQVMMIWLEHLIEGRL---------------------------------- 243
AmelOr23        AILTVHACGQIQVMMVWLKHLIDGRL---------------------------------- 243
AmelOr35        AVFVMHACGQFQILVTKLRRLIDGLKED-------------------------------- 250
AmelOr27        AVLAAHAYGQLSVVMVWITEFVNQSR---------------------------------- 244
AmelOr28        AILGAHACGQLSVVMTWITEFVNKSK---------------------------------- 245
AmelOr33        AVFAAHACGQINLLMAWIRQLVNHSN---------------------------------- 246
AmelOr34        AVFTAHACGQVSLLMAWIRQFVDHSN---------------------------------- 246
AmelOr32        AVFAAHACGQLSLLMVWIREFVDHSK---------------------------------- 245
AmelOr31        AVFTAHACGQLTIIKRWIREYINRSK---------------------------------- 245
AmelOr36        TVFACHASGQLTIMIIWIEEFINRS----------------------------------- 242
```

```
AmelOr37        TVFACHASGQLTIMIIWIKEFINRP----------------------------------- 243
AmelOr38        IVFACHASGQLTIMIIWIEEFINRP----------------------------------- 243
AmelOr39        TVFACHTIGQLTIMVTWIEEFINRP----------------------------------- 243
AmelOr47        TVFACHAIGQLTIMISWIQEFVNQP----------------------------------- 242
AmelOr48        TVFAYHAIGQLTIMISWIQEFVNQP----------------------------------- 242
AmelOr40        TVFACHAAGQLTIMVTWIKEFVNRP----------------------------------- 242
AmelOr43        TVFACHAIGQLTIMITWIEEFVNRP----------------------------------- 243
AmelOr45        TVFACHAVGQLTIMITWIEEFVNRP----------------------------------- 242
AmelOr41        TVFACHAVGQLTIMVTWIEEFVNRP----------------------------------- 242
AmelOr42        TVFACHAVGQLTIMVTWIEEFVNRP----------------------------------- 242
AmelOr44        TVFAYHAVGQLTIMVTWIEEFVNRP----------------------------------- 242
AmelOr46        AVFASHALGQLNIMVAWINEFVNRP----------------------------------- 242
AmelOr49        AICASHALGQLNLMVIWINEYVNRP----------------------------------- 242
AmelOr50        TIFTCHVLGQLNIMMIWINEFVDRLQ---------------------------------- 244
AmelOr26        AVLAMHACGQFNVVMIRSDKLVKDNNE--------------------------------- 250
AmelOr53        TIFVTHICGQIQVQIARLQDLVESK----------------------------------- 249
AmelOr54        AIFVTHICGQIQIQIARLQKLVECK----------------------------------- 247
AmelOr52        AIFVTHICGQIQIQATRLQNLVENK----------------------------------- 246
AmelOr51        TIFITHACGQIKIQMLRLENLKNEK----------------------------------- 246
AmelOr55        ALFVTHACGQIDVVISRLDDLVAGQF---------------------------------- 245
AmelOr57        ALFVTHACGQIDVIMSRLDDIVAGQ----------------------------------- 245
AmelOr56        ALFVTHACGQIDIITSRLDDIVAGQF---------------------------------- 245
AmelOr58        ALFATHICGQIDIVMAKLEDLVDGKF---------------------------------- 247
AmelOr59        ALFATHACGQIDIVIARLNDLIHGKY---------------------------------- 248
AmelOr60        ALFVTHACGQIDIVIARLNDLVHAKY---------------------------------- 248
AmelOr61        ALFATHACGQIDIVIARLNDLIHGKY---------------------------------- 248
AmelOr63        FSIVFHLCGQVEILRLEYNKLSNENE---------------------------------- 239
AmelOr64        FGIAFHLCGQVEILRLEYNKLSNENE---------------------------------- 235
AmelOr66        FGIAFHLCGQVEILRLKYNKLSNENE---------------------------------- 235
AmelOr65        FGIVFHLCGQVEVLKREYSKLFNKNE---------------------------------- 235
AmelOr67        FGIVFHLCGQVEILKLKYNKLSNTNE---------------------------------- 235
AmelOr114       AMLILHVCGQLSGIKNELSRLP-------------------------------------- 204
AmelOr115       AVLVLHLCGQLTVLGTALEDLVNATR---------------------------------- 227
AmelOr118       AALILYVGSQIDLFCQNLT--FHSYK---------------------------------- 212
AmelOr164       ATFVLHIASQLDIICDRLSEILDEHK---------------------------------- 223
AmelOr163       AALVLHIGSQIDITCQELI---EIPR---------------------------------- 225
AmelOr165       LMMVLHIGCQIDILCQNLLDIPHIST---------------------------------- 221
AmelOr166       LMIVLHVGCQIDIMCQTLMKTTHRDQ---------------------------------- 221
AmelOr167       ITLIFHIGGQIEILHKTLKNISINDE---------------------------------- 227
DmelOr35a       CELIVHLNGSYMLLKRDLQLAIEKILVA---------R---------------------- 236
DmelOr74a       GELMMHLNARYIQLGQDLRRSAQMLLKK---------S---------------------- 237
AgamOr3         FSNVKYCSAMLKLVALRIHCLAR------------------------------------- 251
AgamOr5         FSNVKYCSAMLRLVAMRIQFMDR------------------------------------- 232
AgamOr13        CNITMYCTLYFQLVQLKLRTAT-------------------------------------- 231
AgamOr15        CNITTYCTLYFQLVQLKLRTVT-------------------------------------- 231
AgamOr17        CDTTIHCILYFQLAQLKLRIVT-------------------------------------- 231
AgamOr16        LSLIKYCTIYFQLVKVKLEEVG-------------------------------------- 229
AgamOr55        FNFTSYCTVYFRLVALQLQYQT-------------------------------------- 231
AgamOr18        FNMIKYCQTVLRIVVLKLRLLKTL------------------------------------ 238
AgamOr14        ASSVRYAEMLLHVVMLKVDNLHRI------------------------------------ 251
AgamOr51        ASSVRYAEMLLHIVMLKVD---SL------------------------------------ 239
AgamOr12        LSTIKYCSTLLQLVNIAIEGLSS------------------------------------- 241
AgamOr19        LSTIKYCSTLLQLVNIAIEGLSS------------------------------------- 241
AgamOr20        LTTIKYCNTLLQLVIIEVDNLNH------------------------------------- 241
AgamOr21        LSTIKYCSTLLQLVGVEVDNLNH------------------------------------- 238
AgamOr50        LSTIKYCSTLFHLVSIGIDELNL------------------------------------- 240
AgamOr29        VAVIKTTCLLFQTTAMQIRELKDN------------------------------------ 233
AgamOr53        IAAIKTVTLVFGIVTMQIRDLHEQ------------------------------------ 223
AgamOr30        FSLIRTASMLFQATAQQIRNLPPG------------------------------------ 234
AgamOr46        FGLIRFCSTVLGIIVLKIERLSQIGPV--------------------------------- 233
AgamOr47        FGLIRFCSTVLGIIVLKIERLSQIGPV--------------------------------- 233
AgamOr48        M-LIRYNTLLYQLVNRLLQQYDRESAG--------------------------------- 241
AgamOr49        IGIIVYNTLQYQLVSRGVRELK--SLD--------------------------------- 248
AgamOr61        TSVQFTVMRRLKQRMETLAASGHSSTQ--------------------------------- 250
AgamOr62        QQLEETLVP-------TGVGMDDQNEW--------------------------------- 248
AgamOr63        EEVER---------SDRLESDFGDSAD--------------------------------- 243
AgamOr60        ERIDECFHKYR---PELNTASAGEREE--------------------------------- 245
AgamOr75        DSLDDQLQQ------RREQSQGAPDEE--------------------------------- 251
AgamOr76        DSLDDQLRQ------RREQAQGAPDEE--------------------------------- 251
AgamOr78        DSLDDQLRQ------RREQSQGAPDEE--------------------------------- 251
```

```
AgamOr77        LTLTIELRILN----DSYAAAPTDPQQ--------------------------------- 231
AgamOr79        LTLTIELRILN----DSYAAAPTDPQQ--------------------------------- 229
AmelOr99        ILIIHFNSGLFAVCGVQVLEATMNSNL--------------------------------- 241
AmelOr101       ILIIHFNSGLFAVCGVQVLEATMNSNL--------------------------------- 241
AmelOr102       ILIIHHCSGLFTVCGYQVQKVISNAKS--------------------------------- 241
AmelOr103       ILIIHHCSGLFTVCGYQVQQVTGNAKS--------------------------------- 241
AmelOr104       MIIIHHSSGMFAMCGYKVQEATKYQNL--------------------------------- 241
AmelOr98        IIIIHHVCGLFAVCSYEIQKTVKDLTV--------------------------------- 240
AmelOr100       ILIIHHNSGMFAVCGYQIQKIFAEK----------------------------------- 165
AmelOr97        MLIIHHASGLFAMCGYQIAKATECN----------------------------------- 191
AmelOr105       ISIIYHACGLFAACGYQIQKLTKVHTI--------------------------------- 241
AmelOr109       VQIVHHGCALLAISGYHFKHAVDDVKFC-------------------------------- 229
AmelOr110       VQVVHHGCALLAISGYHFKHAVDDVKIC-------------------------------- 229
AmelOr111       VYIVHHGCALLAISGYRFKHAVDDIKLC-------------------------------- 229
AmelOr108       VQIVHHGCALLAISGYHFKHAVDDMKFS-------------------------------- 229
AmelOr107       IHVVHHGCALLTISGYHFKHAMDNVDLC-------------------------------- 229
AmelOr112       VYAVQHACGLLAIAKYRFKNVCKNLK---------------------------------- 227
AmelOr113       MVFVQHVCGLLAVAGYRFKHAIDDSA---------------------------------- 228
AmelOr106       VYVVQHACGLLTLAGYRFRNSLNDLYS--------------------------------- 226
AmelOr122       IFSYYIC-RQFNIVNYRIRKIIEDLS---------------------------------- 227
AmelOr125       IFSYYVC-RQFNIVNYRIRKIIEDLS---------------------------------- 226
AmelOr123       IVSYYIC-RQFHIVCYRIRKIITDLS---------------------------------- 224
AmelOr126       IFSYYIC-RQFHIVCYRIRKIITDLS---------------------------------- 224
AmelOr127       IVSYYIC-RQFHIVSYRIRKIITNLS---------------------------------- 224
AmelOr128       IFSYYIY-RQFDIVSYRIQKIIADLS---------------------------------- 224
AmelOr129       IFSYYVC-RQFDIVSYRMRKIIVNLS---------------------------------- 224
AmelOr133       IYIYHTS-ILFKIISHRIQKIIAYLN---------------------------------- 127
AmelOr134       IYIYHTG-VLFKIISHRIQKIITYLT---------------------------------- 223
AmelOr131       LYSYHTG-MLFKIISYRIRKIITYLT---------------------------------- 225
AmelOr132       LYSYHTS-MLFKIISYRIQKIITYLT---------------------------------- 225
AmelOr135       LYSYHIS-VLFKIINHRIQKIITYLT---------------------------------- 223
AmelOr136       LYSYHTS-IMFKIIGHRIQKIVKYLT---------------------------------- 225
AmelOr138       LYCFHAN-ILFKIISYRIQKIVTYLT---------------------------------- 131
AmelOr130       IYSYYIC-ILLKIVSYRIQKIIMYLA---------------------------------- 225
AmelOr137       LYCFHAS-ILFKIIIYRIQKIVTYLT---------------------------------- 224
AmelOr139       LYTIVTTSLFFKIIGYRIQKIITXLT---------------------------------- 147
BmorOr20        SLMISTVSGHFRILIHNLLTFPLPEAITASKKFVDKHR---------------------- 264
BmorOr3         SLMISTVSGHFRILIHNLLTFPLPEAITASKKFVDKHR---------------------- 264
BmorOr18        SLMISTVSGHFRILIHNLLTFPLPEAITASKKFVDKHR---------------------- 264
HvirOr6         SLLVFNLWGHFKILISTLNEFPRP-----SSKSVDTQ----------------------- 264
BmorOr23        SLMIICLWGHLRIFIHNLNHIPAP------------------------------------ 251
BmorOr16        SLMIICLWGHLRIFIHNLNHIPAP------------------------------------ 251
BmorOr4         SLMIICLWGHLRIFIHNLNHIPAP------------------------------------ 250
BmorOr5         SMMIIHLWGHIRIFIHNLNHIPAP------------------------------------ 251
BmorOr22        SLLILHLWGHLRIILNNLKTFPKPY----------------------------------- 258
BmorOr17        SLLILHLWGHLRIILNNLKTFPKPY----------------------------------- 258
BmorOr1         CLMVMHVWGHLKILSHNLINFPRPKA---------------------------------- 255
BmorOr1a        CLMVMHVWGHLKILSHNLINFPRPKA---------------------------------- 255
BmorOr1b        CLMVMHVWGHLKILSHNLINFPRPKA---------------------------------- 255
BmorOr19        FIAVFHVVAHIQILKNNVKS---------------------------------------- 246
BmorOr15        FIAVFHVVAHIQILKNNVKS---------------------------------------- 246
TcasOr5         SQLLNCTKFKESAQQVKELG---------------------------------------- 208
TcasOr7         AKILIDFFYASTDVFYGTAS---------------------------------------- 215
HvirOr4         SLLVDTMTGREHVVIIKRTASGEPRLIV-------------------------------- 233
BmorOr13        SIDTLAFVVTKRSVRFPTAGWMDQR----------------------------------- 279
DmelOr10a       FEFCAHLSALFEVLQAEIESMFRPYTDHLELS---------------------------- 248
DmelOr33c       PLTLCLLAGHVHLWSIRMGQLGYFDDET-------------------------------- 225
DmelOr7a        VIYLYVVRIQVQLLARRVEKLGTDDSGQVEIYP--------------------------- 245
DmelOr22c       ICSCLYICGAFRLVQQDIRRIFADLHG--------------------------------- 238
AgamOr1         SGLMLHINGQIVRLGSMVKKLGHDVPPERQLVATDAEWKEMRKR---------------- 250
AgamOr4         CCSIGHCTLYTRMTIEMVEQLESMAS---------------------------------- 236
DmelOr47b       ISTFLQTALNLKLLCIEIRKLGDMEVS--------------------------------- 254
AmelOr73        AGLMIQAGVQFEIFCHRAQNLPS------------------------------------- 216
AgamOr68        SLYVDILRFHLDETSELLASPGYQTRNS-------------------------------- 235
DmelOr59a       AVVLCLISSHIKMLYNRFEEVGLDPAR--------------------------------- 224
DmelOr23a       PLVLYLISEQCQLLILRISEIGYGYKT--------------------------------- 219
AgamOr31        GTFSSCLVAHFVIIQERFEGLRFDDG---------------------------------- 221
DmelOr2a        PAFLCLLTGHMRALELRVRRIGCRTEKSNKG----------------------------- 233
AmelOr62        FSLTMHLCGQFEILKTRIAKIEFVDR---------------------------------- 244
AmelOr121       GILILHICGQLTNLRFQLANIKESEAS--------------------------------- 241
```

```
AmelOr140      NVTDHHLLEKYKNITLLSKDEKK------------------------------------- 206
TcasOr12       SGTIMVISTQLSLLKDGLENVARNIKHD-------------------------------- 233
AgamOr8        CAIVLLLCLQFDIVAYRLSHALPDDH---------------------------------- 241
BmorOr9        CVMTSLVCNNFSLISFKLQKVNRN------------------------------------ 226
AgamOr40       MVFVFVVCTQFDMLCCSLSNVRQSAMILNGGYGPELRDYQDNYELDTRDYVLQEVFREDL 273
TcasOr63       WKLITLASAQFEILKENLTKIDYEGG---------------------------------- 224
TcasOr4        TILITLGSAQFDILKIRLENITSVDTS--------------------------------- 218
TcasOr13       GEDFTIVRIDRKKINSIVDTLKVEKKKQ-------------------------------- 243
AgamOr24       IYNLLTAYGQLDVLMTITEELNEQLERN-------------------------------- 221
AgamOr54       GYTLNYGFQMLETTLMVIGIISSES----------------------------------- 215
AgamOr23       LVLIMNATGQMDVIIVYLKQLTLLIDNNHTG----------------------------- 228
AgamOr64       TTTLKQAANEEQQQQQHQHAVDIRCAYR-------------------------------- 253
DmelOr82a      ISFAINLRAHFQTLQRQIENWEFPSSE--------------------------------- 230
AmelOr116      TGFIIITCCQLTILKKCIARNNNINIEK-------------------------------- 236
AmelOr160      VTLTFHLCSQLSIVAYRMRNVNIEPK---------------------------------- 234
TcasOr10       GICFGLFLISKEGLPTEPERLSKYFP---------------------------------- 201
TcasOr26       SETIEHLILRVEDLKILFPKIVAERSP--------------------------------- 218
AmelOr117      MTIILHVSGQFKLINIWINNIGIEINCN-------------------------------- 239
AmelOr120      ATALLHTCGHFTVLKENLKNLDTYIYDLTKTNLKTN------------------------ 237
BmorOr7        ATILGQCKTQFTILRIKFETLPTRAKQALRCD---------------------------- 229
AgamOr6        CILAAHLCMQFRILAHNFGHVVEVANG--------------------------------- 241
DmelOr45a      SWLTHNVVIQFQLLELVLEEKDLNGG---------------------------------- 226
DmelOr67d      FLFVTHVPLIKDIFCVKLTEFNELVMKR-------------------------------- 236
DmelOr83c      FLGLTQILTFADMLQVKVKELNDALEQKAEYR---------------------------- 235
AmelOr162      KFQLILTYLFFFLLIFQISRFLS------------------------------------- 230
HvirOr3        CSMIMYLCDQLTHLQTELRDLTYVKES--------------------------------- 111
TcasOr20       CGVLIHITVQLQCLRKIILDLSQVND---------------------------------- 230
AgamOr11       VTLMIYGRTECSVLNHRLCFLERYHTAGKDD----------------------------- 262
DmelOr56a      TCLMKYVNLKLQILNKRVEEMDITRLNSKLVIG--------------------------- 255
AgamOr28       STLVMHIKMMFKVLKFEIEQLGLDLSAG-------------------------------- 260
AgamOr58       SLEFYWLGQEFEQVFAQCNTFAATARKR-------------------------------- 229
AgamOr59       ELLATCLGELFETVERQVKEEGPTGRPD-------------------------------- 245
DmelOr1a       GWCALGVSLQYRRLGQQLKRIPSCFNP--------------------------------- 232
DmelOr13a      GFFITYTCGQFHLLHQRIAGLFAGSN---------------------------------- 238
AmelOr161      LVVIIHGSGKFAVLRRRMEALNGADPN--------------------------------- 232
AmelOr141      ALFILIICGYFDTIQSRIENLHSFDTSLSSSLLNILSR---------------------- 260
AgamOr52       LLVEFAFDGLLERVERHVQAAGAQESP--------------------------------- 216
AgamOr36       MGLYDELTSIAQEYGRLLTKEKNGQAD--------------------------------- 238
AgamOr25       VLSILAAVHQIKLLKITIQELDIGAEQ--------------------------------- 211
AgamOr45       IVALLNICTRIAALQLLLEELDAKLG---------------------------------- 230
TcasOr3        ITIMSCISSHLKILQGAFKTVRARCIMR-------------------------------- 250
DmelOr88a      NVMQGHLVMHLGHLARQFSAIDPRQSLTD------------------------------- 247
AgamOr22       YLFVCYSASQLEILIVYLRQIGERPDN--------------------------------- 235
DmelOr49a      MCVSSQISMHLGYLANMLASIRPSPETE-------------------------------- 240
DmelOr85f      LYFQVQINLHFRGIIRSLADHKPSVKHD-------------------------------- 237
AmelOr119      TMLLDALSVNFENCNGETKRTICNRHGKEER----------------------------- 253
DmelOr63a      IVLCLHSVGLMRSLNQMVEQATSELVPP-------------------------------- 261
DmelOr67b      VVLTRYESGLIKVLRFLVQNSTSDILVP-------------------------------- 262
BmorOr21       GLCINHACLKMKLYCRALEDAMRSESR--------------------------------- 242
BmorOr11       PIFVLHACGQLDLLSLRITKLFSDTKNP-------------------------------- 241
BmorOr12       KYQYKILCSYFESLNKIFDDETSSHEVK-------------------------------- 244
AgamOr33       RPVDTGPGVQCDSFTPAVAVVSIGKTR--------------------------------- 218
HvirOr1        ICMSYYLTSRLEQVNRKLLAAQGKYLP--------------------------------- 258
HvirOr5        LAKVCELEKQHKDSDKKIEAVKVY------------------------------------ 180
TcasOr1        VKCVSNYTKQQSKIHLIVRSVFVTTY---------------------------------- 193
TcasOr2        VVVNQQIGGIVTYFSTNLPKRETEVRK--------------------------------- 220


TcasOr16       HFRG--GALQNFGSGGVGPN---------GLTKKQELMVRSAIKYWVERHKHVVRLVTAI 344
AmelOr2        TFRP--TAGMTFN-GGVGPN---------GLTKKQEMLVRSAIKYWVERHKHIVRLVTAI 347
AgamOr7        QFRAP-STLQTFDENGRNGN-------PNGLTRKQEMMVRSAIKYWVERHKHVVRLVSAI 347
AaegOr7        QFRAP-STLQTFGDNGINGN-------PNGLTKKQELMVRSAIKYWVERHKHVVRLVSAI 331
DmelOr83b      QFRAP-STLQSFGGNGGGGNGLVNGANPNGLTKKQEMMVRSAIKYWVERHKHVVRLVAAI 355
BmorOr2        TLRGAGGKLQNFNAEN-NPN---------GLTAKQEMLARSAIKYWVERHKHVVRLVASI 341
BmorOr2a       TLRGAGGKLQNFNAEN-NPN---------GLTAKQEMLARSAIKYWVERHKHVVRLVASI 341
HvirOr2        TLRGAGGRLQNFGQQNPNPN---------GLTPKQEMLARSAIKYWVERHKHVVRLVASI 341
AgamOr56       KLKT-----------------------------------------FIALHDSVLRSAETL 264
AgamOr57       KLKT-----------------------------------------FIALHDSVLRSAETL 264
AgamOr26       KFKT-----------------------------------------FIALHDSVLRSARTL 264
AgamOr27       EFEK-----------------------------------------FSTLHDSVLRSAGTL 264
```

```
HvirOr9         KFKE-----------------------------------------LAVRHIELMRCVNLL 268
HvirOr7         KFTE-----------------------------------------LLKWHQDIIYSSTIL 252
DmelOr85b       FLVD-----------------------------------------IVRYHERILRLSDAV 258
DmelOr85c       FLAK-----------------------------------------TVQYHQRILRLMDVL 257
DmelOr85d       FLQA-----------------------------------------TVAYHQSLIHLCQDI 280
DmelOr67a       KLQS-----------------------------------------LVANHIDILRLTDLM 276
DmelOr67c       FLIG-----------------------------------------IIRYHDKCLKLCEHV 272
DmelOr92a       YLHN-----------------------------------------LVVYHARALDLSEEV 277
DmelOr69a       ELSI-----------------------------------------LIAHHSRILQLGDQV 264
DmelOr69b       QLKY-----------------------------------------LIVYHTKLLNLADRV 264
TcasOr6         ---------------------------------NKVYSVMLLNQHLCSLFVQLTQVMDKV 246
TcasOr11        ---------------------------------GHVT----------------KYVPYVI 242
AgamOr41        --------------------------------------IGCKLRSILRNQQRLIRFIANI 257
AgamOr42        --------------------------------------VRDQLVKIIQLQQESTKYITHI 262
AgamOr43        ------------------------------------TSVKLKLREIVLLHQRVLEYEDDL 259
AgamOr44        ------------------------------------TPVKLKLREIVLLHQRVLEYEDDL 259
AgamOr66        ---------------------------------VYKTFCRQRMRAIYQYHQSVIFYLESM 263
AgamOr67        ---------------------------------VYKTFCRQRMRAIYQYHQSVIFYLESM 263
AgamOr72        ---------------------------------EHSAELRHKWVQCMHDHQQSTSFFSTI 259
AgamOr73        ---------------------------------EHSANVRHKWIQCMHDHQQSTSFFSTV 259
AgamOr71        ---------------------------------EHTEDIRRKWVQCMHDHQQSTSFFSTI 259
AgamOr74        ---------------------------------ERTAEIRRKWIQCMHDHQQSTSFFTMI 259
AgamOr70        ---------------------------------KNQSSVREKWVQCMFEHQQTTNFLNSI 259
AgamOr69        ---------------------------------KQTSIMRQKWIQCMQDHQQATSFLNTT 263
TcasOr9         ----------DSFN------EEIFEIMNSAKFKKNNARY---LRKNVKHHQQLLQYCADV 165
TcasOr18        ----------HEFRSFYIKYGNIFKILNNAKFQTLNRAF---LKRNIKHHKLLLRFCEDL 276
DmelOr83a       ----DVEP--GQYAYSVEEETPLQELLKVGSSMDFSSAFRLSFVRCIQHHRYIVAALKKI 317
DmelOr85e       ----DSKRELNQYVLLQEHPTDLLRLSAGRKCPDQGNAFHNALVECIRLHRFILHCSQEL 328
TcasOr24        ---------------------------------------RRRLNECIKYHKEIISYSEIF 255
TcasOr25        ---------------------------------------RERLDFCIRYHNVIIDFSERF 255
TcasOr19        ---------------------------------------RKRLNDCVQYHIDIISYSERF 220
TcasOr22        ---------------------------------------EKRLLHCIRYQQHIIRTNELF 253
TcasOr23        ---------------------------------------EQKLLHCIRYQQHIIRTNELF 253
AmelOr168       -------------------------------------TIRQEMKLLIRHFETVIEMTGIL 256
AmelOr170       ----RN------------------------------DNVRQEMKLVTRHFETVIETTTIL 264
AmelOr169       ----WQNV-----------------------PCEYDKMVKQEMKLLTRHFEIVVEMTVML 270
DmelOr65b       -----------------------------------LQELRMETNRLVKLHQKIVEILDRT 252
DmelOr65c       -----------------------------------YEQLRLETNRLVQFHQKIVHILDHT 252
DmelOr65a       -----------------------------------EDMLYRELCRMTKFHQQIILLTDRC 284
TcasOr17        ----------------------------------NSKEIGKNLVKCIEHHREILR----- 243
TcasOr21        ----------------------------------GCS-IERDLITCIKHHQEILK----- 240
TcasOr56        ------------------------IERIELLDKRTQDYVAKYANECVYHHQELIALCDHA 301
TcasOr15        ------------------------INRIEMLDDGTQVFVEKCANECVYHHQELIALCEHA 295
AgamOr9         ----------------------------SVPPKSDADVVYNELLQCIIFHQEVTGFLREV 284
AgamOr65        ----------------------------SVPTKSDADEVYNELLQCIIFHQEVTGFLREV 287
BmorOr6         ----------------------------SRNRSELHEKNNGILIDCIKHHQAIVRFSELC 257
BmorOr10        ----------------------------QKQ--EVLNSNYELLVECVRRYQSVVRFIELT 254
AgamOr34        -------------------------------------EFDRSFVPLVQHYNKIHRMLCRV 245
AgamOr37        -------------------------------------QFRESFVGLMNHHGKVQRMTAEI 257
DmelOr94a       -------------------------------------IFGQQLRAIFIMHQRIRSLTLTC 253
DmelOr94b       -------------------------------------AR-PELRRIFQLHTKVRRLTREC 249
DmelOr71a       -------------------------------------DLREKFLELIHLHQRLKQQALSI 244
DmelOr46a       -------------------------------------TVEQQLKENIRYHMTIVELSKTV 248
DmelOr46b       -------------------------------------KIAMELRECAAYYNRIVRFKDLV 248
HvirOr8         ----------------------------SRQHRDNHKEAIKKINHCIIHYEKIHKYCSLV 264
BmorOr8         ----------------------------DRGLMN--ITEVLKLNEILKHYELVLKYCSTV 260
AmelOr68        -----------------------------------NVENRLNIGKIVYFHNYLYGYAFMV 242
AmelOr69        -----------------------------------HEKNEVTK-LCIHYHNLIYRLATTI 237
AmelOr70        -----------------------------------QDESNYSAKQCVKHYHQIYKFSRTL 240
AmelOr71        -----------------------------------SDK-NYTMKQCVRHHNHIYKYGEMV 239
AmelOr72        -----------------------------------EDA-IQSLKYCARQHELIYRFTELM 240
DmelOr19a       --------------------------------------MQAILVGYIHDHQIILRLFKSL 251
DmelOr19b       --------------------------------------MQAILVGYIHDHQIILRLFKSL 251
DmelOr33a       --------------------------------------NTRKLIEGIQDHRKLMKIIRLL 246
DmelOr33b       --------------------------------------TGKQLIESIEDHRKLMKIVELL 248
DmelOr22a       --------------------------------------YLEELTECIRDHRLLLDYVDAL 261
DmelOr22b       --------------------------------------YLKELADCVRDHRLILDYVDAL 261
DmelOr42b       --------------------------------------SYEELVKCVMDHKLILRYCAII 261
DmelOr59b       --------------------------------------NYQDLVNCVLDHKTILKCCDMI 262
DmelOr59c       --------------------------------------HYEQMVACIQDHRTIIQCSQII 264
```

```
DmelOr98a       --------------------------------------NEQDLIKCIKDHNLIIDYAAAI 259
DmelOr42a       --------------------------------------KYERLVQCIQDHKVILRFVDCL 264
DmelOr85a       --------------------------------------HYAELVECVKDHKLIVEYGNTL 259
DmelOr43b       --------------------------------------MFKSLVDCIKAHRTMLNFCDAI 264
AgamOr2         --------------------------------GHSAGTLFAELKECLKYHKQIIQYVHDL 247
AgamOr10        ---------------------------------ESTVVLNRKLQKLIEDHKRIIRYVQDL 244
DmelOr30a       -----------------------------------NEQVRGEIIWCIKYQLKLSGFVDSM 246
DmelOr49b       ----------------------------------DPRELREEIIACIRYQQSIIEYMDHI 244
DmelOr43a       ----------------------------------SEEERFQRLASCIAYHTQVMRYVWQL 244
AgamOr32        ------------------------------DLSAAQPYAEHQLRNVITYHREVLSLAQKM 247
AgamOr35        ------------------------------DFAART--AEDELEVVVAYHKDVLQLCLAM 257
DmelOr47a       ------------------------------KGGSLKE-SQATLNKVFALYQTSLDMCNDL 250
DmelOr98b       ------------------------------EGRNTKE-THENLKHVFQLYALCLNLGHFL 251
DmelOr9a        ------------------------------PAVGGKE-ELEGLVQVLLLHQKGLQIADHI 262
AgamOr38        ------------------------------SSAAETLRINLELRRINKRHQTIIDVVSEV 279
AgamOr39        ------------------------------SSAAETHRINLELRRISKRHQAIIDLVSEV 280
DmelOr24a       ------------------------------PSEAEEARIVREMEKLVDRHNEVAELTERL 259
DmelOr45b       ------------------------------PSLRESKICCQRLADIVDRHNEIEKIVKEF 268
AmelOr74        ------------------------------SVAAYTRGCHVALKKCIRRHQSLIEFCNKL 262
AmelOr86        ------------------------------KFN-YASKCYEALKECIRQHQSLIEFCDKL 261
AmelOr75        ------------------------------GLPRYEKCCYERLKDCVVQHQTLIEYCKRL 261
AmelOr84        ------------------------------ESYR-ANVCHAKLRSCVIRHQTLTKYCKQL 258
AmelOr87        ------------------------------SWSSCMENCYATFKLCVKQHQDRITYCHRL 286
AmelOr76        ------------------------------EESALSFYEYSKLKAYIRQHQALIEYCKKL 264
AmelOr85        ------------------------------KSPYLSICKYAKLKIYIRQHQTLIEYCRKL 269
AmelOr88        -------------------------------SSKYCINEYKSFKTCVQQHQALIEYCKKL 267
AmelOr91        ------------------------------DSLYFSNKCYTTFKKYIRQHQALIAYCRKL 268
AmelOr92        ------------------------------DSLHFSNKCYTTFKKYIRQHQTLIAYCRKI 265
AmelOr95        ------------------------------DSCYFASKYYAIFKKCIRQHQALIAYCRKL 264
AmelOr93        ------------------------------DSX--ATKCYAIFKKXQHQALIYLIYCRKL 252
AmelOr77        ------------------------------NTSYFAKKCYEAFKKCIREHQALIAYCEKL 267
AmelOr94        ------------------------------ENMNSSNRCYAILKNCIRYHQALIQFSITL 263
AmelOr96        ------------------------------ANKNSSDECYKAFKNYVQQHQALLDFCETL 263
AmelOr79        ------------------------------TLTKQMHKFYEQFKECVRHHQALIDYHQNL 263
AmelOr83        ------------------------------TFAKQVYKFYEQFKKCVRYHQALIDYYQNL 268
AmelOr80        ------------------------------ILSKQMHKFYEKFRKCVQHHQALIDFYQNL 266
AmelOr81        ------------------------------ILSKQMQKFYEKFKKCVQHHQTLIDFYQNL 263
AmelOr82        ------------------------------ILANRVPKFYEKFRKCVQHHQALIDFYQNL 265
AmelOr78        ------------------------------ILEEQMQKFHEKFKKYVRRHQALIDYHQKL 265
AmelOr89        ---------------------------------AHAREFYDRFKARVPYHQALIDYCEKI 262
AmelOr90        -------------------------------LSKHVHTCYEKFKEYVRYHQALINFYTKL 263
AmelOr142       ---------------------------------------DGEFDACIKKHDELLRYSREI 239
AmelOr146       ---------------------------------------IESLIKCIEKYYAIRRYAEEV 239
AmelOr148       ---------------------------------------IDMLIVCFKKLLLLRRYAEEV 236
AmelOr158       ---------------------------------------IDMLIVCLKKLLFLRRYAEEV 237
AmelOr147       ---------------------------------------IEMLIVCLKKQLLLRRYAEDV 237
AmelOr150       ---------------------------------------ISMLIVCIKKQLHIRRYAKKV 239
AmelOr151       ---------------------------------------IGMLIACVKKQLRIRRYAKKV 239
AmelOr152       ---------------------------------------IGMLIVCVKKQLHIRRYARRV 239
AmelOr154       ---------------------------------------IDMMIACIKKQLQLKRYAEEI 239
AmelOr155       ---------------------------------------IDMVIACVKKQVQLRSYAKEV 239
AmelOr157       ---------------------------------------VDTLVVCLEKHSRLKRYAEEV 236
AmelOr156       ---------------------------------------KDMMVICIKKQLQIRRYAKEI 239
AmelOr153       ---------------------------------------INMLIVCX--------YAKKM 215
AmelOr144       ---------------------------------------YDMLVSCIKEHQKILGFVEDT 240
AmelOr145       ---------------------------------------YDMLVSCIKEHQKILGFVENS 241
AmelOr143       ---------------------------------------YDTLVSCIKEHQKIIGFVEDT 235
AmelOr1         -----------------------------------NRDLHDRVIDIVQSHLKAIEYSARV 266
AmelOr3         -----------------------------------NRDLRGRIADIIQSHLKAIEYSALV 269
AmelOr6         ----------------------------------SENTMQKRIVDIVEYQTKIKRFLGNT 261
AmelOr7         ----------------------------------SENTMQKRIVDIVEYQTKIKRFLGNT 261
AmelOr4         ----------------------------------SEKIMQQRIVDIIEYQTRIKRFLSNT 263
AmelOr5         ----------------------------------SENIIQEKIVEIVEYQTKIKRFLGNA 263
AmelOr8         -----------------------------------ERVVQEKIVHIVEYQTRIKEFLNHV 263
AmelOr9         -----------------------------------EKVVQEKIVYIVQYQTRIKEFYNYV 263
AmelOr11        -----------------------------------EQVVQEKIVHIVEYQTRIKEFLNQL 261
AmelOr12        -----------------------------------EKVVQEKIAHIVDYQTRIIEFLNDL 263
AmelOr10        -----------------------------------ERVVQEKIVHIVEYQMKIKEFLKQI 262
AmelOr14        ----------------------------------NEVVVHEKIVDIVKYQTKVKGFLKNV 265
AmelOr15        ----------------------------------NESAVHMKITDIVRYQTKIKQFLNDV 266
```

```
AmelOr13        ----------------------------------CEIIVRKKLADIVEYQMKIIDFLNHV 257
AmelOr16        ----------------------------------SEVTLQRKIMDIVEYQMKIKRFLKNI 268
AmelOr17        ----------------------------------SEVVLQRKIMDIVEYQMKIKRFLKNI 268
AmelOr18        --------------------------------NDNENL-DQRLANIVKQHVRIINFIALT 277
AmelOr19        --------------------------------NDDENL-DQRLVNIVEQHVRIINFITLT 277
AmelOr22        --------------------------------DMYNNV-DSRIASIVSQHVRILKFLALT 278
AmelOr24        --------------------------------DMYNNV-DSRIASIVNQHVRILKFLALT 270
AmelOr20        --------------------------------DMDERL-DGRIADVIRQHVRVLKFLALT 274
AmelOr25        --------------------------------DMSKAI-DDRIANIVIQHDRILKFLALT 269
AmelOr29        --------------------------------DMSKKI-DDRIANIVIQHDRILKFLALT 270
AmelOr30        --------------------------------DMNKIV-DKRIAKIVVQHDRILKFLALT 270
AmelOr21        --------------------------------DMCYSV-DQRIAKIVSQHVRILKFLSLI 270
AmelOr23        --------------------------------DMCNSI-DQRIATIVNQHVRILKFLSLI 270
AmelOr35        -------------------------------KDMENIVHEQRLGNIVEHHLHILGFISQI 279
AmelOr27        --------------------------------NQKKTDDFKEIGIIVERHLRVLNFITYL 272
AmelOr28        --------------------------------KREK-MIFREIGLIVEHHLRTLNFISCI 272
AmelOr33        --------------------------------VNNKNVGLDKISNIVRHHLRILSFITGI 274
AmelOr34        --------------------------------IQDKNIVLNDIGEIIRHHLKILSFITGI 274
AmelOr32        -------------------------------KIHDKNIGLNKIGKIVRHHLRTLSFVTGI 274
AmelOr31        --------------------------------DNNKNVVINEIGEIVEYHLRILNFIEGI 273
AmelOr36        -------------------------------QKENKN-RIDEISVIIEHHMRILSFLERA 270
AmelOr37        -------------------------------QKENKN-RIDEISVIIEHHMRILSFLERA 271
AmelOr38        -------------------------------QEENKNVHIDKISVIIKHHMRILSFLERA 272
AmelOr39        -------------------------------QEENKNVHIDKISVIIKHHMRILSFLERA 272
AmelOr47        -------------------------------QKQNKNIRIDEISVIIEHHLRILSFLERT 271
AmelOr48        -------------------------------QKQNKNIRIDEISIIIEHHLRILSFLERT 271
AmelOr40        -------------------------------QEENKNMRVNEISVIIEHHLRILSFLGRT 271
AmelOr43        -------------------------------QEENKNMRVNEISVIIEHHLRILSFLGRT 272
AmelOr45        -------------------------------QEENKNVRVEEISVIIEHHLRILSFLERT 271
AmelOr41        -------------------------------QEEKKNMRINEISVIIEHHLRILSFLERT 271
AmelOr42        -------------------------------QEEKKNMRINEISVIIEHHLRILSFLERT 271
AmelOr44        -------------------------------QEEKKNMRIDEISVIIEHHLRILSFLGRI 271
AmelOr46        -------------------------------IDLNN-VYVNKISIIVEHHLRILSFITHI 270
AmelOr49        -------------------------------KKLNNNAYINKIGIIVEHHLRILSFIARV 271
AmelOr50        -------------------------------RKENKDNHINKIGVIVEHHLRILSLIARI 273
AmelOr26        -------------------------------KKQDEQTLHKKLGFIVEHHLRTLSLVWYM 279
AmelOr53        --------------------------------EKRKYKDCDPFALIVHDHVEVLRFSNNI 277
AmelOr54        --------------------------------ERKKYESCNLFALIVHDHVEILRFSNNI 275
AmelOr52        --------------------------------DKK--NNCDPFALIVHDHVEILRFSKNV 272
AmelOr51        --------------------------------KVLETGIESHLAVVVKNHVEILRFAKNV 274
AmelOr55        --------------------------------YKKNSNPNIQVIKIIKHHIKILKFSAVV 273
AmelOr57        --------------------------------YKKNSNANIRLMEIIKHHTRILKFSAVV 273
AmelOr56        --------------------------------YSKNLNPDIRLMGIIKHHIRILKFSAVV 273
AmelOr58        --------------------------------SKENSNPNIRLIEIIEHHIKILRFSAMV 275
AmelOr59        --------------------------------SKEKINLNARFTKIIEHHLRILRFSATV 276
AmelOr60        --------------------------------GKGKFNLNARLIKIVEHHLQILRFSATV 276
AmelOr61        --------------------------------TKNTFNLNTRLVKIVKHHLRILRFSESI 276
AmelOr63        -------------------------------------RTTKHITLLIKRHIYLLKLGDML 262
AmelOr64        -------------------------------------RATKDIILLTKRHIYLLKLSDML 258
AmelOr66        -------------------------------------RTMKHISLLTRRHIYLLKLSDML 258
AmelOr65        -------------------------------------KITEHFILLIKRHIYLLNLSKML 258
AmelOr67        -------------------------------------RTMEHIILLTKRHIYLLNLSKML 258
AmelOr114       --------------------------------TYDKKDLKRRLKEIVQKHEYVNRFAETI 232
AmelOr115       --------------------------------RNDYKTFEQRLSSIVNRHNHLSRFAVIV 255
AmelOr118       --------------------------------KRESQ--D-TIKDIIVRHQKIIQLSKNI 237
AmelOr164       --------------------------------EQELR--IRIIKKLIAKHQRTLNLSENI 249
AmelOr163       --------------------------------HKGKT--SYILKNIIVKHQRILRLSENV 251
AmelOr165       --------------------------------SH--------LKFFIIRYQEIITFAERV 241
AmelOr166       --------------------------------KK--------LKFFIKRHQEIILLAEKI 241
AmelOr167       --------------------------------KPESS--RIIIKSLIDRHYRIIIGSEYI 253
DmelOr35a       ------------------------------DRPHMAKQLKVLITKTLRKNVALNQFGQQL 266
DmelOr74a       ------------------------------SSLNVAIAYRLNLTHILRRNAALRDFGQRV 267
AgamOr3         ---------------------------------VAQDRAEKELNEIISMHQRVLNCVFLL 278
AgamOr5         ---------------------------------LDEREAEKELIEIIVMHQKALKCVELL 259
AgamOr13        ---------------------------------EDNTFNRQEVKSIVVMHQDALNCASLV 258
AgamOr15        ---------------------------------QDNTF-RQELKSVIKMHQDALNCASLV 257
AgamOr17        ---------------------------------QDNTF-RQELKTVIKMHQDALNCASLL 257
AgamOr16        ---------------------------------RSQNY-ASDIRSAIQMHQSALSCADVL 255
AgamOr55        ---------------------------------RTVPSDRDSIRTIVAMHQQALCCADLL 258
AgamOr18        ---------------------------------PDVRQRSDGMSEVWVLHQRALRCAELL 265
```

```
AgamOr14        ---------------------------------PNQKSIREELHDIIHVHQRTLNCITLL 278
AgamOr51        ---------------------------------PKQKSIREELHDIIKVHQRTLDCIALL 266
AgamOr12        ---------------------------------ISPENIDLELKKLIQMHQLAIRCVALL 268
AgamOr19        ---------------------------------ISPENIDLELKKLIQMHQLAIRCVALL 268
AgamOr20        ---------------------------------ATSNAIDRELKQVIQLHQRAVRCVALL 268
AgamOr21        ---------------------------------TSSEAIGRELKKVIQLHQLALRCVALL 265
AgamOr50        ---------------------------------VSPAHIDRELKKLIQMHQLAIRCDVLL 267
AgamOr29        -------------------------------------ISQAQLSVVIKSHRDTLLCAQYL 256
AgamOr53        -------------------------------------ITQERLNRVIKSHSNALSCATQL 246
AgamOr30        -------------------------------------ASQAKLEAIIHSHRSTLKCAAQL 257
AgamOr46        ---------------------------------D---QYTAELSEIIELHQLVIKCSRQL 257
AgamOr47        ---------------------------------D---KYTAELNEIIELHQLAIKCSRQL 257
AgamOr48        ---------------------------------GLLAQKHRRLQQIVQLHYRAIECTKLL 268
AgamOr49        ---------------------------------PTTTQFRHRLTEIIDQHGAATRCTKLL 275
AgamOr61        ---------------------------------VFWSILQPELNSHISRHVDLLDNLKEF 277
AgamOr62        ---------------------------------EYWDNLQQRLTHRIKRHLELLENLRIF 275
AgamOr63        ---------------------------------IFWNNIVDQLRPHMQRLDELFIQLQHL 270
AgamOr60        ---------------------------------EFWRELQALIKLNVQRHVELLENLVDF 272
AgamOr75        ------------------------------------RILRELLYEHARHHSQLIVVVTHL 275
AgamOr76        ------------------------------------RILRELLYEHARHHSQLIVVVTHL 275
AgamOr78        ------------------------------------RILRELLYEHARHHSQLIVVVTHL 275
AgamOr77        --------------------------------------LVAFLKERVVYKAALLQHIRTI 253
AgamOr79        --------------------------------------LVAFLKERVVYKAALLQHIRTI 251
AmelOr99        ----------------------------ISKDAFSENSSYEKFRTCVIMHNEVIEFYNIL 273
AmelOr101       ----------------------------VSKDAFSENSSYEKFRTCVIMHNEVIEFYNIL 273
AmelOr102       ----------------------------FN-GTVLNNYTYEQIKNCVIMHDEAIQFYNIL 272
AmelOr103       ----------------------------LN-KIVSNNYTYEQIRNCVITHDEAIQFYNIL 272
AmelOr104       ----------------------------FNDRIISENYTYEQLKNCITIHNKALQFYNIL 273
AmelOr98        ----------------------------FT---DIEKCSYKELKNCVIKHKKAIKFYNIL 269
AmelOr100       --------------------------------VFSNIHIYEQFKNCLIVHSEAIQFFSIL 193
AmelOr97        ----------------------------------DRHNENELFRQCVMTHNKAYKFFEIM 217
AmelOr105       ----------------------------EKNGPNISNIDYEEFKQCVIMHYKCLQLYDVL 273
AmelOr109       ---------------------------DGYYIDASMDETYVKIRQSIKAHKTAVQYVDKI 262
AmelOr110       ---------------------------DENYITL-MDETYTRVRHSIKAHRTAVEYVDKI 261
AmelOr111       ---------------------------GGDCIDPLTDETYTKVRQSIKAHKMAVEYVDKI 262
AmelOr108       ---------------------------NGNYIDLLMDETYKKVKQSIKAHKTAVEYVDKI 262
AmelOr107       ---------------------------NEKYSDELMEKTYAKVSQSIDAHKKAVEYVNKI 262
AmelOr112       ----------------------------EDHAIPLEEIKYKSICESIKAHQHALKYLKLI 259
AmelOr113       ----------------------------SKNSEEKIKETCKKIRSSIQGHEGAIRYLKKI 260
AmelOr106       ----------------------------MRKDSKMDEKIYRRMCYAIKTHKRALAYLTKI 258
AmelOr122       ----------------------------------------------------TRNLS--K 233
AmelOr125       ----------------------------------------------------TPNLS--K 232
AmelOr123       ----------------------------------------------------MPNLP--K 230
AmelOr126       ----------------------------------------------------TPNLP--K 230
AmelOr127       ----------------------------------------------------MSNLP--Q 230
AmelOr128       ----------------------------------------------------MPNLP--K 230
AmelOr129       ----------------------------------------------------MPNLP--K 230
AmelOr133       ----------------------------------------------------MFNLLSNQ 135
AmelOr134       ----------------------------------------------------IIDLSSKQ 231
AmelOr131       ----------------------------------------------------MFNVSSKQ 233
AmelOr132       ----------------------------------------------------IVNLSSKQ 233
AmelOr135       ----------------------------------------------------IVNLSSKQ 231
AmelOr136       ----------------------------------------------------MFNLSSKQ 233
AmelOr138       ----------------------------------------------------MFNLSSKQ 139
AmelOr130       ----------------------------------------------------MFKLSPKQ 233
AmelOr137       ----------------------------------------------------MFNLSSKQ 232
AmelOr139       ----------------------------------------------------IFNLSSKQ 155
BmorOr20        ---------------CNGNRSEFVLEEAKLYSPAEMWQVTDRLRQCIDYHRKLVEFTGDI 309
BmorOr3         ---------------CNGNRSEFVLEEAKLYSPAEMWQVTDRLRQCIDYHRKLVEFTGDI 309
BmorOr18        ---------------CNGNRSEFVLEEAKLYSPAEMWQVTDRLRQCIDYHRKLVEFTGDI 309
HvirOr6         -------------------------ESPYKYTEEELIEVAEKLKDCINYHREIKIFTNRM 299
BmorOr23        -------------------------RNSFEYTKEERQEVDDTLKKCIQHHTLIIGFVRIM 286
BmorOr16        -------------------------RNSFEYTKEERQEVDDTLKKCIQHHTLIIGFVRIM 286
BmorOr4         -------------------------RNSFEYTKEERQEVDDTLKKCIQHHTLIIGFVRIM 285
BmorOr5         -------------------------RNSLEYTREERQEVDNTLKKCIQHHTLIIGFVRIM 286
BmorOr22        -------------------------TNNSMYTEEENQVVLLKLQECIRYHNFIISFTVMM 293
BmorOr17        -------------------------TNNSMYTEEENQVVLLKLQECIRYHNFIISFTVMM 293
BmorOr1         ---------------SHVITTPNGPTNVETYTEEESKEVFARLRECIKHYGTVDDFANDM 300
BmorOr1a        ---------------SHVITTPNGPTNVETYTEEESKEVFARLRECIKHYGTVDDFANDM 300
BmorOr1b        ---------------SHVITTPNGPTNVETYTEEESKEVFARLRECIKHYGTVDDFANDM 300
```

```
BmorOr19        -------------------------NWSDDFNESEKK---GYLVSILEYHAYIIRIFGEV 278
BmorOr15        -------------------------NWSDDFNESEKK---GYLVSILEYHAYIIRIFGEV 278
TcasOr5         -----------------------------------------------KIHKDLNKLAMEV 221
TcasOr7         -----------------------------------------------ALEVQTSLLATVL 228
HvirOr4         -------------------------------------SPCDAVRRLAALHGTLCDVVNSI 256
BmorOr13        -----------------------------------------TIRRLALSYGSICEVVRQI 298
DmelOr10a       --------------------------------PVQLYILEQKMRSVIIRHNAIIDLTRFF 276
DmelOr33c       ------------------------------------VVNHQRLLDYIEQHKLLVRFHNLV 249
DmelOr7a        -------------------------------DERRQEEHCAELQRCIVDHQTMLQLLDCI 274
DmelOr22c       -------------------------DSVDVFTEEMNAEVRHRLAQVVERHNAIIDFCTDL 273
AgamOr1         ----------------------------IDHHSKVYGTMYAKVTECVLFHKDILRFGDEV 282
AgamOr4         -----------------------------------AERTASAIRNVGQMHSGLLKCIRLL 261
DmelOr47b       -----------------------------------DKRFHEEFCRVVRFHQHIIKLVGKA 279
AmelOr73        --------------------------------------------LVLTVTRNSNVFAETV 232
AgamOr68        --------------------------------PEIMAKVSRKWRMCLVEHQRIVEYYDNF 263
DmelOr59a       -------------------------------------DAEKDLEACITDHKHILELFRRI 247
DmelOr23a       -----------------------------------LEENEQDLVNCIRDQNALYRLLDVT 244
AgamOr31        ---------------------------------------NRELKKLIEHHKYILRISDRV 242
DmelOr2a        -----------------------------QTYEAWREEVYQELIECIRDLARVHRLREII 264
AmelOr62        ----------------------------------------RKIGPLVKRHCQLAVLVNDL 264
AmelOr121       ---------------------------------------NFILIAIVKDHIRLIRVIELL 262
AmelOr140       --------------------------------------VYRKLFICIKNHQNAIKYSNLL 228
TcasOr12        -----------------------------------KSSVNKNLIQCACHYRSIIQFAAEV 258
AgamOr8         ----------------------------------------QELVGCVRIHQAVIELCNEL 261
BmorOr9         --------------------------------------TAHLLKEVVKEQQYVLKLAEDL 248
AgamOr40        DTVQG----------TKTASRLDQLSPAQSYLMELAPELTCVMEDCIQHHLILLRFCQLL 323
TcasOr63        -----------------------------------FNETKGALVRCITHHAKIVNYTERV 249
TcasOr4         ----------------------------------KSWLVKKAIKKCVIYHTILLKKIFYG 244
TcasOr13        ---------------------------------AKCGVTNEQVRDLIIAYQKLTDAANLV 270
AgamOr24        ---------------------------------ESLDTIQDKIVEIIRQYQHHRTYLKQL 248
AgamOr54        --------------------------------------AFFMFQQNACLQVDMLRYSKSM 237
AgamOr23        ---------------------------------QHDEEIADIIKEIVLKHLEHTKYMTDM 255
AgamOr64        -------------------------------------SFCKQFYANIRRHTELLQSIATF 276
DmelOr82a       ------------------------------------PDTQIRLKSIVEYHVLLLSLSRKL 254
AmelOr116       ------------------------------SPSKIYNKFYENLKHCVKHSIIIFDFTKQI 266
AmelOr160       -------------------------------------IYFPKMKALVERHTELLRLANIL 257
TcasOr10        -------------------------------------YIFSFTMQSFTFCMTGTMLSDWS 224
TcasOr26        ------------------------------------EVRRKMLAKWVDYHLWLLSIGKLM 242
AmelOr117       --------------------------------PNYMRKLKVDLIKCIRHHQQLIHVVNNV 267
AmelOr120       ----------------------------SKYINKNLYEIKTQIIYIIKHHQLVLWFCDNM 269
BmorOr7         ------------------------------SEQNYDEVLMRLFHDCLKHYQKIVDTTSSL 259
AgamOr6         -------------------------------AREGDSGSTSRLRDAIRIHQELIGYEGTP 270
DmelOr45a       ---------------------------------------DSRLTGFVSRHRIALDLAKEL 247
DmelOr67d       ---------------------------------NDFPKVRAMLCDLLVWHQLYTRMLQTT 263
DmelOr83c       -------------------------------ALVRVGASIDGAENRQRLLLDVIRWHQLF 264
AmelOr162       -----------------------------------------------------LRFAKIL 237
HvirOr3         -----------------------------------EMAMRLKFKNAIRKHIRLMGYSGRM 136
TcasOr20        -----------------------------------LEILEHKMKFCVKYHTAILDYGIRT 255
AgamOr11        -----------------------------PTAPVNNAERRSLMIDCIKRQASLVSFTREL 293
DmelOr56a       ----------------------------RLTASELTFWQMQLFKEFVKEQLRIRKFVQEL 287
AgamOr28        ---------------------------------KSHVELQAKLKQIILKHKTNLSLIEQL 287
AgamOr58        ----------------------------------YWDGLKRRIDTCVIEHQRLLGQISTL 255
AgamOr59        ------------------------------SRALYWSTLHGELRRCAQRHCAIFTMLPKL 275
DmelOr1a        ------------------------------------SRSDFGLSGIFVEHARLLKIVQHF 256
DmelOr13a       -------------------------------AELAESIQLERLKRIVEKHNNIISFAKRL 267
AmelOr161       --------------------------------------SAAIMGDNVIRHQQAIKFADTL 254
AmelOr141       -------------------KITTAKMSDIKTEASNSVQMRNLRMCVIHHQLLLNRFCEDI 301
AgamOr52        ---------------------------------LLWQQFNRELGRCVREHCVVLKQVREV 243
AgamOr36        ----------------------------------IWTSFERNTARAVRRHETFLWQLGQL 264
AgamOr25        ------------------------------------AELHRELVRIIQIHQRIQQFIHQL 235
AgamOr45        -----------------------------TVQWQQTAHLDAELNRIIELHIDTKRFARVI 261
TcasOr3         ------------------------------TRAERLLKEETLHDPPFLENCVNKEMIRKL 280
DmelOr88a       -----------------------------------EKRFFVDLRLLVQRQQLLNGLCRKY 272
AgamOr22        -----------------------------------VQEQRRLMRKVFEIHTGLSQFIARC 260
DmelOr49a       ------------------------------------QQDCDFLASIIKRHQLMIRLQKDV 264
DmelOr85f       ------------------------------------QEDRKFIAKIVDKQVHLVSLQNDL 261
AmelOr119       --------------------------------NDNNNRFLDRYKKCVQFHQRLVVISRDY 281
DmelOr63a       ------------------------------------DRRVEYLRCCIYQYQRVANFATEV 285
DmelOr67b       -----------------------------------KDQRVKYLQCCVRLFARISSHHNQI 287
BmorOr21        --------------------------------------RHEKIVAVIEEQRRTYEYIALI 264
```

```
BmorOr11        ------------------------------------RIIAKELKVIISKLQELYGFVNFI 265
BmorOr12        -----------------------------------EAEFENAFCNGIKIHTQIIWCVRRC 269
AgamOr33        -------------------------------------MSLALFSTPERPLLFTGSFCNRL 241
HvirOr1         ---------------------------------------EIFWRATREDYCRVTQIVRKV 279
HvirOr5         -----------------------------------------TWRKIREAYVKQAMLVRKI 199
TcasOr1         ------------------------------------LMFDYCTVLRVQRFNNYQSLAHYL 217
TcasOr2         --------------------------------------KSLDFGKLCSLHHHLSKLIKSF 242


TcasOr16        GDAYGVALLLHMLTSTIMLTLLAYQATKITGVDKYAATVLG--YLLFA------------ 390
AmelOr2         GDAYGVALLLHMLTTTITLTLLAYQATKIHAVDTYAASVVG--YLLYS------------ 393
AgamOr7         GDTYGPALLLHMLTSTIKLTLLAYQATKIDGVNVYGLTVIG--YLCYA------------ 393
AaegOr7         GETYGAALLLHMLTSTIKLTLLAYQATKIDALNVYGLTVIG--YLVYA------------ 377
DmelOr83b       GDTYGAALLLHMLTSTIKLTLLAYQATKINGVNVYAFTVVG--YLGYA------------ 401
BmorOr2         GDTYGTALLFHMLVSTITLTLLAYQATKINGINVYAFSTIG--YLVYT------------ 387
BmorOr2a        GDTYGTALLFHMLVSTITLTLLAYQATKINGINVYAFSTIG--YLVYT------------ 387
HvirOr2         GDTYGTALLFHMLVSTITLTLLAYQATKINGINVYAFSTIG--YLSYT------------ 387
AgamOr56        EEILSLQMLLLYISTILALCLGMVVLSLAVNEVYVLLTTMA--VFGYC------------ 310
AgamOr57        EEILSLQMLLLYLSTILALCLGMVVLSLAVNEVYVLLTTMA--VFGYC------------ 310
AgamOr26        EAILSGQILLLYVSTILAVCLGMVVLTLAIEDVYLLLTTFV--AFGYC------------ 310
AgamOr27        EEILSGHMLLLYVSTIFSLCLSMVVLSLPIDDFILLLTTFV--AFGYC------------ 310
HvirOr9         EKIFSKSILFNALTSSVIICVTGFNVLVVDNIVMMASFTA---FLLFG------------ 313
HvirOr7         EIIYSKSTLFNFLSSSLVICLTGFNVTIVDDIVIIITFLT---FLSMA------------ 297
DmelOr85b       NDIFGIPLLLNFMVSSFVICFVGFQMTVGVPPDIVVKLFL---FLVSS------------ 303
DmelOr85c       NDIFGIPLLLNFMVSTFVICFVGFQMTVGVPPDIMIKLFL---FLFSS------------ 302
DmelOr85d       NEIFGVSLLSNFVSSSFIICFVGFQMTIGSKIDNLVMLVL---FLFCA------------ 325
DmelOr67a       NEVFGIPLLLNFIASALLVCLVGVQLTIALSPEYFCKQML---FLISV------------ 321
DmelOr67c       NDLYSFSLLLNFLMASMQICFIAFQVTEST-VEVIIIYCI---FLMTS------------ 316
DmelOr92a       NNIFSFLILWNFIAASLVICFAGFQITASN-VEDIGVYFI---FFSAS------------ 321
DmelOr69a       NDIMNFVFGSSLVGATIAICMSSVSIMLLD-LASAFKYAS---GLVAF------------ 308
DmelOr69b       NRSFNFTFLISLSVSMISNCFLAFSMTMFD-FGTSLKHLL---GLLLF------------ 308
TcasOr6         EFSDPLFFLNVIGMHPFKADKFSKFRLAFSIAVYFA-------VIFSG------------ 287
TcasOr11        SFITQTFTFCFIGS----------LLITWSLQVPDA-------IFYN------------- 272
AgamOr41        EAAKNVHSAVEVLSLGLQIVITLFVLQFSLWIPGLV-------LIPVF------------ 298
AgamOr42        ERFYQLQSFVEFLCNSLQVALTLNELHRNFWLPGLF-------ILPIA------------ 303
AgamOr43        DKRYYLNNWVQVASSIFNLTGALFGCYVSNSFTMYA-------LAITI------------ 300
AgamOr44        EKRYYLNNWVQVASSIFNLTGALFGCYVSNSFTMYA-------LAIAV------------ 300
AgamOr66        QECYRNICVVQVASCSLSTVFNLFLALTTDWYATYG-------FIVIS------------ 304
AgamOr67        QECYRNICVVQVASCSLSTVFNLFLALTTDWYATYG-------FIVIS------------ 304
AgamOr72        ENIFGLMCLAQVATATLSICDAMLLVLLTDWYPTYS-------YLYVM------------ 300
AgamOr73        ENIFGLMCLAQVSTATLSICDAMLLVLLTDWYPTYS-------YLYVV------------ 300
AgamOr71        ENLFGLMCLAQVATEFLSICDAMLLVLLTDWYPTYS-------YLYVM------------ 300
AgamOr74        EHIFGLICLTQVATATFSICDAMLLVILTDWYPTYS-------YLYVM------------ 300
AgamOr70        EDIFGLMCLAQVSMGVFTICDAMLLVALTDWYPTYS-------FLLVM------------ 300
AgamOr69        EDIFGITCLAQVLMGIFTVCDGMLLVALTFWFPTYC-------FLLVM------------ 304
TcasOr9         SDILSIFLMGKVSAAIFNTLFMAFSLITTGNR---AMIFGLGSYMVST------------ 210
TcasOr18        NKILNTFLLIRVSAIVFNLIFIGFNIIISTDR---TLMLGFCNYFCFG------------ 321
DmelOr83a       ESFYSPIWFVKIGEVTFLMCLVAFVSTKSTAANSFMRMVSLGQYLLLV------------ 365
DmelOr85e       ENLFSPYCLVKSLQITFQLCLLVFVG--VSGTREVLRIVNQLQYLGLT------------ 374
TcasOr24        SKCFSIEM---FTHLTTTGIICGCLENQVVQEHRPE----AILHIGGW------------ 296
TcasOr25        SRCFSYVM---FIHLAITGIIIGCLENQIVQEHQPE----AMLHMGGW------------ 296
TcasOr19        NNCFTNGM---FIHLATTGIIIGCLENQNVEGATPG----GVLHLFGW------------ 261
TcasOr22        NVCYKHCN---GCYVVMVGIIIASLLNQIIKVTAPGLKK-ASELLSIL------------ 297
TcasOr23        NVCFKHCN---GCYVVMVGIIIASLLNQILKEKSVG----ALVHFAGW------------ 294
AmelOr168       KKLLSPNIGILYLNYVFRFCFLSFMFATTSLSEKLTYTI-IVSYTTGA------------ 303
AmelOr170       KKLISPNIGFLYLSYVFRFCFLSFMFAMT-TAKYFEKCL-LASYTIGA------------ 310
AmelOr169       KKLLSPNIGILYINYVFRFCFLSFMLATS-SGMHFEKCL-LVSYTIGA------------ 316
DmelOr65b       NDVFHGTLIMQMGVNFSLVSLSVLEAVEARKDP---KVVAQFAVLMLL------------ 297
DmelOr65c       NKVFHGTLIMQMGVNFFLVSLSVLEAMEARKDP---KVVAQFAVLMLL------------ 297
DmelOr65a       NHIFNGAFIMQMLINFLLVSLSLFEVLAAKKNP---QVAVEYMIIMLM------------ 329
TcasOr17        ------------------------------------------------------------
TcasOr21        ------------------------------------------------------------
TcasOr56        EEDFCYLMLLQFISSLLIVCFQLFQVSTLSPDS---VEFFSMVCYLLL------------ 346
TcasOr15        EEDFCYLMLLQFISSLLIVCFQLFQLS--------------------------------- 322
AgamOr9         LTLFSGPMLSQLYCSVFILCITEFRLLTDVNTM---ADTMQAVMYLVC------------ 329
AgamOr65        LDLFSGPLLAQLYCSVFILCITEFRLLTDVNTM---ADTVQAVIYLLC------------ 332
BmorOr6         EGTFQVHSFFHLGGIVFMICVIGFRMAGESPVS---AQFWAALSYLVI------------ 302
BmorOr10        EKTYHANIFFQLSGSVFIICNIGFRIAIVDSNS---LQFYSMLTYLVT------------ 299
AgamOr34        QNLFSPAYFVQFSVSGLVICASAYQVASMLNLN-DFSKLMN-VFYMMS------------ 291
AgamOr37        EQLFSAAYFAQFGSSGLVICASAFKTSSMFNLY-ELTAIQN-LLYMLS------------ 303
```

```
DmelOr94a       QRIVSPYILSQIILSALIICFSGYRLQHVGIRD-NPGQFISMLQFVSV------------ 300
DmelOr94b       EVLVSPYVLSQVVFSAFIICFSAYRLVHMGFKQ-RPGLFVTTVQFVAV------------ 296
DmelOr71a       EIFISKSTFTQILVSSLIICFTIYSMQMSPVLQ-DLPGFAAMMQYLVA------------ 291
DmelOr46a       ERLLCKPISVQIFCSVLVLTANFYAIAVLSDER---LELFKYVTYQAC------------ 293
DmelOr46b       ELFIKGPGSVQLMCSVLVLVSNLYDMSTMSIANGDAIFMLKTCIYQLV------------ 296
HvirOr8         EDVFSITLFVQFGMASCIICICLMRFTMP-APLVYYLFLAT---YMFV------------ 308
BmorOr8         QSILDVAMFVQFGVASAIICVAMCGLIMVRSSTETLLFMVT---YLFA------------ 305
AmelOr68        QEKFKKIIGIQLLSSTLVVCFILYKLANTSLIST---KFLEFVLYLAC------------ 287
AmelOr69        NEQFKMVIFVQFTVSTLTICVNLYILMGTQITFE---RIMQLAIYSSC------------ 282
AmelOr70        NEKFKVILFLQFCAIAFILCFNLYRMTTITMIP----KLLEASLYLIR------------ 284
AmelOr71        NDAFQSIMFFQFCTSLSMICFNFYRIMQIEMDS----RYVGTILYMVC------------ 283
AmelOr72        NKLFSSILCLQFLISAVAICFSVYRVIYTKTDS----QFAGAIIFVFS------------ 284
DmelOr19a       ERSLSMTCFLQFFSTACAQCTICYFLLFGNVG---IMRFMNMLFLLVI------------ 296
DmelOr19b       ERSLSMTCFLQFFSTACAQCTICYFLLFGNVG---IMRFMNMLFLLVI------------ 296
DmelOr33a       RSTLHLSQLGQFLSSGINISITLINILFFAEN---NFAMLYYAVFFAA------------ 291
DmelOr33b       RSTMNISQLGQFISSGVNISITLVNILFFADN---NFAITYYGVYFLS------------ 293
DmelOr22a       RPVFSGTIFVQFLLIGTVLGLSMINLMFFST----FWTGVATCLFMFD------------ 305
DmelOr22b       RSVFSGTIFVQFLLIGIVLGLSMINIMFFST----LSTGVAVVLFMSC------------ 305
DmelOr42b       KPVIQGTIFTQFLLIGLVLGFTLINVFFFSD----IWTGIASFMFVIT------------ 305
DmelOr59b       RPMISRTIFVQFALIGSVLGLTLVNVFFFSN----FWKGVASLLFVIT------------ 306
DmelOr59c       RPILSITIFAQFMLVGIDLGLAAISILFFPNT---IWTIMANVSFIVA------------ 309
DmelOr98a       RPAVTRTIFVQFLLIGICLGLSMINLLFFAD----IWTGLATVAYING------------ 303
DmelOr42a       RPVISGTIFVQFLVVGLVLGFTLINIVLFAN----LGSAIAALSFMAA------------ 308
DmelOr85a       RPMISATMFIQLLSVGLLLGLAAVSMQFYNT----VMERVVSGVYTIA------------ 303
DmelOr43b       QPIISGTIFAQFIICGSILGIIMINMVLFAD----QSTRFGIVIYVMA------------ 308
AgamOr2         NSLVTHLCLLEFLSFGMMLCALLFLLSISNQLAQMIMIGSYIFMILSQ------------ 295
AgamOr10        NDLVTYICLIEFLSFGLMLCALLFLLNIISVMAQIVIVGAYIFMILTQ------------ 292
DmelOr30a       NALNTHLHLVEFLCFGAMLCVLLFSLIIAQTIAQTVIVIAYMVMIFAN------------ 294
DmelOr49b       NELTTMMFLFELMAFSALLCALLFMLIIVSGTSQLIIVCMYINMILAQ------------ 292
DmelOr43a       NKLVANIVAVEAIIFGSIICSLLFCLNIITSPTQVISIVMYILTMLYV------------ 292
AgamOr32        VQLYQQSAFYLLLLVSTILCLLGYEFVMVSNIYKRMQVAILASIMIGQ------------ 295
AgamOr35        TNLFQYTVFYLLLLDSVLLCVIGYQFVIFMNTPRVLMLASMAFVMVLQ------------ 305
DmelOr47a       NQCYQPIICAQFFISSLQLCMLGYLFSITFAQTEGVYYASFIATIIIQ------------ 298
DmelOr98b       NEYFRPLIC-QFVAASLHLCVLCYQLSANILQPALLFYAAFTAAVVGQ------------ 298
DmelOr9a        ADKYRPLIFLQFFLSALQICFIGFQVADLFPNPQSLYFIAFVGSLLIA------------ 310
AgamOr38        RHAFTPTILIVHICAAFMICVISMAMFLAEG-INKLTYMPYTFTVLML------------ 326
AgamOr39        RSAFTPNVLLVYTATAIIMCIVCIAMLVVEG-IYKLTYLPYAFAELTL------------ 327
DmelOr24a       SGVMVEITLAHFVTSSLIIGTSVVDILLFSG-LGIIVYVVYTCAVGVE------------ 306
DmelOr45b       SGIMAAPTFVHFVSASLVIATSVIDILLYSG-YNIIRYVVYTFTVSSA------------ 315
AmelOr74        EQVFTFPILSHVVVFSLLMCFDTYEILLADIPTLKRLIFLCHMVASFI------------ 310
AmelOr86        EHVYTLPIFGHVVVFSLLMCFDTYEIFLANVPVSMRLIFFFHMVGSFI------------ 309
AmelOr75        EDIFTVMVLGQVMFLAVVICLVGFQLFLADTSASKKASLVLNLGGTFF------------ 309
AmelOr84        ENIFTIIVLGQVLFLALVICLVGFQLFLMDTPASRKVSLTLNFAGTLC------------ 306
AmelOr87        NDIFTIIVLGHILVFSLLMCLVGFQVLMANSPPTRRLIFVFHITGSLC------------ 334
AmelOr76        EQVFNSIVFGQVLLFSLLMCLDGYLILMEETPFGRRVTFTFHITGCMC------------ 312
AmelOr85        EMVSNFIIFGQVLLFSLLICLDGYLILMEDTSNMSRLIFTFHLISCMC------------ 317
AmelOr88        EDVFSIIVLAQVLLFSLLICLDGYLVLMEDTSRAKRVIFTFHLMGCMC------------ 315
AmelOr91        EVVFNWIVLEQVLMFSLLICLDGYQILMANGDIKTRLTFSFHILACLC------------ 316
AmelOr92        EVVFNWIVLEQVLMFSLLICLDGYQILMADEDIKTRSIFSFHILSCLC------------ 313
AmelOr95        EEVFNLIVLEQVLMFSLLICLDGYLVLMADTSTTTRLIFGLHITVCLC------------ 312
AmelOr93        EEVFNLIVLEQVLMFSLLICLDGYQILMADGDVKTRLIFSFHILGFLC------------ 300
AmelOr77        EKVFSLIILCQVLTFSLIICLDGYQIIL-------------------------------- 295
AmelOr94        EEIFTIITLGQVLIFSTLICFVGYQVLLVNMTLSWRISFLCFLITNMC------------ 311
AmelOr96        EEIFTIIVIGQILMFSILFCFLGYQVILADLTPSYRISFISYLFAGMC------------ 311
AmelOr79        ENVYTIITLGQVLVFSVLICLFGYQVFVATASFARRSIFVFMLNGSMF------------ 311
AmelOr83        ENVYTIITLGQVLVFSVLICLFGYQVFVAAASTARRFIFVFLLSGSMF------------ 316
AmelOr80        ENVYTMITFGQVLVFSVLICLFGYQVLVATISFARRFIFVFMLNGSMF------------ 314
AmelOr81        ENVYTTITLEQVLVFSVLICLFGYQVLVATASFARRFIFVFLLNGSIF------------ 311
AmelOr82        ENVYTRIAFGEMLVYSILICLFGYQVLVATASFARRSIFVFLLNGSTF------------ 313
AmelOr78        ENVYTTIMLSQVLLFSVLICLFGYQVLLATASLARRSIFIFLLMGAMF------------ 313
AmelOr89        ENVSPTIILEPVMVFSVIICLFGYRILWANAPSTRRSIFIFLLIGAMS------------ 310
AmelOr90        ENVYTMIILGQVIVFSALICLFCYQVLLANAPSARRSIFIFLLIGAMS------------ 311
AmelOr142       RQSIKFLILATNGTTVIAVIFGSLNLIANQ-PLILKALYAIVVFSASV------------ 286
AmelOr146       VNSARYTTLITLCICGVESVFGGIIFIGRQ-PFTVKLQFLTLSATTLL------------ 286
AmelOr148       VSCIRFLVFYAIAVGTFMLTLSGIIMIINS-PILVKIQFIIICMSSLM------------ 283
AmelOr158       VSSIRFLVFYAITISTFTLTLSGIIMIINC-PLFVKMEFITISISLLV------------ 284
AmelOr147       VNCIRFIIFYTMAVSTIVLTLSGIILITTS-SLLVKIQFLTICISILL------------ 284
AmelOr150       VIGFRFIILCAIGISTFALTLGGVIMIKKA-PFIVKVQFITLILTLLT------------ 286
AmelOr151       VISFRFIILYAIAVSTFVLILDGIIMIMKV-SLIVKVQFITLSLTMLT------------ 286
AmelOr152       VISFRFIILCAMGVSIFSLTLGGIIMITKS-PFIVKVQFITLILTLLT------------ 286
```

```
AmelOr154       INCFRYIVLYGIAVTTFALILCGIILLMNI-PLIVKIQFVIICITIMT------------ 286
AmelOr155       IKCFRYIVLYYITITTFALIISCIILLMNV-PLIVKMQFIIICVTIMT------------ 286
AmelOr157       VSCIRFLVFHAILLGTFVLTLCGIVLIINS-PLIVKAQFIIICVCILL------------ 283
AmelOr156       ANCFRHIIFYTIIATSFNMILAGIILIMNP-LLVIKIQFMITCFTALI------------ 286
AmelOr153       INWFRFIIFNAIGLSILVFTLASIILIMIS-ICMYIVVCSCMYNFINK------------ 262
AmelOr144       NATVETLLFKTNVTMGSTVICGAFPLLNNQ-SLDVVTQFLPLVLSGML------------ 287
AmelOr145       NATVETLIFKTNITMGSIVICGAFPLLNNQ-SLDVVTQFLPLILTGML------------ 288
AmelOr143       KATVETLLFKTNVTMGSTVMCGAFPLLNNQ-SLAAISQFLPLVLSGIL------------ 282
AmelOr1         GESLSIVFLSEMLGCTIIICFLEFGVIMELEDHKTLSTVTYFVLMTSI------------ 314
AmelOr3         GKSLSIVFLSEMLGCTIIICFLEFGVIVEWEDHKTFSMVTYFVLVTSM------------ 317
AmelOr6         ELITQYFCFYEISCNTCLICFIGYCIILEWENSNVVAIVVHFMLLGTC------------ 309
AmelOr7         ELITQYFCFYEISCNTCLICFIGYCIILEWENRNVIAIVVHFMLLGTC------------ 309
AmelOr4         ELITQYFCFYDIGCSTCLICFIGYSIIVEWENHNIASTVIYFSGLVTC------------ 311
AmelOr5         ELITEYFCFYDIGCNMCLMCFIGYSAILEWENHNIAAIVVHFMLLGTC------------ 311
AmelOr8         DQFVPYVYLIEIFVGVLITCILGYCIIVEWEDSDAMAIIAYVALQTTC------------ 311
AmelOr9         DQFVPYVYFIEMIVGVLITCVLGYCIIVEWEDSDAMAIIAYVVLQVTC------------ 311
AmelOr11        DQFVPAIYLIEVVIQVLIICIIGYCIIMEWEDSNAMAMVIYVVFQVTC------------ 309
AmelOr12        NQFVPSVYFFEIILEVLIICIIGYCLITEWEDNNTMATVIFVIFQITC------------ 311
AmelOr10        DQFVPTIYLFEVFIQVLIMCIIGYCIIMEWKESNGMGLITYVIVQMTC------------ 310
AmelOr14        EQLTTYLFLLEIMVETSIGCVIGYNVVTEWEDSNAAAMIIHLMMQVST------------ 313
AmelOr15        EHITTYLFLLEIIDETGIGCVIGYCAITEWEDSDATAAIIYLLLEASV------------ 314
AmelOr13        EQLTSYLYFCEILEYVCGACVIGYCLITEWENSNAAALIVYFILEFLC------------ 305
AmelOr16        ETVTEYICLIEMIGGTCLMCLVGYCILMELENTNTMAVVVYITLQISI------------ 316
AmelOr17        ETITEYICLIEMIGSTCMICLVGYCILMEWENTNTMAIVIYITIQISI------------ 316
AmelOr18        EDLLHEISLIEVVGCTLNICFLGYYSMMEWDSKQPVSGVTYIILLISV------------ 325
AmelOr19        EDLLREISLVEVVGCTINICFLGYYSMMEWDTEHLIRGMTYIILLTSV------------ 325
AmelOr22        EKALQQVSFVEFLGCMLNICLLGYYVITEWSSSHLTSAITFFILLISL------------ 326
AmelOr24        EKALQQVSFVEFLGCMLDICLLGYYVIMEWSSSHLTSAITFFILLISL------------ 318
AmelOr20        EKTLQQISFTEFLGCTLDICLVGYYVIMESKSNDVTSVITYIILLISL------------ 322
AmelOr25        EKALQQISFVEFLGCTANMCLLGYYLIVEWNPKEIILSVTYVALIISI------------ 317
AmelOr29        ERALQQISFVEFLGCTANMCLLGYYLIVEWNPKELIVSFTYIAIIASI------------ 318
AmelOr30        ERALQQISFVEFLGCTMNMCLLGYYLIVEWNPKEISLSLTYISLLISF------------ 318
AmelOr21        EKILQQVSYMEFLECTVNVCLLGYCAIIEWESNHLTEVVTYVIILITI------------ 318
AmelOr23        EKILQQVSYMEFLECTMNVCLLGYCAIMEWESNHLTEVITYLILLITI------------ 318
AmelOr35        EELLNEICFVEFIGCTLNICFLGYFLLKEWEQSETIGILTYCILLISF------------ 327
AmelOr27        ENIMNRIYFLELFRCTMIICIVGYYILTEWAEKNVQNLTTYFMMLLSI------------ 320
AmelOr28        EETINRIIFLEVFRCCLHICCLGYYILMEWSDYDKRSMIIYFMLFVSV------------ 320
AmelOr33        ENVMSGICFMELFKCTMNICMLGYYVLTAWIDNDMRNLIVCSVILFSM------------ 322
AmelOr34        ENVMSGICFMELFKCTVNICMLGYYILTAWTGHDIQSLIVFSVILFSM------------ 322
AmelOr32        ENVMSGICFMELFKCTVNICMLGYYILTAWSVHDIQNMVVFLVILLSM------------ 322
AmelOr31        EDVLNRFCFMELFKSTLDISMLGYYILTEWADHDIRNLTTYFMILTSM------------ 321
AmelOr36        EHLLSPICFMEMFKNILSICLFSYCILAEWSEHNIRILSTYILAVINI------------ 318
AmelOr37        EHLLSPICFMEMFKNILSICLFSYCILAEWSEHNIRILGTYIFAVINI------------ 319
AmelOr38        EHLLSPICFMEMFKNILSICLFSYCILAEWSEHNIRILGTYIITVINI------------ 320
AmelOr39        EHLLSPICFMEMFKNILSICMFSYCILAEWSEHDIRILTTYTFAVMNL------------ 320
AmelOr47        EHLLSPICFMEMFKNILSICMFSYCILAEWSEHDVRVLGIYAFAVICI------------ 319
AmelOr48        EHLLSPICFMEMFKNILSICMFSYCILAEWSERDIRVLGIYTFAVMNV------------ 319
AmelOr40        EHLLSPICFMEMFKNVLSICMLSYCILVEWSGRDIRALSAYTFSVMNI------------ 319
AmelOr43        EHLLSPICFMEMFKNILSICMLSYCILAEWYGRDVRVLGAYAFSVTCI------------ 320
AmelOr45        EHLLSPICFMEMFKNILTICMLSYCILAEWSGHDIRALSAYASAVMNI------------ 319
AmelOr41        EHLLNPIYFMEMFKNILTTCMLSYCILVEWSGHDIKVLSAYSFTITNI------------ 319
AmelOr42        EHLLNPIYFMEMFKNILTTCMLSYCILVEWSGHDIKVLSAYSFTITNI------------ 319
AmelOr44        EHLLSPICFMEMFKNILSICMISYCILAEWSGRDVRALSTYAFCVTCI------------ 319
AmelOr46        EHLMNPICFMEMFKCMVGMCMPSYYILAEWSEHNVQNLAVYVMIIISM------------ 318
AmelOr49        EHVMSPICFMEMIKCMVGICMPIYYILMEWSEHNIQNLTVYVMIIISM------------ 319
AmelOr50        ERITCPIYFMELFKCMMGMCMPSYYFLAEWSERNIQNLTIYVMVALSM------------ 321
AmelOr26        EKVMNMICLVELVGCTMNMCILKYYFLTEKSK---TILGIYAIVYASM------------ 324
AmelOr53        EEALREICFTEIIECTIDMCMLEYYCIMEWSVGDTITLLTFFTLLISF------------ 325
AmelOr54        EEALKEICFTEIIECTLNMCMLEYYCLIEWSAGDTITFLTFFTLLTSF------------ 323
AmelOr52        EEALREICLAEIIESTIIMCLLEYYCMTEWQNNDAIAILTYFTLLISF------------ 320
AmelOr51        ETTLRELFLVEVIVSTLLMCLLEYYCMVEWETSDSAAILTYVILLFSF------------ 322
AmelOr55        EKVLQEVFFLEFISSTFVICLLEYYCITDWEQNNIISLTSYALLLISL------------ 321
AmelOr57        ETVLQEVFFLEFVSSTFVICLLEYYCITDWEQKNIISLTSYILLLISM------------ 321
AmelOr56        ETILQEVFFLEFIGSTFVICLLEYYCIADWEQKNIISLTSYVLLLISL------------ 321
AmelOr58        ETVLQEVCFLEFIGSTFVICLLEYYCITDWQQNNTIGLTTYSLLLISL------------ 323
AmelOr59        QEVLQELCFLECIGSTFLICLLEYYCITDWELNNTISLTTYIILLISL------------ 324
AmelOr60        QVILQEVCFLEFIGSIFLICLLEYYCITDWELKNTISLTTYIILLISL------------ 324
AmelOr61        EMALQELCFLECIGSTFLICLLEYYCITDWELSNTISLTTYTMLLISL------------ 324
AmelOr63        NKTISSILIVQLSSSCMLICTTGFEFILALSIGNIVMIVKTFAVICVL------------ 310
AmelOr64        NETISSILIVQLFSSCVLICTTGFEFILALNIGNIVMTIKTFIVMCVL------------ 306
```

```
AmelOr66        NETISSILVIQLFSSCVLICTTGFEFILALSIGNIVMMIRICIAMCVL------------ 306
AmelOr65        NETISSILIIQLFSSCVLICTTGFQFILALSIGNIVLTIKILIIMCVL------------ 306
AmelOr67        NETVSSILVIQLFSSCVLICTTGFQLILTLTFGNVVLTIKILAEISIL------------ 306
AmelOr114       ENCFNVMLLIQILGCTVQLCFQCFQAIMSFGGGEAQEYLFFQLMFLLVYVF--------- 283
AmelOr115       EDCFNITLLVQTLICTAMFCLTGYRMITSVDREDEADVPIVGIIFFIIHVI--------- 306
AmelOr118       ETIFTYISLCQFVSNMLVICFISFVLTVSLHTEQTIVLIMKCLPYYIA------------ 285
AmelOr164       ENIFTFISLSQFFFNILVICFVNFILVTSIGTEQAPTVISKCFPYYIA------------ 297
AmelOr163       KYLFLYTSLIQFLSNILVICFLGFILVNALGTEQESTIFIKCFPYYIA------------ 299
AmelOr165       EKLFTYIALSQLVSNTLITCCVGFLIVIAIHEDNGLPLLLKSVLFYMV------------ 289
AmelOr166       EKFFTYIALSQLISNTLITCCLGYLIVITLHLGNNI-ILIKYIMFYVA------------ 288
AmelOr167       ESLFSYIALMQLICNTLIICCIGFLIVVALN--SNLKLLIRISFFYIA------------ 299
DmelOr35a       EAQYTVRVFIMFAFAAGLLCALSFKAYTNPMANYIYAIWFGAKTVELL------------ 314
DmelOr74a       EKEFTLRIFVMFAFSAGLLCALFFKAFTNPWGNVAYIVWFLAKFMELL------------ 315
AgamOr3         ETTFRWVFFVQFIQCTMIWCSLILYIAVTG----FSSTVANVCVQIIL------------ 322
AgamOr5         EIIFRWVFLGQFIQCVMIWCSLVLYVAVTG----LSTKAANVGVLFIL------------ 303
AgamOr13        ESITSLVLLQQLVLCVMIWSSMLLYFTVSG----FDLNFINLLVLFAF------------ 302
AgamOr15        ESITSLALLQQLLLCVLIWSSMLLYFTVSG----FNVNFMNLFVLFVF------------ 301
AgamOr17        ESITSLVLLLQLILCVLIWSSMLLYFTVSG----FDLNFISLIILFVF------------ 301
AgamOr16        HDITAPIMLLQLVLCIMVWSSMLLYFTVAG----FNTQFISLFILVMF------------ 299
AgamOr55        ETIVSPIMLMQIVLCVLMSSSMVLYFTFPT----MSGHMINVLLLFLV------------ 302
AgamOr18        ELVLQPLLLMQFVLCILIWCMMLLYFTVSG----LNVKFINMFLLFLF------------ 309
AgamOr14        VQALRPILMVQLVFCVFIWCLMMLFFTIADK---FSVAFFNLAILFVV------------ 323
AgamOr51        VKALRPVLLLQLVFCVLIWCLMMLYFTITDG---LSVKFFNIGIMFLV------------ 311
AgamOr12        NQTLSYVFALQLVLCMLTWCFTLLYVLVVR----LDVTSMMSLLIMIN------------ 312
AgamOr19        NQTLSYVFALQLVLCMLTWCFTLLYVLVVR----LDVTSMMSLLIMIN------------ 312
AgamOr20        NQTLSFVMTVQLALCILTWCFTLLYILTVG----LDVTGMNGLLIMFN------------ 312
AgamOr21        NQTLSFVMALQLALCILTWCFTLLYILIVG----FNAIATNGLLIMIN------------ 309
AgamOr50        NQTLSYVMALQLALGTLTWCFTLLYILIIG----LDVTAMMGLLIMMN------------ 311
AgamOr29        QEALNLSLLFQLTFCSLIWCLMMFYILLMG----FDSRILNVLILLLI------------ 300
AgamOr53        EQALNLSVLFQFASCSAIWCLMLFYILLMG----LDSRVLSVVLLLVI------------ 290
AgamOr30        QNALNPALLIQITFCTAIWCLMLFYILLLG----FTSKVMNVCLLLLV------------ 301
AgamOr46        QNILMEILLAQFTGCVFIWCFMLYYVMISG----ITAEGIAVGAMLIA------------ 301
AgamOr47        QNILMEILLAQFTGCVFIWCFMLYYVMISG----ITAEGIAVVAMLIA------------ 301
AgamOr48        DSILSLILLIQCVGCLLMLCLMLFYITRN-----HSLNVINIAVLLMS------------ 311
AgamOr49        DSVLNLMLLVQFTNTVLMCCLFLFYISKN-----FNSGAVNVLLLFLA------------ 318
AgamOr61        SSIVGPFSFVQYYGTLALIADCGF-ILSIEG---LSANGMIYLLFVTV------------ 321
AgamOr62        RSIVAPFAFLQYYSTFGLIADSFF-VVSFEG---FTGYSMAYVLFASF------------ 319
AgamOr63        KAVIGPIAFVQYYSTYLIIADCCL-ILVSHG---LSSFSIVYFISMLV------------ 314
AgamOr60        GSILKPFSFIQYYGSFTLIGYYCF-ILMYKG---VTPLTVVYIAFIVF------------ 316
AgamOr75        RTLLRNYSLVHFSFYMIIVATFMTRVLIIPG---RSSFGLAIPLLVTT------------ 320
AgamOr76        RTLLRNYSLVHFFFYMIIVATFMTRVLIIPG---RSSFGLAIPLLTTT------------ 320
AgamOr78        RTLLHNYSLVHFFFYMIIVATFMTRVLIIPG---RSSFGLAHPPVDDDD----------- 321
AgamOr77        KRHMNGSVLLELLFIVCLLALNGLRLCTTTT---TSDLSELALSSSMIL----------- 299
AgamOr79        KRHMNGSVLLELLFIVCLLALNGLRLCTTTT---TSDLSELALSSSMIL----------- 297
AmelOr99        NENCQYSYLIQVGLNMLGMSTTAVQTVINLD-RPDVAIRSAVFFGADQ------------ 320
AmelOr101       NENCQYSYLIQVGLNMLGMSTTAVQTVINLD-RPDVAIRSAVFFGANQ------------ 320
AmelOr102       NESNRNSYLIQVGLNMLAISATAVQAVVNLD-RPEEAIRSAVFCGANQ------------ 319
AmelOr103       NESNRNSYLIQVGLNMLAISATAVQAVVNLD-RPEEAIRSAVFCGANQ------------ 319
AmelOr104       NESSRNSYLIQVGLNMMGISVTAVQTVVNLD-RPEEAIRTAVFLGAEQ------------ 320
AmelOr98        NNSSQLSYLLQIGINIMGISTTAFQLAVNLDTRPQEAIRNAVFCGANQ------------ 317
AmelOr100       DESSRNTYLFLVGTNIMATSISAVQVVLNLD-KLEVAIKSAVFLIAAQ------------ 240
AmelOr97        NKSSRNSYFLQISLTIIGISIIAVQIVMYLH-KPEEAFRISLFLIAAQ------------ 264
AmelOr105       EKCCRNLYLIQMGLNIMIISVTCVEVVVFLD-RPKEAIRAIIYVIAQQ------------ 320
AmelOr109       DACHIHYFLLVIGMIVLAFTGTFLKLSTMEV--GIRFFTFCAYTIAQL------------ 308
AmelOr110       DACHIYYFLLTIGMIVLTFTGTFVKLSTMEM--GIRFFTFCAYIAAQL------------ 307
AmelOr111       DACHIHYFLLIIGMIVLAFTGTFVKLSSMEV--NVRFFTFCAFTVGQL------------ 308
AmelOr108       DACHIYYFLLIIGMIVLAFTGTFLKLSTMEI--EIRFFTFCGYTVAQL------------ 308
AmelOr107       DACHIHYFFILLGMIIVTFTGTFIKLTSMEI--GGRFFTFCTFTIGQL------------ 308
AmelOr112       ENSYHTYLFVSMGLLIMAISVSLLEVANGKN--GSRELVQATFLFAQL------------ 305
AmelOr113       EDTHVNLLFISLGLIIMCFSITLLKVVTMDY--CLDFYKYSSFLIVQL------------ 306
AmelOr106       EDFYSMNIFAQVGASILCLTVTLMKIATIKW--SMETNQYYGFVIAQV------------ 304
AmelOr122       IDLKIKDIHRVVDIHCHAIELLYKALITMDN--RIEIFGSTLIVIYHL------------ 279
AmelOr125       IDLKIKDIHRVVDIHCHAIELLYKALTAMDD--RMEILGSTLIVIYHL------------ 278
AmelOr123       TDLKLRDIHRVVDIHCHAIELLYKALVTTDN--RMEILGSTLIVIYHV------------ 276
AmelOr126       TDLKLRDIHRVVDIHCHAIELLYKALITMDN--RMEILGSILIVIYHL------------ 276
AmelOr127       IDLKLRDIHRVVDIHCHAIELLYNALITMDN--RVEIFCSTIVVTYHL------------ 276
AmelOr128       TDLKFRDIHRVVDIHCHAIELLYKALITMDN--RMEIFGCILVVAYHL------------ 276
AmelOr129       TDLKLRDIHRVVDIHCRTIELLYNALIIMNN--RMEIFGSALMVMYHL------------ 276
AmelOr133       IESKLAELYCVVDIHNQAIQLLVNAITIKKD--QLEILISLIIFVNHL------------ 181
AmelOr134       IDSKLAELYRVVDIHNQAIQLLVNAITIKKD--QLEILISLIIFTNQL------------ 277
```

```
AmelOr131       IDSKLAELHRVVDIHNQAIGLVVNAITIKKD--QLEILITLIIFANHL------------ 279
AmelOr132       IDSKLAELYHVVDIHNQAIQLLENAIIVTKD--HLEILICLMLFVKQL------------ 279
AmelOr135       IETKLAELYRVVDMHNQAIELLVNAIIIKKD--QLEISISFIFFVNQL------------ 277
AmelOr136       IDSKLAELYRIVDIHNQAIELLLNAIVIKKD--ELEILISFIFFTTQL------------ 279
AmelOr138       IDMKLTELYRAVDIHNQAIGLLVNAIIVKKD--QLEMLISFMILMAQL------------ 185
AmelOr130       IDSKLIELYRVVDIHNQTIELLVNATLIKKN--QLEMLFCFTLVAIHL------------ 279
AmelOr137       IDTKLTELYRAVDIHNQTIGL--------------------------------------- 253
AmelOr139       INSKLVELYHVVDFHNQVIELLVNMILIRKD--QLEIFMFFIFLVSQM------------ 201
BmorOr20        SEAFGPMLFVYYLFHQVSGCLLLLECSQLNTAALVRYGVLTVVLYQQL------------ 357
BmorOr3         SEAFGPMLFVYYLFHQVSGCLLLLECSQLNTAALVRYGVLTVVLYQQL------------ 357
BmorOr18        SEAFGPMLFVYYLFHQVSGCLLLLECSQLNTAALVRYGVLTVVLYQQL------------ 357
HvirOr6         SDVFGPMLFIYYAFHQASGCLLLLECSQMTARALMRYLPLTIIMLQQL------------ 347
BmorOr23        SETYGLAVLIYYAFQQVVGCLLLLQCSQMELKTVTRFGFLTLVLNQQL------------ 334
BmorOr16        SETYGLAVLIYYAFQQVVGCLLLLQCSQMELKTVTRFGFLTLVLNQQL------------ 334
BmorOr4         SETYGLAVLIYYAFQQVVGCLLLLQCSQMELKTVTRFGFLTLVLNQQL------------ 333
BmorOr5         SETYGLAVLIYYAFQQVVGCLLLLQCSRLDLKTITRFGFLTTMVNQQL------------ 334
BmorOr22        SNVYDVVIIVYYLFHQVTGCLLLLQCSTLDWESLSRYGPLTLIIFQQL------------ 341
BmorOr17        SNVYDVVIIVYYLFHQVTGCLLLLQCSTLDWESLSRYGPLTLIIFQQL------------ 341
BmorOr1         SETFGVILLVYYGFHQVSLCMLLLECSDLSTKAMLRYGPLTLIMIQQL------------ 348
BmorOr1a        SETFGVILLVYYGFHQVSLCMLLLECSDLSTKAMLRYGPLTLIMIQQL------------ 348
BmorOr1b        SETFGVILLVYYGFHQVSLCMLLLECSDLSTKAMLRYGPLTLIMIQQL------------ 348
BmorOr19        QSAFGLNVASNYLQNLIEDGLFLYQIMNGEKENVLMYGLMIILYLGGL------------ 326
BmorOr15        QSAFGLNVASNYLQNLIEDGLFLYQIMNGEKENVLMYGLMIILYLGGL------------ 326
TcasOr5         NETFGELMLLRFSG-----DFIYLIVGLSS------------------------------ 246
TcasOr7         SSFIVVLWLLFVVHSIVTISFVFGKVKEQHQ----------------------------- 259
HvirOr4         DDSYGLPLVVILISTLLHLIVTPYFLIMEIIVST-----NRIHFLVLQFLW--------- 302
BmorOr13        DNNNGIIVLLLLASFLLHLVVTPYYLIISFVTESPHTGFEKVLNPILQTVW--------- 349
DmelOr10a       RDRYTIITLAHFVSAAMVIGFSMVNLLTLGNNGLGAMLYVAYTVAALS------------ 324
DmelOr33c       SRTISEVQLVQLGGCGATLCIIVSYMLFFVGDTISLVYYLVFFGVVCV------------ 297
DmelOr7a        SPVISRTIFVQFLITAAIMGTTMINIFIFANTNTKIASIIYLLAVTLQ------------ 322
DmelOr22c       TRQFTVIVLMHFLSAAFVLCSTILDIMLNTSSLSGLTYICYIIAALTQ------------ 321
AgamOr1         QDIFQGSIFAQVCASVIIICMTLLQATGDDVTMADLLGCGFYLLVMTS------------ 330
AgamOr4         NTSIRSMLMLQWLTCVLNWSISLIYLTNVGISLQSVTVVVMFFLATAE------------ 309
DmelOr47b       NRAFNGAFNAQLMASFSLISISTFETMAAAAVDPKMAAKFVLLMLVAF------------ 327
AmelOr73        NTVFQYMIFLQFTISSVVLCLSIYKFSTVDPLSMNFVWSGFYLCCMLM------------ 280
AgamOr68        SDLWSMINLAQVGCSVFGICINMLIIFLTDWYAAYAILFALFIDLSVH------------ 311
DmelOr59a       EAFISLPMLIQFTVTALNVCIGLAALVFFVSEPMARMYFIFYSLAMPL------------ 295
DmelOr23a       KSLVSYPMMVQFMVIGINIAITLFVLIFYVETLYDRIYYLCFLLGITV------------ 292
AgamOr31        INAYKNVILNQLLISSVLLCMLGFQLVISVGTNIMVVYVAYGMAITIQ------------ 290
DmelOr2a        QRVLSVPCMAQFVCSAAVQCTVAMHFLYVADDHDHTAMIISIVFFSAVT----------- 313
AmelOr62        EQTFNMIIFVQLLMSALLICVEGFVFLVCLSTKDNIGALKSMVLMVTL------------ 312
AmelOr121       KMFVEQIINLIITIFKYFFKCLKMYIEEEQFSLFRIIYLICNFTNTFL------------ 310
AmelOr140       ESLFTKSILVQLFFNVLCLSITGVETVIKLGNLSEMMRFGSFTFAQAV------------ 276
TcasOr12        TNLFTTCITAQFVVGVIIVCMSMFQMSLVSVLSFQFAAMLLYQICVLM------------ 306
AgamOr8         EHMFSPSLLVNFLSSSVIICLVGFQATAGITPADLFKFVLFLVSPLVQ------------ 309
BmorOr9         ENIFTAPNLFNVLIGSVEICALGFNLMIGDLTQIPGCILFLSSVLLQI------------ 296
AgamOr40        ENCYHPYVLLKLLQILLLLCFLSFMATVESLSTMKLINVLEYFMLSIT------------ 371
TcasOr63        EAIFSKGIFLQLFGSVIVICTTGFQLIVVPIPSVQFAVLGTYLCGMTT------------ 297
TcasOr4         FELWQIKPKIDSCKFIAKIFRILCILVVLFYTLVPYLDDKEDLSLPLPG----------- 293
TcasOr13        NDSFGLLILIVISGCLVHLLATPYALYAIIFTTGNTMFIITQSIWMTG------------ 318
AgamOr24        VDFLNPYHFVTLISTVPTMVISVLGLVLLDWYPGAAIVFLG------------------- 289
AgamOr54        CSLFELHFFIVFGCIFFQLVSNVVVIVSVP-----DWYPGYFLFIMLT------------ 280
AgamOr23        DKLLKKQFFINFGCMIFELVASLAIVVRVPWYPGMAICLICTNQLFIN------------ 303
AgamOr64        CKVFSPISVVLYYGALITITCTCFYVMKHDVSTTTVCYAGFAVYMLFN------------ 324
DmelOr82a       RSIYTPTVMGQFVITSLQVGVIIYQLVTNMDSVMDLLLYASFFGSIML------------ 302
AmelOr116       QDIFGIIIFFQLFVNCIIVCLAAFNLSQIKNYITPEFFGSLLYICCMIY----------- 315
AmelOr160       ANTFSSLMFVQTLGLIFSLCIVVYQLLMTSESGEDMNTIHFIIYSCAV------------ 305
TcasOr10        SEISDEIFHSDWSDDQVYKNKTARLIVMNRAQRPAKISIGKFLDLNLR------------ 272
TcasOr26        NDTYRYSFSVIVLCAGTLFGCIGYTVMKFHS----------------------------- 273
AmelOr117       NNLFTPIIFIQLLTSGIEICLSGYAVLDNNSANADLLKFISYFISMGI------------ 315
AmelOr120       EKNFHLILFLQAITSSLIICFVGFQISIALTERSKFLESFSHLIVSLF------------ 317
BmorOr7         QSIFSSAILIQFGIGAWILCMAAYKIVNLSVLSIEFASMILFISCILT------------ 307
AgamOr6         LDAGVNQWRNGYMLVKFVLFMLCFLIELLMLCAYGEDIVESVRHQAVMS----------- 319
DmelOr45a       SSIFGEIVFVKYMLSYLQLCMLAFRFSRSGWSAQVPFRATFLVAIIIQ------------ 295
DmelOr67d       KKIYSIVLFVQLSTTCVGLLCTISCIFMKAWP----AAPLYLLYAAIT------------ 307
DmelOr83c       TDYCRAINALYYELIATQVLSMALAMMLSFCINLSSFHMPSAIFFVVS------------ 312
AmelOr162       EDTNTTSYLLQLGFNMICISFTQFQAIINIEDTPKVLRYVSITIALLC------------ 285
HvirOr3         ESIFKEYFLVQNLAVTVELCLNAVMVTVVGVHQITLLVSFVAYLLLALVN---------- 186
TcasOr20        DLAFNQMMLLHITWTGFIISVLGFEISTTDDYVEAFRFFMHLLGWLGM------------ 303
AgamOr11        EQLTRAAVFLDFVVFSVLLCALLFEASMTPSGVQVFIDVCYITTMTTI------------ 341
```

```
DmelOr56a       QYLICVPVMADFIIFSVLICFLFFALTVGVPSKMDYFFMFIYLFVMAG------------ 335
AgamOr28        EDGFSFFLMAQFLTSSILVCVVLYELTMVFGWNEDTFKTVTYLPGAIL------------ 335
AgamOr58        RNNLKLYLLLNLVADFSLITFAGCQMVISSEGDQHLYSILAALTACLN------------ 303
AgamOr59        QRMASFVFLQHHIFSLGLVVAGSYVTLRGPALRENIVLSEYPISVVLEY----------- 324
DmelOr1a        NYSFMEIAFVEVVIICGLYCSVICQYIMPHTNQNFAFLGFFSLVVTTQ------------ 304
DmelOr13a       EDFFNPILLANLMISSVLICMVGFQIVTGKNMFIGDYVKFIIYISSALS----------- 316
AmelOr161       ERIINLLALGQFVISTGLICFAGFQITSMMEDKGRLMKYSTFLNSAIL------------ 302
AmelOr141       EHLTSGMFFIQVIASTYNISLVGFKLLEDTPDKFKYITQLIILIIQLF------------ 349
AgamOr52        NRVHSFSITVQYYTALLSLAIDAFFISYYGLNFVSVCVSIFSVLLVFE------------ 291
AgamOr36        KPFLQTTFLVMFYSAALFLAIGTFMISANGTTTYGVILSGFLFALLLE------------ 312
AgamOr25        EQTYYIDLLVDFGLVCLILCMGLNVIADEVINAIWFFLIAVVFQLSLL------------ 283
AgamOr45        YETYQMHFFSMFSVLCFVICMCMNVVARDPR-----STLIPFGLASTG------------ 304
TcasOr3         ESIYSTQTFVQTFISLGEMCFSLYLLSETADQNIGNEITYLIATGFEL------------ 328
DmelOr88a       NDIFKVAFLVSNFVGAGSLCFYLFMLSETSDVLIIAQYILPTLVLVGF------------ 320
AgamOr22        SSIYREVYLMQVLCSIVHICVSLFHIQIKFK-----NGSYGMLLTNVN------------ 303
DmelOr49a       NYVFGLLLASNLFTTSCLLCCMAYYTVVEGFNWEGISYMMLFASVAAQ------------ 312
DmelOr85f       NGIFGKSLLLSLLTTAAVICTVAVYTLIQGPTLEGFTYVIFIGTSVMQ------------ 309
AmelOr119       NKIYSLSMLVQMISSTSIICLTGFQAVVVGGQSSNIMKYGIYLSAAMS------------ 329
DmelOr63a       NNCFRHITFTQFLLSLFNWGLALFQMSVGLGNNSSITMIRMTMYLVAAG----------- 334
DmelOr67b       ENLFKYIILVQCSVSSILICMLLYKISTVLEVGWVWMGMIMVYFVTIA------------ 335
BmorOr21        QDTFNIWLGLIYVATMIQMCTCMYHIVQSFNIDVRYIIFVISIIHIYLP----------- 313
BmorOr11        KVNFSILYEYNMKITTISMPLSAFQVVESLRRGEFNIEFTYFFFGCIL------------ 313
BmorOr12        QIMCRTVFSANIMLDTFVLVILMLAMVNSENDFYGLCSQMSSVLVTVV------------ 317
AgamOr33        KRLYEPNIMAQFVCSMLIICLTAFELMFAKGDPMQMVRFGAYMLAGFY------------ 289
HvirOr1         DEVISGVVFISFANNLFFICLQLFNTLEDGLKGTGECTQLNSQSKLKKIVVSKSGPLGGH 339
HvirOr5         DVALGGIVILSCSCNFYFICLQMFLGITQGLSSDLLSLIYYVISLAWLCT---------- 249
TcasOr1         RVFFTVFNVVHCYLASELVLMLKNRFVTLNVQLTKLTKNCATKAQSVVLG---------- 267
TcasOr2         NEIYGVPLLLFFGLNFLIITQAMFLVVGQLQASQIHWQKIIIIVMSSV------------ 290


TcasOr16        -------------LAQVFHFCIFGNRLIEESSSVMEAAYS---CHWYDGSEEA------- 427
AmelOr2         -------------LGQVFMLCIFGNRLIEESSSVMEAAYS---CHWYDGSEEA------- 430
AgamOr7         -------------LAQVFLFCIFGNRLIEESSSVMKAAYS---CHWYDGSEEA------- 430
AaegOr7         -------------LAQVFLFCIFGNRLIEESSSVMEAAYS---CHWYDGSEEA------- 414
DmelOr83b       -------------LAQVFHFCIFGNRLIEESSSVMEAAYS---CHWYDGSEEA------- 438
BmorOr2         -------------LGQVFHFCIFGNRLIEESSSVMEAAYS---CQWYDGSEEA------- 424
BmorOr2a        -------------LGQVFHFCIFGNRLIEESSSVMEAAYS---CQWYDGSEEA------- 424
HvirOr2         -------------LGQVFHFCIFGNRLIEESSSVMEAAYS---CQWYDGSEEA------- 424
AgamOr56        -------------IFQTFSFSYLGTELIEQSEAVADAIFH---SKWYTQKLNR------- 347
AgamOr57        -------------IFQTFSFSYLGTELIEQSEAVADAIFH---SKWYTQKLNR------- 347
AgamOr26        -------------MFQMFSFSYLGTELIEQSEAVADGIFN---SKWYEEDVKV------- 347
AgamOr27        -------------IFQTFSFSYLGTELIEQSEAVADAIFH---SKWYTQKLNR------- 347
HvirOr9         -------------LMQIFLYCYYGDTIMRSSMQVSTSIYN---SPWYNIRAAD------- 350
HvirOr7         -------------LMQVFFLCFFADLMMTASLEISNAVYN---CRWYSANIKV------- 334
DmelOr85b       -------------MSQVYLICHYGQLVADASYGFSVATYN---QKWYKADVRY------- 340
DmelOr85c       -------------LSQVYLICHYGQLIADASSSLSISAYK---QNWQNADIRY------- 339
DmelOr85d       -------------MVQVFMIATHAQRLVDASEQIGQAVYN---HDWFRADLRY------- 362
DmelOr67a       -------------LLEVYLLCSFSQRLIDASENVGHAAYD---MDWLGSDKRF------- 358
DmelOr67c       -------------MVQVFMVCYYGDTLIAASLKVGDAAYN---QKWFQCSKSY------- 353
DmelOr92a       -------------LVQVFVVCYYGDEMISSSSRIGHSAFN---QNWLPCSTKY------- 358
DmelOr69a       -------------VLYNFVICYMGTEVTLASGKVLPAAFY---NNWYEGDLVY------- 345
DmelOr69b       -------------ITYNFSMCRSGTHLILTSGKVLPAAFY---NNWYEGDLVY------- 345
TcasOr6         -------------VLELIVNSQGLETYARASDTLIPQCQL---VCKIFVLAKY------- 324
TcasOr11        -------------------DWGKNQAYKYKTD---------------------------- 285
AgamOr41        -------------TMQLFLFCLLGTIIEDKGVKFSAGVYS---LTWNELSKQD------- 335
AgamOr42        -------------VMQMLILCSLGTLIELKSDQFKDQLYD---IAWPEMDLPE------- 340
AgamOr43        -------------VIQLFELCLLGTILSIKNEEIEHAFYD---SLWYLMDHSE------- 337
AgamOr44        -------------VVQLFELCLLGTILSIKNEEIEHAFYD---SLWYLMDHSE------- 337
AgamOr66        -------------VFQLFVYCLMGTVMQIMNERMIDYISN---LPWYMLPTEE------- 341
AgamOr67        -------------VFQLFVYCLMGTVMQIMNERMIDYISN---LPWYMLPTEE------- 341
AgamOr72        -------------FVQLSGFFVIGHLVELKIDAMYNKIIS---MPWYKLPLKE------- 337
AgamOr73        -------------FVQLSGFFVIGHLVELKIDALYNKIIS---MPWYRLPVKE------- 337
AgamOr71        -------------FVQLSGYFVIGHLVEIKIDALYNKIIS---LPWYKLPVKE------- 337
AgamOr74        -------------FVQLSGFFLIGHLVELKNDALYNKVIS---MPRYKLPVKE------- 337
AgamOr70        -------------FIELSLYFLIGHLIEQKIDAMYNKIIS---MPWFKLPVKE------- 337
AgamOr69        -------------FVELSIYFVIGHFVELKIDEMYNSIIS---MPWYKLPVEE------- 341
TcasOr9         -------------SIELLIYTYSGQVLTQNADIVG-TLYE---SPWYMCDVHF------- 246
TcasOr18        -------------STELFIHTYSGQILTENADFLW-TLYE---CPWYLCDVTY------- 357
DmelOr83a       -------------LYELFIICYFADIVFQNSQRCGEALWR---SPWQRHLKDV------- 402
DmelOr85e       -------------IFELLMFTYCGELLSRHSIRSGDAFWR---GAWWKHAHFI------- 411
```

```
TcasOr24        -------------ITAIFVSSFGGQILIDSSLSVAEAAYS---SAWYEADIMK------- 333
TcasOr25        -------------STATFIACYGGQLLMDASTSIADEFYN---CPWYEADVKM------- 333
TcasOr19        -------------IISLFTSCLAGQILLDSSTSVADALYN---SQWYTADVKL------- 298
TcasOr22        -------------QAGFAAFSSAVTRVISESLTIPDAALD---SKWYEAPIKS------- 334
TcasOr23        -------------ICSFFICCHAGQAVISESLTIPEAALD---SHWYEAPVKY------- 331
AmelOr168       -------------LIQFYILCYCIQDLFEASTSIADDVVY---EKWYSYDVRF------- 340
AmelOr170       -------------LIQFYILCYCIQRLFEASSSIADDVVY---EKWYYYDVRF------- 347
AmelOr169       -------------LIQFYILCYCIQQLLEASTTVADDVVH---EKWYLHDVKF------- 353
DmelOr65b       ------------ALGHLSMWSYCGDQLSQKSLQISEAAYE---AYDPTKGSKD------- 335
DmelOr65c       ------------ALGHLSMWSYFGDLLSQKSLTISEAAYE---AYDPIKGSKD------- 335
DmelOr65a       ------------TLGHLSFWSKFGDMFSKESEQVALAVYE---AYDPNVGSKS------- 367
TcasOr17        ------------------------------------------------------------
TcasOr21        ------------------------------------------------------------
TcasOr56        ------------MLFQLLCYCWHGNEVQIVSGELSRYAFG---INWIIM-RES------- 383
TcasOr15        ------------------------------------------------------------
AgamOr9         ------------LVIQVVQYCYFGNEINYMAQKVHQATAF---VNYPDM-NIK------- 366
AgamOr65        ------------LVIQVAQYCYFGNEINYMAQKVHRATAF---VNYPDM-NIK------- 369
BmorOr6         ------------ILGQLYLYCWCANELTTKSEQLRDTLYL---TPWYDQ-DMK------- 339
BmorOr10        ------------MLSQLFQYCWCGHELTIRGEELRETLYQ---SPWHEQ-DIR------- 336
AgamOr34        ------------MTMQIGLPCYYGNEVTLKSYALTNAIYS---SRWYDM-PQS------- 328
AgamOr37        ------------MIFQMFLPCRFGNEVTRKSHLLRTSIYS---SRWYEM-GLQ------- 340
DmelOr94a       ------------MILQIYLPCYYGNEITVYANQLTNEVYH---TNWLEC-RPP------- 337
DmelOr94b       ------------MIVQIFLPCYYGNELTFHANALTNSVFG---TNWLEY-SVG------- 333
DmelOr71a       ------------MIMQVMLPTIYGNAVIDSANMLTDSMYN---SDWPDM-NCR------- 328
DmelOr46a       ------------MLIQIFILCYYAGEVTQRSLDLPHELYK---TSWVDW-DYR------- 330
DmelOr46b       ------------MLWQIFIICYASNEVTVQSSRLCHSIYS---SQWTGW-NRA------- 333
HvirOr8         ------------MILQILVPCWFGQRIIDKSNLLAFSAYD---CEWTSE-TRQ------- 345
BmorOr8         ------------MTLQIFVPAWMGTQLHFQSQELVFAAYN---SEWIPR-CQS------- 342
AmelOr68        ------------MMTQIFVYCWYGNQLKLKSVEVVDTIFE---LDWISL-DNR------- 324
AmelOr69        ------------MLTQIYIFCWYGNEVKLKSLDISNMIFE---LDWPDL-DNT------- 319
AmelOr70        ------------VLVQILYYCWFSNEVKLKSLEVPGMIFK---SDWTSW-DDK------- 321
AmelOr71        ------------SLMQIFYYCWFSNEVKLKSLELSDMIFR---SNWTSL-NNN------- 320
AmelOr72        ------------ALIQIFYFCWHGDIAKYKSLEIPDMIFN---SNWPNL-SNE------- 321
DmelOr19a       ------------LTTETLLLCYTAELPCKEGESLLTAVYS---CNWLSQ-SVN------- 333
DmelOr19b       ------------LTTETLLLCYTAELPCKEGESLLTAVYS---CNWLSQ-SVN------- 333
DmelOr33a       ------------MLIELFPSCYYGILMTMEFDKLPYAIFS---SNWLKM-DKR------- 328
DmelOr33b       ------------MVLELFPCCYYGTLISVEMNQLTYAIYS---SNWMSM-NRS------- 330
DmelOr22a       ------------VSMETFPFCYLCNMIIDDCQEMSNCLFQ---SDWTSA-DRR------- 342
DmelOr22b       ------------VSMQTFPFCYLCNMIMDDCQEMADSLFQ---SDWTSA-DRR------- 342
DmelOr42b       ------------ILLQTFPFCYTCNLIMEDCESLTHAIFQ---SNWVDA-SRR------- 342
DmelOr59b       ------------ILLQTFPFCYTCNMLIDDAQDLSNEIFQ---SNWVDA-EPR------- 343
DmelOr59c       ------------ICTESFPCCMLCEHLIEDSVHVSNALFH---SNWITA-DRS------- 346
DmelOr98a       ------------LMVQTFPFCFVCDLLKKDCELLVSAIFH---SNWINS-SRS------- 340
DmelOr42a       ------------VLLETTPFCILCNYLTEDCYKLADALFQ---SNWIDE-EKR------- 345
DmelOr85a       ------------ILSQTFPFCYVCEQLSSDCESLTNTLFH---SKWIGA-ERR------- 340
DmelOr43b       ------------VLLQTFPLCFYCNAIVDDCKELAHALFH---SAWWVQ-DKR------- 345
AgamOr2         ----------------MFAFYWHANEVLEQSLGIGDAIYN---GAWPDFEEP-------- 328
AgamOr10        ----------------IFAFYWHSNEVREESMAIAQASYS---GPWLNVDDT-------- 325
DmelOr30a       ----------------SVVLYYVANELYFQSFDIAIAAYE---SNWMDFDVD-------- 327
DmelOr49b       ----------------ILALYWYANELREQNLAVATAAYE---TEWFTFDVP-------- 325
DmelOr43a       ----------------LFTYYNRANEICLENNRVAEAVYN---VPWYEAGTR-------- 325
AgamOr32        ----------------AAIYTYHGSAISAKSVSVADAIYG---TNWYDAPLAV------- 329
AgamOr35        ----------------AVIYCYHGSMMYDESLKVADAIYQ---SNWYEAPPAE------- 339
DmelOr47a       ----------------AYIYCYCGENLKTESASFEWAIYD---SPWHESLGAGG------ 333
DmelOr98b       ----------------VSIYCFCGSSIHSECQLFGQAIYE---SSWPHLLQEN------- 332
DmelOr9a        ----------------LFIYSKCGENIKSASLDFGNGLYE---TNWTDFSPPT------- 344
AgamOr38        ----------------LFMYSYGGTVVRESSEAIQTVAYG---FPWYQFDRNT------- 360
AgamOr39        ----------------LFLYSYSGTIIRDSSEAVQTVAYD---FPWYRYDRNT------- 361
DmelOr24a       ----------------IFLYCLGGSHIMEACSNLARSTFS---SHWYGHSVRV------- 340
DmelOr45b       ----------------IFLYCYGGTEMSTESLSLGEAAYS---SAWYTWDRET------- 349
AmelOr74        ---------------HIIFFTYICHGLMEESGNVGLATYS---GWWTTLPMNE------- 345
AmelOr86        ---------------HIIFFTYICGGLIEESSNIGLATYS---GWWTVLPMDE------- 344
AmelOr75        ---------------QLLIFTYSCDNLIRQSVNVGNAVFS---GPWVNLPMSK------- 344
AmelOr84        ---------------QLLMFTYSCDDLIRESVNVGNAVFS---GPWAELPMDK------- 341
AmelOr87        ---------------QLLLFTYSCDSLIQESTNVGSAVYS---GPWICLPMNR------- 369
AmelOr76        ---------------QLLMFTYSCDCLIRDSMDIADAAYN---CSWSFLPMDK------- 347
AmelOr85        ---------------QLLMFTYSCDCLIRDSTNIANATYN---SLWSFMPMDK------- 352
AmelOr88        ---------------QLLMFTYSCDCLMHDSMSVANAAYN---SLWPCLPMDK------- 350
AmelOr91        ---------------QLLMFSYSCDCIIRESVSVATAAYG---GPWTLLPMTI------- 351
AmelOr92        ---------------QLLMFSYSCDCILRESVSVATAAYE---GPWTLPPMTI------- 348
```

```
AmelOr95        ---------------QLLMFTYSCDCIIRESLSVATAANR---GPWPMIPMTT------- 347
AmelOr93        ---------------QLLMFSYSCDCIIRWTLX-------------PLLSMTS------- 325
AmelOr77        ------------------------------------------------------------
AmelOr94        ---------------QLWMFTYSCDCMTRESVNVASAVYC---IPWTRIPMDK------- 346
AmelOr96        ---------------QLWMFTYSCDCITQESAKIASAAYA---SPWINLPMDK------- 346
AmelOr79        ---------------LLFMVTYSCNGVTEHSDNVAIGAYS---ALWTIVPMDK------- 346
AmelOr83        ---------------LLFMFTYSCNDVMEHSDNVAIGAYS---ALWTILPMDK------- 351
AmelOr80        ---------------LLFMVTYSCNGVIEHSDNVAVGAYS---ALWTIMPMDK------- 349
AmelOr81        ---------------LLFMVTYSCNGVIEHSDNVAIGAYS---ALWTIMPMDK------- 346
AmelOr82        ---------------LLFMVTYSCNGVIEHSDNVAIGAYS---ALWTIVPMDK------- 348
AmelOr78        ---------------LLFMFTFSCNGVMEQSDNVAVGTYS---ALWTVMPMEK------- 348
AmelOr89        ---------------LLFMFTFSCNCVTEHSENIAIGAYS---ALWTAMPMDK------- 345
AmelOr90        ---------------LLFMFTYSCDGVIEQSDNVAVGAYS---ALWTIMPMDK------- 346
AmelOr142       ---------------ELFMYAWPADSLMHMTMKMATKVYN---MDWYGKDIR-------- 320
AmelOr146       ---------------AVFMCAWPADYLMDVSENTMRAVYE---SEWYKRSLK-------- 320
AmelOr148       ---------------EIYMYAWPADHMQDASINILRSAYN---SIWYEQSLD-------- 317
AmelOr158       ---------------QIYIYAWPADYMQDMSINVLRSAYN---SIWYEQTLD-------- 318
AmelOr147       ---------------EIYMYAWPADHMYDMSITVLQSVYD---SMWYGQTLN-------- 318
AmelOr150       ---------------EIYIYTWPANHMKDMSINVSQSIYN---ITWYKQTLR-------- 320
AmelOr151       ---------------EIYIYAWPADYMKDMSTNVSKSVYN---ITWYKQTLR-------- 320
AmelOr152       ---------------EIYMYAWPADHMKDMSINVSKSVYN---TIWYEQTLR-------- 320
AmelOr154       ---------------EVYMYAWPADYVKNMSINISRSVYE---LSWYEQTIE-------- 320
AmelOr155       ---------------EIYIYAWPADYVKNMSINISKSVYE---LSWYEQTLE-------- 320
AmelOr157       ---------------EIYLYALPADYMYDMSMNISRSVYD---SIWYEQRLD-------- 317
AmelOr156       ---------------EVYLYAWPAQYMDDMSKNVSISAYN---LKWYEQTSE-------- 320
AmelOr153       ---------------NYYMYIWPADYMTDKSINVSRKIYD---SMXYKQMLK-------- 296
AmelOr144       ---------------HLFVISWPADDLRESSIQFAESIND---IQWLGQSKK-------- 321
AmelOr145       ---------------HLFVIAWPADDLRESSIQFAESIND---IQWLGQLKK-------- 322
AmelOr143       ---------------HLYVIAWPADDLRESSVQFSNSISD---IQWLGQSNK-------- 316
AmelOr1         -------------FVNVFIISFIGDRLKQESERIRETSYF---IPWYDFPTE-------- 350
AmelOr3         -------------FVNVFILSFIGDRLKQESERIGQTSYF---LPWYEFPTE-------- 353
AmelOr6         -------------ILVTYIVCYIGQLLIDESNNLARTCIT---LNWYHFPTR-------- 345
AmelOr7         -------------IFVTYIVCYIGQLLLDESNNLARTCIT---LNWYHFPTR-------- 345
AmelOr4         -------------TLMIYIICYIGQLLLDESNNLAQTCIT---LNWYRFPKK-------- 347
AmelOr5         -------------IFIIYIVCYIGQLLLDESNNLAQQCIT---LSWYHFPTR-------- 347
AmelOr8         -------------VFGTFSICYVGQLLVDESESVRQACKT---LKWYRLPTK-------- 347
AmelOr9         -------------VFGTFSICYAGQLLVDESENVRQACNT---LKWYRLPTK-------- 347
AmelOr11        -------------VIGTFSVCYVGQLLLDESENIRQAYNT---LNWYRLPVK-------- 345
AmelOr12        -------------FIGTFAVCYAGQLLVDESENVRQACST---LNWYRLPVK-------- 347
AmelOr10        -------------LIGSFSVCYVGQLLIDESENIRQAFIA---LKWYQLPVK-------- 346
AmelOr14        -------------ISCTFIMCYVGQTLIDEGNNVRRMSIT---LDWYRFPVK-------- 349
AmelOr15        -------------FGVTFTMCYVGQILIDEGNNVRRMSIT---IDWYRFPAK-------- 350
AmelOr13        -------------IFCTLTICYIGQLLIDESDKVRQISVT---LDWYRLPVN-------- 341
AmelOr16        -------------IFCVFILCYIGQMLVDENYIVSQASST---INWYRLSIK-------- 352
AmelOr17        -------------IFCIFILCYIGQLLVDENYIVSQASST---INWYRLSIK-------- 352
AmelOr18        -------------TFNIFIFCYIGQLLAEQTVKVGEKSYM---IDWHRMPWK-------- 361
AmelOr19        -------------TFNIFIFCYIGELLAEQTVKVGEKFYM---IDWYRMPWK-------- 361
AmelOr22        -------------TFNIFIFCYIGELVAEQCKKIGEISYM---VDWYRLEGN-------- 362
AmelOr24        -------------TFNIFIFCYIGELVAEQCKKVGEISYM---VDWYRLEGN-------- 354
AmelOr20        -------------TFNIFIFCYIGEIVAEECRKIGEISYM---IEWYRLMGN-------- 358
AmelOr25        -------------TFNIFIFCYIGDGVAEQCQKVGEMAYM---IEWYRLTGK-------- 353
AmelOr29        -------------TFNIFIFCYIGELVAEQTEKVGEVAYM---IEWYRIRGK-------- 354
AmelOr30        -------------TFNIFIFCYIGDLVAEQCQKVGEMTYM---IEWYRLTGK-------- 354
AmelOr21        -------------IFNIFVFCYIGELLADQSRKIGEVTYM---IEWYRLSGK-------- 354
AmelOr23        -------------IFNIFIFCYIGELLANQSRNIGEVTYM---IEWYQLFGK-------- 354
AmelOr35        -------------IFNIFILCYIGEILSEECKSIGLSAYM---IDWHRLPGK-------- 363
AmelOr27        -------------CFNIFIICYIGEILTEQCMKIGEVVYM---TDWYYLPDK-------- 356
AmelOr28        -------------CFNIFIICYIGEILAEESMKVGEVVYM---TDWYYLPDK-------- 356
AmelOr33        -------------IFNIFIICYIGDILTEQCKMIGEAVYM---TNWYYLPGK-------- 358
AmelOr34        -------------IFNIFIICYIGDVLTEQCKMIGEAVYM---TNWYYLPGK-------- 358
AmelOr32        -------------IFNIFIICYIGDILTEQCKMIGEAVYM---TNWYYLPGK-------- 358
AmelOr31        -------------SFNIFIICYIGDILMEQCRKVGEVLYM---TNWYYLPYK-------- 357
AmelOr36        -------------TLNTFLICYIGEVLTERCKEIGNMVYM---TNWYRLPKK-------- 354
AmelOr37        -------------TLNTFLICYIGEVLTERCKKIGNMVYM---TNWYRLPKK-------- 355
AmelOr38        -------------TLNTFLICYIGEVLTERCKEIGDMVYM---TNWYRLPKK-------- 356
AmelOr39        -------------IFSTFLICYIGEILTERCKEIGNMVYM---TNWYQLHDK-------- 356
AmelOr47        -------------TLNTFLICYIGEVLTERCKEIRNMVYM---TNWYRLPDK-------- 355
AmelOr48        -------------ILSTFLICYIGEVLTERCKEIGNMVYM---TNWYHLPDK-------- 355
AmelOr40        -------------ALSTFLICYIGEVLTEKCKEIGNMVYM---TNWYRLSDK-------- 355
AmelOr43        -------------TLNTFLICYIGEVLSEKCKKISNMIYM---TNWYRLSEK-------- 356
```

```
AmelOr45        -------------SLGTFLICYVGEILTEKCKEIGNMVYM---TNWYRLPKK-------- 355
AmelOr41        -------------ILSLFLICYISEVLNEKCKEIGNIVYM---TNWYRLSDK-------- 355
AmelOr42        -------------ILSLFLICYISEVLNEKCKEIGNIVYM---TNWYRLSDK-------- 355
AmelOr44        -------------ILNTFLICYIGEILSEKCKKISDMIYM---TNWYQLSDK-------- 355
AmelOr46        -------------TCNIFLICYIGEILTEQCKKIGEIIYM---TNWYELSNK-------- 354
AmelOr49        -------------TYNIFLVCYIGEIITEECKKIGDIVYM---TNWYELSDK-------- 355
AmelOr50        -------------SFNILLVCCIGEILREQCKKVGDMVYM---TNWYQLPDK-------- 357
AmelOr26        -------------VFNIFIFCYIAEIVTEQGKKVGEKFYM---TEWYQLPHK-------- 360
AmelOr53        -------------TFNIFIFCYIGEILTEQCSQIGTVSYE---IDWYKLSPK-------- 361
AmelOr54        -------------TFNIFIFCYIGEILTEQCSQIGTVSYE---IDWYKLSPK-------- 359
AmelOr52        -------------TFNIFIFCYIGEILSEQCSQIGTISYE---INWYKLPAK-------- 356
AmelOr51        -------------TFNILIFCYVGELLLGQGSEIATALYE---IEWYNLPGR-------- 358
AmelOr55        -------------TFNMFLLCYIGDLLIHKSGNIGVAVFM---IDWYHLPAK-------- 357
AmelOr57        -------------TFNMFLLCYIGDLLIEKSGNVGVAVFM---IDWYHLPTK-------- 357
AmelOr56        -------------TFNMFLLCYIGDLLIQKSSNIGVAVFM---IDWFHLPTK-------- 357
AmelOr58        -------------VFNIFLLCYIGNLLIEKSSNIGIVCCM---IDWYQLPIK-------- 359
AmelOr59        -------------IFNIFILCYIGELLMEKSSNIGLSCFM---IDWYYLPSK-------- 360
AmelOr60        -------------TFNIFILCYIGELLMEKSSSIGLSCFM---IDWYHLPVK-------- 360
AmelOr61        -------------TFNIFILCYIGERLMEKSSSIGLSCFM---IDWFQLPTK-------- 360
AmelOr63        -------------LIQLFAYSYVGEYLKTQTEGLGNSIYF---CTWYDMPKN-------- 346
AmelOr64        -------------LIQLFAYSYVGEYLKTQTEDLSNSVYF---CTWYDMPKN-------- 342
AmelOr66        -------------LIQLFAYSYVGEYLKTQTESLGNSVYF---CTWYEMPKN-------- 342
AmelOr65        -------------LIQLFAYSYVGEYLKTQTESVGNSVYF---CTWYDMPKN-------- 342
AmelOr67        -------------LIQLFAYSYVGEYLKTQTEGIGNSVYF---CTWYDMPKN-------- 342
AmelOr114       -----------YVMLQLYLYCYVGERLSVESMEIVNAAYN---TEWYTLPTN-------- 321
AmelOr115       -----------YTMLHLFIYCYVGETLLGQSTGIGLSTYH---CNWYDLPSR-------- 344
AmelOr118       ------------VNCEAFILCYTGEYITSKSENINKAVYN---FLWYNLKPR-------- 322
AmelOr164       ------------LNFEALILCYTGEYLSSKSENISWIAYN---SNWYELSIY-------- 334
AmelOr163       ------------ANCEAFILCYTGEYLMFKNESIVHAAYD---TLWYNLNPR-------- 336
AmelOr165       ------------ICLEAFIYCFAGEYLRIK------------------------------ 307
AmelOr166       ------------VCSEAFIYCFAGEYLSIKSKLIADTAYE---FLWYNMNPN-------- 325
AmelOr167       ------------ITLEAFIFSIAGEYLSNKSLSVSISAYE---SPWYLLSPK-------- 336
DmelOr35a       ------------------SLGQIGSDLAFTTDSLSTMYYL---THWEQILQYSTN-PS-- 350
DmelOr74a       ------------------ALGMLGSILLKTTDELGMMYYT---ADWEQVIHQSDN-VG-- 351
AgamOr3         ------------VTVETYGYGYFGTDLTTESYGVALAIYD---SEWYKFSIS-------- 359
AgamOr5         ------------LTVETYGFCYFGSDLTSESLSVARAAYG---SLWYRRSVS-------- 340
AgamOr13        ------------DTTETIAYCYFGEQLSNESARVAHTVYE---SGWETQTPD-------- 339
AgamOr15        ------------DTTETFAYCYLGEKLSYESARVAHTIYE---SGWETQTTD-------- 338
AgamOr17        ------------DTTETFAYCYLGELLSYESARIAHIVYK---NGWERQHAY-------- 338
AgamOr16        ------------DTTETFGYCYLGNQLSDESARVASVVYD---CRWEGMPPA-------- 336
AgamOr55        ------------VSTETFGYCYLGTQLSMESARIAYAVYN---GKWEQQPRE-------- 339
AgamOr18        ------------VSIETFGYCYLGTQLSQESINVGQALYA---SGWYEYDVQ-------- 346
AgamOr14        ------------ITIETFSACYFGTRLSTQAVELSKSVYG---CGWPAMDRD-------- 360
AgamOr51        ------------ITIETFSPCYFGTRLSTQAVELSESVYA---CGWPAMDRD-------- 348
AgamOr12        ------------LTPEMFGYCFFCSDLTTTGTTISRQMYE---FQWERHRPA-------- 349
AgamOr19        ------------LTPEMFGYCFFCSDLTTTGTTISRQMYE---FQWERHRPA-------- 349
AgamOr20        ------------MTLEMFGYCFFCTELATTGTLIACQSYE---FRWEEHDPK-------- 349
AgamOr21        ------------MTLEMFGYCFFCTELDTTGKIVSRQMYE---FRWEQHRPT-------- 346
AgamOr50        ------------MTSEMFGYCLFCTELTNTATTISQQIYV---FQWEKHSPA-------- 348
AgamOr29        ------------VTVETYTYCTLGTQLTDKGEEVLMALQQ---LAWYDQSIP-------- 337
AgamOr53        ------------VSIETYAYCMLGSQLTTQGEDLLMALQQ---LSWYDQPVP-------- 327
AgamOr30        ------------LTCETYSYCQLGTQFTSNAEEVLDELQQ---LARYDQSIP-------- 338
AgamOr46        ------------LSTETFIFCLLGNELTLKGLEISTAMYS---TNWYDQPVK-------- 338
AgamOr47        ------------LSTETFIFCLLGNELTLKGLEISTAMYF---TNWYDQPVK-------- 338
AgamOr48        ------------IFIEMMCFSYLGNQLTEENANISHSAFN---CRWYDEPIV-------- 348
AgamOr49        ------------LTVENLCFSYFGNRLSTENTSVAIAVYS---TDWYNYPPC-------- 355
AgamOr61        ------------LVFQSFILCRGIEKLNDLNEAIGQALYSG--FDWPDMLQY-DQ-RFRR 365
AgamOr62        ------------LILESLLLCRGVEDLNDLNRQIGTILYN---FDWPRLLRF-SI-HYRR 362
AgamOr63        ------------FLTESFLLCHGVENLRDLKPRIASTVYD---FDWMLQMRCPNP-RHRA 358
AgamOr60        ------------LVAESFLFCRILSQINDLHARIGTVMCE---LEWYDKLRF-ST-RFAS 359
AgamOr75        -----------IYFLETFGMCMLVEMLVQLNRKVSTSLYG---FSWPQYLRY-------- 358
AgamOr76        -----------IYFFETFGMCMLVEMLVQLNRKVSTSLYG---FSWPQYLRY-------- 358
AgamOr78        -----------LFLSKTFGMCMLVEMLVQLNRKVSTSLYG---FSWPQYLRY-------- 359
AgamOr77        -----------IYLLELFQYCWQVDEMELLHEGQAFAVYS---TPWTGAIMQ-------- 337
AgamOr79        -----------IYLLELFQYCWQVDEMELLHEGQAFAVYS---TPWTGAIMQ-------- 335
AmelOr99        --------------FHLFLLSLPGQILLDHCADFANAIYD---STWYGTSLE-------- 355
AmelOr101       --------------FHLFLLSLPGQILLDHCADFANAIYD---TTWYGTSLE-------- 355
AmelOr102       --------------FHLFVLSLPGQVLLDHCSEFSNNIYS---CIWYRAPVR-------- 354
AmelOr103       --------------FHLFVLSLPGQVLLDHCSEFSNNIYN---CIWYRVPVR-------- 354
AmelOr104       --------------FHLFVISLPGQVLLDHCTELANNIYS---STWYRIPVK-------- 355
```

```
AmelOr98        --------------FHLFVLSLPGQILLDHCAELSNTIYC---SMWYKLPVK-------- 352
AmelOr100       --------------FHLFILSIPGQILLNHYSNLKNNIFM---SSWYNMPIE-------- 275
AmelOr97        --------------FHLFIITLTGQVIADQSSKLSNNMYC---TTWYRMPPN-------- 299
AmelOr105       --------------FHLYAISLPGETLLNQSSKLADKIYD---SEWYKIPMK-------- 355
AmelOr109       --------------IHLFFLTIMGQFLINANEETFKTIYE---ADWYNGSSK-------- 343
AmelOr110       --------------THLFFLTIMGQFLINANEEIFRTIYE---ARWYNGSSK-------- 342
AmelOr111       --------------THLFFLTIMGQFLINANEEIFKTIYE---ARWYNGSSR-------- 343
AmelOr108       --------------THLFFLTIMGQFLINANDEIFNTIYE---AHWYNGSSR-------- 343
AmelOr107       --------------THLLFLMVMGQFLIDSNEEVFKTIYD---ARWYYGSSK-------- 343
AmelOr112       --------------FHTFILTVQGQFVINELQDVYESIYE---SPWYTFSPR-------- 340
AmelOr113       --------------MHLCYVMIQGQFVIDSCNEIYYSIYE---ASWYNINPK-------- 341
AmelOr106       --------------VHIFFLTAQGQFVIDSHDNVYRDMYE---PYWYNVQYK-------- 339
AmelOr122       --------------MIAFYNNHCGQLIIDSNLGIFNELFA---STWYRIPLK-------- 314
AmelOr125       --------------MIAFYNNHCGQLIIDSNLGIFNELFA---STWYRIPLK-------- 313
AmelOr123       --------------MMALYNNHYGQLIINSNHGIFNELCA---STWYRIPLK-------- 311
AmelOr126       --------------MMALYNNHYGQLIINSNHGIFNELCA---STWYRIPLK-------- 311
AmelOr127       --------------MTALYNNHYGQLIINSNHDIFNELCA---STWYRIPLK-------- 311
AmelOr128       --------------MIAFYSNYCGQLIIDSNLGIFNELYA---STWYRIPLK-------- 311
AmelOr129       --------------MIAFYNNHCGQLIIDSNFGIFKELYA---STWYRIPLK-------- 311
AmelOr133       --------------VIMFLCNHTAQILINNNEEFFHELYI---SVWYSVPLK-------- 216
AmelOr134       --------------VFIFLCNHTAQILINNSEEFFYELYI---SVWYFVPLK-------- 312
AmelOr131       --------------MIMFLCNYNGQILINSNEEFFHELYI---PVWYFVPLK-------- 314
AmelOr132       --------------MIMFLCNYNGQILIDNSEELFDELYF---SIWYFVPLK-------- 314
AmelOr135       --------------IIMFLCNHSGQILIDNSQKLFNELYI---SIWYFVPLK-------- 312
AmelOr136       --------------VITFLNNNCNQILIDNSQELFIELYI---SMWYFVPLK-------- 314
AmelOr138       --------------IITFLCNYNNQILIDNSQELLDELYI---SAWYFVPLK-------- 220
AmelOr130       --------------VIIFLNNYNGQIVMNSSQELFDELYN---SMWYFMPLK-------- 314
AmelOr137       ------------------------------------------------------------
AmelOr139       --------------MIMFICNYSSQILIDNSQELLYDLYI---SMWYFVPLK-------- 236
BmorOr20        -----------------IQLSVIVESVGTVTGRLKDAVYE---VPWEYMDTSN------- 390
BmorOr3         -----------------IQLSVIVESVGTVTGRLKDAVYE---VPWEYMDTSN------- 390
BmorOr18        -----------------IQLSVIVESVGTVTGRLKDAVYE---VPWEYMDTSN------- 390
HvirOr6         -----------------IQLSVIFELVGTESEKLKDAVYG---VPWDCMDTKN------- 380
BmorOr23        -----------------IQISVIFELLGYMSDKLQDAVYC---VPWEYMDTSH------- 367
BmorOr16        -----------------IQISVIFELLGYMSDKLQDAVYC---VPWEYMDTSH------- 367
BmorOr4         -----------------IQISVIFELLGYMSDKLQDAVYC---VPWEYMDTCH------- 366
BmorOr5         -----------------IQISVIFELLGYMNDKLQEAVYC---VPWEYMDTSH------- 367
BmorOr22        -----------------IQVSMIFEILGFLSDKLPNAVYS---IPWEAMNVTN------- 374
BmorOr17        -----------------IQVSMIFEILGFLSDKLPNAVYS---IPWEAMNVTN------- 374
BmorOr1         -----------------IQISIIFELLGSVADRIPDAVYQ---LPWECMDVKN------- 381
BmorOr1a        -----------------IQISIIFELLGSVADRIPDAVYQ---LPWECMDVKN------- 381
BmorOr1b        -----------------IQISIIFELLGSVADRIPDAVYQ---LPWECMDVKN------- 381
BmorOr19        -----------------IFLSIVLEEIRRQNYDLCEYVYA---LPWEGMSLEN------- 359
BmorOr15        -----------------IFLSIVLEEIRRQNYDLCEYVYA---LPWEGMSLEN------- 359
TcasOr5         -----------------------NAVLNAEYETVLDFVWT-------------------- 263
TcasOr7         ---------------------VMNEIICERFRSKAAKNYR-------------------- 278
HvirOr4         -----------CVTHMLRMIVVVEPGHYTIAEGKRTEGLVC--RLMTSAPSTG------- 342
BmorOr13        -----------CLYHTFGLVMIIEPCHRTHEEMETTRELVS--RVMCSADPRD------- 389
DmelOr10a       ---------------QLLVYCYGGTLVAESSTGLCRAMFS---CPWQLFKPKQ------- 359
DmelOr33c       ---------------QLFPSCYFASEVAEELERLPYAIFS---SRWYDQSRDHR------ 333
DmelOr7a        ----------------TAPCCYQATSLMLDNERLALAIFQ---CQWLGQSAR-------- 355
DmelOr22c       ----------------LFLYCFGGNHVSESSAAVADVLYD---MEWYKCDART------- 355
AgamOr1         ---------------QVFIFCYVGNEISYTTDKFTEFVGF---SNYFKFDKRT------- 365
AgamOr4         ----------------TFLYCLLGTRLATQQQLLEHALYA---TRWYNYPIAF------- 343
DmelOr47b       --------------IQLSLWCVSGTLVYTQSVEVAQAAFDI--NDWHTKSPG-------- 363
AmelOr73        ---------------QVYLYCWFGNEVTLKSNKVSDAIYE---MDWTILPSN-------- 314
AgamOr68        --------------------FVLGAIIERKVDDLHISLMH---FPWYLMDDRR------- 341
DmelOr59a       ---------------QIFPSCFFGTDNEYWFGRLHYAAFS---CNWHTQNRS-------- 329
DmelOr23a       ---------------QTYPLCYYGTMVQESFAELHYAVFC---SNWVDQSASY------- 327
AgamOr31        ----------------VTYYCYYGSQLYYESTQVHDAVFK---SKWYDASVAT------- 324
DmelOr2a        --------------LEVFVICYFGDRMRTQSEALCDAFYD---CNWIEQLPKF------- 349
AmelOr62        -------------LIQLYLYAYAGDALESRTEEIAQAAFH---SFWYQSRGRT------- 349
AmelOr121       ---------------QTFLYFMAGQMLVTQSEEVHNAAYE---CEWVSLKYTK------- 345
AmelOr140       ---------------HIFFLCLPGQRLLNHSEELHVSACE---VTWYIFPKKY------- 311
TcasOr12        ---------------EIYLWCFYGNEVMLKSDQLTQAAYM---SEWLDGTEEF------- 341
AgamOr8         ----------------VFLLCYYGNKLIVASSQIPYSAFE---GNWIGASVSY------- 343
BmorOr9         -----------------LIMSVFGENLISESSRIAEAAFL---CKWYEMDQKS------- 329
AgamOr40        ---------------ELYLYCFLGQTLKNQGLKVGDALWK---SPWHLCGASY------- 406
TcasOr63        ---------------QVATYCYYGHEVMTTSDAIGMSLYL---SNWYASHVK-------- 331
TcasOr4         -------------WLPYNTKKYYYPTVIFQVMSVSVSAYNN--SSIDVLTCMLIT----- 333
```

```
TcasOr13        ------------HVLRLLLIVEPCHGCILVAKTTTQLVCK---LLCLDLDKEVK------ 357
AgamOr24        -------------SVQIFYICFLGTSLELKGGVTNALTLKIGAIHWDKLSGRD------- 329
AgamOr54        --------------VQLFFSCALGETFNIKSDELTVAIYN---VPWYNMEVRD------- 316
AgamOr23        --------------------CALGTFLSSKNEKLVEEIYN---VNWYGLTTKH------- 333
AgamOr64        ----------------TLLFCKSIDSVNDLHTEVGYIMYS---EYWPATLQYADQGLSTE 365
DmelOr82a       ---------------QLFIYCYGGEIIKAESLQVDTAVRL---SNWHLASPKT------- 337
AmelOr116       ---------------QIFIYCWHGNELYLHSMKICLSAYK---NNWWNNNKN-------- 349
AmelOr160       -------------ILLAFCYCFLGECLINESSEVQMACYF---TNWYDLPEQY------- 342
TcasOr10        ----------------SFILSLDISNVIFYVDWLDDEVYKS------------------- 297
TcasOr26        ---------------------------------VGTTVYN---SEWCDTDVDF------- 290
AmelOr117       ---------------QLLLWCWPGEILIQESQEIGHVIYLN--IPWYNLPPIY------- 351
AmelOr120       ---------------QLLLFCFPGDILIRQSFNISIAAYS---MQWYQLPTFI------- 352
BmorOr7         ---------------ELFLYCYYGNEVSTESDRLVTSIYS---MEWVGARLG-------- 341
AgamOr6         ----------------ESRVIEAFAFKTHQSLGVIDAAYG---CEWYREGSVA------- 353
DmelOr45a       ----------------LSSYCYGGEYIKQQSLAIAQAVYG--QINWPEMTPKK------- 330
DmelOr67d       ----------------LYTFCGLGTLVENSNEDFLSVIYTN--CLWYELPVKE------- 342
DmelOr83c       -------------AYSMSIYCILGTILEFAYDQVYESICN---VTWYELSGEQ------- 349
AmelOr162       ---------------DLLFVSWTGQQLSNSTERIFEYTTN---GKWYQSSISC------- 320
HvirOr3         ----------------AYVYCYLGNELIIQSQGIALAAYES---TWTSWPVDL------- 220
TcasOr20        ----------------LFVVCYYGQKILDESLAIADAVYT---FLWYKKSVIV------- 337
AgamOr11        ----------------LFLYYWHANEIHACADRLSMSAYK---SDWYRYDRGT------- 375
DmelOr56a       ---------------ILWIYHWHATLIVECHDELSLAYFS---CGWYNFEMP-------- 369
AgamOr28        ---------------QLFLFCWYAQQITEEARLVSDHIYN---IPWYLADPKL------- 370
AgamOr58        ----------------MLNFGGLCDLLKIQVHAIKFRLYS---SQWTDYLRPVSG-PLYP 343
AgamOr59        -----------------FIFCQLVEKLQDMNRSIGDRLYG---TEWMLKLQYSRDFQR-- 362
DmelOr1a        ----------------LCIYLFGAEQVRLEAERFSRLLYEV--IPWQNLPPKH------- 339
DmelOr13a       ---------------QLYVLCENGDALIKQSTLTAQILYECQWEGSDRIEIQSFTP---- 357
AmelOr161       ---------------ELFMFSFSGNGLIDESEGIGESAYN---SGWIGSRFCR------- 337
AmelOr141       ------------------LCNWPADLLLSKSIDISRATYS---MPWYGYSYN-------- 380
AgamOr52        ----------------WYYCCKLVEDLQATNKRIGWTLYNDKWSDWLQYGREQP-----A 330
AgamOr36        ----------------CYWCCQLVDRLNEVNTQIGILLYS---LDWPVELQYTKATASR- 352
AgamOr25        --------------------CFSGNLLLIESDSLSSCVYS---IDWHTMPVPE------- 313
AgamOr45        ---------------QLFVICMLGNVLYIVSDRLKDSVYG---IRWYRCTVSQ------- 339
TcasOr3         -----------------LMYCWFGNRITEASLKISYALYES---DWFPTSLS-------- 360
DmelOr88a       ----------------TFEICLRGTQLEKASEGLESSLRS---QEWYLGSRRY------- 354
AgamOr22        ---------------KIWLFCYCGELVVSKAGEVNTGVYAN---QWYRLWNRR------- 338
DmelOr49a       ----------------FYVVSSHGQMLIDLSTNLAKAAFES---KWYEGSLRY------- 346
DmelOr85f       ----------------VYLVCYYGQQVLDLSGEVAHAVYN---HDFHDASIAY------- 343
AmelOr119       ---------------QLFYICWLGNELGYASSTLDKNQWFS---GWCNERLTG------- 364
DmelOr63a       --------------YQIVVYCYNGQRFATASEEIANAFYQ---VRWYGESRE-------- 369
DmelOr67b       --------------LEITLYNVSAQKVESQSELLFHDWYN---CSWYNESRE-------- 370
BmorOr21        --------------------CRYAANLKCMAAETPTLIYC---CGWESVSDLR------- 343
BmorOr11        ---------------HFVMPCYYSNLLMERSENFRFAIYS---CGWENHHDKN------- 348
BmorOr12        ---------------LMAFFMWTAGDINVQASQLPDAIYG---SGWYNCRGKSS------ 353
AgamOr33        ---------------QVFVWSFFGNRVTNMSTGISDATIS---CNWIVLADG-------- 323
HvirOr1         EAAAYFLFSLVYLLSRSVAVSLIASQVNSASSVPAPVLYDVPSPVYCVEVQRFLDQVNGD 399
HvirOr5         ---------------RVISVVLAASSVNTHSKLALNHLYN-----YETHCYN-------- 281
TcasOr1         --------------RICTLHHHLSKLVTRFNEIFGLGLLLMFGVSFLLITQT-------- 305
TcasOr2         -------------------------TYGIDTVAVCDACYS-------------------- 305


TcasOr16        -----KTFVQIVCQQCQKAMSISGAK----FFTISLDLFASVLGAVVTYFMVLVQLK--- 475
AmelOr2         -----KTFVQIVCQQCQKAMSISGAK----FFTVSLDLFASVLGAMVTYFMVLVQLK--- 478
AgamOr7         -----KTFVQIVCQQCQKAMTISGAK----FFTVSLDLFASVLGAVVTYFMVLVQLK--- 478
AaegOr7         -----KTFVQIVCQQCQKAMTISGAK----FFTVSLDLFASVLGAVVTYFMVLVQLK--- 462
DmelOr83b       -----KTFVQIVCQQCQKAMSISGAK----FFTVSLDLFASVLGAVVTYFMVLVQLK--- 486
BmorOr2         -----KTFVQIVCQQCQKAMTISGAK----FFNVSLDLFASVLGAVVTYFMVLIQLK--- 472
BmorOr2a        -----KTFVQIVCQQCQKAMTISGAK----FFNVSLDLFASVLGAVVTYFMVLIQLK--- 472
HvirOr2         -----KTFVQIVCQQCQKAMSISGAK----FFTVSLDLFASVLGAVVTYFMVLVQLK--- 472
AgamOr56        -----QKDMCFLMMRANKPVKLTAAK----LFVVTRDSFTQVIKQAYTIFTLMSQFLDNP 398
AgamOr57        -----QKDMCFLMMRAKRPVKLTAAK----LFVVTRDSFTQVIKQAYTIFALMSQVLDDT 398
AgamOr26        -----QKDLSFVLMRAKKPVRLTAAK----LFVVTRDSFTQVMKQAYTIFALMSQFLDDI 398
AgamOr27        -----QKDMCFLMMRANKPVKLTAAK----LFVVTRDSFTQVIKQAYTIFTLMSQFLDNP 398
HvirOr9         -----RKGFFIVIIRAQKPCELTANG----FFKINLSAFTSILSTSWSYFALLKTMYHPE 401
HvirOr7         -----GKQILFVQTRAQEPCKLTAAG----FADVNLNAFMRVLSSAWSYFALLQTVYGGK 385
DmelOr85b       -----KRALVIIIARSQKVTFLKATI----FLDITRSTMTDLLQISYKFFALLRTMYTQ- 390
DmelOr85c       -----RRALVFFIARPQRTTYLKATI----FMNITRATMTDLLQVSYKFFALLRTMYIK- 389
DmelOr85d       -----RKMLILIIKRAQQPSRLKATM----FLNISLVTVSDLLQLSYKFFALLRTMYVN- 412
DmelOr67a       -----KKILIFISMRSQKPVCLKATV----VLDLSMPTMSIFLGMSYKFFCAVRTMYQ-- 407
DmelOr67c       -----CTMLKLLIMRSQKPASIRPPT----FPPISLVTYMKVISMSYQFFALLRTTYSNN 404
```

```
DmelOr92a       -----KRILQFIIARSQKPASIRPPT----FPPISFNTFMKVISMSYQFFALLRTTYYG- 408
DmelOr69a       -----RRMLLILMMRATKPYMWKTYK----LAPVSITTYMATLKFSYQMFTCVRSLK--- 393
DmelOr69b       -----RRMLLILMMRATKPYMWKTYK----LAPVSITTYMATLKFSYQMFTCVRSLK--- 393
TcasOr6         -----KKQIARLLNGSQ--RFWDLGQ----FGARYGNSFGKTHKYLKSFFLLYKVMLTFT 373
TcasOr11        ------KIIAMIRGQRA--AKLTLGG----FGDLDLESFNLVVKNAFSFFTFVNAMNQK- 332
AgamOr41        -----KQIFRLLLLSSQQPQTLTCAG----MTCISLNLFVNMSQKFYSIFMMLRNM---- 382
AgamOr42        -----QGMFKYVLKSAQQPKQLTCGR----FAVINMNLFLAIHKKIYSFFMMLQNM---- 387
AgamOr43        -----KKDFLIMFHKCQHAKEMTVAS----MAPLNIVLFIAIMQKIYALAMMMMRFSE-- 386
AgamOr44        -----KKDFLIMFHKSQHAMEMTVAS----MAPLNIVLFIAIMQKIYAYAMMLMNFFE-- 386
AgamOr66        -----QKQFKFMLARSQLSAEIMIRS----VGPMNMETFTDIMQKMYSAFAMMYSFLVDL 392
AgamOr67        -----QKQFKFMLARSQLSAEIMIRS----VGPMNMETFTDIMQKMYSAFAMMYSFLVDL 392
AgamOr72        -----QKEFRFLMSRQQCPMILTAYG----FHPMNFEAYMSVLKVLYQFFVMIMQYIDRN 388
AgamOr73        -----QKEFRFLLSRQQCPMILTAYG----FHPMNFEAYMSVLKGLYQFFVMILQYIGRN 388
AgamOr71        -----QKEFRFLMSRQQYPMMLTAYG----FHPMNFEAYMSVLKRLYQFFVMLMQYVG-- 386
AgamOr74        -----QKDFRFLMSRQQNPMMLTAYG----FHPMNFEVYMSVLKRLYQFFVMIMQYVG-- 386
AgamOr70        -----QKEFRFLMSRQQCPMMLTAYG----FHPMNFEAYMSVLKRLYQFFVMVMQYVG-- 386
AgamOr69        -----QKEFAFLMCRQQRPMMLTAYG----FLTMNFESYMSVLKGLYQFFVMIMQYVE-- 390
TcasOr9         -----QRTFHIVQMRASKIVNVKAGN----YFTMSASSYITFMKSLGIVYSALEGVN--- 294
TcasOr18        -----QKMLILVQMRVKRMVSTKAGN----FFTMIAPSFIAFQRAVFSYITLLKEVTDLG 408
DmelOr83a       -----RSDYMFFMLNSRRQFQLTAGK----ISNLNVDRFRGTITTAFSFLTLLQKMDARE 453
DmelOr85e       -----RQDILIFLVNSRRAVHVTAGK----FYVMDVNRLRSVITQAFSFLTLLQKLAAKK 462
TcasOr24        -----MSYSILTILQ--------------------------------------------- 343
TcasOr25        -----RKDLILIIILRAQKALFVSTGP----FNVLSFALFVSLKTSFIWPPKNEEMTPSRW 384
TcasOr19        -----RKNLILMIQRSQKPLFLATGA----FNIMSFALFVTV------------------ 331
TcasOr22        -----QKMLLLLLVRSQKAFNLHATP----VGIMSFALFVALLKTSYSYFTLLHQST--- 382
TcasOr23        -----KKVLLLLLVRSQKAFNLQATP----IGIMSFDL---------------------- 360
AmelOr168       -----QRVILMISLANELKCKISNFQ----NIDLTLPSFMSILNQAYSICLLFLKTKQD- 390
AmelOr170       -----QRVILMISLSNELKCKISNFQ----NIDLTLPTFMSILNQAYSVCLLFLKARQD- 397
AmelOr169       -----QHIILMITLANKLKCKLSSFR----NIDLTLPSFMSILNQAYSVCLLFLKARQS- 403
DmelOr65b       ----VYRDLCVIIRRGQDPLIMRASP----FPSFNLINYSAILNQCYGILTFLLKTLD-- 385
DmelOr65c       ----VYRDLCLIIRRGQEPLIMRASP----FPSFNFINYSAILNQCYGILTFLLKTLD-- 385
DmelOr65a       ----IHRQFCFFIQRAQKPLIMKASP----FPPFNLENYMFILKQCYSILTILANTLE-- 417
TcasOr17        ------------------------------------------------------------
TcasOr21        ------------------------------------------------------------
TcasOr56        ----PKKTLLLLMMRAQRPCYFTAGK----FSLLSLQTFMTIVRGAGSYFMFLRQMNI-- 433
TcasOr15        ----------------------------------------TVVRGAGSYFMFLKQMNT-- 340
AgamOr9         ----TRKLLIAFQQITARGIKCSAKYI-F-TIELSMQTFVTIIKTSYSYLAVLRSMTD-- 418
AgamOr65        ----CRKLLIAFQQITAVGIKCSAKYV-F-TIQLSMETFVTILKTSYSYLAVLRSMTD-- 421
BmorOr6         ----FKRNLCIAMECMAKSLTFRAG---S-YIPLSRAMFVSILRSSYSYFAFLNQANEQY 391
BmorOr10        ----FRKVLIITMERMKRPIIFKAG---H-YIPLSRPTFVAILRCSYSYFAVLNRVRNE- 387
AgamOr34        ----NRKSVQMFLVRTNKPFAVAAFG----YFNFNLPAFTTILNMAYSVYCVLQRKAKNV 380
AgamOr37        ----ERKTLRMLLQRMNKPLTLKAFY----FFNYNLQAFTTTLNMAYSLYALLQRNALKK 392
DmelOr94a       ----IRKLLNAYMEHLKKPVTIRAGN----FFAVGLPIFVKTINNAYSFLALLLNVSN-- 387
DmelOr94b       ----TRKLLNCYMEFLKRPVKVRAGV----FFEIGLPIFVKTINNAYSFFALLLKISK-- 383
DmelOr71a       ----MRRLVLMFMVYLNRPVTLKAGG----FFHIGLPLFTKTMNQAYSLLALLLNMNQ-- 378
DmelOr46a       ----SRRIALLFMQRLHSTLRIRTLN---PSLGFDLMLFSSIVNCSYSYFALLKRVNS-- 381
DmelOr46b       ----NRRIVLLMMQRFNSPMLLSTFN---PTFAFSLEAFGSIVNCSYSYFALLKRVNS-- 384
HvirOr8         ----FKSSMRIFIERAHKPLSITGGK----MFCLSLVTFTSIMNTAYSFFTLLQNVKSRK 397
BmorOr8         ----FKRSIIIFVERAKIPITITGLK----MFPLSLATFTSIMKTAYSFFTLIRNMQALQ 394
AmelOr68        ----SKKDLINIMRRAMNPIELTCAY----IFTIDLRTFVTILKMSYSTYNFLQRTKVN- 375
AmelOr69        ----TKRDLLMIMMRASYPIEMTSVH----VITMNLDSFVILLKTSYSAYNLLQSNRE-- 369
AmelOr70        ----TKKIFLIIMTRATQPFEFTSGY----LVTLNLEFFVALIKASYSVFNLLQRTK--- 370
AmelOr71        ----VQRAILLVMRRSMKPIEFTSIY----IVSVNLDSFMTLLKSSYSAFSVLQQSRES- 371
AmelOr72        ----AKKILLIIMARSLTPVEVVSAH----IIPLNLESFKRLIKATYSAYNMLQQTK--- 370
DmelOr19a       ----FRRLLLLMLARCQIPMILVSGV----IVPISMKTFTVMIKGAYTMLTLLNEIRKTS 385
DmelOr19b       ----FRRLLLLMLARCQIPMILVSGV----IVPISMKTFTVMIKGAYTMLTLLNEIRKTS 385
DmelOr33a       ----YNRSLIILMQLTLVPVNIKAGG----IVGIDMSAFFATVRMAYSFYTLALSFRV-- 378
DmelOr33b       ----YSRILLIFMQLTLAEVQIKAGG----MIGIGMNAFFATVRLAYSFFTLAMSLR--- 379
DmelOr22a       ----YKSTLVYFLHNLQQPITLTAGG----VFPISMQTNLAMVKLAFSVVTVIKQFNLAE 394
DmelOr22b       ----YKSTLVYFLHNLQQPIILTAGG----VFPISMQTNLNMVKLAFTVVTIVKQFNLAE 394
DmelOr42b       ----YKTTLLYFLQNVQQPIVFIAGG----IFQISMSSNISVAKFAFSVITITKQMNIAD 394
DmelOr59b       ----YKATLVLFMHHVQQPIIFIAGG----IFPISMNSNITVAKFAFSIITIVRQMNLAE 395
DmelOr59c       ----YKSAVLYFLHRAQQPIQFTAGS----IFPISVQSNIAVAKFAFTIITIVNQMNLGE 398
DmelOr98a       ----YKSSLRYFLKNAQKSIAFTAGS----IFPISTGSNIKVAKLAFSVVTFVNQLNIAD 392
DmelOr42a       ----YQKTLMYFLQKLQQPITFMAMN----VFPISVGTNISVTKFSFSVFTLVKQMNISE 397
DmelOr85a       ----YRTTMLYFIHNVQQSILFTAGG----IFPICLNTNIKMAKFAFSVVTIVNEMDLAE 392
DmelOr43b       ----YQRTVIQFLQKLQQPMTFTAMN----IFNINLATNINVAKFAFTVYAIASGMNLDQ 397
AgamOr2         ----IRKRLILIIARAQRPMVIKVGN---VYP-MTLEMFQKLLNVSYSYFTLLRRVYN-- 378
AgamOr10        ----IKKKLLMMTIRAQRPLEITVGN---VYP-MTLEMFQSLLNASYSYFTLLRRVYN-- 375
DmelOr30a       ----TQKTLKFLIMRSQKPLAILVGG---TYP-MNLKMLQSLLNAIYSFFTLLRRVYG-- 377
```

```
DmelOr49b       ----LRKNILFMMMRAQRPAAILLGN---IRP-ITLELFQNLLNTTYTFFTVLKRVYG-- 375
DmelOr43a       ----FRKTLLIFLMQTQHPMEIRVGN---VYP-MTLAMFQSLLNASYSYFTMLRGVTGK- 376
AgamOr32        -----KKLVYICLMRAQKPVIMKSGF---IEA--SLPTLKKILSSSASYITMLMSLEADL 379
AgamOr35        -----QKRLRLCIMRAQKPIVTKGGF---IKA--TLPTLKKILNSTGSYITMLLSLETEQ 389
DmelOr47a       ASTSICRSLLISMMRAHRGFRITGYF---FEA--NMEAFSSIVRTAMSYITMLRSFS--- 385
DmelOr98b       --LQLVSSLKIAMMRSSLGCPIDGYF---FEA--NRETLITIVRTAISYVTLLRSLA--- 382
DmelOr9a        -----KRALLIAAMRAQRPCQMKGYF---FEA--SMATFSTIVRSAVSYIMMLRSFNA-- 392
AgamOr38        -----RHLVQMMMVRAKYGCNVDVPF---FRT--SMATFSVIVRSAMSYITLMKSFL--- 407
AgamOr39        -----RHLIQMMMIRAQYGSNVDVPF---FET--SMASFSAIVRTASSYITLMKSFL--- 408
DmelOr24a       -----QKMTLLMVARAQRVLTIKIPF---FSP--SLETLTSILRFTGSLIALAKSVI--- 387
DmelOr45b       -----RRRVFLIILRAQRPITVRVPF---FAP--SLPVFTSVIKFTGSIVALAKTIL--- 396
AmelOr74        TGRMLRKDIRIIMMKSMRPCYLSRSG---FFP-MSLETSTA------------------- 382
AmelOr86        AGRMLREDVKVMIMKSMRPCHLSAGG---FFP-VSLETSTALMSSTLSYFTLMRESSKDK 400
AmelOr75        AGILVRKNLIIVIMRSQKICCLTAGK---FFP-VSLETSTAVLSTAISYFTLLKQSSLEN 400
AmelOr84        VGRVVRKNLIIVVARSHRVCCLTAGK---FFP-VSLETSTAVLSTAMSYFTLLR------ 391
AmelOr87        IGRTLRRDLRMVIIRSRKPCCLTASR---FFP-VSLETCTTVLSTAMSYFTLMRQSFAN- 424
AmelOr76        YGKMIRRDLMFVITRSRTPCCLTACG---FFA-VSLETYTKVLSTAISIFTILKRYEKEF 403
AmelOr85        YGKMLRKDLILVIMRSKSPCYLTALG---FFP-VSLETYISILSTAISYFTLLRNRAEQT 408
AmelOr88        YGKSLRKDLTFVIMRSRSPCCLTACG---FFP-VSLETYTGILSVAVSWFTSLKKYEKKL 406
AmelOr91        SGRMMRKDLIIVIMRASIPCCLSGKG---YFI-VSLETYTSVLSTAASYFTLLRNNIESD 407
AmelOr92        SGRMMRKDLIVVIMRASIPCCLSGKG---YFI-VSLETYTSVLSTAASYFTLLRNNIESE 404
AmelOr95        SGRMMKKDLILVIMRSGTPCCLTGRG---FFV-VSLETYTNVLSTAASYFTLLKQHSEAH 403
AmelOr93        SRRMIRKDLILVIMRSNVPCYLTGRG---FFI-VSLEMYX-VLSTAAX-FTLLKQRTEAT 379
AmelOr77        ------------------------------------------------------------
AmelOr94        FGKMIRKDLQFIVVRSRRACCLTGCG---FFD-ISLETYTKIMSTAMSYFTILKQRIVEV 402
AmelOr96        FGKMLRQDLQIVVMRSRRACCLTACG---FFP-ISLETYTKIMSTTMSYFTLLKQRTVDT 402
AmelOr79        FGRMLRKDLIMVIKRSRRVCCLTANG---FFP-VSLETYTKILSTAVSYFTLLNNRIENA 402
AmelOr83        FGRMLRNDLIMVIKRSRRVCYLTANG---FFP-VSLETYTKILSTAVSYFTLLNNRVENA 407
AmelOr80        FGKILRKDLIIVIRRSRRVCCLTANG---FFP-VSLETYTKILSTALSYFTLLSNRIENS 405
AmelOr81        FGKIFRKDLMIVIRRSRRVCCLTANG---FFP-VSLETYTKILSTALSYFTLLSNRVENA 402
AmelOr82        FGRMLRKDLIMVITRSRRVCCLTANG---FFP-VSLESYTKILSTALSYFTLLSNRVETA 404
AmelOr78        FGRMLRKDLIMVIMRSRRVCCLTANR---FFP-ISLETYTKILSTAVSYFTLLSKHVDNS 404
AmelOr89        FGKMLRNDLIMVIKRSRRVCCLTANG---FFP-VSLETYTTILSTAVSYFTLLRNNVEKA 401
AmelOr90        FGRMLRNDLIMVIERSRRVCCLTANG---FFP-VSLETYTTILSTAVSYFTLLRNNMEND 402
AmelOr142       ----TQRKILFIILRSQKYESFGING---IVPALSLSYYGKYLYTSLSYFNALRIMVEDT 373
AmelOr146       ----LQKFVLFATIP-QTPVILKVRC---IIPAFSLNYYCSFITNVLSMFTALRVLMYKD 372
AmelOr148       ----MQKDLLIILMY-QRPVILSINV---LLPELTLRYYCSYVANAFSVFTALRAVVEDK 369
AmelOr158       ----MQKTLLIMMAY-QKPVTFSINV---LLPELTLRYCCSYVSNALSIFTALRAVVEVT 370
AmelOr147       ----MQKLVLITLIY-QKPVTISINV---VLPTTYF---------ALLLF---------- 351
AmelOr150       ----MQKDVLTVLMY-QQPIILSINC---ILPELTLHYYCS------------------- 353
AmelOr151       ----MQKDVLNILVY-QQPIIFSVNC---ILPELSLRYYCSYLSNAFSIFTAIRVMIEDD 372
AmelOr152       ----MQKNLLNILMY-QQPIILSINC---ILPELSLRYYCSYLSNAFSIFTAIRVIIENN 372
AmelOr154       ----MQKNFLNVLVY-QKPVIFSISC---IVPELSLRYYCSYLSNVFSIFTTLRVLLEDT 372
AmelOr155       ----MRKYLLNVLIY-QKPITFSISC---IVPELTLRYYCS------------------- 353
AmelOr157       ----LQKALLTVLAF-QKPIAVSINV---LLPELTIRYYCSYVSNALSIFAALRTVVE-- 367
AmelOr156       ----MQQNILIMLIF-QKPISLSINF---LMPKLSLRSYCAYLSNAFSIFTALRVILKDN 372
AmelOr153       ----MQKNLLKXLIF-QRPVXIYRLZ---LLSKLILRYYCLYLSNVFSIFTALHVLEDNI 348
AmelOr144       ----MKSCVIFMMIRSQKLFLIRMSS---LLPPLSLEYCSNFVTTVSSYFMAMRTMIES- 373
AmelOr145       ----MKSCVIFMMIRSQKLFLIRMSS---LLPPLSLEYCSNFVTTISSYFMAMRTMIES- 374
AmelOr143       ----MKSCVIFMMMRSQKAFLIRMSN---LLPPLSLEYCSNFITTVSSYFMAMRTMIES- 368
AmelOr1         ----VAKNIKTIILRASRPSSLSGAK---ILE-LSLQAFCDVCKTSAAYFNFLRAMTV-- 400
AmelOr3         ----IAKNIRIIILRASRPSSLSGAK---MLD-LSLRVFCDVFKTSAAYLNFLRTMTV-- 403
AmelOr6         ----KARCLILIIIMSNYPVKLTAAK---VVD-VSLTTFTDVMKAAMGYLNMLREVI--- 394
AmelOr7         ----KARCLILIIIMSNYPVKLTAAK---VVD-VSLTTFTDVMKAAMGYLNMLREVT--- 394
AmelOr4         ----KARYLILMIIMSNYPIKLTAAK---VVD-VSLTTFTDVMKAAVGYLNMLREVI--- 396
AmelOr5         ----KARCLILMIIMSNYPVKLTAAK---VVD-VSLTTFTDVMKAAMGYLNMLREVI--- 396
AmelOr8         ----KARSLILLIIMSNYPIKVTAGR---LVD-VSLVTFTSIIKSAVGYMNILQQVT--- 396
AmelOr9         ----KARSLILLIIMSNYPLKVTAGR---IVD-VSLVTFTSIIKSAVGYMNILQQIT--- 396
AmelOr11        ----KARSLILLILMSHYPIKVTAGR---IMD-LSLVTFTSIIKSAVGYMNMLRTVT--- 394
AmelOr12        ----KARSLILLILMSNYPIKVTAGR---IVD-VSLVTFTSIIKNSVGYMNILQQVT--- 396
AmelOr10        ----KSRSLILLIIISNYPIKVTAGK---IID-LSLVTFITIIKTAVSYMNMLQQIT--- 395
AmelOr14        ----EARNLILVIIMSSYPVKLTAGK---VVD-ISLATFTDIIKTTVGYLNMLQKVT--- 398
AmelOr15        ----EARNLILVIIMSSYPVKLTAGK---VVD-ISLSTYTDIIKATVGYLNMLRKVT--- 399
AmelOr13        ----EARGLILVIIMSNYPIKVTAGK---IVD-ISLITFTDIVKTSVGYLNILRTVA--- 390
AmelOr16        ----NMRCLILIIAMSNYPMKLKAAK---MME-MSLTTFTDVMKMSMGYLNILREVI--- 401
AmelOr17        ----NMRCLILIIAMSNYPMKLKAAK---MME-MSLITFTDIMKVSMGYLNILREII--- 401
AmelOr18        ----KSLAIPLMISMSHSTTKITAGN---IIE-LSISSFGDVIKTSVAYLNMLRTFTT-- 411
AmelOr19        ----KSLAISLIISISRSTTKITAGN---IIE-LSISSFGAIIKTSFAYLNILRTLTS-- 411
AmelOr22        ----KKLCFVLIIAMSNSSIKLTAGN---MVE-LCLTTFSDIVKTAVAFLNVLRTLTI-- 412
AmelOr24        ----KKLCFVLIIAMSNSSIKLTAGN---MVE-LCLTTFSDIVKTAVAFLNVLRTLT--- 403
```

```
AmelOr20        ----KKLFCILIIAMSNSSIKLTAGN---IVN-LSISTFTDVVKTAVTYLNVLQKTT--- 407
AmelOr25        ----KKLCCILIIAMSNSSVKFTAGN---MVE-LSIYTFSDVVKTSVAFLNMFRALT--- 402
AmelOr29        ----KKLCCVLIIAMSNSSIKFTAGN---MVE-LSIYTFSDVVKTSVAFLNMLRALT--- 403
AmelOr30        ----KKLCCVLIIAMSNSSIKFTAGN---MVE-LSIYTFSDVVKTSVAFLNMLRALT--- 403
AmelOr21        ----KKLCCVLIIAMSNSSMKLTAGN---LIE-LSMSTFSDVVKTSFAFLNVLRTLT--- 403
AmelOr23        ----KKLCCVLIIAMSNSSTKLTAGN---LIE-LSMSTFSDVIKTSFAFLNVLRTLT--- 403
AmelOr35        ----KALSLILISAASNSSTKLTAGK---LVE-LSLSSFCSVLKSSLAYLSLLRTLTT-- 413
AmelOr27        ----TILNLILIILRSTVVVQITAGK---LFN-MSIYTFGDVLKTAFAYLNLLRQMT--- 405
AmelOr28        ----TILDLTLIIARSSVVVQITAGK---LIH-MSIQTFTDVIKTGFAYLNLLRQVT--- 405
AmelOr33        ----DILDLIQIILRSSMVIKITAGK---LVH-MSIYTFGNVMKTAFTYLNLLRQLT--- 407
AmelOr34        ----DILNLIQIILRSSMVIKITAGK---LVH-MSIYTFGNVMKTAFTYLNLLRQMT--- 407
AmelOr32        ----DILNLVQIILRSSMVIKITAGK---LVH-MSIYTFGNVMKTAFAYLNLLRQMT--- 407
AmelOr31        ----DILDLILIISRSNAVIKITAGK---LTN-MSIYTFGNVMKTTFTYFNLLRHVT--- 406
AmelOr36        ----DILNLIMIITRSSVEYKMTAGK---IID-MSVITFGNIIKTVFGYLNILRQVTML- 405
AmelOr37        ----DILNLIMIITRSSVEYKITAGK---IID-MSVITFGNIIKTVFGYLNILRQTTML- 406
AmelOr38        ----DILNLIMIITRSSVEYKMTAGK---IID-MSVITFGNIIKTVFGYLNILRQTTML- 407
AmelOr39        ----DILNLIMIIVRSSVEYKMTAGK---IMD-MSVITFGNIIKTVFGYLNILRQTTML- 407
AmelOr47        ----DILNLIMIITRSGVEYKMTAGK---IID-ISVITFGNIIKTVFAYLNILRQMTIL- 406
AmelOr48        ----DIFNLIMIIVRSGVEYKMTAGK---IID-ISVITFGNIVKTVFVYLNILRQMTIL- 406
AmelOr40        ----DILNLIMIITRSSVEYKMTAGK---IID-MSVITFGNIIKTIFAYLNILRQMTIL- 406
AmelOr43        ----DILNLIMIMIRSGMEYKMTAGK---IIN-MSVVTFGNIIKTILAYLNILRQMTIL- 407
AmelOr45        ----DILNLIMIITRCSMEYKMSAGK---MID-MSVITFGNIVKTIFAYLNILRQMTIL- 406
AmelOr41        ----DILNLIMIIIRSSVEYKMTAGK---IID-MSVITFSNIIKTIFAYLNILRQVTIL- 406
AmelOr42        ----DILNLIMIIIRSSVEYKMTAGK---IID-MSVITFSNIIKTIFAYLNILRQVTIL- 406
AmelOr44        ----DILNLIMIMIRSGVEYKMTAGK---IVN-MSVITFGNIIKTIFTYLNILFQMTML- 406
AmelOr46        ----DIFNLMMIISRSSISVNMSAGK---LID-MSVLTFGNTVKSVFVYLNMLRQMTMI- 405
AmelOr49        ----NILNLMMIITRSSMNINMTAGK---MTN-MSVLTFGKIVKSIFAYLNVLRQITMI- 406
AmelOr50        ----DILNLIMIISRSSVEVKITAGK---IIT-MSIYTFGNIVKTVFAYLNMLRQITMM- 408
AmelOr26        ----TALGLVLIISRSSMVIKITAGK---LIQ-ISIATFAAVFKASFAYLNMIRTIAM-- 410
AmelOr53        ----EAYDLILLISISQHPPKLTAGK---IIE-LSLNTFSTVAKTSVVYLNLLRTVTDW- 412
AmelOr54        ----EAYDLILLISISQYPPKLTAGK---IIE-LSFNTFSSVAKTSVVYLNLLRTVTDW- 410
AmelOr52        ----KAHDLILLISISQYPPKLTAGK---IID-LSFNTFSSVVKTSVIYLNLLRTVTD-- 406
AmelOr51        ----KARDIILLLVISKYPPKLTAGK---IFI-LSMNTFSVVLKSSVVYLNMLRTITEL- 409
AmelOr55        ----TIQNLILIMAMSNSPAKLSVGR---IVD-LSLSTFGNVLKTTFVYLNFLQTAVMQ- 408
AmelOr57        ----TIQNLILIMAMSNTPAKLSVGR---ILD-LSLSTFGNVLKTTFVYLNFLQTAVM-- 407
AmelOr56        ----TIQNLILIMAMSNTPAKLTVGR---IVD-LSLSTFGNVLKTTFVYLNFLQTAVMQ- 408
AmelOr58        ----TIQGLILMIAMSNSPAKISAAG---IAD-LSLSTFGSVLKTSFAYLNFIRTTIM-- 409
AmelOr59        ----TIRGLILMIAISSNPTKISAGG---IVD-LSLSTFGNVLKTSFAYLNFFRTTIM-- 410
AmelOr60        ----TIQGLILIIAISNSPTKISAGG---IVD-LSLFTFANILKTSFVYLNFIRAAIM-- 410
AmelOr61        ----TIHDLILIIAMSNNPIKISAGS---IVD-LSLYTFGGVLKTSLVYLSFLRTTIM-- 410
AmelOr63        ----VSHNITFIIMRAQHPVLLTAGK---FFV-INMETYMSILRISMSYLSVLRVMVNS- 397
AmelOr64        ----VTQNIIFIIMRAQHPVFLTAGK---FFV-VNMETYMSILKTSMSYLSVLRVMVNS- 393
AmelOr66        ----VSQNITFIIMRAQHPFLLTAGK---FFV-VNMETYMSILKTSMSYLSVLRVMVN-- 392
AmelOr65        ----VSKDIIFIIMKAQRPVLLRAGK---IFV-VNMETYISILKTSMSYLSVLRVMVNS- 393
AmelOr67        ----VSKDIIFIIMKSQRPVLLTAGK---FFV-INMETYMSILKTSMSYLSVLRVMVNS- 393
AmelOr114       ----ITKMLIIVMCRAKSPLTVTAGR---FCS-FTLQLFSEVLKTSMRYLSVLYAVKDKI 373
AmelOr115       ----RAVLLMIVIRRANVSFQITAGK---FSP-FSLEFFNAVLKTSAGYLSVLLAMKDRL 396
AmelOr118       ----DVRIMLMIILRSQKQLTLTAGK---FIC-LSLEAFANMLKASASYVSVLYARY--- 371
AmelOr164       ----EIRVLLLLIMRSQKPLTLTIGK---YMK-LSLETFANMLKISASYASVLYALE--- 383
AmelOr163       ----DSRIVLLILIQAQRKLILSAGN---FVT-LSVQTFASMQKVSASYISILMTIY--- 385
AmelOr165       ------------------------------------------------------------
AmelOr166       ----ESRLLIPIILRAQRGFTFTFGK---FAT-LSMESFTA------------------- 358
AmelOr167       ----NRGVMILLMVRSQRRLTITAGK---FMD-LSMQGFAN------------------- 369
DmelOr35a       ENLRLLKLINLAIEMNSKPFYVTGLK---YFR-VSLQAGLKILQASFSYFTFLTSMQRRQ 406
DmelOr74a       ENVKLMKLVTLAIQLNSRPFFITGLN---YFR-VSLTAVLKIIQGAFSYFTFLNSMR--- 404
AgamOr3         ----MRRKLRLLLQRSQKPLGVTAGK----FRFVNVAQFGKMLKMSYSFYVVLKEQF--- 408
AgamOr5         ----IQRKLRMVLQRAQKPVGISAGK----FCFVDIEQFGNMAKTSYSFYIVLKDQF--- 389
AgamOr13        ----IQKDLQLIIARAQRPVGITAGK----FCYMNMEQLGIIVKTTYSIYIILRDQF--- 388
AgamOr15        ----IQKDLQLILVRAQSPVGITAGK----FYYMNMEQFGIIVKTTYSFFVILRDQI--- 387
AgamOr17        ----VQKDLQVIIARAQKTVGITAGK----FCYMNMAQLGIIVKTTYSFFVILRDQI--- 387
AgamOr16        ----LRKDLQLIMLRAQRSVGITAGK----FCFMNMEQFGEVVKTTYSFFVVLRDQF--- 385
AgamOr55        ----IAKHLQLILLRAQKPIGITAGK----FCFINMEQFAKLLKTTYSVFILLRDLL--- 388
AgamOr18        ----MRKHISFMIMRSQRRVGLTAAK----FCFVDMEQFGAMLNMSYSFFVVLKDAF--- 395
AgamOr14        ----IQQGLRMVLHRTQSPVGIQAGK----FCFVDVELFQNMVNKSYSFFIVLKDAF--- 409
AgamOr51        ----IQQRLRMVLHRTQSPVGIQAGK----FCFVDMELFQKMVNKSYSCFIVLKDAL--- 397
AgamOr12        ----IQKTVAMVIARGQARLRITAWG----IIPIDLELFAKVVKASYTVLLVLKDFI--- 398
AgamOr19        ----IQKTVAMVIARGQARLRITAWG----IIPIDLELFAKVVKASYTVLLVLKDFI--- 398
AgamOr20        ----IQKMISTIVARSQLPLRITACG----FITVNVELFAKVVKTTYSGFIVLKDFI--- 398
AgamOr21        ----VQKMVAMIIARSQTPLQITACG----FIPINLELFTKVVKHSYTVLAVLKDLI--- 395
AgamOr50        ----VQKMVAMIIARGQAPLQIKACG----FIPINLELFAKVVKTSYSVLIVLRDFV--- 397
```

```
AgamOr29        ----IQKQLLFMIRRSQKPIILSAGK----IFYANVLQFSEMVQKSYSFYLVLKNVF--- 386
AgamOr53        ----IQRQILLMIRRSQTPLILRAGK----LFSANVVQFGDIVQKSYSFFLVLKNVF--- 376
AgamOr30        ----IQKQIYFMIHRSQTRIELTAGK----LFPVNIAQFSEIVKKSYSYYLVLKDIF--- 387
AgamOr46        ----LQKMVVPIIQQSQQRIGITAAK----FYYIDYNRYGQSLKTAYSFYLLLKDIF--- 387
AgamOr47        ----LQKMVVPIIQQSQQRIGITAAK----FYYIDYNRYGQSLKTAYSFYLLLKDIF--- 387
AgamOr48        ----IRKYFLRIILQAHRKATITAGK----FYNVNIVTFAQLIKTSYTYYMIMKEMF--- 397
AgamOr49        ----LQKQFQQMIRHAYIPRGITVGK----FHFVDMASFGQLLKAIFSYYLILKELF--- 404
AgamOr61        QYVTVRHTLMLVIGRSQKGFQCSYGG----LGSISMERFAQLMQKSYSLLTILLQFAK-- 419
AgamOr62        QYFSVRRTILLVILQSQQSLRFSYGA----HGEISMHSFAELMQKSYSMLTFMLQFQN-- 416
AgamOr63        QYRHVRRTLLLLTAQSDQTIQFSFAG----IGEISMNSFAQLLEKSYSMLTFLLQFAK-- 412
AgamOr60        AYRQMRASFLIIIIRSQKPLSFSISA----AGTISMARFADLLNSSYSLMTVMFQLKERI 415
AgamOr75        -GRTIKRPMMLMIMQANMTKDFSAGG----LTTVSAELFAKTCRMIYTMMMFMANMAT-- 411
AgamOr76        -GRTIKRPMMLMIMQANMTKDFSAGG----LTTVSAELFAKTCRMIYTMMMFMANMAT-- 411
AgamOr78        -GRTIKRPMMLMIMQANMTKDFSAGG----LTTVSAELFAKTCRMIYTMMMFMANMAT-- 412
AgamOr77        ----SKPFLLITIRMAQVPLRFMCGR----MYQLSTELFTSVVQFIYSLIMMLLQFK--- 386
AgamOr79        ----SRPFLLITIRMAQVPLRFMCGG----MYQLSTELFASVVQFIYSLIMMLLQFK--- 384
AmelOr99        ----IQKMLYMMQIRSKKLCALTAGG----LYDMNIENFGITFKTCMSYFTMIMSFK--- 404
AmelOr101       ----IQKMLYMMQIRSKKLCALTAGG----LYDMNIENFGITFKTCMSYFTMIMSLK--- 404
AmelOr102       ----IQKVLYIMQIRSKKLCTLSAGG----LYEMNIENFGITFKTCMSYFTMIMSLK--- 403
AmelOr103       ----IQKVLYVMQIRSKKLCTLSAGG----LYEMNIENFGTTFKTCMSYFTMIMSLK--- 403
AmelOr104       ----IQKVLHMMQIRSKKPCSLTAGG----LYEMNMENFGITFKTCMSYFTMLMSLKK-- 405
AmelOr98        ----IQKMFNIMLMRSKKSCALTVYG----LYELNMENFGTTFKACISYFTMMLSLK--- 401
AmelOr100       ----VQKMFYVMQIRCKKPCSLTACG----LYEMNMENFGTALKTCMSYITMILSLK--- 324
AmelOr97        ----VQKIFHIIQIKSSKPCKLTAGG----ILELNLENFGIALKTCMSYFTIFLSLQD-- 349
AmelOr105       ----VQKVLHIMQIRSNKPCILTAAG----LYEMKIESFGITIKTCMSYFMMFLSLRE-- 405
AmelOr109       ----MQSLYVLVLRKCLSPPKLTGGG----FVALNLDSFVQILKASFSYYTVFRS----- 390
AmelOr110       ----TQSLYVLVLRKCLTFPKLTGGG----LIILNLNSFVQILKASFSYYTVFRS----- 389
AmelOr111       ----TQSLYILVLRKCLSPPKLTGGG----LVALNLDSFLQILKASFSYYTVFRS----- 390
AmelOr108       ----TQSLYVLVLRKCLNPPTLTGGG----LIVLNLDSFVQILKLSFSYYTVFRS----- 390
AmelOr107       ----TQSLYLLVLRKCLNPPKLTGGG----LIALNLDSFVKVLKTSFSYYTVFRSS---- 391
AmelOr112       ----IRSLYVLSLRSCLNFPTLTAGG----LIVLNLQSFAEIIKAAVSYYTVMQTT---- 388
AmelOr113       ----IQALYILALRRSLTPPRLTAGG----LIELNMQSFSEVIKLSISYYTVLRST---- 389
AmelOr106       ----IQAMFVLILRRNLNPPLLTAGG----LMQLNLNTFAQVVKTSVSYFTVLKSV---- 387
AmelOr122       ----AQKLLLFMILRSSMGCEICLSG----LFTPSYAGLTSMMSSSFSYCTVIYSIQ--- 363
AmelOr125       ----AQKLLLFMILRSSMDCELRLSG----LFTPSYAGLTSMMSSSFSYCTVIYSIQ--- 362
AmelOr123       ----AQKLLLFMILRSSMGCEICLSG----LFTPSYAGLTSMMSSSFSYCAVIYSIQ--- 360
AmelOr126       ----AQKLLLFMILRSSMGCEICLSG----LFTPSYAGLTSMMSSSFSYCAVIYSIQ--- 360
AmelOr127       ----AQKLLLFMILRSSMGCEICLSG----LFTPSYAGLTSMMSSSFSYCAVIYSIQ--- 360
AmelOr128       ----AQKLLLLMMLRSTVGCELHLSG----LFTPSYAGFTSMMSSSFSYCAVIYSIQ--- 360
AmelOr129       ----AQKLLLFMMFKSSVGCELRLCG----LFTASYAGFTSMMSSSFSYCAVIYSIQ--- 360
AmelOr133       ----VQKILLLIMIRSSMACIFHICG----VFVPCHAGFTTMLSTSFSYFTLMYSIQ--- 265
AmelOr134       ----IQKILLLIMIRSSTACMFHIFG----VFVSCHAGFTTMLSTSFSYFTLMYSIQ--- 361
AmelOr131       ----VQKILLLIMIRSSMACIFHIFG----VFIPCYVGFTTMLSTSFSYFTLIYSIQ--- 363
AmelOr132       ----VQKILLLIMTRSSTTCMFHILG----VFVPCYTGFTTMLSTSFSYFTLMYSIQ--- 363
AmelOr135       ----VQKILLLIMIRSSTRCMFHILD----IFTPCYAGFSKMLSTSFSYFTLIYSMQ--- 361
AmelOr136       ----VQKILLLIMIRSSTACMINILG----VFTPCYIGFSKMLSTSFSYFTLMHSIQ--- 363
AmelOr138       ----VQKILLLIMIRSSTSCTFHILG----VFIPCYTGFSKILSTSFSYFTMIYSIQ--- 269
AmelOr130       ----AQKILLLIMLQSTTKHAFNILG----LFTPCYAGFSTMLSSSFSYFTLMYSIQ--- 363
AmelOr137       ------------------------------------------------------------
AmelOr139       ----VZKILLLIMIQSSITYMISILG----VFILCHIGFSTMLNTSFSYFTLIXSTQ--- 285
BmorOr20        -----RKTVAIFLMNVQEPLHVNALG----LAKVGVQSMAAILKTSFSYFTFLRTVSE-- 439
BmorOr3         -----RKTVAIFLMNVQEPLHVNALG----LAKVGVQSMAAILKTSFSYFTFLRTVSE-- 439
BmorOr18        -----RKTVAIFLMNVQEPLHVNALG----LAKVGVQSMAAILKTSFSYFTFLRTVSE-- 439
HvirOr6         -----RKVVMFFLMNVQEPVHVKAMG----LANVGVTTMASILKTSLSYFTFLLSQTKEE 431
BmorOr23        -----RKMVYMMFRQSQIPLQLKAMN----MLSIGVKTMVSILKTSVTYYLILKTVTTD- 417
BmorOr16        -----RKMVYMMFRQSQIPLQLKAMN----MLSIGVKTMVSILKTSVTYYLILKTVTTD- 417
BmorOr4         -----RKMVYMMFRQSQIPLQLKAMN----MLSIGVKTMVSILKTSVTYYLILKTVTTDE 417
BmorOr5         -----RKMVYMMFRQSQIPLQLKAMN----MLSIGVKTMASILKTSVTYYLMLKTITANE 418
BmorOr22        -----RKLVQVLLQKSQKPIQFKAMN----MMSVGVQTMASIIKTSISYFIMLRTIARD- 424
BmorOr17        -----RKLVQVLLQKSQKPIQFKAMN----MMSVGVQTMASIIKTSISYFIMLRTIARD- 424
BmorOr1         -----RRVVYGFLRRTQNPVRFKAMG----MLDVGVQTMASILKTSISYFVMLRTVAT-- 430
BmorOr1a        -----RRVVYGFLRRTQNPVRFKAMG----MLDVGVQTMASILKTSISYFVMLRTVAT-- 430
BmorOr1b        -----RRVVYGFLRRTQNPVRFKAMG----MLDVGVQTMASILKTSISYFVMLRTVAT-- 430
BmorOr19        -----QKIFVVFLQRTQPDLEFETVC----GMKAGVKPAFSIVKSMFSYYVMINSRF--- 407
BmorOr15        -----QKIFVVFLQRTQPDLEFETVC----GMKAGVKPAFSIVKSMFSYYVMINSRF--- 407
TcasOr5         -----FTVYVLWIPMALGFIALTVWY------------FEDILHHVGLQLVIL------- 299
TcasOr7         -----CEAKLLLR-GKVNPLKFTICG------------FLPLDYCLAYTVS--------- 311
HvirOr4         -----VLPSRLEIFSRQLMLQSVSYAP-MGMCTLHRPLIASVIGAVTTYLVILIQFQRYD 396
BmorOr13        -----PISIELEMFFRQLVLNKASYAP-LKVCTLTRSLVATILGSITTYLIVIVQLEIKN 443
DmelOr10a       -----RRLVQLLILRSQRPVSMAVPF-----FSPSLATFAAILQTSGSIIALVKSFQ--- 406
```

```
DmelOr33c     ----FDLLIFTQLTLGNRGWIIKAGG----LIELNLNAFFATLKMAYSLFAVVVRAKGI- 384
DmelOr7a      ----FRKMLLYYLHRAQQPITLTAMK----LFPINLATYFSIAKFSFSLYTLIKGMNLGE 407
DmelOr22c     -----RKVILMILRRSQRAKTIAVPF-----FTPSLPALRSILSTAGSYITLLKTFL--- 402
AgamOr1       -----SQAMIFFLQMTLKDVHIKVGS--VLKVTLNLHTFLQIMKLSYSYLAVLQSMESE- 417
AgamOr4       -----RSSIRMMLRQSQRHAHITVGK----FFRVNLEEFSRIVNLSYSAYVVLKDVIKMD 394
DmelOr47b     ----IQRDISFVILRAQKPLMYVAEP----FLPFTLGTYMLVLKNCYRLLALMQESM--- 412
AmelOr73      ----VMKDLLLVIARSKKPVKITSGQ----IFILSTESFMKIMKISYSSFNILKNSTMK- 365
AgamOr68      -----QKEYKLLLLRAQQPSGMSIAG----LTPVNYETYTQIMKMLYQLFALAMNFLK-- 390
DmelOr59a     ----FKRKMMLFVEQSLKKSTAVAGG----MMRIHLDTFFSTLKGAYSLFTIIIRMRK-- 379
DmelOr23a     -----RGHMLILAERTKRMQLLLAGN----LVPIHLSTYVACWKGAYSFFTLMADRDGLG 378
AgamOr31      -----QKMLINCMMRAKKPVNAKSGFTQASLPTLNAVWWQYILNSAGSYVALLMSLME-- 377
DmelOr2a      -----KRELLFTLARTQRPSLIYAGN----YIALSLETFEQVMRFTYSVFTLLLRAK--- 397
AmelOr62      -----ARDLILIICRGNSSYHVTAGK----FVFMNIFTFKEILKSSASYLSVLKVMMDT- 399
AmelOr121     -----AKSLIIIMARSKKPLYLTAGK----LFPVTMLTFCNILKISLSYISFLLTIL--- 393
AmelOr140     -----QNLYKFLLARSLIFSKLTAFK----VTTLSMQTFLAIIQTAMSYFTVLLSTT--- 359
TcasOr12      -----KQNLLFLMTRTQFPLKLYASG---------------GKVTQSYLLNLE------ 374
AgamOr8       -----QRSLLFVMLRSTTVQKLTALK----FSIVSLASYSKILSTSFSYFTLLKAMYEPN 394
BmorOr9       -----KKTILTIMIRSHKPKKLTAYK----FSVISYGSFSKIISTSWSYFTILRTMYTPP 380
AgamOr40      -----RRRLVIILMNAQRPVRLTGLK----LYELNLETYYTVLKAAFSYYTIIKKFR--- 454
TcasOr63      ----IRKIVMIFLEKTKKPTIVKAGN----FITLSLATLTQILRSAYSYFAVLQRLYKDS 383
TcasOr4       ----VASAEFNLLKGALKTIDFHPKG------HNTKQLIEAKFENCVNHHKEIVK----- 378
TcasOr13      -----KSLEFFMTYLAECQIRFTAYG----FTKLHRGLLTTITGAVTTYLVILFQFN--- 405
AgamOr24      -----MKFMKMVLMMSQKPKMLMAAT-----LPLNITAFLQIHKFIYSLIMMLENTKG-- 377
AgamOr54      -----QKAMRLLLMASQNPGRLSYGFG-----TVNMRAFFEIFRKTYSIAMMMISVNEEE 366
AgamOr23      -----QKTLQQILLTSQHPVVLSDGFS-----PIDLFNFVEIYKKIYSYLMVLQKVS--- 380
AgamOr64      TLRPLRRSILIVLQQTLRPLRFGYGVS----GSLSMQRFGEFMQQIYSLIMFLAQLN--- 418
DmelOr82a     -----RTSLSLIILQSQKEVLIRAGF-----FVASLANFVGICRTALSLITLIKSIE--- 384
AmelOr116     ----FNYALLIIMIRTQIPLIIIVGK----VMELSLQNFLLILRTSYSIFTLLKTFTT-- 399
AmelOr160     -----TRSLIFCIARAQKPLYLTAGK----FYVFSLETFAVIVKASMAYLSVLKSII--- 390
TcasOr10      -----KTARLVIMNKAQSPVKLTIGK----FTGMDLRTFLLV------------------ 330
TcasOr26      -----QKGVILITIRAQKPVRIYAGP----FSYVSHLLILTVFQTSYSYINLLNASS--- 338
AmelOr117     -----QKYLYFMIVRSQQYCRITALT----FQTLSICTLSNVFNTSVSYFTLLRQMQQ-- 400
AmelOr120     -----KDEICMIILRSQRPSFITAGK----LYIMHLENFTAILSTAFSYFMMLQSFNTEA 403
BmorOr7       ----FQRGLLVLLERARRPVRPAAGL----VIPLSLQTFLKIIKSSYTFYAVLRQTK--- 390
AgamOr6       ----FHRSVLQIIHRSQQSVILTAWK----IWPIQMSTFSQILQASWSYFTLLKTVYGN- 404
DmelOr45a     -----RRLWQMVIMRAQRPAKIFGFM-----FVVDLPLLLWVIRTAGSFLAMLRTFER-- 378
DmelOr67d     -----EKLIIMMLAKAQNEVVLTAAD----MAPLSMNTALQLTKGIYSFSMMLMNYLG-- 391
DmelOr83c     -----RKLFGFLLRESQYPHNIQILG----VMSLSVRTALQIVKLIYSVSMMMMNRA--- 397
AmelOr162     -----RKLLAIMLSKSIAPLRLTACK----LYTLNLESFTTIAKTSVSYTMVLCSLQ--- 368
HvirOr3       -----QKDLLIVILAAQRPLKLSAGG----MALLCIQTFSQALYNGYSIFAVLNDAVN-- 269
TcasOr20      -----QRYVLLILLRSQKPLTLRACG----VKVMSLATFLGVLYSAYSYFTLLLKLKP-- 386
AgamOr11      -----NRMLQIFILYSNRPLKMHAFF-----ISMSLDTFLAILRASYSYFTILKQLPTNF 425
DmelOr56a     ----LQKMLVFMMMHAQRPMKMRALL-----VDLNLRTFIDIGRGAYSYFNLLRSSHLY- 419
AgamOr28      -----QKDILTFMVKAQKPTGVTASK----FYMVTLQTFQRISSTSYSYFTLLQTINQQ- 420
AgamOr58      RCRRIRSSILIVMTRAEHELRISCGS----VYDMSLTTCWAVLQFSYSVFTLLLSFFENE 399
AgamOr59      EYRSAALTIRLLIMRSQHRVRFTCGS----INPVSMEKFTEFLNLSYSIVMFLLSIN--- 415
DmelOr1a      -----RKLFLFPIERAQRETVLGAYF-----FELGRPLLVWIFRTAGSFTTLMNALYAKY 389
DmelOr13a     TTKRIRNQIWFMILCSQQPVRITAFK----FSTLSLQSFTAILSTSISYFTLLRSVYFDD 413
AmelOr161     -------SVQIMMMRSKIPSKITAAK----FYSMSLESFSAVLSTSFSYFTVLTATKNE- 385
AmelOr141     ----LQKITNILMIRSQKAVRLTAGK----FIGLSLETFASMISTAASFFTMVRSMN--- 429
AgamOr52      ALREFRTTLSIILLATQRSLSLRGSD----IVEVSWQTFASMLKTSYSVMMFLIELRRLN 386
AgamOr36      -YRQARSSLLIMMSKTQKSLGIRCGG----MFEMSSEAFASLVKLTYTMLMFLRDTQKPN 407
AgamOr25      -----QKLLMVMIAHAQKPQVLRGIF-----MPLIMSSFLSVIKASYSYFTLLH------ 357
AgamOr45      -----QKRLMFLLANAQPEIVMGAVF-----IPVTMTSFVTIIRAAYSYFTILY------ 383
TcasOr3       ----FKKQIIFTMTRMQKPINVTIGK----ITPLAFSTFLTIARGAYSFFTFLKQRHGIN 412
DmelOr88a     -----RKFYLLWTQYCQRTQQLGAFG----LIQVNMVHFTEIMQLAYRLFTFLKSH---- 401
AgamOr22      ----DLHDILFMLRNAQRNYGFSIGG----FGFLSFATFTAVMKTAYSCNAFLHRVMN-- 388
DmelOr49a     -----KKEILILMAQAQRPLEISARG----VIIISLDTFKILMTITYRFFAVIRQTVEK- 396
DmelOr85f     -----KRYLLIIIIRAQQPVELNAMG----YLSISLDTFKQLMSVSYRVITMLMQMIQ-- 392
AmelOr119     ----IGQVFTLSTVFTRKSIILRASV----FYVLSLETFIAIIKRSYSFFTLLNNMDLTD 416
DmelOr63a     ----FRHLIRMMLMRTNRGFRLDVSW----FMQMSLPTLMAMVRTSGQYFLLLQNVNQK- 420
DmelOr67b     ----FKFMIKMMLLFSRRTFVLSVGG----FTSLSHKFLVQVFRLSANFFLLLRNMNNK- 421
BmorOr21      ----IKRMMPFMVARSQVIVEITAFN----MFAFDMELFVWIMKTSYSMFTLMRS----- 390
BmorOr11      ----IRQMLLFMLTRATEPLGIATVF-----TNNSLDTFAEMCRQSYTIFNLMNAAWA-- 397
BmorOr12      --ARIRSLVTISMNKAQQPILMWALG----FVELSHKNFVAIIKSAYSVFSVFY------ 401
AgamOr33      ----LKKDLRFTTMRSQKPFVIDVYW----LFPLTYETFIAILSRSYSIFTLLRTMIE-- 373
HvirOr1       KVALSGLQFFSVTRGLLLTVAGTIVTYELVMFQFNSSTPSLNITSPTSATHIITTLAT-- 457
HvirOr5       -----VEVERLQDQLTKDYIALSGMG----FFYLNKTILLQMAGAIVTYELVLIQFDDQG 332
TcasOr1       ------IFIICVIVQSEQIAWLHLLY----IFLVGIMYAADVFYICHVCCSTIHEVRE-- 353
TcasOr2       ------------------------------------TIEEVISGI-------------- 314
```

```
TcasOr16        ------------------------------------------------------------
AmelOr2         ------------------------------------------------------------
AgamOr7         ------------------------------------------------------------
AaegOr7         ------------------------------------------------------------
DmelOr83b       ------------------------------------------------------------
BmorOr2         ------------------------------------------------------------
BmorOr2a        ------------------------------------------------------------
HvirOr2         ------------------------------------------------------------
AgamOr56        VN---------------------------------------------------------- 400
AgamOr57        MG---------------------------------------------------------- 400
AgamOr26        AN---------------------------------------------------------- 400
AgamOr27        VN---------------------------------------------------------- 400
HvirOr9         ------------------------------------------------------------
HvirOr7         ------------------------------------------------------------
DmelOr85b       ------------------------------------------------------------
DmelOr85c       ------------------------------------------------------------
DmelOr85d       ------------------------------------------------------------
DmelOr67a       ------------------------------------------------------------
DmelOr67c       ------------------------------------------------------------
DmelOr92a       ------------------------------------------------------------
DmelOr69a       ------------------------------------------------------------
DmelOr69b       ------------------------------------------------------------
TcasOr6         CLQFLAVKIIFKIPKPIAISFGETKGLEPLYDHLYLVLHAMITLVTINLVNGFDGLFFYF 433
TcasOr11        ------------------------------------------------------------
AgamOr41        ------------------------------------------------------------
AgamOr42        ------------------------------------------------------------
AgamOr43        ------------------------------------------------------------
AgamOr44        ------------------------------------------------------------
AgamOr66        G----------------------------------------------------------- 393
AgamOr67        G----------------------------------------------------------- 393
AgamOr72        ------------------------------------------------------------
AgamOr73        ------------------------------------------------------------
AgamOr71        ------------------------------------------------------------
AgamOr74        ------------------------------------------------------------
AgamOr70        ------------------------------------------------------------
AgamOr69        ------------------------------------------------------------
TcasOr9         ------------------------------------------------------------
TcasOr18        KD---------------------------------------------------------- 410
DmelOr83a       ------------------------------------------------------------
DmelOr85e       TESEL------------------------------------------------------- 467
TcasOr24        ------------------------------------------------------------
TcasOr25        FYIKLAVLLTVSVIILYVPVCIHIHKLFERDLDASEDFALMFSYYGFLSTMIFYSKNHKK 444
TcasOr19        ------------------------------------------------------------
TcasOr22        ------------------------------------------------------------
TcasOr23        ------------------------------------------------------------
AmelOr168       ------------------------------------------------------------
AmelOr170       ------------------------------------------------------------
AmelOr169       ------------------------------------------------------------
DmelOr65b       ------------------------------------------------------------
DmelOr65c       ------------------------------------------------------------
DmelOr65a       ------------------------------------------------------------
TcasOr17        ------------------------------------------------------------
TcasOr21        ------------------------------------------------------------
TcasOr56        ------------------------------------------------------------
TcasOr15        ------------------------------------------------------------
AgamOr9         ------------------------------------------------------------
AgamOr65        ------------------------------------------------------------
BmorOr6         HVLDQQKSAENKDSK--------------------------------------------- 406
BmorOr10        ------------------------------------------------------------
AgamOr34        ------------------------------------------------------------
AgamOr37        V----------------------------------------------------------- 393
DmelOr94a       ------------------------------------------------------------
DmelOr94b       ------------------------------------------------------------
DmelOr71a       ------------------------------------------------------------
DmelOr46a       ------------------------------------------------------------
DmelOr46b       ------------------------------------------------------------
HvirOr8         ------------------------------------------------------------
BmorOr8         EE---------------------------------------------------------- 396
```

```
AmelOr68        ------------------------------------------------------------
AmelOr69        ------------------------------------------------------------
AmelOr70        ------------------------------------------------------------
AmelOr71        ------------------------------------------------------------
AmelOr72        ------------------------------------------------------------
DmelOr19a       LE---------------------------------------------------------- 387
DmelOr19b       LE---------------------------------------------------------- 387
DmelOr33a       ------------------------------------------------------------
DmelOr33b       ------------------------------------------------------------
DmelOr22a       RFQ--------------------------------------------------------- 397
DmelOr22b       KFQ--------------------------------------------------------- 397
DmelOr42b       KFKTD------------------------------------------------------- 399
DmelOr59b       QFQ--------------------------------------------------------- 398
DmelOr59c       KFFSDRSNGDINP----------------------------------------------- 411
DmelOr98a       RLTKN------------------------------------------------------- 397
DmelOr42a       KLAKSEMEE--------------------------------------------------- 406
DmelOr85a       KLRRE------------------------------------------------------- 397
DmelOr43b       KLSIKE------------------------------------------------------ 403
AgamOr2         ------------------------------------------------------------
AgamOr10        ------------------------------------------------------------
DmelOr30a       ------------------------------------------------------------
DmelOr49b       ------------------------------------------------------------
DmelOr43a       ------------------------------------------------------------
AgamOr32        NEKKT------------------------------------------------------- 384
AgamOr35        ------------------------------------------------------------
DmelOr47a       ------------------------------------------------------------
DmelOr98b       ------------------------------------------------------------
DmelOr9a        ------------------------------------------------------------
AgamOr38        ------------------------------------------------------------
AgamOr39        ------------------------------------------------------------
DmelOr24a       ------------------------------------------------------------
DmelOr45b       ------------------------------------------------------------
AmelOr74        ------------------------------------------------------------
AmelOr86        ------------------------------------------------------------
AmelOr75        M----------------------------------------------------------- 401
AmelOr84        ------------------------------------------------------------
AmelOr87        ------------------------------------------------------------
AmelOr76        KSDSS------------------------------------------------------- 408
AmelOr85        IMDA-------------------------------------------------------- 412
AmelOr88        LQYACIANQSLLKEEESKIIFYHQNI---------------------------------- 432
AmelOr91        N----------------------------------------------------------- 408
AmelOr92        IMKHD------------------------------------------------------- 409
AmelOr95        S----------------------------------------------------------- 404
AmelOr93        S----------------------------------------------------------- 380
AmelOr77        ------------------------------------------------------------
AmelOr94        ENT--------------------------------------------------------- 405
AmelOr96        ------------------------------------------------------------
AmelOr79        NGL--------------------------------------------------------- 405
AmelOr83        ------------------------------------------------------------
AmelOr80        S----------------------------------------------------------- 406
AmelOr81        ------------------------------------------------------------
AmelOr82        NDT--------------------------------------------------------- 407
AmelOr78        ------------------------------------------------------------
AmelOr89        NEA--------------------------------------------------------- 404
AmelOr90        KDD--------------------------------------------------------- 405
AmelOr142       VN---------------------------------------------------------- 375
AmelOr146       EN---------------------------------------------------------- 374
AmelOr148       ------------------------------------------------------------
AmelOr158       ------------------------------------------------------------
AmelOr147       ------------------------------------------------------------
AmelOr150       ------------------------------------------------------------
AmelOr151       P----------------------------------------------------------- 373
AmelOr152       PS---------------------------------------------------------- 374
AmelOr154       SA---------------------------------------------------------- 374
AmelOr155       ------------------------------------------------------------
AmelOr157       ------------------------------------------------------------
AmelOr156       SI---------------------------------------------------------- 374
AmelOr153       ------------------------------------------------------------
AmelOr144       ------------------------------------------------------------
AmelOr145       ------------------------------------------------------------
```

```
AmelOr143       ------------------------------------------------------------
AmelOr1         ------------------------------------------------------------
AmelOr3         ------------------------------------------------------------
AmelOr6         ------------------------------------------------------------
AmelOr7         ------------------------------------------------------------
AmelOr4         ------------------------------------------------------------
AmelOr5         ------------------------------------------------------------
AmelOr8         ------------------------------------------------------------
AmelOr9         ------------------------------------------------------------
AmelOr11        ------------------------------------------------------------
AmelOr12        ------------------------------------------------------------
AmelOr10        ------------------------------------------------------------
AmelOr14        ------------------------------------------------------------
AmelOr15        ------------------------------------------------------------
AmelOr13        ------------------------------------------------------------
AmelOr16        ------------------------------------------------------------
AmelOr17        ------------------------------------------------------------
AmelOr18        ------------------------------------------------------------
AmelOr19        ------------------------------------------------------------
AmelOr22        ------------------------------------------------------------
AmelOr24        ------------------------------------------------------------
AmelOr20        ------------------------------------------------------------
AmelOr25        ------------------------------------------------------------
AmelOr29        ------------------------------------------------------------
AmelOr30        ------------------------------------------------------------
AmelOr21        ------------------------------------------------------------
AmelOr23        ------------------------------------------------------------
AmelOr35        ------------------------------------------------------------
AmelOr27        ------------------------------------------------------------
AmelOr28        ------------------------------------------------------------
AmelOr33        ------------------------------------------------------------
AmelOr34        ------------------------------------------------------------
AmelOr32        ------------------------------------------------------------
AmelOr31        ------------------------------------------------------------
AmelOr36        ------------------------------------------------------------
AmelOr37        ------------------------------------------------------------
AmelOr38        ------------------------------------------------------------
AmelOr39        ------------------------------------------------------------
AmelOr47        ------------------------------------------------------------
AmelOr48        ------------------------------------------------------------
AmelOr40        ------------------------------------------------------------
AmelOr43        ------------------------------------------------------------
AmelOr45        ------------------------------------------------------------
AmelOr41        ------------------------------------------------------------
AmelOr42        ------------------------------------------------------------
AmelOr44        ------------------------------------------------------------
AmelOr46        ------------------------------------------------------------
AmelOr49        ------------------------------------------------------------
AmelOr50        ------------------------------------------------------------
AmelOr26        ------------------------------------------------------------
AmelOr53        ------------------------------------------------------------
AmelOr54        ------------------------------------------------------------
AmelOr52        ------------------------------------------------------------
AmelOr51        ------------------------------------------------------------
AmelOr55        ------------------------------------------------------------
AmelOr57        ------------------------------------------------------------
AmelOr56        ------------------------------------------------------------
AmelOr58        ------------------------------------------------------------
AmelOr59        ------------------------------------------------------------
AmelOr60        ------------------------------------------------------------
AmelOr61        ------------------------------------------------------------
AmelOr63        ------------------------------------------------------------
AmelOr64        ------------------------------------------------------------
AmelOr66        ------------------------------------------------------------
AmelOr65        ------------------------------------------------------------
AmelOr67        ------------------------------------------------------------
AmelOr114       KR---------------------------------------------------------- 375
AmelOr115       VEGK-------------------------------------------------------- 400
AmelOr118       ------------------------------------------------------------
AmelOr164       ------------------------------------------------------------
```

```
AmelOr163       ------------------------------------------------------------
AmelOr165       ------------------------------------------------------------
AmelOr166       ------------------------------------------------------------
AmelOr167       ------------------------------------------------------------
DmelOr35a       MSN--------------------------------------------------------- 409
DmelOr74a       ------------------------------------------------------------
AgamOr3         ------------------------------------------------------------
AgamOr5         ------------------------------------------------------------
AgamOr13        ------------------------------------------------------------
AgamOr15        ------------------------------------------------------------
AgamOr17        ------------------------------------------------------------
AgamOr16        ------------------------------------------------------------
AgamOr55        ------------------------------------------------------------
AgamOr18        ------------------------------------------------------------
AgamOr14        ------------------------------------------------------------
AgamOr51        ------------------------------------------------------------
AgamOr12        ------------------------------------------------------------
AgamOr19        ------------------------------------------------------------
AgamOr20        ------------------------------------------------------------
AgamOr21        ------------------------------------------------------------
AgamOr50        ------------------------------------------------------------
AgamOr29        ------------------------------------------------------------
AgamOr53        ------------------------------------------------------------
AgamOr30        ------------------------------------------------------------
AgamOr46        ------------------------------------------------------------
AgamOr47        ------------------------------------------------------------
AgamOr48        ------------------------------------------------------------
AgamOr49        ------------------------------------------------------------
AgamOr61        ------------------------------------------------------------
AgamOr62        ------------------------------------------------------------
AgamOr63        ------------------------------------------------------------
AgamOr60        IAKLTSDGNN-------------------------------------------------- 425
AgamOr75        ------------------------------------------------------------
AgamOr76        ------------------------------------------------------------
AgamOr78        ------------------------------------------------------------
AgamOr77        ------------------------------------------------------------
AgamOr79        ------------------------------------------------------------
AmelOr99        ------------------------------------------------------------
AmelOr101       ------------------------------------------------------------
AmelOr102       ------------------------------------------------------------
AmelOr103       ------------------------------------------------------------
AmelOr104       ------------------------------------------------------------
AmelOr98        ------------------------------------------------------------
AmelOr100       ------------------------------------------------------------
AmelOr97        ------------------------------------------------------------
AmelOr105       ------------------------------------------------------------
AmelOr109       ------------------------------------------------------------
AmelOr110       ------------------------------------------------------------
AmelOr111       ------------------------------------------------------------
AmelOr108       ------------------------------------------------------------
AmelOr107       ------------------------------------------------------------
AmelOr112       ------------------------------------------------------------
AmelOr113       ------------------------------------------------------------
AmelOr106       ------------------------------------------------------------
AmelOr122       ------------------------------------------------------------
AmelOr125       ------------------------------------------------------------
AmelOr123       ------------------------------------------------------------
AmelOr126       ------------------------------------------------------------
AmelOr127       ------------------------------------------------------------
AmelOr128       ------------------------------------------------------------
AmelOr129       ------------------------------------------------------------
AmelOr133       ------------------------------------------------------------
AmelOr134       ------------------------------------------------------------
AmelOr131       ------------------------------------------------------------
AmelOr132       ------------------------------------------------------------
AmelOr135       ------------------------------------------------------------
AmelOr136       ------------------------------------------------------------
AmelOr138       ------------------------------------------------------------
AmelOr130       ------------------------------------------------------------
AmelOr137       ------------------------------------------------------------
```

```
AmelOr139       ------------------------------------------------------------
BmorOr20        ------------------------------------------------------------
BmorOr3         ------------------------------------------------------------
BmorOr18        ------------------------------------------------------------
HvirOr6         ------------------------------------------------------------
BmorOr23        ------------------------------------------------------------
BmorOr16        ------------------------------------------------------------
BmorOr4         A----------------------------------------------------------- 418
BmorOr5         A----------------------------------------------------------- 419
BmorOr22        ------------------------------------------------------------
BmorOr17        ------------------------------------------------------------
BmorOr1         ------------------------------------------------------------
BmorOr1a        ------------------------------------------------------------
BmorOr1b        ------------------------------------------------------------
BmorOr19        ------------------------------------------------------------
BmorOr15        ------------------------------------------------------------
TcasOr5         ------------------------------------------------------------
TcasOr7         ------------------------------------------------------------
HvirOr4         N----------------------------------------------------------- 397
BmorOr13        MQ---------------------------------------------------------- 445
DmelOr10a       ------------------------------------------------------------
DmelOr33c       ------------------------------------------------------------
DmelOr7a        RFNRTN------------------------------------------------------ 413
DmelOr22c       ------------------------------------------------------------
AgamOr1         ------------------------------------------------------------
AgamOr4         VQ---------------------------------------------------------- 396
DmelOr47b       ------------------------------------------------------------
AmelOr73        ------------------------------------------------------------
AgamOr68        ------------------------------------------------------------
DmelOr59a       ------------------------------------------------------------
DmelOr23a       S----------------------------------------------------------- 379
AgamOr31        ------------------------------------------------------------
DmelOr2a        ------------------------------------------------------------
AmelOr62        ------------------------------------------------------------
AmelOr121       ------------------------------------------------------------
AmelOr140       ------------------------------------------------------------
TcasOr12        ------------------------------------------------------------
AgamOr8         EKKMK------------------------------------------------------- 399
BmorOr9         GTKFQDDL---------------------------------------------------- 388
AgamOr40        ------------------------------------------------------------
TcasOr63        ------------------------------------------------------------
TcasOr4         ------------------------------------------------------------
TcasOr13        ------------------------------------------------------------
AgamOr24        ------------------------------------------------------------
AgamOr54        ------------------------------------------------------------
AgamOr23        ------------------------------------------------------------
AgamOr64        ------------------------------------------------------------
DmelOr82a       ------------------------------------------------------------
AmelOr116       ------------------------------------------------------------
AmelOr160       ------------------------------------------------------------
TcasOr10        ------------------------------------------------------------
TcasOr26        ------------------------------------------------------------
AmelOr117       ------------------------------------------------------------
AmelOr120       ------------------------------------------------------------
BmorOr7         ------------------------------------------------------------
AgamOr6         ------------------------------------------------------------
DmelOr45a       ------------------------------------------------------------
DmelOr67d       ------------------------------------------------------------
DmelOr83c       ------------------------------------------------------------
AmelOr162       ------------------------------------------------------------
HvirOr3         ------------------------------------------------------------
TcasOr20        ------------------------------------------------------------
AgamOr11        ------------------------------------------------------------
DmelOr56a       ------------------------------------------------------------
AgamOr28        ------------------------------------------------------------
AgamOr58        PRDQ-------------------------------------------------------- 403
AgamOr59        ------------------------------------------------------------
DmelOr1a        ETH--------------------------------------------------------- 392
DmelOr13a       EKKLD------------------------------------------------------- 418
AmelOr161       ------------------------------------------------------------
```

```
AmelOr141       ------------------------------------------------------------
AgamOr52        R----------------------------------------------------------- 387
AgamOr36        ------------------------------------------------------------
AgamOr25        ------------------------------------------------------------
AgamOr45        ------------------------------------------------------------
TcasOr3         H----------------------------------------------------------- 413
DmelOr88a       ------------------------------------------------------------
AgamOr22        ------------------------------------------------------------
DmelOr49a       ------------------------------------------------------------
DmelOr85f       ------------------------------------------------------------
AmelOr119       H----------------------------------------------------------- 417
DmelOr63a       ------------------------------------------------------------
DmelOr67b       ------------------------------------------------------------
BmorOr21        ------------------------------------------------------------
BmorOr11        ------------------------------------------------------------
BmorOr12        ------------------------------------------------------------
AgamOr33        ------------------------------------------------------------
HvirOr1         ------------------------------------------------------------
HvirOr5         NDALNATKI--------------------------------------------------- 341
TcasOr1         ------------------------------------------------------------
TcasOr2         ------------------------------------------------------------


TcasOr16        ------------------------------------------------------------
AmelOr2         ------------------------------------------------------------
AgamOr7         ------------------------------------------------------------
AaegOr7         ------------------------------------------------------------
DmelOr83b       ------------------------------------------------------------
BmorOr2         ------------------------------------------------------------
BmorOr2a        ------------------------------------------------------------
HvirOr2         ------------------------------------------------------------
AgamOr56        ------------------------------------------------------------
AgamOr57        ------------------------------------------------------------
AgamOr26        ------------------------------------------------------------
AgamOr27        ------------------------------------------------------------
HvirOr9         ------------------------------------------------------------
HvirOr7         ------------------------------------------------------------
DmelOr85b       ------------------------------------------------------------
DmelOr85c       ------------------------------------------------------------
DmelOr85d       ------------------------------------------------------------
DmelOr67a       ------------------------------------------------------------
DmelOr67c       ------------------------------------------------------------
DmelOr92a       ------------------------------------------------------------
DmelOr69a       ------------------------------------------------------------
DmelOr69b       ------------------------------------------------------------
TcasOr6         IGHVLTELKMVKVAFGDSPIETNWSEEKRFKFAVRHHRFVLE------------------ 475
TcasOr11        ------------------------------------------------------------
AgamOr41        ------------------------------------------------------------
AgamOr42        ------------------------------------------------------------
AgamOr43        ------------------------------------------------------------
AgamOr44        ------------------------------------------------------------
AgamOr66        ------------------------------------------------------------
AgamOr67        ------------------------------------------------------------
AgamOr72        ------------------------------------------------------------
AgamOr73        ------------------------------------------------------------
AgamOr71        ------------------------------------------------------------
AgamOr74        ------------------------------------------------------------
AgamOr70        ------------------------------------------------------------
AgamOr69        ------------------------------------------------------------
TcasOr9         ------------------------------------------------------------
TcasOr18        ------------------------------------------------------------
DmelOr83a       ------------------------------------------------------------
DmelOr85e       ------------------------------------------------------------
TcasOr24        ------------------------------------------------------------
TcasOr25        ICGLIYNLCSFEEFGKPPQFDELNQRLDFYSKLASGYTIIGMAVYIGMKIYKIPDCKAER 504
TcasOr19        ------------------------------------------------------------
TcasOr22        ------------------------------------------------------------
TcasOr23        ------------------------------------------------------------
AmelOr168       ------------------------------------------------------------
AmelOr170       ------------------------------------------------------------
```

```
AmelOr169       ------------------------------------------------------------
DmelOr65b       ------------------------------------------------------------
DmelOr65c       ------------------------------------------------------------
DmelOr65a       ------------------------------------------------------------
TcasOr17        ------------------------------------------------------------
TcasOr21        ------------------------------------------------------------
TcasOr56        ------------------------------------------------------------
TcasOr15        ------------------------------------------------------------
AgamOr9         ------------------------------------------------------------
AgamOr65        ------------------------------------------------------------
BmorOr6         ------------------------------------------------------------
BmorOr10        ------------------------------------------------------------
AgamOr34        ------------------------------------------------------------
AgamOr37        ------------------------------------------------------------
DmelOr94a       ------------------------------------------------------------
DmelOr94b       ------------------------------------------------------------
DmelOr71a       ------------------------------------------------------------
DmelOr46a       ------------------------------------------------------------
DmelOr46b       ------------------------------------------------------------
HvirOr8         ------------------------------------------------------------
BmorOr8         ------------------------------------------------------------
AmelOr68        ------------------------------------------------------------
AmelOr69        ------------------------------------------------------------
AmelOr70        ------------------------------------------------------------
AmelOr71        ------------------------------------------------------------
AmelOr72        ------------------------------------------------------------
DmelOr19a       ------------------------------------------------------------
DmelOr19b       ------------------------------------------------------------
DmelOr33a       ------------------------------------------------------------
DmelOr33b       ------------------------------------------------------------
DmelOr22a       ------------------------------------------------------------
DmelOr22b       ------------------------------------------------------------
DmelOr42b       ------------------------------------------------------------
DmelOr59b       ------------------------------------------------------------
DmelOr59c       ------------------------------------------------------------
DmelOr98a       ------------------------------------------------------------
DmelOr42a       ------------------------------------------------------------
DmelOr85a       ------------------------------------------------------------
DmelOr43b       ------------------------------------------------------------
AgamOr2         ------------------------------------------------------------
AgamOr10        ------------------------------------------------------------
DmelOr30a       ------------------------------------------------------------
DmelOr49b       ------------------------------------------------------------
DmelOr43a       ------------------------------------------------------------
AgamOr32        ------------------------------------------------------------
AgamOr35        ------------------------------------------------------------
DmelOr47a       ------------------------------------------------------------
DmelOr98b       ------------------------------------------------------------
DmelOr9a        ------------------------------------------------------------
AgamOr38        ------------------------------------------------------------
AgamOr39        ------------------------------------------------------------
DmelOr24a       ------------------------------------------------------------
DmelOr45b       ------------------------------------------------------------
AmelOr74        ------------------------------------------------------------
AmelOr86        ------------------------------------------------------------
AmelOr75        ------------------------------------------------------------
AmelOr84        ------------------------------------------------------------
AmelOr87        ------------------------------------------------------------
AmelOr76        ------------------------------------------------------------
AmelOr85        ------------------------------------------------------------
AmelOr88        ------------------------------------------------------------
AmelOr91        ------------------------------------------------------------
AmelOr92        ------------------------------------------------------------
AmelOr95        ------------------------------------------------------------
AmelOr93        ------------------------------------------------------------
AmelOr77        ------------------------------------------------------------
AmelOr94        ------------------------------------------------------------
AmelOr96        ------------------------------------------------------------
AmelOr79        ------------------------------------------------------------
AmelOr83        ------------------------------------------------------------
```

```
AmelOr80        ------------------------------------------------------------
AmelOr81        ------------------------------------------------------------
AmelOr82        ------------------------------------------------------------
AmelOr78        ------------------------------------------------------------
AmelOr89        ------------------------------------------------------------
AmelOr90        ------------------------------------------------------------
AmelOr142       ------------------------------------------------------------
AmelOr146       ------------------------------------------------------------
AmelOr148       ------------------------------------------------------------
AmelOr158       ------------------------------------------------------------
AmelOr147       ------------------------------------------------------------
AmelOr150       ------------------------------------------------------------
AmelOr151       ------------------------------------------------------------
AmelOr152       ------------------------------------------------------------
AmelOr154       ------------------------------------------------------------
AmelOr155       ------------------------------------------------------------
AmelOr157       ------------------------------------------------------------
AmelOr156       ------------------------------------------------------------
AmelOr153       ------------------------------------------------------------
AmelOr144       ------------------------------------------------------------
AmelOr145       ------------------------------------------------------------
AmelOr143       ------------------------------------------------------------
AmelOr1         ------------------------------------------------------------
AmelOr3         ------------------------------------------------------------
AmelOr6         ------------------------------------------------------------
AmelOr7         ------------------------------------------------------------
AmelOr4         ------------------------------------------------------------
AmelOr5         ------------------------------------------------------------
AmelOr8         ------------------------------------------------------------
AmelOr9         ------------------------------------------------------------
AmelOr11        ------------------------------------------------------------
AmelOr12        ------------------------------------------------------------
AmelOr10        ------------------------------------------------------------
AmelOr14        ------------------------------------------------------------
AmelOr15        ------------------------------------------------------------
AmelOr13        ------------------------------------------------------------
AmelOr16        ------------------------------------------------------------
AmelOr17        ------------------------------------------------------------
AmelOr18        ------------------------------------------------------------
AmelOr19        ------------------------------------------------------------
AmelOr22        ------------------------------------------------------------
AmelOr24        ------------------------------------------------------------
AmelOr20        ------------------------------------------------------------
AmelOr25        ------------------------------------------------------------
AmelOr29        ------------------------------------------------------------
AmelOr30        ------------------------------------------------------------
AmelOr21        ------------------------------------------------------------
AmelOr23        ------------------------------------------------------------
AmelOr35        ------------------------------------------------------------
AmelOr27        ------------------------------------------------------------
AmelOr28        ------------------------------------------------------------
AmelOr33        ------------------------------------------------------------
AmelOr34        ------------------------------------------------------------
AmelOr32        ------------------------------------------------------------
AmelOr31        ------------------------------------------------------------
AmelOr36        ------------------------------------------------------------
AmelOr37        ------------------------------------------------------------
AmelOr38        ------------------------------------------------------------
AmelOr39        ------------------------------------------------------------
AmelOr47        ------------------------------------------------------------
AmelOr48        ------------------------------------------------------------
AmelOr40        ------------------------------------------------------------
AmelOr43        ------------------------------------------------------------
AmelOr45        ------------------------------------------------------------
AmelOr41        ------------------------------------------------------------
AmelOr42        ------------------------------------------------------------
AmelOr44        ------------------------------------------------------------
AmelOr46        ------------------------------------------------------------
AmelOr49        ------------------------------------------------------------
AmelOr50        ------------------------------------------------------------
```

```
AmelOr26        ------------------------------------------------------------
AmelOr53        ------------------------------------------------------------
AmelOr54        ------------------------------------------------------------
AmelOr52        ------------------------------------------------------------
AmelOr51        ------------------------------------------------------------
AmelOr55        ------------------------------------------------------------
AmelOr57        ------------------------------------------------------------
AmelOr56        ------------------------------------------------------------
AmelOr58        ------------------------------------------------------------
AmelOr59        ------------------------------------------------------------
AmelOr60        ------------------------------------------------------------
AmelOr61        ------------------------------------------------------------
AmelOr63        ------------------------------------------------------------
AmelOr64        ------------------------------------------------------------
AmelOr66        ------------------------------------------------------------
AmelOr65        ------------------------------------------------------------
AmelOr67        ------------------------------------------------------------
AmelOr114       ------------------------------------------------------------
AmelOr115       ------------------------------------------------------------
AmelOr118       ------------------------------------------------------------
AmelOr164       ------------------------------------------------------------
AmelOr163       ------------------------------------------------------------
AmelOr165       ------------------------------------------------------------
AmelOr166       ------------------------------------------------------------
AmelOr167       ------------------------------------------------------------
DmelOr35a       ------------------------------------------------------------
DmelOr74a       ------------------------------------------------------------
AgamOr3         ------------------------------------------------------------
AgamOr5         ------------------------------------------------------------
AgamOr13        ------------------------------------------------------------
AgamOr15        ------------------------------------------------------------
AgamOr17        ------------------------------------------------------------
AgamOr16        ------------------------------------------------------------
AgamOr55        ------------------------------------------------------------
AgamOr18        ------------------------------------------------------------
AgamOr14        ------------------------------------------------------------
AgamOr51        ------------------------------------------------------------
AgamOr12        ------------------------------------------------------------
AgamOr19        ------------------------------------------------------------
AgamOr20        ------------------------------------------------------------
AgamOr21        ------------------------------------------------------------
AgamOr50        ------------------------------------------------------------
AgamOr29        ------------------------------------------------------------
AgamOr53        ------------------------------------------------------------
AgamOr30        ------------------------------------------------------------
AgamOr46        ------------------------------------------------------------
AgamOr47        ------------------------------------------------------------
AgamOr48        ------------------------------------------------------------
AgamOr49        ------------------------------------------------------------
AgamOr61        ------------------------------------------------------------
AgamOr62        ------------------------------------------------------------
AgamOr63        ------------------------------------------------------------
AgamOr60        ------------------------------------------------------------
AgamOr75        ------------------------------------------------------------
AgamOr76        ------------------------------------------------------------
AgamOr78        ------------------------------------------------------------
AgamOr77        ------------------------------------------------------------
AgamOr79        ------------------------------------------------------------
AmelOr99        ------------------------------------------------------------
AmelOr101       ------------------------------------------------------------
AmelOr102       ------------------------------------------------------------
AmelOr103       ------------------------------------------------------------
AmelOr104       ------------------------------------------------------------
AmelOr98        ------------------------------------------------------------
AmelOr100       ------------------------------------------------------------
AmelOr97        ------------------------------------------------------------
AmelOr105       ------------------------------------------------------------
AmelOr109       ------------------------------------------------------------
AmelOr110       ------------------------------------------------------------
AmelOr111       ------------------------------------------------------------
```

```
AmelOr108       ------------------------------------------------------------
AmelOr107       ------------------------------------------------------------
AmelOr112       ------------------------------------------------------------
AmelOr113       ------------------------------------------------------------
AmelOr106       ------------------------------------------------------------
AmelOr122       ------------------------------------------------------------
AmelOr125       ------------------------------------------------------------
AmelOr123       ------------------------------------------------------------
AmelOr126       ------------------------------------------------------------
AmelOr127       ------------------------------------------------------------
AmelOr128       ------------------------------------------------------------
AmelOr129       ------------------------------------------------------------
AmelOr133       ------------------------------------------------------------
AmelOr134       ------------------------------------------------------------
AmelOr131       ------------------------------------------------------------
AmelOr132       ------------------------------------------------------------
AmelOr135       ------------------------------------------------------------
AmelOr136       ------------------------------------------------------------
AmelOr138       ------------------------------------------------------------
AmelOr130       ------------------------------------------------------------
AmelOr137       ------------------------------------------------------------
AmelOr139       ------------------------------------------------------------
BmorOr20        ------------------------------------------------------------
BmorOr3         ------------------------------------------------------------
BmorOr18        ------------------------------------------------------------
HvirOr6         ------------------------------------------------------------
BmorOr23        ------------------------------------------------------------
BmorOr16        ------------------------------------------------------------
BmorOr4         ------------------------------------------------------------
BmorOr5         ------------------------------------------------------------
BmorOr22        ------------------------------------------------------------
BmorOr17        ------------------------------------------------------------
BmorOr1         ------------------------------------------------------------
BmorOr1a        ------------------------------------------------------------
BmorOr1b        ------------------------------------------------------------
BmorOr19        ------------------------------------------------------------
BmorOr15        ------------------------------------------------------------
TcasOr5         ------------------------------------------------------------
TcasOr7         ------------------------------------------------------------
HvirOr4         ------------------------------------------------------------
BmorOr13        ------------------------------------------------------------
DmelOr10a       ------------------------------------------------------------
DmelOr33c       ------------------------------------------------------------
DmelOr7a        ------------------------------------------------------------
DmelOr22c       ------------------------------------------------------------
AgamOr1         ------------------------------------------------------------
AgamOr4         ------------------------------------------------------------
DmelOr47b       ------------------------------------------------------------
AmelOr73        ------------------------------------------------------------
AgamOr68        ------------------------------------------------------------
DmelOr59a       ------------------------------------------------------------
DmelOr23a       ------------------------------------------------------------
AgamOr31        ------------------------------------------------------------
DmelOr2a        ------------------------------------------------------------
AmelOr62        ------------------------------------------------------------
AmelOr121       ------------------------------------------------------------
AmelOr140       ------------------------------------------------------------
TcasOr12        ------------------------------------------------------------
AgamOr8         ------------------------------------------------------------
BmorOr9         ------------------------------------------------------------
AgamOr40        ------------------------------------------------------------
TcasOr63        ------------------------------------------------------------
TcasOr4         ------------------------------------------------------------
TcasOr13        ------------------------------------------------------------
AgamOr24        ------------------------------------------------------------
AgamOr54        ------------------------------------------------------------
AgamOr23        ------------------------------------------------------------
AgamOr64        ------------------------------------------------------------
DmelOr82a       ------------------------------------------------------------
AmelOr116       ------------------------------------------------------------
```

```
AmelOr160      ------------------------------------------------------------
TcasOr10       ------------------------------------------------------------
TcasOr26       ------------------------------------------------------------
AmelOr117      ------------------------------------------------------------
AmelOr120      ------------------------------------------------------------
BmorOr7        ------------------------------------------------------------
AgamOr6        ------------------------------------------------------------
DmelOr45a      ------------------------------------------------------------
DmelOr67d      ------------------------------------------------------------
DmelOr83c      ------------------------------------------------------------
AmelOr162      ------------------------------------------------------------
HvirOr3        ------------------------------------------------------------
TcasOr20       ------------------------------------------------------------
AgamOr11       ------------------------------------------------------------
DmelOr56a      ------------------------------------------------------------
AgamOr28       ------------------------------------------------------------
AgamOr58       ------------------------------------------------------------
AgamOr59       ------------------------------------------------------------
DmelOr1a       ------------------------------------------------------------
DmelOr13a      ------------------------------------------------------------
AmelOr161      ------------------------------------------------------------
AmelOr141      ------------------------------------------------------------
AgamOr52       ------------------------------------------------------------
AgamOr36       ------------------------------------------------------------
AgamOr25       ------------------------------------------------------------
AgamOr45       ------------------------------------------------------------
TcasOr3        ------------------------------------------------------------
DmelOr88a      ------------------------------------------------------------
AgamOr22       ------------------------------------------------------------
DmelOr49a      ------------------------------------------------------------
DmelOr85f      ------------------------------------------------------------
AmelOr119      ------------------------------------------------------------
DmelOr63a      ------------------------------------------------------------
DmelOr67b      ------------------------------------------------------------
BmorOr21       ------------------------------------------------------------
BmorOr11       ------------------------------------------------------------
BmorOr12       ------------------------------------------------------------
AgamOr33       ------------------------------------------------------------
HvirOr1        ------------------------------------------------------------
HvirOr5        ------------------------------------------------------------
TcasOr1        ------------------------------------------------------------
TcasOr2        ------------------------------------------------------------


TcasOr16       ------------------------------------------------------------
AmelOr2        ------------------------------------------------------------
AgamOr7        ------------------------------------------------------------
AaegOr7        ------------------------------------------------------------
DmelOr83b      ------------------------------------------------------------
BmorOr2        ------------------------------------------------------------
BmorOr2a       ------------------------------------------------------------
HvirOr2        ------------------------------------------------------------
AgamOr56       ------------------------------------------------------------
AgamOr57       ------------------------------------------------------------
AgamOr26       ------------------------------------------------------------
AgamOr27       ------------------------------------------------------------
HvirOr9        ------------------------------------------------------------
HvirOr7        ------------------------------------------------------------
DmelOr85b      ------------------------------------------------------------
DmelOr85c      ------------------------------------------------------------
DmelOr85d      ------------------------------------------------------------
DmelOr67a      ------------------------------------------------------------
DmelOr67c      ------------------------------------------------------------
DmelOr92a      ------------------------------------------------------------
DmelOr69a      ------------------------------------------------------------
DmelOr69b      ------------------------------------------------------------
TcasOr6        ------------------------------------------------------------
TcasOr11       ------------------------------------------------------------
AgamOr41       ------------------------------------------------------------
AgamOr42       ------------------------------------------------------------
```

```
AgamOr43        ------------------------------------------------------------
AgamOr44        ------------------------------------------------------------
AgamOr66        ------------------------------------------------------------
AgamOr67        ------------------------------------------------------------
AgamOr72        ------------------------------------------------------------
AgamOr73        ------------------------------------------------------------
AgamOr71        ------------------------------------------------------------
AgamOr74        ------------------------------------------------------------
AgamOr70        ------------------------------------------------------------
AgamOr69        ------------------------------------------------------------
TcasOr9         ------------------------------------------------------------
TcasOr18        ------------------------------------------------------------
DmelOr83a       ------------------------------------------------------------
DmelOr85e       ------------------------------------------------------------
TcasOr24        ------------------------------------------------------------
TcasOr25        GAIQVCGLTVPLLCEQFNEAYYWTAFSHIVCSVLACACVAHQAVNQDVLAVLHYIGWIMS 564
TcasOr19        ------------------------------------------------------------
TcasOr22        ------------------------------------------------------------
TcasOr23        ------------------------------------------------------------
AmelOr168       ------------------------------------------------------------
AmelOr170       ------------------------------------------------------------
AmelOr169       ------------------------------------------------------------
DmelOr65b       ------------------------------------------------------------
DmelOr65c       ------------------------------------------------------------
DmelOr65a       ------------------------------------------------------------
TcasOr17        ------------------------------------------------------------
TcasOr21        ------------------------------------------------------------
TcasOr56        ------------------------------------------------------------
TcasOr15        ------------------------------------------------------------
AgamOr9         ------------------------------------------------------------
AgamOr65        ------------------------------------------------------------
BmorOr6         ------------------------------------------------------------
BmorOr10        ------------------------------------------------------------
AgamOr34        ------------------------------------------------------------
AgamOr37        ------------------------------------------------------------
DmelOr94a       ------------------------------------------------------------
DmelOr94b       ------------------------------------------------------------
DmelOr71a       ------------------------------------------------------------
DmelOr46a       ------------------------------------------------------------
DmelOr46b       ------------------------------------------------------------
HvirOr8         ------------------------------------------------------------
BmorOr8         ------------------------------------------------------------
AmelOr68        ------------------------------------------------------------
AmelOr69        ------------------------------------------------------------
AmelOr70        ------------------------------------------------------------
AmelOr71        ------------------------------------------------------------
AmelOr72        ------------------------------------------------------------
DmelOr19a       ------------------------------------------------------------
DmelOr19b       ------------------------------------------------------------
DmelOr33a       ------------------------------------------------------------
DmelOr33b       ------------------------------------------------------------
DmelOr22a       ------------------------------------------------------------
DmelOr22b       ------------------------------------------------------------
DmelOr42b       ------------------------------------------------------------
DmelOr59b       ------------------------------------------------------------
DmelOr59c       ------------------------------------------------------------
DmelOr98a       ------------------------------------------------------------
DmelOr42a       ------------------------------------------------------------
DmelOr85a       ------------------------------------------------------------
DmelOr43b       ------------------------------------------------------------
AgamOr2         ------------------------------------------------------------
AgamOr10        ------------------------------------------------------------
DmelOr30a       ------------------------------------------------------------
DmelOr49b       ------------------------------------------------------------
DmelOr43a       ------------------------------------------------------------
AgamOr32        ------------------------------------------------------------
AgamOr35        ------------------------------------------------------------
DmelOr47a       ------------------------------------------------------------
DmelOr98b       ------------------------------------------------------------
DmelOr9a        ------------------------------------------------------------
```

```
AgamOr38        ------------------------------------------------------------
AgamOr39        ------------------------------------------------------------
DmelOr24a       ------------------------------------------------------------
DmelOr45b       ------------------------------------------------------------
AmelOr74        ------------------------------------------------------------
AmelOr86        ------------------------------------------------------------
AmelOr75        ------------------------------------------------------------
AmelOr84        ------------------------------------------------------------
AmelOr87        ------------------------------------------------------------
AmelOr76        ------------------------------------------------------------
AmelOr85        ------------------------------------------------------------
AmelOr88        ------------------------------------------------------------
AmelOr91        ------------------------------------------------------------
AmelOr92        ------------------------------------------------------------
AmelOr95        ------------------------------------------------------------
AmelOr93        ------------------------------------------------------------
AmelOr77        ------------------------------------------------------------
AmelOr94        ------------------------------------------------------------
AmelOr96        ------------------------------------------------------------
AmelOr79        ------------------------------------------------------------
AmelOr83        ------------------------------------------------------------
AmelOr80        ------------------------------------------------------------
AmelOr81        ------------------------------------------------------------
AmelOr82        ------------------------------------------------------------
AmelOr78        ------------------------------------------------------------
AmelOr89        ------------------------------------------------------------
AmelOr90        ------------------------------------------------------------
AmelOr142       ------------------------------------------------------------
AmelOr146       ------------------------------------------------------------
AmelOr148       ------------------------------------------------------------
AmelOr158       ------------------------------------------------------------
AmelOr147       ------------------------------------------------------------
AmelOr150       ------------------------------------------------------------
AmelOr151       ------------------------------------------------------------
AmelOr152       ------------------------------------------------------------
AmelOr154       ------------------------------------------------------------
AmelOr155       ------------------------------------------------------------
AmelOr157       ------------------------------------------------------------
AmelOr156       ------------------------------------------------------------
AmelOr153       ------------------------------------------------------------
AmelOr144       ------------------------------------------------------------
AmelOr145       ------------------------------------------------------------
AmelOr143       ------------------------------------------------------------
AmelOr1         ------------------------------------------------------------
AmelOr3         ------------------------------------------------------------
AmelOr6         ------------------------------------------------------------
AmelOr7         ------------------------------------------------------------
AmelOr4         ------------------------------------------------------------
AmelOr5         ------------------------------------------------------------
AmelOr8         ------------------------------------------------------------
AmelOr9         ------------------------------------------------------------
AmelOr11        ------------------------------------------------------------
AmelOr12        ------------------------------------------------------------
AmelOr10        ------------------------------------------------------------
AmelOr14        ------------------------------------------------------------
AmelOr15        ------------------------------------------------------------
AmelOr13        ------------------------------------------------------------
AmelOr16        ------------------------------------------------------------
AmelOr17        ------------------------------------------------------------
AmelOr18        ------------------------------------------------------------
AmelOr19        ------------------------------------------------------------
AmelOr22        ------------------------------------------------------------
AmelOr24        ------------------------------------------------------------
AmelOr20        ------------------------------------------------------------
AmelOr25        ------------------------------------------------------------
AmelOr29        ------------------------------------------------------------
AmelOr30        ------------------------------------------------------------
AmelOr21        ------------------------------------------------------------
AmelOr23        ------------------------------------------------------------
AmelOr35        ------------------------------------------------------------
```

```
AmelOr27        ------------------------------------------------------------
AmelOr28        ------------------------------------------------------------
AmelOr33        ------------------------------------------------------------
AmelOr34        ------------------------------------------------------------
AmelOr32        ------------------------------------------------------------
AmelOr31        ------------------------------------------------------------
AmelOr36        ------------------------------------------------------------
AmelOr37        ------------------------------------------------------------
AmelOr38        ------------------------------------------------------------
AmelOr39        ------------------------------------------------------------
AmelOr47        ------------------------------------------------------------
AmelOr48        ------------------------------------------------------------
AmelOr40        ------------------------------------------------------------
AmelOr43        ------------------------------------------------------------
AmelOr45        ------------------------------------------------------------
AmelOr41        ------------------------------------------------------------
AmelOr42        ------------------------------------------------------------
AmelOr44        ------------------------------------------------------------
AmelOr46        ------------------------------------------------------------
AmelOr49        ------------------------------------------------------------
AmelOr50        ------------------------------------------------------------
AmelOr26        ------------------------------------------------------------
AmelOr53        ------------------------------------------------------------
AmelOr54        ------------------------------------------------------------
AmelOr52        ------------------------------------------------------------
AmelOr51        ------------------------------------------------------------
AmelOr55        ------------------------------------------------------------
AmelOr57        ------------------------------------------------------------
AmelOr56        ------------------------------------------------------------
AmelOr58        ------------------------------------------------------------
AmelOr59        ------------------------------------------------------------
AmelOr60        ------------------------------------------------------------
AmelOr61        ------------------------------------------------------------
AmelOr63        ------------------------------------------------------------
AmelOr64        ------------------------------------------------------------
AmelOr66        ------------------------------------------------------------
AmelOr65        ------------------------------------------------------------
AmelOr67        ------------------------------------------------------------
AmelOr114       ------------------------------------------------------------
AmelOr115       ------------------------------------------------------------
AmelOr118       ------------------------------------------------------------
AmelOr164       ------------------------------------------------------------
AmelOr163       ------------------------------------------------------------
AmelOr165       ------------------------------------------------------------
AmelOr166       ------------------------------------------------------------
AmelOr167       ------------------------------------------------------------
DmelOr35a       ------------------------------------------------------------
DmelOr74a       ------------------------------------------------------------
AgamOr3         ------------------------------------------------------------
AgamOr5         ------------------------------------------------------------
AgamOr13        ------------------------------------------------------------
AgamOr15        ------------------------------------------------------------
AgamOr17        ------------------------------------------------------------
AgamOr16        ------------------------------------------------------------
AgamOr55        ------------------------------------------------------------
AgamOr18        ------------------------------------------------------------
AgamOr14        ------------------------------------------------------------
AgamOr51        ------------------------------------------------------------
AgamOr12        ------------------------------------------------------------
AgamOr19        ------------------------------------------------------------
AgamOr20        ------------------------------------------------------------
AgamOr21        ------------------------------------------------------------
AgamOr50        ------------------------------------------------------------
AgamOr29        ------------------------------------------------------------
AgamOr53        ------------------------------------------------------------
AgamOr30        ------------------------------------------------------------
AgamOr46        ------------------------------------------------------------
AgamOr47        ------------------------------------------------------------
AgamOr48        ------------------------------------------------------------
AgamOr49        ------------------------------------------------------------
```

```
AgamOr61        ------------------------------------------------------------
AgamOr62        ------------------------------------------------------------
AgamOr63        ------------------------------------------------------------
AgamOr60        ------------------------------------------------------------
AgamOr75        ------------------------------------------------------------
AgamOr76        ------------------------------------------------------------
AgamOr78        ------------------------------------------------------------
AgamOr77        ------------------------------------------------------------
AgamOr79        ------------------------------------------------------------
AmelOr99        ------------------------------------------------------------
AmelOr101       ------------------------------------------------------------
AmelOr102       ------------------------------------------------------------
AmelOr103       ------------------------------------------------------------
AmelOr104       ------------------------------------------------------------
AmelOr98        ------------------------------------------------------------
AmelOr100       ------------------------------------------------------------
AmelOr97        ------------------------------------------------------------
AmelOr105       ------------------------------------------------------------
AmelOr109       ------------------------------------------------------------
AmelOr110       ------------------------------------------------------------
AmelOr111       ------------------------------------------------------------
AmelOr108       ------------------------------------------------------------
AmelOr107       ------------------------------------------------------------
AmelOr112       ------------------------------------------------------------
AmelOr113       ------------------------------------------------------------
AmelOr106       ------------------------------------------------------------
AmelOr122       ------------------------------------------------------------
AmelOr125       ------------------------------------------------------------
AmelOr123       ------------------------------------------------------------
AmelOr126       ------------------------------------------------------------
AmelOr127       ------------------------------------------------------------
AmelOr128       ------------------------------------------------------------
AmelOr129       ------------------------------------------------------------
AmelOr133       ------------------------------------------------------------
AmelOr134       ------------------------------------------------------------
AmelOr131       ------------------------------------------------------------
AmelOr132       ------------------------------------------------------------
AmelOr135       ------------------------------------------------------------
AmelOr136       ------------------------------------------------------------
AmelOr138       ------------------------------------------------------------
AmelOr130       ------------------------------------------------------------
AmelOr137       ------------------------------------------------------------
AmelOr139       ------------------------------------------------------------
BmorOr20        ------------------------------------------------------------
BmorOr3         ------------------------------------------------------------
BmorOr18        ------------------------------------------------------------
HvirOr6         ------------------------------------------------------------
BmorOr23        ------------------------------------------------------------
BmorOr16        ------------------------------------------------------------
BmorOr4         ------------------------------------------------------------
BmorOr5         ------------------------------------------------------------
BmorOr22        ------------------------------------------------------------
BmorOr17        ------------------------------------------------------------
BmorOr1         ------------------------------------------------------------
BmorOr1a        ------------------------------------------------------------
BmorOr1b        ------------------------------------------------------------
BmorOr19        ------------------------------------------------------------
BmorOr15        ------------------------------------------------------------
TcasOr5         ------------------------------------------------------------
TcasOr7         ------------------------------------------------------------
HvirOr4         ------------------------------------------------------------
BmorOr13        ------------------------------------------------------------
DmelOr10a       ------------------------------------------------------------
DmelOr33c       ------------------------------------------------------------
DmelOr7a        ------------------------------------------------------------
DmelOr22c       ------------------------------------------------------------
AgamOr1         ------------------------------------------------------------
AgamOr4         ------------------------------------------------------------
DmelOr47b       ------------------------------------------------------------
AmelOr73        ------------------------------------------------------------
```

```
AgamOr68        ------------------------------------------------------------
DmelOr59a       ------------------------------------------------------------
DmelOr23a       ------------------------------------------------------------
AgamOr31        ------------------------------------------------------------
DmelOr2a        ------------------------------------------------------------
AmelOr62        ------------------------------------------------------------
AmelOr121       ------------------------------------------------------------
AmelOr140       ------------------------------------------------------------
TcasOr12        ------------------------------------------------------------
AgamOr8         ------------------------------------------------------------
BmorOr9         ------------------------------------------------------------
AgamOr40        ------------------------------------------------------------
TcasOr63        ------------------------------------------------------------
TcasOr4         ------------------------------------------------------------
TcasOr13        ------------------------------------------------------------
AgamOr24        ------------------------------------------------------------
AgamOr54        ------------------------------------------------------------
AgamOr23        ------------------------------------------------------------
AgamOr64        ------------------------------------------------------------
DmelOr82a       ------------------------------------------------------------
AmelOr116       ------------------------------------------------------------
AmelOr160       ------------------------------------------------------------
TcasOr10        ------------------------------------------------------------
TcasOr26        ------------------------------------------------------------
AmelOr117       ------------------------------------------------------------
AmelOr120       ------------------------------------------------------------
BmorOr7         ------------------------------------------------------------
AgamOr6         ------------------------------------------------------------
DmelOr45a       ------------------------------------------------------------
DmelOr67d       ------------------------------------------------------------
DmelOr83c       ------------------------------------------------------------
AmelOr162       ------------------------------------------------------------
HvirOr3         ------------------------------------------------------------
TcasOr20        ------------------------------------------------------------
AgamOr11        ------------------------------------------------------------
DmelOr56a       ------------------------------------------------------------
AgamOr28        ------------------------------------------------------------
AgamOr58        ------------------------------------------------------------
AgamOr59        ------------------------------------------------------------
DmelOr1a        ------------------------------------------------------------
DmelOr13a       ------------------------------------------------------------
AmelOr161       ------------------------------------------------------------
AmelOr141       ------------------------------------------------------------
AgamOr52        ------------------------------------------------------------
AgamOr36        ------------------------------------------------------------
AgamOr25        ------------------------------------------------------------
AgamOr45        ------------------------------------------------------------
TcasOr3         ------------------------------------------------------------
DmelOr88a       ------------------------------------------------------------
AgamOr22        ------------------------------------------------------------
DmelOr49a       ------------------------------------------------------------
DmelOr85f       ------------------------------------------------------------
AmelOr119       ------------------------------------------------------------
DmelOr63a       ------------------------------------------------------------
DmelOr67b       ------------------------------------------------------------
BmorOr21        ------------------------------------------------------------
BmorOr11        ------------------------------------------------------------
BmorOr12        ------------------------------------------------------------
AgamOr33        ------------------------------------------------------------
HvirOr1         ------------------------------------------------------------
HvirOr5         ------------------------------------------------------------
TcasOr1         ------------------------------------------------------------
TcasOr2         ------------------------------------------------------------


TcasOr16        ------------------------------------------------------------
AmelOr2         ------------------------------------------------------------
AgamOr7         ------------------------------------------------------------
AaegOr7         ------------------------------------------------------------
DmelOr83b       ------------------------------------------------------------
```

```
BmorOr2         ------------------------------------------------------------
BmorOr2a        ------------------------------------------------------------
HvirOr2         ------------------------------------------------------------
AgamOr56        ------------------------------------------------------------
AgamOr57        ------------------------------------------------------------
AgamOr26        ------------------------------------------------------------
AgamOr27        ------------------------------------------------------------
HvirOr9         ------------------------------------------------------------
HvirOr7         ------------------------------------------------------------
DmelOr85b       ------------------------------------------------------------
DmelOr85c       ------------------------------------------------------------
DmelOr85d       ------------------------------------------------------------
DmelOr67a       ------------------------------------------------------------
DmelOr67c       ------------------------------------------------------------
DmelOr92a       ------------------------------------------------------------
DmelOr69a       ------------------------------------------------------------
DmelOr69b       ------------------------------------------------------------
TcasOr6         ------------------------------------------------------------
TcasOr11        ------------------------------------------------------------
AgamOr41        ------------------------------------------------------------
AgamOr42        ------------------------------------------------------------
AgamOr43        ------------------------------------------------------------
AgamOr44        ------------------------------------------------------------
AgamOr66        ------------------------------------------------------------
AgamOr67        ------------------------------------------------------------
AgamOr72        ------------------------------------------------------------
AgamOr73        ------------------------------------------------------------
AgamOr71        ------------------------------------------------------------
AgamOr74        ------------------------------------------------------------
AgamOr70        ------------------------------------------------------------
AgamOr69        ------------------------------------------------------------
TcasOr9         ------------------------------------------------------------
TcasOr18        ------------------------------------------------------------
DmelOr83a       ------------------------------------------------------------
DmelOr85e       ------------------------------------------------------------
TcasOr24        ------------------------------------------------------------
TcasOr25        IFVVCWAGQILTDSSVTVADACFGSNWLRACPRLRRDLSFMMRRSQKCVELRAGPFNVLC 624
TcasOr19        ------------------------------------------------------------
TcasOr22        ------------------------------------------------------------
TcasOr23        ------------------------------------------------------------
AmelOr168       ------------------------------------------------------------
AmelOr170       ------------------------------------------------------------
AmelOr169       ------------------------------------------------------------
DmelOr65b       ------------------------------------------------------------
DmelOr65c       ------------------------------------------------------------
DmelOr65a       ------------------------------------------------------------
TcasOr17        ------------------------------------------------------------
TcasOr21        ------------------------------------------------------------
TcasOr56        ------------------------------------------------------------
TcasOr15        ------------------------------------------------------------
AgamOr9         ------------------------------------------------------------
AgamOr65        ------------------------------------------------------------
BmorOr6         ------------------------------------------------------------
BmorOr10        ------------------------------------------------------------
AgamOr34        ------------------------------------------------------------
AgamOr37        ------------------------------------------------------------
DmelOr94a       ------------------------------------------------------------
DmelOr94b       ------------------------------------------------------------
DmelOr71a       ------------------------------------------------------------
DmelOr46a       ------------------------------------------------------------
DmelOr46b       ------------------------------------------------------------
HvirOr8         ------------------------------------------------------------
BmorOr8         ------------------------------------------------------------
AmelOr68        ------------------------------------------------------------
AmelOr69        ------------------------------------------------------------
AmelOr70        ------------------------------------------------------------
AmelOr71        ------------------------------------------------------------
AmelOr72        ------------------------------------------------------------
DmelOr19a       ------------------------------------------------------------
DmelOr19b       ------------------------------------------------------------
```

```
DmelOr33a       ------------------------------------------------------------
DmelOr33b       ------------------------------------------------------------
DmelOr22a       ------------------------------------------------------------
DmelOr22b       ------------------------------------------------------------
DmelOr42b       ------------------------------------------------------------
DmelOr59b       ------------------------------------------------------------
DmelOr59c       ------------------------------------------------------------
DmelOr98a       ------------------------------------------------------------
DmelOr42a       ------------------------------------------------------------
DmelOr85a       ------------------------------------------------------------
DmelOr43b       ------------------------------------------------------------
AgamOr2         ------------------------------------------------------------
AgamOr10        ------------------------------------------------------------
DmelOr30a       ------------------------------------------------------------
DmelOr49b       ------------------------------------------------------------
DmelOr43a       ------------------------------------------------------------
AgamOr32        ------------------------------------------------------------
AgamOr35        ------------------------------------------------------------
DmelOr47a       ------------------------------------------------------------
DmelOr98b       ------------------------------------------------------------
DmelOr9a        ------------------------------------------------------------
AgamOr38        ------------------------------------------------------------
AgamOr39        ------------------------------------------------------------
DmelOr24a       ------------------------------------------------------------
DmelOr45b       ------------------------------------------------------------
AmelOr74        ------------------------------------------------------------
AmelOr86        ------------------------------------------------------------
AmelOr75        ------------------------------------------------------------
AmelOr84        ------------------------------------------------------------
AmelOr87        ------------------------------------------------------------
AmelOr76        ------------------------------------------------------------
AmelOr85        ------------------------------------------------------------
AmelOr88        ------------------------------------------------------------
AmelOr91        ------------------------------------------------------------
AmelOr92        ------------------------------------------------------------
AmelOr95        ------------------------------------------------------------
AmelOr93        ------------------------------------------------------------
AmelOr77        ------------------------------------------------------------
AmelOr94        ------------------------------------------------------------
AmelOr96        ------------------------------------------------------------
AmelOr79        ------------------------------------------------------------
AmelOr83        ------------------------------------------------------------
AmelOr80        ------------------------------------------------------------
AmelOr81        ------------------------------------------------------------
AmelOr82        ------------------------------------------------------------
AmelOr78        ------------------------------------------------------------
AmelOr89        ------------------------------------------------------------
AmelOr90        ------------------------------------------------------------
AmelOr142       ------------------------------------------------------------
AmelOr146       ------------------------------------------------------------
AmelOr148       ------------------------------------------------------------
AmelOr158       ------------------------------------------------------------
AmelOr147       ------------------------------------------------------------
AmelOr150       ------------------------------------------------------------
AmelOr151       ------------------------------------------------------------
AmelOr152       ------------------------------------------------------------
AmelOr154       ------------------------------------------------------------
AmelOr155       ------------------------------------------------------------
AmelOr157       ------------------------------------------------------------
AmelOr156       ------------------------------------------------------------
AmelOr153       ------------------------------------------------------------
AmelOr144       ------------------------------------------------------------
AmelOr145       ------------------------------------------------------------
AmelOr143       ------------------------------------------------------------
AmelOr1         ------------------------------------------------------------
AmelOr3         ------------------------------------------------------------
AmelOr6         ------------------------------------------------------------
AmelOr7         ------------------------------------------------------------
AmelOr4         ------------------------------------------------------------
AmelOr5         ------------------------------------------------------------
```

```
AmelOr8         ------------------------------------------------------------
AmelOr9         ------------------------------------------------------------
AmelOr11        ------------------------------------------------------------
AmelOr12        ------------------------------------------------------------
AmelOr10        ------------------------------------------------------------
AmelOr14        ------------------------------------------------------------
AmelOr15        ------------------------------------------------------------
AmelOr13        ------------------------------------------------------------
AmelOr16        ------------------------------------------------------------
AmelOr17        ------------------------------------------------------------
AmelOr18        ------------------------------------------------------------
AmelOr19        ------------------------------------------------------------
AmelOr22        ------------------------------------------------------------
AmelOr24        ------------------------------------------------------------
AmelOr20        ------------------------------------------------------------
AmelOr25        ------------------------------------------------------------
AmelOr29        ------------------------------------------------------------
AmelOr30        ------------------------------------------------------------
AmelOr21        ------------------------------------------------------------
AmelOr23        ------------------------------------------------------------
AmelOr35        ------------------------------------------------------------
AmelOr27        ------------------------------------------------------------
AmelOr28        ------------------------------------------------------------
AmelOr33        ------------------------------------------------------------
AmelOr34        ------------------------------------------------------------
AmelOr32        ------------------------------------------------------------
AmelOr31        ------------------------------------------------------------
AmelOr36        ------------------------------------------------------------
AmelOr37        ------------------------------------------------------------
AmelOr38        ------------------------------------------------------------
AmelOr39        ------------------------------------------------------------
AmelOr47        ------------------------------------------------------------
AmelOr48        ------------------------------------------------------------
AmelOr40        ------------------------------------------------------------
AmelOr43        ------------------------------------------------------------
AmelOr45        ------------------------------------------------------------
AmelOr41        ------------------------------------------------------------
AmelOr42        ------------------------------------------------------------
AmelOr44        ------------------------------------------------------------
AmelOr46        ------------------------------------------------------------
AmelOr49        ------------------------------------------------------------
AmelOr50        ------------------------------------------------------------
AmelOr26        ------------------------------------------------------------
AmelOr53        ------------------------------------------------------------
AmelOr54        ------------------------------------------------------------
AmelOr52        ------------------------------------------------------------
AmelOr51        ------------------------------------------------------------
AmelOr55        ------------------------------------------------------------
AmelOr57        ------------------------------------------------------------
AmelOr56        ------------------------------------------------------------
AmelOr58        ------------------------------------------------------------
AmelOr59        ------------------------------------------------------------
AmelOr60        ------------------------------------------------------------
AmelOr61        ------------------------------------------------------------
AmelOr63        ------------------------------------------------------------
AmelOr64        ------------------------------------------------------------
AmelOr66        ------------------------------------------------------------
AmelOr65        ------------------------------------------------------------
AmelOr67        ------------------------------------------------------------
AmelOr114       ------------------------------------------------------------
AmelOr115       ------------------------------------------------------------
AmelOr118       ------------------------------------------------------------
AmelOr164       ------------------------------------------------------------
AmelOr163       ------------------------------------------------------------
AmelOr165       ------------------------------------------------------------
AmelOr166       ------------------------------------------------------------
AmelOr167       ------------------------------------------------------------
DmelOr35a       ------------------------------------------------------------
DmelOr74a       ------------------------------------------------------------
AgamOr3         ------------------------------------------------------------
```

```
AgamOr5         ------------------------------------------------------------
AgamOr13        ------------------------------------------------------------
AgamOr15        ------------------------------------------------------------
AgamOr17        ------------------------------------------------------------
AgamOr16        ------------------------------------------------------------
AgamOr55        ------------------------------------------------------------
AgamOr18        ------------------------------------------------------------
AgamOr14        ------------------------------------------------------------
AgamOr51        ------------------------------------------------------------
AgamOr12        ------------------------------------------------------------
AgamOr19        ------------------------------------------------------------
AgamOr20        ------------------------------------------------------------
AgamOr21        ------------------------------------------------------------
AgamOr50        ------------------------------------------------------------
AgamOr29        ------------------------------------------------------------
AgamOr53        ------------------------------------------------------------
AgamOr30        ------------------------------------------------------------
AgamOr46        ------------------------------------------------------------
AgamOr47        ------------------------------------------------------------
AgamOr48        ------------------------------------------------------------
AgamOr49        ------------------------------------------------------------
AgamOr61        ------------------------------------------------------------
AgamOr62        ------------------------------------------------------------
AgamOr63        ------------------------------------------------------------
AgamOr60        ------------------------------------------------------------
AgamOr75        ------------------------------------------------------------
AgamOr76        ------------------------------------------------------------
AgamOr78        ------------------------------------------------------------
AgamOr77        ------------------------------------------------------------
AgamOr79        ------------------------------------------------------------
AmelOr99        ------------------------------------------------------------
AmelOr101       ------------------------------------------------------------
AmelOr102       ------------------------------------------------------------
AmelOr103       ------------------------------------------------------------
AmelOr104       ------------------------------------------------------------
AmelOr98        ------------------------------------------------------------
AmelOr100       ------------------------------------------------------------
AmelOr97        ------------------------------------------------------------
AmelOr105       ------------------------------------------------------------
AmelOr109       ------------------------------------------------------------
AmelOr110       ------------------------------------------------------------
AmelOr111       ------------------------------------------------------------
AmelOr108       ------------------------------------------------------------
AmelOr107       ------------------------------------------------------------
AmelOr112       ------------------------------------------------------------
AmelOr113       ------------------------------------------------------------
AmelOr106       ------------------------------------------------------------
AmelOr122       ------------------------------------------------------------
AmelOr125       ------------------------------------------------------------
AmelOr123       ------------------------------------------------------------
AmelOr126       ------------------------------------------------------------
AmelOr127       ------------------------------------------------------------
AmelOr128       ------------------------------------------------------------
AmelOr129       ------------------------------------------------------------
AmelOr133       ------------------------------------------------------------
AmelOr134       ------------------------------------------------------------
AmelOr131       ------------------------------------------------------------
AmelOr132       ------------------------------------------------------------
AmelOr135       ------------------------------------------------------------
AmelOr136       ------------------------------------------------------------
AmelOr138       ------------------------------------------------------------
AmelOr130       ------------------------------------------------------------
AmelOr137       ------------------------------------------------------------
AmelOr139       ------------------------------------------------------------
BmorOr20        ------------------------------------------------------------
BmorOr3         ------------------------------------------------------------
BmorOr18        ------------------------------------------------------------
HvirOr6         ------------------------------------------------------------
BmorOr23        ------------------------------------------------------------
BmorOr16        ------------------------------------------------------------
```

```
BmorOr4          ------------------------------------------------------------
BmorOr5          ------------------------------------------------------------
BmorOr22         ------------------------------------------------------------
BmorOr17         ------------------------------------------------------------
BmorOr1          ------------------------------------------------------------
BmorOr1a         ------------------------------------------------------------
BmorOr1b         ------------------------------------------------------------
BmorOr19         ------------------------------------------------------------
BmorOr15         ------------------------------------------------------------
TcasOr5          ------------------------------------------------------------
TcasOr7          ------------------------------------------------------------
HvirOr4          ------------------------------------------------------------
BmorOr13         ------------------------------------------------------------
DmelOr10a        ------------------------------------------------------------
DmelOr33c        ------------------------------------------------------------
DmelOr7a         ------------------------------------------------------------
DmelOr22c        ------------------------------------------------------------
AgamOr1          ------------------------------------------------------------
AgamOr4          ------------------------------------------------------------
DmelOr47b        ------------------------------------------------------------
AmelOr73         ------------------------------------------------------------
AgamOr68         ------------------------------------------------------------
DmelOr59a        ------------------------------------------------------------
DmelOr23a        ------------------------------------------------------------
AgamOr31         ------------------------------------------------------------
DmelOr2a         ------------------------------------------------------------
AmelOr62         ------------------------------------------------------------
AmelOr121        ------------------------------------------------------------
AmelOr140        ------------------------------------------------------------
TcasOr12         ------------------------------------------------------------
AgamOr8          ------------------------------------------------------------
BmorOr9          ------------------------------------------------------------
AgamOr40         ------------------------------------------------------------
TcasOr63         ------------------------------------------------------------
TcasOr4          ------------------------------------------------------------
TcasOr13         ------------------------------------------------------------
AgamOr24         ------------------------------------------------------------
AgamOr54         ------------------------------------------------------------
AgamOr23         ------------------------------------------------------------
AgamOr64         ------------------------------------------------------------
DmelOr82a        ------------------------------------------------------------
AmelOr116        ------------------------------------------------------------
AmelOr160        ------------------------------------------------------------
TcasOr10         ------------------------------------------------------------
TcasOr26         ------------------------------------------------------------
AmelOr117        ------------------------------------------------------------
AmelOr120        ------------------------------------------------------------
BmorOr7          ------------------------------------------------------------
AgamOr6          ------------------------------------------------------------
DmelOr45a        ------------------------------------------------------------
DmelOr67d        ------------------------------------------------------------
DmelOr83c        ------------------------------------------------------------
AmelOr162        ------------------------------------------------------------
HvirOr3          ------------------------------------------------------------
TcasOr20         ------------------------------------------------------------
AgamOr11         ------------------------------------------------------------
DmelOr56a        ------------------------------------------------------------
AgamOr28         ------------------------------------------------------------
AgamOr58         ------------------------------------------------------------
AgamOr59         ------------------------------------------------------------
DmelOr1a         ------------------------------------------------------------
DmelOr13a        ------------------------------------------------------------
AmelOr161        ------------------------------------------------------------
AmelOr141        ------------------------------------------------------------
AgamOr52         ------------------------------------------------------------
AgamOr36         ------------------------------------------------------------
AgamOr25         ------------------------------------------------------------
AgamOr45         ------------------------------------------------------------
TcasOr3          ------------------------------------------------------------
DmelOr88a        ------------------------------------------------------------
```

```
AgamOr22        ------------------------------------------------------------
DmelOr49a       ------------------------------------------------------------
DmelOr85f       ------------------------------------------------------------
AmelOr119       ------------------------------------------------------------
DmelOr63a       ------------------------------------------------------------
DmelOr67b       ------------------------------------------------------------
BmorOr21        ------------------------------------------------------------
BmorOr11        ------------------------------------------------------------
BmorOr12        ------------------------------------------------------------
AgamOr33        ------------------------------------------------------------
HvirOr1         ------------------------------------------------------------
HvirOr5         ------------------------------------------------------------
TcasOr1         ------------------------------------------------------------
TcasOr2         ------------------------------------------------------------


TcasOr16        ------------------------------------------------------------
AmelOr2         ------------------------------------------------------------
AgamOr7         ------------------------------------------------------------
AaegOr7         ------------------------------------------------------------
DmelOr83b       ------------------------------------------------------------
BmorOr2         ------------------------------------------------------------
BmorOr2a        ------------------------------------------------------------
HvirOr2         ------------------------------------------------------------
AgamOr56        ------------------------------------------------------------
AgamOr57        ------------------------------------------------------------
AgamOr26        ------------------------------------------------------------
AgamOr27        ------------------------------------------------------------
HvirOr9         ------------------------------------------------------------
HvirOr7         ------------------------------------------------------------
DmelOr85b       ------------------------------------------------------------
DmelOr85c       ------------------------------------------------------------
DmelOr85d       ------------------------------------------------------------
DmelOr67a       ------------------------------------------------------------
DmelOr67c       ------------------------------------------------------------
DmelOr92a       ------------------------------------------------------------
DmelOr69a       ------------------------------------------------------------
DmelOr69b       ------------------------------------------------------------
TcasOr6         ------------------------------------------------------------
TcasOr11        ------------------------------------------------------------
AgamOr41        ------------------------------------------------------------
AgamOr42        ------------------------------------------------------------
AgamOr43        ------------------------------------------------------------
AgamOr44        ------------------------------------------------------------
AgamOr66        ------------------------------------------------------------
AgamOr67        ------------------------------------------------------------
AgamOr72        ------------------------------------------------------------
AgamOr73        ------------------------------------------------------------
AgamOr71        ------------------------------------------------------------
AgamOr74        ------------------------------------------------------------
AgamOr70        ------------------------------------------------------------
AgamOr69        ------------------------------------------------------------
TcasOr9         ------------------------------------------------------------
TcasOr18        ------------------------------------------------------------
DmelOr83a       ------------------------------------------------------------
DmelOr85e       ------------------------------------------------------------
TcasOr24        ------------------------------------------------------------
TcasOr25        FSLFVSPIITKHICERQNAARHFDDICGLVTNSWMPFDIKKFPNKQLFYLWQTYSVYWTY 684
TcasOr19        ------------------------------------------------------------
TcasOr22        ------------------------------------------------------------
TcasOr23        ------------------------------------------------------------
AmelOr168       ------------------------------------------------------------
AmelOr170       ------------------------------------------------------------
AmelOr169       ------------------------------------------------------------
DmelOr65b       ------------------------------------------------------------
DmelOr65c       ------------------------------------------------------------
DmelOr65a       ------------------------------------------------------------
TcasOr17        ------------------------------------------------------------
TcasOr21        ------------------------------------------------------------
TcasOr56        ------------------------------------------------------------
```

```
TcasOr15        ------------------------------------------------------------
AgamOr9         ------------------------------------------------------------
AgamOr65        ------------------------------------------------------------
BmorOr6         ------------------------------------------------------------
BmorOr10        ------------------------------------------------------------
AgamOr34        ------------------------------------------------------------
AgamOr37        ------------------------------------------------------------
DmelOr94a       ------------------------------------------------------------
DmelOr94b       ------------------------------------------------------------
DmelOr71a       ------------------------------------------------------------
DmelOr46a       ------------------------------------------------------------
DmelOr46b       ------------------------------------------------------------
HvirOr8         ------------------------------------------------------------
BmorOr8         ------------------------------------------------------------
AmelOr68        ------------------------------------------------------------
AmelOr69        ------------------------------------------------------------
AmelOr70        ------------------------------------------------------------
AmelOr71        ------------------------------------------------------------
AmelOr72        ------------------------------------------------------------
DmelOr19a       ------------------------------------------------------------
DmelOr19b       ------------------------------------------------------------
DmelOr33a       ------------------------------------------------------------
DmelOr33b       ------------------------------------------------------------
DmelOr22a       ------------------------------------------------------------
DmelOr22b       ------------------------------------------------------------
DmelOr42b       ------------------------------------------------------------
DmelOr59b       ------------------------------------------------------------
DmelOr59c       ------------------------------------------------------------
DmelOr98a       ------------------------------------------------------------
DmelOr42a       ------------------------------------------------------------
DmelOr85a       ------------------------------------------------------------
DmelOr43b       ------------------------------------------------------------
AgamOr2         ------------------------------------------------------------
AgamOr10        ------------------------------------------------------------
DmelOr30a       ------------------------------------------------------------
DmelOr49b       ------------------------------------------------------------
DmelOr43a       ------------------------------------------------------------
AgamOr32        ------------------------------------------------------------
AgamOr35        ------------------------------------------------------------
DmelOr47a       ------------------------------------------------------------
DmelOr98b       ------------------------------------------------------------
DmelOr9a        ------------------------------------------------------------
AgamOr38        ------------------------------------------------------------
AgamOr39        ------------------------------------------------------------
DmelOr24a       ------------------------------------------------------------
DmelOr45b       ------------------------------------------------------------
AmelOr74        ------------------------------------------------------------
AmelOr86        ------------------------------------------------------------
AmelOr75        ------------------------------------------------------------
AmelOr84        ------------------------------------------------------------
AmelOr87        ------------------------------------------------------------
AmelOr76        ------------------------------------------------------------
AmelOr85        ------------------------------------------------------------
AmelOr88        ------------------------------------------------------------
AmelOr91        ------------------------------------------------------------
AmelOr92        ------------------------------------------------------------
AmelOr95        ------------------------------------------------------------
AmelOr93        ------------------------------------------------------------
AmelOr77        ------------------------------------------------------------
AmelOr94        ------------------------------------------------------------
AmelOr96        ------------------------------------------------------------
AmelOr79        ------------------------------------------------------------
AmelOr83        ------------------------------------------------------------
AmelOr80        ------------------------------------------------------------
AmelOr81        ------------------------------------------------------------
AmelOr82        ------------------------------------------------------------
AmelOr78        ------------------------------------------------------------
AmelOr89        ------------------------------------------------------------
AmelOr90        ------------------------------------------------------------
AmelOr142       ------------------------------------------------------------
```

```
AmelOr146       ------------------------------------------------------------
AmelOr148       ------------------------------------------------------------
AmelOr158       ------------------------------------------------------------
AmelOr147       ------------------------------------------------------------
AmelOr150       ------------------------------------------------------------
AmelOr151       ------------------------------------------------------------
AmelOr152       ------------------------------------------------------------
AmelOr154       ------------------------------------------------------------
AmelOr155       ------------------------------------------------------------
AmelOr157       ------------------------------------------------------------
AmelOr156       ------------------------------------------------------------
AmelOr153       ------------------------------------------------------------
AmelOr144       ------------------------------------------------------------
AmelOr145       ------------------------------------------------------------
AmelOr143       ------------------------------------------------------------
AmelOr1         ------------------------------------------------------------
AmelOr3         ------------------------------------------------------------
AmelOr6         ------------------------------------------------------------
AmelOr7         ------------------------------------------------------------
AmelOr4         ------------------------------------------------------------
AmelOr5         ------------------------------------------------------------
AmelOr8         ------------------------------------------------------------
AmelOr9         ------------------------------------------------------------
AmelOr11        ------------------------------------------------------------
AmelOr12        ------------------------------------------------------------
AmelOr10        ------------------------------------------------------------
AmelOr14        ------------------------------------------------------------
AmelOr15        ------------------------------------------------------------
AmelOr13        ------------------------------------------------------------
AmelOr16        ------------------------------------------------------------
AmelOr17        ------------------------------------------------------------
AmelOr18        ------------------------------------------------------------
AmelOr19        ------------------------------------------------------------
AmelOr22        ------------------------------------------------------------
AmelOr24        ------------------------------------------------------------
AmelOr20        ------------------------------------------------------------
AmelOr25        ------------------------------------------------------------
AmelOr29        ------------------------------------------------------------
AmelOr30        ------------------------------------------------------------
AmelOr21        ------------------------------------------------------------
AmelOr23        ------------------------------------------------------------
AmelOr35        ------------------------------------------------------------
AmelOr27        ------------------------------------------------------------
AmelOr28        ------------------------------------------------------------
AmelOr33        ------------------------------------------------------------
AmelOr34        ------------------------------------------------------------
AmelOr32        ------------------------------------------------------------
AmelOr31        ------------------------------------------------------------
AmelOr36        ------------------------------------------------------------
AmelOr37        ------------------------------------------------------------
AmelOr38        ------------------------------------------------------------
AmelOr39        ------------------------------------------------------------
AmelOr47        ------------------------------------------------------------
AmelOr48        ------------------------------------------------------------
AmelOr40        ------------------------------------------------------------
AmelOr43        ------------------------------------------------------------
AmelOr45        ------------------------------------------------------------
AmelOr41        ------------------------------------------------------------
AmelOr42        ------------------------------------------------------------
AmelOr44        ------------------------------------------------------------
AmelOr46        ------------------------------------------------------------
AmelOr49        ------------------------------------------------------------
AmelOr50        ------------------------------------------------------------
AmelOr26        ------------------------------------------------------------
AmelOr53        ------------------------------------------------------------
AmelOr54        ------------------------------------------------------------
AmelOr52        ------------------------------------------------------------
AmelOr51        ------------------------------------------------------------
AmelOr55        ------------------------------------------------------------
AmelOr57        ------------------------------------------------------------
```

```
AmelOr56        ------------------------------------------------------------
AmelOr58        ------------------------------------------------------------
AmelOr59        ------------------------------------------------------------
AmelOr60        ------------------------------------------------------------
AmelOr61        ------------------------------------------------------------
AmelOr63        ------------------------------------------------------------
AmelOr64        ------------------------------------------------------------
AmelOr66        ------------------------------------------------------------
AmelOr65        ------------------------------------------------------------
AmelOr67        ------------------------------------------------------------
AmelOr114       ------------------------------------------------------------
AmelOr115       ------------------------------------------------------------
AmelOr118       ------------------------------------------------------------
AmelOr164       ------------------------------------------------------------
AmelOr163       ------------------------------------------------------------
AmelOr165       ------------------------------------------------------------
AmelOr166       ------------------------------------------------------------
AmelOr167       ------------------------------------------------------------
DmelOr35a       ------------------------------------------------------------
DmelOr74a       ------------------------------------------------------------
AgamOr3         ------------------------------------------------------------
AgamOr5         ------------------------------------------------------------
AgamOr13        ------------------------------------------------------------
AgamOr15        ------------------------------------------------------------
AgamOr17        ------------------------------------------------------------
AgamOr16        ------------------------------------------------------------
AgamOr55        ------------------------------------------------------------
AgamOr18        ------------------------------------------------------------
AgamOr14        ------------------------------------------------------------
AgamOr51        ------------------------------------------------------------
AgamOr12        ------------------------------------------------------------
AgamOr19        ------------------------------------------------------------
AgamOr20        ------------------------------------------------------------
AgamOr21        ------------------------------------------------------------
AgamOr50        ------------------------------------------------------------
AgamOr29        ------------------------------------------------------------
AgamOr53        ------------------------------------------------------------
AgamOr30        ------------------------------------------------------------
AgamOr46        ------------------------------------------------------------
AgamOr47        ------------------------------------------------------------
AgamOr48        ------------------------------------------------------------
AgamOr49        ------------------------------------------------------------
AgamOr61        ------------------------------------------------------------
AgamOr62        ------------------------------------------------------------
AgamOr63        ------------------------------------------------------------
AgamOr60        ------------------------------------------------------------
AgamOr75        ------------------------------------------------------------
AgamOr76        ------------------------------------------------------------
AgamOr78        ------------------------------------------------------------
AgamOr77        ------------------------------------------------------------
AgamOr79        ------------------------------------------------------------
AmelOr99        ------------------------------------------------------------
AmelOr101       ------------------------------------------------------------
AmelOr102       ------------------------------------------------------------
AmelOr103       ------------------------------------------------------------
AmelOr104       ------------------------------------------------------------
AmelOr98        ------------------------------------------------------------
AmelOr100       ------------------------------------------------------------
AmelOr97        ------------------------------------------------------------
AmelOr105       ------------------------------------------------------------
AmelOr109       ------------------------------------------------------------
AmelOr110       ------------------------------------------------------------
AmelOr111       ------------------------------------------------------------
AmelOr108       ------------------------------------------------------------
AmelOr107       ------------------------------------------------------------
AmelOr112       ------------------------------------------------------------
AmelOr113       ------------------------------------------------------------
AmelOr106       ------------------------------------------------------------
AmelOr122       ------------------------------------------------------------
AmelOr125       ------------------------------------------------------------
```

```
AmelOr123       ------------------------------------------------------------
AmelOr126       ------------------------------------------------------------
AmelOr127       ------------------------------------------------------------
AmelOr128       ------------------------------------------------------------
AmelOr129       ------------------------------------------------------------
AmelOr133       ------------------------------------------------------------
AmelOr134       ------------------------------------------------------------
AmelOr131       ------------------------------------------------------------
AmelOr132       ------------------------------------------------------------
AmelOr135       ------------------------------------------------------------
AmelOr136       ------------------------------------------------------------
AmelOr138       ------------------------------------------------------------
AmelOr130       ------------------------------------------------------------
AmelOr137       ------------------------------------------------------------
AmelOr139       ------------------------------------------------------------
BmorOr20        ------------------------------------------------------------
BmorOr3         ------------------------------------------------------------
BmorOr18        ------------------------------------------------------------
HvirOr6         ------------------------------------------------------------
BmorOr23        ------------------------------------------------------------
BmorOr16        ------------------------------------------------------------
BmorOr4         ------------------------------------------------------------
BmorOr5         ------------------------------------------------------------
BmorOr22        ------------------------------------------------------------
BmorOr17        ------------------------------------------------------------
BmorOr1         ------------------------------------------------------------
BmorOr1a        ------------------------------------------------------------
BmorOr1b        ------------------------------------------------------------
BmorOr19        ------------------------------------------------------------
BmorOr15        ------------------------------------------------------------
TcasOr5         ------------------------------------------------------------
TcasOr7         ------------------------------------------------------------
HvirOr4         ------------------------------------------------------------
BmorOr13        ------------------------------------------------------------
DmelOr10a       ------------------------------------------------------------
DmelOr33c       ------------------------------------------------------------
DmelOr7a        ------------------------------------------------------------
DmelOr22c       ------------------------------------------------------------
AgamOr1         ------------------------------------------------------------
AgamOr4         ------------------------------------------------------------
DmelOr47b       ------------------------------------------------------------
AmelOr73        ------------------------------------------------------------
AgamOr68        ------------------------------------------------------------
DmelOr59a       ------------------------------------------------------------
DmelOr23a       ------------------------------------------------------------
AgamOr31        ------------------------------------------------------------
DmelOr2a        ------------------------------------------------------------
AmelOr62        ------------------------------------------------------------
AmelOr121       ------------------------------------------------------------
AmelOr140       ------------------------------------------------------------
TcasOr12        ------------------------------------------------------------
AgamOr8         ------------------------------------------------------------
BmorOr9         ------------------------------------------------------------
AgamOr40        ------------------------------------------------------------
TcasOr63        ------------------------------------------------------------
TcasOr4         ------------------------------------------------------------
TcasOr13        ------------------------------------------------------------
AgamOr24        ------------------------------------------------------------
AgamOr54        ------------------------------------------------------------
AgamOr23        ------------------------------------------------------------
AgamOr64        ------------------------------------------------------------
DmelOr82a       ------------------------------------------------------------
AmelOr116       ------------------------------------------------------------
AmelOr160       ------------------------------------------------------------
TcasOr10        ------------------------------------------------------------
TcasOr26        ------------------------------------------------------------
AmelOr117       ------------------------------------------------------------
AmelOr120       ------------------------------------------------------------
BmorOr7         ------------------------------------------------------------
AgamOr6         ------------------------------------------------------------
```

```
DmelOr45a       ------------------------------------------------------------
DmelOr67d       ------------------------------------------------------------
DmelOr83c       ------------------------------------------------------------
AmelOr162       ------------------------------------------------------------
HvirOr3         ------------------------------------------------------------
TcasOr20        ------------------------------------------------------------
AgamOr11        ------------------------------------------------------------
DmelOr56a       ------------------------------------------------------------
AgamOr28        ------------------------------------------------------------
AgamOr58        ------------------------------------------------------------
AgamOr59        ------------------------------------------------------------
DmelOr1a        ------------------------------------------------------------
DmelOr13a       ------------------------------------------------------------
AmelOr161       ------------------------------------------------------------
AmelOr141       ------------------------------------------------------------
AgamOr52        ------------------------------------------------------------
AgamOr36        ------------------------------------------------------------
AgamOr25        ------------------------------------------------------------
AgamOr45        ------------------------------------------------------------
TcasOr3         ------------------------------------------------------------
DmelOr88a       ------------------------------------------------------------
AgamOr22        ------------------------------------------------------------
DmelOr49a       ------------------------------------------------------------
DmelOr85f       ------------------------------------------------------------
AmelOr119       ------------------------------------------------------------
DmelOr63a       ------------------------------------------------------------
DmelOr67b       ------------------------------------------------------------
BmorOr21        ------------------------------------------------------------
BmorOr11        ------------------------------------------------------------
BmorOr12        ------------------------------------------------------------
AgamOr33        ------------------------------------------------------------
HvirOr1         ------------------------------------------------------------
HvirOr5         ------------------------------------------------------------
TcasOr1         ------------------------------------------------------------
TcasOr2         ------------------------------------------------------------


TcasOr16        ------------------------------------------------------------
AmelOr2         ------------------------------------------------------------
AgamOr7         ------------------------------------------------------------
AaegOr7         ------------------------------------------------------------
DmelOr83b       ------------------------------------------------------------
BmorOr2         ------------------------------------------------------------
BmorOr2a        ------------------------------------------------------------
HvirOr2         ------------------------------------------------------------
AgamOr56        ------------------------------------------------------------
AgamOr57        ------------------------------------------------------------
AgamOr26        ------------------------------------------------------------
AgamOr27        ------------------------------------------------------------
HvirOr9         ------------------------------------------------------------
HvirOr7         ------------------------------------------------------------
DmelOr85b       ------------------------------------------------------------
DmelOr85c       ------------------------------------------------------------
DmelOr85d       ------------------------------------------------------------
DmelOr67a       ------------------------------------------------------------
DmelOr67c       ------------------------------------------------------------
DmelOr92a       ------------------------------------------------------------
DmelOr69a       ------------------------------------------------------------
DmelOr69b       ------------------------------------------------------------
TcasOr6         ------------------------------------------------------------
TcasOr11        ------------------------------------------------------------
AgamOr41        ------------------------------------------------------------
AgamOr42        ------------------------------------------------------------
AgamOr43        ------------------------------------------------------------
AgamOr44        ------------------------------------------------------------
AgamOr66        ------------------------------------------------------------
AgamOr67        ------------------------------------------------------------
AgamOr72        ------------------------------------------------------------
AgamOr73        ------------------------------------------------------------
AgamOr71        ------------------------------------------------------------
```

```
AgamOr74        ------------------------------------------------------------
AgamOr70        ------------------------------------------------------------
AgamOr69        ------------------------------------------------------------
TcasOr9         ------------------------------------------------------------
TcasOr18        ------------------------------------------------------------
DmelOr83a       ------------------------------------------------------------
DmelOr85e       ------------------------------------------------------------
TcasOr24        ------------------------------------------------------------
TcasOr25        EGSAAISYLIVATMEHVLMRIWHLQGMIQEAINTEDAQVRKSRLDKCLEYHNHVFLLAAD 744
TcasOr19        ------------------------------------------------------------
TcasOr22        ------------------------------------------------------------
TcasOr23        ------------------------------------------------------------
AmelOr168       ------------------------------------------------------------
AmelOr170       ------------------------------------------------------------
AmelOr169       ------------------------------------------------------------
DmelOr65b       ------------------------------------------------------------
DmelOr65c       ------------------------------------------------------------
DmelOr65a       ------------------------------------------------------------
TcasOr17        ------------------------------------------------------------
TcasOr21        ------------------------------------------------------------
TcasOr56        ------------------------------------------------------------
TcasOr15        ------------------------------------------------------------
AgamOr9         ------------------------------------------------------------
AgamOr65        ------------------------------------------------------------
BmorOr6         ------------------------------------------------------------
BmorOr10        ------------------------------------------------------------
AgamOr34        ------------------------------------------------------------
AgamOr37        ------------------------------------------------------------
DmelOr94a       ------------------------------------------------------------
DmelOr94b       ------------------------------------------------------------
DmelOr71a       ------------------------------------------------------------
DmelOr46a       ------------------------------------------------------------
DmelOr46b       ------------------------------------------------------------
HvirOr8         ------------------------------------------------------------
BmorOr8         ------------------------------------------------------------
AmelOr68        ------------------------------------------------------------
AmelOr69        ------------------------------------------------------------
AmelOr70        ------------------------------------------------------------
AmelOr71        ------------------------------------------------------------
AmelOr72        ------------------------------------------------------------
DmelOr19a       ------------------------------------------------------------
DmelOr19b       ------------------------------------------------------------
DmelOr33a       ------------------------------------------------------------
DmelOr33b       ------------------------------------------------------------
DmelOr22a       ------------------------------------------------------------
DmelOr22b       ------------------------------------------------------------
DmelOr42b       ------------------------------------------------------------
DmelOr59b       ------------------------------------------------------------
DmelOr59c       ------------------------------------------------------------
DmelOr98a       ------------------------------------------------------------
DmelOr42a       ------------------------------------------------------------
DmelOr85a       ------------------------------------------------------------
DmelOr43b       ------------------------------------------------------------
AgamOr2         ------------------------------------------------------------
AgamOr10        ------------------------------------------------------------
DmelOr30a       ------------------------------------------------------------
DmelOr49b       ------------------------------------------------------------
DmelOr43a       ------------------------------------------------------------
AgamOr32        ------------------------------------------------------------
AgamOr35        ------------------------------------------------------------
DmelOr47a       ------------------------------------------------------------
DmelOr98b       ------------------------------------------------------------
DmelOr9a        ------------------------------------------------------------
AgamOr38        ------------------------------------------------------------
AgamOr39        ------------------------------------------------------------
DmelOr24a       ------------------------------------------------------------
DmelOr45b       ------------------------------------------------------------
AmelOr74        ------------------------------------------------------------
AmelOr86        ------------------------------------------------------------
AmelOr75        ------------------------------------------------------------
```

```
AmelOr84        ------------------------------------------------------------
AmelOr87        ------------------------------------------------------------
AmelOr76        ------------------------------------------------------------
AmelOr85        ------------------------------------------------------------
AmelOr88        ------------------------------------------------------------
AmelOr91        ------------------------------------------------------------
AmelOr92        ------------------------------------------------------------
AmelOr95        ------------------------------------------------------------
AmelOr93        ------------------------------------------------------------
AmelOr77        ------------------------------------------------------------
AmelOr94        ------------------------------------------------------------
AmelOr96        ------------------------------------------------------------
AmelOr79        ------------------------------------------------------------
AmelOr83        ------------------------------------------------------------
AmelOr80        ------------------------------------------------------------
AmelOr81        ------------------------------------------------------------
AmelOr82        ------------------------------------------------------------
AmelOr78        ------------------------------------------------------------
AmelOr89        ------------------------------------------------------------
AmelOr90        ------------------------------------------------------------
AmelOr142       ------------------------------------------------------------
AmelOr146       ------------------------------------------------------------
AmelOr148       ------------------------------------------------------------
AmelOr158       ------------------------------------------------------------
AmelOr147       ------------------------------------------------------------
AmelOr150       ------------------------------------------------------------
AmelOr151       ------------------------------------------------------------
AmelOr152       ------------------------------------------------------------
AmelOr154       ------------------------------------------------------------
AmelOr155       ------------------------------------------------------------
AmelOr157       ------------------------------------------------------------
AmelOr156       ------------------------------------------------------------
AmelOr153       ------------------------------------------------------------
AmelOr144       ------------------------------------------------------------
AmelOr145       ------------------------------------------------------------
AmelOr143       ------------------------------------------------------------
AmelOr1         ------------------------------------------------------------
AmelOr3         ------------------------------------------------------------
AmelOr6         ------------------------------------------------------------
AmelOr7         ------------------------------------------------------------
AmelOr4         ------------------------------------------------------------
AmelOr5         ------------------------------------------------------------
AmelOr8         ------------------------------------------------------------
AmelOr9         ------------------------------------------------------------
AmelOr11        ------------------------------------------------------------
AmelOr12        ------------------------------------------------------------
AmelOr10        ------------------------------------------------------------
AmelOr14        ------------------------------------------------------------
AmelOr15        ------------------------------------------------------------
AmelOr13        ------------------------------------------------------------
AmelOr16        ------------------------------------------------------------
AmelOr17        ------------------------------------------------------------
AmelOr18        ------------------------------------------------------------
AmelOr19        ------------------------------------------------------------
AmelOr22        ------------------------------------------------------------
AmelOr24        ------------------------------------------------------------
AmelOr20        ------------------------------------------------------------
AmelOr25        ------------------------------------------------------------
AmelOr29        ------------------------------------------------------------
AmelOr30        ------------------------------------------------------------
AmelOr21        ------------------------------------------------------------
AmelOr23        ------------------------------------------------------------
AmelOr35        ------------------------------------------------------------
AmelOr27        ------------------------------------------------------------
AmelOr28        ------------------------------------------------------------
AmelOr33        ------------------------------------------------------------
AmelOr34        ------------------------------------------------------------
AmelOr32        ------------------------------------------------------------
AmelOr31        ------------------------------------------------------------
AmelOr36        ------------------------------------------------------------
```

```
AmelOr37        ------------------------------------------------------------
AmelOr38        ------------------------------------------------------------
AmelOr39        ------------------------------------------------------------
AmelOr47        ------------------------------------------------------------
AmelOr48        ------------------------------------------------------------
AmelOr40        ------------------------------------------------------------
AmelOr43        ------------------------------------------------------------
AmelOr45        ------------------------------------------------------------
AmelOr41        ------------------------------------------------------------
AmelOr42        ------------------------------------------------------------
AmelOr44        ------------------------------------------------------------
AmelOr46        ------------------------------------------------------------
AmelOr49        ------------------------------------------------------------
AmelOr50        ------------------------------------------------------------
AmelOr26        ------------------------------------------------------------
AmelOr53        ------------------------------------------------------------
AmelOr54        ------------------------------------------------------------
AmelOr52        ------------------------------------------------------------
AmelOr51        ------------------------------------------------------------
AmelOr55        ------------------------------------------------------------
AmelOr57        ------------------------------------------------------------
AmelOr56        ------------------------------------------------------------
AmelOr58        ------------------------------------------------------------
AmelOr59        ------------------------------------------------------------
AmelOr60        ------------------------------------------------------------
AmelOr61        ------------------------------------------------------------
AmelOr63        ------------------------------------------------------------
AmelOr64        ------------------------------------------------------------
AmelOr66        ------------------------------------------------------------
AmelOr65        ------------------------------------------------------------
AmelOr67        ------------------------------------------------------------
AmelOr114       ------------------------------------------------------------
AmelOr115       ------------------------------------------------------------
AmelOr118       ------------------------------------------------------------
AmelOr164       ------------------------------------------------------------
AmelOr163       ------------------------------------------------------------
AmelOr165       ------------------------------------------------------------
AmelOr166       ------------------------------------------------------------
AmelOr167       ------------------------------------------------------------
DmelOr35a       ------------------------------------------------------------
DmelOr74a       ------------------------------------------------------------
AgamOr3         ------------------------------------------------------------
AgamOr5         ------------------------------------------------------------
AgamOr13        ------------------------------------------------------------
AgamOr15        ------------------------------------------------------------
AgamOr17        ------------------------------------------------------------
AgamOr16        ------------------------------------------------------------
AgamOr55        ------------------------------------------------------------
AgamOr18        ------------------------------------------------------------
AgamOr14        ------------------------------------------------------------
AgamOr51        ------------------------------------------------------------
AgamOr12        ------------------------------------------------------------
AgamOr19        ------------------------------------------------------------
AgamOr20        ------------------------------------------------------------
AgamOr21        ------------------------------------------------------------
AgamOr50        ------------------------------------------------------------
AgamOr29        ------------------------------------------------------------
AgamOr53        ------------------------------------------------------------
AgamOr30        ------------------------------------------------------------
AgamOr46        ------------------------------------------------------------
AgamOr47        ------------------------------------------------------------
AgamOr48        ------------------------------------------------------------
AgamOr49        ------------------------------------------------------------
AgamOr61        ------------------------------------------------------------
AgamOr62        ------------------------------------------------------------
AgamOr63        ------------------------------------------------------------
AgamOr60        ------------------------------------------------------------
AgamOr75        ------------------------------------------------------------
AgamOr76        ------------------------------------------------------------
AgamOr78        ------------------------------------------------------------
```

```
AgamOr77        ------------------------------------------------------------
AgamOr79        ------------------------------------------------------------
AmelOr99        ------------------------------------------------------------
AmelOr101       ------------------------------------------------------------
AmelOr102       ------------------------------------------------------------
AmelOr103       ------------------------------------------------------------
AmelOr104       ------------------------------------------------------------
AmelOr98        ------------------------------------------------------------
AmelOr100       ------------------------------------------------------------
AmelOr97        ------------------------------------------------------------
AmelOr105       ------------------------------------------------------------
AmelOr109       ------------------------------------------------------------
AmelOr110       ------------------------------------------------------------
AmelOr111       ------------------------------------------------------------
AmelOr108       ------------------------------------------------------------
AmelOr107       ------------------------------------------------------------
AmelOr112       ------------------------------------------------------------
AmelOr113       ------------------------------------------------------------
AmelOr106       ------------------------------------------------------------
AmelOr122       ------------------------------------------------------------
AmelOr125       ------------------------------------------------------------
AmelOr123       ------------------------------------------------------------
AmelOr126       ------------------------------------------------------------
AmelOr127       ------------------------------------------------------------
AmelOr128       ------------------------------------------------------------
AmelOr129       ------------------------------------------------------------
AmelOr133       ------------------------------------------------------------
AmelOr134       ------------------------------------------------------------
AmelOr131       ------------------------------------------------------------
AmelOr132       ------------------------------------------------------------
AmelOr135       ------------------------------------------------------------
AmelOr136       ------------------------------------------------------------
AmelOr138       ------------------------------------------------------------
AmelOr130       ------------------------------------------------------------
AmelOr137       ------------------------------------------------------------
AmelOr139       ------------------------------------------------------------
BmorOr20        ------------------------------------------------------------
BmorOr3         ------------------------------------------------------------
BmorOr18        ------------------------------------------------------------
HvirOr6         ------------------------------------------------------------
BmorOr23        ------------------------------------------------------------
BmorOr16        ------------------------------------------------------------
BmorOr4         ------------------------------------------------------------
BmorOr5         ------------------------------------------------------------
BmorOr22        ------------------------------------------------------------
BmorOr17        ------------------------------------------------------------
BmorOr1         ------------------------------------------------------------
BmorOr1a        ------------------------------------------------------------
BmorOr1b        ------------------------------------------------------------
BmorOr19        ------------------------------------------------------------
BmorOr15        ------------------------------------------------------------
TcasOr5         ------------------------------------------------------------
TcasOr7         ------------------------------------------------------------
HvirOr4         ------------------------------------------------------------
BmorOr13        ------------------------------------------------------------
DmelOr10a       ------------------------------------------------------------
DmelOr33c       ------------------------------------------------------------
DmelOr7a        ------------------------------------------------------------
DmelOr22c       ------------------------------------------------------------
AgamOr1         ------------------------------------------------------------
AgamOr4         ------------------------------------------------------------
DmelOr47b       ------------------------------------------------------------
AmelOr73        ------------------------------------------------------------
AgamOr68        ------------------------------------------------------------
DmelOr59a       ------------------------------------------------------------
DmelOr23a       ------------------------------------------------------------
AgamOr31        ------------------------------------------------------------
DmelOr2a        ------------------------------------------------------------
AmelOr62        ------------------------------------------------------------
AmelOr121       ------------------------------------------------------------
```

```
AmelOr140      ------------------------------------------------------------
TcasOr12       ------------------------------------------------------------
AgamOr8        ------------------------------------------------------------
BmorOr9        ------------------------------------------------------------
AgamOr40       ------------------------------------------------------------
TcasOr63       ------------------------------------------------------------
TcasOr4        ------------------------------------------------------------
TcasOr13       ------------------------------------------------------------
AgamOr24       ------------------------------------------------------------
AgamOr54       ------------------------------------------------------------
AgamOr23       ------------------------------------------------------------
AgamOr64       ------------------------------------------------------------
DmelOr82a      ------------------------------------------------------------
AmelOr116      ------------------------------------------------------------
AmelOr160      ------------------------------------------------------------
TcasOr10       ------------------------------------------------------------
TcasOr26       ------------------------------------------------------------
AmelOr117      ------------------------------------------------------------
AmelOr120      ------------------------------------------------------------
BmorOr7        ------------------------------------------------------------
AgamOr6        ------------------------------------------------------------
DmelOr45a      ------------------------------------------------------------
DmelOr67d      ------------------------------------------------------------
DmelOr83c      ------------------------------------------------------------
AmelOr162      ------------------------------------------------------------
HvirOr3        ------------------------------------------------------------
TcasOr20       ------------------------------------------------------------
AgamOr11       ------------------------------------------------------------
DmelOr56a      ------------------------------------------------------------
AgamOr28       ------------------------------------------------------------
AgamOr58       ------------------------------------------------------------
AgamOr59       ------------------------------------------------------------
DmelOr1a       ------------------------------------------------------------
DmelOr13a      ------------------------------------------------------------
AmelOr161      ------------------------------------------------------------
AmelOr141      ------------------------------------------------------------
AgamOr52       ------------------------------------------------------------
AgamOr36       ------------------------------------------------------------
AgamOr25       ------------------------------------------------------------
AgamOr45       ------------------------------------------------------------
TcasOr3        ------------------------------------------------------------
DmelOr88a      ------------------------------------------------------------
AgamOr22       ------------------------------------------------------------
DmelOr49a      ------------------------------------------------------------
DmelOr85f      ------------------------------------------------------------
AmelOr119      ------------------------------------------------------------
DmelOr63a      ------------------------------------------------------------
DmelOr67b      ------------------------------------------------------------
BmorOr21       ------------------------------------------------------------
BmorOr11       ------------------------------------------------------------
BmorOr12       ------------------------------------------------------------
AgamOr33       ------------------------------------------------------------
HvirOr1        ------------------------------------------------------------
HvirOr5        ------------------------------------------------------------
TcasOr1        ------------------------------------------------------------
TcasOr2        ------------------------------------------------------------


TcasOr16       -----------------------------------------------
AmelOr2        -----------------------------------------------
AgamOr7        -----------------------------------------------
AaegOr7        -----------------------------------------------
DmelOr83b      -----------------------------------------------
BmorOr2        -----------------------------------------------
BmorOr2a       -----------------------------------------------
HvirOr2        -----------------------------------------------
AgamOr56       -----------------------------------------------
AgamOr57       -----------------------------------------------
AgamOr26       -----------------------------------------------
AgamOr27       -----------------------------------------------
```

```
HvirOr9         -----------------------------------------------
HvirOr7         -----------------------------------------------
DmelOr85b       -----------------------------------------------
DmelOr85c       -----------------------------------------------
DmelOr85d       -----------------------------------------------
DmelOr67a       -----------------------------------------------
DmelOr67c       -----------------------------------------------
DmelOr92a       -----------------------------------------------
DmelOr69a       -----------------------------------------------
DmelOr69b       -----------------------------------------------
TcasOr6         -----------------------------------------------
TcasOr11        -----------------------------------------------
AgamOr41        -----------------------------------------------
AgamOr42        -----------------------------------------------
AgamOr43        -----------------------------------------------
AgamOr44        -----------------------------------------------
AgamOr66        -----------------------------------------------
AgamOr67        -----------------------------------------------
AgamOr72        -----------------------------------------------
AgamOr73        -----------------------------------------------
AgamOr71        -----------------------------------------------
AgamOr74        -----------------------------------------------
AgamOr70        -----------------------------------------------
AgamOr69        -----------------------------------------------
TcasOr9         -----------------------------------------------
TcasOr18        -----------------------------------------------
DmelOr83a       -----------------------------------------------
DmelOr85e       -----------------------------------------------
TcasOr24        -----------------------------------------------
TcasOr25        VDRIYQRSLFVHVLFSGVLFGIMGFSILTVGISVKTLTCRRKGFMMG 791
TcasOr19        -----------------------------------------------
TcasOr22        -----------------------------------------------
TcasOr23        -----------------------------------------------
AmelOr168       -----------------------------------------------
AmelOr170       -----------------------------------------------
AmelOr169       -----------------------------------------------
DmelOr65b       -----------------------------------------------
DmelOr65c       -----------------------------------------------
DmelOr65a       -----------------------------------------------
TcasOr17        -----------------------------------------------
TcasOr21        -----------------------------------------------
TcasOr56        -----------------------------------------------
TcasOr15        -----------------------------------------------
AgamOr9         -----------------------------------------------
AgamOr65        -----------------------------------------------
BmorOr6         -----------------------------------------------
BmorOr10        -----------------------------------------------
AgamOr34        -----------------------------------------------
AgamOr37        -----------------------------------------------
DmelOr94a       -----------------------------------------------
DmelOr94b       -----------------------------------------------
DmelOr71a       -----------------------------------------------
DmelOr46a       -----------------------------------------------
DmelOr46b       -----------------------------------------------
HvirOr8         -----------------------------------------------
BmorOr8         -----------------------------------------------
AmelOr68        -----------------------------------------------
AmelOr69        -----------------------------------------------
AmelOr70        -----------------------------------------------
AmelOr71        -----------------------------------------------
AmelOr72        -----------------------------------------------
DmelOr19a       -----------------------------------------------
DmelOr19b       -----------------------------------------------
DmelOr33a       -----------------------------------------------
DmelOr33b       -----------------------------------------------
DmelOr22a       -----------------------------------------------
DmelOr22b       -----------------------------------------------
DmelOr42b       -----------------------------------------------
DmelOr59b       -----------------------------------------------
DmelOr59c       -----------------------------------------------
```

```
DmelOr98a       -----------------------------------------------
DmelOr42a       -----------------------------------------------
DmelOr85a       -----------------------------------------------
DmelOr43b       -----------------------------------------------
AgamOr2         -----------------------------------------------
AgamOr10        -----------------------------------------------
DmelOr30a       -----------------------------------------------
DmelOr49b       -----------------------------------------------
DmelOr43a       -----------------------------------------------
AgamOr32        -----------------------------------------------
AgamOr35        -----------------------------------------------
DmelOr47a       -----------------------------------------------
DmelOr98b       -----------------------------------------------
DmelOr9a        -----------------------------------------------
AgamOr38        -----------------------------------------------
AgamOr39        -----------------------------------------------
DmelOr24a       -----------------------------------------------
DmelOr45b       -----------------------------------------------
AmelOr74        -----------------------------------------------
AmelOr86        -----------------------------------------------
AmelOr75        -----------------------------------------------
AmelOr84        -----------------------------------------------
AmelOr87        -----------------------------------------------
AmelOr76        -----------------------------------------------
AmelOr85        -----------------------------------------------
AmelOr88        -----------------------------------------------
AmelOr91        -----------------------------------------------
AmelOr92        -----------------------------------------------
AmelOr95        -----------------------------------------------
AmelOr93        -----------------------------------------------
AmelOr77        -----------------------------------------------
AmelOr94        -----------------------------------------------
AmelOr96        -----------------------------------------------
AmelOr79        -----------------------------------------------
AmelOr83        -----------------------------------------------
AmelOr80        -----------------------------------------------
AmelOr81        -----------------------------------------------
AmelOr82        -----------------------------------------------
AmelOr78        -----------------------------------------------
AmelOr89        -----------------------------------------------
AmelOr90        -----------------------------------------------
AmelOr142       -----------------------------------------------
AmelOr146       -----------------------------------------------
AmelOr148       -----------------------------------------------
AmelOr158       -----------------------------------------------
AmelOr147       -----------------------------------------------
AmelOr150       -----------------------------------------------
AmelOr151       -----------------------------------------------
AmelOr152       -----------------------------------------------
AmelOr154       -----------------------------------------------
AmelOr155       -----------------------------------------------
AmelOr157       -----------------------------------------------
AmelOr156       -----------------------------------------------
AmelOr153       -----------------------------------------------
AmelOr144       -----------------------------------------------
AmelOr145       -----------------------------------------------
AmelOr143       -----------------------------------------------
AmelOr1         -----------------------------------------------
AmelOr3         -----------------------------------------------
AmelOr6         -----------------------------------------------
AmelOr7         -----------------------------------------------
AmelOr4         -----------------------------------------------
AmelOr5         -----------------------------------------------
AmelOr8         -----------------------------------------------
AmelOr9         -----------------------------------------------
AmelOr11        -----------------------------------------------
AmelOr12        -----------------------------------------------
AmelOr10        -----------------------------------------------
AmelOr14        -----------------------------------------------
AmelOr15        -----------------------------------------------
```

```
AmelOr13        -----------------------------------------------
AmelOr16        -----------------------------------------------
AmelOr17        -----------------------------------------------
AmelOr18        -----------------------------------------------
AmelOr19        -----------------------------------------------
AmelOr22        -----------------------------------------------
AmelOr24        -----------------------------------------------
AmelOr20        -----------------------------------------------
AmelOr25        -----------------------------------------------
AmelOr29        -----------------------------------------------
AmelOr30        -----------------------------------------------
AmelOr21        -----------------------------------------------
AmelOr23        -----------------------------------------------
AmelOr35        -----------------------------------------------
AmelOr27        -----------------------------------------------
AmelOr28        -----------------------------------------------
AmelOr33        -----------------------------------------------
AmelOr34        -----------------------------------------------
AmelOr32        -----------------------------------------------
AmelOr31        -----------------------------------------------
AmelOr36        -----------------------------------------------
AmelOr37        -----------------------------------------------
AmelOr38        -----------------------------------------------
AmelOr39        -----------------------------------------------
AmelOr47        -----------------------------------------------
AmelOr48        -----------------------------------------------
AmelOr40        -----------------------------------------------
AmelOr43        -----------------------------------------------
AmelOr45        -----------------------------------------------
AmelOr41        -----------------------------------------------
AmelOr42        -----------------------------------------------
AmelOr44        -----------------------------------------------
AmelOr46        -----------------------------------------------
AmelOr49        -----------------------------------------------
AmelOr50        -----------------------------------------------
AmelOr26        -----------------------------------------------
AmelOr53        -----------------------------------------------
AmelOr54        -----------------------------------------------
AmelOr52        -----------------------------------------------
AmelOr51        -----------------------------------------------
AmelOr55        -----------------------------------------------
AmelOr57        -----------------------------------------------
AmelOr56        -----------------------------------------------
AmelOr58        -----------------------------------------------
AmelOr59        -----------------------------------------------
AmelOr60        -----------------------------------------------
AmelOr61        -----------------------------------------------
AmelOr63        -----------------------------------------------
AmelOr64        -----------------------------------------------
AmelOr66        -----------------------------------------------
AmelOr65        -----------------------------------------------
AmelOr67        -----------------------------------------------
AmelOr114       -----------------------------------------------
AmelOr115       -----------------------------------------------
AmelOr118       -----------------------------------------------
AmelOr164       -----------------------------------------------
AmelOr163       -----------------------------------------------
AmelOr165       -----------------------------------------------
AmelOr166       -----------------------------------------------
AmelOr167       -----------------------------------------------
DmelOr35a       -----------------------------------------------
DmelOr74a       -----------------------------------------------
AgamOr3         -----------------------------------------------
AgamOr5         -----------------------------------------------
AgamOr13        -----------------------------------------------
AgamOr15        -----------------------------------------------
AgamOr17        -----------------------------------------------
AgamOr16        -----------------------------------------------
AgamOr55        -----------------------------------------------
AgamOr18        -----------------------------------------------
```

```
AgamOr14        -----------------------------------------------
AgamOr51        -----------------------------------------------
AgamOr12        -----------------------------------------------
AgamOr19        -----------------------------------------------
AgamOr20        -----------------------------------------------
AgamOr21        -----------------------------------------------
AgamOr50        -----------------------------------------------
AgamOr29        -----------------------------------------------
AgamOr53        -----------------------------------------------
AgamOr30        -----------------------------------------------
AgamOr46        -----------------------------------------------
AgamOr47        -----------------------------------------------
AgamOr48        -----------------------------------------------
AgamOr49        -----------------------------------------------
AgamOr61        -----------------------------------------------
AgamOr62        -----------------------------------------------
AgamOr63        -----------------------------------------------
AgamOr60        -----------------------------------------------
AgamOr75        -----------------------------------------------
AgamOr76        -----------------------------------------------
AgamOr78        -----------------------------------------------
AgamOr77        -----------------------------------------------
AgamOr79        -----------------------------------------------
AmelOr99        -----------------------------------------------
AmelOr101       -----------------------------------------------
AmelOr102       -----------------------------------------------
AmelOr103       -----------------------------------------------
AmelOr104       -----------------------------------------------
AmelOr98        -----------------------------------------------
AmelOr100       -----------------------------------------------
AmelOr97        -----------------------------------------------
AmelOr105       -----------------------------------------------
AmelOr109       -----------------------------------------------
AmelOr110       -----------------------------------------------
AmelOr111       -----------------------------------------------
AmelOr108       -----------------------------------------------
AmelOr107       -----------------------------------------------
AmelOr112       -----------------------------------------------
AmelOr113       -----------------------------------------------
AmelOr106       -----------------------------------------------
AmelOr122       -----------------------------------------------
AmelOr125       -----------------------------------------------
AmelOr123       -----------------------------------------------
AmelOr126       -----------------------------------------------
AmelOr127       -----------------------------------------------
AmelOr128       -----------------------------------------------
AmelOr129       -----------------------------------------------
AmelOr133       -----------------------------------------------
AmelOr134       -----------------------------------------------
AmelOr131       -----------------------------------------------
AmelOr132       -----------------------------------------------
AmelOr135       -----------------------------------------------
AmelOr136       -----------------------------------------------
AmelOr138       -----------------------------------------------
AmelOr130       -----------------------------------------------
AmelOr137       -----------------------------------------------
AmelOr139       -----------------------------------------------
BmorOr20        -----------------------------------------------
BmorOr3         -----------------------------------------------
BmorOr18        -----------------------------------------------
HvirOr6         -----------------------------------------------
BmorOr23        -----------------------------------------------
BmorOr16        -----------------------------------------------
BmorOr4         -----------------------------------------------
BmorOr5         -----------------------------------------------
BmorOr22        -----------------------------------------------
BmorOr17        -----------------------------------------------
BmorOr1         -----------------------------------------------
BmorOr1a        -----------------------------------------------
BmorOr1b        -----------------------------------------------
```

```
BmorOr19        -----------------------------------------------
BmorOr15        -----------------------------------------------
TcasOr5         -----------------------------------------------
TcasOr7         -----------------------------------------------
HvirOr4         -----------------------------------------------
BmorOr13        -----------------------------------------------
DmelOr10a       -----------------------------------------------
DmelOr33c       -----------------------------------------------
DmelOr7a        -----------------------------------------------
DmelOr22c       -----------------------------------------------
AgamOr1         -----------------------------------------------
AgamOr4         -----------------------------------------------
DmelOr47b       -----------------------------------------------
AmelOr73        -----------------------------------------------
AgamOr68        -----------------------------------------------
DmelOr59a       -----------------------------------------------
DmelOr23a       -----------------------------------------------
AgamOr31        -----------------------------------------------
DmelOr2a        -----------------------------------------------
AmelOr62        -----------------------------------------------
AmelOr121       -----------------------------------------------
AmelOr140       -----------------------------------------------
TcasOr12        -----------------------------------------------
AgamOr8         -----------------------------------------------
BmorOr9         -----------------------------------------------
AgamOr40        -----------------------------------------------
TcasOr63        -----------------------------------------------
TcasOr4         -----------------------------------------------
TcasOr13        -----------------------------------------------
AgamOr24        -----------------------------------------------
AgamOr54        -----------------------------------------------
AgamOr23        -----------------------------------------------
AgamOr64        -----------------------------------------------
DmelOr82a       -----------------------------------------------
AmelOr116       -----------------------------------------------
AmelOr160       -----------------------------------------------
TcasOr10        -----------------------------------------------
TcasOr26        -----------------------------------------------
AmelOr117       -----------------------------------------------
AmelOr120       -----------------------------------------------
BmorOr7         -----------------------------------------------
AgamOr6         -----------------------------------------------
DmelOr45a       -----------------------------------------------
DmelOr67d       -----------------------------------------------
DmelOr83c       -----------------------------------------------
AmelOr162       -----------------------------------------------
HvirOr3         -----------------------------------------------
TcasOr20        -----------------------------------------------
AgamOr11        -----------------------------------------------
DmelOr56a       -----------------------------------------------
AgamOr28        -----------------------------------------------
AgamOr58        -----------------------------------------------
AgamOr59        -----------------------------------------------
DmelOr1a        -----------------------------------------------
DmelOr13a       -----------------------------------------------
AmelOr161       -----------------------------------------------
AmelOr141       -----------------------------------------------
AgamOr52        -----------------------------------------------
AgamOr36        -----------------------------------------------
AgamOr25        -----------------------------------------------
AgamOr45        -----------------------------------------------
TcasOr3         -----------------------------------------------
DmelOr88a       -----------------------------------------------
AgamOr22        -----------------------------------------------
DmelOr49a       -----------------------------------------------
DmelOr85f       -----------------------------------------------
AmelOr119       -----------------------------------------------
DmelOr63a       -----------------------------------------------
DmelOr67b       -----------------------------------------------
BmorOr21        -----------------------------------------------
```

```
BmorOr11        -----------------------------------------------
BmorOr12        -----------------------------------------------
AgamOr33        -----------------------------------------------
HvirOr1         -----------------------------------------------
HvirOr5         -----------------------------------------------
TcasOr1         -----------------------------------------------
TcasOr2         -----------------------------------------------
```

Figure S1.
