# Supplementary material for: A Family of Chemoreceptors in Tribolium castaneum (Tenebrionidae: Coleoptera)
Source: PLoS One. 2007 Dec 19;2(12):e1319. doi: 10.1371/journal.pone.0001319 (PMC2121604; doi:10.1371/journal.pone.0001319)
Supplement: Figure S2 — The signature motif of the Gr receptors in insect species. Sequence alignments of the Gr gene families of T. castaneum, D. melanogaster, A. gambiae, A. mellifera, and A. aegypti revealed a common amino acid motif in the Gr sequences. The Gr sequences are marked to the left with their proposed gene name. The average similarity in the sequence motif was more than 50%. Alignment of the amino acid sequence was analyzed using CLC Free Workbench 3.2.2. software. The consensus alignment and the colouring of the conserved residues was asigned using ClustalX. (0.88 MB PDF) [file pone.0001319.s004.pdf]

TcasGr10	HEIIDFSLQILNERAEFNAAGFFPIDYTLVFSV	33
TcasGr2	LFNGLKALIFQISNLQTTFGGTFGDTFIILLSM	33
TcasGr38	HDIIDFSLQIANEQVEFNAAGFFPINYTLVFSV	33
TcasGr5	HFTAA-RFFDLNRKTILGVLNALFTFLIVVVQF	32
TCasGr6	PKVSLKGYTVVNRELVTASVATIAIYLIVLLQF	33
TcasGr7	VAFTGKNFFSITRGLILSIAGAIVSYELVLMQF	33
TcasGr104	VHITA-NYFEINKATLCGIFGTTTTYFIVILQF	32
TcasGr1	PIMNLNGYANVNRKLISSTVTSIATYLVMLMQF	33
TcasGr12	FPVFSCDYFTIETKTVLDVLGVTVTYLIVIIQF	33
TcasGr13	FPVFSCDYFTIETKTVLDVLGVTVTYLIVIIQF	33
TcasGr14	LQFTAKDFFNLDWTFCHMMIAALTTYLVILIQF	33
TcasGr15	RKPSFVIQFSHIPVDNIEFAGALVTYELTLFQY	33
TcasGr16	LEITACKFFSIDNALLLSICGASSSYLFIMIQL	33
TcasGr150	IEITACRLFNIDNALLFAICGSAFSYLFIMLQF	33
TcasGr19	THFYCFSLYPLKSTFYLNVVRPFPERSMGITPM	33
TcasGr20	IKFSSFGMLKISRSLLTSFGGALTTFLVILFQY	33
TcasGr21	ACEVM-AIVWITSETCEEIVAGVTTYLVILIQF	32
TcasGr22	LEITACRLFSIDNALLFSICGAASSYLFIMLQL	33
TcasGr79	PTFSA--FYGIDFSTFLGLVGAVVTFLIVLLQF	31
TcasGr123	VEFNAAGFFAINYTLVFSILGGVTTYIIILIQL	33
TcasGr25	ARKNAGRAEPVFDGKVPSNGSLNQISDIDDVNF	33
TcasGr26	PKVSLKGYTVVNRELVTASVATIAIYLIVLLQF	33
TcasGr27	PIVFYVAIFSLDLDTFKKIMQKAWSFFTALKNM	33
TcasGr28	TLYSG-DEMYFR--VAFPSVFTLIDYSLWKACF	30
TcasGr29	VAFTGKNFFSITRGLILSIAGAIVSYELVLMQF	33
TcasGr30	VAFTGKNFFSITRGLILSIAGAIVSYELVLMQF	33
TcasGr32	VAHTAARFYAINRSTIFRMFNAIVTFLIVMVQF	33
TcasGr47	VKITAADFFILDKSTILKVLDTVVAFLMVVAQF	33
TcasGr34	PKFTAGGFFHVKKSTIFSILNTVSTLLIVMVQF	33
TcasGr35	PEFKA--FFSIDRSTLFSVLNSLTTFLLVMIQF	31
TcasGr105	AQITAANFFDISRSTFLGILATITTYFIVIIEF	33
TcasGr37	PVFAAARFYAINRSTIFRMFNAIVTFLIVMVQF	33
TcasGr3	TDISLVGFFDVNRNLFKSLLATMVTYLVVLLQF	33
TcasGr39	PIMNLNGYANVNRKLISSTVTSIATYLVMLMQF	33
TcasGr40	TCSQI--PSEVEDK-YTTYFLNTYPHLFNFFEF	30
TcasGr41	VAFKV-GFLNIDYGTLYSLFGSTAMNVIILLQF	32
TcasGr71	PDFSAAGFFSINKTTLLQIIGNVTTFFIIIIQF	33
TcasGr43	PKFSAANFFDIERSTILSVLGTACTFLIIIVQF	33
TcasGr45	VHITA-NYFEINKATLCGIFGTTTTYFIVILQF	32
TcasGr46	HEIIDAGFFPIDYTLVFSKFTSSIRMLLIQGQI	33
TcasGr33	PKVSLKGYTVVNRELVTASVATIAIYLIVLLQF	33
TcasGr48	VVFTAEHFFSVKCSILFPVAGFISDFDCDINSI	33
TcasGr49	LQFTAKDFFNLDWTFCHMVSHKWLNYINQFEAN	33
TcasGr50	YSVNKLIFRSNDPKVTLELCGMISTYLIIMIQL	33
TcasGr51	RTFSA-GFFDIRKNLIFSLIANVAMYFVISVQF	32
TcasGr54	PRFYAARFFVINRGTILGILDAIVTFLIVMIQF	33
TcasGr62	VEFNAAGFFAINYTLVFSILGGVTTYIIILIQL	33
TcasGr52	AKFTAADFFEIDRGTFFGILSTTTSYFIIMIQF	33
TcasGr125	VQFSVLGFFVVDYTLLYSIVGAVTTYLVIFIQF	33
TcasGr57	TSRTSAGFFPVDFTLLGFIFGSVTSYIIISIQF	33
TcasGr98	ANFTAAGFFDVKRSTLFGILATTTTYLIVTIQF	33
TcasGr59	ARKNAGRAEPVFDGKVPSNGSLNQISDIDDVNF	33
TcasGr60	TTCDIATILDFWVRCVLSLHCAAVGGSANIMSG	33
TcasGr61	PTFTALGLFPINGSFAFTVVGAATTYITIIYQF	33
TcasGr53	PVFAAARFYAINRSTIFRMFNAIVTFLI-----	28
AgamGr1	PKFRI-KSINLSFNLIYASCGVIATYLTVFLQF	32
AgamGr2	LNFSACGFFDLDMTTLYAITGAITSYLIILIQF	33
AgamGr3	IKLTAKDIFDYDYTLIRTLVIVILTYVIIFIEI	33
AgamGr4	IRLTAHGLFEINYSLLKMFGTGITTYMIIFITF	33
AgamGr5	IQLTA-DFFDLNFSPILTIIFSVGAYISIFIQQ	32
AgamGr6	INFSAANFFDIRLSSLTTIFGSITSYLVIYINF	33
AgamGr7	MSFSL-GFFDMDFVLLKEIAAAITTYMVIFIQF	32
AgamGr8	PFTMTLSMYRLDYGTLMQVLKIVTTYKILFDCY	33
AgamGr9a	KKFSACGFFDIDNTVIYMVFSSIVTYLVILIQF	33
AgamGr9b	KKFSACGFFDIDNTVIYMVFSSIVTYLVILIQF	33
AgamGr9c	KKFSACGFFDIDNTVIYMVFSSIVTYLVILIQF	33
AgamGr9d	KKFSACGFFDIDNTVIYMVFSSIVTYLVILIQF	33
AgamGr9e	KKFSACGFFDIDNTVIYMVFSSIVTYLVILIQF	33
AgamGr9f	KKFSACGFFDIDNTVIYMVFSSIVTYLVILIQF	33
AgamGr9g	KKFSACGFFDIDNTVIYMVFSSIVTYLVILIQF	33
AgamGr9h	KKFSACGFFDIDNTVIYMVFSSIVTYLVILIQF	33
AgamGr9i	KKFSACGFFDIDNTVIYMVFSSIVTYLVILIQF	33
AgamGr9j	KKFSACGFFDIDNTVIYMVFSSIVTYLVILIQF	33
AgamGr9k	KKFSACGFFDIDNTVIYMVFSSIVTYLVILIQF	33
AgamGr9l	KKFSACGFFDIDNTVIYMVFSSIVTYLVILIQF	33
AgamGr9m	KKFSACGFFDIDNTVIYMVFSSIVTYLVILIQF	33
AgamGr9n	KKFSACGFFDIDNTVIYMVFSSIVTYLVILIQF	33
AgamGr10	KKFSACGFFDIDNTVIYMVFSSIVTYLVILIQF	33
AgamGr11	INFSVCGMFDIDYAMIHMVLSSIFTYLVILVQF	33
AgamGr12	NQQRF-GLFVLDNKLVCTALTSMITYLVILIQF	32
AgamGr13	LKINI-GMFTMDFELLTGMLAAISNFVVLLMQF	32
AgamGr15	VALTGLKFFSMTRQLVLNVTGAIITYELVLIQF	33
AgamGr16	VALNGYGFFYLTRKLILKIAATVVTYELVLTQV	33
AgamGr17	NCLSGHGFFFLNRSVILAMAGTLITYELVMLKE	33
AgamGr18	NVLSGKQFFFLKRQLILAMAGTLVTYELVLLDQ	33
AgamGr19	IAINAMGLFRLTKKTMLTMLGAVITYELVMLHF	33
AgamGr20	VSISGMGFFTITRRIFLTMAGSILTYELVLMRF	33
AgamGr21	VALSGMGFFSLTRQLLFSMAGTIVTYELVMLKF	33
AgamGr22	PTMNLDGYANINRGLITSNISFMATYLVVLMQF	33
AgamGr23	AIVSLKGYAEVNRELLTSSIATIAIYLIVLLQF	33
AgamGr24	SSINLGGFFDVNRTLFKSLLATMVTYLVVLLQF	33
AgamGr25	FSFTACGLCTIDRRIITSYCGAIATYLVILIQF	33
AgamGr26	GPQTACGVINLEMTLISTMVGALTTYLVILIQF	33
AgamGr27	GKRHHCGLITLDLTLISKIVAGLTTYLMILIQF	33
AgamGr28	KRIVPCGLLKLELSSLSSIFVALLSFMIILIQF	33
AgamGr29	TLFLEYGMISIDMTLILSNVGGLTNILVVLVQF	33
AgamGr30	DTHQACGMIKLDMQLVPNVIAVITSILFILMQF	33
AgamGr31	TLFLAYSMISIDMTLILSIVAGLTNILVVLVQF	33
AgamGr32b	NQKAL-MLYRIKRICSVELNEAIEHFISQISNL	32
AgamGr33	LHRKVACGLFSLDFSLIFTLVGAATTYLVILIQ	33
AgamGr34	KVFKFAMGFFQIDCNMLCGMIGAITTYLVIYIQ	33
AgamGr35	VRFSAFGFFTINYNMLSGLVAGMVTYLIIFIQF	33
AgamGr36	PKLRCIFYAYDWKTIYNMFGFVVTYLIILLQFD	33
AgamGr37a	PVITCGLFVYDWTLWYTMIGATATYLIILIQFD	33
AgamGr37b	PVITCGLFVYDWTLWYTMIGATATYLIILIQFD	33
AgamGr37c	PVITCGLFVYDWTLWYTMIGATATYLIILIQFD	33
AgamGr37d	PVITCGLFVYDWTLWYTMIGATATYLIILIQFD	33
AgamGr37e	PVITCGLFVYDWTLWYTMIGATATYLIILIQFD	33
AgamGr37f	PVITCGLFVYDWTLWYTMIGATATYLIILIQFD	33
AgamGr38	PMITCGLFPFDWTLIFSILSTAATYIIILLQFE	33
AgamGr39	PKASC-FYDIDWTFLLSMISSFATYLNILVQFD	32
AgamGr40	PRVSCLLFDFEWPFLVSVAATLVMHVVILVQFD	33

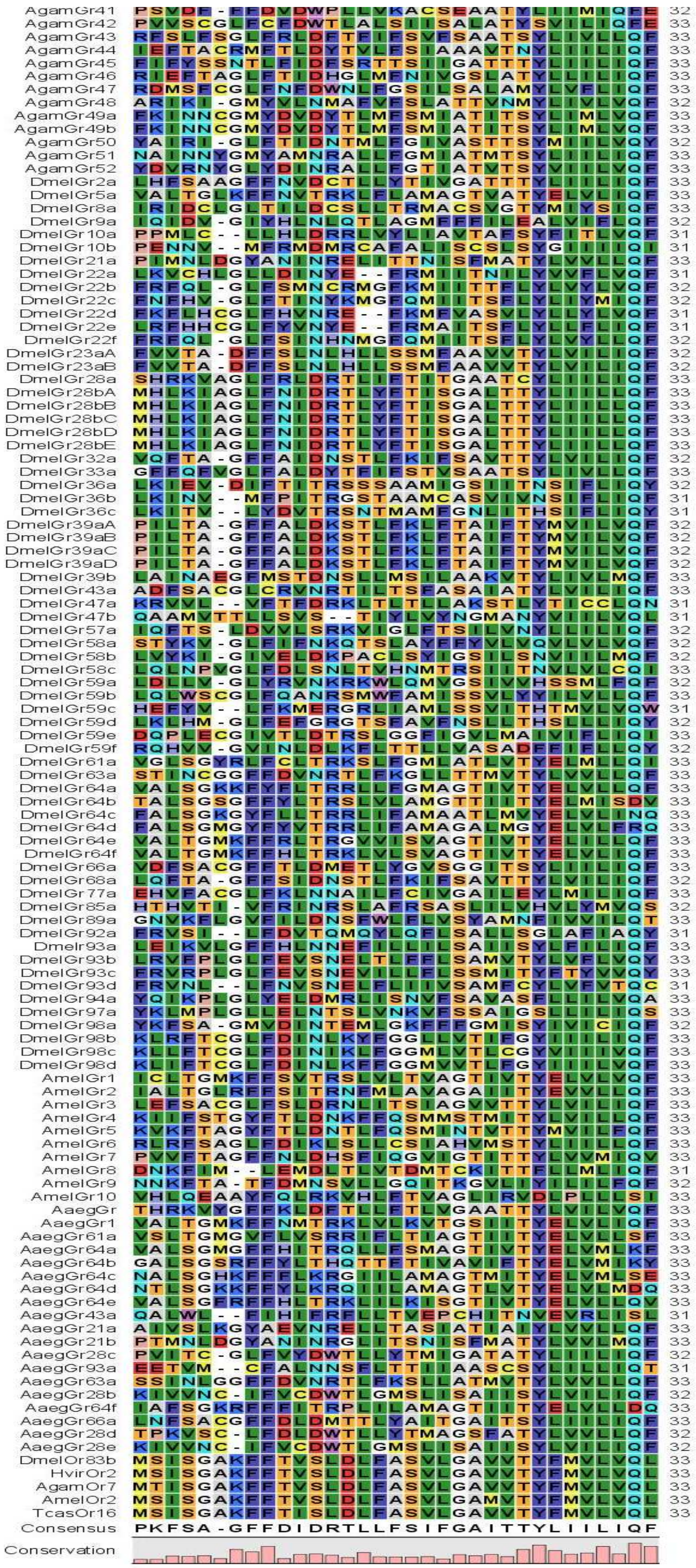
AgamGr41
AgamGr42
AgamGr43
AgamGr44
AgamGr45
AgamGr46
AgamGr47
AgamGr48
AgamGr49a
AgamGr49b
AgamGr50
AgamGr51
AgamGr52
DmelGr2a
DmelGr5a
DmelGr8a
DmelGr9a
DmelGr10a
DmelGr10b
DmelGr21a
DmelGr22a
DmelGr22b
DmelGr22c
DmelGr22d
DmelGr22e
DmelGr22f
DmelGr23aA
DmelGr23aB
DmelGr28a
DmelGr28bA
DmelGr28bB
DmelGr28bC
DmelGr28bD
DmelGr28bE
DmelGr32a
DmelGr33a
DmelGr36a
DmelGr36b
DmelGr36c
DmelGr39aA
DmelGr39aB
DmelGr39aC
DmelGr39aD
DmelGr39b
DmelGr43a
DmelGr47a
DmelGr47b
DmelGr57a
DmelGr58a
DmelGr58b
DmelGr58c
DmelGr59a
DmelGr59b
DmelGr59c
DmelGr59d
DmelGr59e
DmelGr59f
DmelGr61a
DmelGr63a
DmelGr64a
DmelGr64b
DmelGr64c
DmelGr64d
DmelGr64e
DmelGr64f
DmelGr66a
DmelGr68a
DmelGr77a
DmelGr85a
DmelGr89a
DmelGr92a
DmelIr93a
DmelGr93b
DmelGr93c
DmelGr93d
DmelGr94a
DmelGr97a
DmelGr98a
DmelGr98b
DmelGr98c
DmelGr98d
AmelGr1
AmelGr2
AmelGr3
AmelGr4
AmelGr5
AmelGr6
AmelGr7
AmelGr8
AmelGr9
AmelGr10
AaegGr
AaegGr1
AaegGr61a
AaegGr64a
AaegGr64b
AaegGr64c
AaegGr64d
AaegGr64e
AaegGr43a
AaegGr21a
AaegGr21b
AaegGr28c
AaegGr93a
AaegGr63a
AaegGr28b
AaegGr64f
AaegGr66a
AaegGr28d
AaegGr28e
DmelOr83b
HvirOr2
AgamOr7
AmelOr2
TcasOr16
Consensus
PKFSA-GFFDIDRTLLFSIFGAITTYLIILIQF
Conservation

Figure S2.
