# Supplementary figures and images for: A Family of Chemoreceptors in Tribolium castaneum (Tenebrionidae: Coleoptera)
Source: PLoS One. 2007 Dec 19;2(12):e1319. doi: 10.1371/journal.pone.0001319 (PMC2121604; doi:10.1371/journal.pone.0001319)

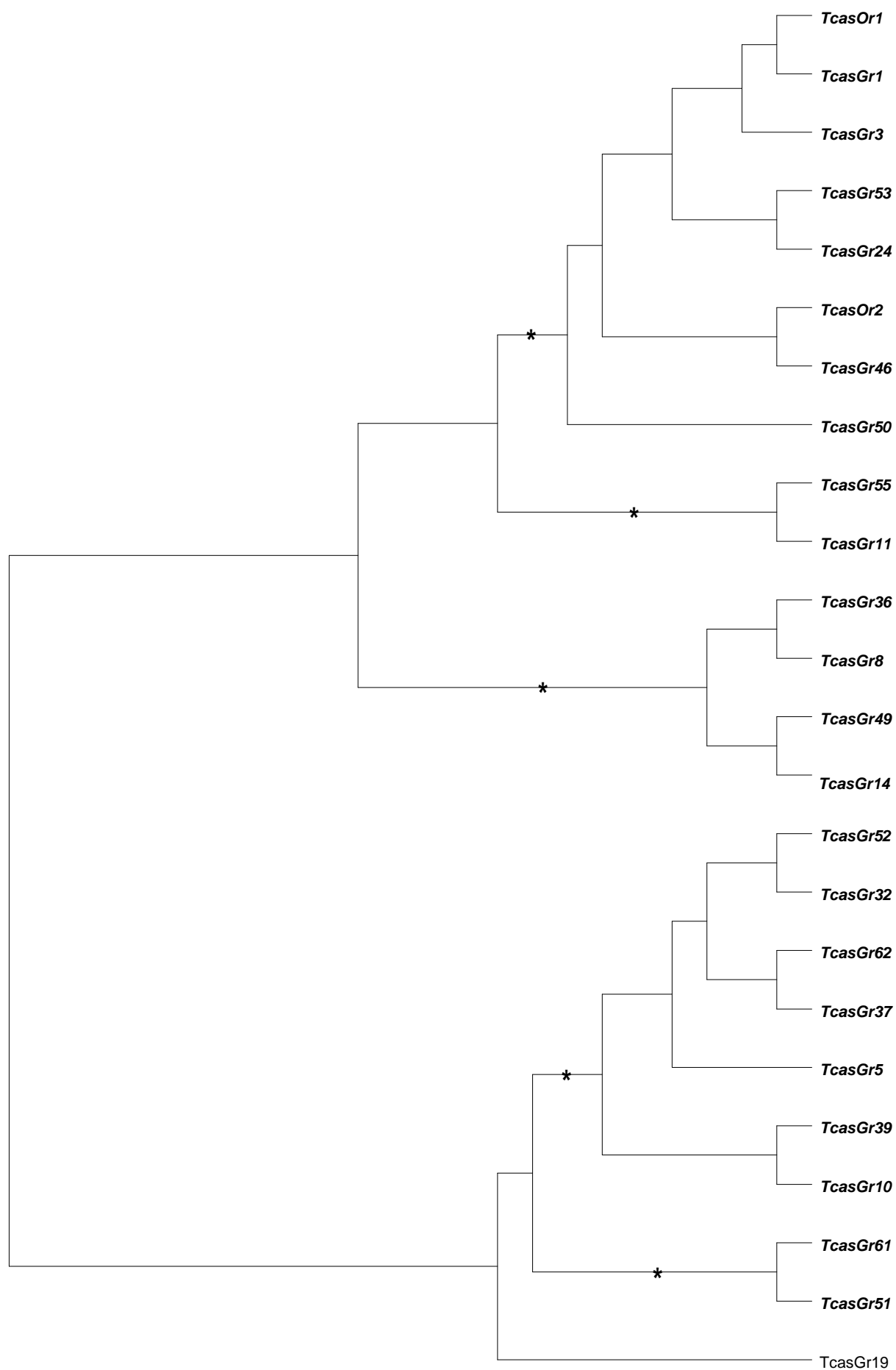

**Figure S3.**

Supplement: Figure S3 — The molecular evolution of 24 chemoreceptor genes localized on chromosome seven of T. castaneum. The proposed gene names for the chemoreceptor are given to the right. The main lineages within the receptors are supported by bootstrap values >50% and are indicated with *. Only TcasGr19 does not belong to any of the orthologs within the tree. For other details see Figure S2. (0.01 MB PDF) [file pone.0001319.s005.pdf]

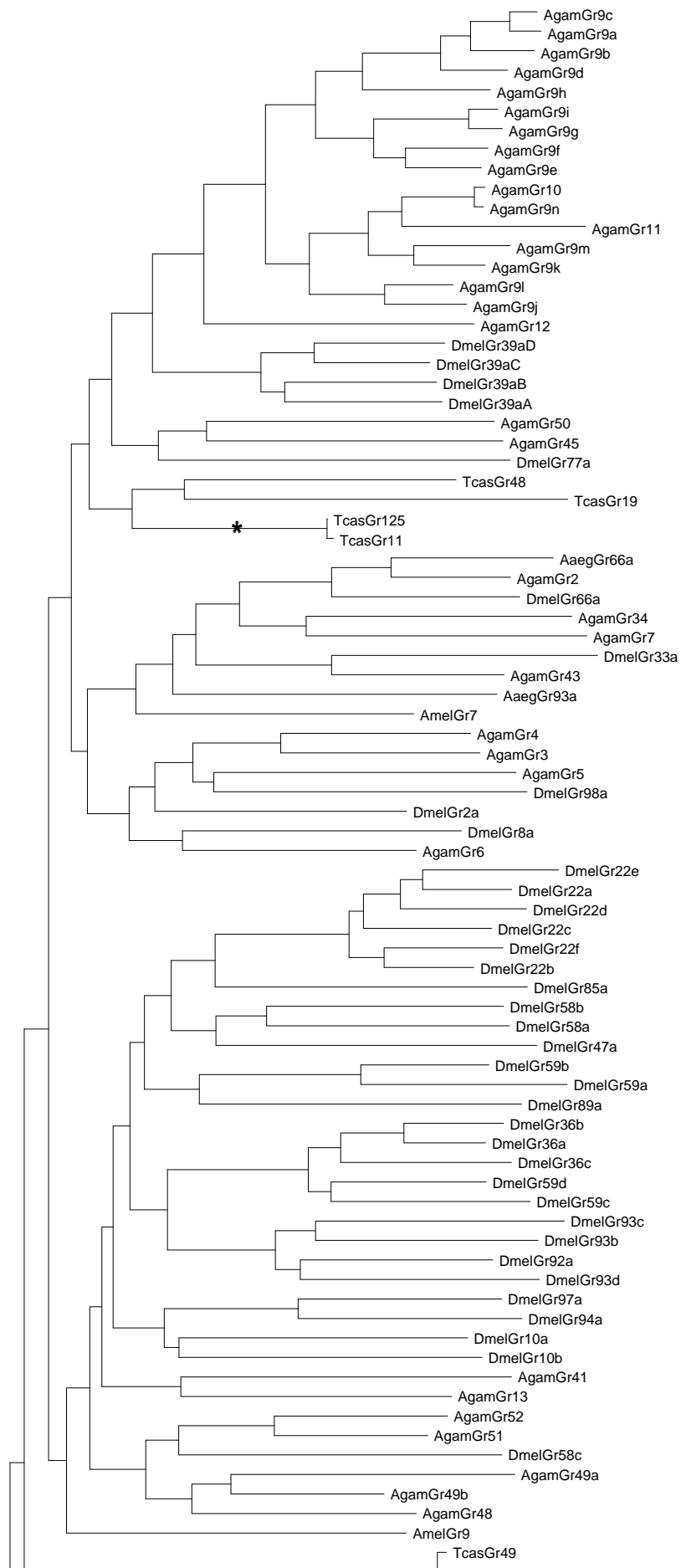

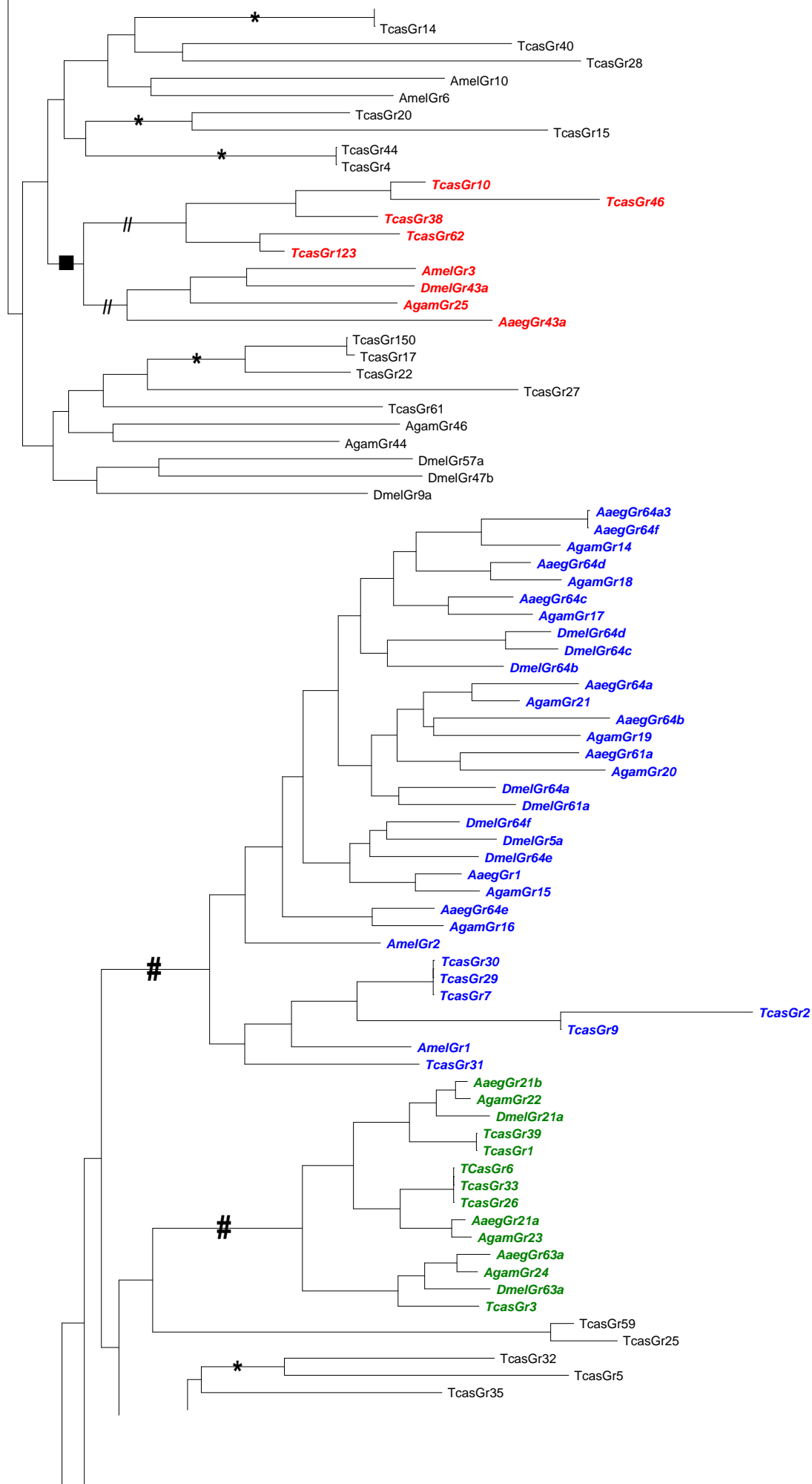

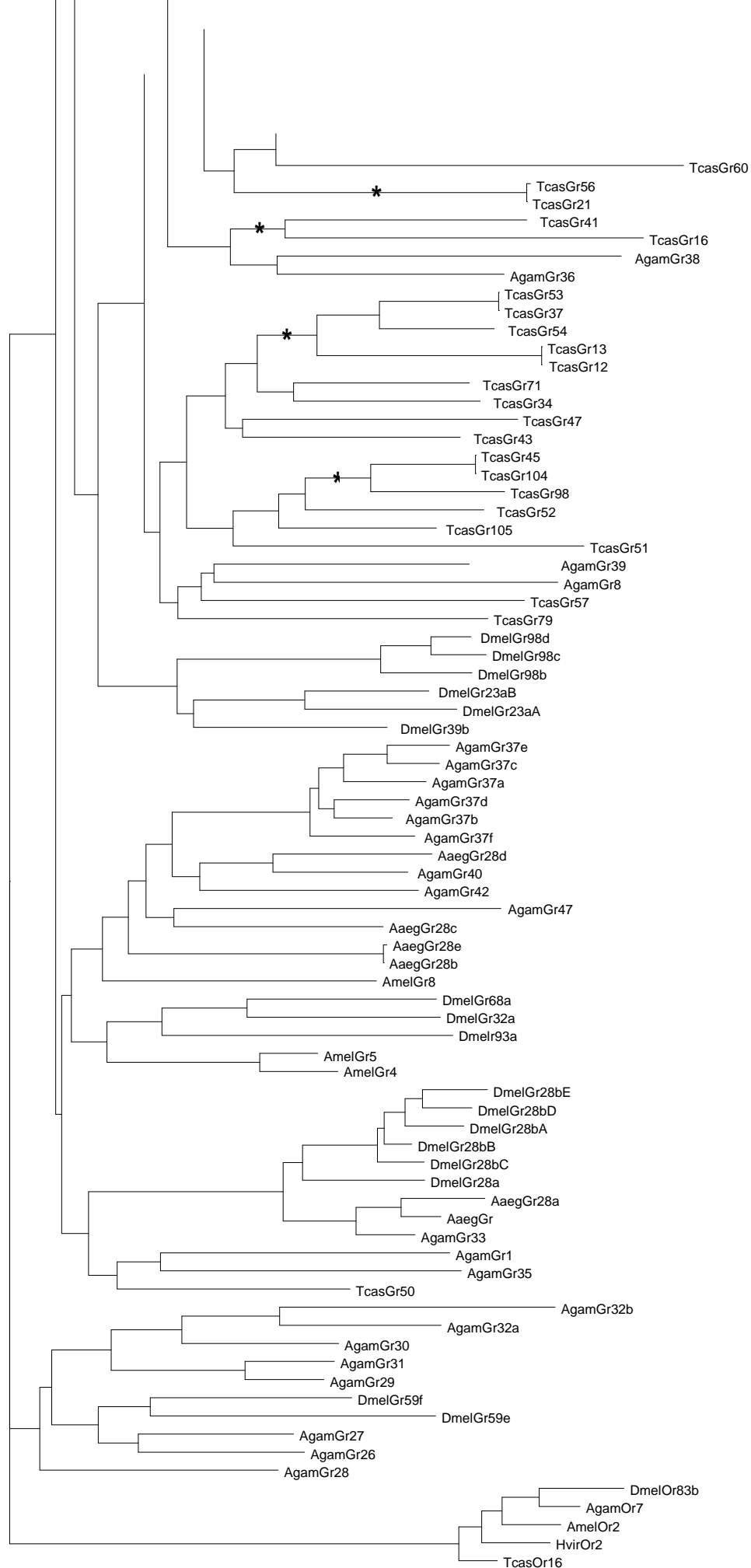

**Figure S4.**

Supplement: Figure S4 — Neighbor joining tree of the insect gustatory receptor superfamily. D. melanogaster (DmelGr), A. gambiae (AgamGr), A. mellifera (AmelGr), A. aegypti (AaegGr) (unpublished data), and T. castaneum Gr receptor gene sequences were used to draw the orthologous relationships between the gustatory receptors of the different insect species. Insect Gr receptors are indicated at the right. Receptors that represent lineages between different insect species are written in bold and italic. The supported bootstrap values (>50%) are denoted with #. The insect sugar receptor subfamily is shown in blue. The insect CO2 receptor subfamily is indicated in green. A weak bootstrap value (<50%) is indicated with a black square, and the appropriate orthologs are written in red. * represents lineages of only T. castaneum Gr receptor orthologs supported with a bootstrap value of >50%. For other details see Figure 1. (0.02 MB PDF) [file pone.0001319.s006.pdf]

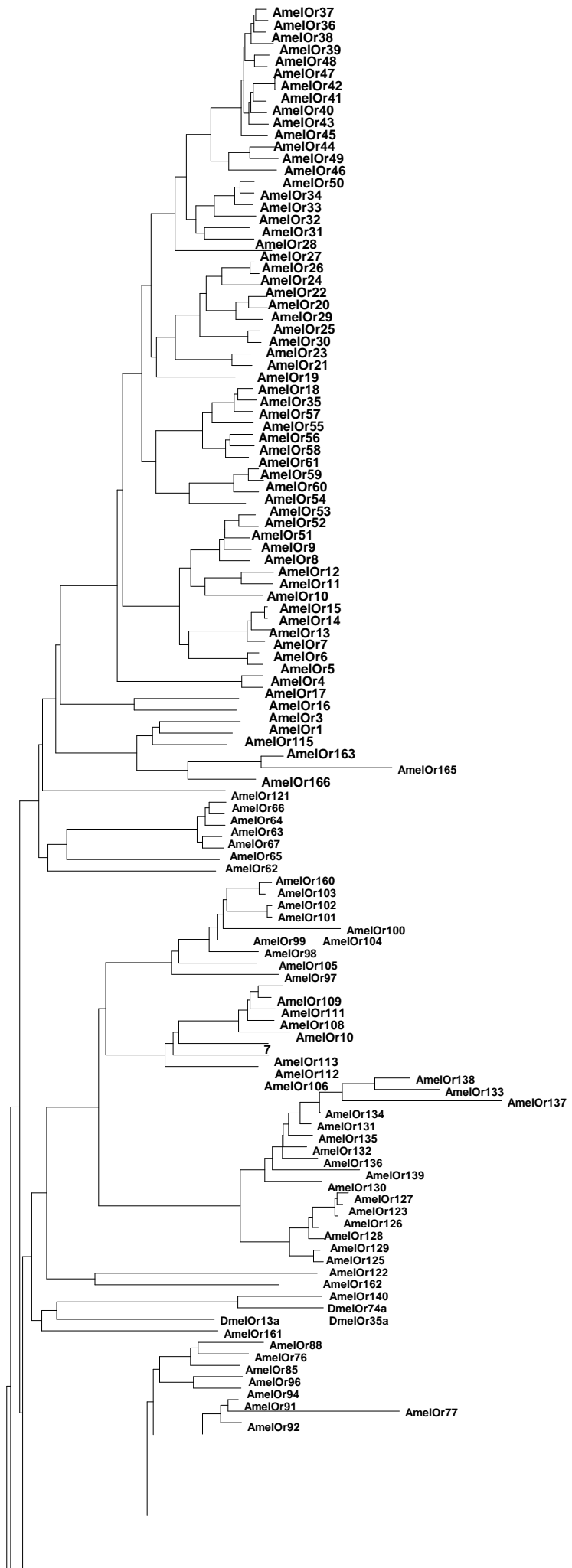

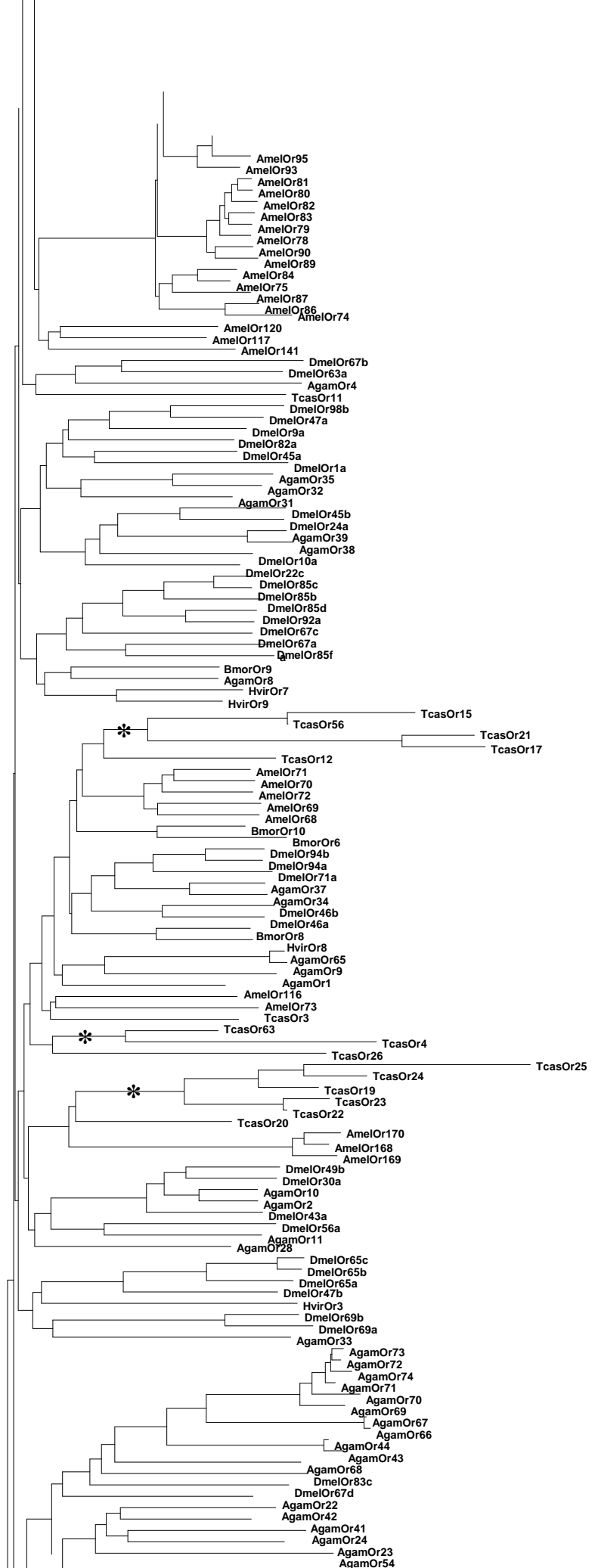

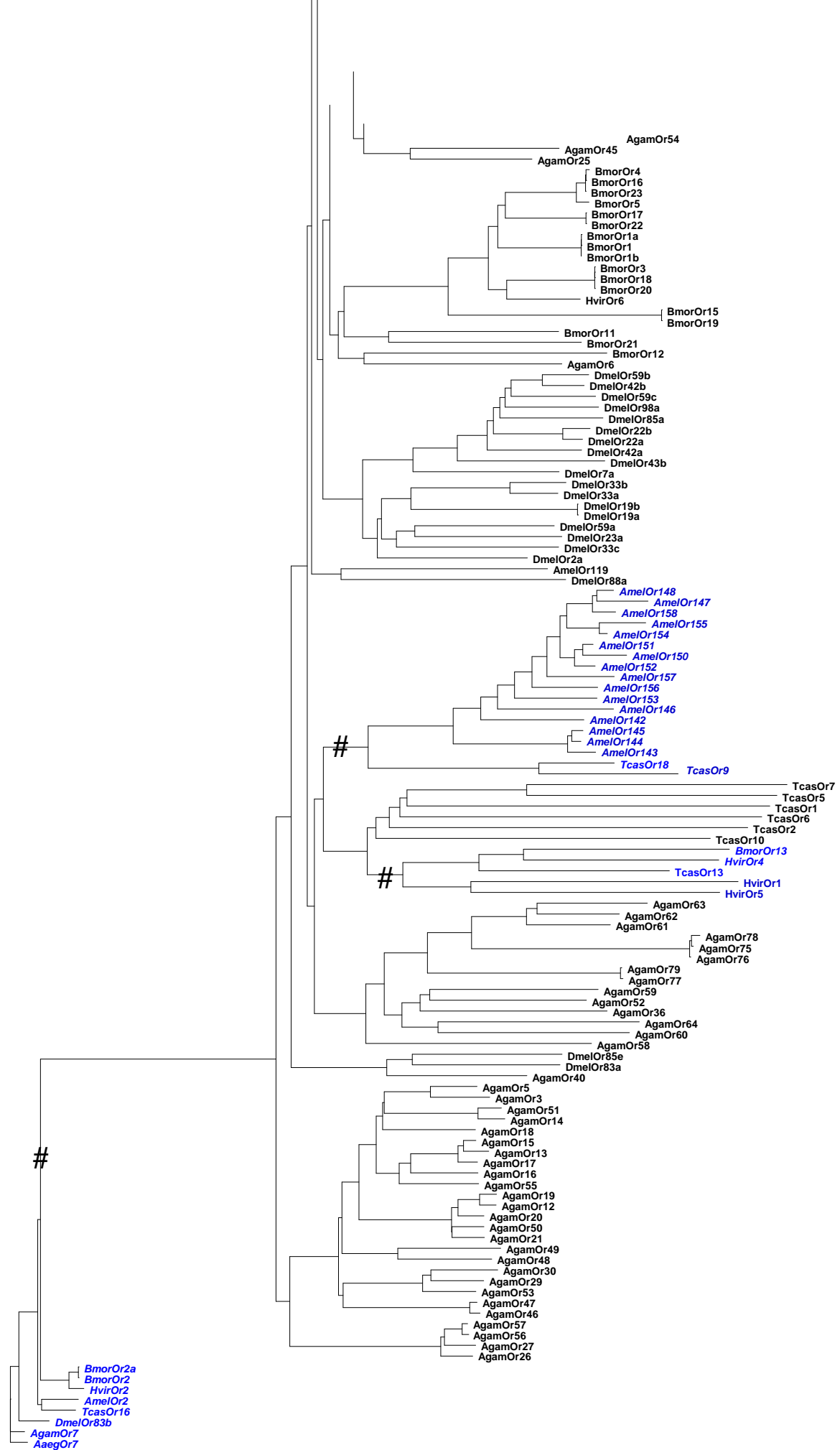

Figure S5.

Supplement: Figure S5 — Neighbor joining tree of the insect olfactory receptor superfamily. Insect olfactory receptor genes (Or) are indicated to the right. The corrected distance tree was rooted by declaring the TcasOr16 and its homologs (DmelOr83b, AmelOr2, AgamOr7, BmorOr2, BmorOr2a, and HvirOr2) as outgroup. Receptors that represent lineages between different insect species are written in blue, and the supported bootstrap values (>50%) are denoted with #. * represents lineages of only T. castaneum Or receptor orthologs supported with a bootstrap value of >50%. For other details see Figure 1. (0.03 MB PDF) [file pone.0001319.s007.pdf]
