# Supplementary material for: A Family of Chemoreceptors in Tribolium castaneum (Tenebrionidae: Coleoptera)
Source: PLoS One. 2007 Dec 19;2(12):e1319. doi: 10.1371/journal.pone.0001319 (PMC2121604; doi:10.1371/journal.pone.0001319)
Supplement: Figure S6 — Tissue specific localization of the T. castaneum Gr receptors. The T. castaneum Gr11-62 were localized in the labium and maxillae of the adult beetles, and in the femur, tibia and tarsus of the larval prolegs. For other details see Figure 5. (2.03 MB PDF) [file pone.0001319.s008.pdf]

*Tcas*Gr11

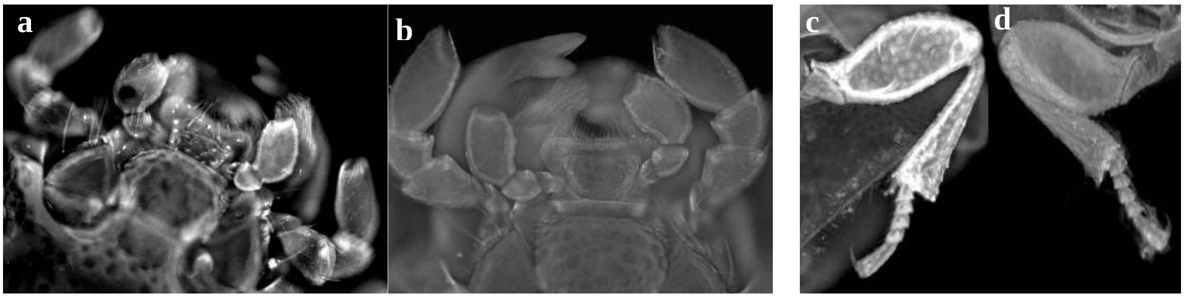

*Tcas*Gr12

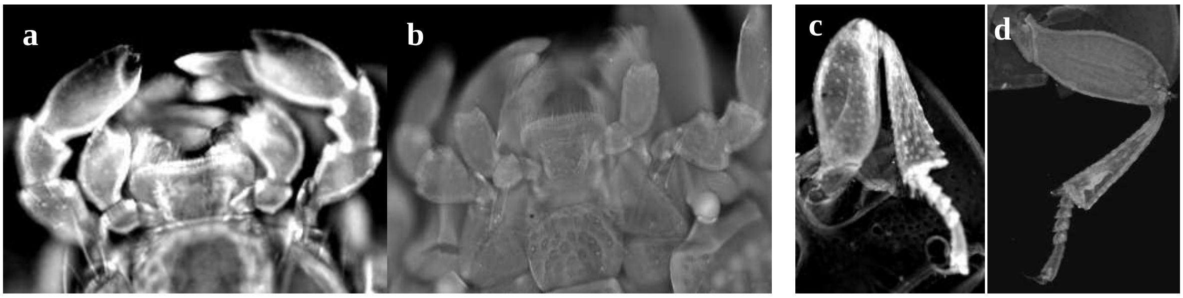

*Tcas*Gr13

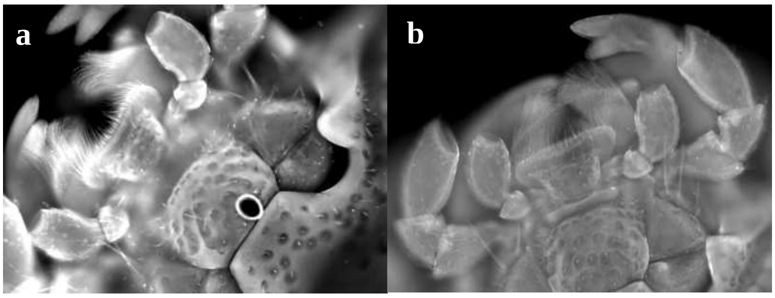

*Tcas*Gr14

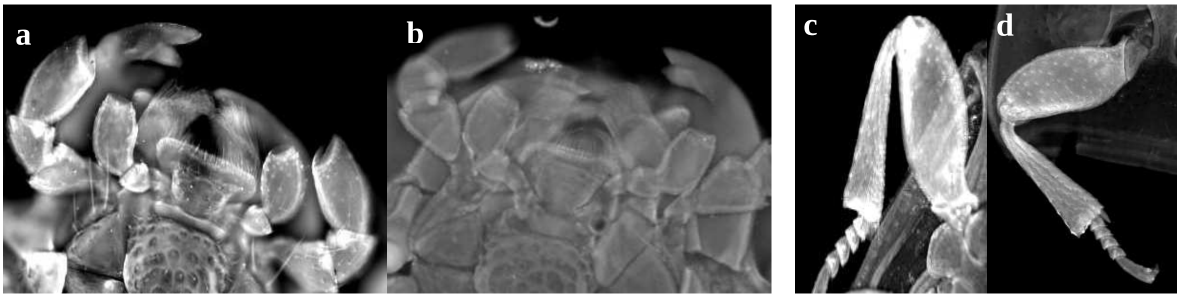

*Tcas*Gr15

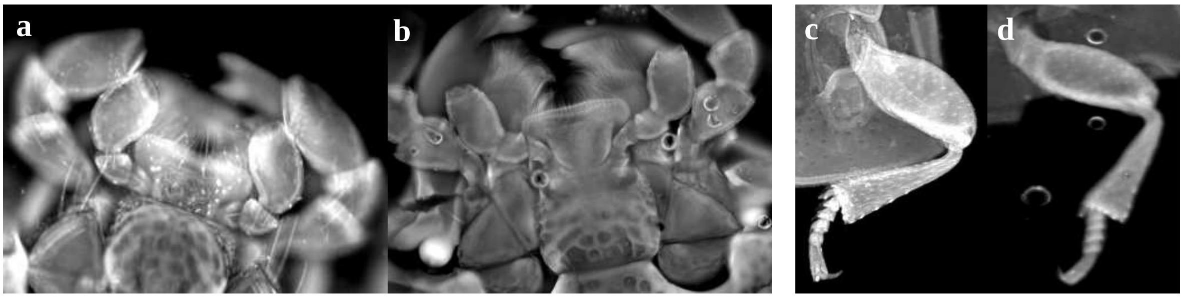

***Tcas*Gr16**

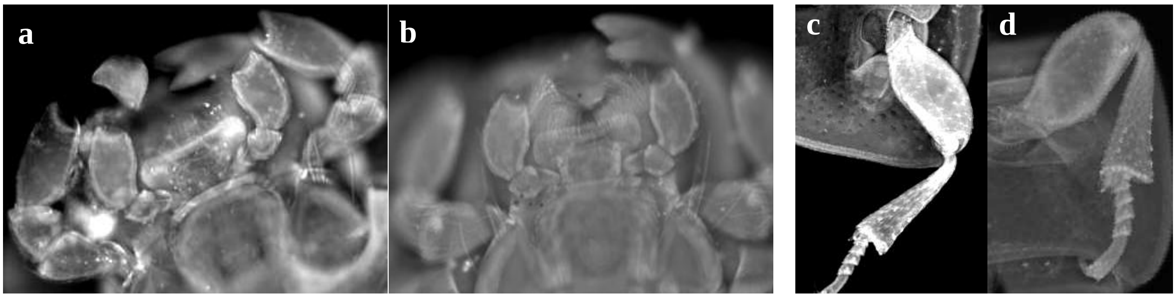

***Tcas*Gr17**

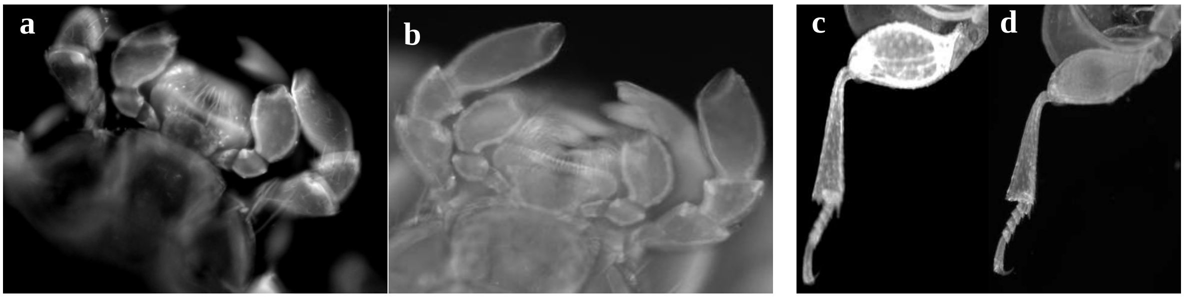

***Tcas*Gr150**

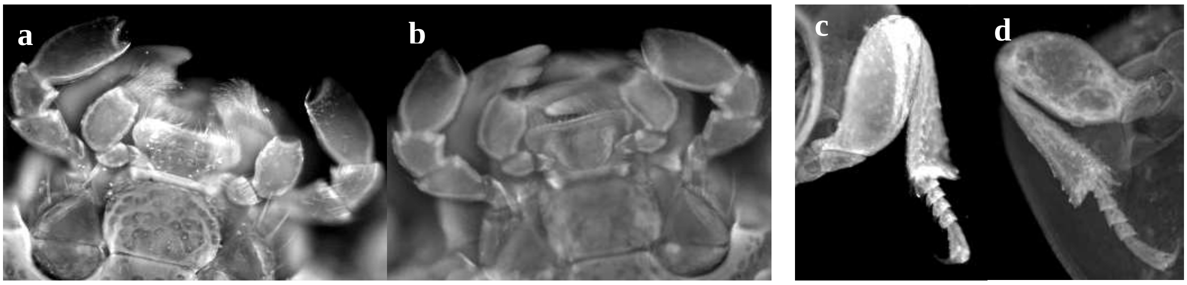

***Tcas*Gr19**

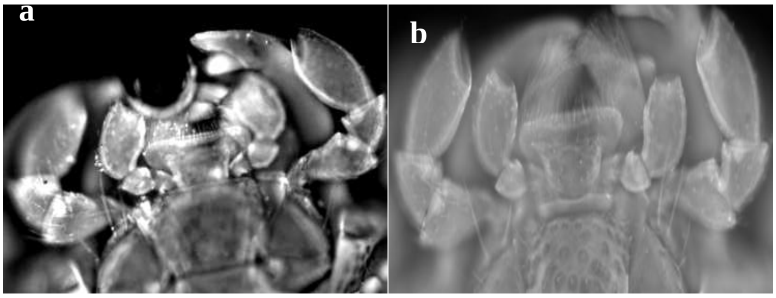

***Tcas*Gr20**

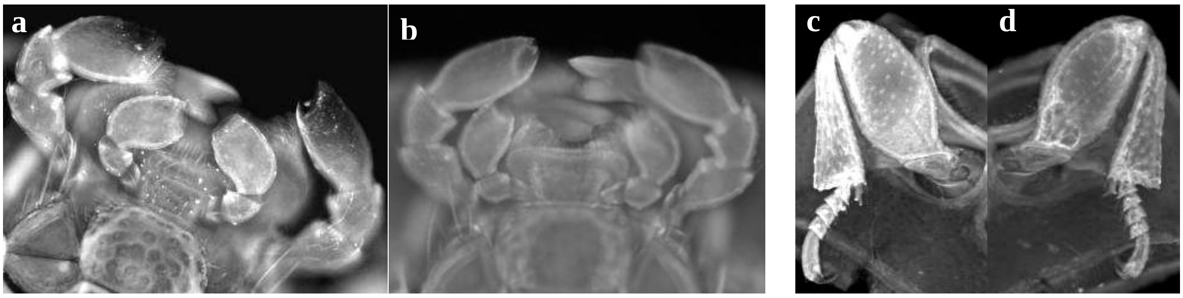

*Tcas*Gr21

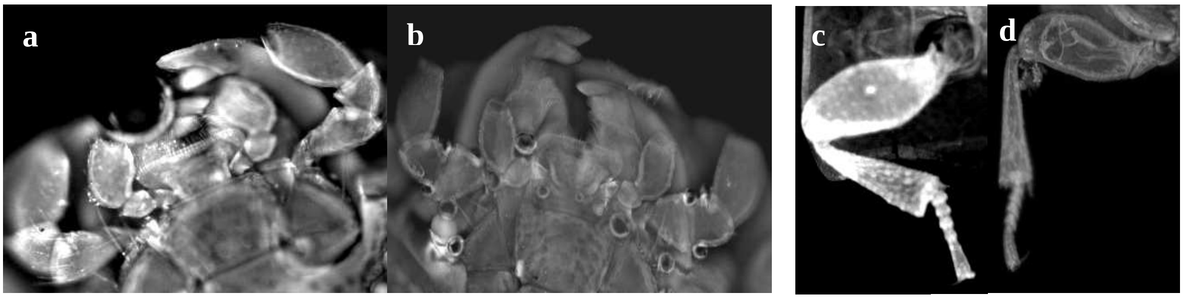

*Tcas*Gr22

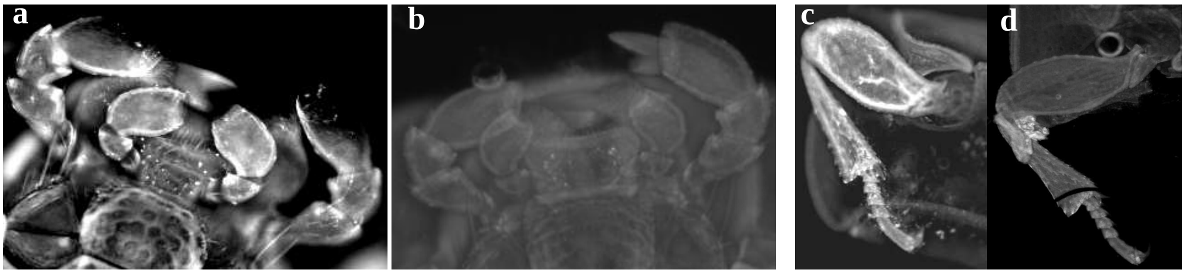

*Tcas*Gr79

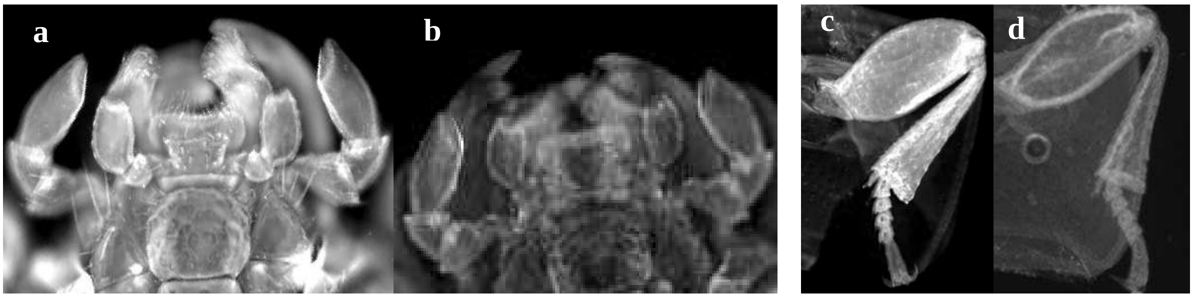

*Tcas*Gr123

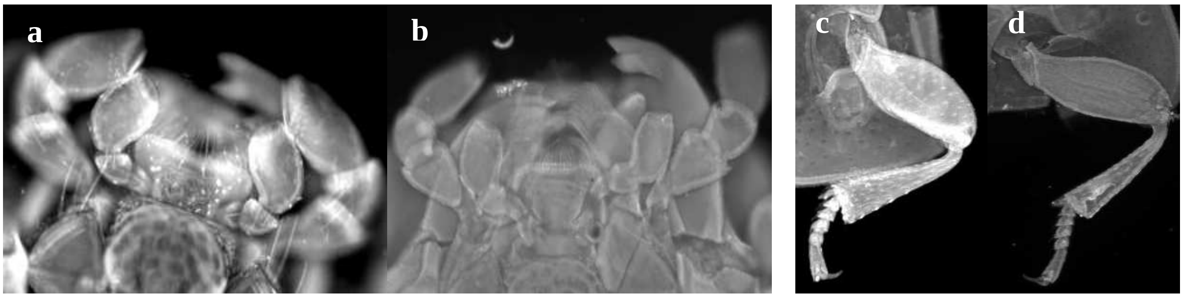

*Tcas*Gr25

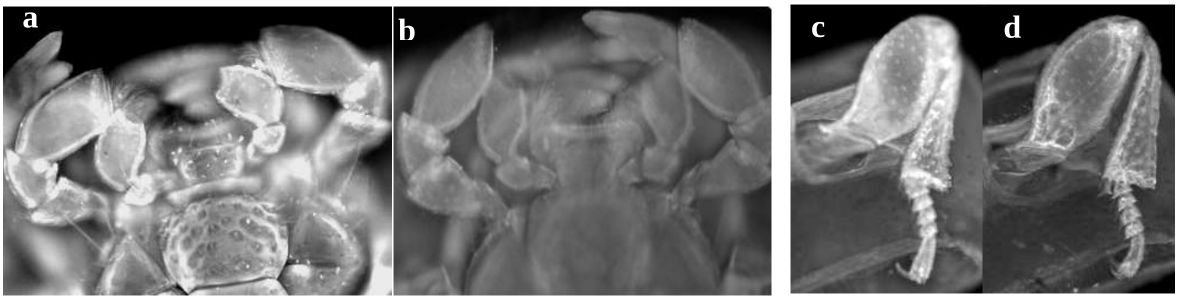

***Tcas*Gr26**

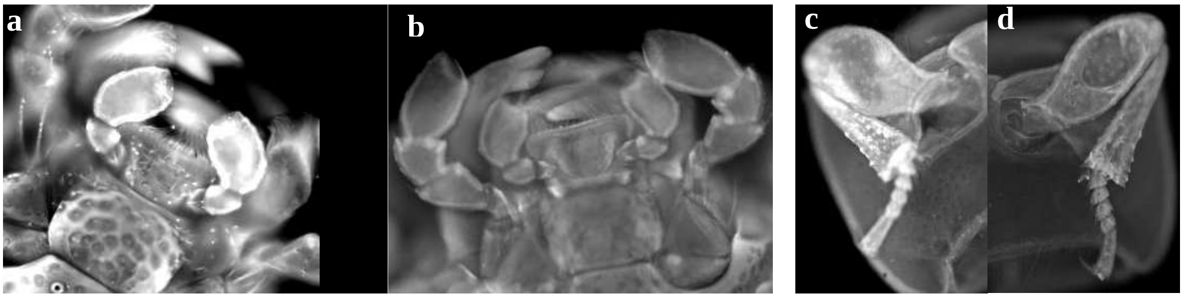

***Tcas*Gr27**

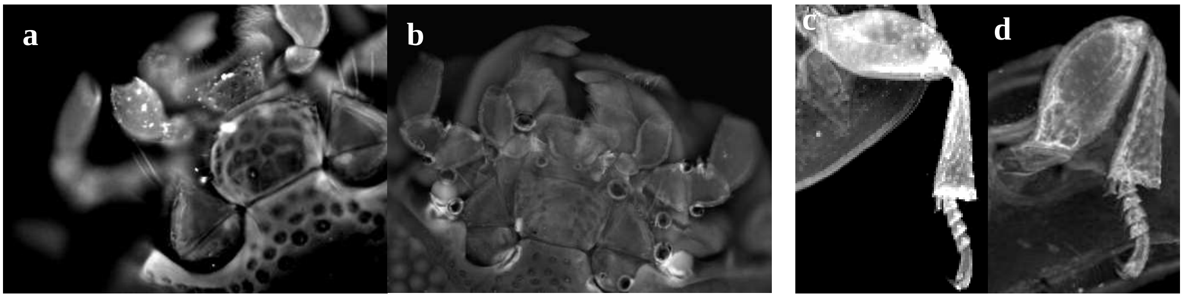

***Tcas*Gr28**

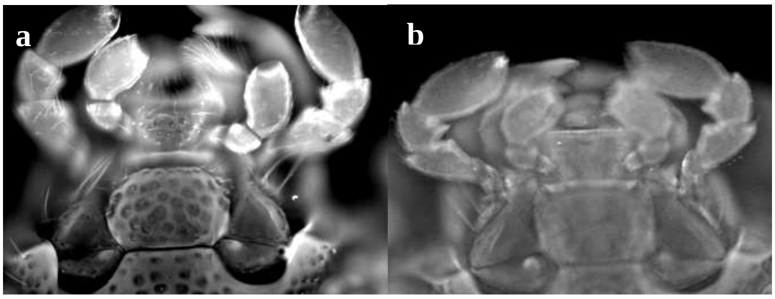

***Tcas*Gr29**

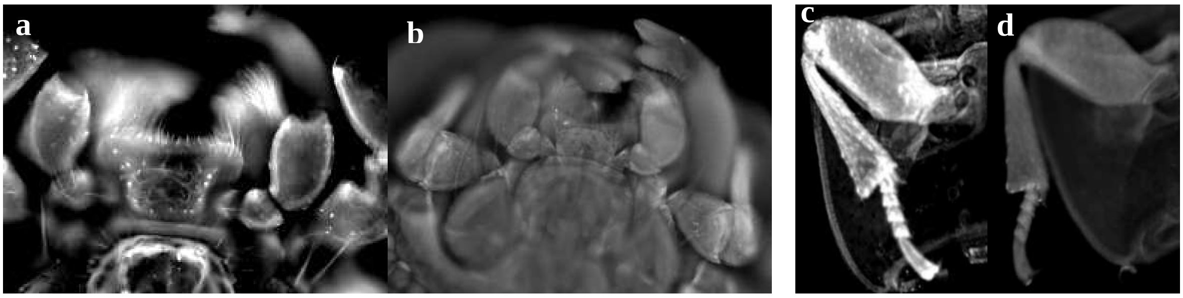

***Tcas*Gr30**

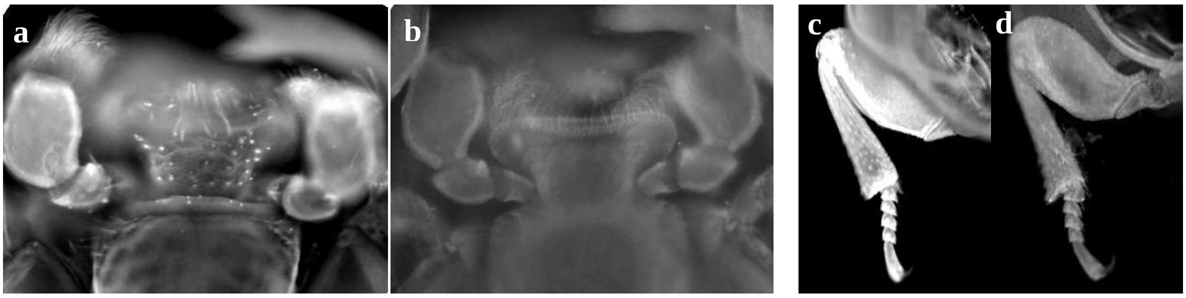

*Tcas*Gr31

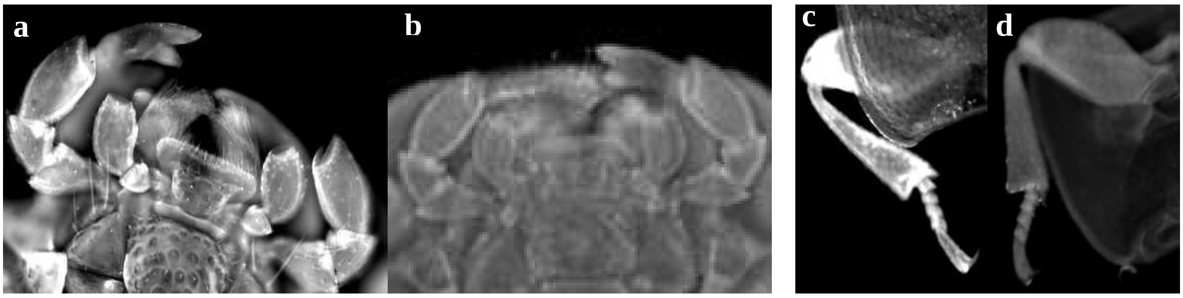

*Tcas*Gr32

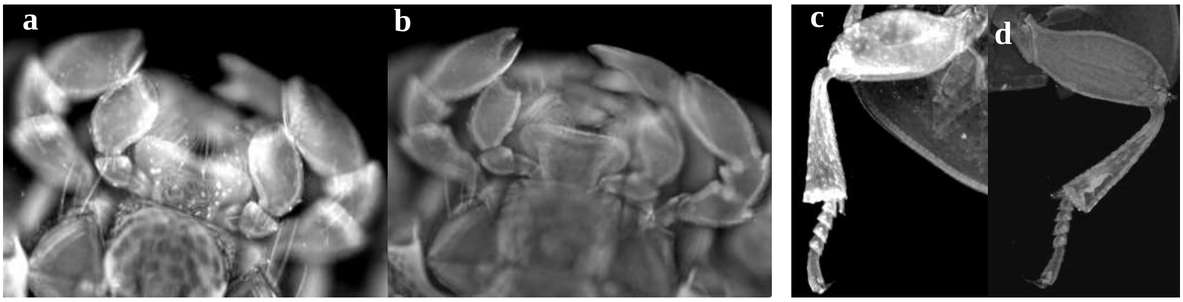

*Tcas*Gr47

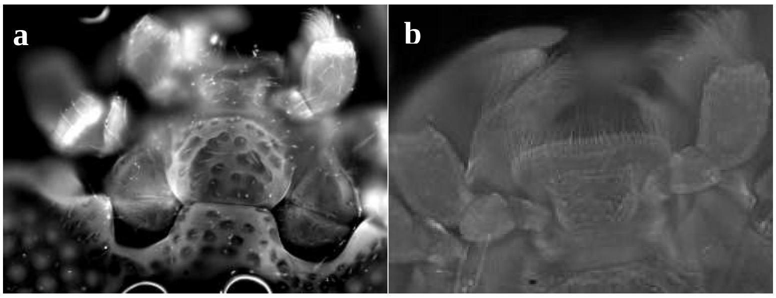

*Tcas*Gr34

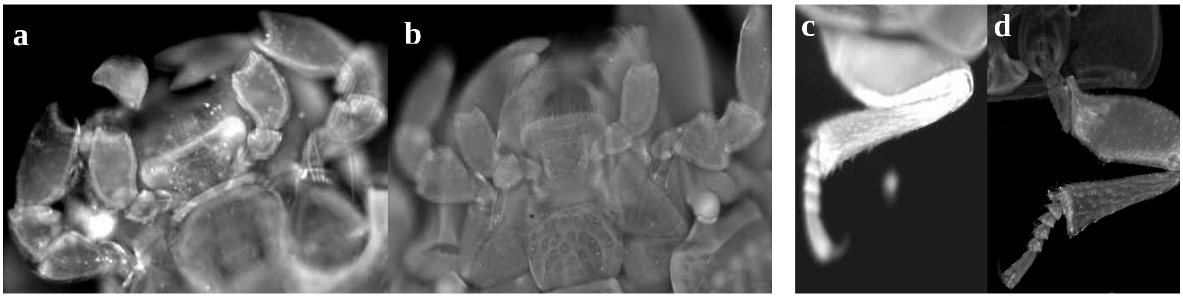

*Tcas*Gr35

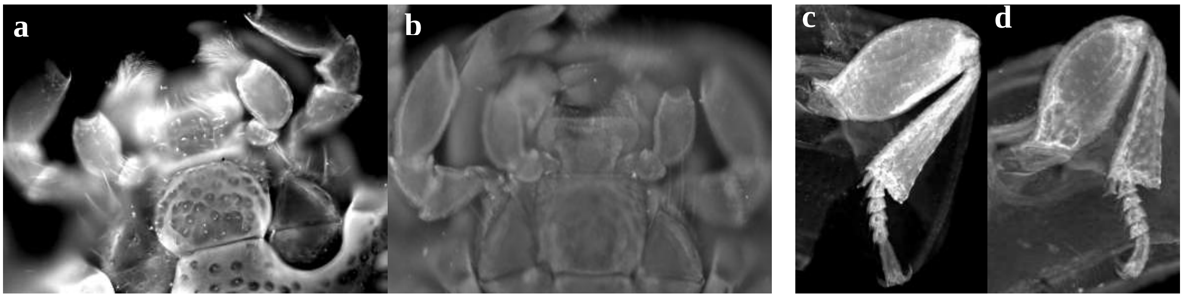

***Tcas*Gr105**

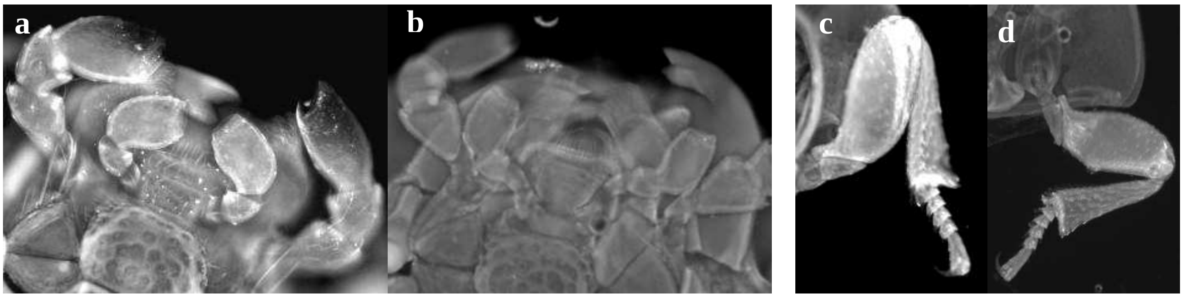

***Tcas*Gr37**

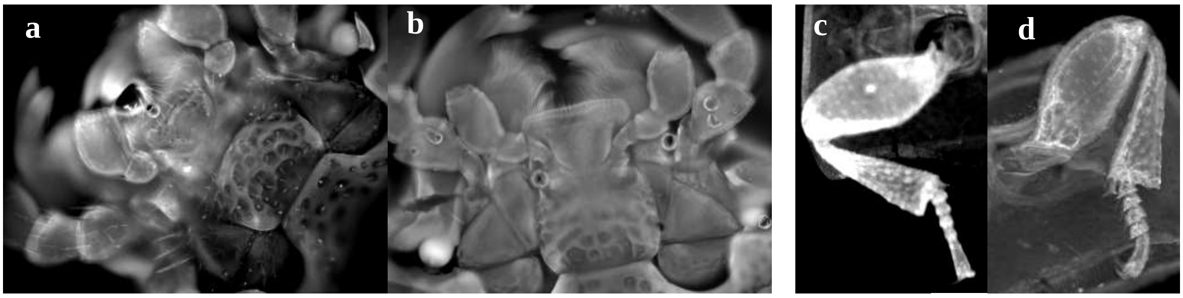

***Tcas*Gr38**

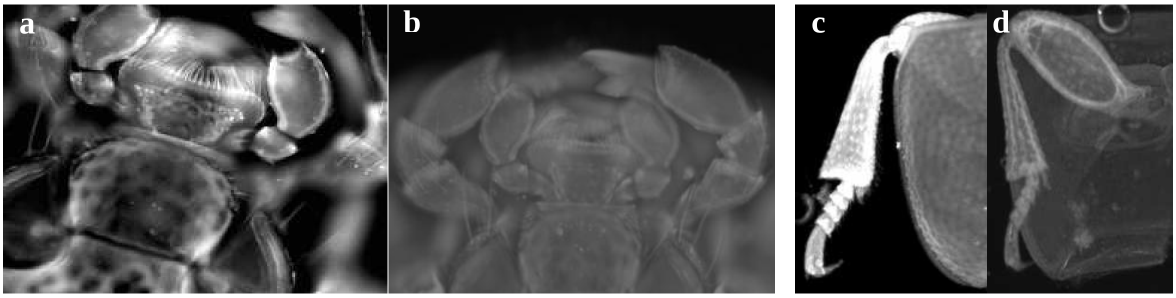

***Tcas*Gr39**

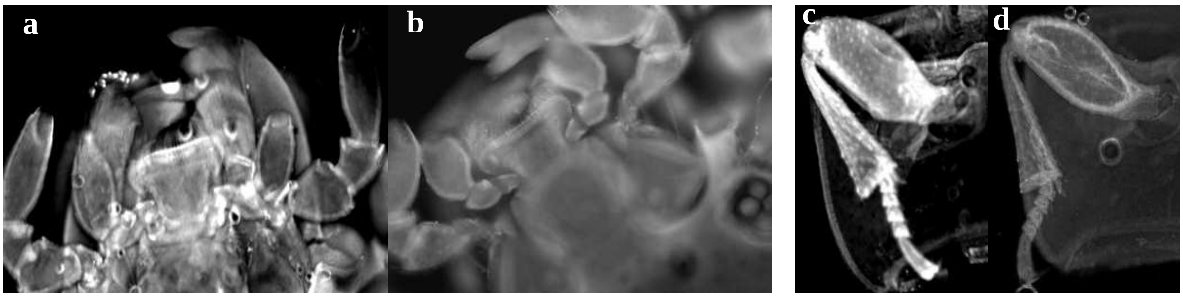

***Tcas*Gr40**

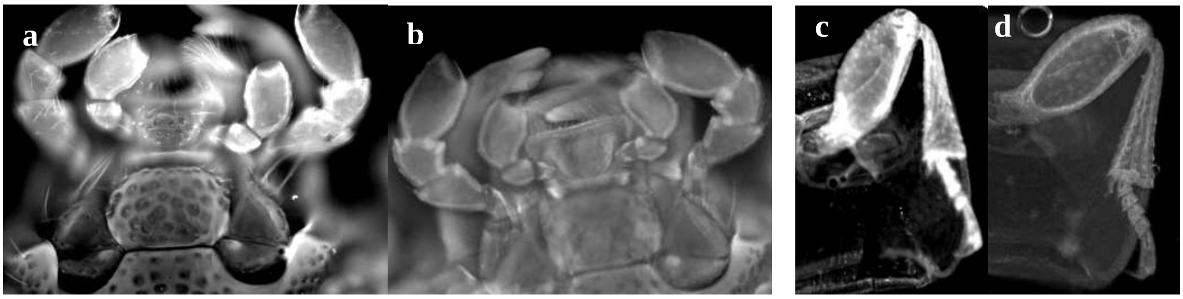

***Tcas*Gr41**

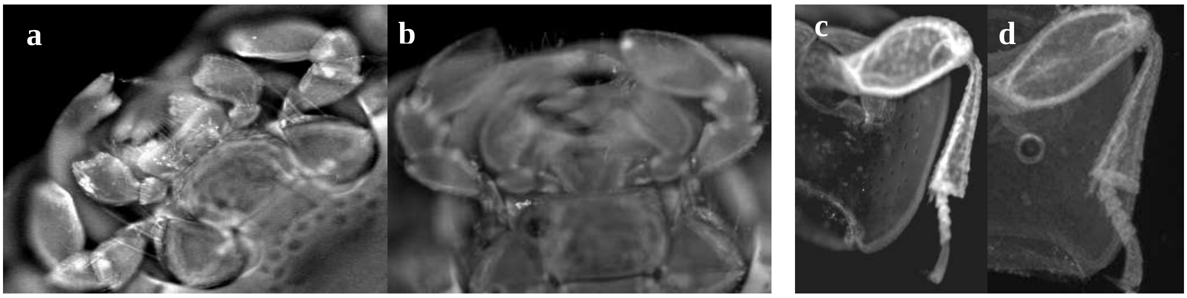

***Tcas*Gr71**

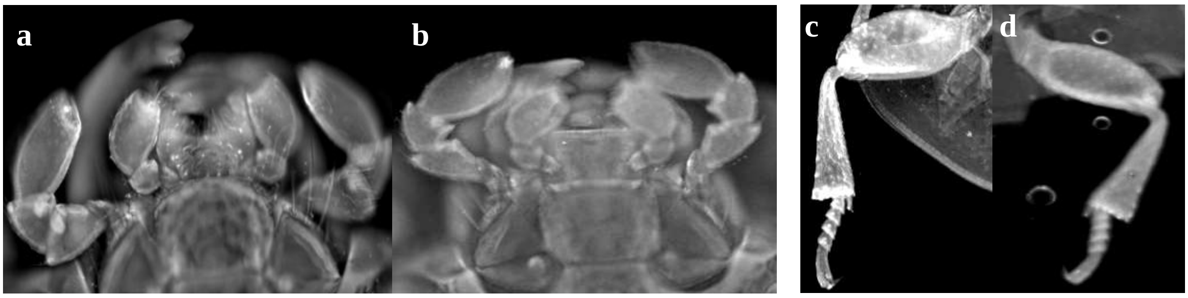

***Tcas*Gr43**

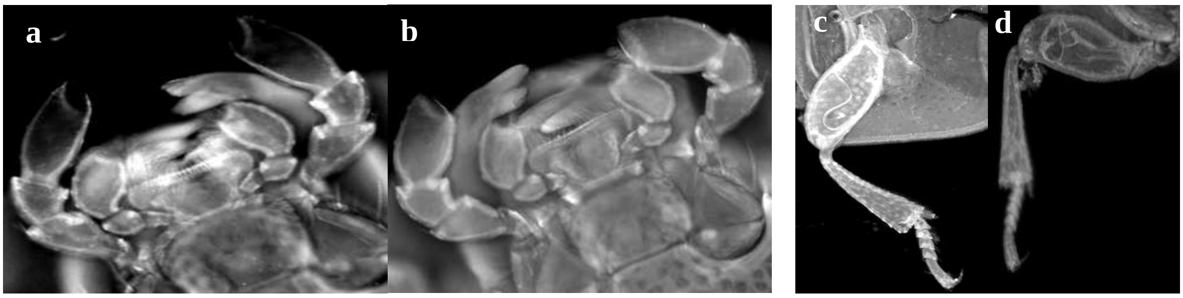

***Tcas*Gr44**

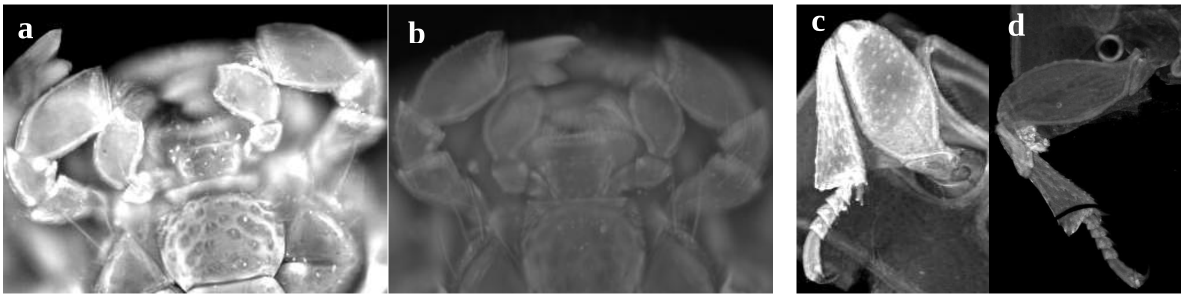

***Tcas*Gr45**

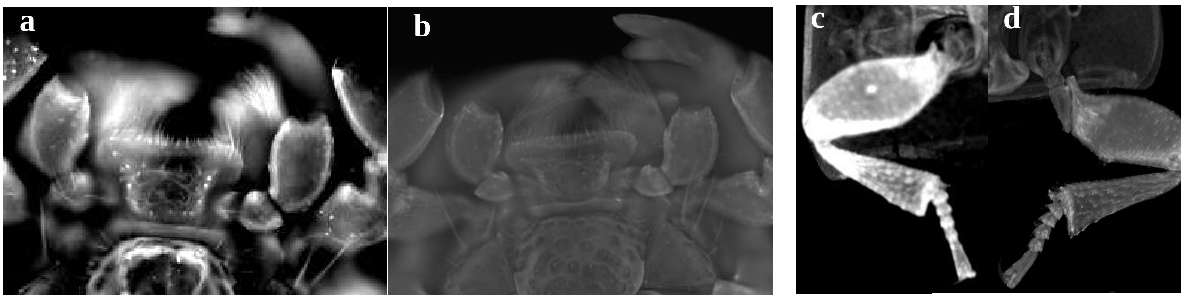

***Tcas*Gr46**

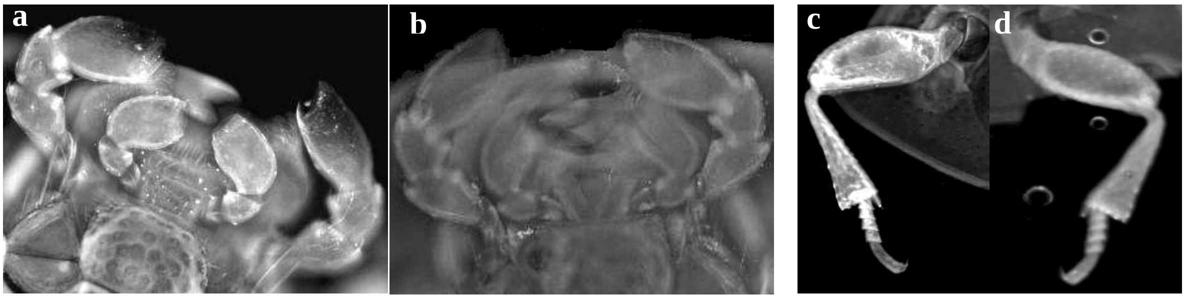

***Tcas*Gr33**

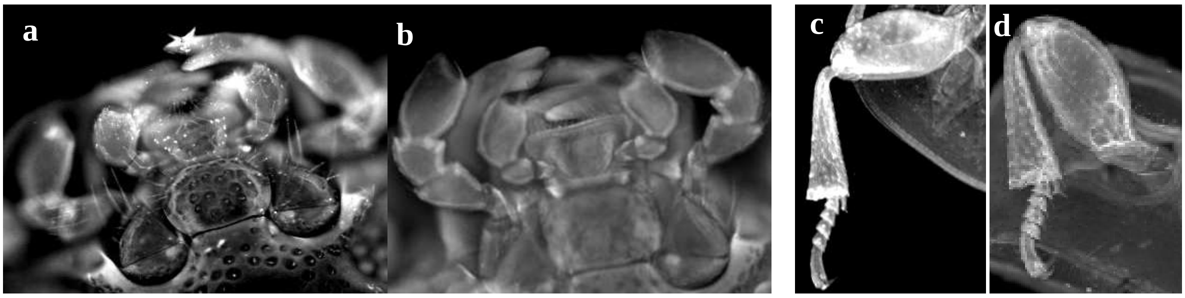

***Tcas*Gr48**

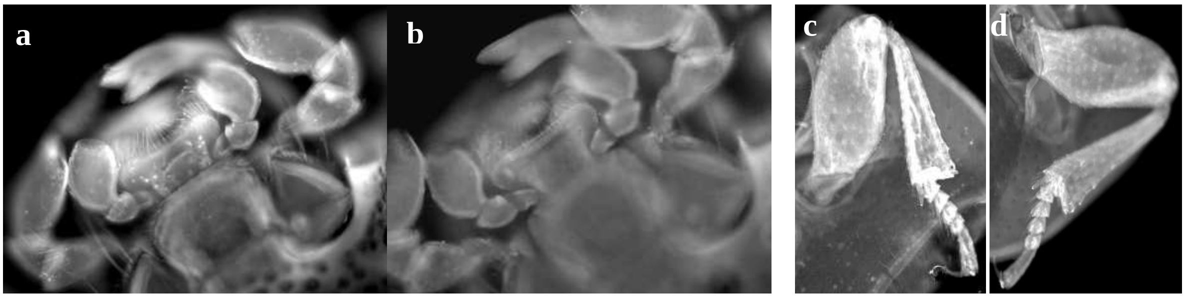

***Tcas*Gr49**

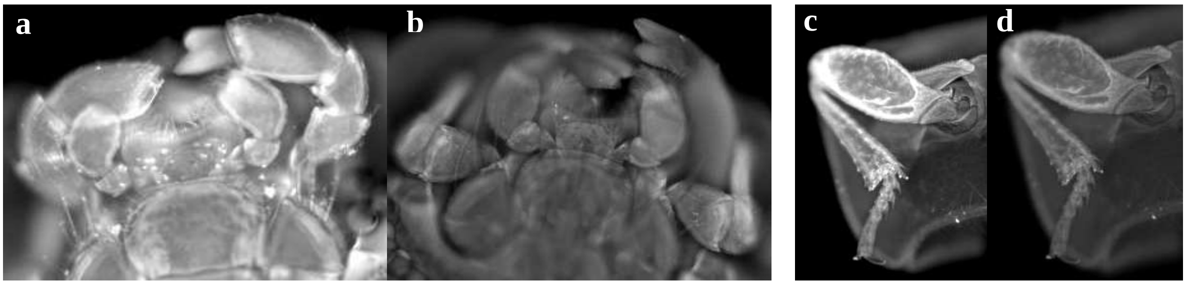

***Tcas*Gr50**

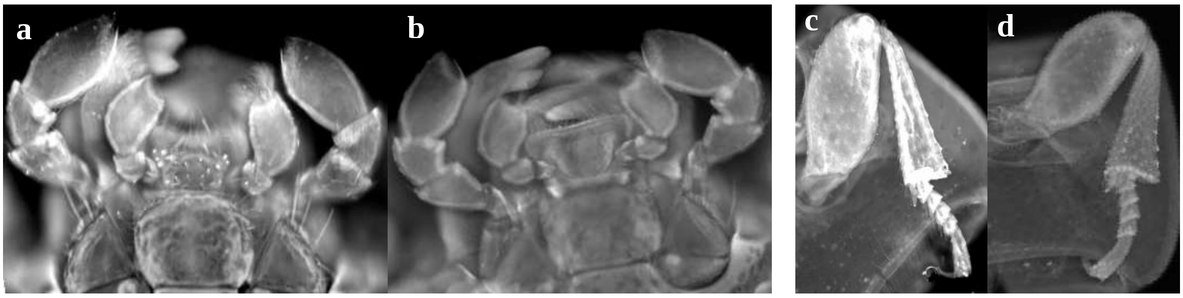

*Tcas*Gr51

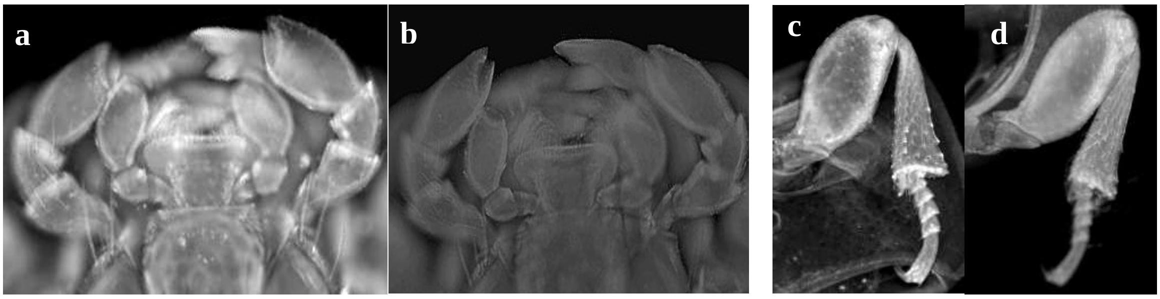

*Tcas*Gr52

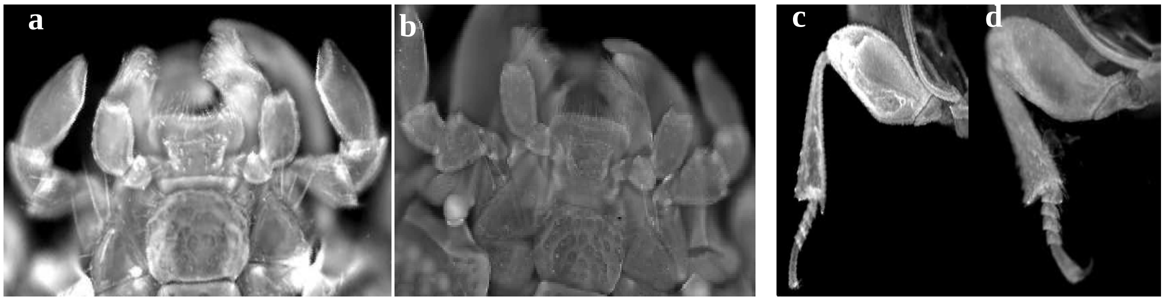

*Tcas*Gr53

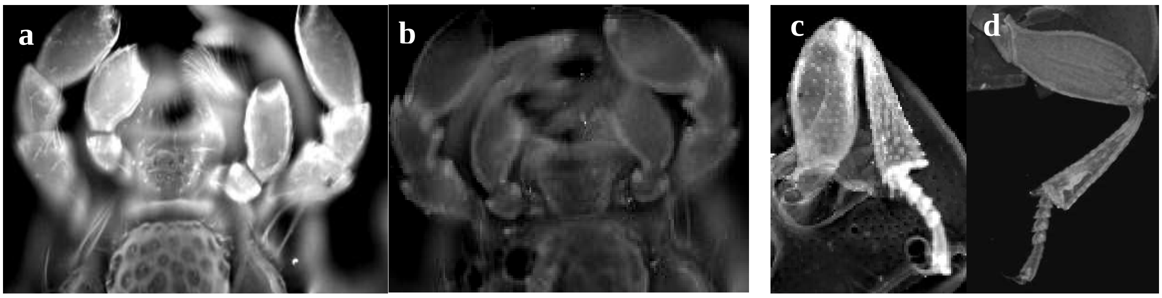

*Tcas*Gr54

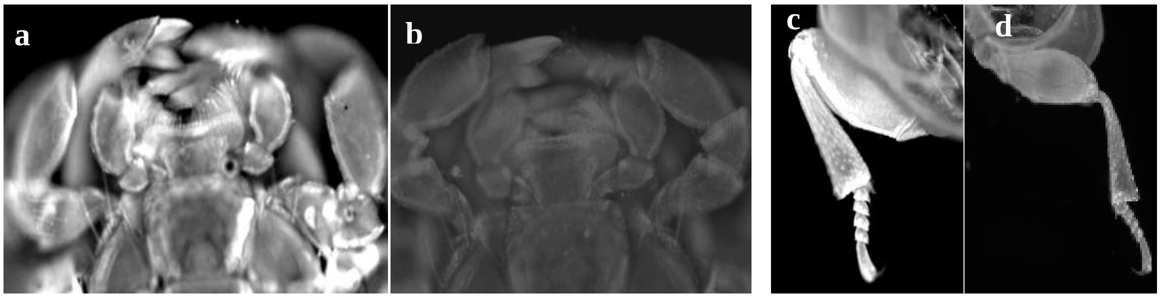

*Tcas*Gr125

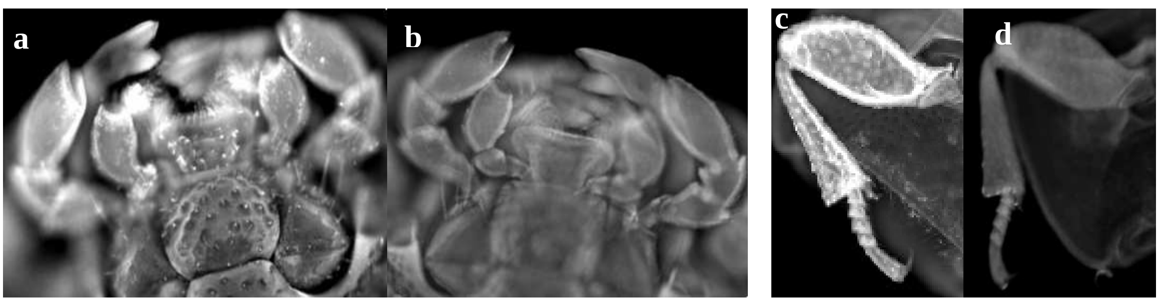

*Tcas*Gr56

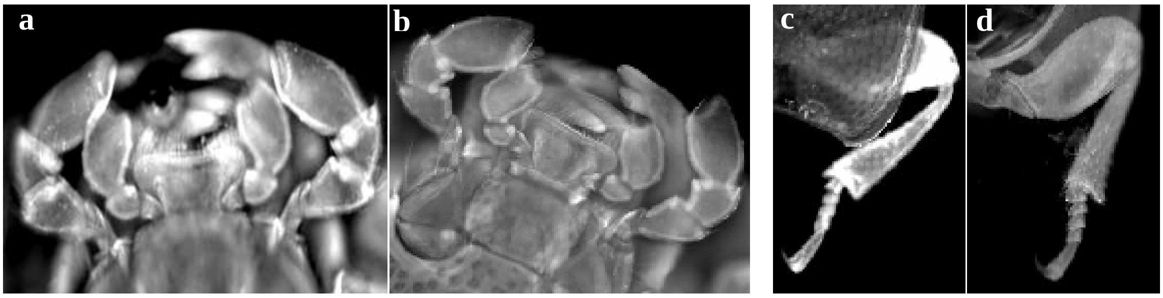

*Tcas*Gr57

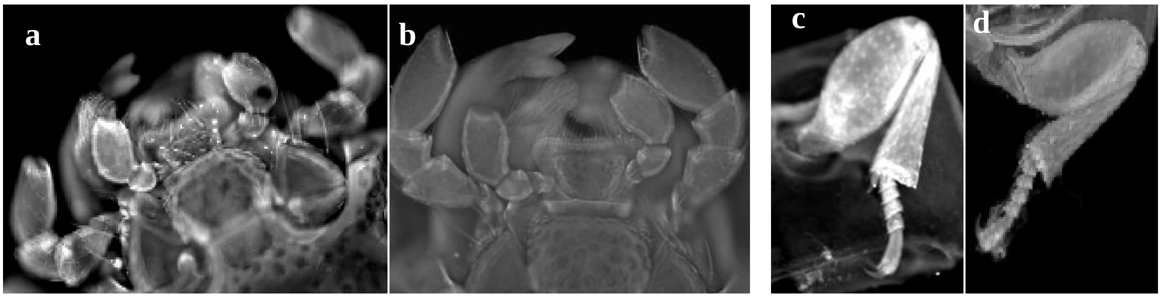

*Tcas*Gr98

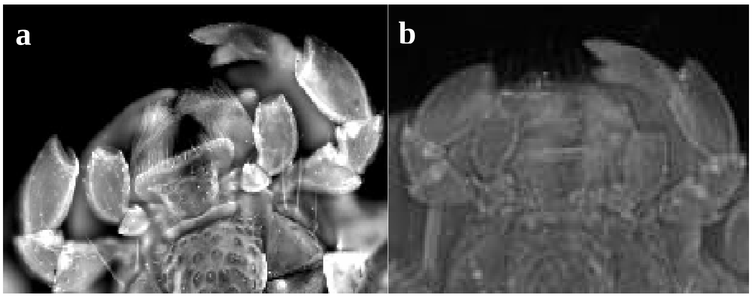

*Tcas*Gr59

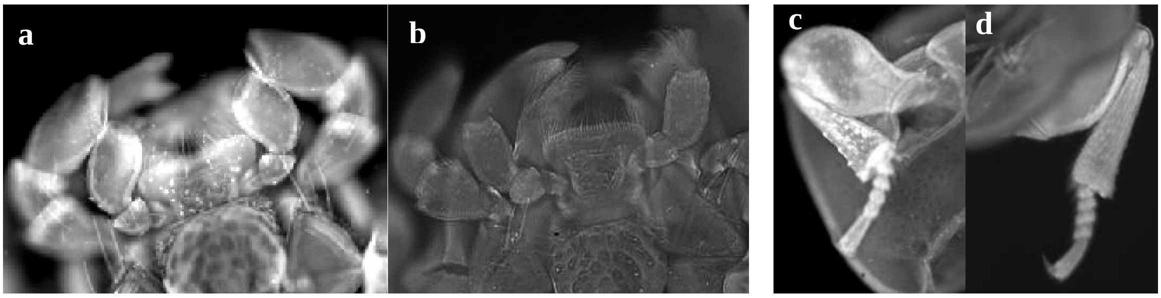

*Tcas*Gr60

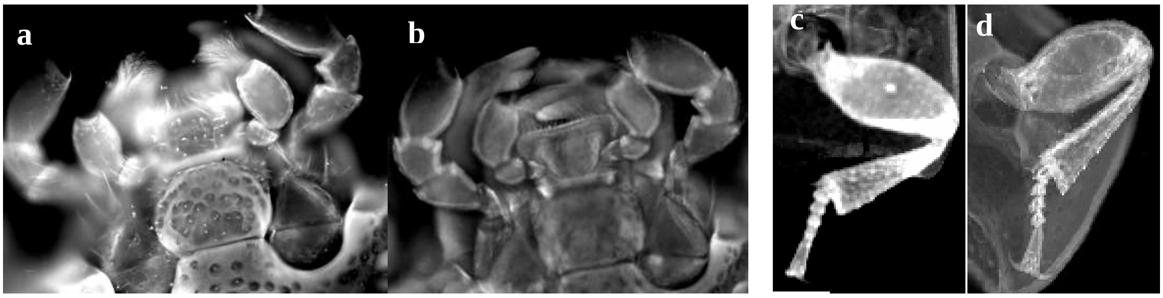

***Tcas*Gr61**

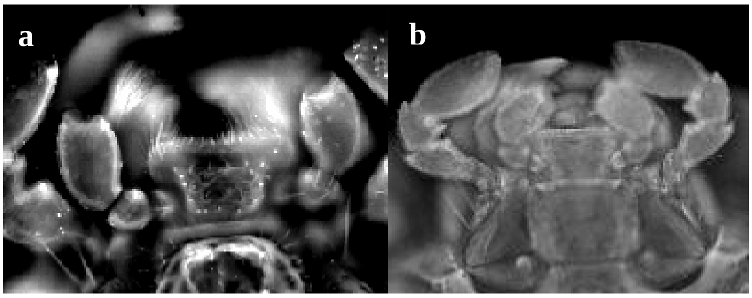

***Tcas*Gr62**

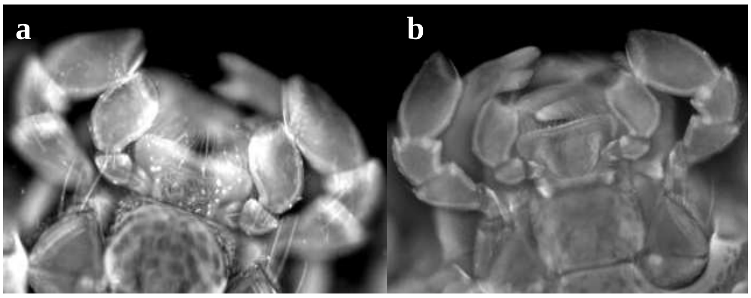

Figure S6.
